# Volatilomics of raspberry fruit germplasm by combining chromatographic and direct-injection mass spectrometric techniques

**DOI:** 10.3389/fmolb.2023.1155564

**Published:** 2023-04-13

**Authors:** Brian Farneti, Iuliia Khomenko, Matteo Ajelli, Karen Elizabeth Wells, Emanuela Betta, Eugenio Aprea, Lara Giongo, Franco Biasioli

**Affiliations:** ^1^ Berries Genetics and Breeding Unit, Research and Innovation Centre of Fondazione Edmund Mach, Trento, Italy; ^2^ Sensory Quality Unit, Research and Innovation Centre of Fondazione Edmund Mach, Trento, Italy; ^3^ Center Agriculture Food Environment C3A, University of Trento, Trento, Italy

**Keywords:** *Rubus idaeus L.*, PTR-ToF-MS, SPME-GC-MS, germplasm, VOCs, phenotyping, quality

## Abstract

The application of direct-injection mass spectrometric (DI-MS) techniques, like Proton Transfer Reaction Time of Flight Mass Spectrometry (PTR-ToF-MS) has been suggested as a reliable phenotyping tool for fruit volatilome assessment in both genetic and quality-related studies. In this study the complexity of raspberry aroma was investigated by a comprehensive untargeted VOC analysis, done by combining SPME-GC-MS and PTR-ToF-MS assessments with multi-block discriminant analysis using the DIABLO mixOmics framework. The aim was to acquire an exhaustive characterization of the raspberry volatilome according to different fruit ripening stages (pink, ripe, and overripe) and genetic variances (50 accessions), as well as to investigate the potential of PTR-ToF-MS as a rapid and high throughput VOC phenotyping tool to address issues related to raspberry fruit quality. Results of this study demonstrated the complementarity between SPME-GC-MS and PTR-ToF-MS techniques to evaluate the raspberry aroma composition. PTR-ToF-MS generates reliable raspberry VOC fingerprints mainly due to a reduced compound fragmentation and precise content estimation. In addition, the high collinearity between isomers of monoterpenes and norisoprenoids, discovered by GC analysis, reduces the main analytic limitation of PTR-ToF-MS of not being able to separate isomeric molecules. The high similarity between the VOC matrices obtained by applying PTR-ToF-MS and SPME-GC-MS confirmed the possibility of using PTR-ToF-MS as a reliable high throughput phenotyping tool for raspberry volatiolome assessment. In addition, results provided by the germplasm collection investigation enabled to distinguish the best performing accessions, based on VOCs composition, to be used as superior parental lines for future breeding programs.

## 1 Introduction

Volatile organic compound (VOC) directly related to odour composition is one of the main attributes that determines consumers’ overall liking of foods compared to other sensory attributes, such as appearance and texture (Klee and Tieman, 2018; Lytou et al., 2019). Therefore, several VOCs are considered key target molecules to improve the flavour perception of fruit and vegetables and, consequently, their marketability. In addition, the end of the “flavour life”, mainly due to changes in VOC composition, often precedes the end of shelf life as determined by external modifications (Mazzucotelli et al., 2022). Thus, VOCs should be considered a central trait to determine the optimal cultivation and storage strategies, especially for products for which repeated purchasing behavior and willingness to pay are associated with positive eating experience.

High priority should be given to replace poor flavour cultivars with favorable ones, exploiting the available natural variability. However, the VOC assessment of a very high number of samples, which is necessary to overcome the usually massive biological and genetic variability among fruit samples, may be laborious and time consuming. The so-called “phenotyping bottleneck”, caused by the absence of high-throughput and non-invasive methodologies, hampers an effective VOC assessment of broad plant collections (Mazzucotelli et al., 2022)). In fact, the use of conventional gas chromatographic (GC) techniques for VOC analysis presents many analytical limitations. First of all, the process of sample preparation and analysis is time consuming, and second, there are limitations of running exhaustive, complex experimental designs due to the length of analysis. The application of direct-injection mass spectrometric (DI-MS) techniques, like Proton Transfer Reaction Time of Flight Mass Spectrometry (PTR-ToF-MS) or Selected Ion Flow Tube Mass Spectrometry (SIFT-MS), has been demonstrated to be a powerful phenotyping tool for destructive and non-destructive fruit volatilome assessment in both genetic and quality-related studies (Farneti et al., 2017a; Farneti et al., 2020; Di Guardo et al., 2021; Li et al., 2021). Depending on the analytical technique, volatilome assessment can contribute to the discrimination of samples in clusters (Farneti et al., 2017a; Farneti et al., 2020; Di Guardo et al., 2021), the identification of specific biomarkers that are crucial for quality, and the prediction of aspects associated with food quality (Farneti et al., 2013; Bianchi et al., 2020; Li et al., 2021).

VOCs play a key role in the formation of the well-recognized and commonly appreciated aroma of soft fruits (Du and Qian, 2010). In particular, aroma is one of main factors impacting raspberry (*Rubus idaeus* L.) fruit quality and consumer appreciation (Valdés García et al., 2020; Zhang et al., 2021). Raspberry VOC profile of distinct genotypes can be qualitatively and quantitatively very different, and it can be influenced by both agronomic practices and microorganism interactions (Durán-Soria et al., 2021; Sangiorgio et al., 2021; Sangiorgio et al., 2022). Raspberry aroma is a complex blend of almost 300 VOCs with major classes of compounds being terpenes, C13-norisoprenoids, esters, alcohols, aldehydes acids, and ketones (Aprea et al., 2015). However only few of these VOCs have been recognized as major aroma-active compounds in raspberry (Larsen et al., 1991; Aaby et al., 2019; Zhang et al., 2021). Among them, Zhang et al. (2021) identified 14 major aroma-active compounds (volatiles with OAVs ≥1): namely, hexanal, (Z)-3-hexenal, (E)-2-hexenal, eucalyptol, (Z)-3-hexen-1-ol, 1-octen-3-ol, linalool, benzyl alcohol, theaspirane, *ß*-damascenone, dihydro-β-ionone, *a*-ionone, *ß*-ionone, and naphthalene. Additionally, several VOCs, such as benzaldehyde, 1-hexanol, 2-nonanone, (E)-2-hexenal, and (Z)-3-hexenol and monoterpenes, are reported to have inhibitive effects against fungi, especially against *Botrytis cinerea* (Vaughn et al., 1993; Aprea et al., 2010).

Improving fruit flavour, selecting cultivars with premium characters, provides unique challenges in raspberry breeding (Paterson et al., 2013). Selection oriented mostly on productive traits, such as yield, size and shelf-life, have had negative effects on fruit flavour for several fruit species during domestication (Aharoni et al., 2004; Goff and Klee, 2006; Farneti et al., 2017a). Therefore, precise methodological strategies are necessary to support the raspberry breeding activity. This needs a greater comprehension of the genetic control of the pathways involved in VOCs synthesis, environmental factors influencing VOCs production and contributions of impact VOCs to sensory character in raspberry.

In this study the complexity of raspberry aroma was explored by an exhaustive untargeted volatilomic analysis, done by combining SPME/GC-MS and PTR-ToF-MS assessments with multivariate statistic models. The aim of this study was to obtain a thorough characterization of the raspberry volatilome according to different fruit ripening stages and genetic differences, as well as to investigate the potential of PTR-ToF-MS as a rapid and high throughput VOC phenotyping tool to address issues related to raspberry fruit quality.

## 2 Material and methods

### 2.1 Plant material and fruit sampling

Raspberry (*R. idaeus* L.) accessions (Supplementary Table S1) were chosen from the experimental field of Fondazione Edmund Mach (FEM) Research and Innovation Center at Pergine (Trento), located in the north of Italy (Trentino Alto Adige region- 46.0744°N, 11,2334°E, 525 m a.s.l.). Plants of raspberry were all grown in 7 L pots, under a hail net and were maintained following standard pruning and agronomical practices (Giongo et al., 2019). Berries were harvested manually at the required stage for each experiment early in the morning and brought to the laboratory within 30 min after picking. Homogeneous fruit, free from external damages or irregularities, were immediately frozen with liquid nitrogen.

For the first experiment, we aimed at acquiring a comprehensive untargeted characterization of the raspberry volatilome according to fruit ripening stages. We employed six raspberry cultivars, namely, “Citria,” “Glen Ericht,” “Himbotop,” “Kweli”, “Paris”, and “Tulameen”. Fruit was collected at three ripening stages based on visual colour evaluation (Pink -P, Ripe-R, and Overripe -OR; Monsalve et al., 2022. “OR” ripening stage corresponds, approximately, to 24 h harvest delay from “R” ripening stage) and analysed by SPME/GC-MS and PTR-ToF-MS.

For the second experiment, aimed at: i) testing the analytical capacity of PTR-ToF-MS as a rapid and high throughput VOC phenotyping tool, and ii) estimating the genetic variability among raspberry cultivars, we employed 50 accessions of raspberry (34 cultivars and 16 advance selections from the FEM raspberry breeding program; Supplementary Table S1). Fruit was harvested at full ripe stage, which corresponds to the OR commercial maturity stage described above.

### 2.2 Sample preparation for VOC analysis

Replicates of 0.5 g of raspberry frozen grinded samples, stored at −80°C, were weighed into 20 mL glass vials with screw cap with PTFE/silicone septum (Agilent, Cernusco sul Naviglio, Italy). 0.5 mL of an antioxidant solution (0.5 mL of deionized water, 200 mg of sodium chloride, 2.5 mg of ascorbic acid, and 2.5 mg of citric acid) was added on top according to Farneti et al., 2017b.

### 2.3 VOC analysis by SPME/GC-MS

Gas chromatographic analysis was performed according to Farneti et al., 2017b. The vials were incubated at 40°C for 10 min constantly stirring before being analysed. Solid-phase microextraction fiber (DVB/CAR/PDMS, 2 cm coating Supelco, Bellefonte, PA, United States) was exposed for 30 min in the vial headspace. Compounds adsorbed by HS-SPME were analysed with a GC interfaced with a mass detector operating in electron ionization (EI) mode (internal ionization source; 70 eV) with a scan range of m/z 33–350 (GC Clarus 500, PerkinElmer, Norwalk CT, United States). HP-INNOWax fused silica capillary column (30 m, 0.32-mm ID, 0.5-μm film thickness; Agilent Technologies, Palo Alto, CA, United States) was used for separation. The initial GC oven temperature was 40°C rising to 220°C at 4°C min^−1^, the temperature of 220°C was maintained for 1 min, then increased at 10°C min^−1^ until it reached 250°C, which was maintained for 1 min. Helium as a carrier gas was kept at a constant column flow rate of 1.5 mL min^−1^. Samples were analysed in triplicates. The content of each compound was expressed as μg L^−1^ equivalent of 2-octanol. Compound identification was based on mass spectra matching with the standard NIST/EPA/NIH (NIST 14) and Wiley 7th Mass Spectral Libraries, and linear retention indices (LRI) compared with the literature. LRI were calculated under the same chromatographic conditions after injection of a C7–C30 n-alkane series (Supelco).

### 2.4 VOC analysis by PTR-ToF–MS

Measurements were performed with a commercial PTR-ToF–MS 8000 apparatus (Ionicon Analytik GmbH, Innsbruck, Austria; Farneti et al., 2017b). The drift tube conditions were as follows: 110°C drift tube temperature, 2.3 mbar drift pressure, 550 V drift voltage with E/N ratio of about 140 Townsend (Td), with E corresponding to the electric field strength and N to the gas number density (1 Td = 10–17 Vcm^2^). The acquisition rate of ToF mass spectrometer was 1 spectrum s^-1^ with a mass spectrum ranging up to *m/z* = 400. The sample headspace was drawn into PTR-MS inlet with 40 sccm flow for 1 min. Flushing of a vial with pure nitrogen during sampling prevented pressure drop inside it. 20 min of sample incubation at 40°C, 1 min of measurement and waiting for 2 min between each measurement were automated by an adapted GC autosampler (MPS Multipurpose Sampler, GERSTEL) coupled to PTR-ToF-MS. The analysis of PTR-ToF-MS spectra proceeded as described in Farneti et al., 2017b.

### 2.5 Data and statistical analysis

The list of mass peaks detected with PTR-ToF-MS was reduced by applying noise and correlation coefficient thresholds. The first step removed peaks not significantly different from blank samples; the second excluded peaks with over 99% correlation, corresponding for the most part to isotopes of monoisotopic mass peaks (Farneti et al., 2017b).

The internal statistical functions of R.3.4.1. (R Foundation for Statistical Computing, Vienna, Austria) and the external packages “mixOmics”, and “ggplot2” were used for the multivariate statistical methods applied in this study.

## 3 Results and discussion

### 3.1 Raspberry VOC modification during fruit ripening

#### 3.1.1 SPME-GC-MS headspace analysis

The gas chromatographic analysis by SPME/GC-MS assessed on the headspace of six raspberry cultivars (“Citria,” “Glen Ericht,” “Himbotop,” “Kweli”, “Paris”, and “Tulameen”) harvested at different ripening stages (pink, ripe, over ripe) allowed the detection of 96 VOCs, among which seven were not identified (reported as “Unknown”; Supplementary Table S2). Aldehydes and monoterpenes were the most represented chemical classes, with 16 identified compounds for each chemical class. The other classes of compounds detected in raspberry fruit are alcohols (11 compounds), ketones (11), norisoprenoids (9), esters (8), acids (6), lactones (4), sesquiterpenes (3), hydrocarbons (2), sulfur (1), and furans (1). Based on VOC relative concentration, the ratio between VOC classes differed both regarding fruit ripening and cultivars. Similar to most other fruit species, the production of volatiles in raspberry is integrated with the ripening process with volatile profiles changing dramatically during ripening.

**FIGURE 1:**
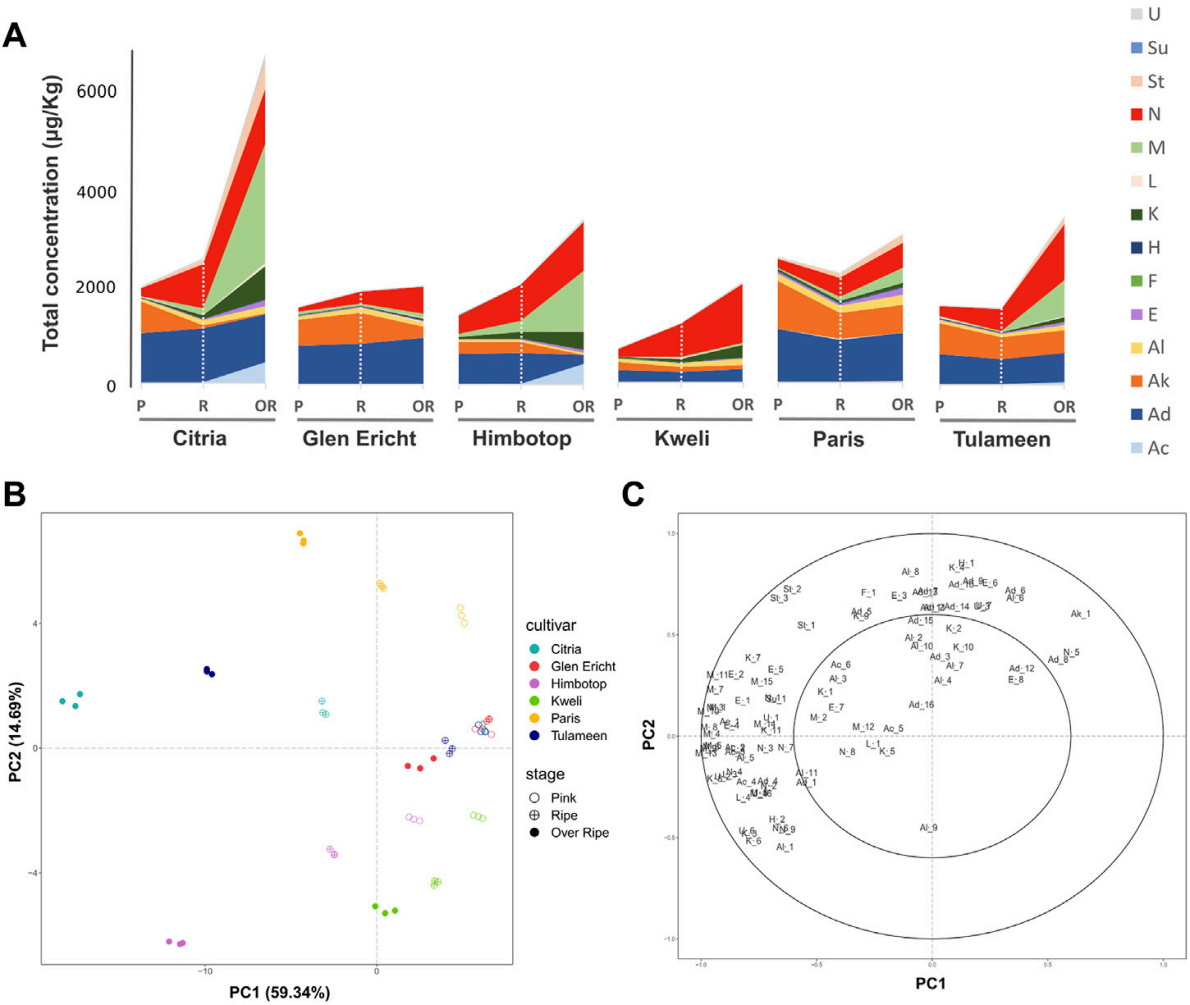
Evolution of raspberry VOC profile during fruit ripening assessed by SPME/GC-MS analysis. The stacked area chart reported in the plot **(A)** illustrates the total VOC concentration, expressed as μg/L of 2-octanol, for each raspberry cultivar (“Citria”, “Glen Ericht”, “Himbotop”, “Kweli”, “Paris”, and “Tulameen”) at three ripening stages [pink (P), ripe (R), and overripe (Or)]. Each VOC classes [acids (Ac), aldehydes (Ad), alkenes (Ak), alcohols (Al), esters (E), furans (F), hydrocarbons (H), ketones (K), lactones (L), monoterpenes (M), norisoprenoids (N), sesquiterpenes (St), sulfurs (Su), unknowns (U)] is described with a different color. Plot **(B)** depicts the VOC profile of the raspberry cultivars during fruit ripening over the PCA score plot defined by the first two principal components. Plot **(C)** shows the PCA loading plot of the VOCs identified by SPME/GC-MS analysis.

During the complete fruit ripening process, an important VOC profile portion is covered by monoterpenes, norisoprenoids and ketones, while for pink fruit around 70% of the VOC profile is covered by aldehydes and alkenes (Figure 1A). These modifications are strictly cultivar dependent. Indeed, the relative abundance of ketones, monoterpenes, norisoprenoids and sesquiterpenes raised to more than 70% of the overall VOC profile for the cultivars “Citria”, “Himbotop”, “Kweli” and “Tulameen”, while fruit of “Gleen Ericht” and “Paris” did not show a so drastic increment (Figure 1A; Supplementary Figure S1).

Most of the identified VOCs significantly differed both between cultivars and especially between ripening stages (Supplementary Table S2; Supplementary Figure S2). Only few compounds decreased during maturation, like 3-methyl-2-hexene and (E)-2-hexenal. Compounds that were unaffected by the ripening stage of the fruit are (E)-2-hexenal, octanal, 2-heptanol, (Z)-3-hexen-1-ol, 2-nonanal, 1-heptanol, 2-ethyl-1-hexanol, decanal, benzaldehyde, undecanal, non-anoic acid. In contrast, the concentration of most other compounds increased during ripening especially in fully ripe fruit (OR).

**FIGURE 2:**
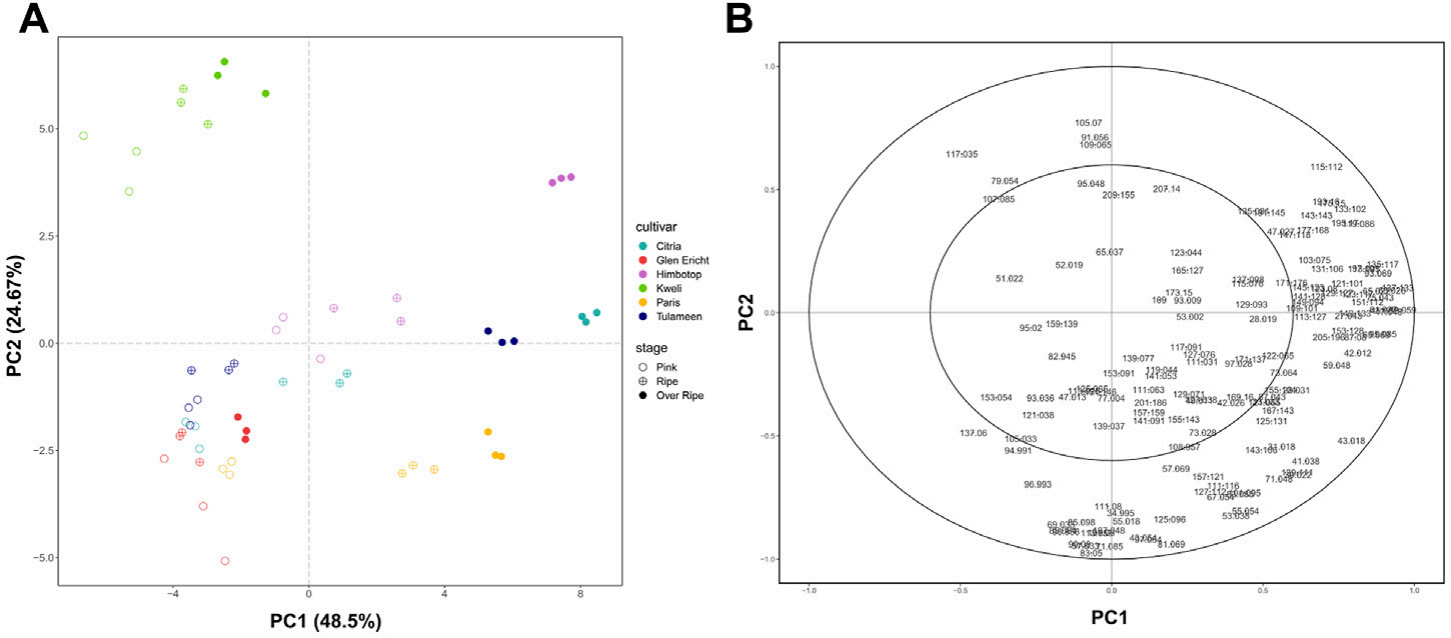
Evolution of raspberry VOC profile during fruit ripening assessed by PTR-ToF-MS analysis Plot **(A)** depicts the VOC profile of each raspberry cultivar (“Citria”, “Glen Ericht”, “Himbotop”, “Kweli”, “Paris”, and “Tulameen”) at three ripening stages [pink (P), ripe (R), and overripe (Or)] over the PCA score plotdefined by the first two principal components. Plot **(B)** shows the PCA loading plot of the VOCs identified by PTR-ToF-MS analysis.

For a rapid and unsupervised data exploration, SPME/GC-MS results were analysed with principal component analysis (PCA). The biplot of the first two PC scores and the loading plot are presented in Figures 1B, C. These VOC profile evolution during fruit ripening is evident in the PCA reported in Figure 2B where nearly 60% of the variability among the fruit VOC profiles (PC1) is strictly associated with the stage of fruit ripening. The variability expressed by the second component (PC2: 15%) is more inherent to differences between accessions. According with these PCA results, the greatest differences among cultivars are detectable among fruits at the most advanced stage of ripening (OR). These differences are mostly related to different concentrations of monoterpenes, norisoprenoids, ketones, sesquiterpenes, and acetate esters (Figure 2C).

The results obtained on these six cultivars support previously published findings on ripening studies performed with different analytical techniques on other raspberry cultivars (Guichard, 1984; Larsen et al., 1991). Guichard (et al., 1984) followed the VOC evolution during ripening in two raspberry cultivars (*R. idaeus* cv. “Lloyd George” and “Rose de Côte d’Or”) by analysing the fruit concentrated extracts by gas chromatography. In both cultivars all the terpenes and sesquiterpenes significantly increased during ripening. However, dissimilarly from our results, the ripe and overripe stages were not significantly different. Several acetate esters, like isopentyl-, pentenyl-, (Z)-3-hexenyl- and methyl-acetate, also increased up to 100 fold during ripening. Norisoprenoids had different trends according to the cultivar. Dihydro-β-ionone was at its highest at the ripe stage then decreased. *a*-Ionone moderately increased during fruit ripening in both varieties, while *ß*-ionone reasonably increased only in fruit of the cultivar “Lloyd George” and not at all in “Rose de Côte d’Or”. Larsen and co-workers (1991) compared the VOC evolution during the ripening of 10 cultivars (cv. “Camenzind”, “Chilcotin”, “Glen Prosen”, “Glen Moy”, “Glen Clova”, “Meeker”, “Rutrago”, “Skeena”, “Vaten” and “Zenith”). Concentrated extracts have been analysed using GC with FID detector. They reported relatively great differences in the concentrations of linalool, geraniol, benzyl alcohol, acetoin, acetic acid, and hexanoic acid during ripening. However, only very tiny differences in norisoprenoid concentrations were observed between the cultivars.

Terpenes, terpenoids and norisoprenoid volatile compounds are the major compounds that have been detected for the differentiation of raspberry genotypes (Larsen et al., 1991; Malowicki et al., 2008) as they are highly related to raspberry odour and flavour. The increase of monoterpenes (i.e., *a*-pinene, *a*-phellanderene, *ß*-Phellandrene, o-Cymene, 4-Terpineol) and sesquiterpenes (i.e., trans-Caryophyllene and *a*-Humulene) highlighted by our results can be associated with a different regulation of the isopentenyl diphosphate (IDP) and its isomer dimethylallyl diphosphate (DMADP) synthesis (Paterson et al., 2013; Pazouki and Niinemets, 2016). The formation of both IDP and DMADP is led by the mevalonate (MVA) and the methylerythritol 4-phosphate (MEP) pathways that are active, respectively, in the cytosol and in the plastid of fruit cells. It is widely acknowledged that monoterpenes are synthesized in the plastids whereas sesquiterpenes in the cytosol (Rohmer, 2003). The total content of terpenes and sesquiterpenes among different cultivars, in fact, did not show any significant correlation in our study (Supplementary Figure S3).

Most of the norisoprenoids detected in this study increased during fruit ripening, similarly to monoterpenes. Only damascenone, detected at very low concentration, slightly decreased during fruit ripening. Norisoprenoids, recognized to be important contributors to raspberry fruit aroma, are generated by oxidative cleavage of the carotenoids (Hampel et al., 2007). In particular, the two ionone stereoisomers, *a*-Ionone and *ß*-ionone, are responsible for raspberry-violet-rose fragrance notes (Breitmaier, 2006). These compounds are respectively derived from the degradation of *a* and *ß*-carotene (Paterson et al., 2013). This could be the reason why their content is not correlated in our study (Supplementary Figure S3). Unidentified compounds 4 and 5, based on their fragmentation spectrum (Supplementary Figure S4) and their high correlation with cycloionone (r^2^:0.98; Supplementary Figure S2), can be classified as norisoprenoids with high probability. Other norisoprenoids highly correlated with cycloionone are *ß*-ionone (r^2^:0.89) and dihydro- *ß*-ionone (r^2^:0.85). The high variability in norisoprenoid content between raspberry cultivars is in agreement with Malowicki et al. (2008) and Paterson et al. (2013), both of which reportedlarge variations in *a*-ionone, *ß*-ionone in different raspberry genotypes.

Ketones are other important compounds for characterizing the aromatic profile of raspberries, particularly 2-nonanone, 2-heptanone, 2-undecanone, and 5-nonen-2-one are responsible for a “fruity” and “cheesy” flavour of fruit (http://www.thegoodscentscompany.com/). The concentration of these molecules increased during ripening, depending on the considered cultivar. The cultivars with higher concentrations of ketones are “Citria”, “Himbotop” and “Kweli”. As also reported previously by Aprea et al. (2009), it was not possible to quantify one of the most characteristic raspberry ketone, raspberry ketone (4-(4-hydroxyphenyl)butan-2-one), since it is not easily detectable without a chemical extraction because of its low volatility.

#### 3.1.2 Direct injection VOC profiling by PTR-ToF-MS

Fruit samples analysed by PTR-ToF-MS were prepared in the same way to the ones used for SPME/GC-MS analysis in order to compare the outcomes of these two methodologies. The whole VOC spectra, assessed in triplicate for each cultivar, were reduced to 148 VOC mass peaks (Supplementary Table S3; Supplementary Figure S5), applying noise and correlation coefficient thresholds. Tentative identification (t.i.) of each mass peak detected by PTR-ToF-MS relied on an in-house library of chemical standards, on the list of compounds detected by SPME/GC-MS analysis, and on compounds reported in the review paper of Aprea et al. (2015). The content of 146 mass peaks was significant different between cultivars, while 112 mass peaks significantly differed between ripening stages (Supplementary Table S3). Among them, only few mass peaks decrease during fruit ripening. These mass peaks, like *m/z* 99.080, *m/z* 81.069, *m/z* 57.033, or *m/z* 43.054, are related to the fragmentation of several c6 aldehydes. The headspace analyses carried out with PTR-ToF-MS allow for the valuation of VOCs that are often omitted from the ordinary gas chromatographic assessments despite their importance for the characterization of fruit quality and freshness, such as methanol (*m/z* 33.033), ethanol (*m/z* 47.049), acetaldehyde (*m/z* 45.033), or dimethyl sulfide (*m/z* 63.026).

PCA analysis (Figure 2) was carried out to describe differences amongst the raspberry VOC profiles considering both cultivars and ripening stages. 73% of the total variation accounted for the first two principal components (Figure 2A). Likewise in the SPME/GC-MS analysis, differences between fruit sampled at different ripening stages were largely accountable to the first principal component (PC1: 48.5%), while the second component (PC2: 24.7%) predominantly defined differences between cultivars. Based on the correlation circle plot obtained from the PCA analysis, only few mass peaks have a weak association (lower than 0.6) with the first two principal components (Figure 2B). These mass peaks are either present at trace concentration levels or with a non-significant (*p* > 0.05) ‘cultivar x stage’ interaction (Supplementary Tablee S3). In accordance with SPME/GC-MS analysis, the greatest differences among cultivars are detectable among fruit at the most advanced stage of ripening (OR). These differences between VOC profiles are mainly related to the different concentrations of mass peaks related to monoterpenes (i.e., *m/z* 137.133), norisoprenoids (i.e., *m/z* 191.145, *m/z* 193.16, *m/z* 195.17), sesquiterpenes (i.e., *m/z* 205.196), ketones (i.e., *m/z* 115.112, *m/z* 141.128, *m/z* 143.143), acetate esters (i.e., *m/z* 75.043, *m/z* 89.059), aldehydes (i.e., *m/z* 45.032, *m/z* 73.064, m*/z* 85.064, *m/z* 87.08, *m/z* 95.085, *m/z* 99.08), and alcohols (i.e., *m/z* 33.033, *m/z* 47.048, *m/z* 83.085). These results agree with the studies of Aprea et al. (2009); Cappellin et al. (2013), in which the VOC profile of some raspberry cultivars was evaluated on intact berries with PTR-MS equipped with a quadrupole mass detector. The main advantages of using PTR-MS equipped with a ToF analyzer are the enhanced mass resolution, allowing the separation of many isobaric compounds and the simultaneous monitoring of multiple peaks at the same nominal mass, and the enhanced speed of analysis since the whole VOC spectrum is acquired in a fraction of a second (Mazzucotelli et al., 2022).

### 3.2 Complementarity between SPME/GC-MS and PTR-ToF-MS headspace VOC assessments

**FIGURE 3:**
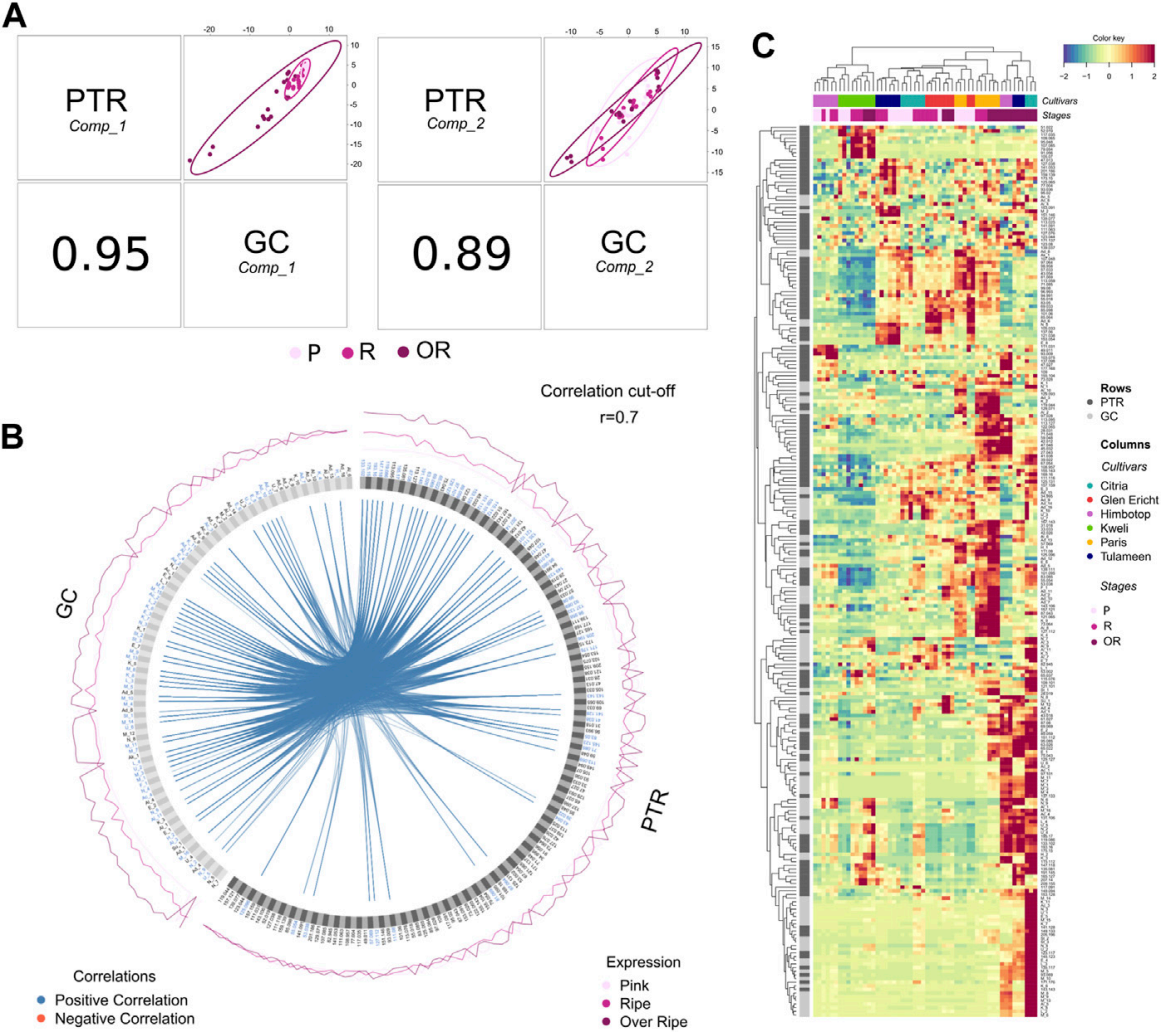
Complementarity between SPME/GC-MS and PTR-ToF-MS headspace VOC assessments defined by applying the DIABLO multi-block discriminant analysis. Plot **(A)** depicts a global overview of the correlation structure at the component level. This function allows for the plotting of the components (component 1 and component 2) across the different data sets for a given dimension. Plot **(B)** reports the correlation circular plot between VOCs assessed by SPME-GC-MS and PTR-ToF-MS built based on a similarity matrix. A cut-off level of 0.7 was arbitrarily included to visualize correlation coefficients above this threshold. Plot **(C)** reports the “Clustered Image Map” representing the multi-omics molecular signature expression for each sample. Each cell’s colour is based on the values of the similarity matrix performed on the two VOC datasets.

The results obtained with the two analytical techniques were compared and combined in order to verify both the complementarity of the two methodologies and the possibility of using PTR-ToF-MS as a fast, comprehensive and reliable VOC phenotyping tool for raspberry fruit, despite its known analytical limitations in separating and identifying isomers. In order to identify a highly correlated VOC signature discriminating known groups of samples (cultivar and/or ripening stages), we applied a multi-block discriminant analysis using the DIABLO mixOmics framework [Data Integration Analysis for Biomarker discovery using a Latent cOmponents (Singh et al., 2019)]. The core DIABLO method extends Generalised Canonical Correlation Analysis, which generalises PLS for multiple matching datasets, and the sparse sGCCA method (Singh et al., 2019). Like PLS, DIABLO generates a pair of components, each associated to each VOC data set. A global overview of the correlation structure at the component level is represented in Figure 3A by using the plotDiablo function. This function allows for the plotting of the components across the different data sets for a given dimension. The results revealed a high correlation between the data obtained by SPME/GC-MS and PTR-ToF-MS, resulting in a correlation of 0.95 and 0.89 for the first and second components, respectively. As reported by previous PCA analyses (Figures 1, 2), values of the first component are more associated with differences in VOC profile due to fruit maturity stages, while in the second component there are more associations with differences between cultivars.

The high similarity between the two matrices is explainable by the great correlation between the content of individual molecules acquired by the two analytical techniques. This result confirms the possibility of using a direct injection mass spectrometry technique (PTR-ToF-MS) for raspberry flavour profile analysis as an alternative to gas chromatographic analysis performed after headspace accumulation (SPME/GC-MS). All the correlations between VOCs assessed by SPME-GC-MS and PTR-ToF-MS are reported in the circos plot (Figure 3B), built based on a similarity matrix. A cut-off level of 0.7 was arbitrarily included to visualize correlation coefficients above this threshold in the multi-omics signature. 60% of the compounds identified and quantified by SPME/GC-MS (58 out of 96) are highly correlated (cut-off of 0.7) with the masses quantified by PTR-ToF-MS (Figure 3B). In contrast, only 32% of the masses considered from the PTR-ToF-MS analyses (48 out of 148) are correlated with the compounds identified by the gas chromatographic analyses. Of these 100 masses without any significant correlation with the gas chromatographic matrix, only a few are present at high concentrations: e.g., *m/z* 33.033 (t.i. methanol), *m/z* 34.995 (t.i. hydrogen sulfide), *m/z* 45.032 (t.i. acetaldehyde), *m/z* 47.048 (t.i. ethanol), *m/z* 107.085 (t.i. ethylbenezene, xylene). These compounds, in fact, cannot be properly identified with the setting of the gas chromatographic methodology applied in this study. Most of the other unrelated masses can be associated with molecules present in raspberry fruit at low concentrations and thus below the LOD of the SPME-GC-MS methodology that we applied. For this reason, the tentative identification of many masses measured by PTR-ToF-MS is based on information extrapolated from published studies about raspberry volatilome assessed by applying different analytical techniques (Aprea et al., 2015).

**FIGURE 4:**
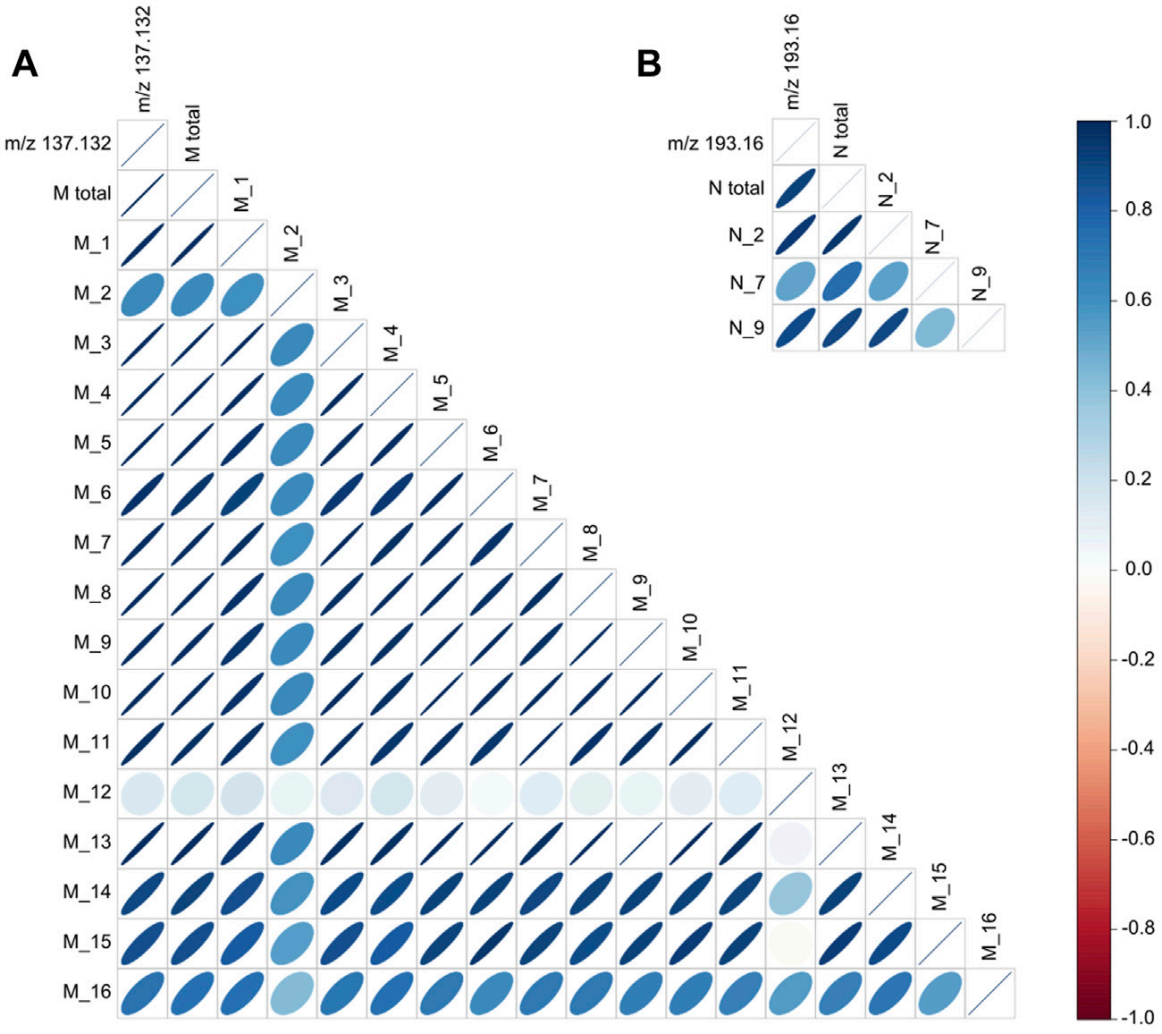
Correlation analysis of monoterpenes and norisoprenoids assessed by using SPME/GC-MS and PTR-ToF-MS. Plot **(A)** reports the correlation analysis between the content of the main PTR-ToF-MS mass peak associated with monoterpenes (*m/z* 137.132), the total amount of monoterpenes (“M total”), and the content of each monoterpene compounds (From M_1 to M_16) detected with SPME-GC-MS. Plot **(B)** reports the correlation analysis between the content of the main PTR-ToF-MS mass peak associated with norisoprenoids (*m/z* 193.16), the total amount of norisoprenoids associated with this mass (“N total”), and the content of each norisoprenoid compounds associated with the mass peak m/z 193.16 (N_2, N_7, and N_9) detected with SPME-GC-MS.

One of the analytical limitations of direct injection mass spectrometry techniques, and thus also of PTR-ToF-MS, is the difficulty in separating and identifying isomers. The VOC profile of raspberry consists of several isomeric compounds important for the aromatic characterization of the fruit, especially in the chemical class of monoterpenes (e.g., *a*-pinene, *a*-phellanderene, *ß*-phellanderene) or norisoprenoids (e.g., *a*-ionone, *ß*-ionone, cycloionone; *ß*-damascenone). As for monoterpenes, the content of *m/z* 137.133 mass is highly correlated with the total monoterpene content (*R* ^2^ = 0.99; Figure 4A). However, unexpectedly, the monoterpenes present in raspberry fruit are highly correlated with each other, except for linalool and geraniol, suggesting a very similar biosynthetic pathway regulation of monoterpenes among the raspberry accessions considered in this study. This high collinearity among monoterpenes reduces the limitation of PTR-ToF-MS of not being able to separate isomeric molecules, at least for the raspberry aroma profile. As for the three main norisoprenoids of raspberry fruit, namely, cycloionone, *a*-ionone and *ß*-ionone, their total content is positively correlated with the mass *m/z* 193.16 (*R* ^2^ = 0.84, Figure 4B). Unlike what was found with monoterpenes, the correlation is less robust mainly due to the lack of collinearity between the concentrations of *a*-ionone compared to *ß*-ionone and cycloionone. *ß* and *a* ionone are derived from the degradation of *a* and *ß*-carotene (Paterson et al., 2013), respectively. This lack of collinearity between the compounds could result from different carotenoid composition and content among raspberry cultivars.

The collinearity and complementarity of the two analytical techniques used in this study is shown graphically in the Clustered Image Map in Figure 3C. The Clustered Image Map represent the multi-omics molecular signature expression for each sample. Each cell’s colour is based on the values of the similarity matrix performed on the two VOC datasets. Dendrograms used along the axes depict how each row (VOCs)/column (cultivars and ripening stages) clusters based on the hierarchical clustering method. Blocks of homogeneous colour depict subsets of features from each dataset which are correlated and suggests a potential causative relationship.

### 3.3 Phenotyping of raspberry germplasm volatilome

In this trial carried out to both test the applicability of PTR-ToF-MS as a fast phenotyping tool for volatile profiling of raspberry fruit and to verify the variability present within raspberry germplasm, we analysed 50 raspberry accessions (Supplementary Table S1), some of which were collected at different ripening epochs during the season. Of these 50 accessions, 34 were cultivars and 16 were advanced selections from the FEM’s breeding program. The whole VOC spectra, assessed in triplicate for each sample, were reduced to 136 VOC mass peaks (Supplementary Table S4), applying noise and correlation coefficient thresholds. The number of mass peaks significantly different from the blank sample is higher than in the previous experiment, most likely because the number of accessions measured is higher and consequently so is the biological variability.

**FIGURE 5:**
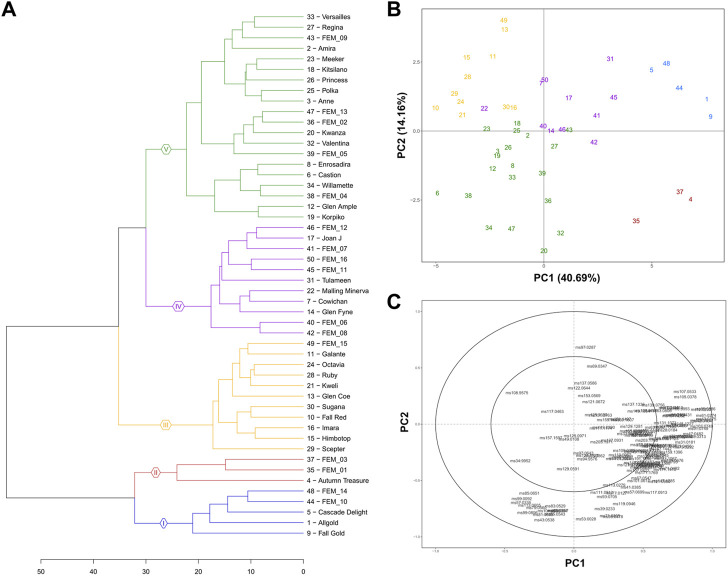
Raspberry germplasm volatilome assessed by PTR-ToF-MS. Hierarchical dendrogram **(A)** principal component analysis (PCA) **(B)** and loading plot **(C)** of VOC profiles of 50 raspberry accessions measured by PTR-ToF-MS. Each VOC concentration is the average of three biological replicates. Each colour of the PCA plot depicts one of the five clusters, determined by gap statistics.

Considering the elevated genetic variability of the germplasm collection employed in this study, we aimed to uncover most of the raspberry natural VOC variability. To avoid any possible statistical bias in the interpretation of results, all data were analysed with unsupervised multivariate statistical methodologies (PCA and hierarchical clustering). Based on the VOCS profile, the 50 accessions were divided into five significantly different clusters (gap statistic) Figure 5A. These five clusters are also distinguishable in the PCA (Figures 5B, C) performed on the raspberry VOC variability defined by the first two PCs, and expressing together 55% of the total variability. According to the correlation loading plot (Figure 5C), the first principal component (PC1), describing 40.7% of the total variability, mainly correlates with VOC concentration magnitude. The second principal component (PC2), instead, resulted mainly related with the VOC chemical composition, describing 14.2% of total aromatic variability. Clusters I and II, in fact, group the 8 accessions with the most intense VOC profile (cluster I: “Fall Gold”, “Allgold”, “Cascade delight”, “FEM_10”, “FEM_14”; cluster II: “Autumn Treasure”, “FEM_01”, “FEM_03”). These two clusters are separated from the remaining 3 clusters (III, IV, V) more by PC1 values. Within cluster V we detected a group of raspberry accessions (“Kwanza”, “Valentina”, “FEM_13”, “Willamette”, “FEM_16”, “FEM_04”, “FEM_05”) that differs from the others by a higher concentration of masses connected with C6 aldehydes (*m/z* 81.069, *m/z* 99.08, *m/z* 101.095) and C6 alcohols (*m/z* 83.085).

**FIGURE 6:**
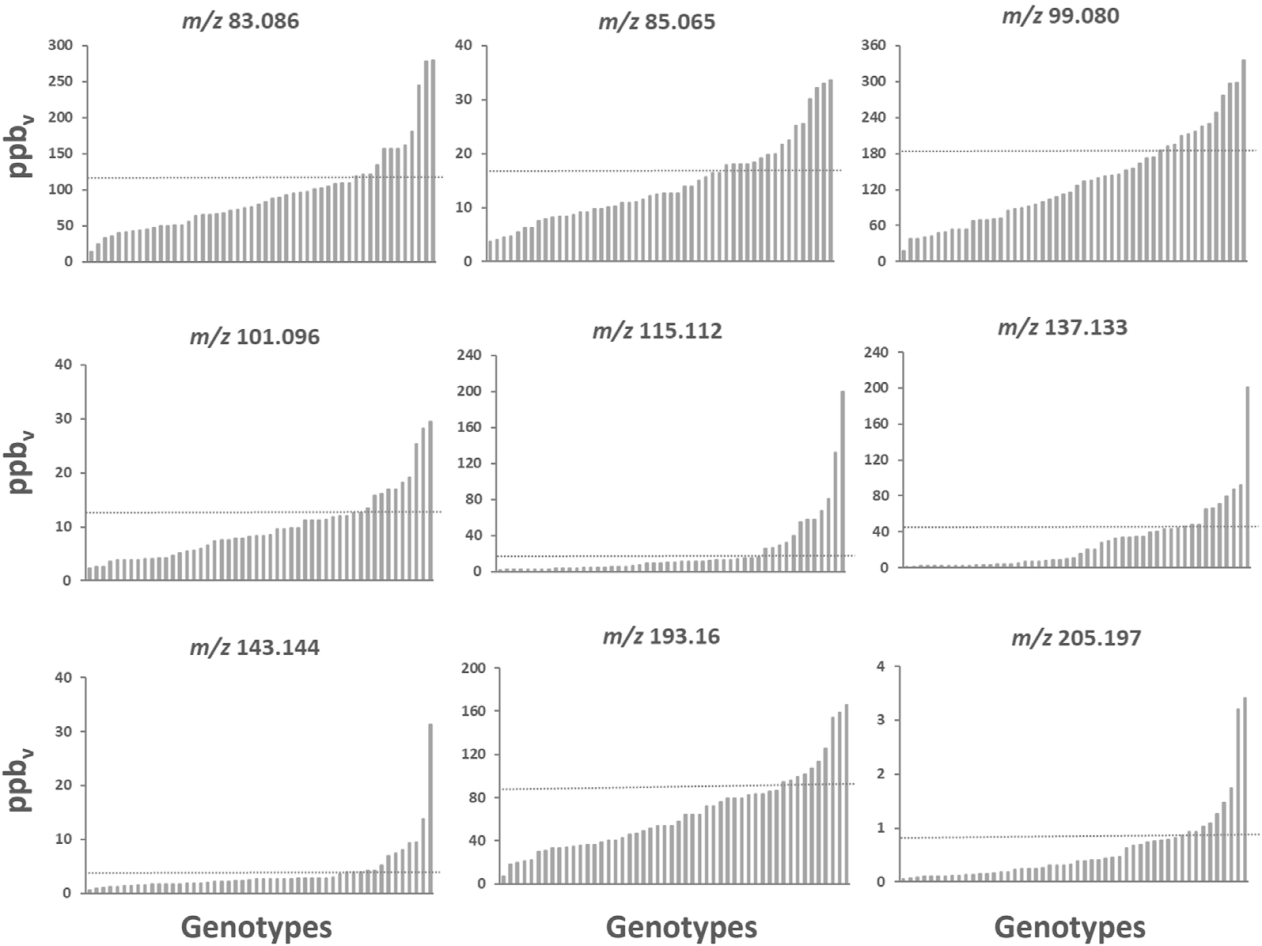
Bar chart plots of the main raspberry VOCs assessed by PTR-ToF-MS in the raspberry germplasm collection. Each bar illustrates the average value of three biological replicates. In each graph, accessions were ordered based on the VOC mass peak concentration. The 75th percentile levels are represented by the dashed line. Bar chart plots of all mass peaks with the corresponding complete names of the accessions are reported in the Supplementary Figure S6.

Considering the whole VOC profile, although useful for getting a general indication of the possible aromatic fingerprint of a genotype, can still limit the information actually present in the dataset. In particular, we can see that a high statistical weight in the analysis of the PCA of the various accessions is given by molecules present at very high concentrations, two or three orders of magnitude higher than the other compounds, that are, however, not closely related to the characteristic raspberry aromatic profile such as methanol, ethanol or acetaldehyde. However, these molecules are an indicator of the degree of anaerobic fermentation and are often associated with off-flavours, and therefore can be considered as possible biomarkers related to fruit ripeness and shelf life (Beaulieu et al., 1997). Instead, to identify possible biomarkers closely related to the raspberry flavour profile to be considered in qualitative “from farm to fork” studies in our opinion, it is more explanatory to consider the concentration of each mass individually. Figure 6 shows 9 masses (over 136) that can be considered as key elements to describe the raspberry aroma profile according to the loading plots of the principal component analysis and to the results of previously published articles on raspberry aroma (Aprea et al., 2015; Zhang et al., 2021). High variability was found for each mass within the genotype pool analysed in this study.

Non-etheless, considering each VOC independently (i.e., Supplementary Figure S6) might be useful for the backcross breeding approach, aimed to introduce, or improve, a distinct quality trait to an elite breeding line (Hospital, 2005).

For a more practical application of these results, especially for breeding purposes, the content of each VOC was grouped based on the distribution quantile (low: 0%–25%; middle-low: 25%–50%; middle-high: 50%–75%; high: 75%–100%), calculated for each compound (Supplementary Figure S7). Therefore, all accessions employed in the study can be sorted and clustered according to each VOC mass peak content, which can be arbitrarily chosen, as implemented in the dedicated webpage https://iuliiakhomenko-fmach.shinyapps.io/QualySort_raspberry/.

Considering that no molecular markers are yet available to predict the VOC content of raspberry fruit, the application of reliable phenotyping techniques combined with an array of VOC biomarkers is still essential to support breeding activity.

## 4 Conclusion

Results of this study confirmed the complementarity between chromatographic and direct-injection spectrometric techniques to study the raspberry aroma composition. The use of PTR-ToF-MS is suitable to generate reliable raspberry VOC fingerprints mainly due to a reduced compound fragmentation and accurate quantification. The high similarity between the VOC matrices obtained by applying PTR-ToF-MS and SPME/GC-MS confirmed the possibility of using a direct injection mass-spectrometry technique (PTR-ToF-MS) as a reliable VOC phenotyping tool in those investigations that require a detailed VOC profile characterization of a large number of raspberry fruit.

A weak aspect of DI-MS methodologies is still represented by compound identification. PTR-ToF-MS can separate many raspberry isobaric compounds; however, many isomers are still not distinguishable without a chromatographic separation. Nevertheless, unexpectedly, most monoterpene and norisoprenoid isomers, important for the raspberry aroma characterization, in this study were highly correlated with each other, suggesting a very similar and solid biosynthetic pathway regulation of both compound classes among the raspberry accessions. This high collinearity, in our opinion, overcomes the main analytic limitation of PTR-ToF-MS of not being able to separate isomeric molecules.

Bearing in mind that the aim of VOC assessment in quality related studies is to obtain an objective estimation of the aroma perceived by the consumer during fruit consumption, we consider unnecessary the application of overly aggressive chemical extraction methodologies (commonly used in several published articles on raspberry VOC profiling). These methodologies are necessary for the quantification of compounds at extremely low concentrations that, in case of raspberry fruit, might be under the perception threshold of the consumer. In addition, results of our study revealed a higher sensitivity of PTR-ToF-MS with respect to SPME/GC-MS, allowing the detection of compounds present in trace amounts.

Pulling together results of the investigations about the role of ripening and of the genetic variability, the array of mass peaks suitable to describe most of raspberry VOC variability can be considerably reduced. This array of VOC biomarkers, in combination with a reliable phenotyping methodology, can be applied for a more targeted VOC assessment for both breeding selection and quality control within the entire production chain. The uncovering of the genetic variability existing within the investigated raspberry germplasm collection allowed us to identify the best performing cultivars, based on VOCs content, to be used as superior parental lines for future breeding programs focused on enhanced fruit quality. In our opinion, a better and more detailed knowledge of the aromatic profile of the fruit is also essential to define the optimal production and storage strategies specific for each genotype. In the case of raspberry, it is evident how anticipated fruit harvesting, which is the common practice to prolong fruit shelf life, may drastically reduce the organoleptic quality of the fruit. These findings should push the forthcoming research activity toward the development of cultivation and storage techniques tailored for enhanced organoleptic quality of the fruit and not just for the external visual quality.

## Data Availability

The original contributions presented in the study are included in the article/Supplementary Material, further inquiries can be directed to the corresponding author.

## Supplementary material

SUPPLEMENTARY FIGURE S1 Stacked area chart of the relative VOC concentration, expressed as %, for each raspberry cultivar (“Citria,” “Glen Ericht,” “Himbotop,” “Kweli,” “Paris,” and “Tulameen”) at three ripening stages [pink (P), ripe (R), and overripe (Or)]. Each VOC classes [acids (Ac), aldehydes (Ad), alkenes (Ak), alcohols (Al), esters (E), furans (F), hydrocarbons (H), ketones (K), lactones (L), monoterpenes (M), norisoprenoids (N), sesquiterpenes (St), sulfurs (Su), unknowns (U)] is described with a different color.

SUPPLEMENTARY FIGURE S2 Bar graph of the content of each VOC classes, expressed as μg/Kg of 2-octanol, assessed by SPME-GC-MS for each raspberry cultivar (“Citria,” “Glen Ericht,” “Himbotop,” “Kweli,” “Paris,” and “Tulameen”) at three ripening stages [pink (P), ripe (R), and overripe (Or)].

SUPPLEMENTARY FIGURE S3 Correlation plot of volatile organic compounds assessed by SPME/GC-MS.

SUPPLEMENTARY FIGURE S4 Mass spectra of unknown compounds detected by SPME/GC-MS.

SUPPLEMENTARY FIGURE S5 Bar graph of the content of each VOC, expressed as ppbv, assessed by PTR-ToF-MS for each raspberry cultivar (“Citria,” “Glen Ericht,” “Himbotop,” “Kweli,” “Paris,” and “Tulameen”) at three ripening stages [pink (P), ripe (R), and overripe (Or)]. Each value is the average plus standard deviation of three replicates. For each graph lines corresponding to the 25%, 50%, and 75% percentile were added.

SUPPLEMENTARY FIGURE S6 Bar graph of the content of each VOC, expressed as ppbv, assessed by PTR-ToF-MS for each raspberry accession of the raspberry germplasm collection. Each value is the average plus standard deviation of three replicates.

SUPPLEMENTARY FIGURE S7 Print screens of the Shiny web application (Qualysort 1.2; Farneti et al. 2020) with an examples of selected VOC traits (same compounds reported in Figure 6). This Shiny web application (https://iuliiakhomenko-fmach.shinyapps.io/QualySort/) is interactive and gives the opportunity to choose an xlsx file with the dataset of phenotypic traits. It is possible to select the raspberry accessions and the VOCs. Each VOC is classified according to the quartile it belongs to (0–25%, red colour; 25–50%, orange colour; 50–75%, light green colour; 75–100% dark green colour) for each type separately. Button “Calculate” plots the heatmap based on the selected classified data with the hierarchical clustering (Gower's distance and clustering method—average) by samples (rows).
