# Supplementary material for: Volatilomics of raspberry fruit germplasm by combining chromatographic and direct-injection mass spectrometric techniques
Source: Front Mol Biosci. 2023 Apr 13;10:1155564. doi: 10.3389/fmolb.2023.1155564 (PMC10133483; doi:10.3389/fmolb.2023.1155564)
Supplement: Supplementary file 3 [file Image5.PDF]

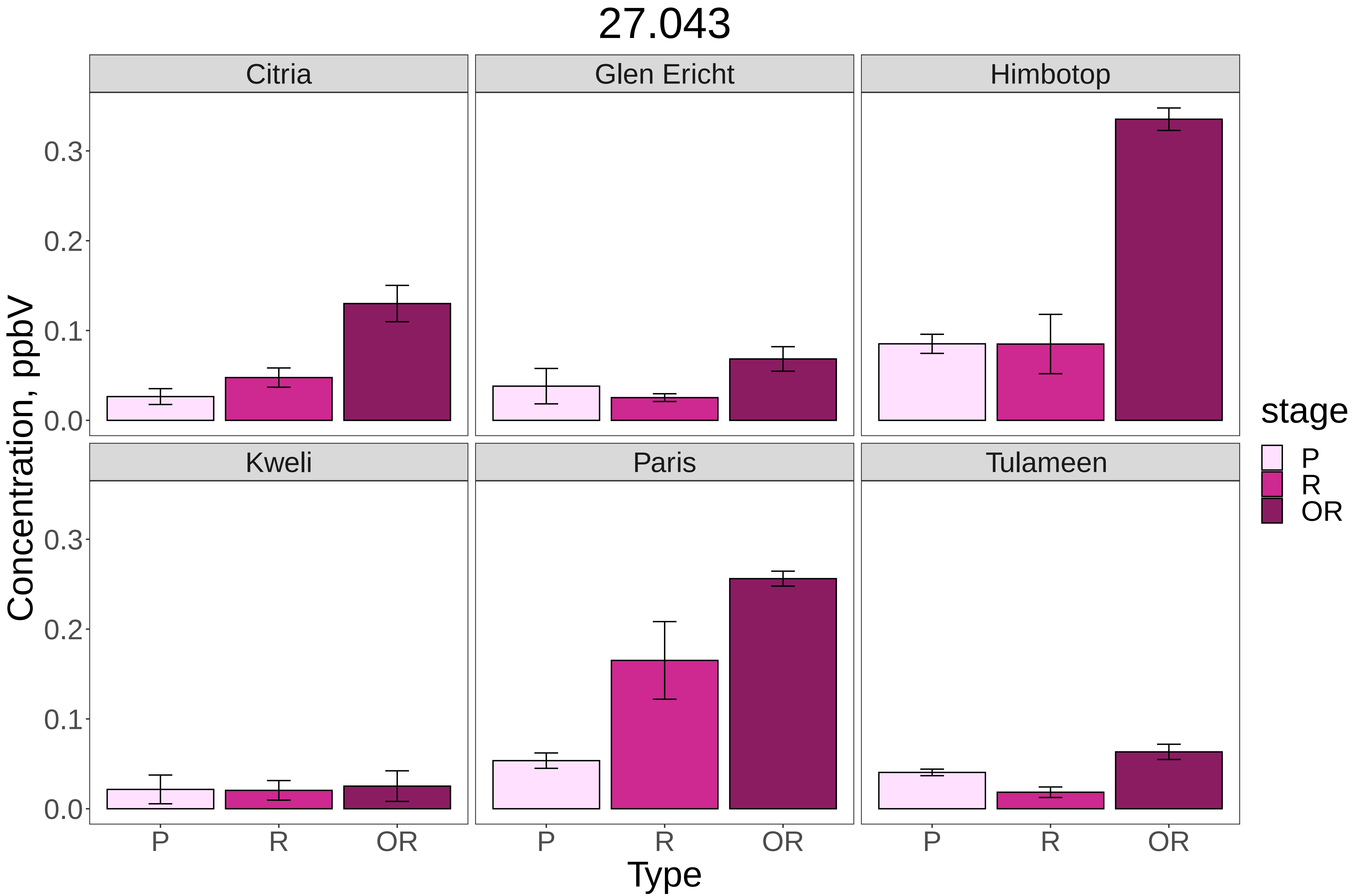
27.043
Citria
Glen Ericht
Himbotop
Kweli
Paris
Tulameen
Concentration, ppbV
0.0
0.1
0.2
0.3
P
R
OR
Type
stage
P
R
OR

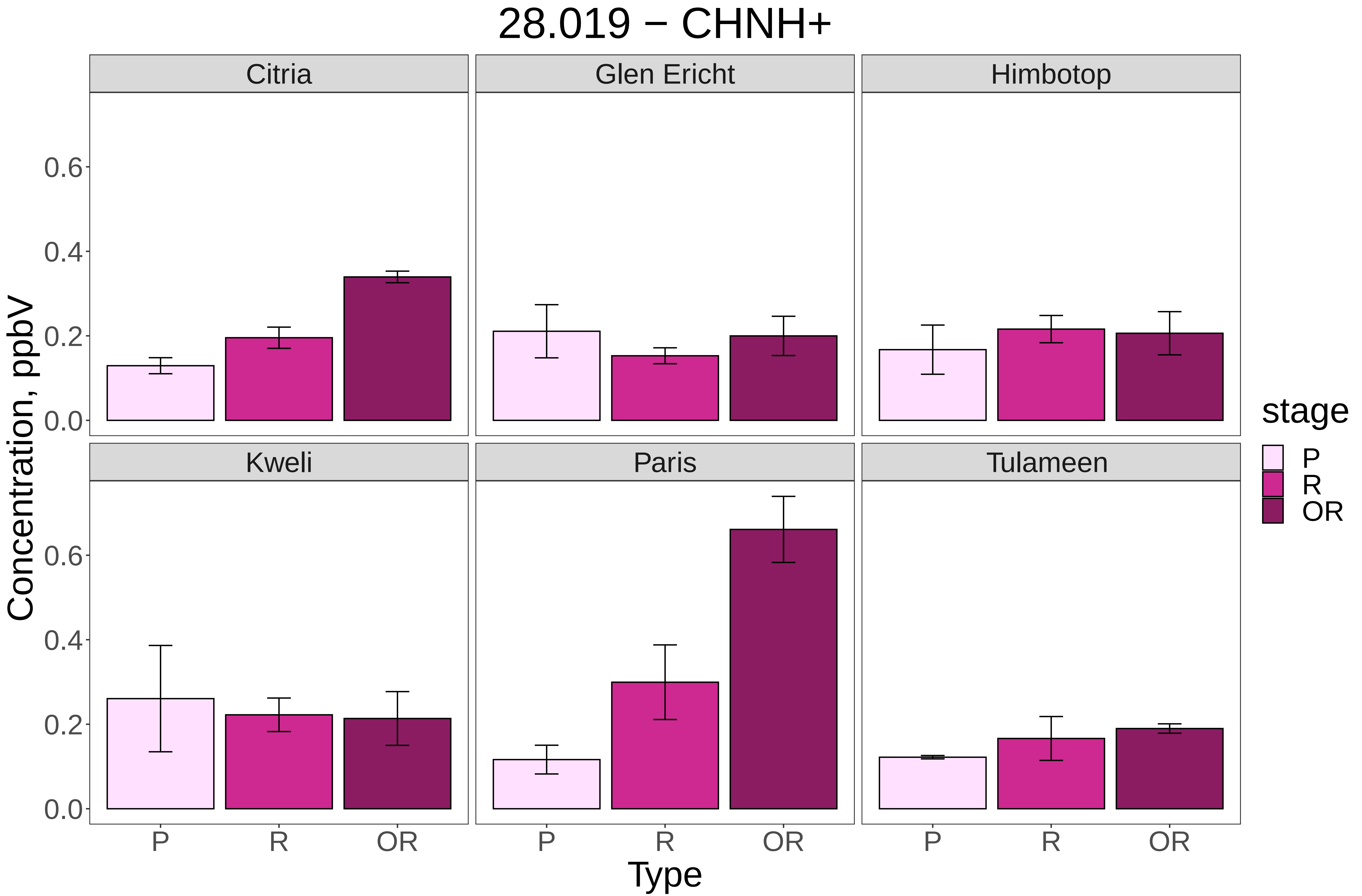
28.019 − CHNH+
Citria
Glen Ericht
Himbotop
Kweli
Paris
Tulameen
0.0
0.2
0.4
0.6
Concentration, ppbV
P
R
OR
Type
stage
P
R
OR

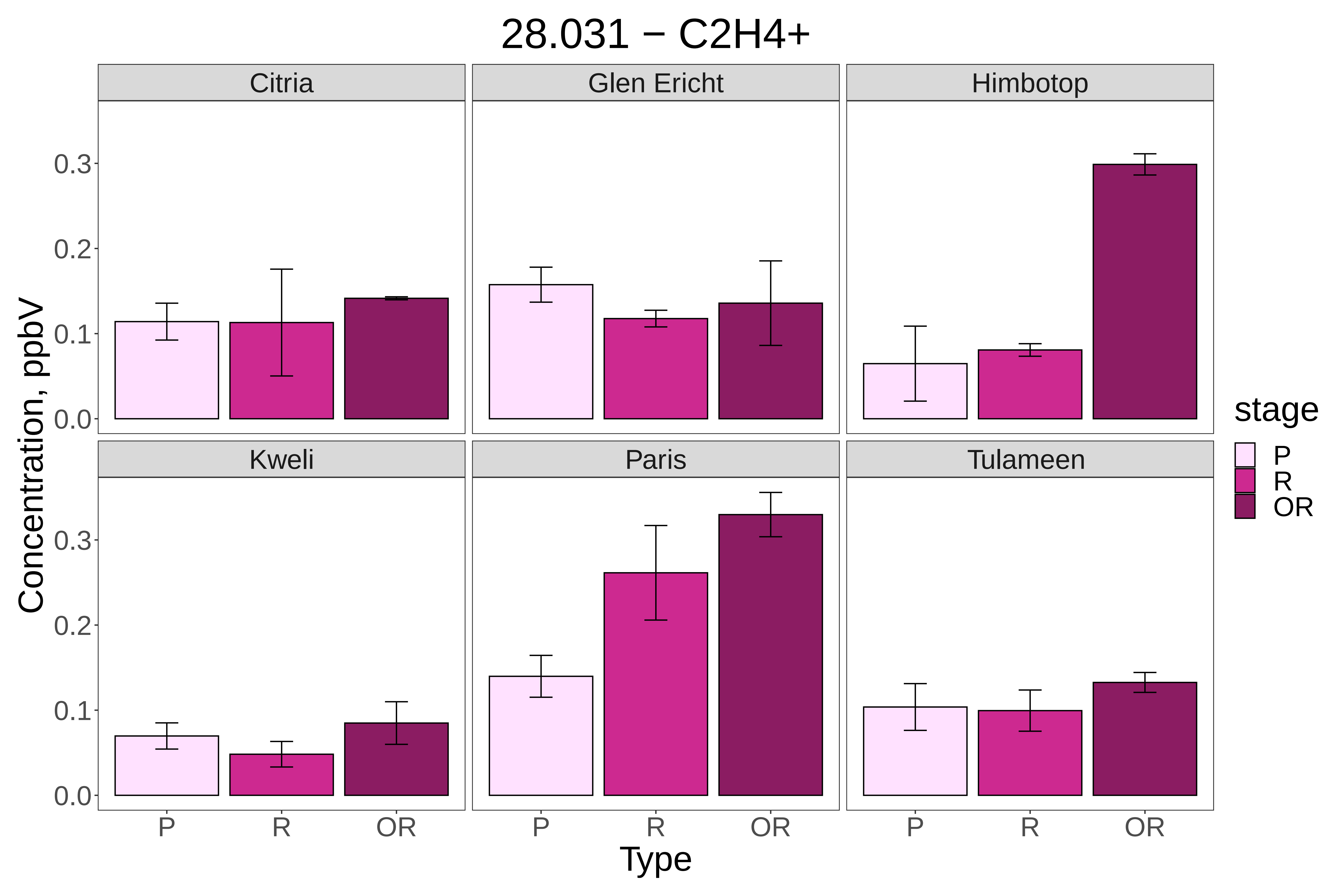

28.031 − C2H4+
Citria
Glen Ericht
Himbotop
Kweli
Paris
Tulameen
Concentration, ppbV
0.0
0.1
0.2
0.3
P
R
OR
Type
stage
P
R
OR

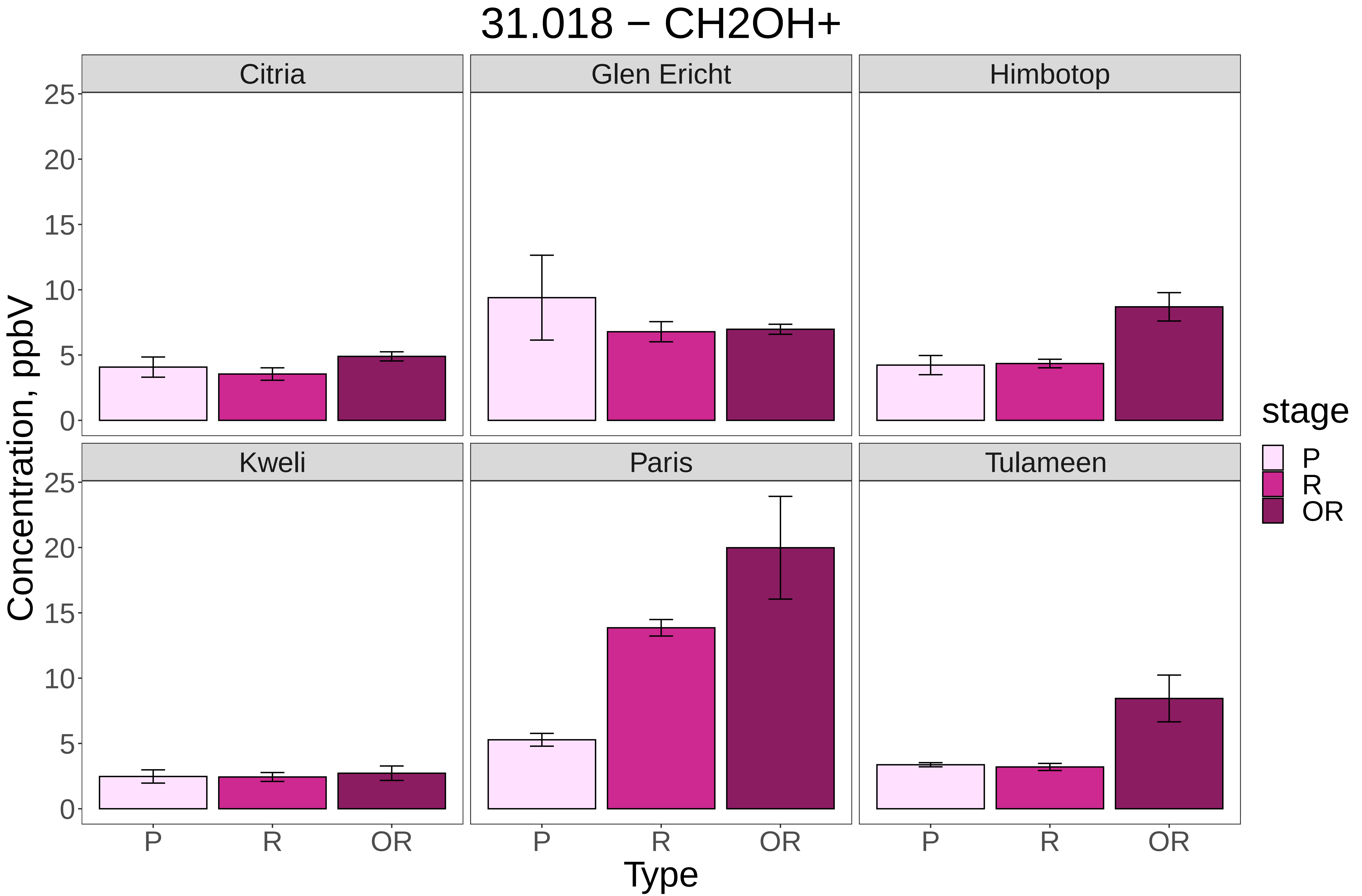

31.018 − CH2OH+
Citria
Glen Ericht
Himbotop
Kweli
Paris
Tulameen
Concentration, ppbV
0
5
10
15
20
25
P
R
OR
Type
stage
P
R
OR

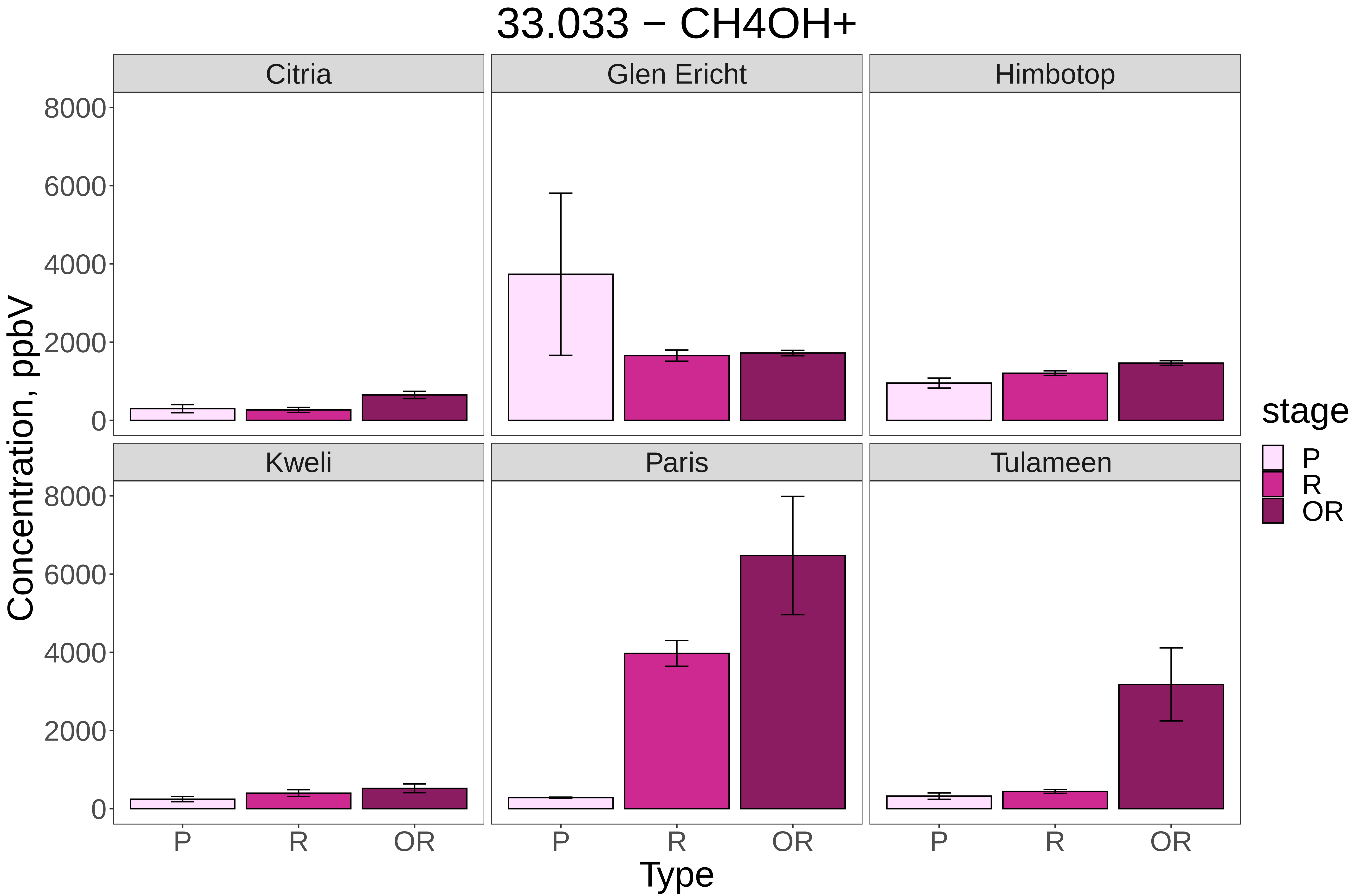
33.033 − CH4OH+
Citria
Glen Ericht
Himbotop
Kweli
Paris
Tulameen
Concentration, ppbV
8000
6000
4000
2000
0
P
R
OR
Type
stage
P
R
OR

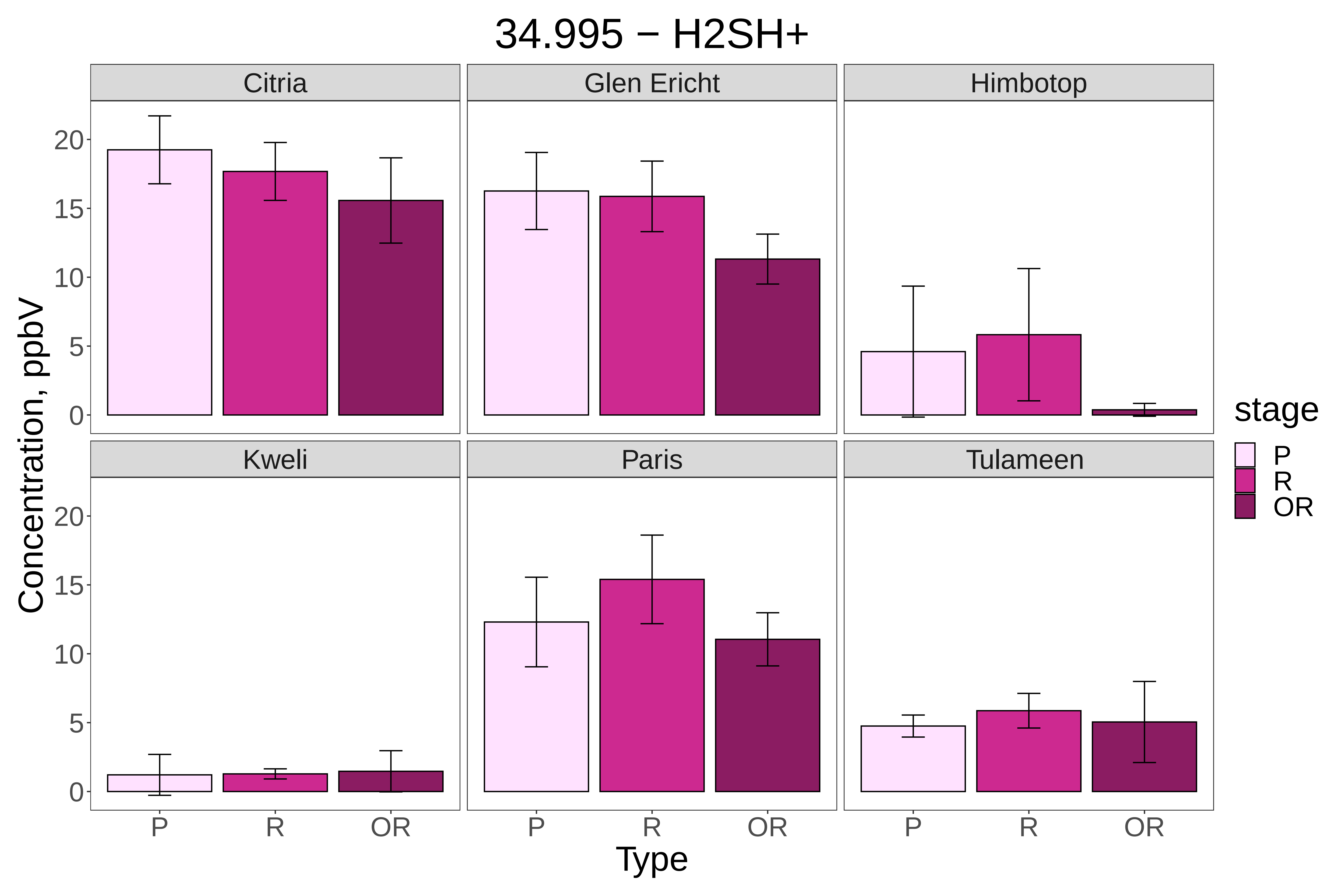
34.995 − H2SH+
Citria
Glen Ericht
Himbotop
Kweli
Paris
Tulameen
Concentration, ppbV
0
5
10
15
20
P
R
OR
Type
stage
P
R
OR

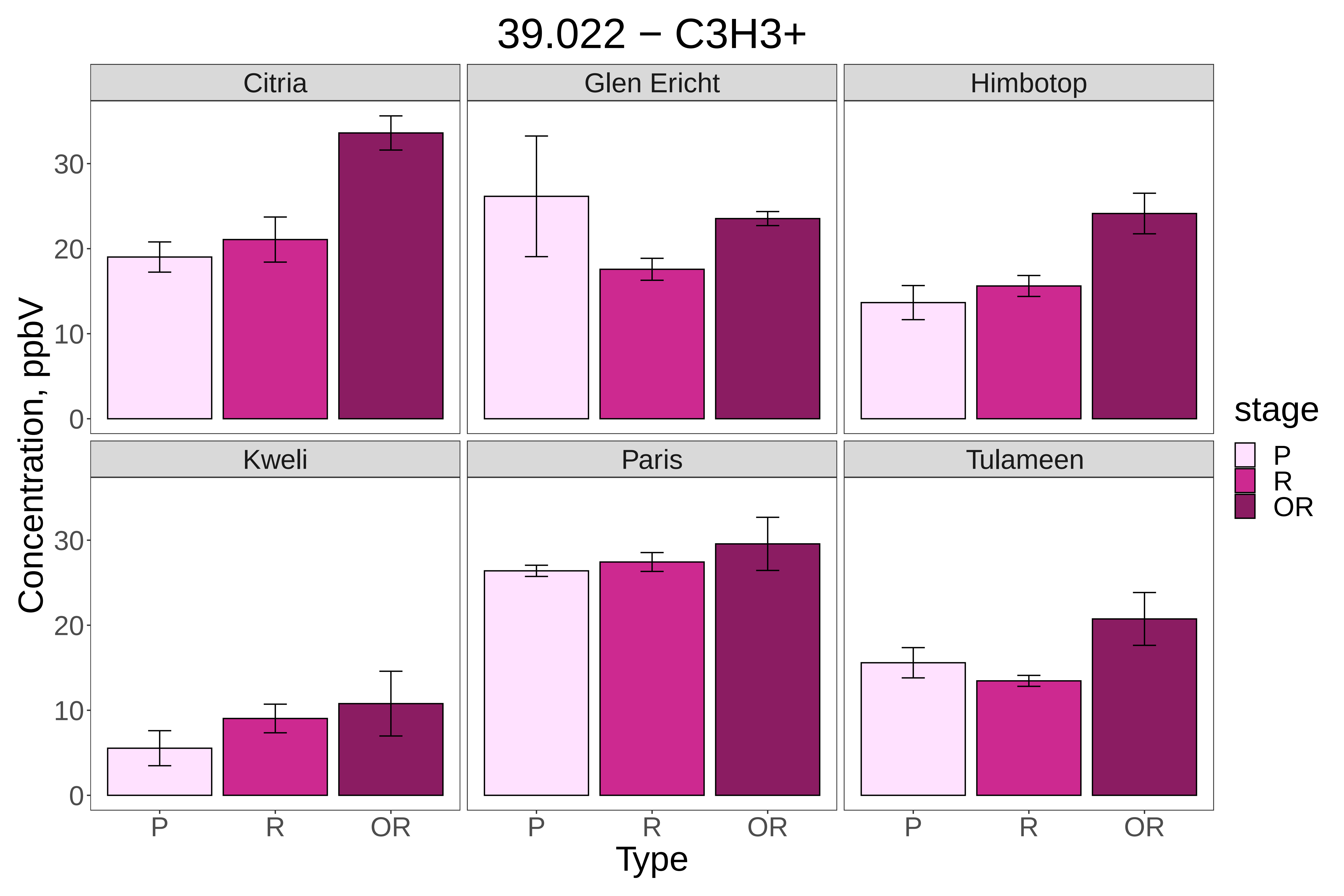

39.022 − C3H3+
Citria
Glen Ericht
Himbotop
Kweli
Paris
Tulameen
Concentration, ppbV
0
10
20
30
P
R
OR
Type
stage
P
R
OR

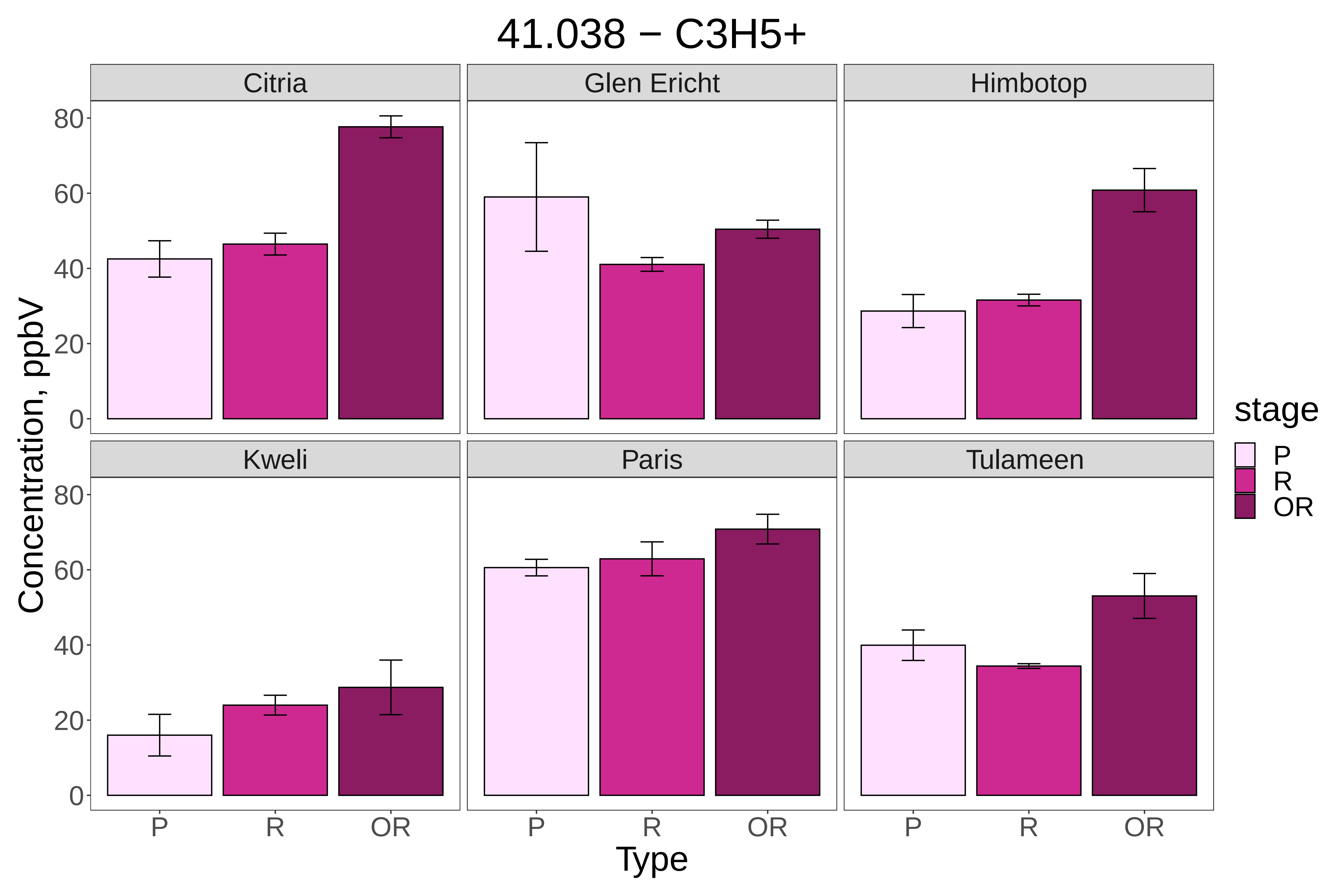

41.038 − C3H5+
Citria
Glen Ericht
Himbotop
Kweli
Paris
Tulameen
Concentration, ppbV
0
20
40
60
80
P
R
OR
Type
stage
P
R
OR

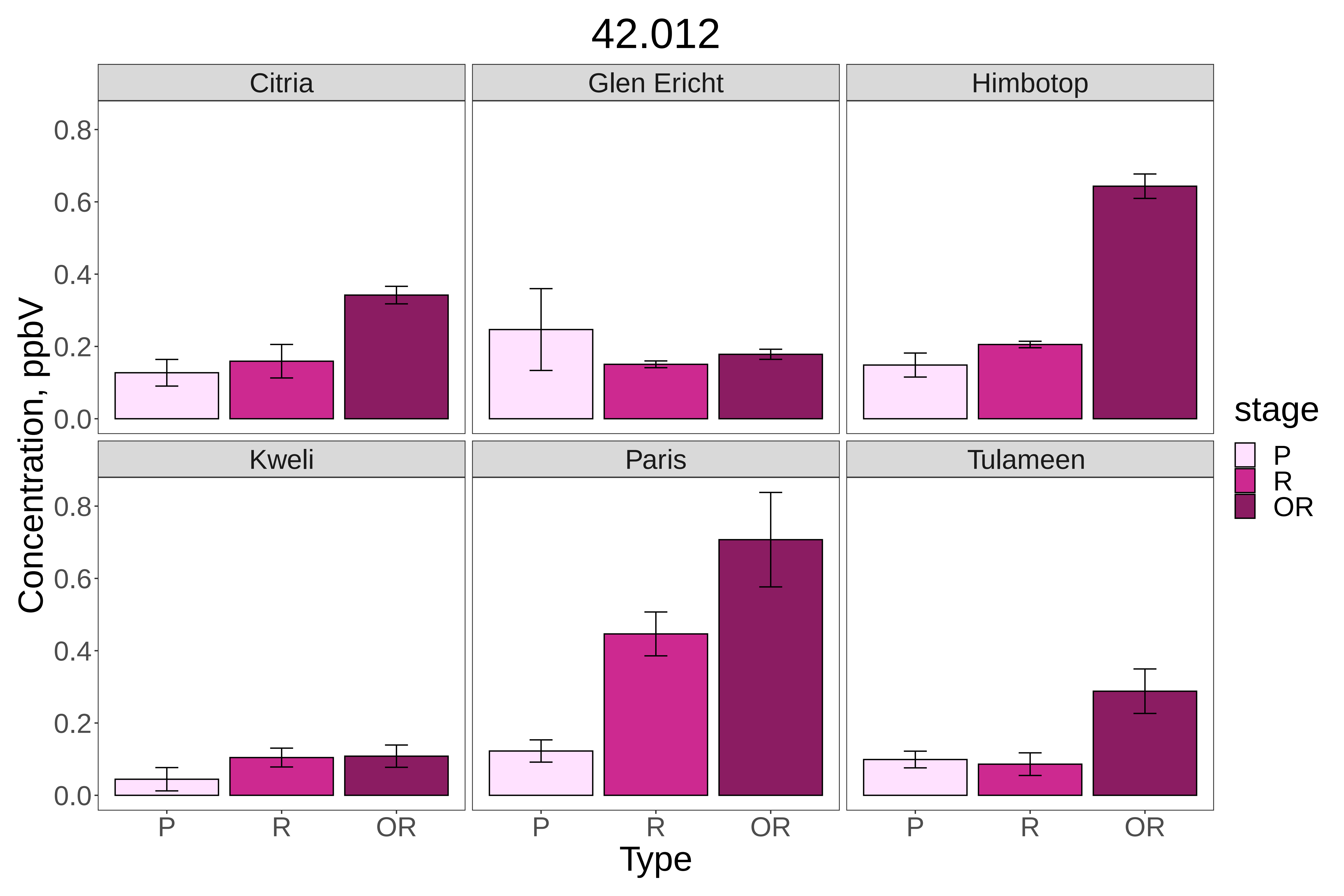

42.012
Citria
Glen Ericht
Himbotop
Kweli
Paris
Tulameen
0.8
0.6
0.4
0.2
0.0
Concentration, ppbV
P
R
OR
Type
stage
P
R
OR

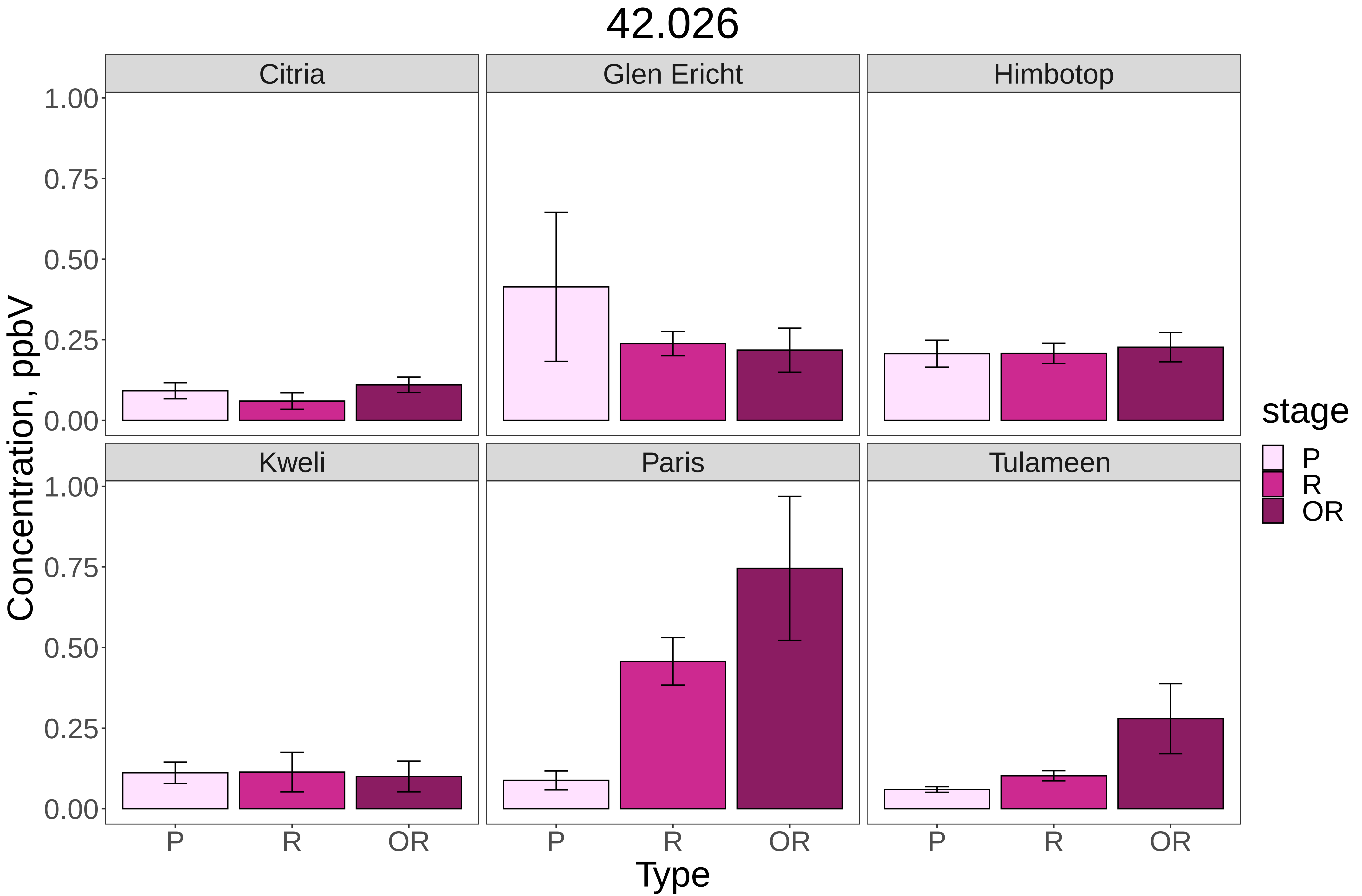
42.026
Citria
Glen Ericht
Himbotop
Kweli
Paris
Tulameen
Concentration, ppbV
0.00
0.25
0.50
0.75
1.00
Type
P
R
OR
stage
P
R
OR

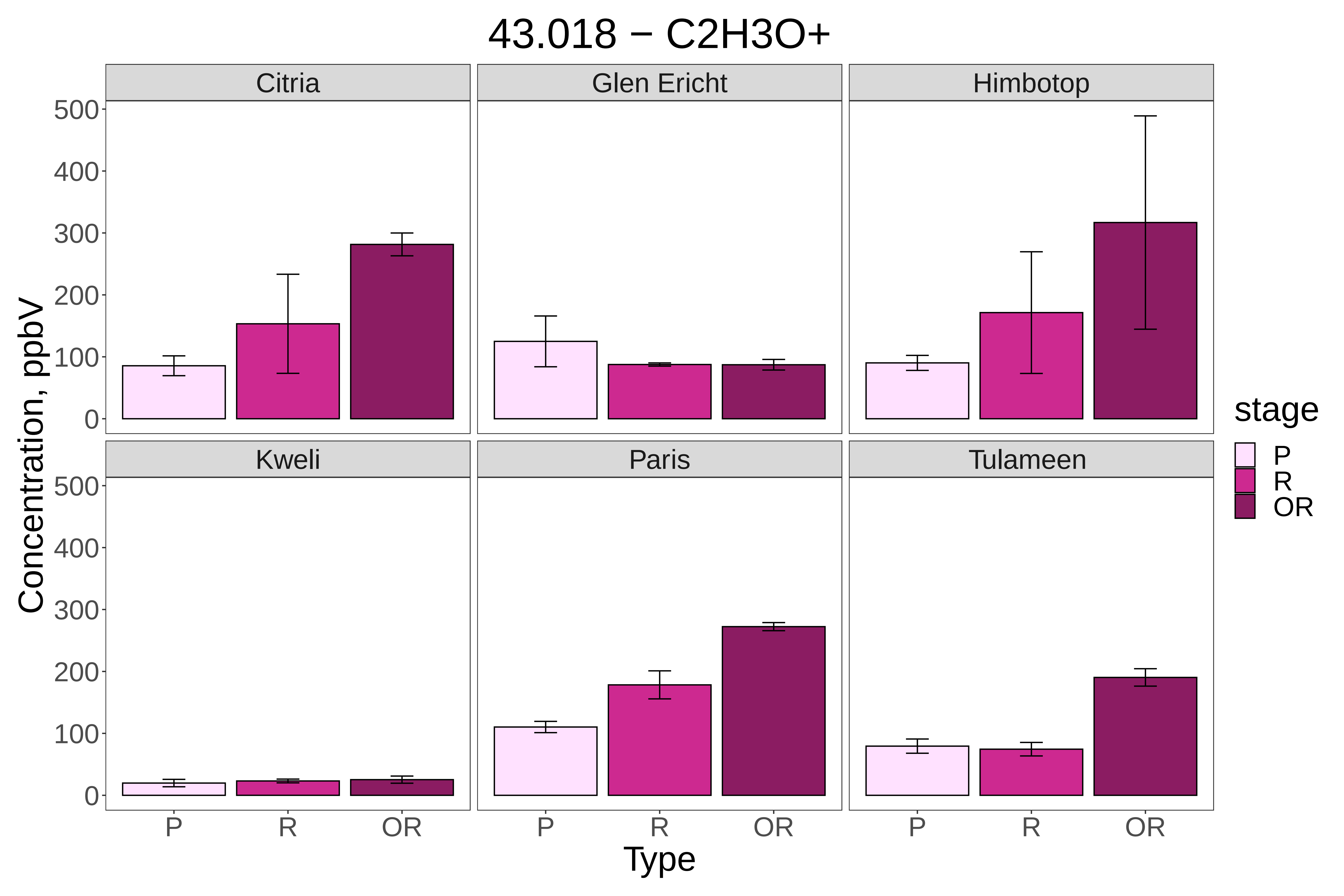

43.018 − C2H3O+
Citria
Glen Ericht
Himbotop
Kweli
Paris
Tulameen
Concentration, ppbV
0
100
200
300
400
500
P
R
OR
Type
stage
P
R
OR

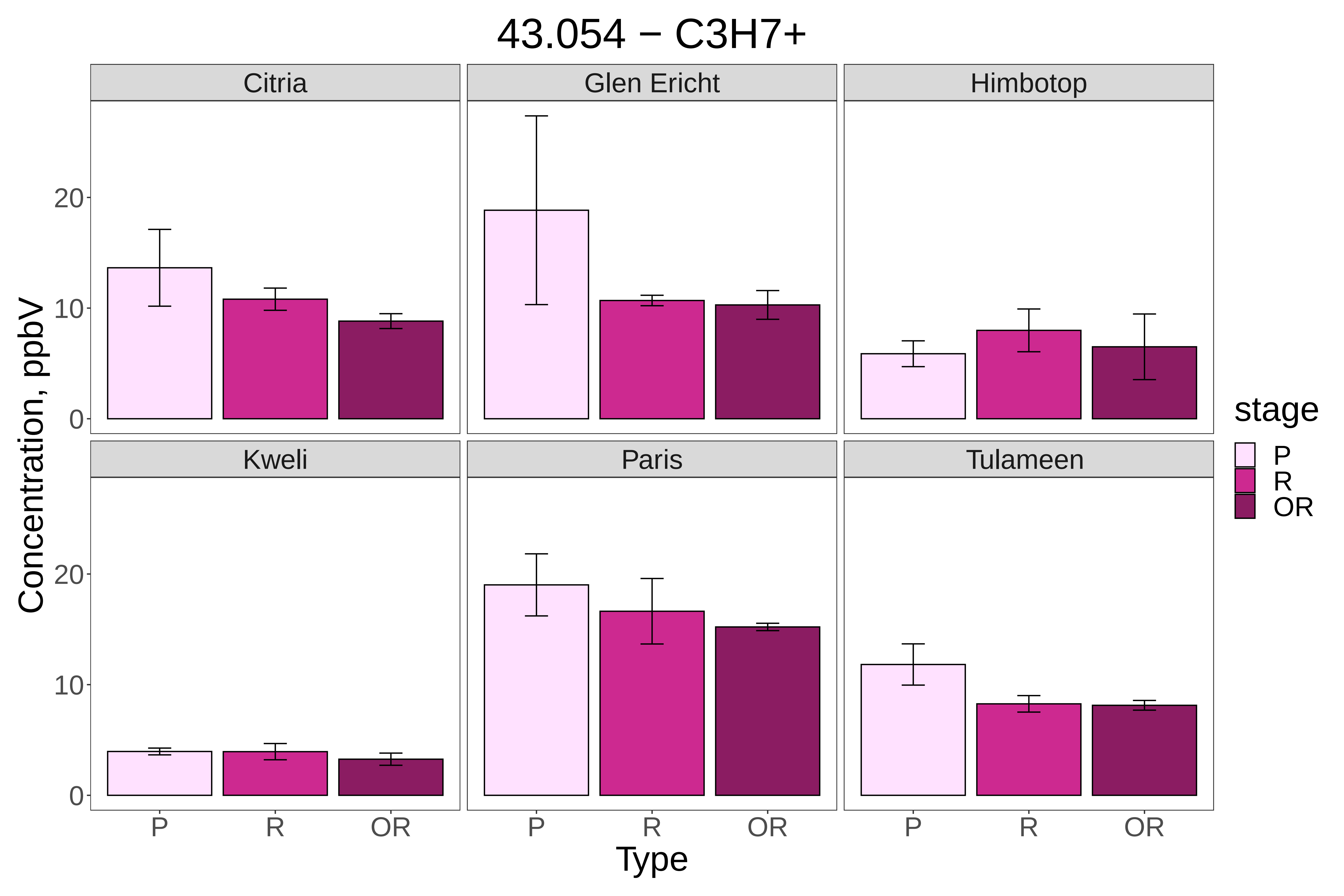
43.054 − C3H7+
Citria
Glen Ericht
Himbotop
Kweli
Paris
Tulameen
Concentration, ppbV
0
10
20
P
R
OR
Type
stage
P
R
OR

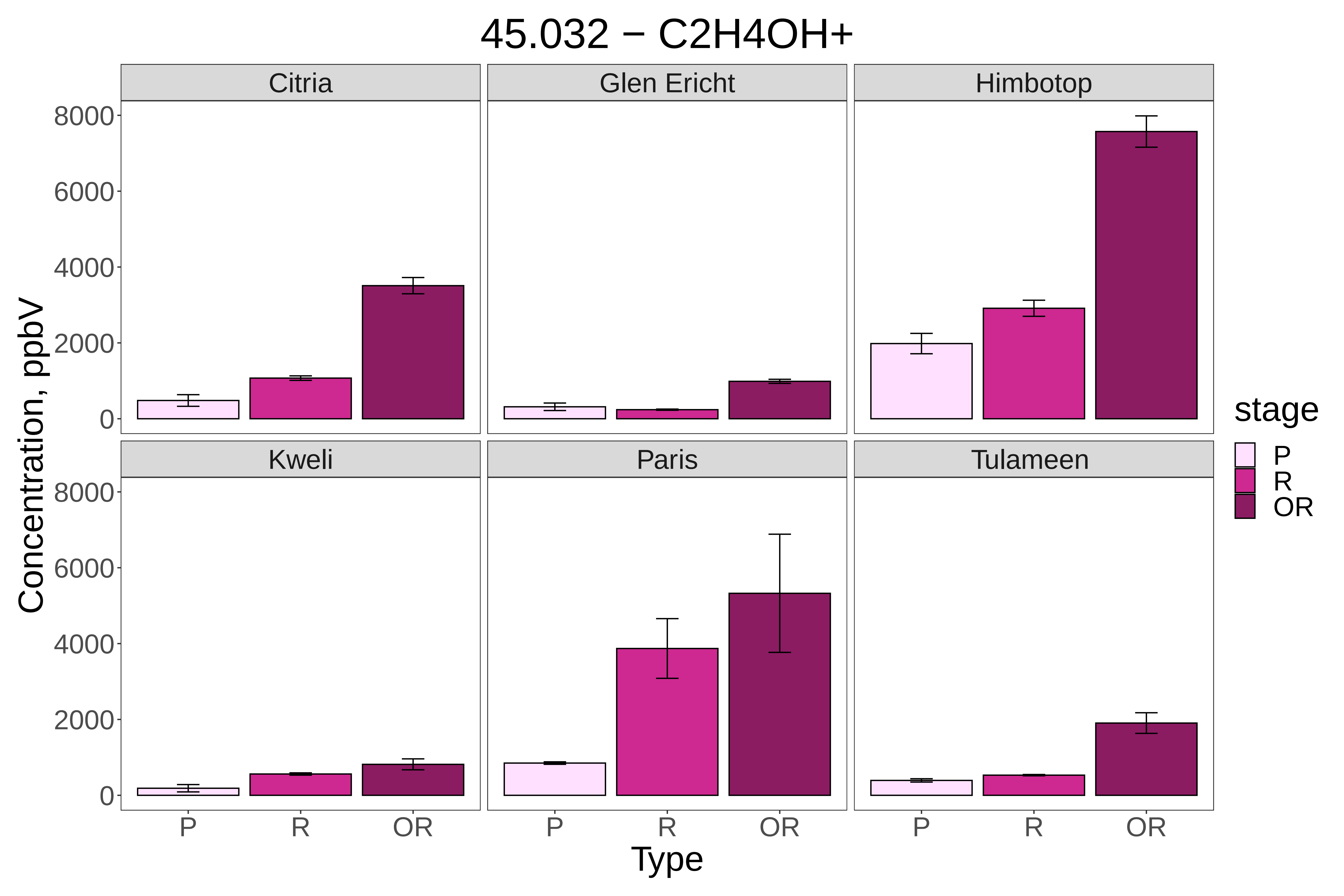
45.032 − C2H4OH+
Citria
Glen Ericht
Himbotop
Kweli
Paris
Tulameen
Concentration, ppbV
0
2000
4000
6000
8000
P
R
OR
Type
stage
P
R
OR

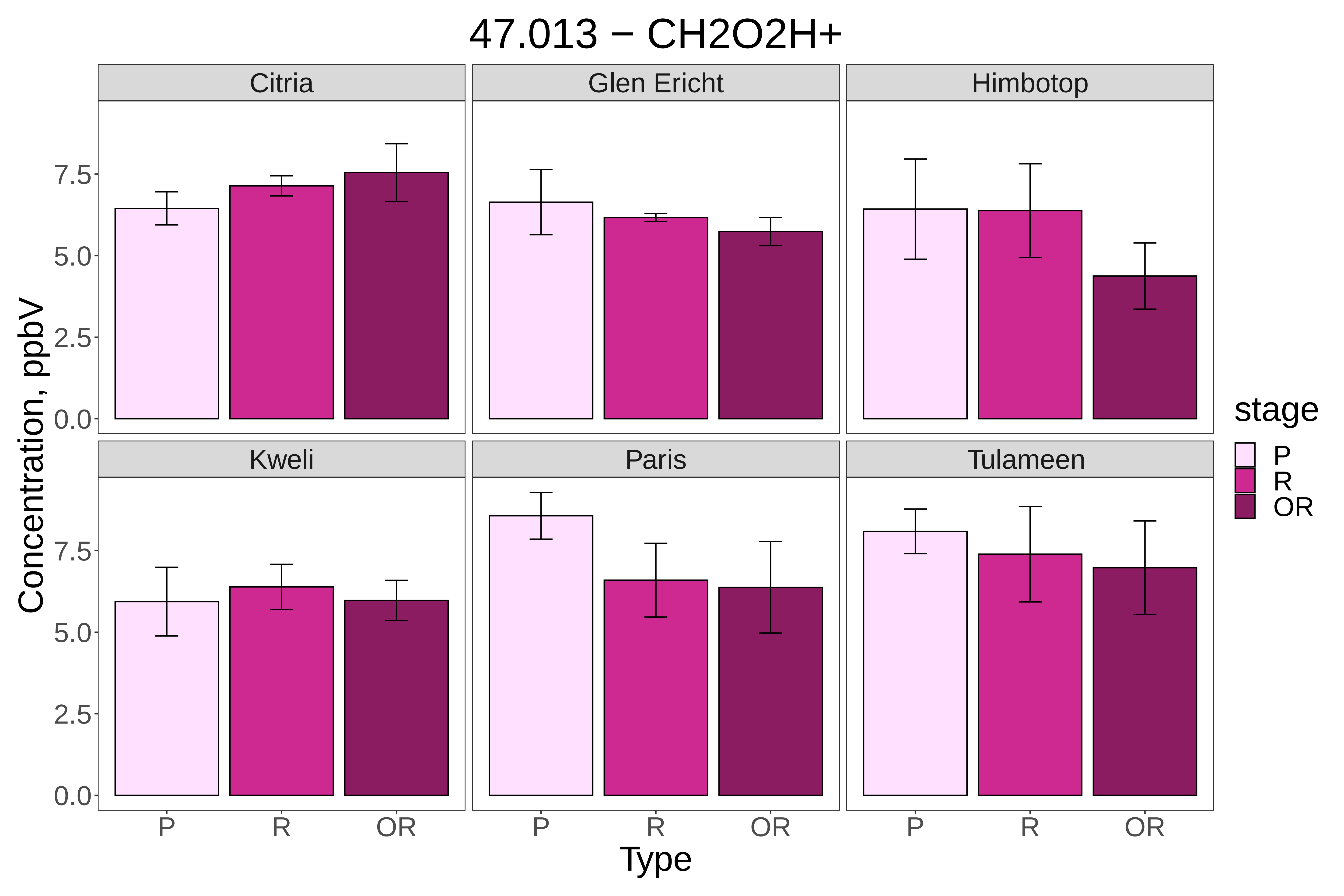

47.013 − CH2O2H+
Citria
Glen Ericht
Himbotop
Kweli
Paris
Tulameen
Concentration, ppbV
0.0
2.5
5.0
7.5
Type
P
R
OR
stage
P
R
OR

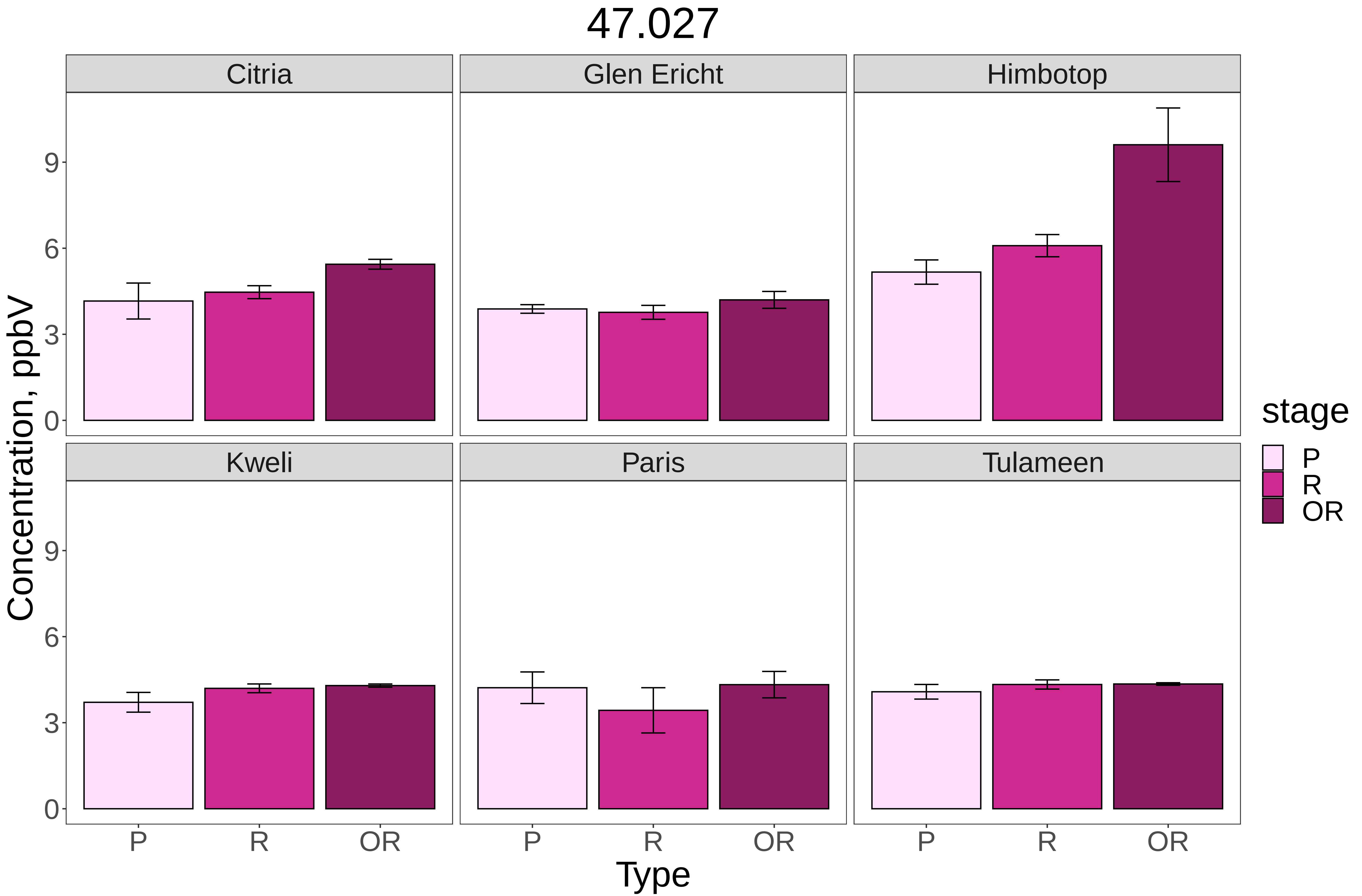
47.027
Citria
Glen Ericht
Himbotop
Kweli
Paris
Tulameen
Concentration, ppbV
0
3
6
9
P
R
OR
Type
stage
P
R
OR

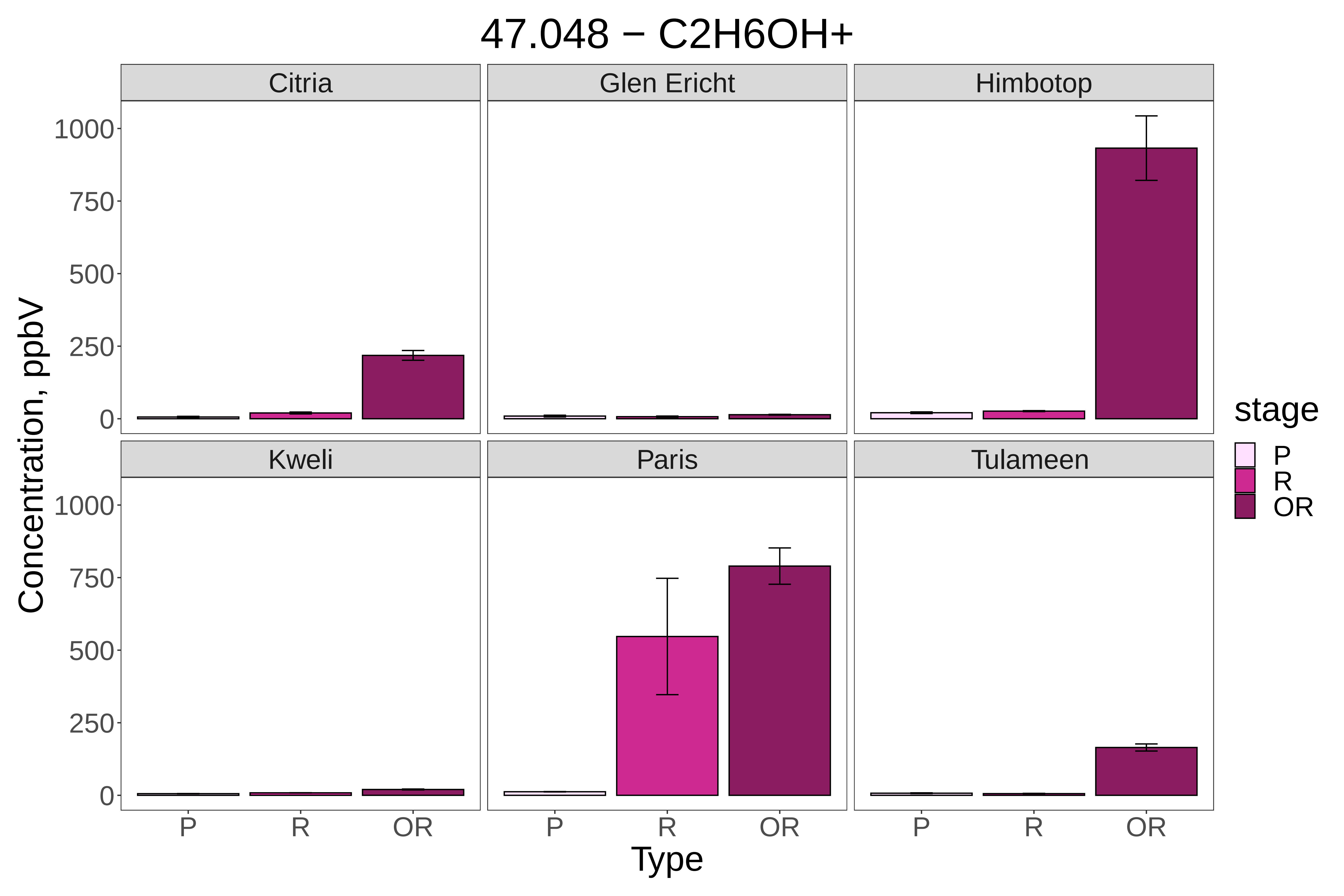

47.048 − C2H6OH+
Citria
Glen Ericht
Himbotop
Kweli
Paris
Tulameen
Concentration, ppbV
0
250
500
750
1000
P
R
OR
Type
stage
P
R
OR

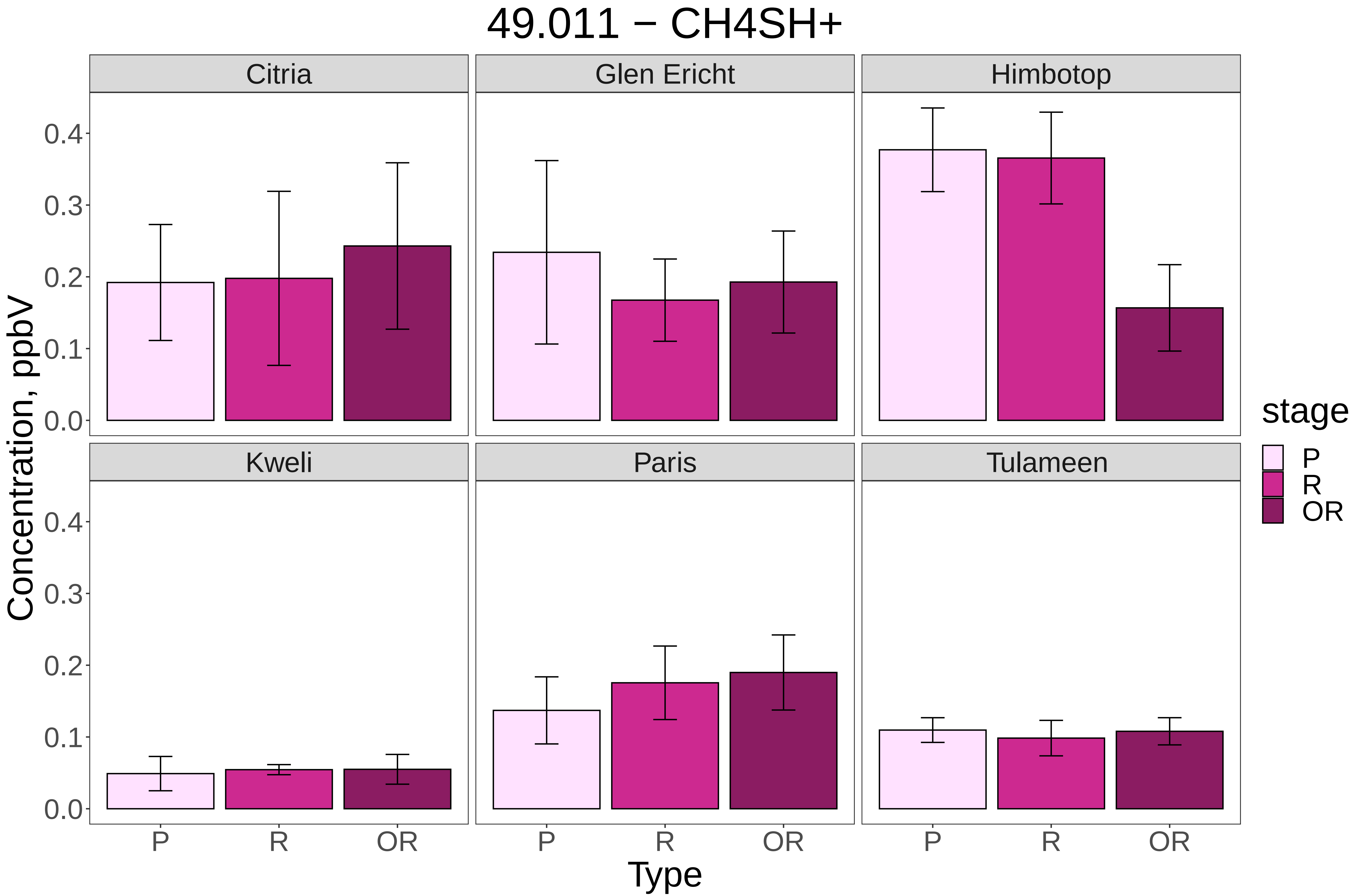

49.011 − CH4SH+
Citria
Glen Ericht
Himbotop
Kweli
Paris
Tulameen
Concentration, ppbV
0.0
0.1
0.2
0.3
0.4
P
R
OR
Type
stage
P
R
OR

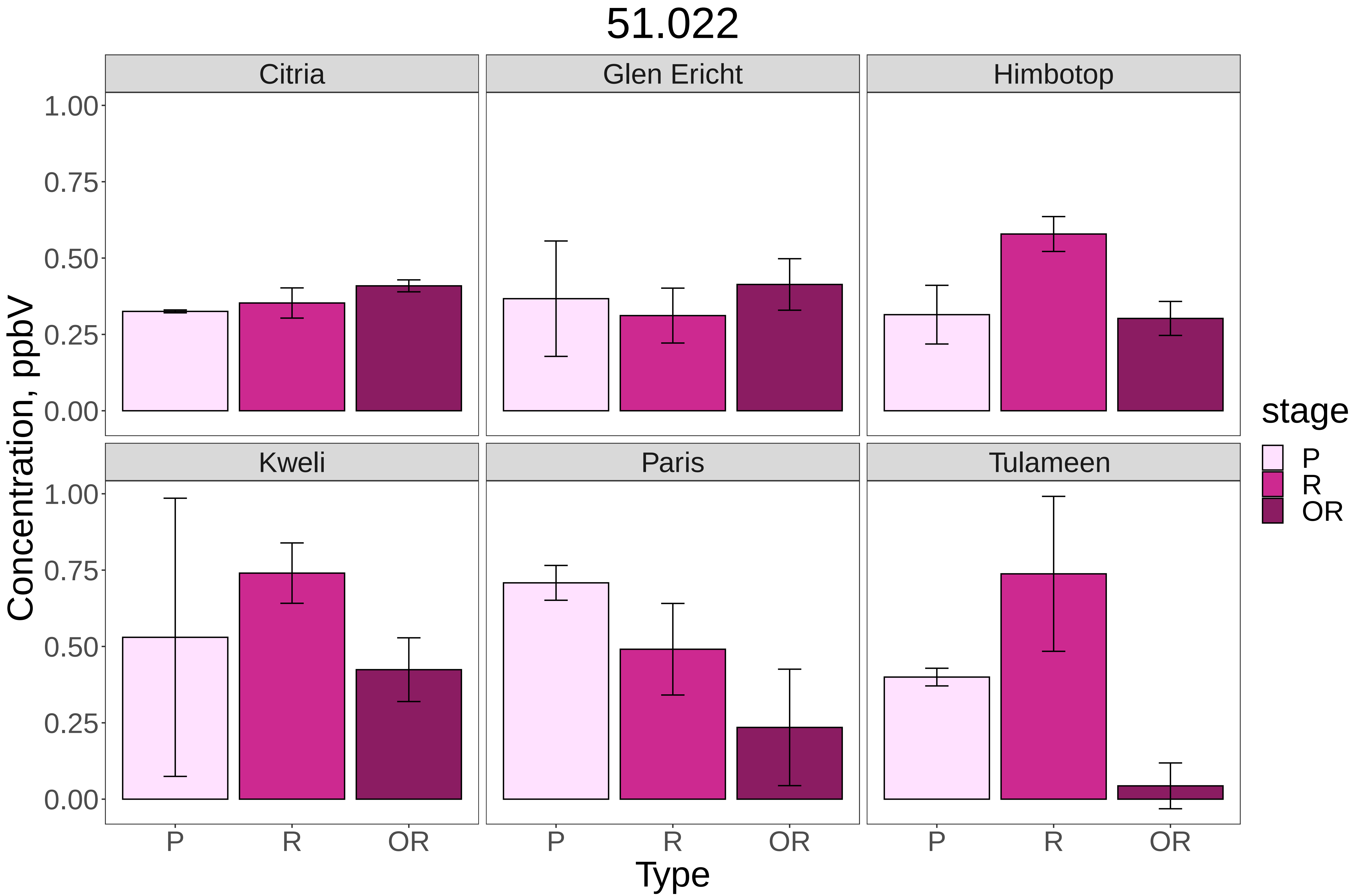

51.022
Citria
Glen Ericht
Himbotop
Kweli
Paris
Tulameen
1.00
0.75
0.50
0.25
0.00
Concentration, ppbV
P
R
OR
Type
stage
P
R
OR

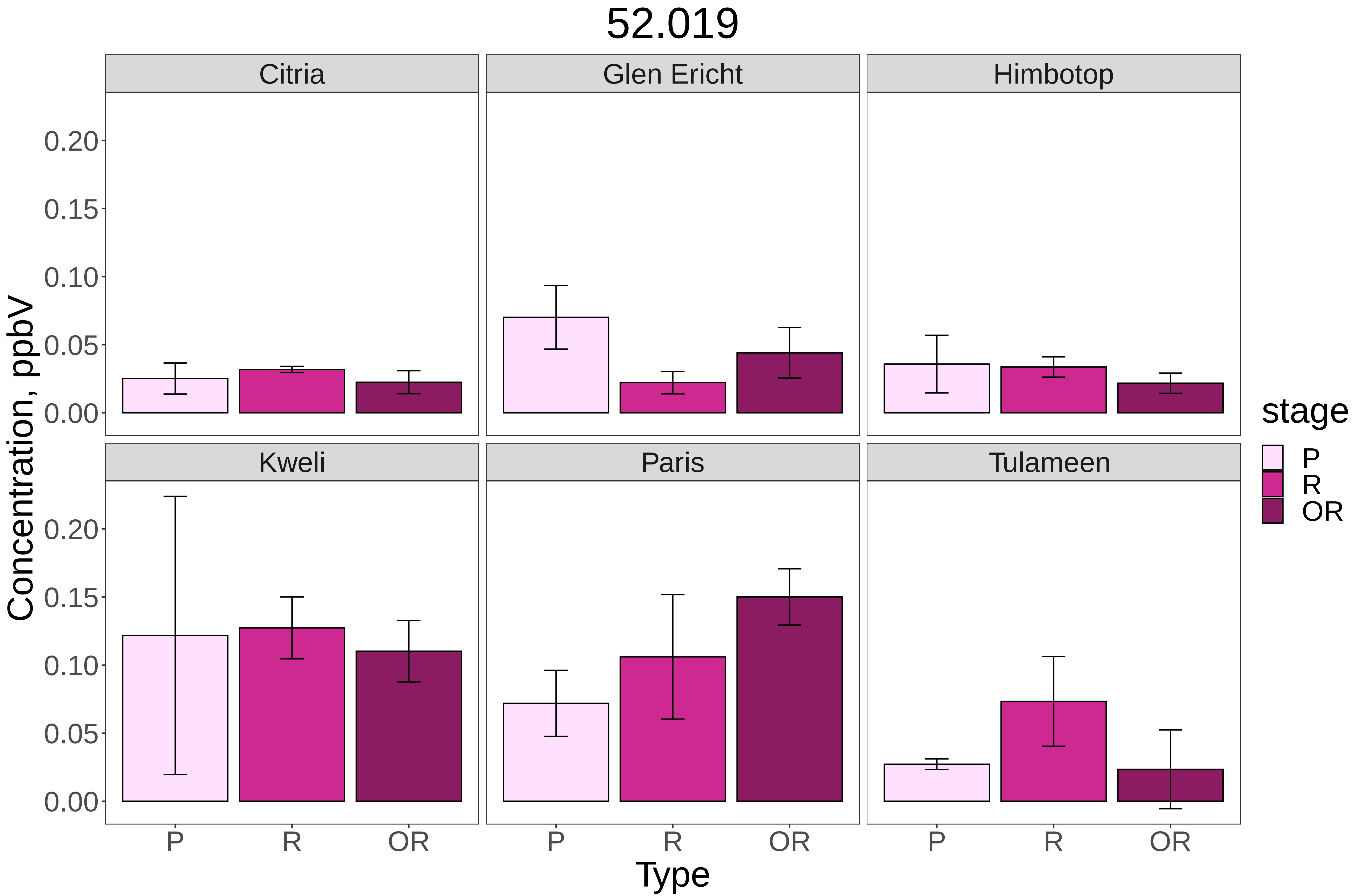

52.019
Citria
Glen Ericht
Himbotop
Kweli
Paris
Tulameen
Concentration, ppbV
0.00
0.05
0.10
0.15
0.20
P
R
OR
Type
stage
P
R
OR

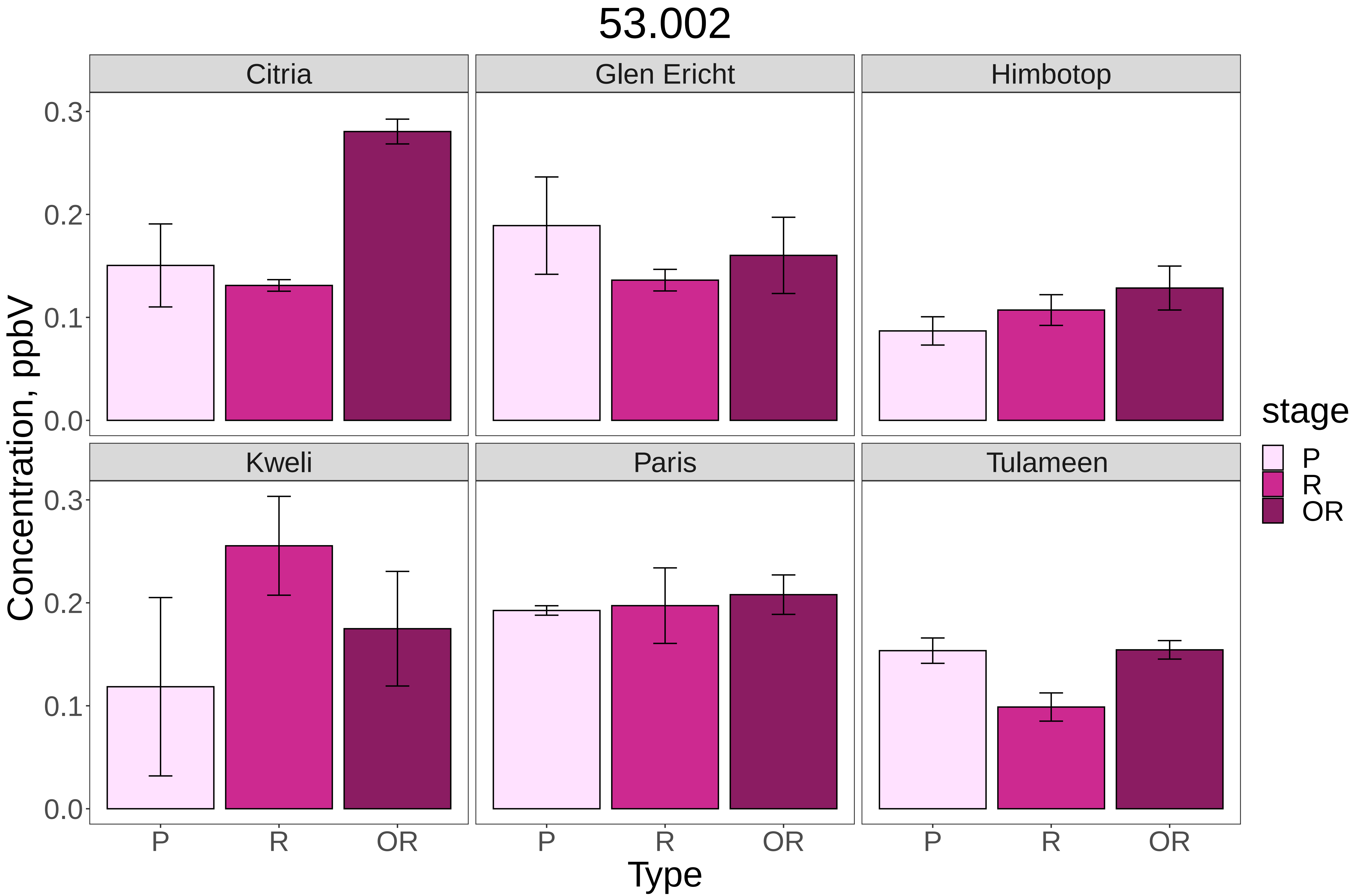
53.002
Citria
Glen Ericht
Himbotop
Kweli
Paris
Tulameen
Concentration, ppbV
0.0
0.1
0.2
0.3
Type
P
R
OR
stage
P
R
OR

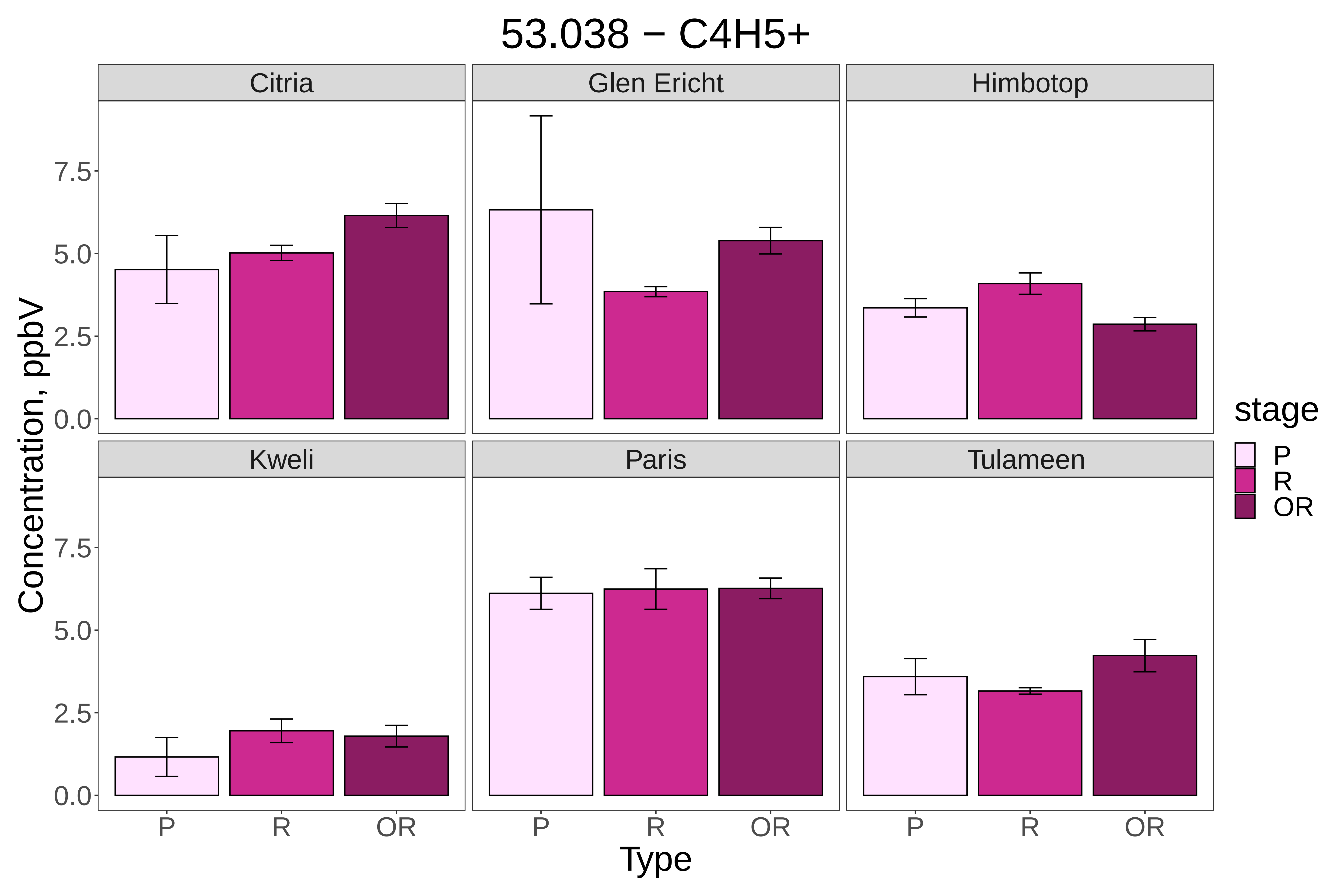

53.038 − C4H5+
Citria
Glen Ericht
Himbotop
Kweli
Paris
Tulameen
Concentration, ppbV
0.0
2.5
5.0
7.5
P
R
OR
Type
stage
P
R
OR

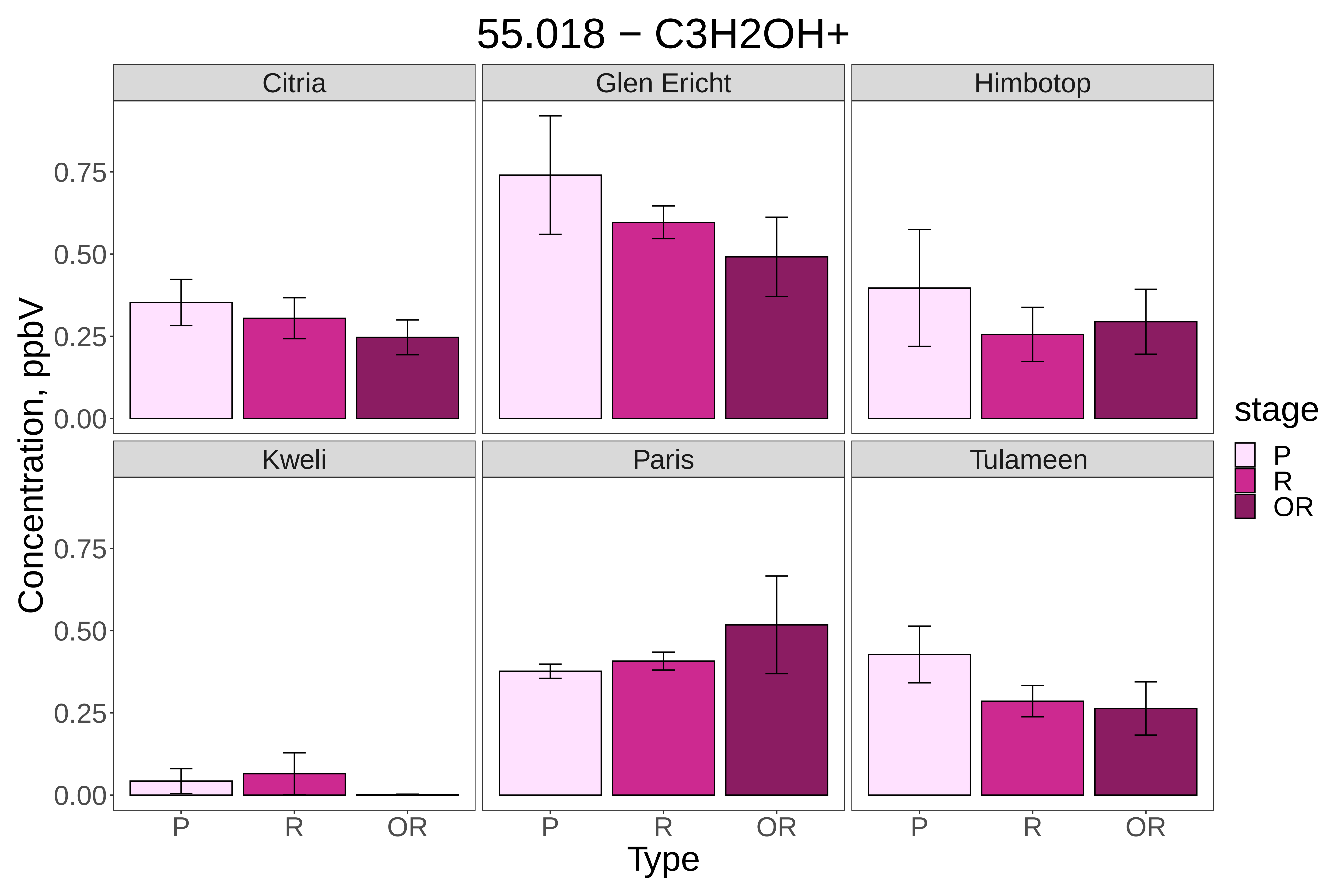

55.018 − C3H2OH+
Citria
Glen Ericht
Himbotop
Kweli
Paris
Tulameen
0.00
0.25
0.50
0.75
Concentration, ppbV
P
R
OR
Type
stage
P
R
OR

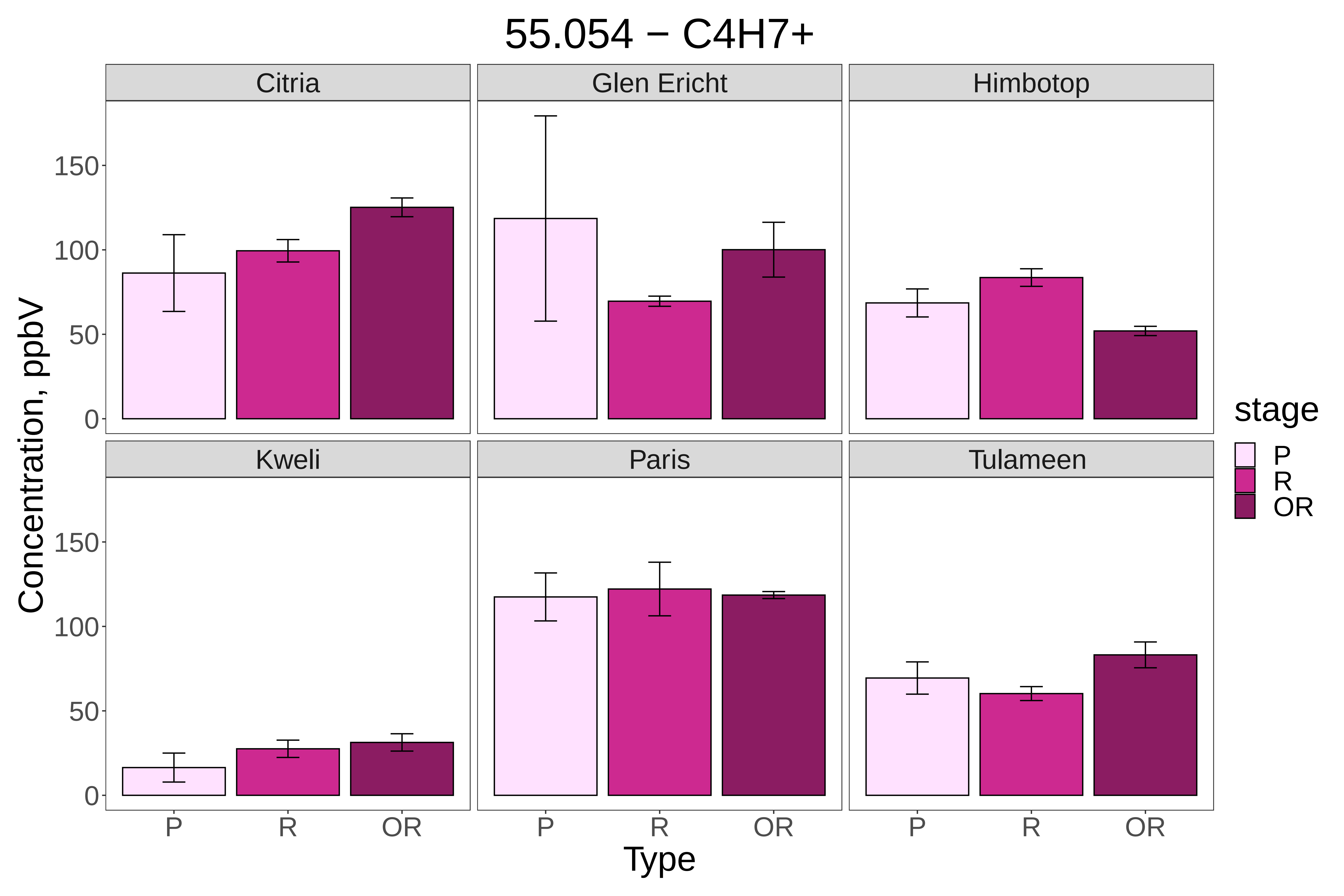
55.054 − C4H7+
Citria
Glen Ericht
Himbotop
Kweli
Paris
Tulameen
Concentration, ppbV
0
50
100
150
P
R
OR
Type
stage
P
R
OR

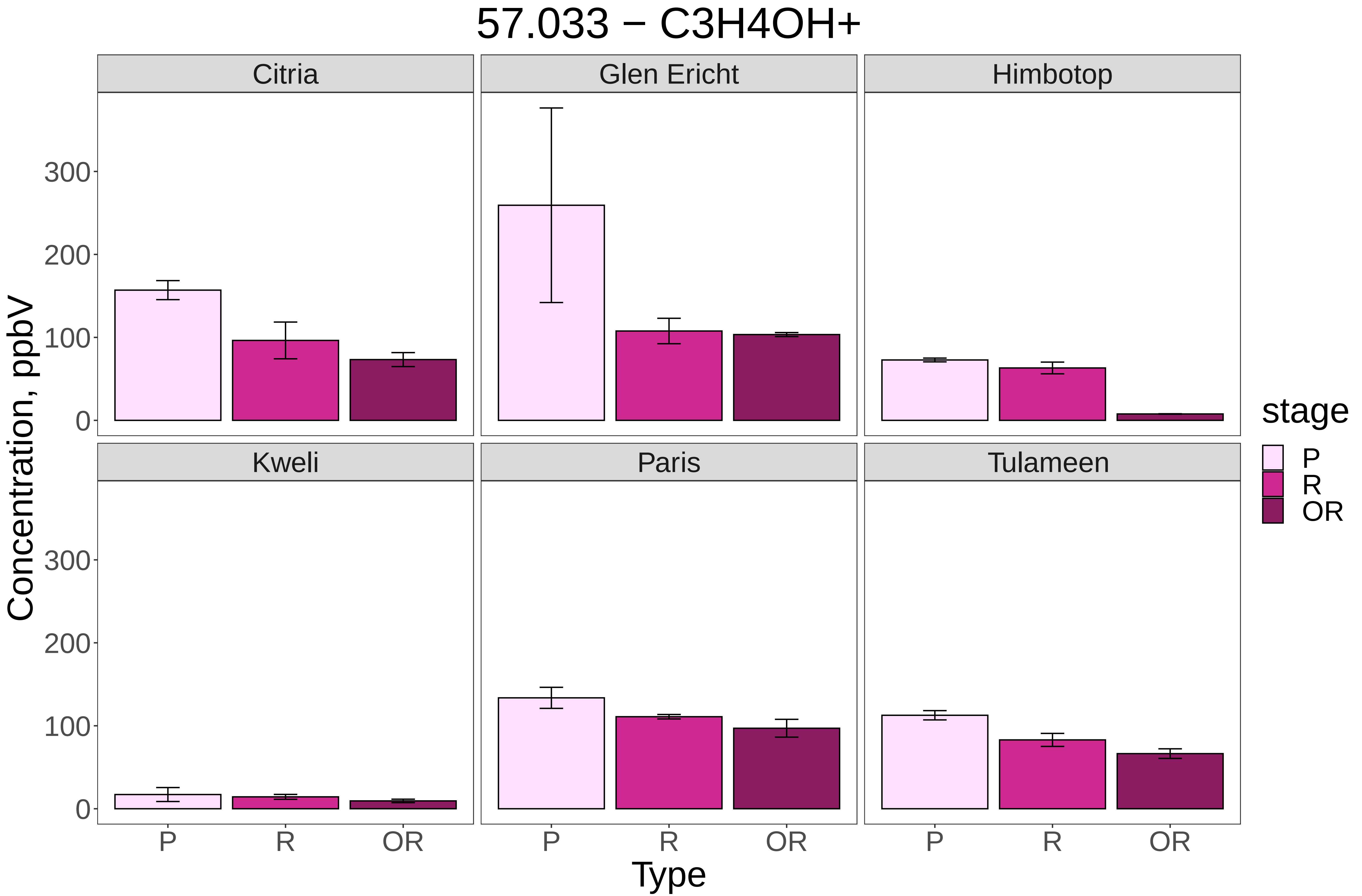

57.033 − C3H4OH+
Citria
Glen Ericht
Himbotop
Kweli
Paris
Tulameen
Concentration, ppbV
0
100
200
300
P
R
OR
Type
stage
P
R
OR

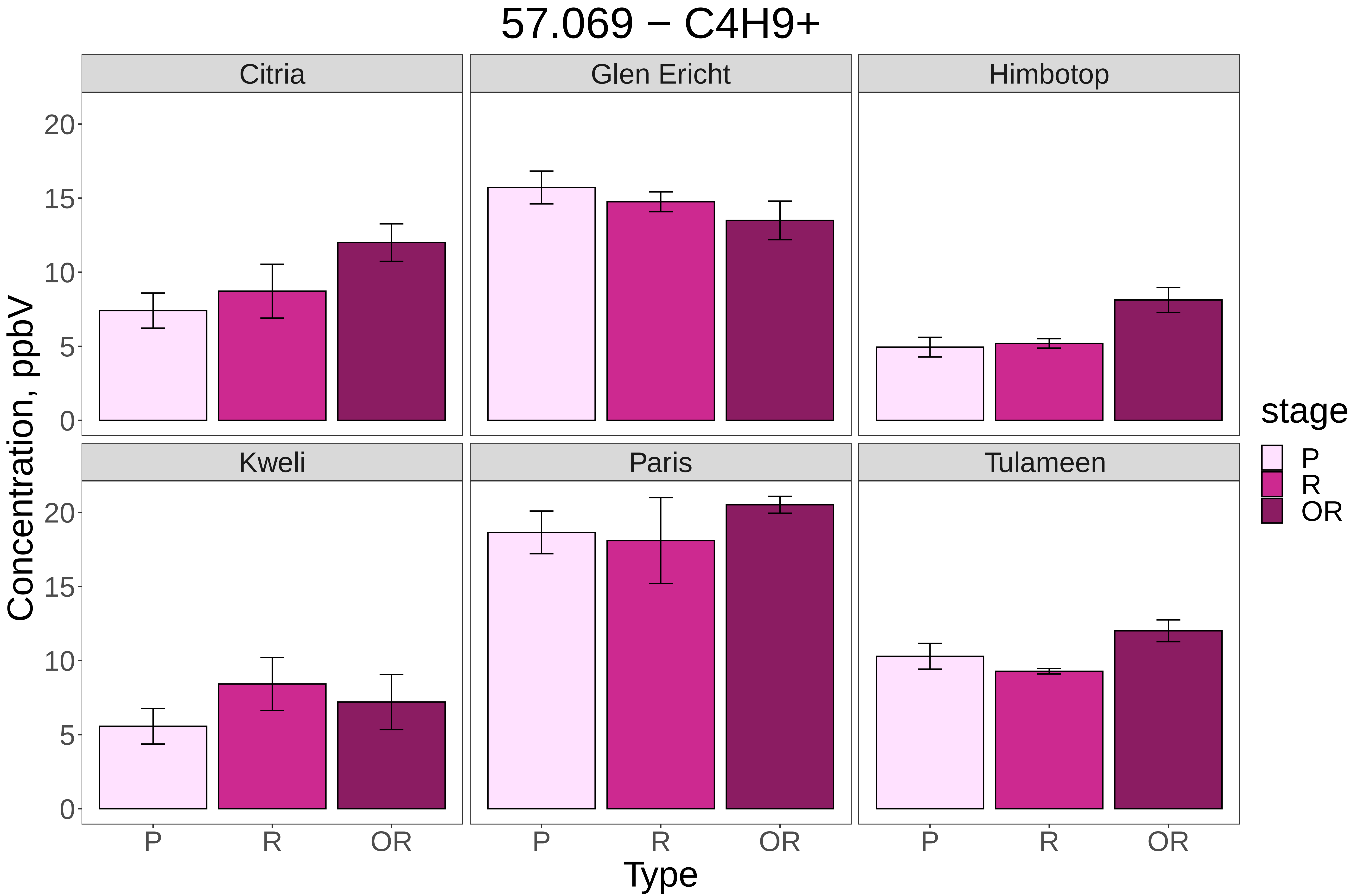

57.069 − C4H9+
Citria
Glen Ericht
Himbotop
Kweli
Paris
Tulameen
Concentration, ppbV
0
5
10
15
20
P
R
OR
Type
stage
P
R
OR

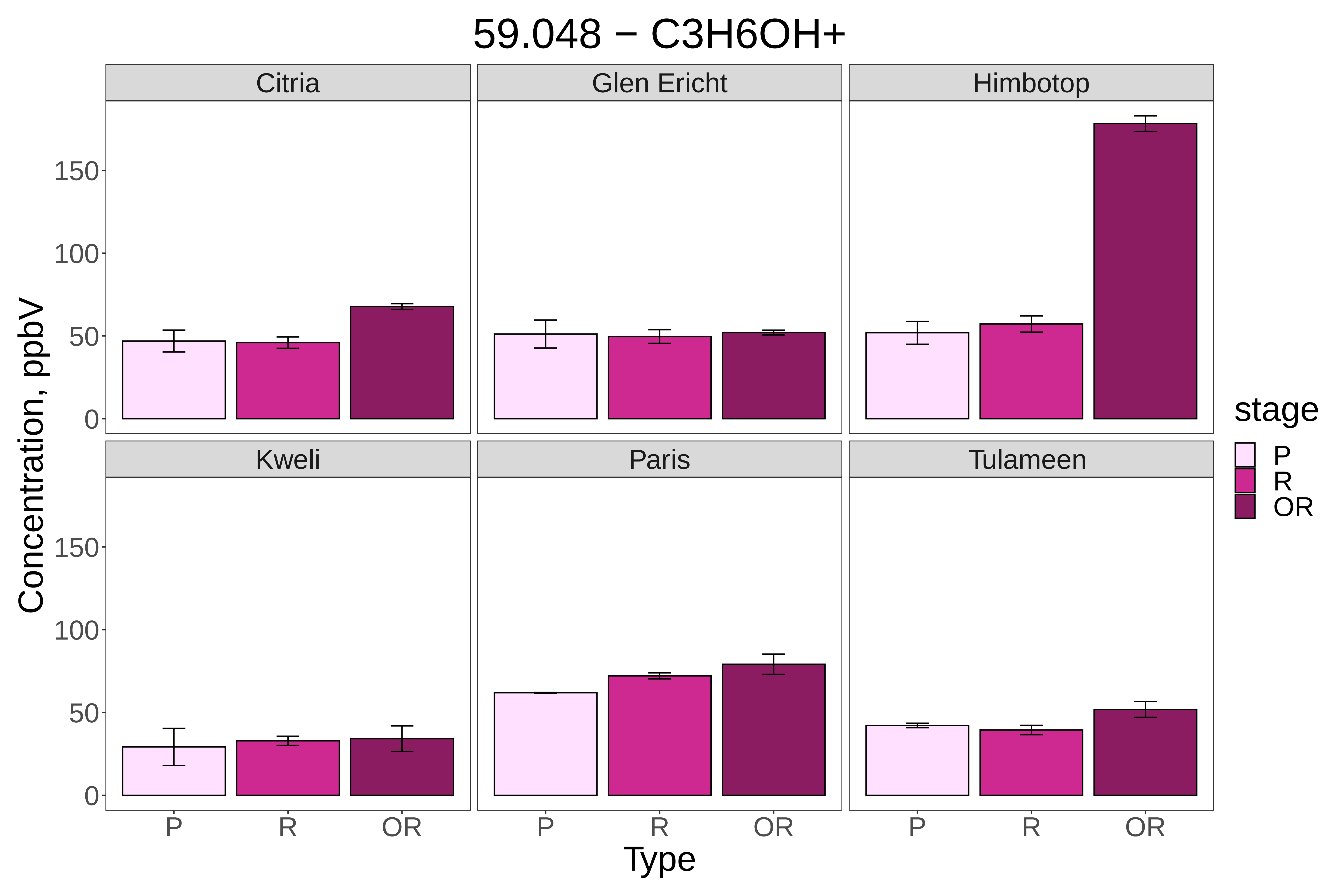

59.048 − C3H6OH+
Citria
Glen Ericht
Himbotop
Kweli
Paris
Tulameen
Concentration, ppbV
0
50
100
150
P
R
OR
Type
stage
P
R
OR

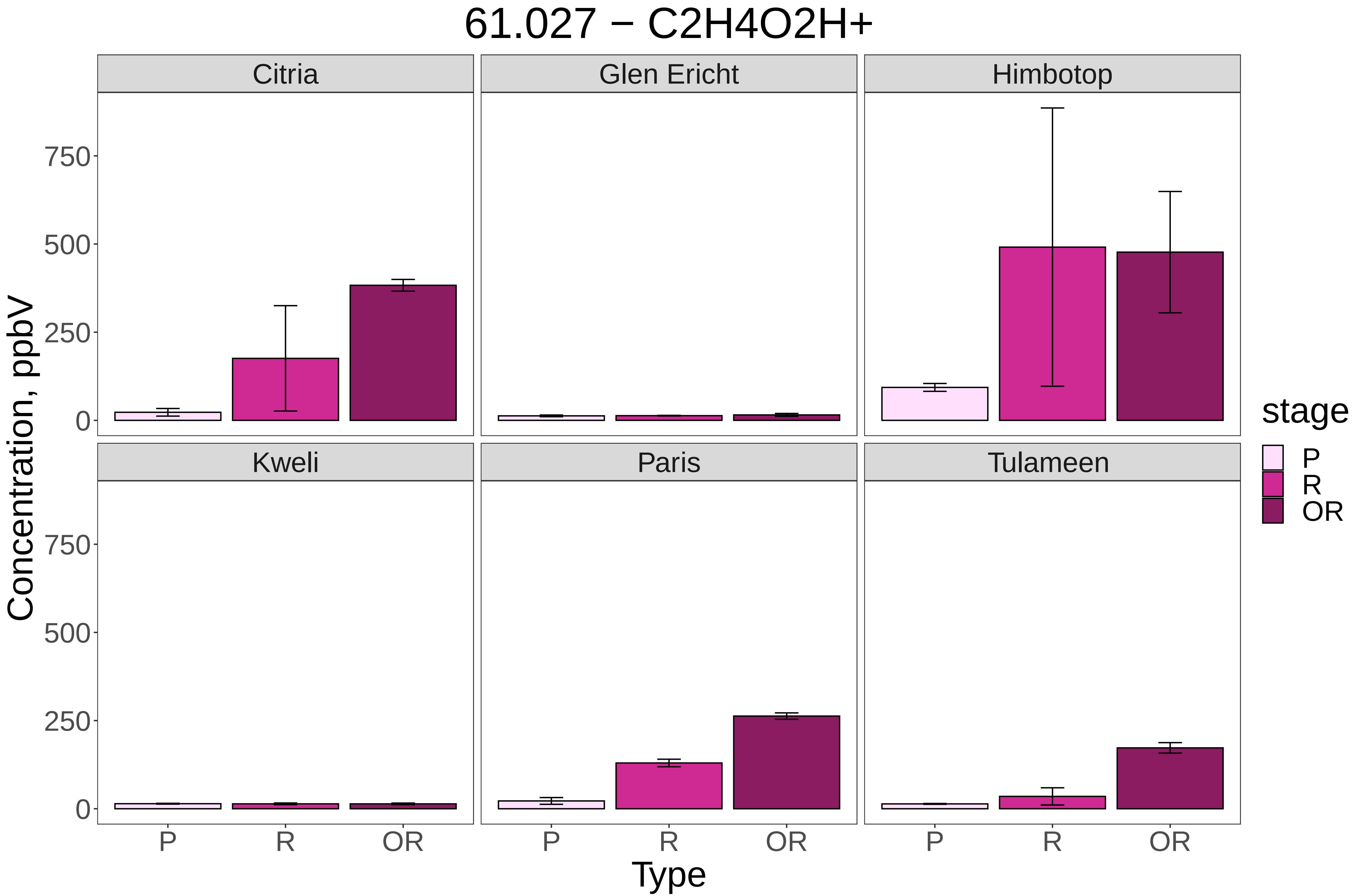

61.027 − C2H4O2H+
Citria
Glen Ericht
Himbotop
Kweli
Paris
Tulameen
Concentration, ppbV
0
250
500
750
Type
P
R
OR
stage
P
R
OR

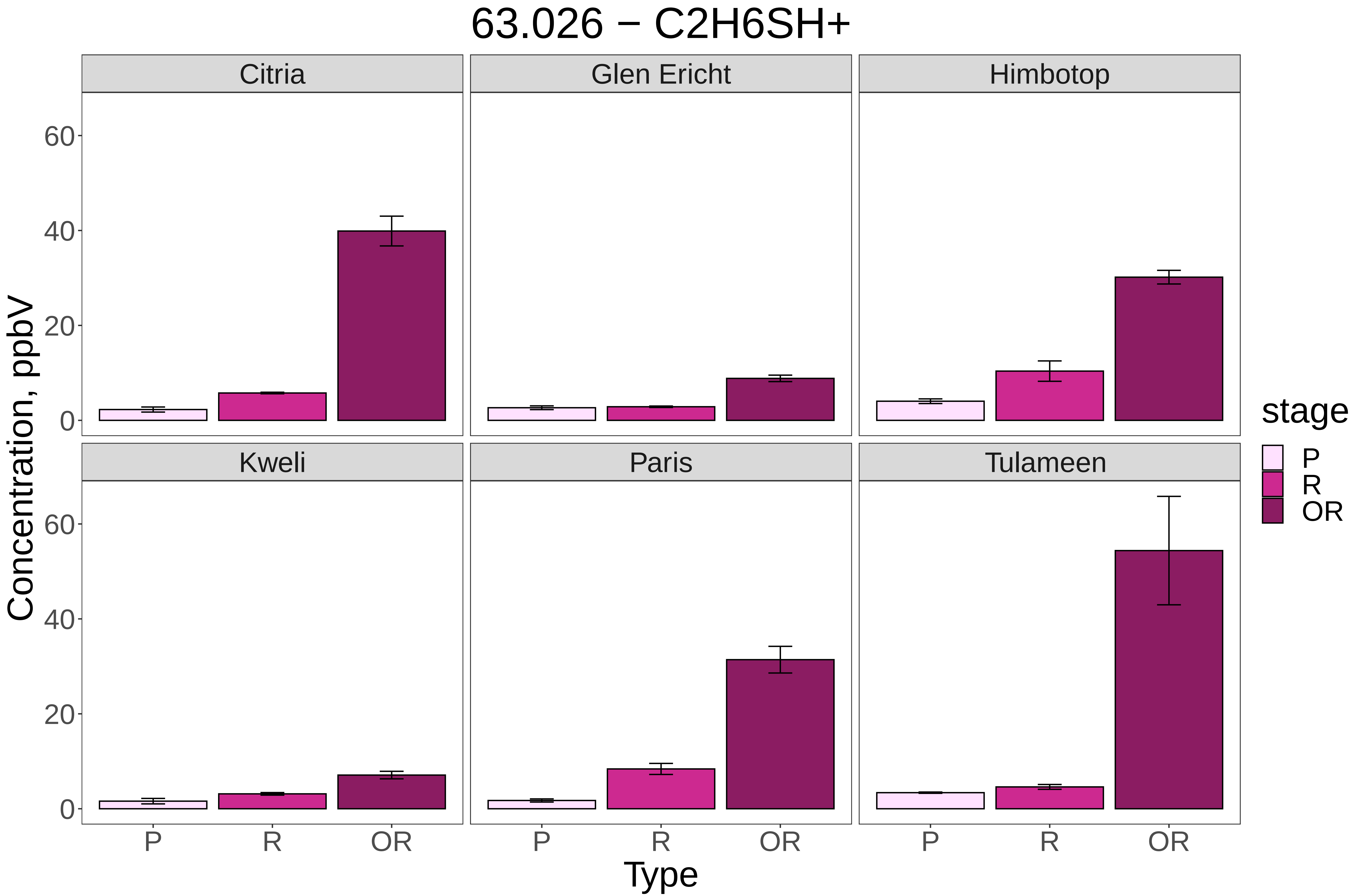

63.026 − C2H6SH+
Citria
Glen Ericht
Himbotop
Kweli
Paris
Tulameen
Concentration, ppbV
0
20
40
60
P
R
OR
Type
stage
P
R
OR

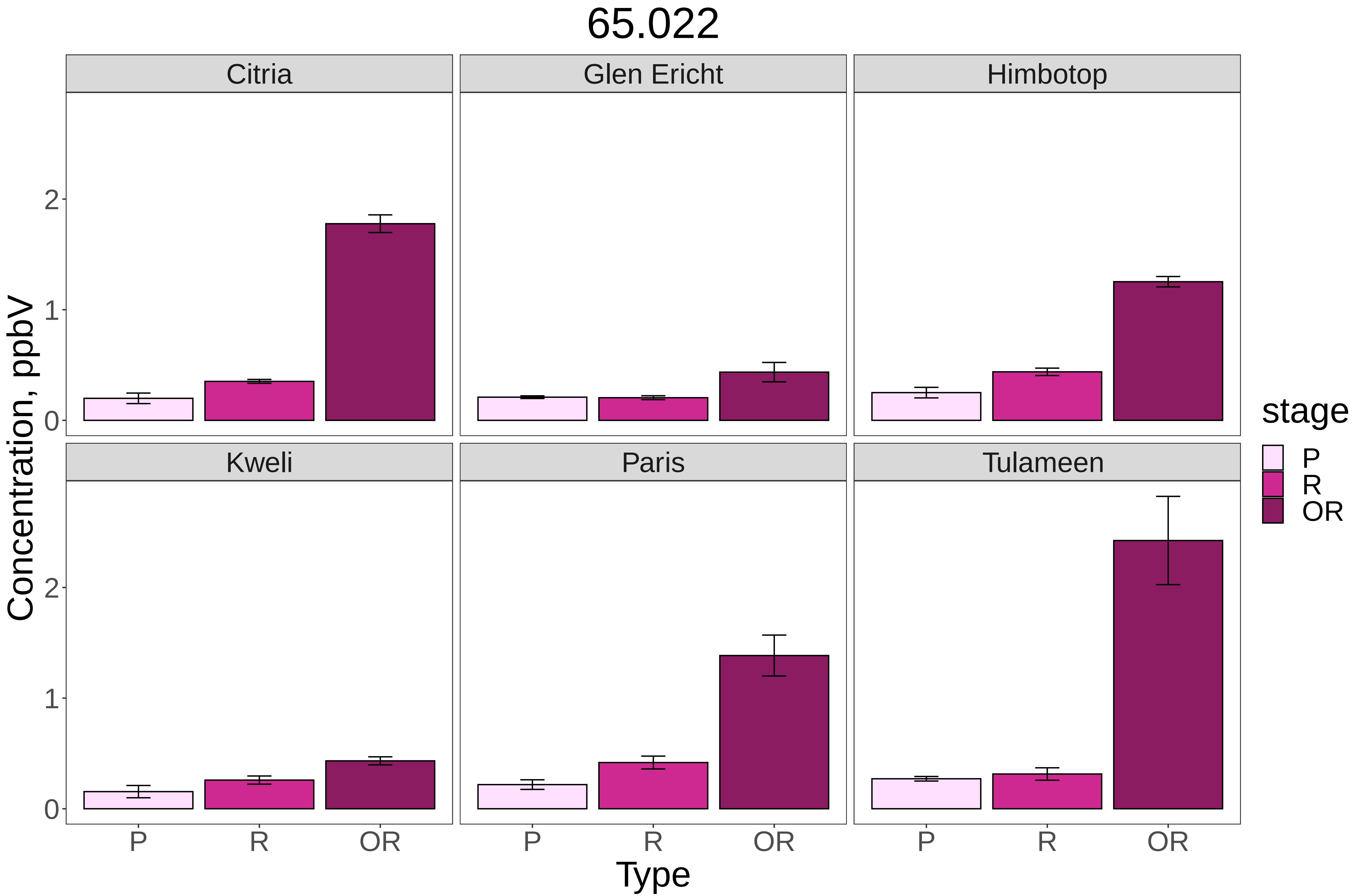

65.022
Citria
Glen Ericht
Himbotop
Kweli
Paris
Tulameen
Concentration, ppbV
0
1
2
P
R
OR
Type
stage
P
R
OR

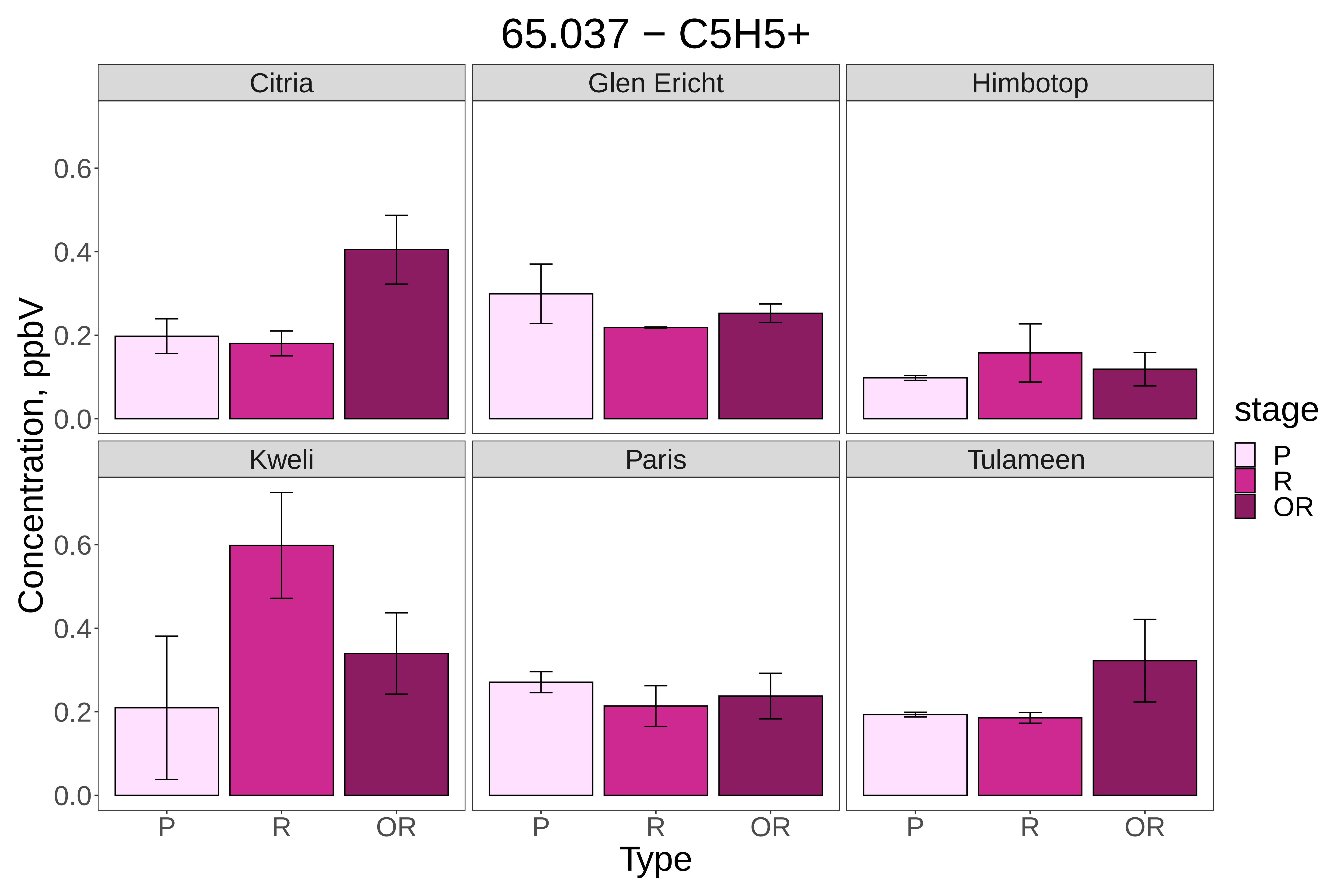

65.037 − C5H5+
Citria
Glen Ericht
Himbotop
Kweli
Paris
Tulameen
Concentration, ppbV
0.0
0.2
0.4
0.6
P
R
OR
Type
stage
P
R
OR

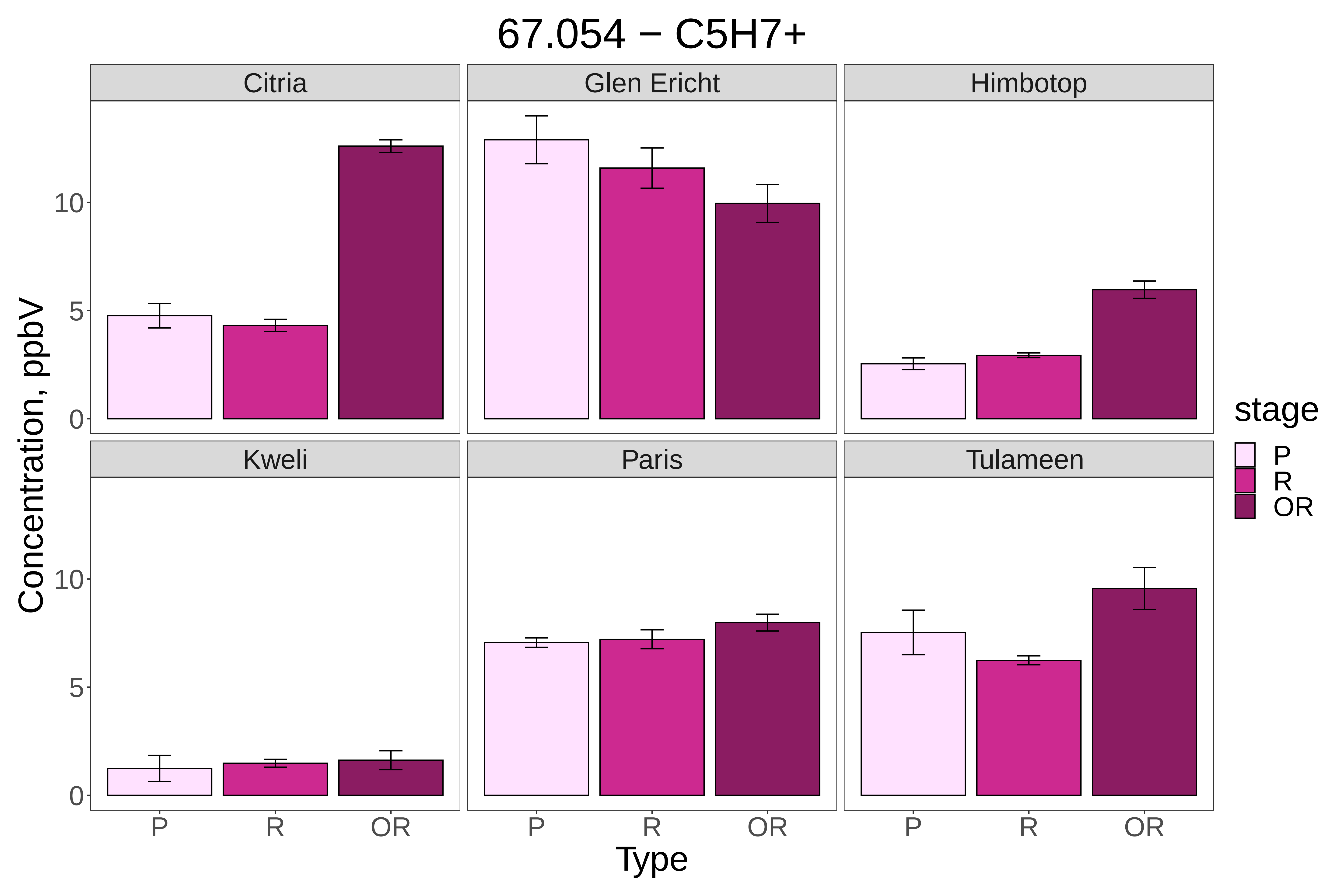
67.054 − C5H7+
Citria
Glen Ericht
Himbotop
Kweli
Paris
Tulameen
Concentration, ppbV
0
5
10
P
R
OR
Type
stage
P
R
OR

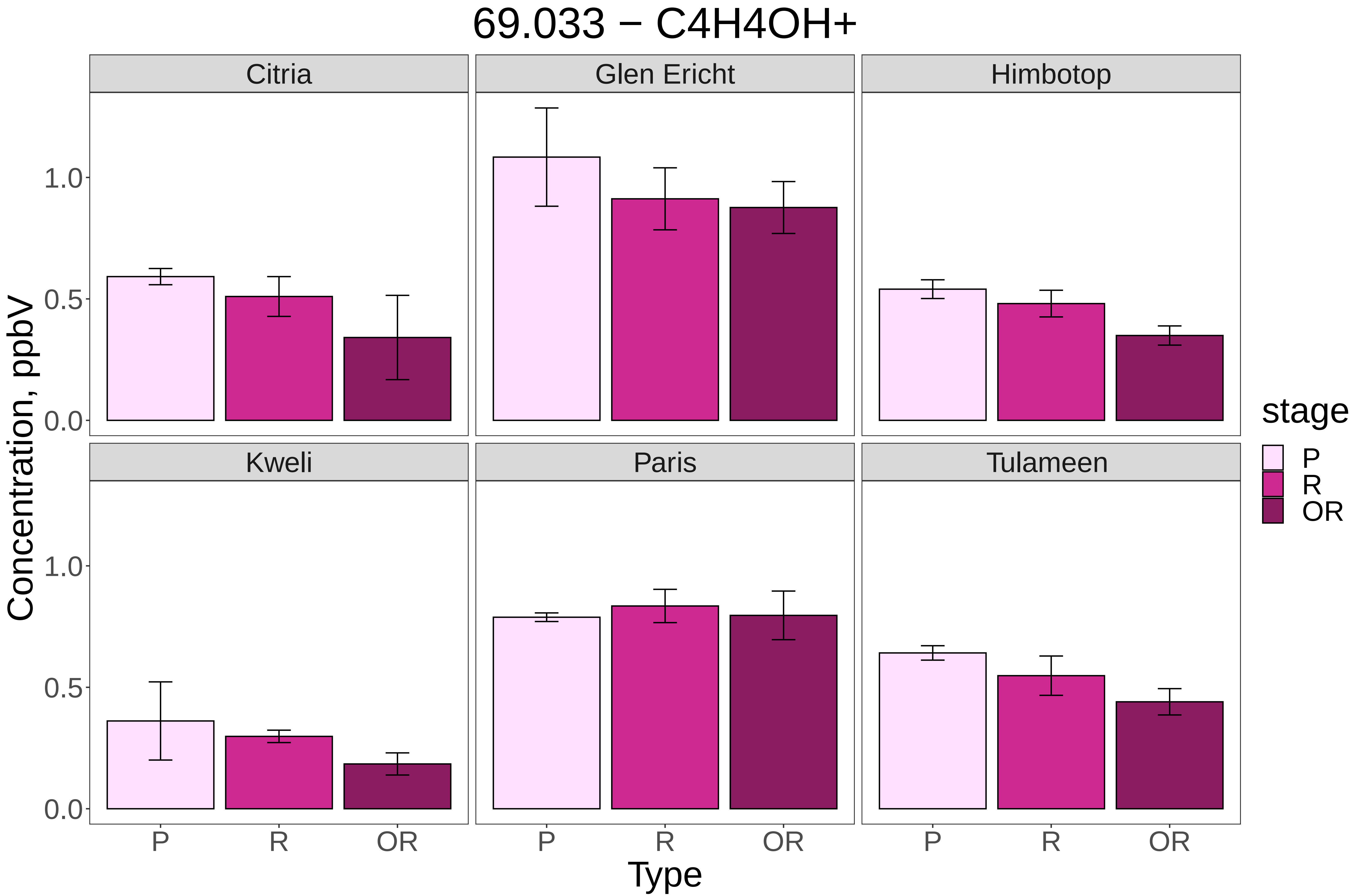
69.033 − C4H4OH+
Citria
Glen Ericht
Himbotop
Kweli
Paris
Tulameen
Concentration, ppbV
0.0
0.5
1.0
P
R
OR
Type
stage

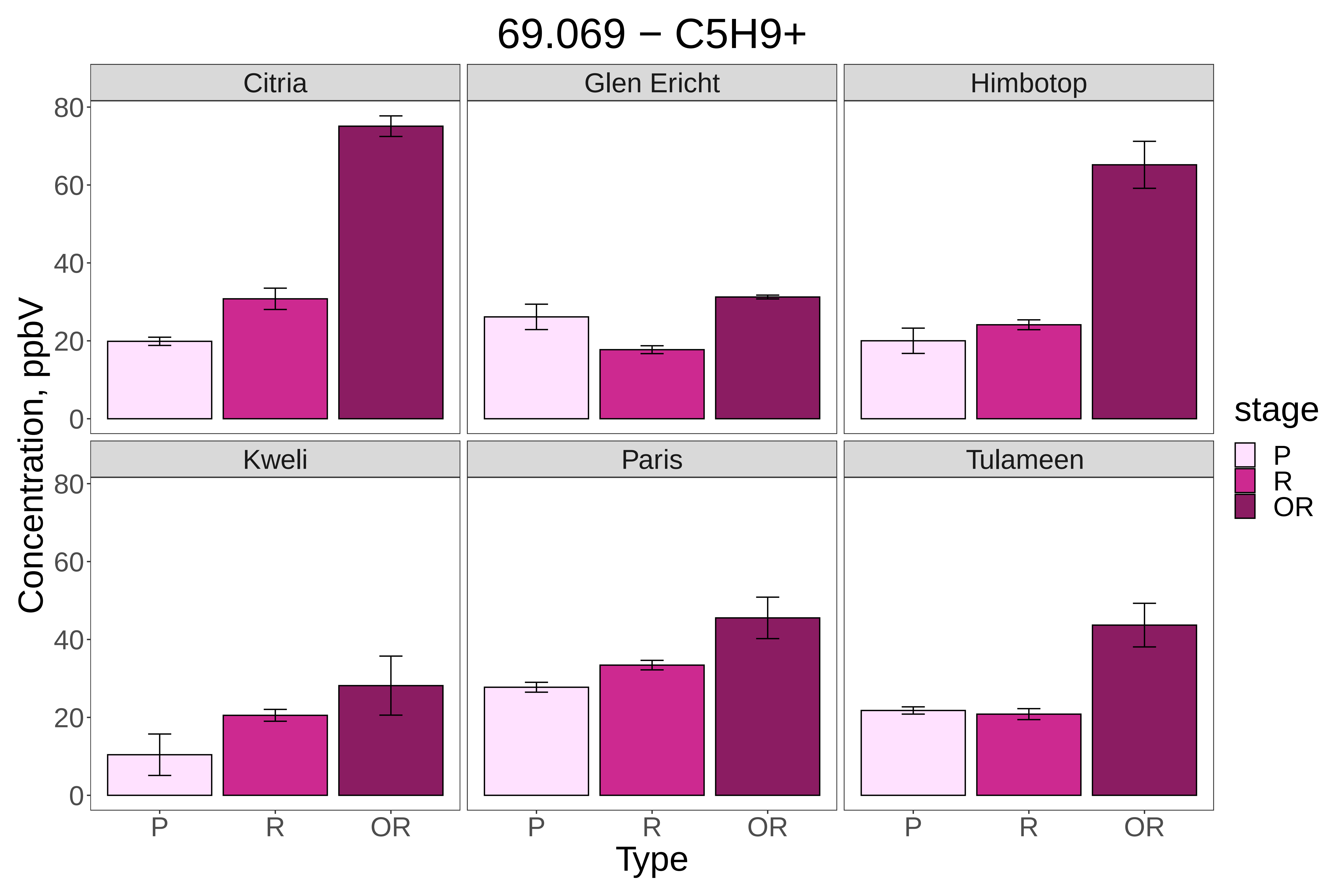
69.069 − C5H9+
Citria
Glen Ericht
Himbotop
Kweli
Paris
Tulameen
Concentration, ppbV
0
20
40
60
80
P
R
OR
Type
stage
P
R
OR

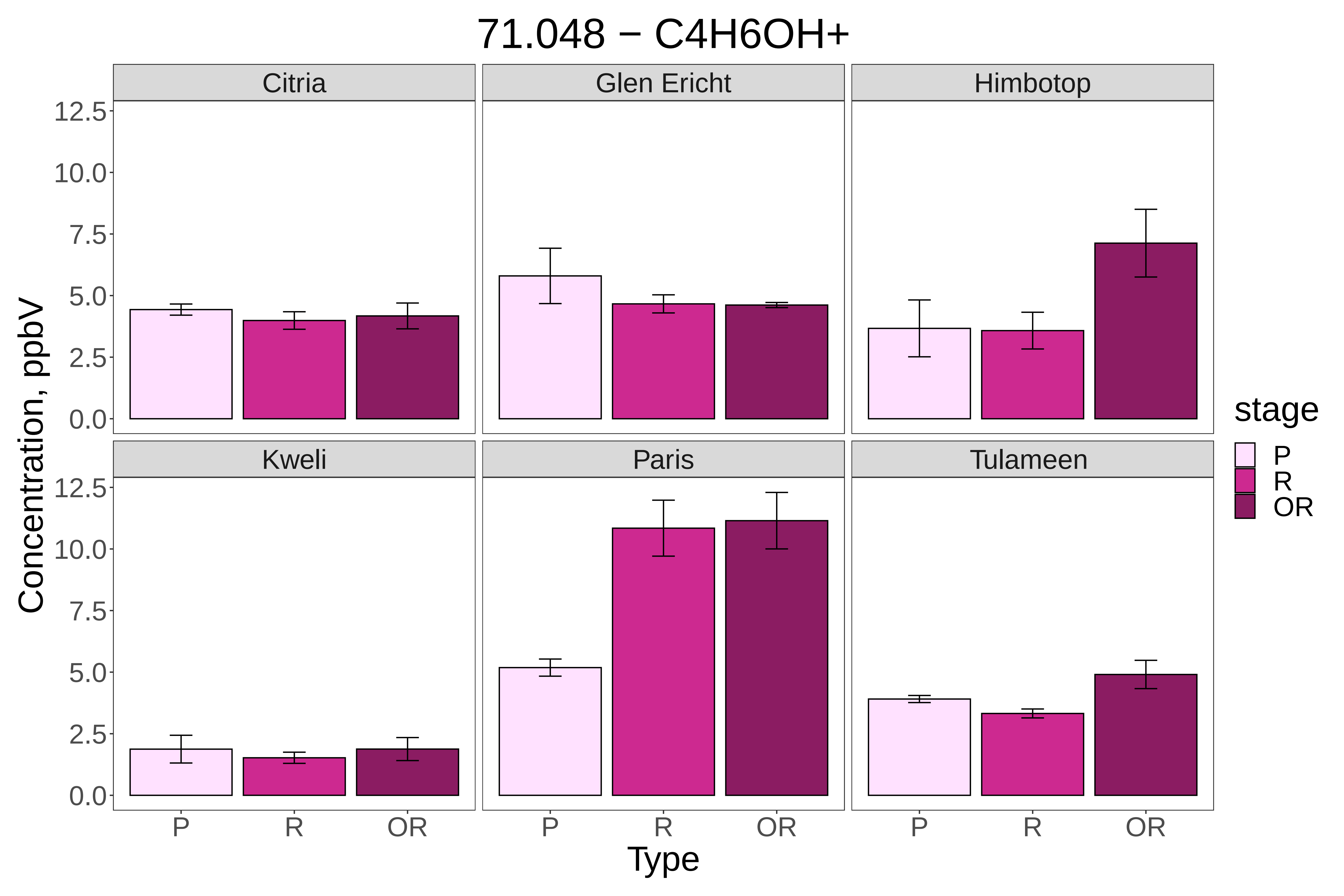

71.048 − C4H6OH+
Citria
Glen Ericht
Himbotop
Kweli
Paris
Tulameen
Concentration, ppbV
0.0
2.5
5.0
7.5
10.0
12.5
P
R
OR
Type
stage
P
R
OR

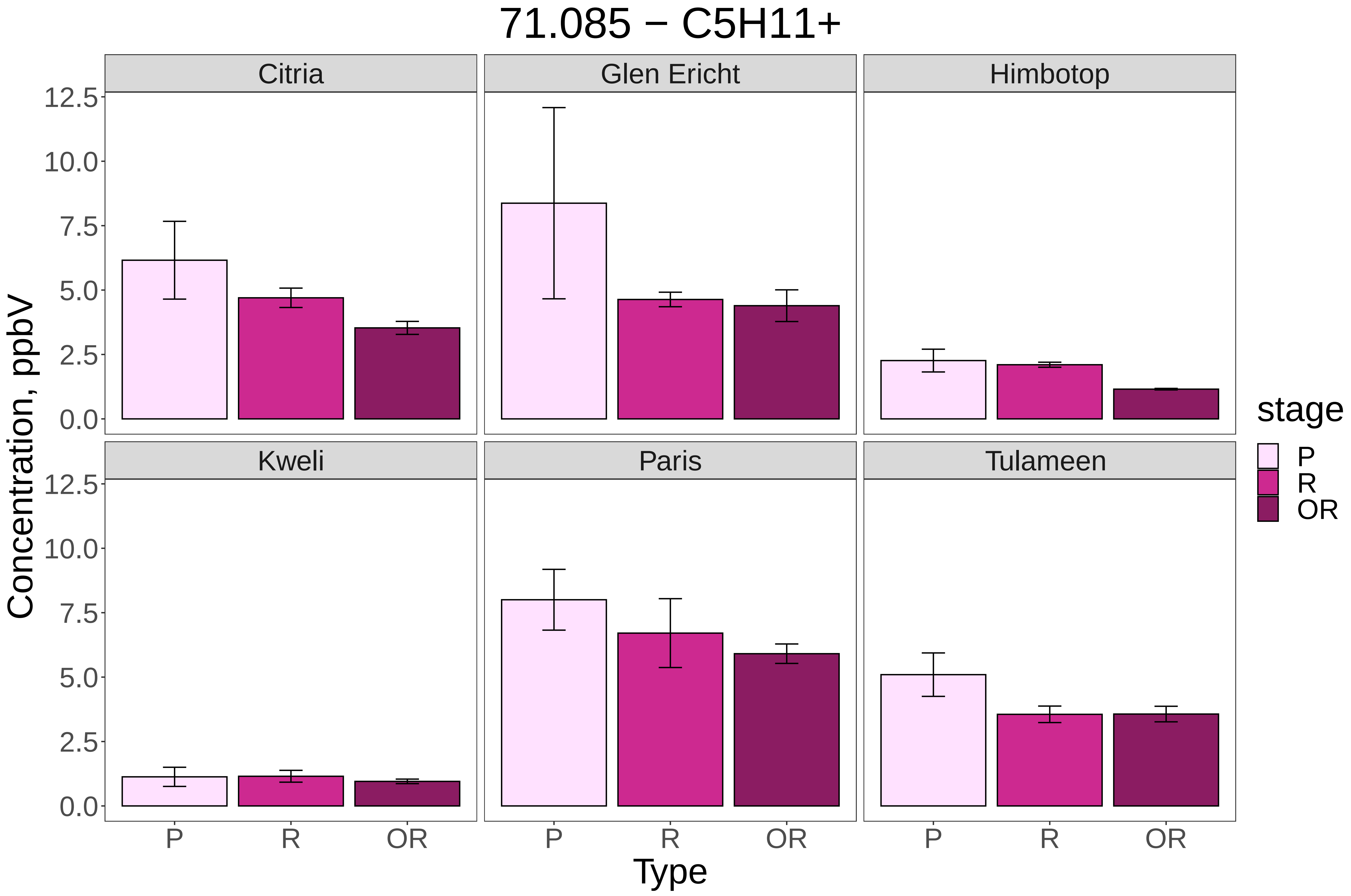

71.085 − C5H11+
Citria
Glen Ericht
Himbotop
Kweli
Paris
Tulameen
Concentration, ppbV
0.0
2.5
5.0
7.5
10.0
12.5
P
R
OR
Type
stage

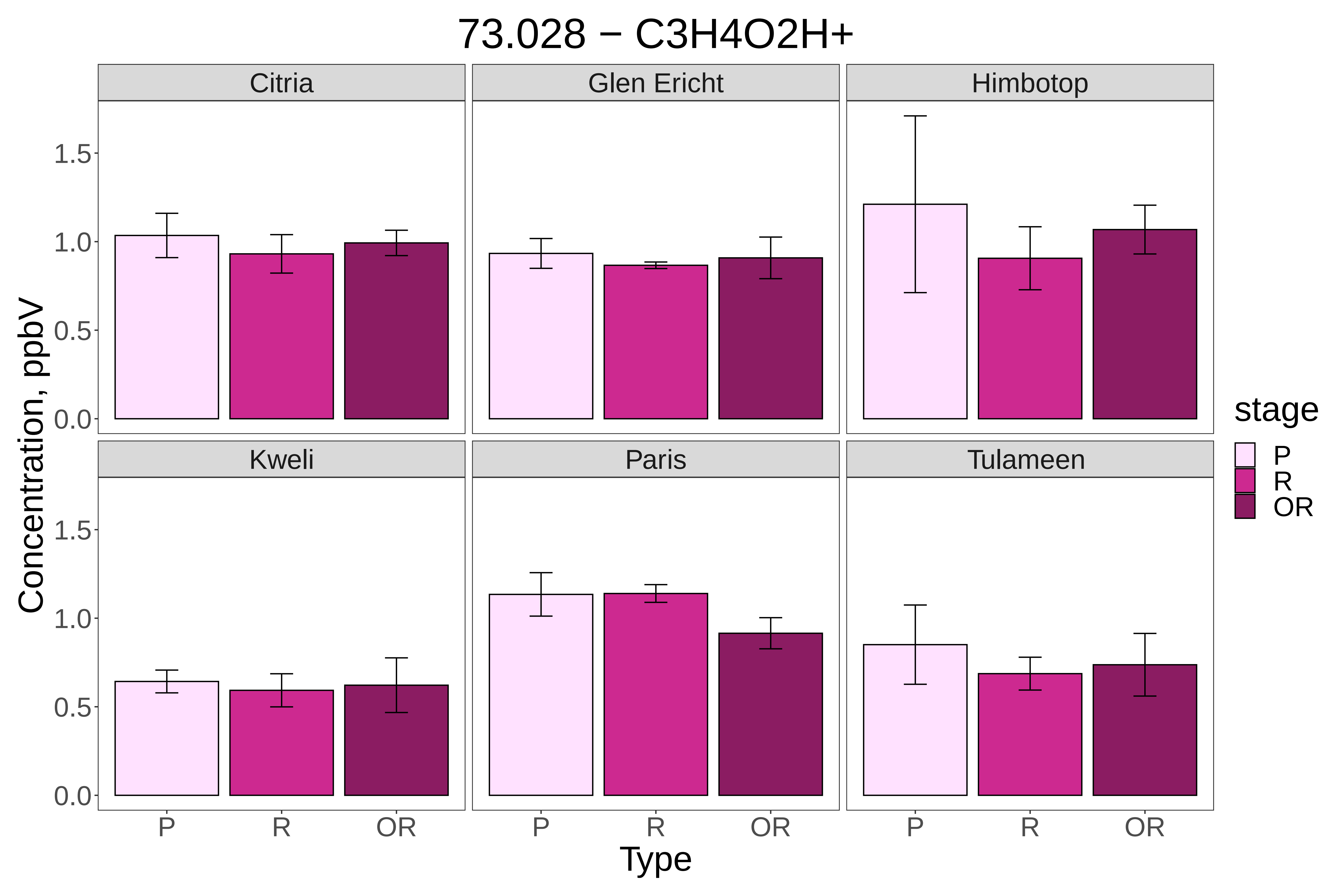

73.028 − C3H4O2H+
Citria
Glen Ericht
Himbotop
Kweli
Paris
Tulameen
Concentration, ppbV
0.0
0.5
1.0
1.5
P
R
OR
Type
stage
P
R
OR

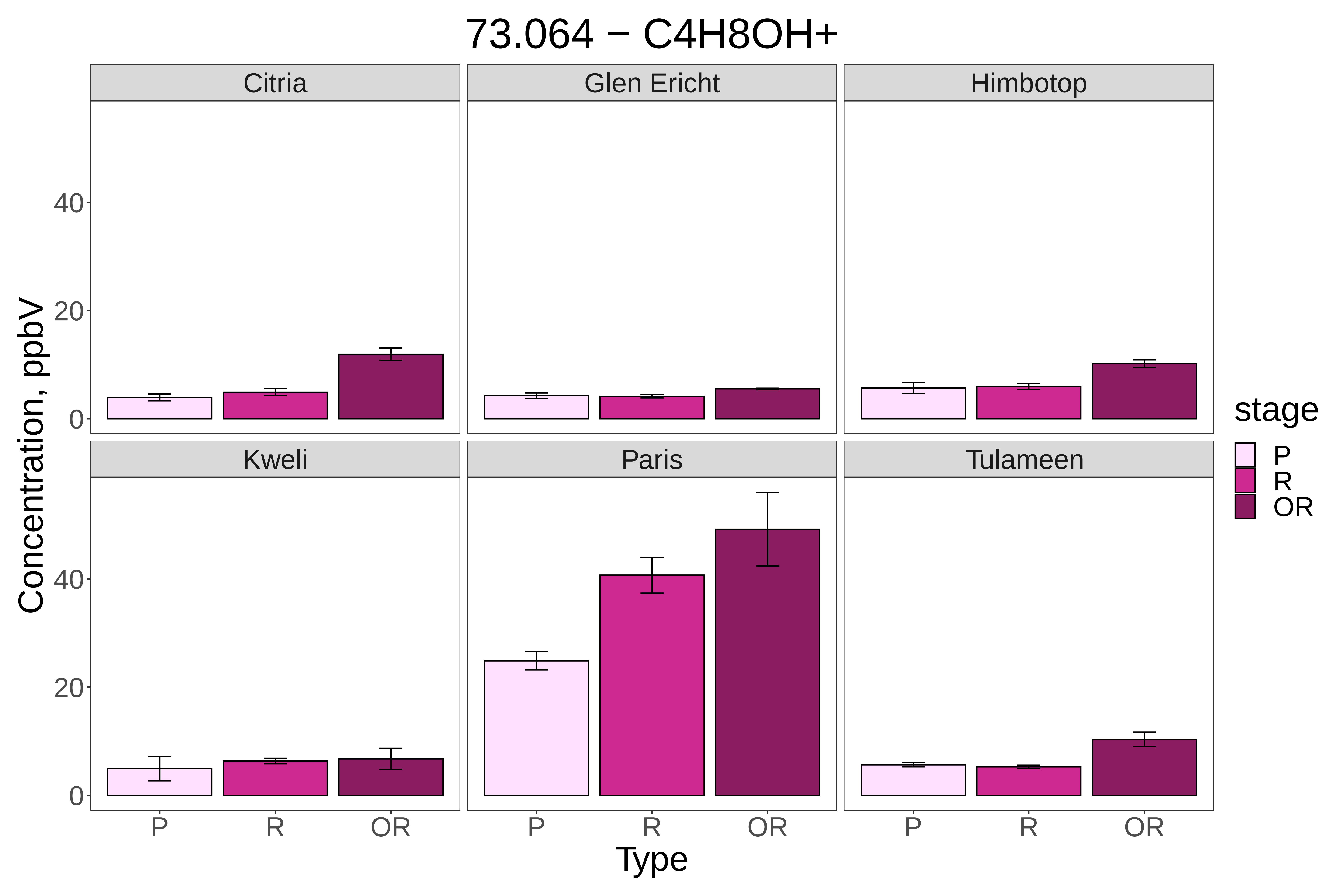

73.064 − C4H8OH+
Citria
Glen Ericht
Himbotop
Kweli
Paris
Tulameen
Concentration, ppbV
0
20
40
P
R
OR
Type
stage
P
R
OR

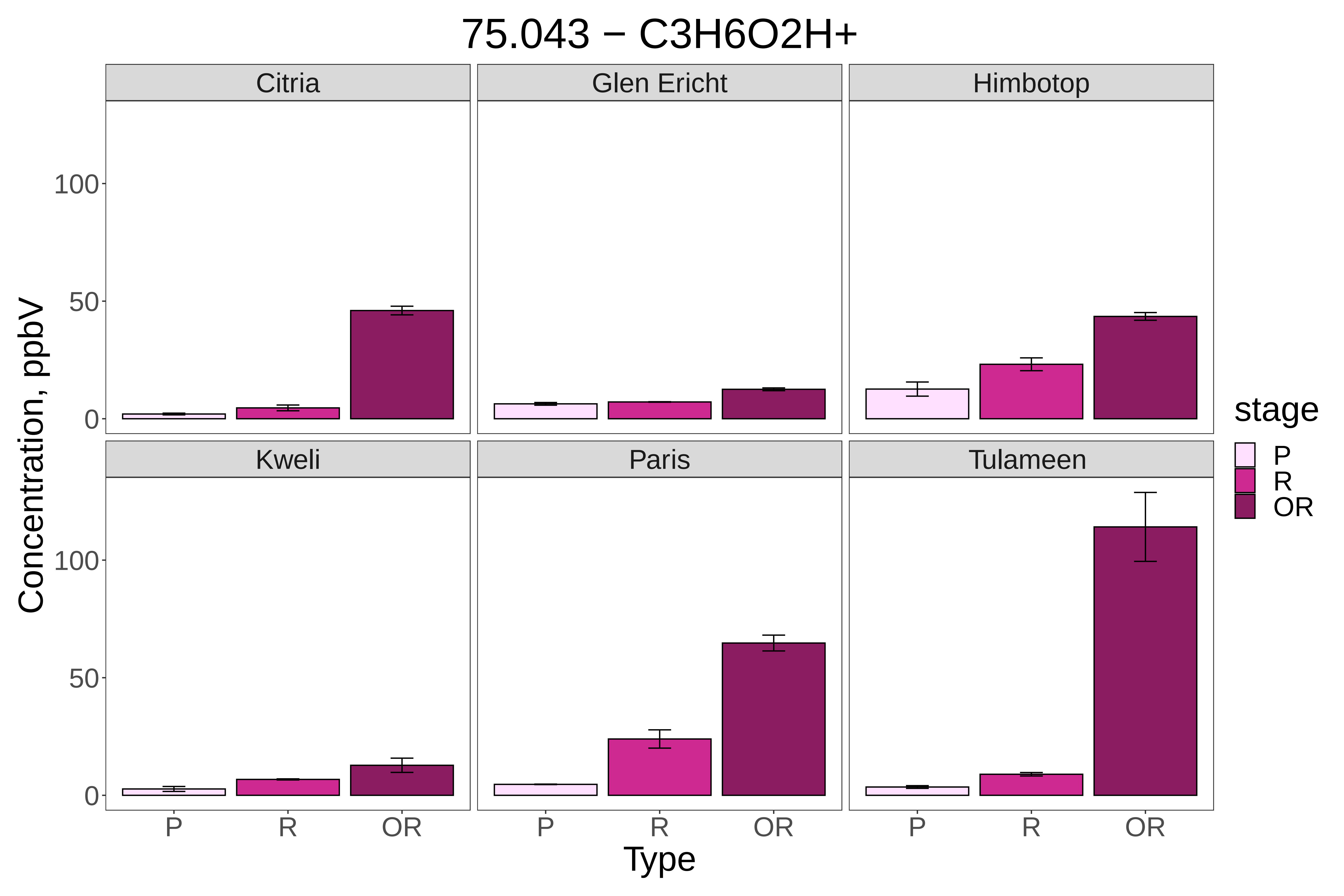
75.043 − C3H6O2H+
Citria
Glen Ericht
Himbotop
Kweli
Paris
Tulameen
Concentration, ppbV
0
50
100
P
R
OR
Type
stage
P
R
OR

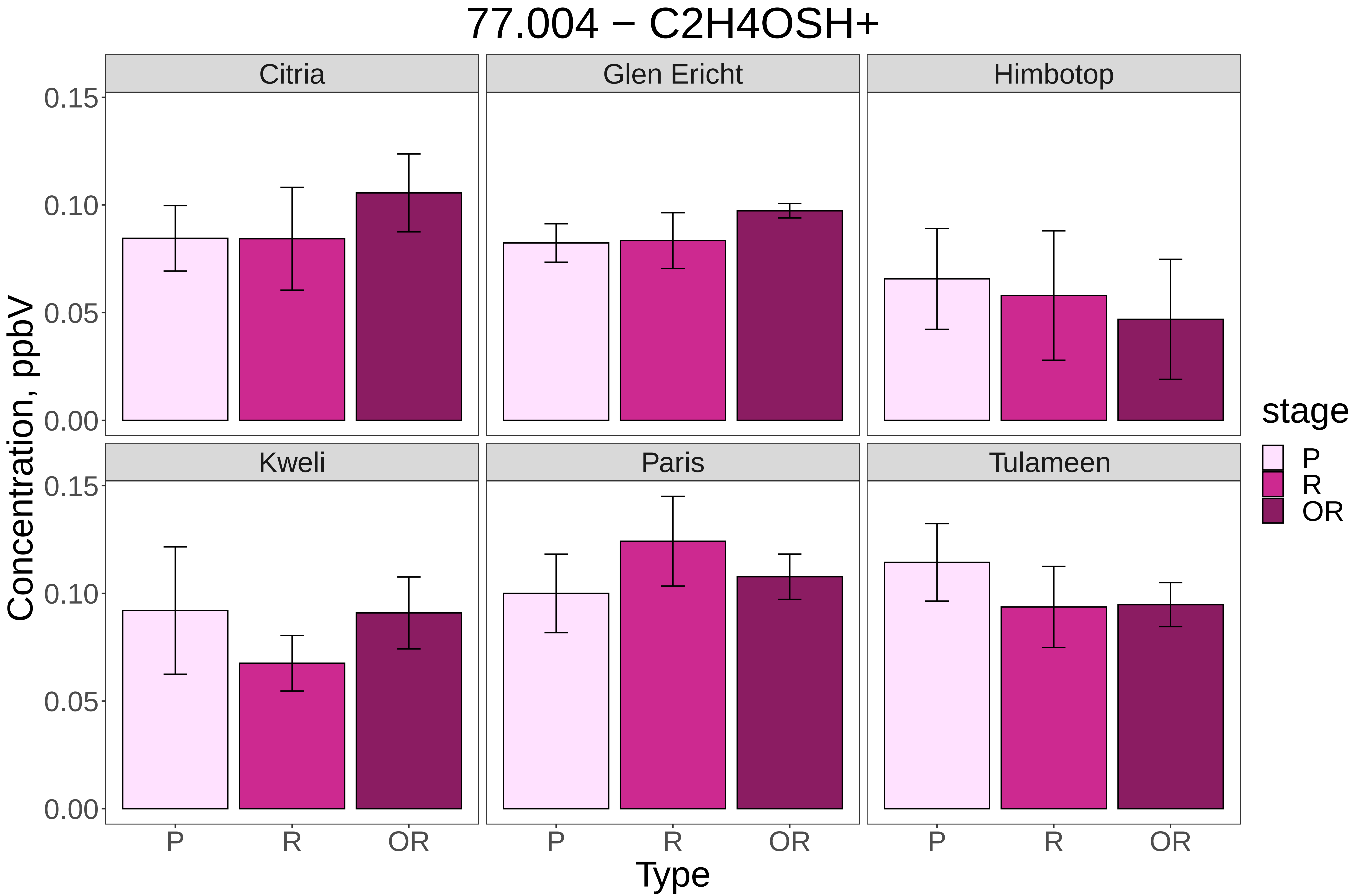

77.004 − C2H4OSH+
Citria
Glen Ericht
Himbotop
Kweli
Paris
Tulameen
Concentration, ppbV
0.15
0.10
0.05
0.00
P
R
OR
Type
stage
P
R
OR

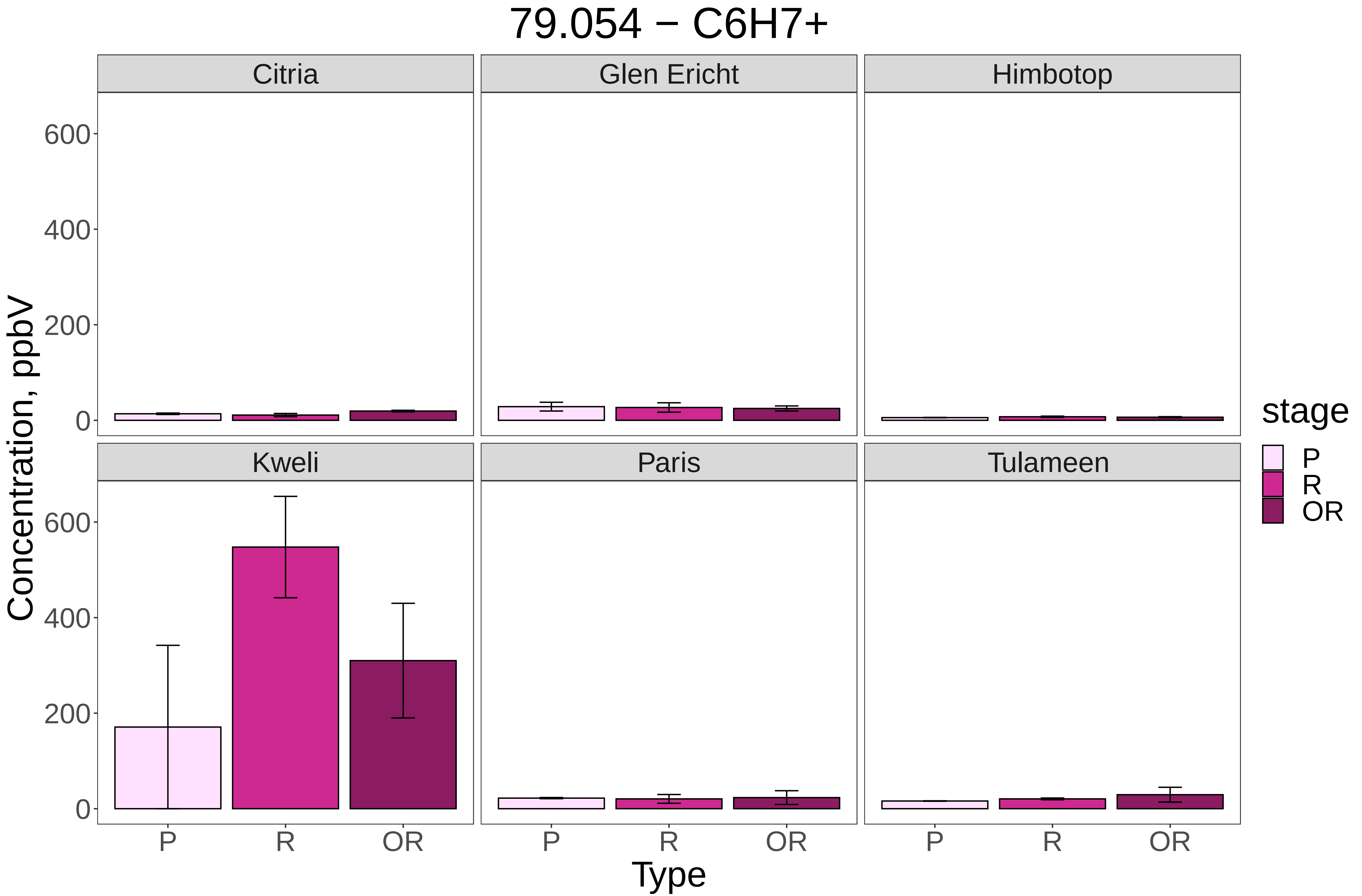

79.054 − C6H7+
Citria
Glen Ericht
Himbotop
Kweli
Paris
Tulameen
Concentration, ppbV
0
200
400
600
P
R
OR
Type
stage
P
R
OR

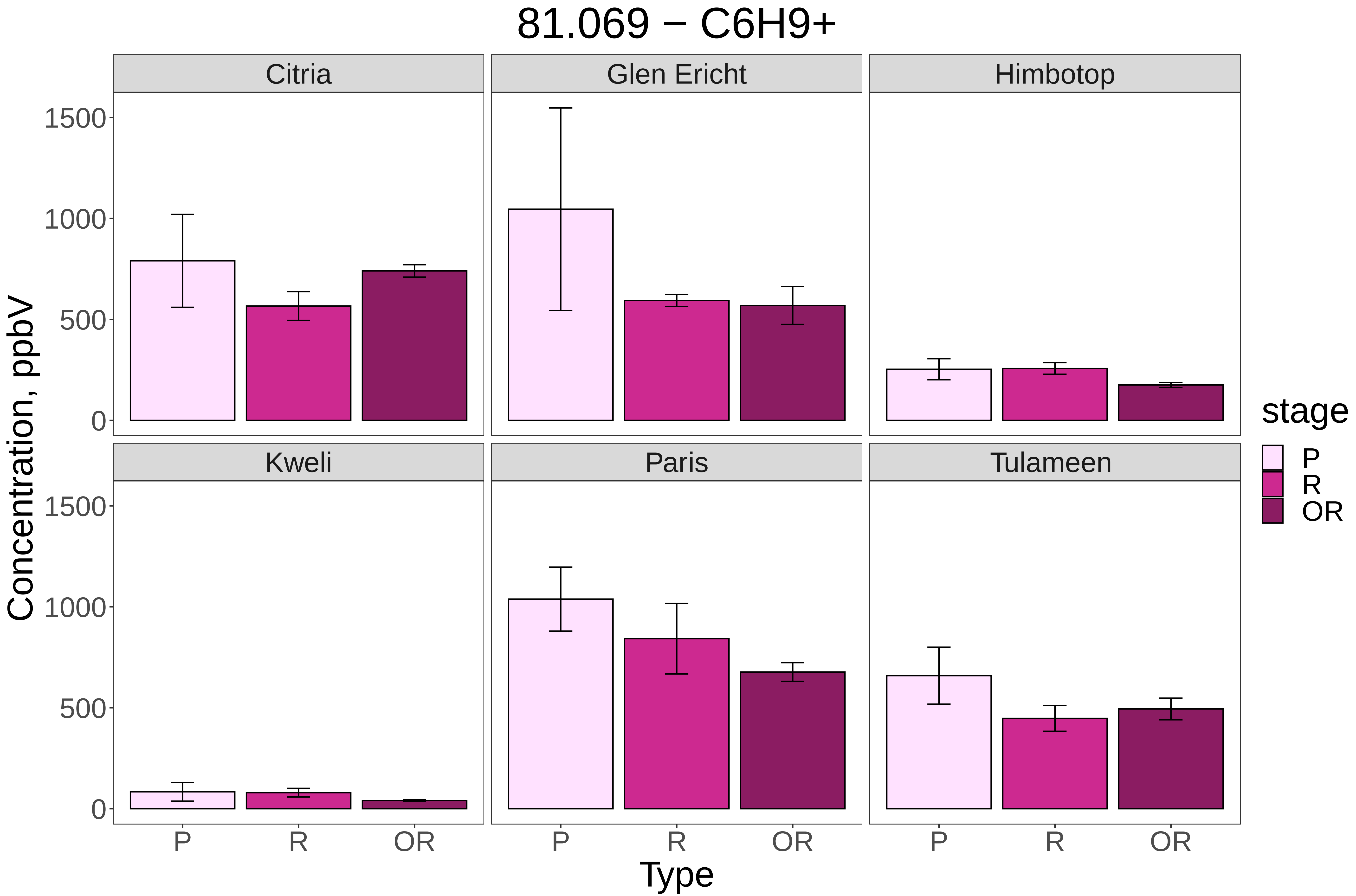
81.069 − C6H9+
Citria
Glen Ericht
Himbotop
Kweli
Paris
Tulameen
Concentration, ppbV
0
500
1000
1500
P
R
OR
Type
stage
P
R
OR

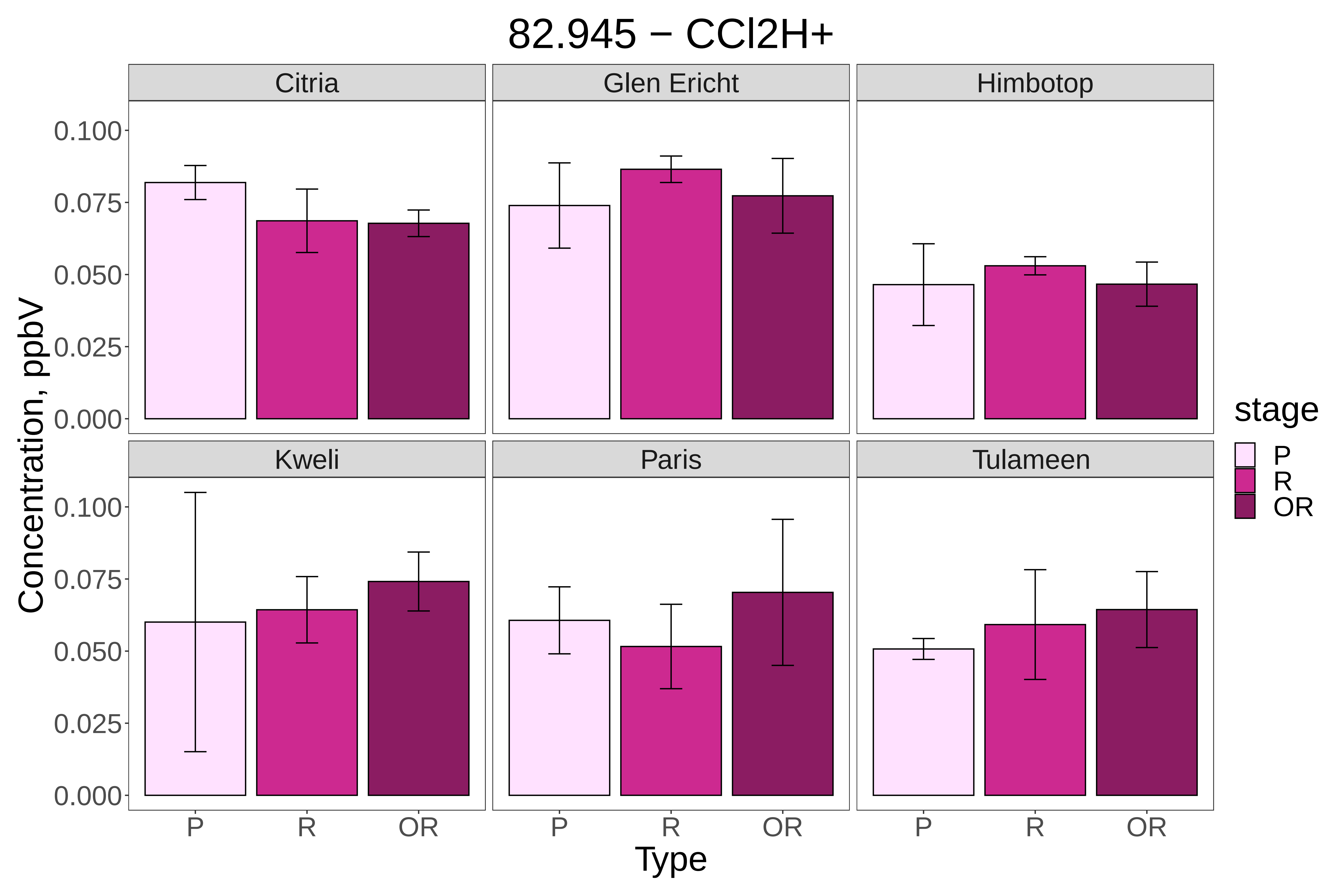

82.945 − CCl2H+
Citria
Glen Ericht
Himbotop
Kweli
Paris
Tulameen
Concentration, ppbV
0.000
0.025
0.050
0.075
0.100
P
R
OR
Type
stage
P
R
OR

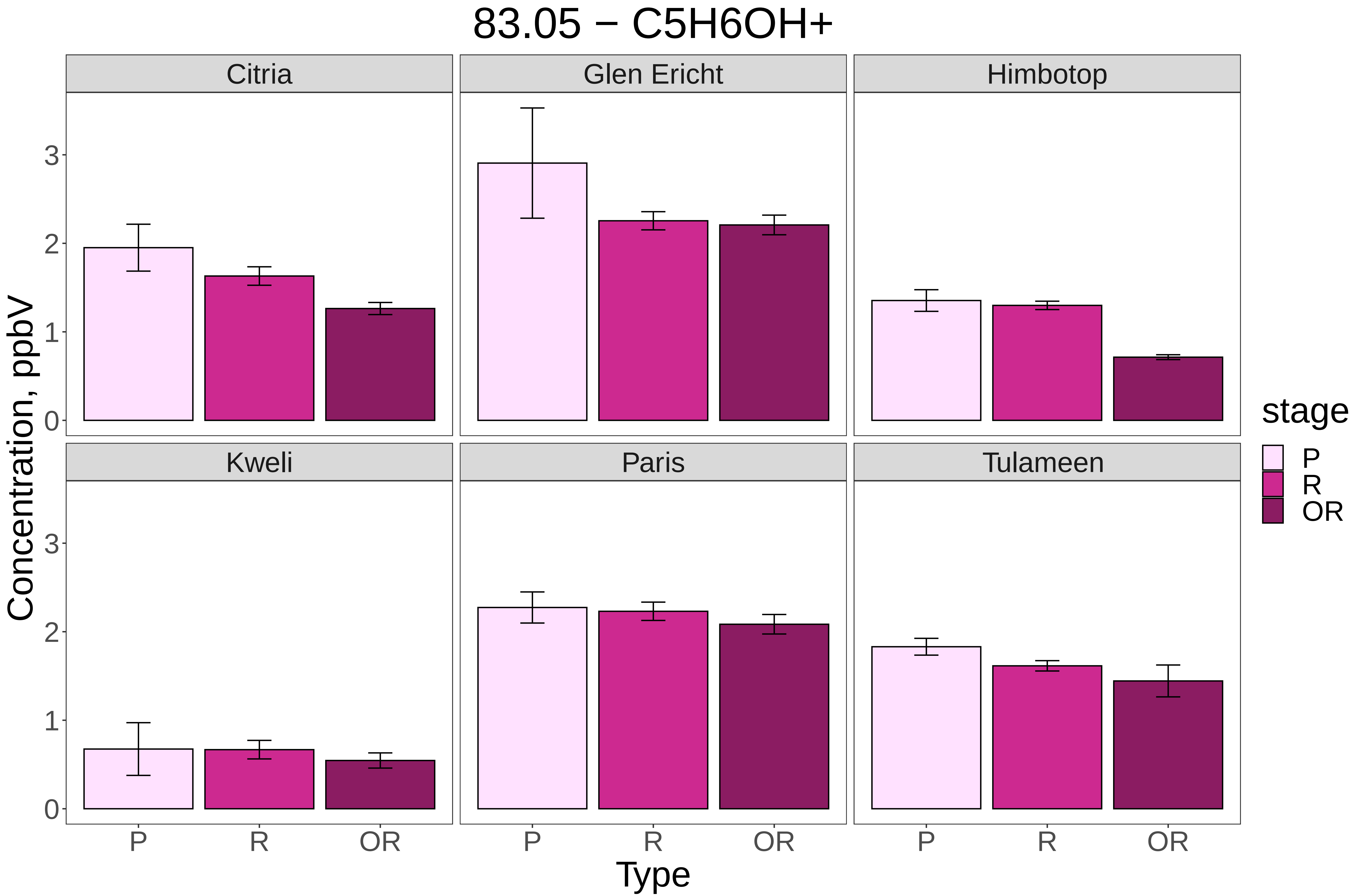

83.05 − C5H6OH+
Citria
Glen Ericht
Himbotop
Kweli
Paris
Tulameen
Concentration, ppbV
0
1
2
3
P
R
OR
Type
stage
P
R
OR

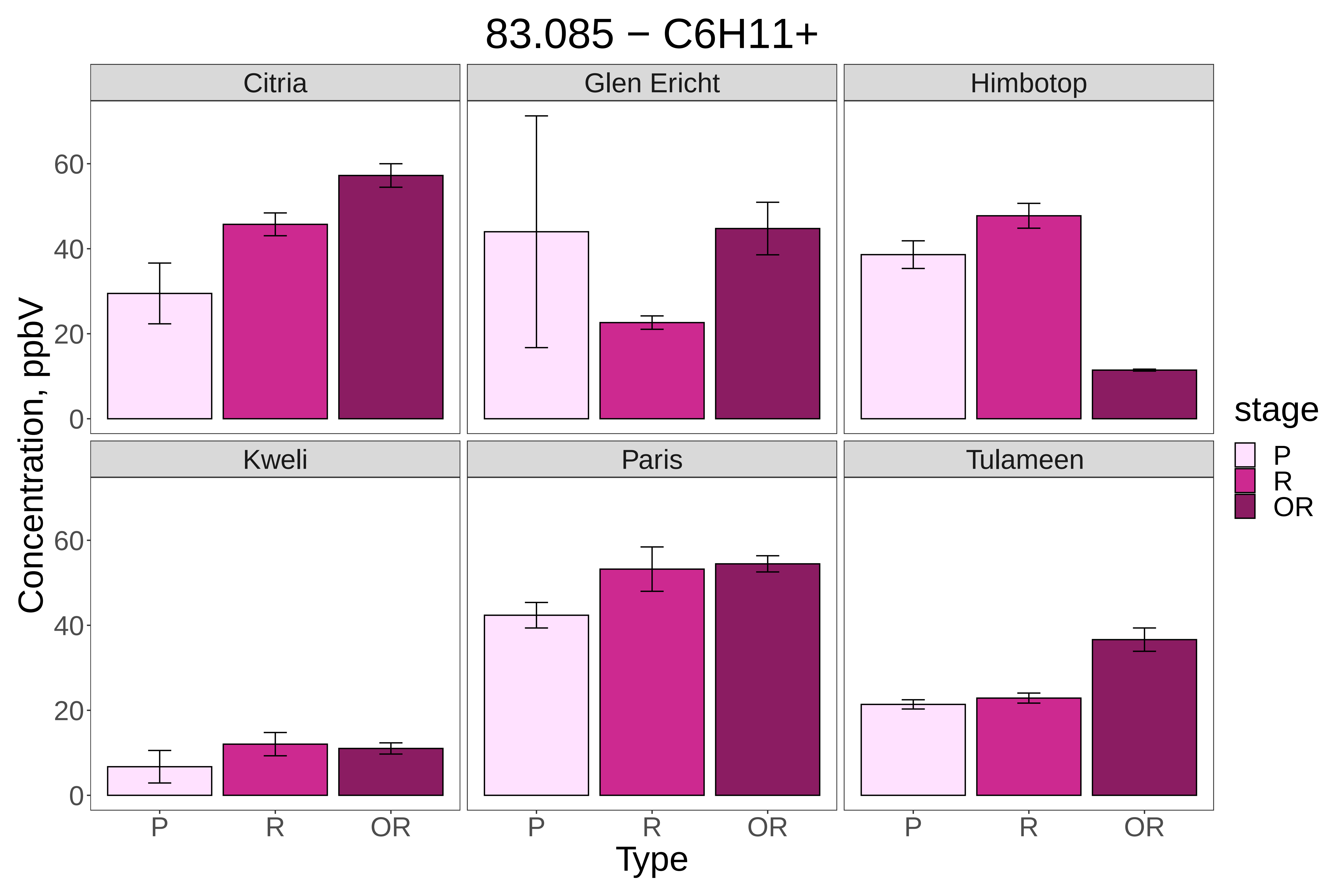

83.085 − C6H11+
Citria
Glen Ericht
Himbotop
Kweli
Paris
Tulameen
Concentration, ppbV
0
20
40
60
P
R
OR
Type
stage
P
R
OR

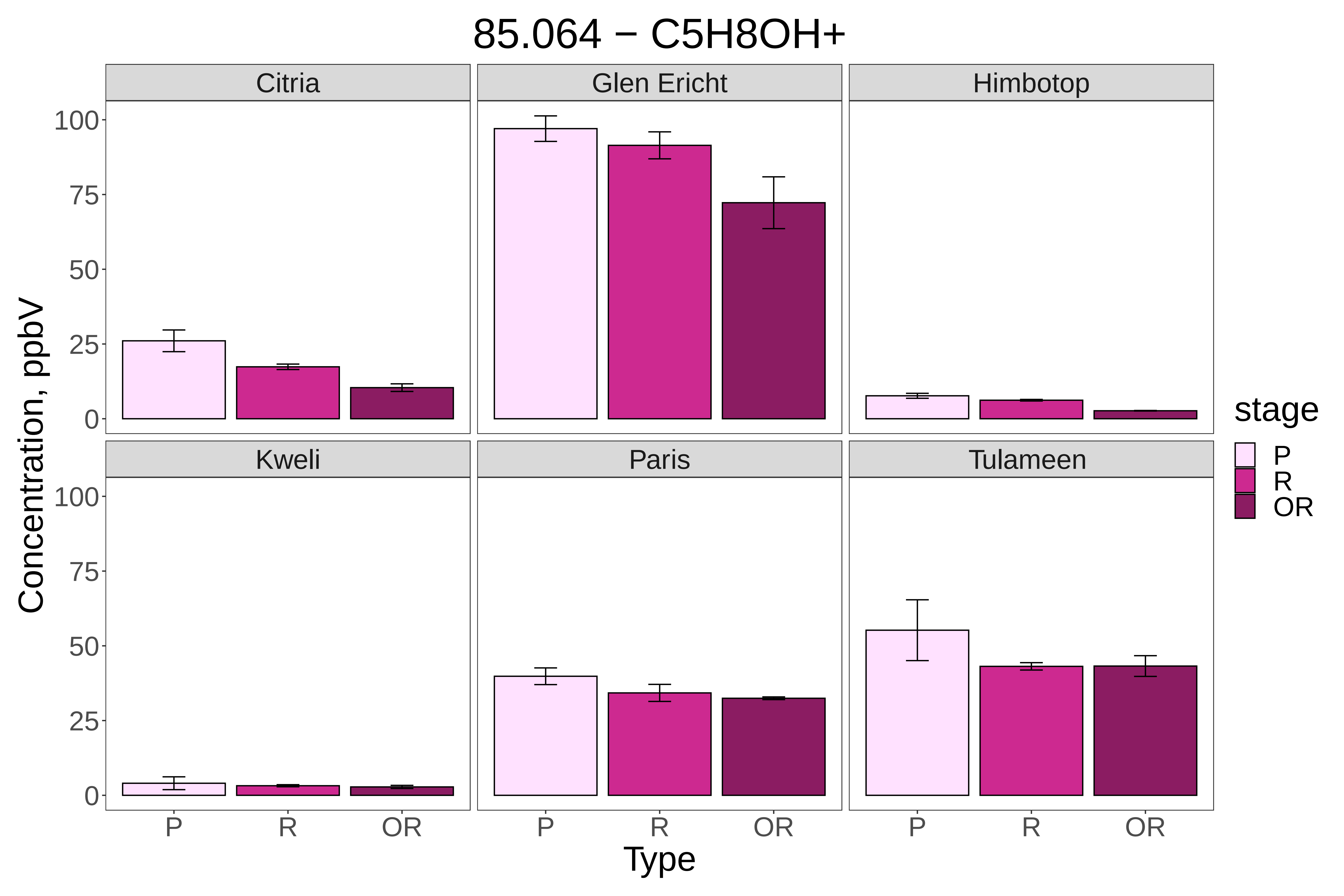
85.064 − C5H8OH+
Citria
Glen Ericht
Himbotop
Kweli
Paris
Tulameen
Concentration, ppbV
0
25
50
75
100
Type
P
R
OR
stage
P
R
OR

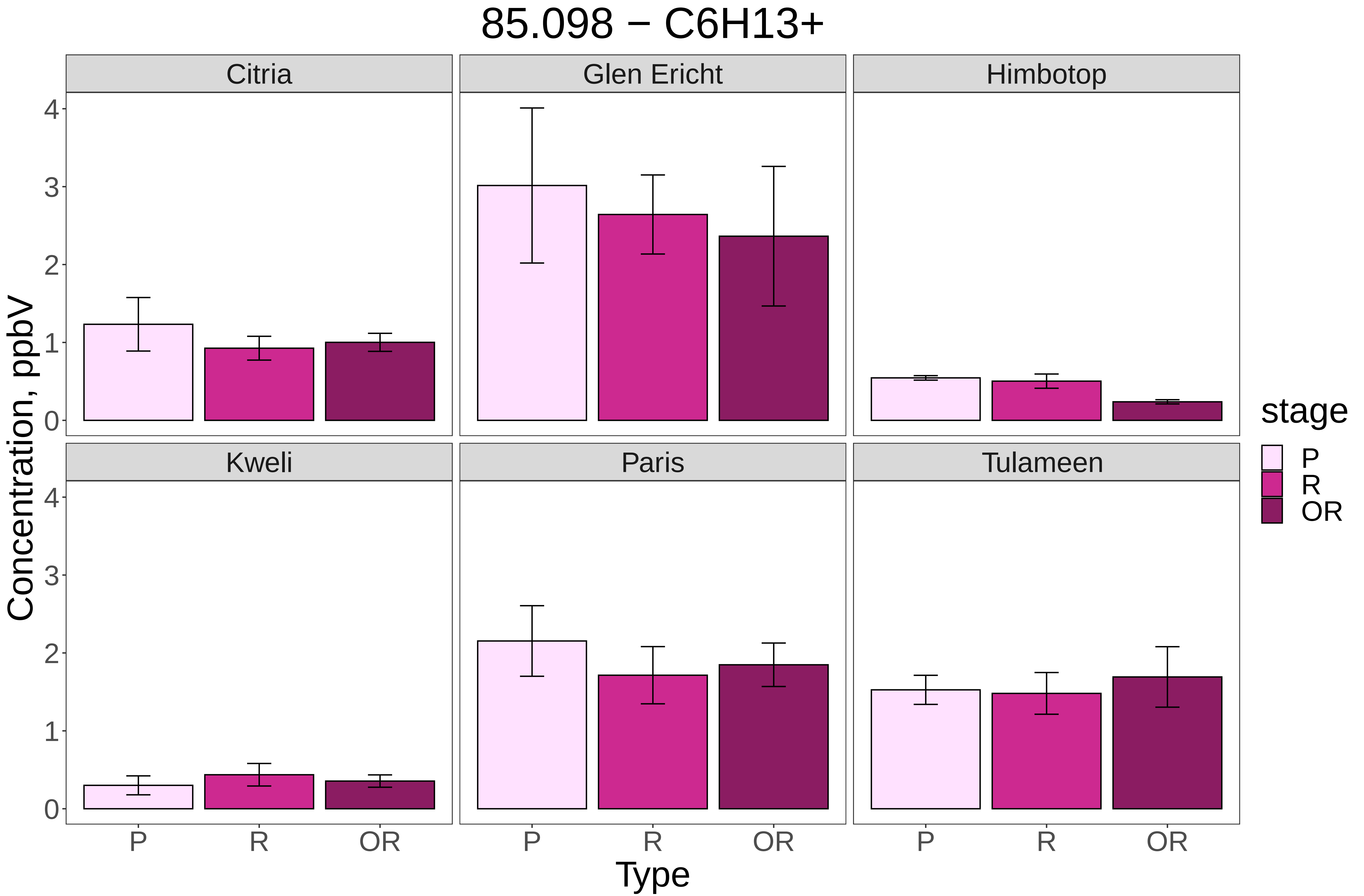

85.098 − C6H13+
Citria
Glen Ericht
Himbotop
Kweli
Paris
Tulameen
Concentration, ppbV
0
1
2
3
4
P
R
OR
Type
stage
P
R
OR

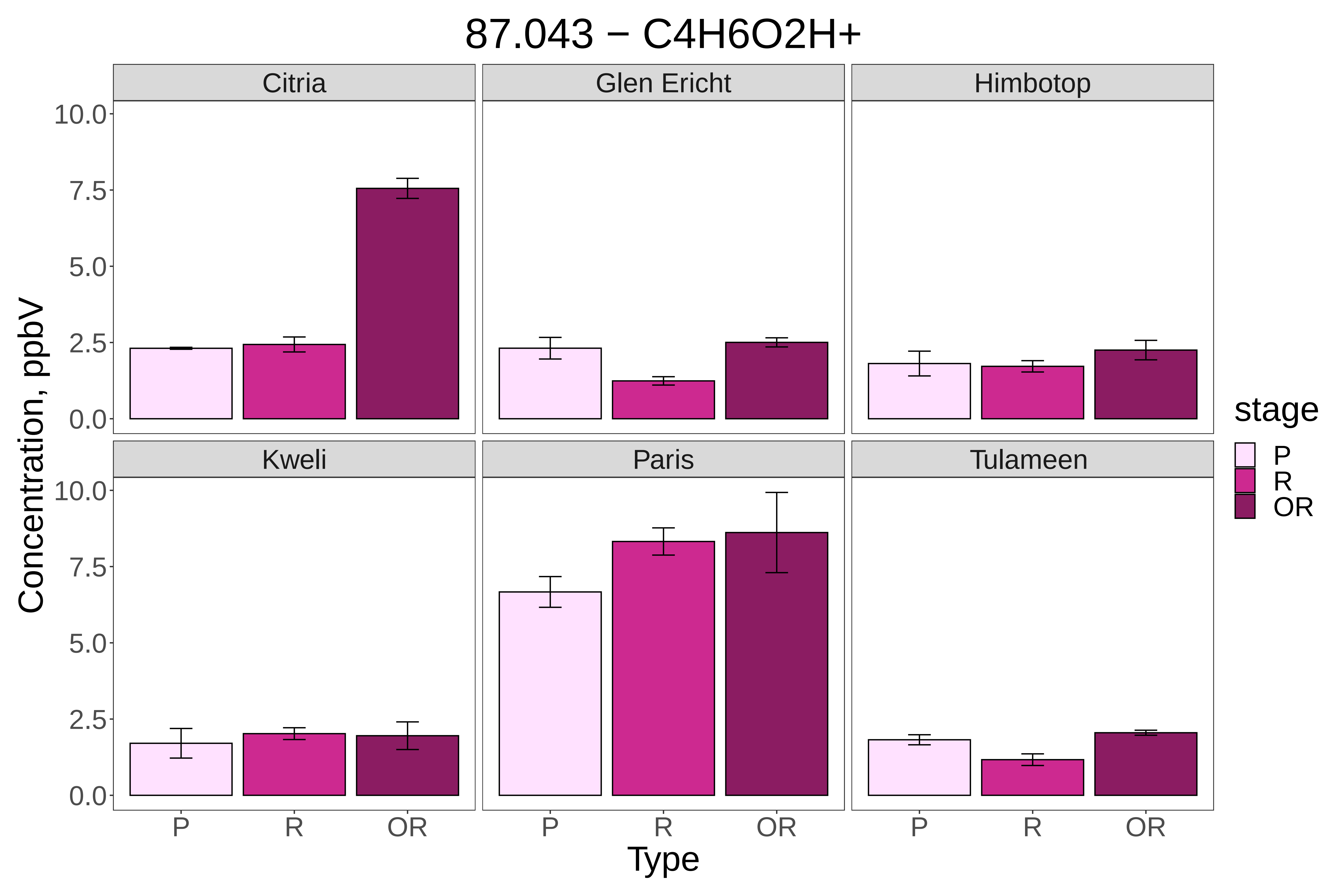

87.043 − C4H6O2H+
Citria
Glen Ericht
Himbotop
Kweli
Paris
Tulameen
Concentration, ppbV
0.0
2.5
5.0
7.5
10.0
Type
P
R
OR
stage
P
R
OR

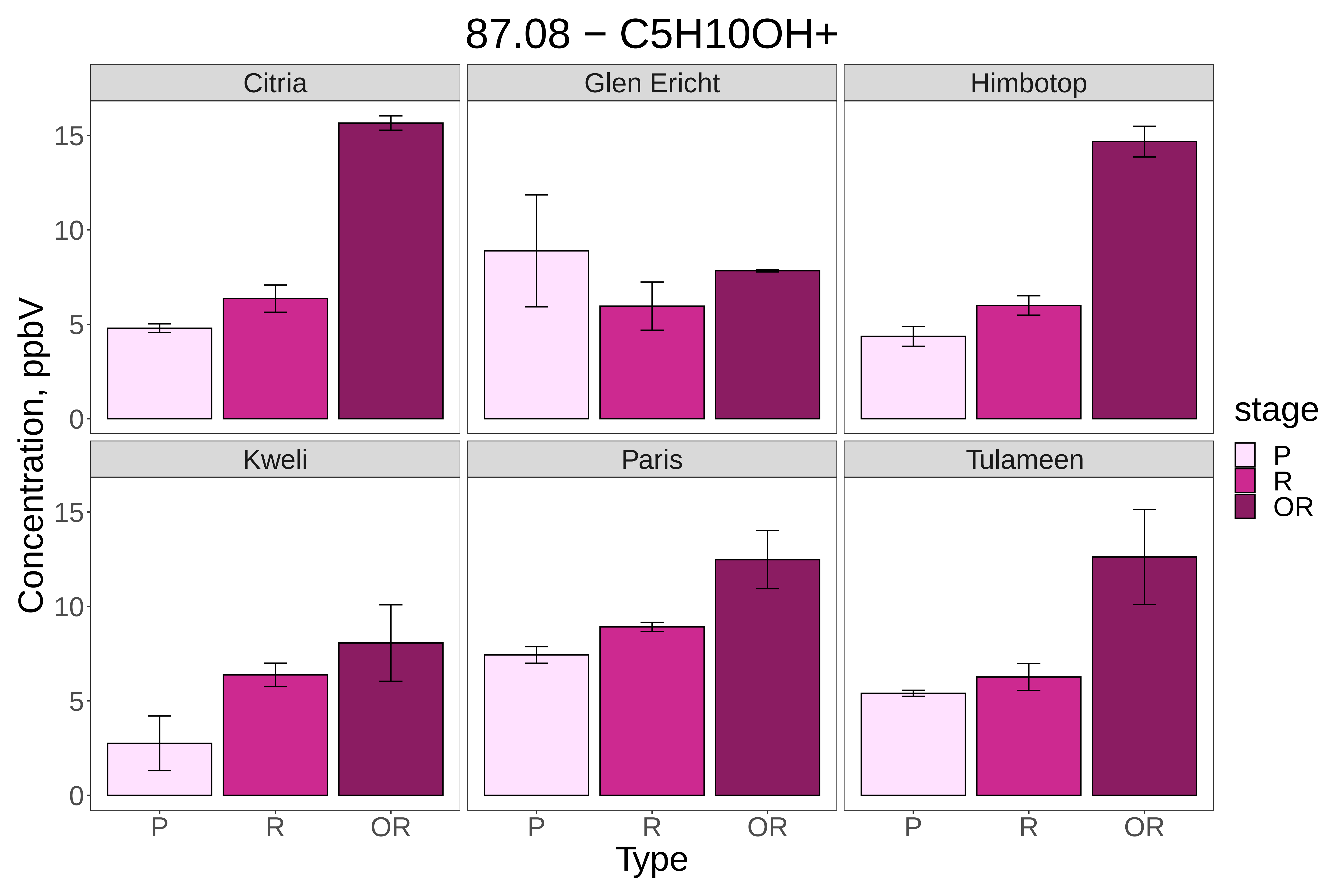

87.08 − C5H10OH+
Citria
Glen Ericht
Himbotop
Kweli
Paris
Tulameen
Concentration, ppbV
0
5
10
15
P
R
OR
Type
stage

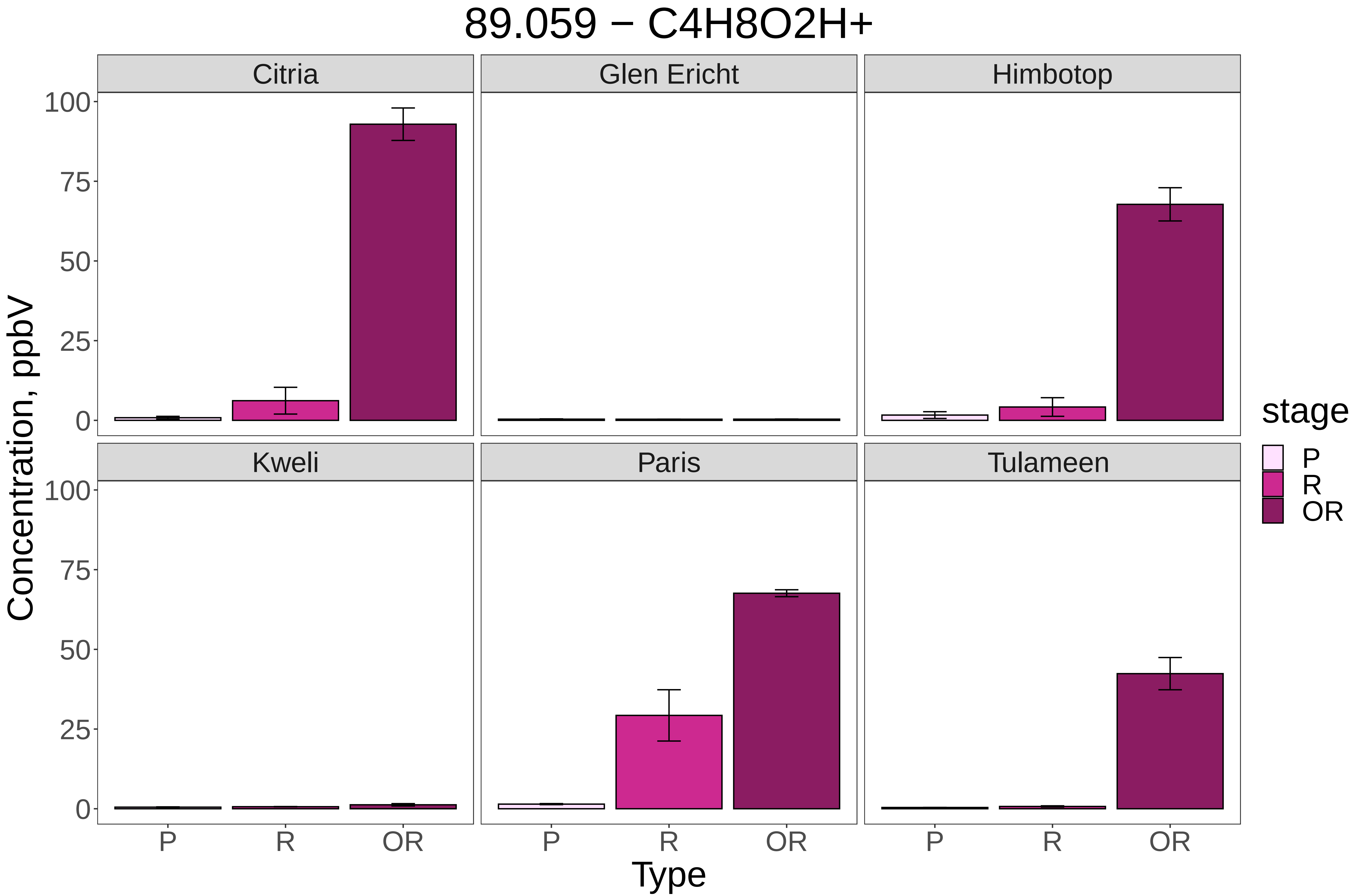
89.059 − C4H8O2H+
Citria
Glen Ericht
Himbotop
Kweli
Paris
Tulameen
Concentration, ppbV
0
25
50
75
100
P
R
OR
Type
stage
P
R
OR

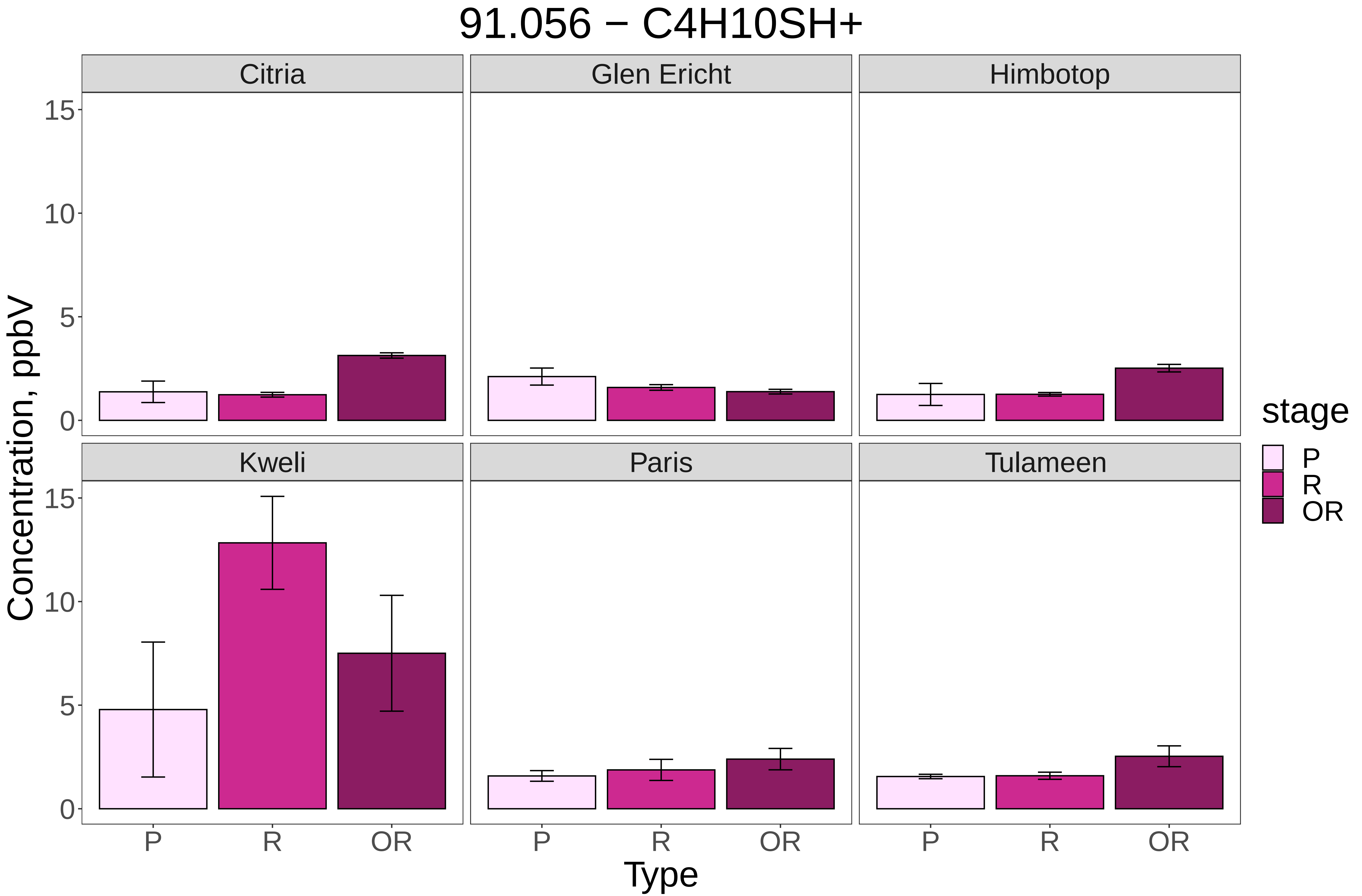

91.056 − C4H10SH+
Citria
Glen Ericht
Himbotop
Kweli
Paris
Tulameen
Concentration, ppbV
0
5
10
15
P
R
OR
Type
stage
P
R
OR

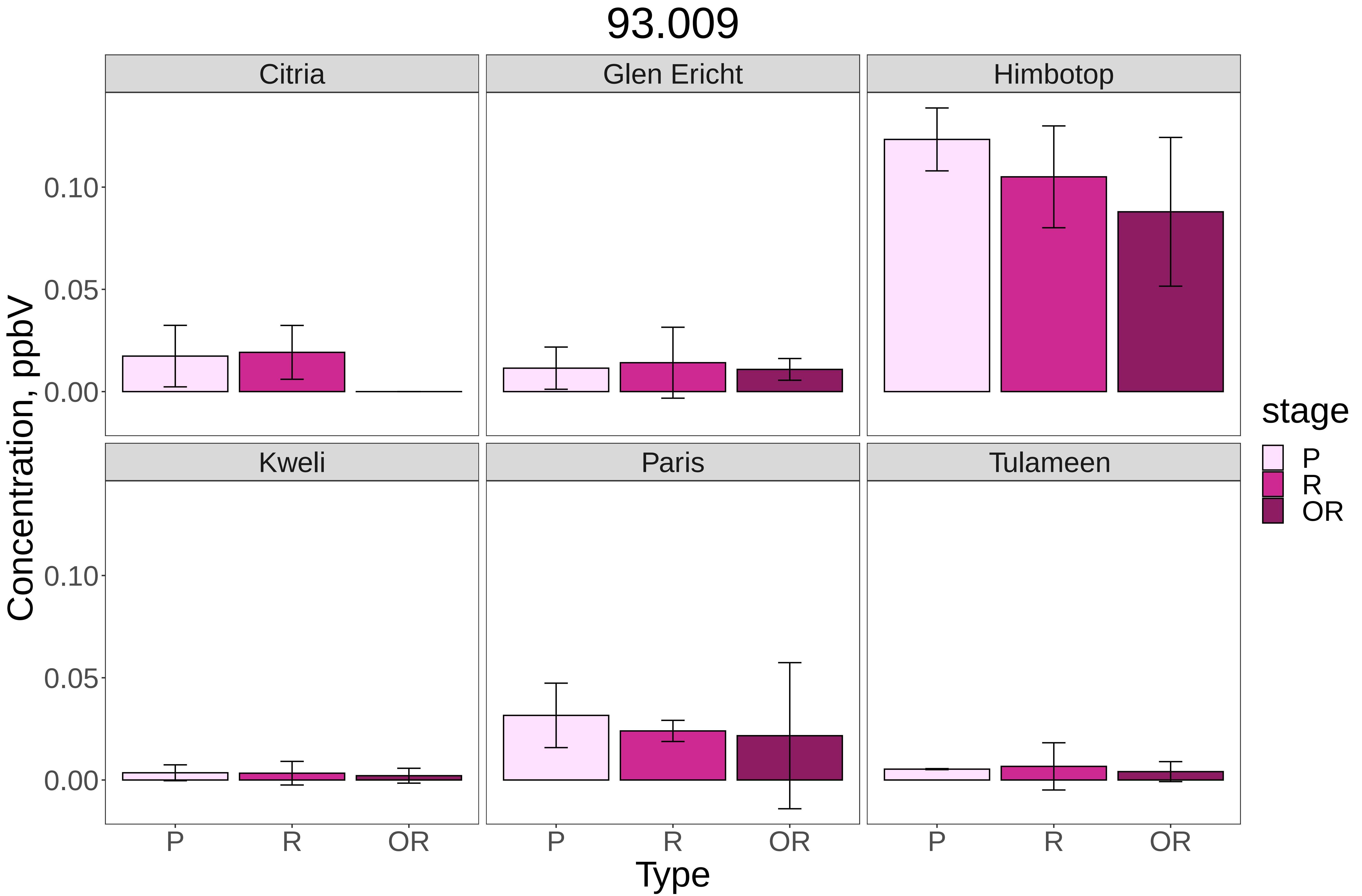

93.009
Citria
Glen Ericht
Himbotop
Kweli
Paris
Tulameen
Concentration, ppbV
0.00
0.05
0.10
P
R
OR
Type
stage

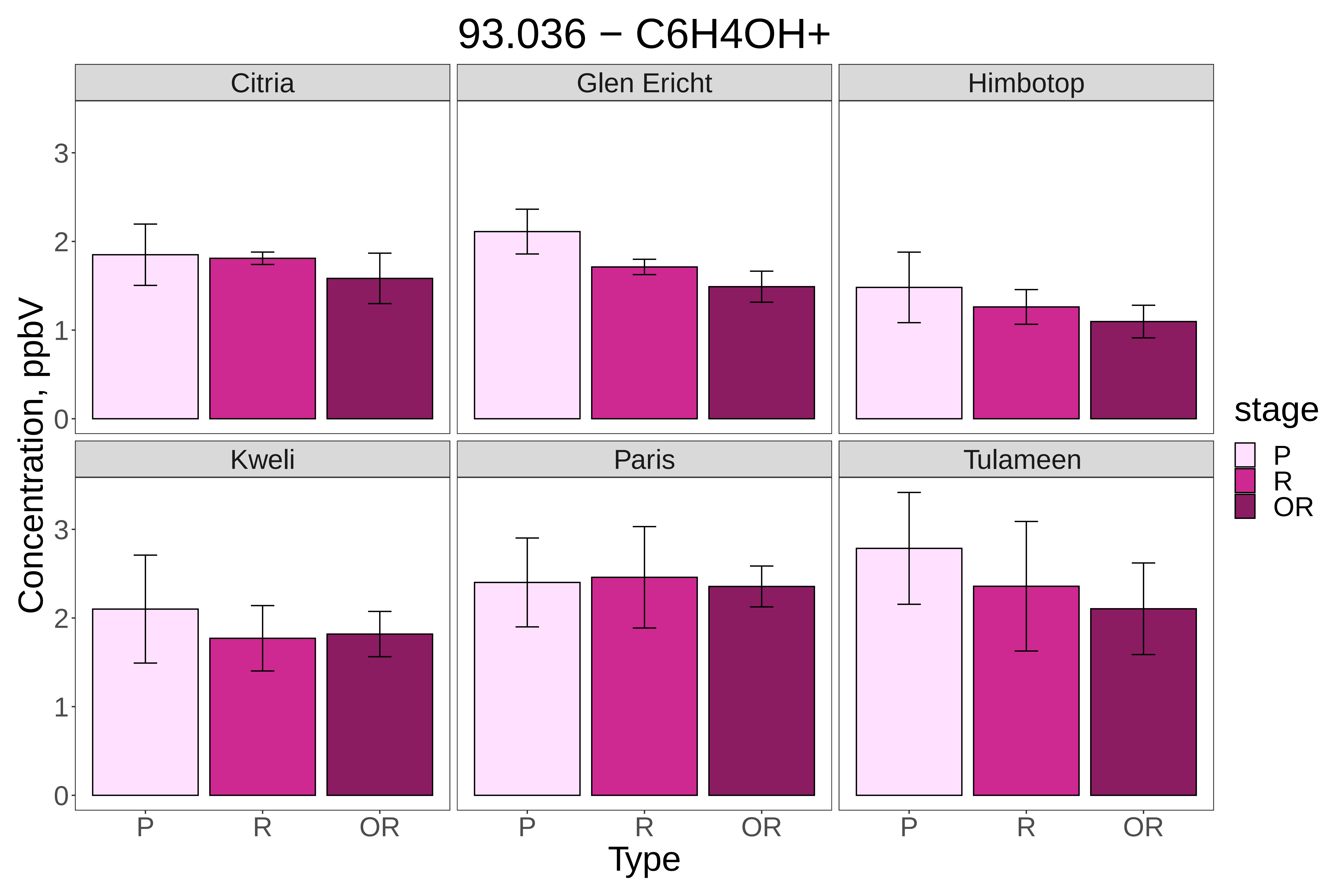

93.036 − C6H4OH+
Citria
Glen Ericht
Himbotop
Kweli
Paris
Tulameen
Concentration, ppbV
0
1
2
3
P
R
OR
Type
stage
P
R
OR

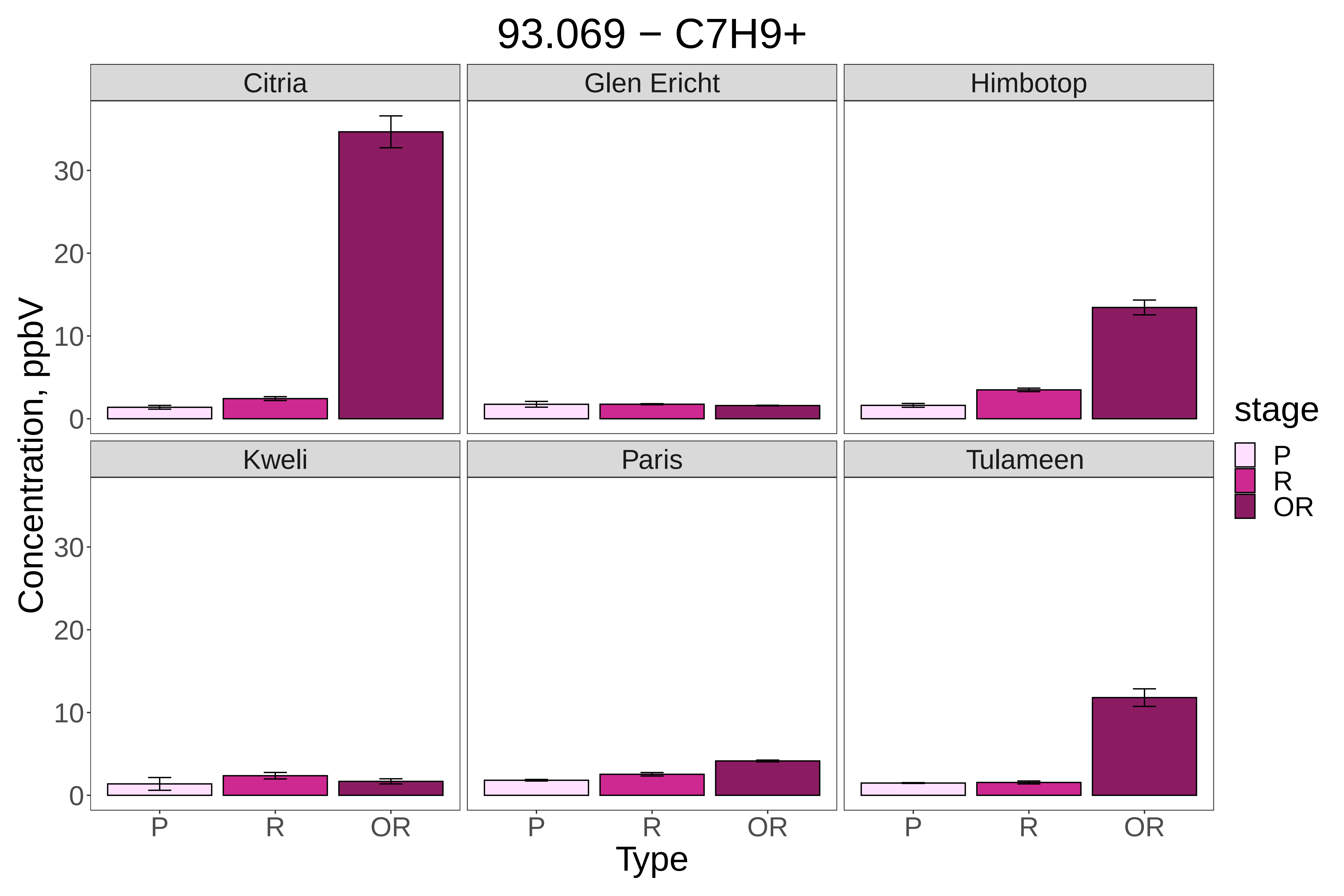

93.069 − C7H9+
Citria
Glen Ericht
Himbotop
Kweli
Paris
Tulameen
Concentration, ppbV
0
10
20
30
P
R
OR
Type
stage
P
R
OR

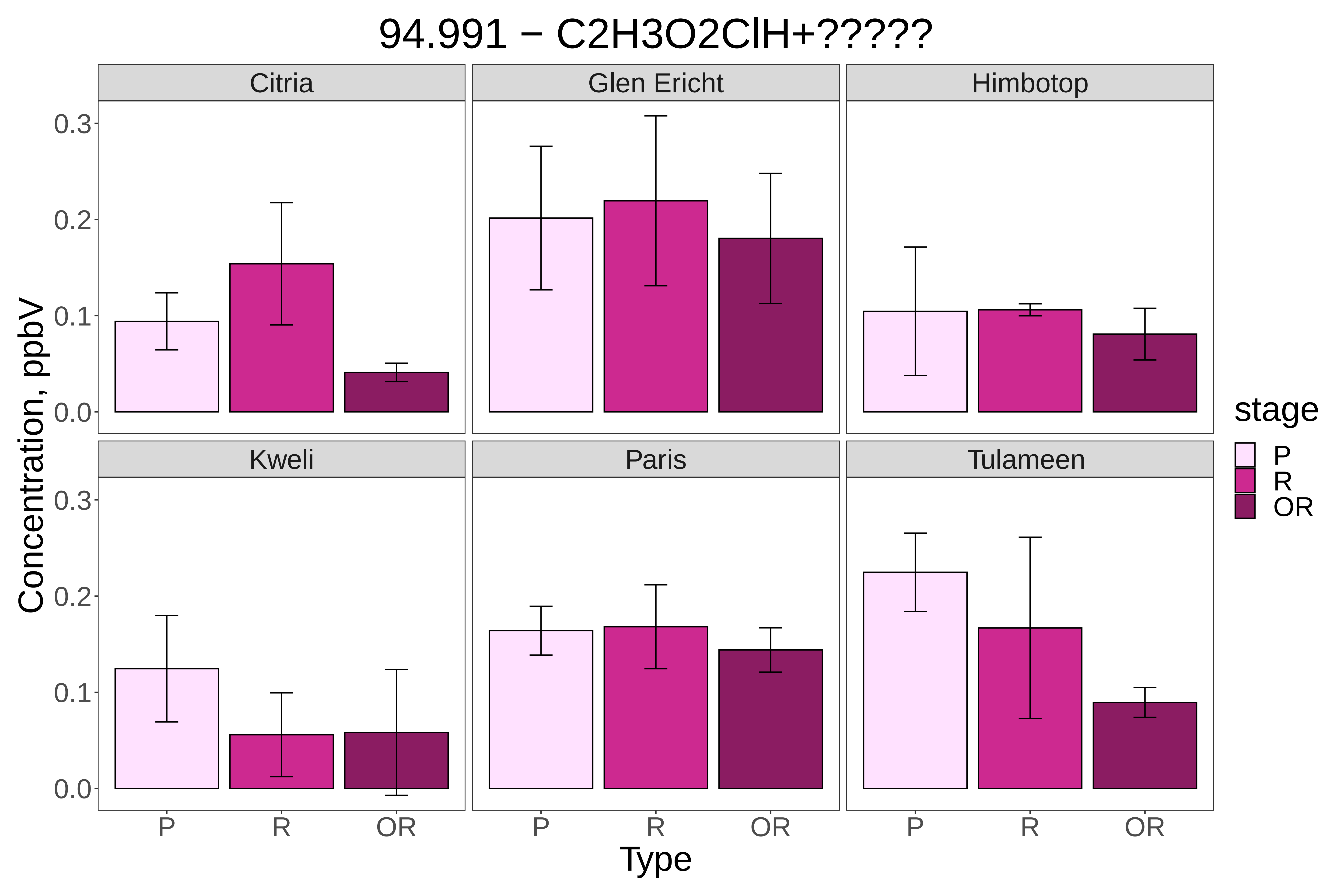

94.991 − C2H3O2ClH+?????
Citria
Glen Ericht
Himbotop
Kweli
Paris
Tulameen
Concentration, ppbV
0.0
0.1
0.2
0.3
P
R
OR
Type
stage
P
R
OR

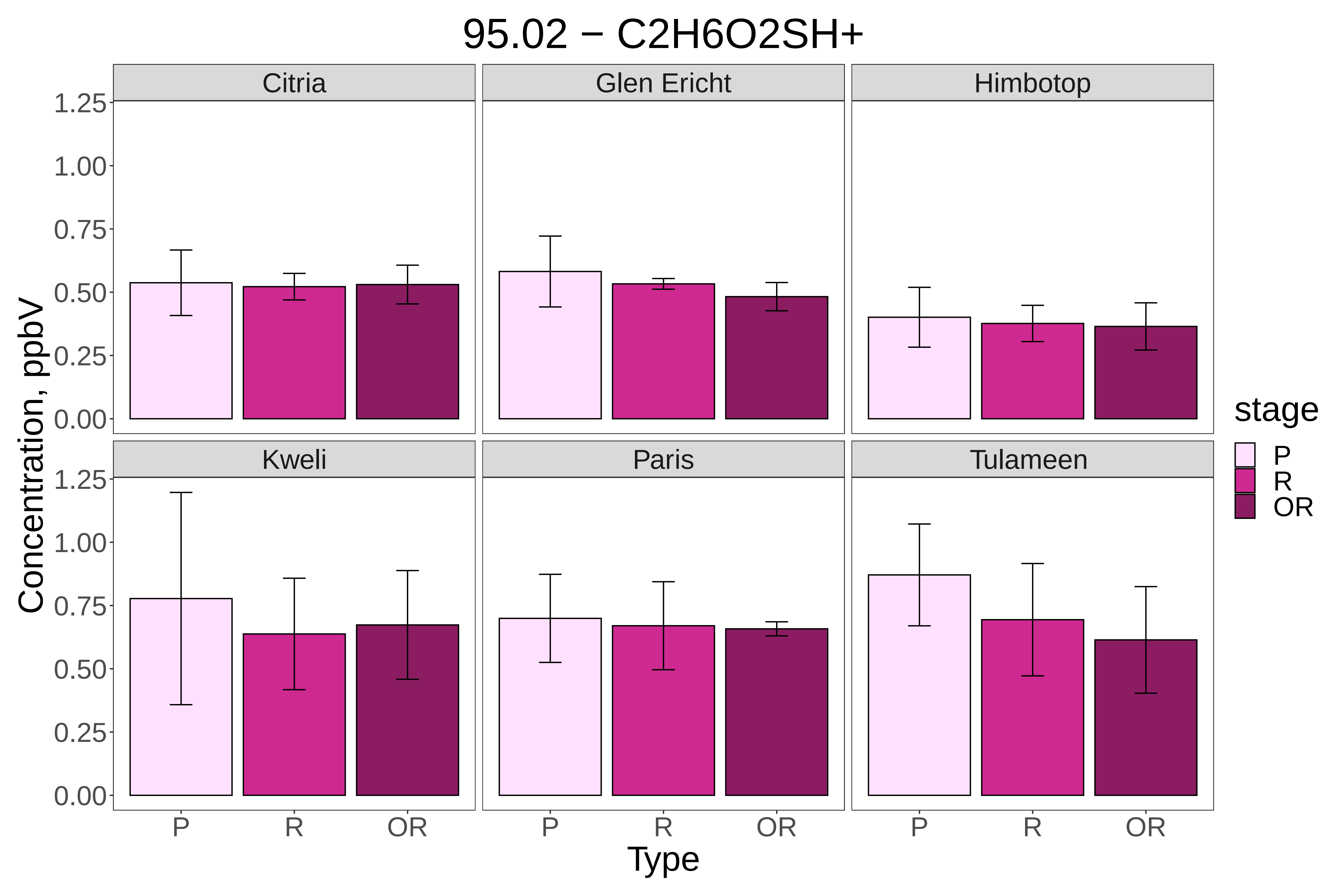
95.02 − C2H6O2SH+
Citria
Glen Ericht
Himbotop
Kweli
Paris
Tulameen
Concentration, ppbV
1.25
1.00
0.75
0.50
0.25
0.00
P
R
OR
Type
stage
P
R
OR

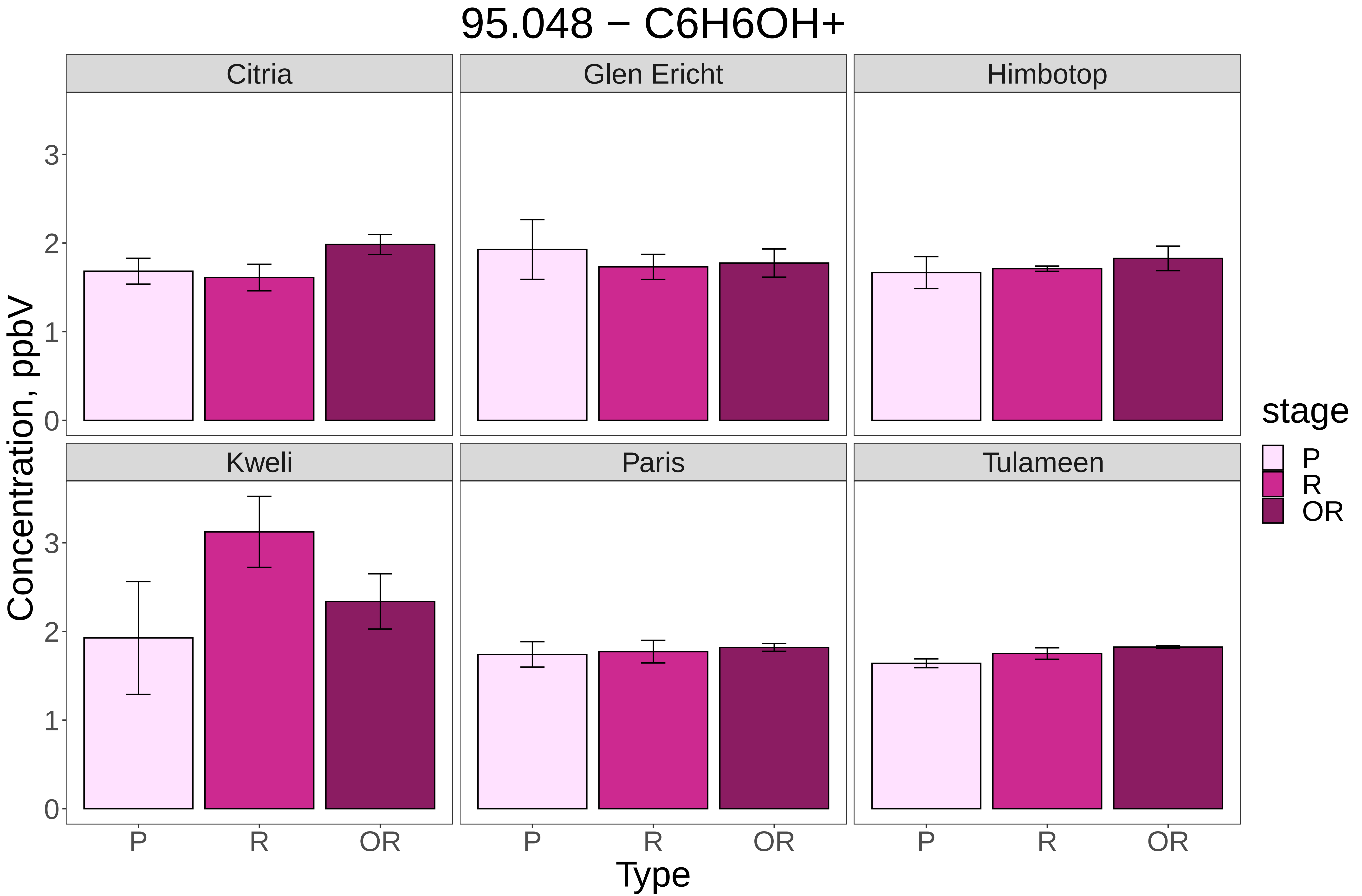

95.048 − C6H6OH+
Citria
Glen Ericht
Himbotop
Kweli
Paris
Tulameen
Concentration, ppbV
0
1
2
3
P
R
OR
Type
stage
P
R
OR

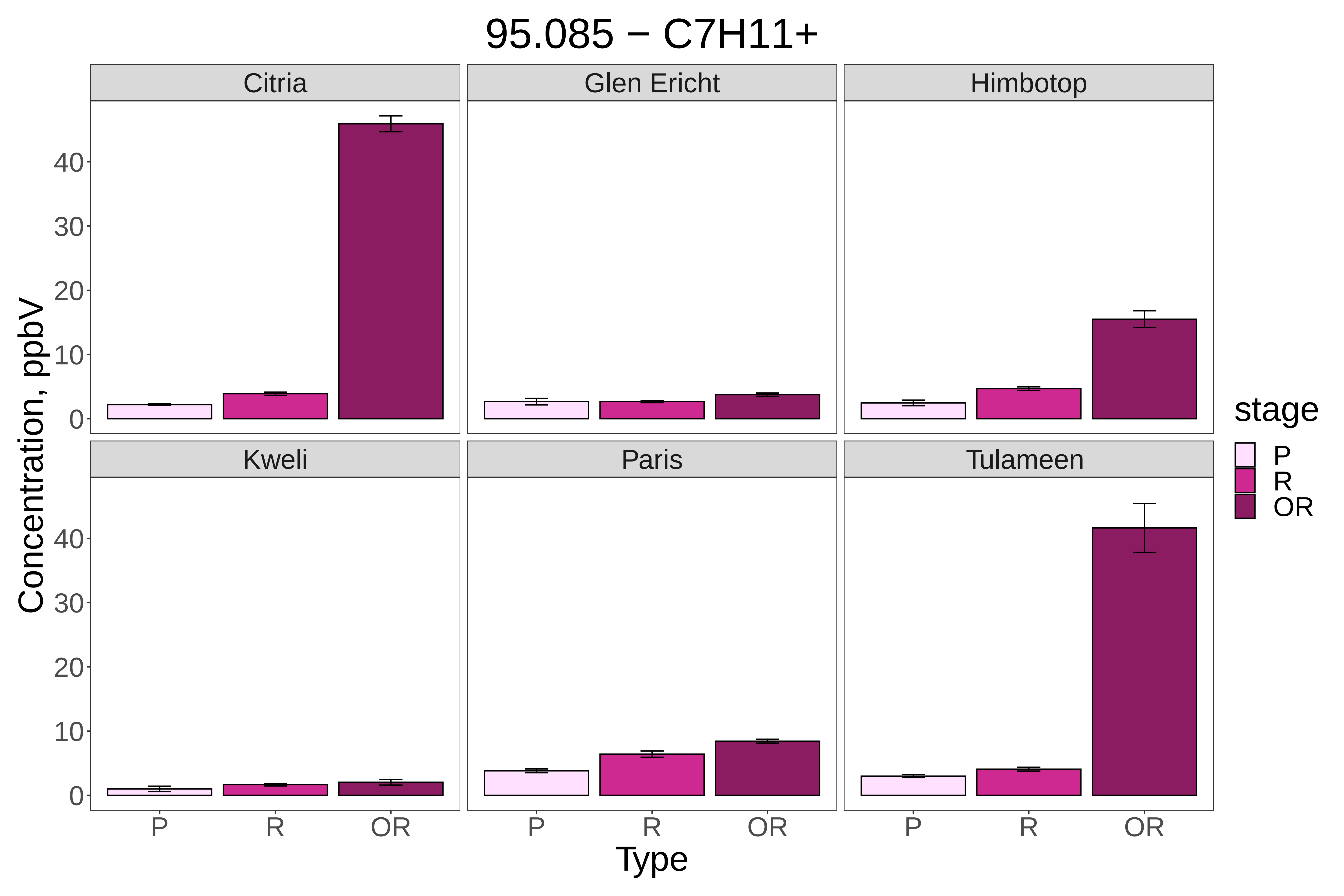

95.085 − C7H11+
Citria
Glen Ericht
Himbotop
Kweli
Paris
Tulameen
Concentration, ppbV
0
10
20
30
40
P
R
OR
Type
stage
P
R
OR

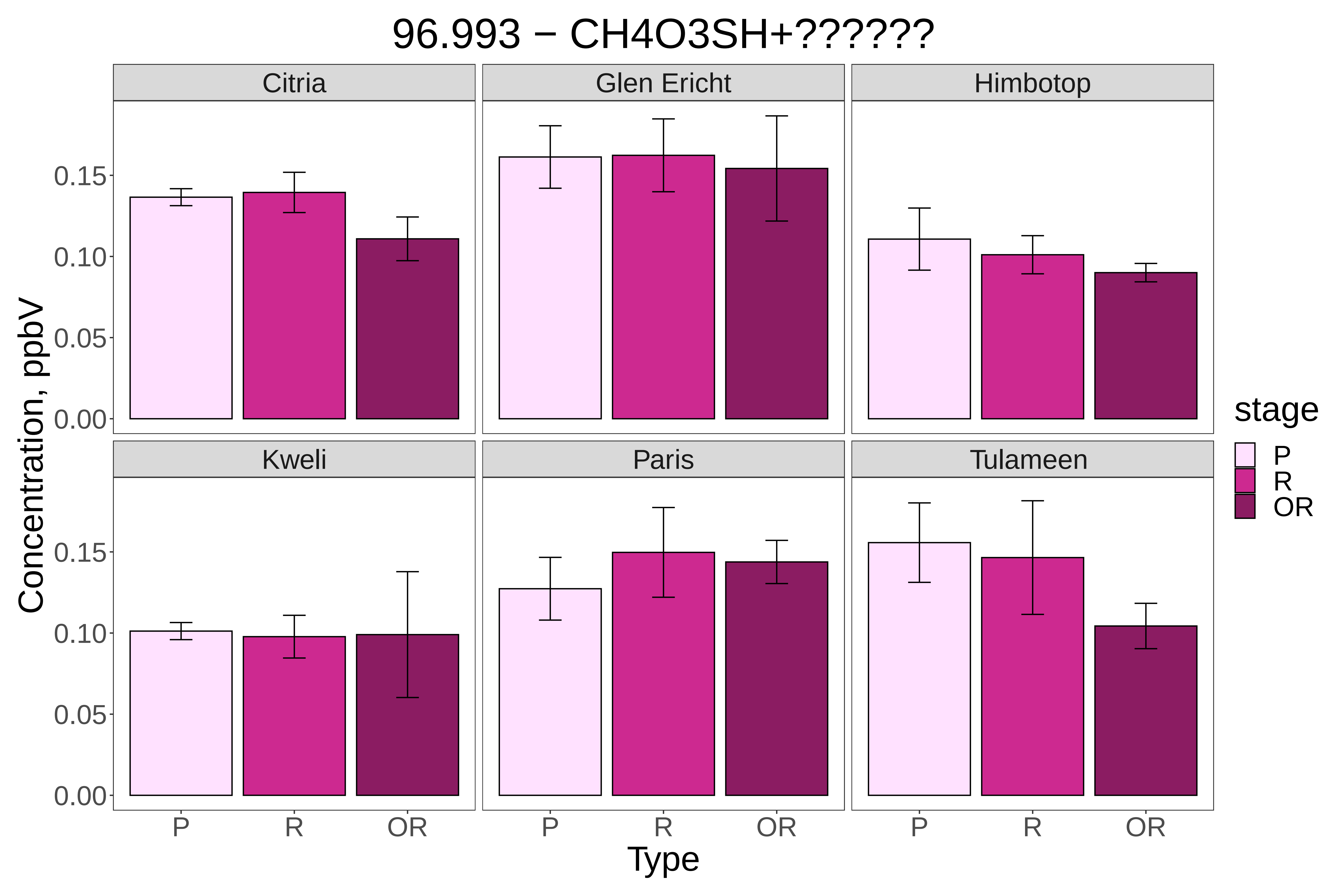

96.993 − CH4O3SH+??????
Citria
Glen Ericht
Himbotop
Kweli
Paris
Tulameen
Concentration, ppbV
0.00
0.05
0.10
0.15
P
R
OR
Type
stage
P
R
OR

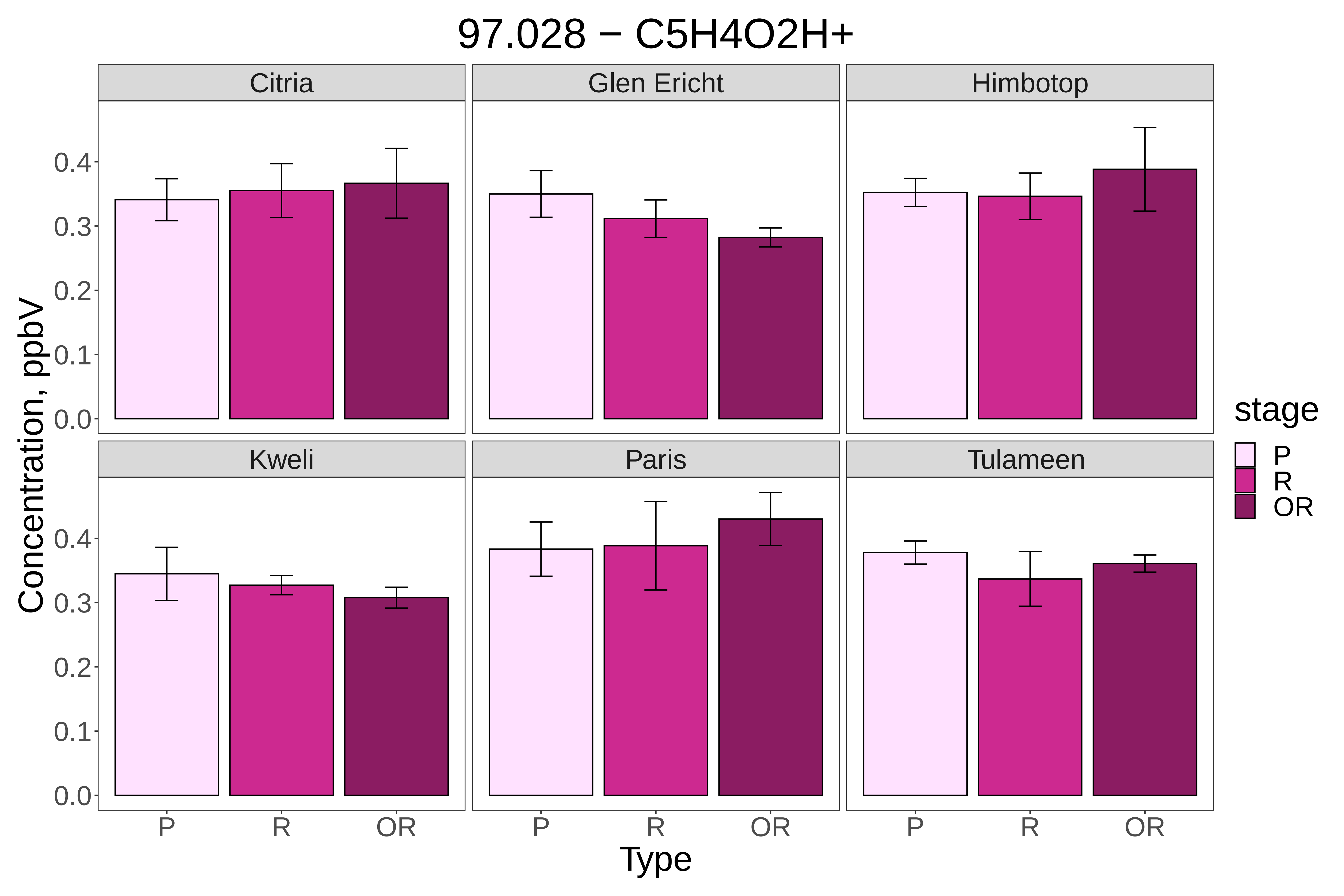

97.028 − C5H4O2H+
Citria
Glen Ericht
Himbotop
Kweli
Paris
Tulameen
Concentration, ppbV
0.0
0.1
0.2
0.3
0.4
P
R
OR
Type
stage

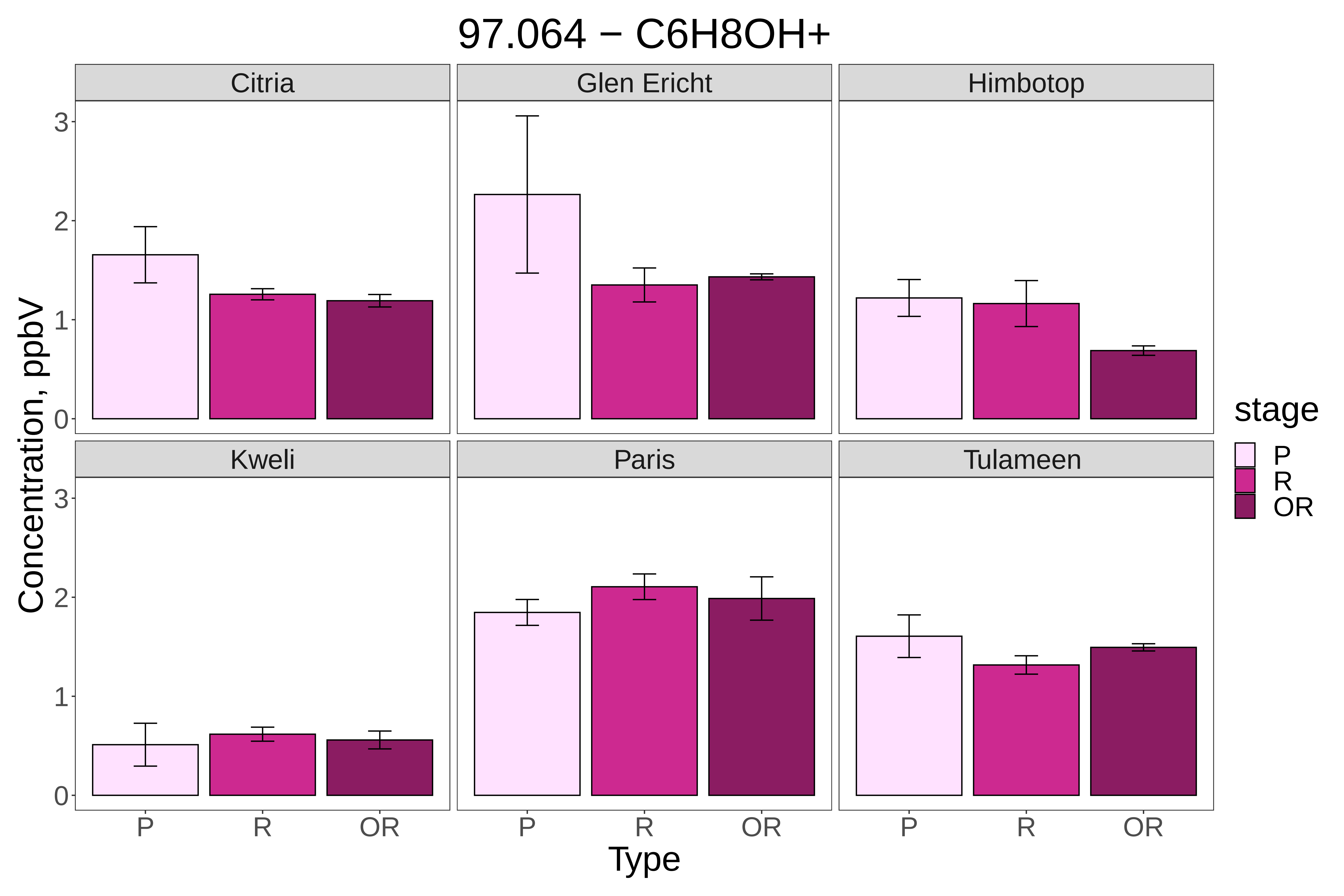

97.064 − C6H8OH+
Citria
Glen Ericht
Himbotop
Kweli
Paris
Tulameen
Concentration, ppbV
0
1
2
3
P
R
OR
Type
stage
P
R
OR

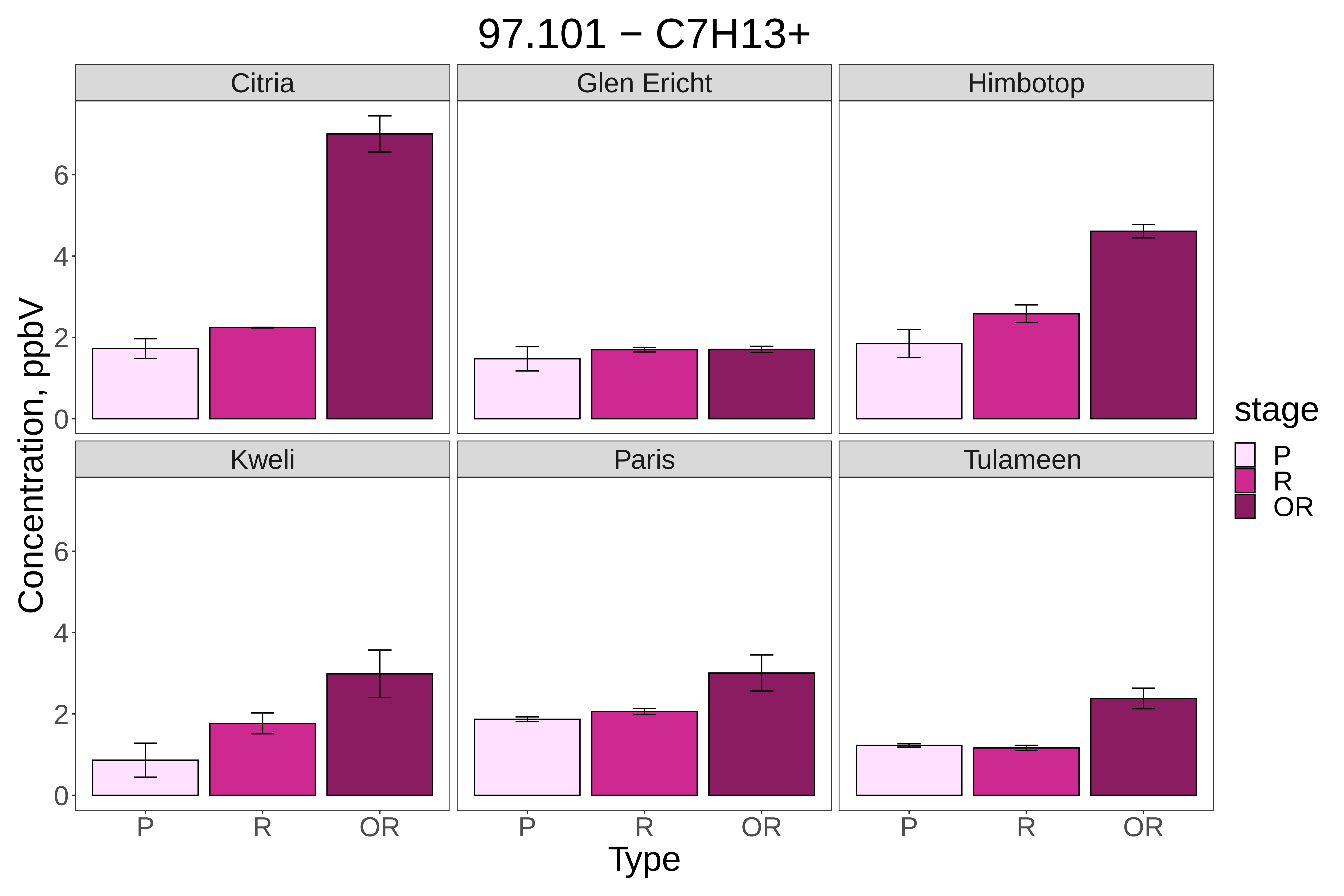

97.101 − C7H13+
Citria
Glen Ericht
Himbotop
Kweli
Paris
Tulameen
Concentration, ppbV
0
2
4
6
P
R
OR
Type
stage
P
R
OR

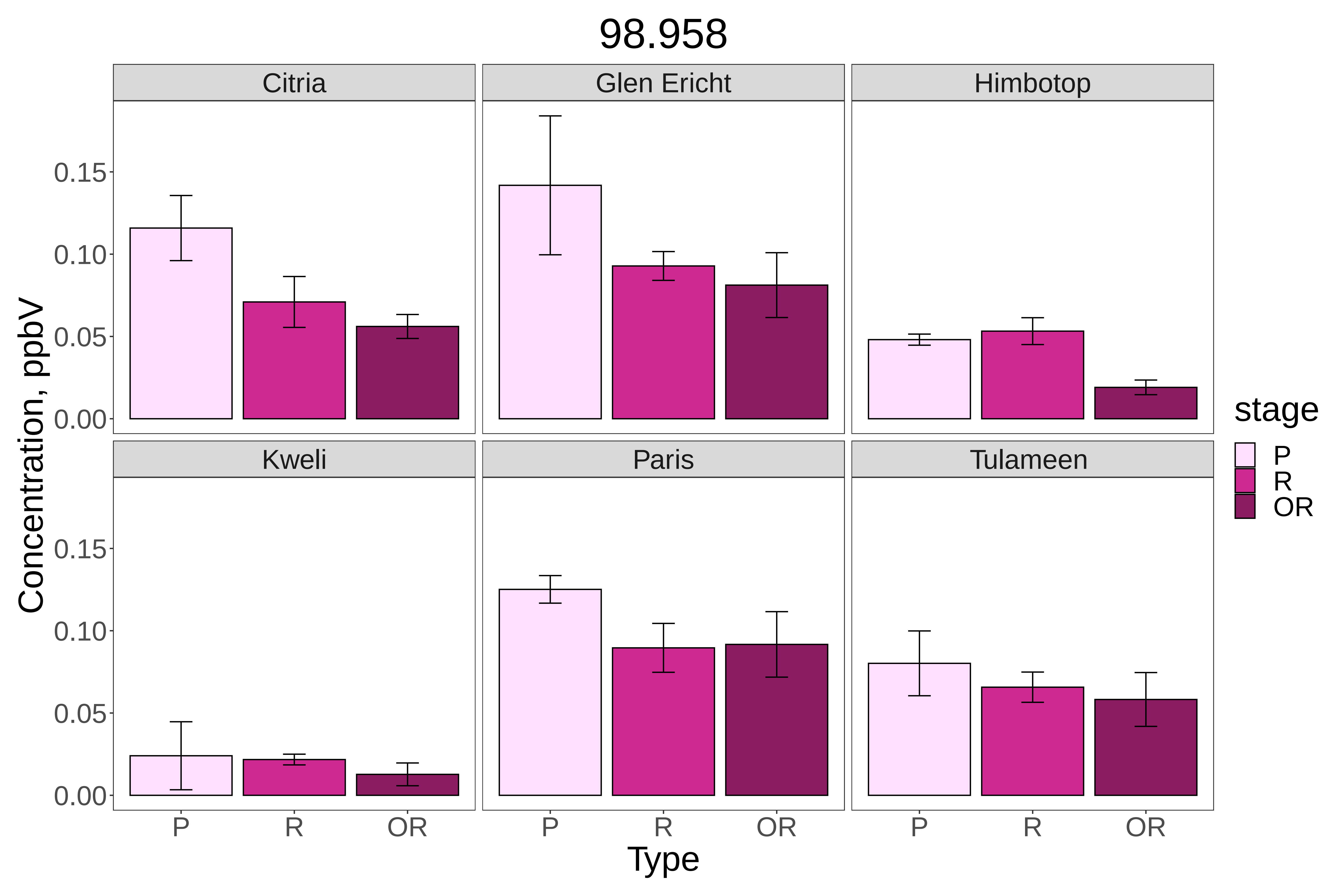

98.958
Citria
Glen Ericht
Himbotop
Kweli
Paris
Tulameen
Concentration, ppbV
0.00
0.05
0.10
0.15
Type
P
R
OR
stage
P
R
OR

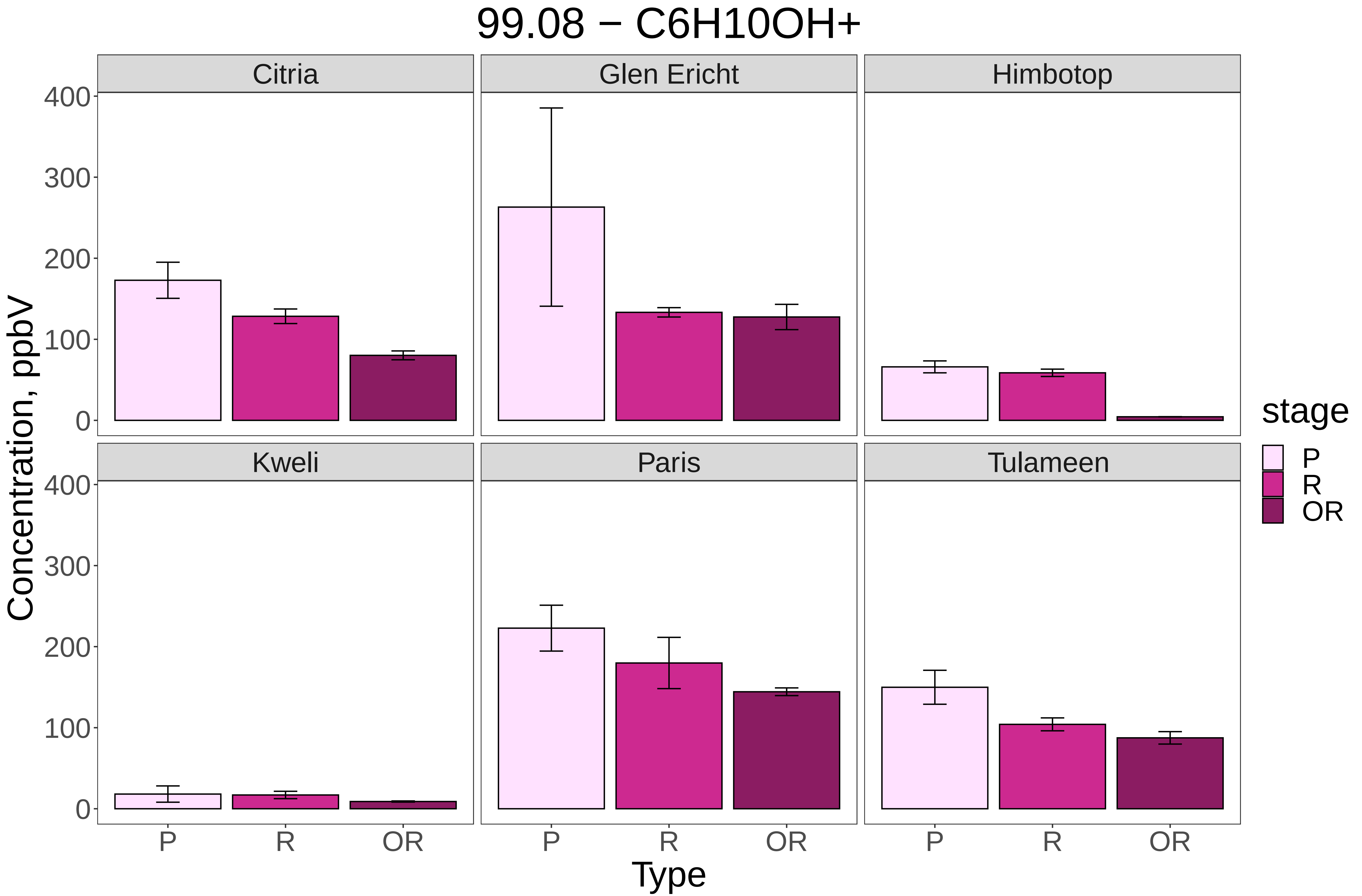
99.08 − C6H10OH+
Citria
Glen Ericht
Himbotop
Kweli
Paris
Tulameen
Concentration, ppbV
0
100
200
300
400
P
R
OR
Type
stage
P
R
OR

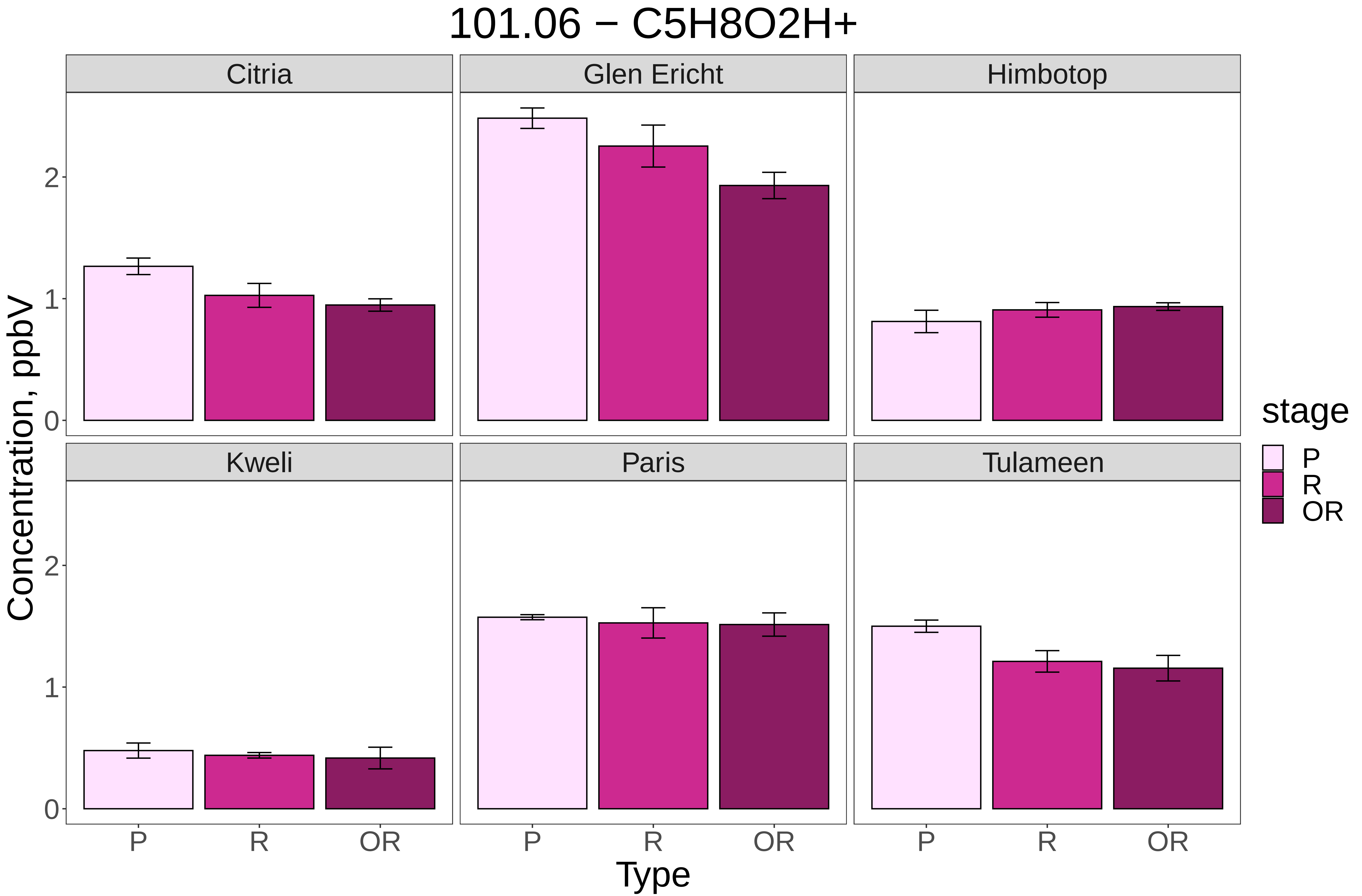

101.06 − C5H8O2H+
Citria
Glen Ericht
Himbotop
Kweli
Paris
Tulameen
Concentration, ppbV
0
1
2
P
R
OR
Type
stage
P
R
OR

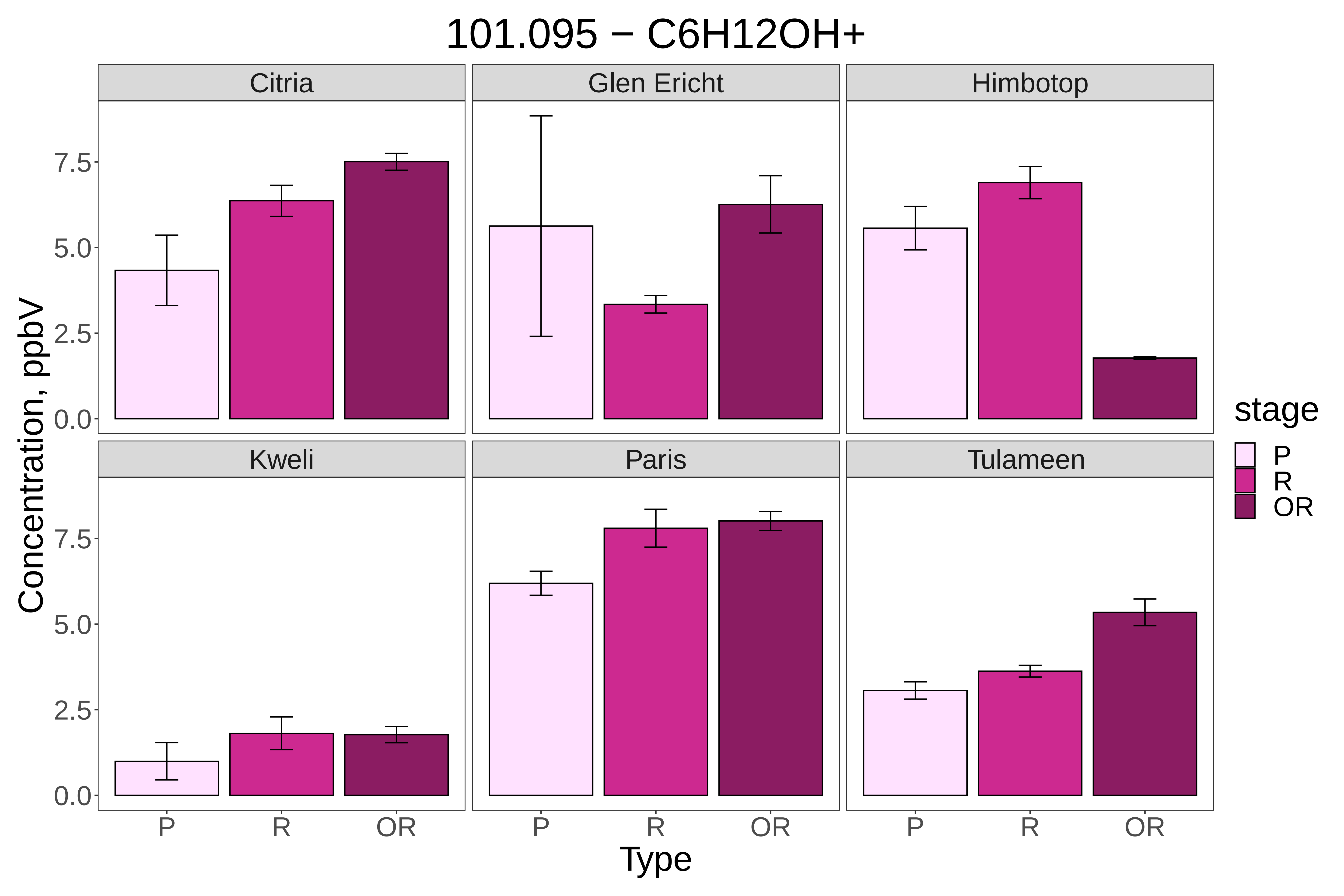

101.095 − C6H12OH+
Citria
Glen Ericht
Himbotop
Kweli
Paris
Tulameen
Concentration, ppbV
0.0
2.5
5.0
7.5
P
R
OR
Type
stage

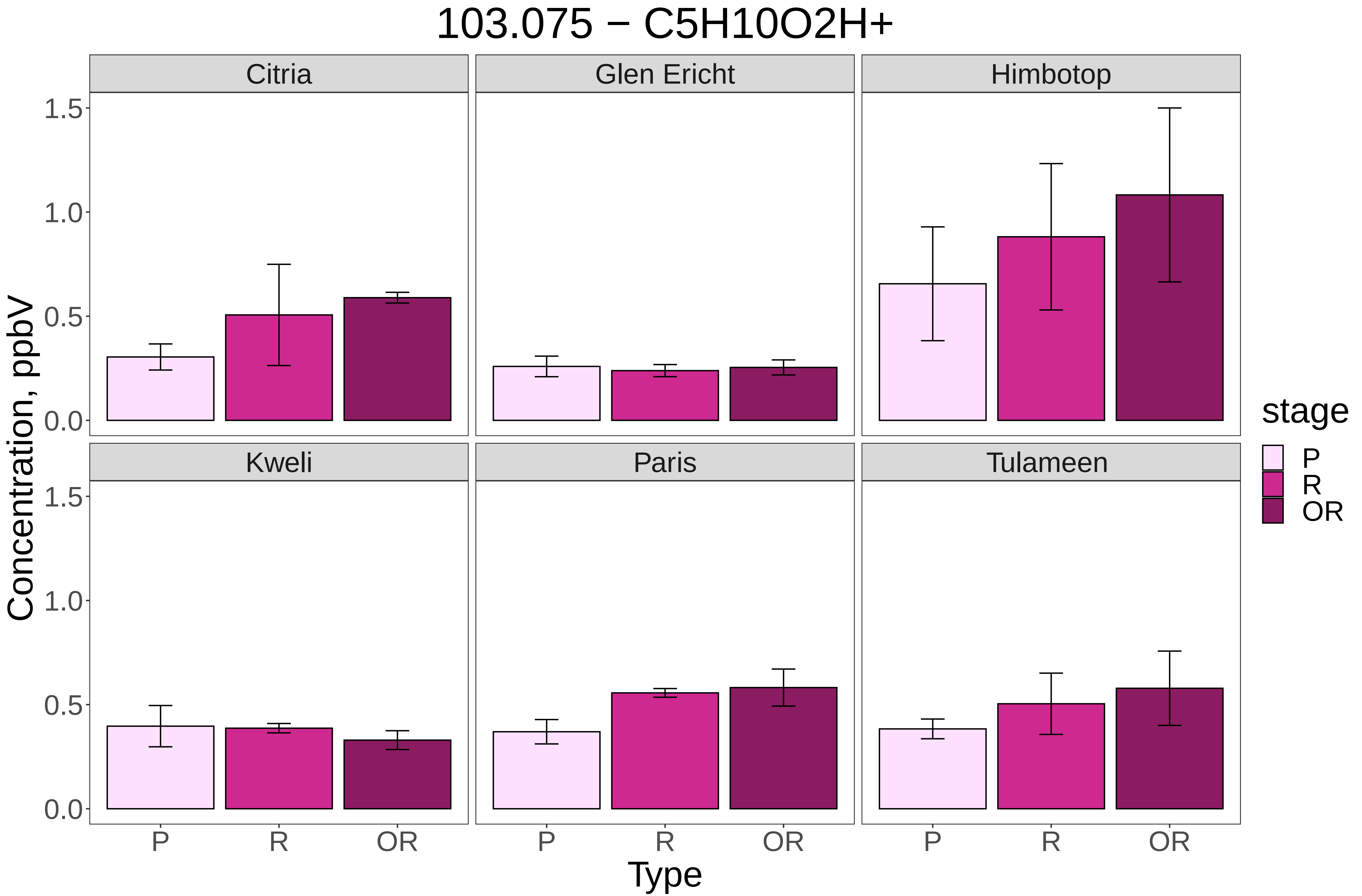
103.075 − C5H10O2H+
Citria
Glen Ericht
Himbotop
Kweli
Paris
Tulameen
Concentration, ppbV
0.0
0.5
1.0
1.5
P
R
OR
Type
stage

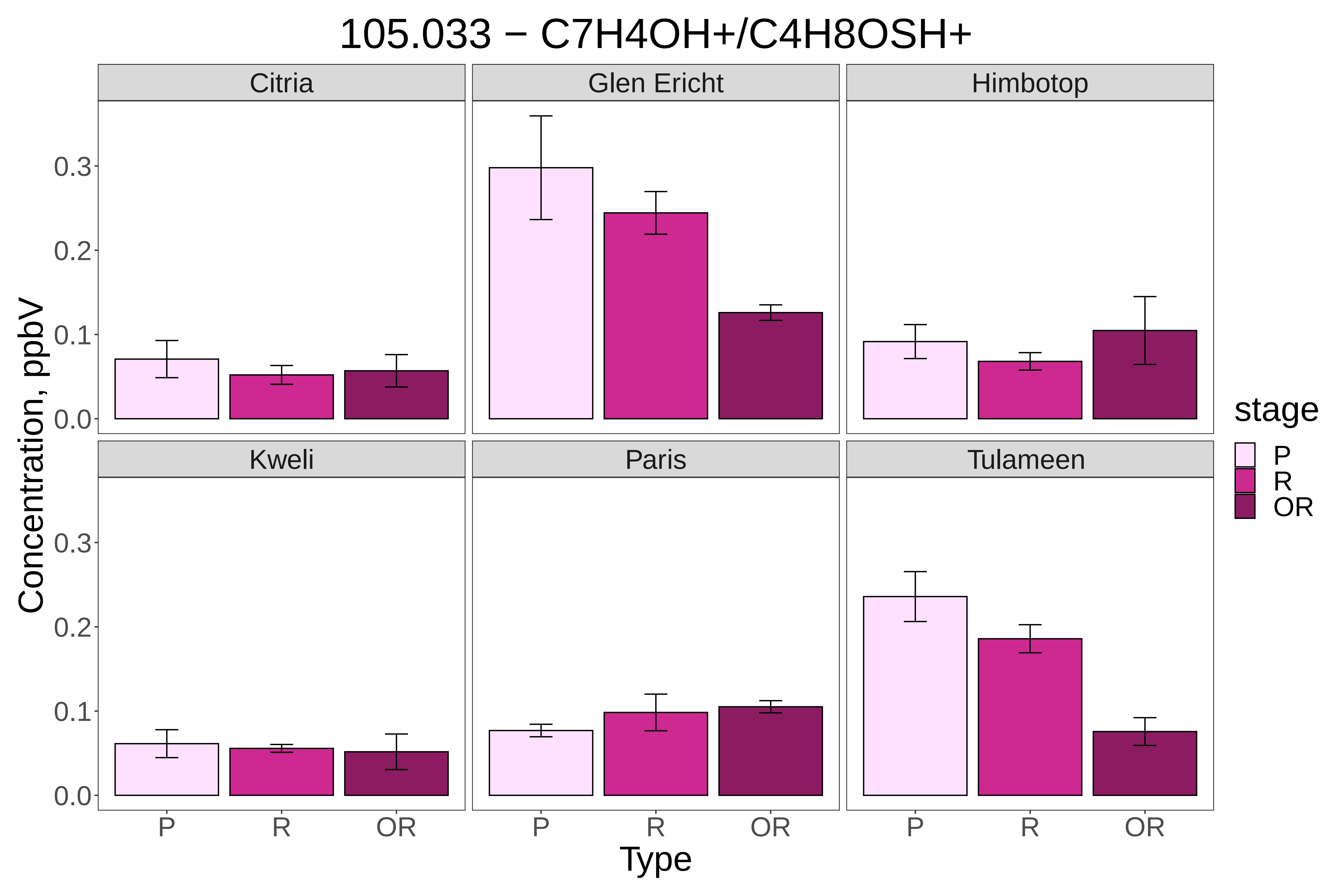

105.033 − C7H4OH+/C4H8OSH+
Citria
Glen Ericht
Himbotop
Kweli
Paris
Tulameen
Concentration, ppbV
0.0
0.1
0.2
0.3
P
R
OR
Type
stage
P
R
OR

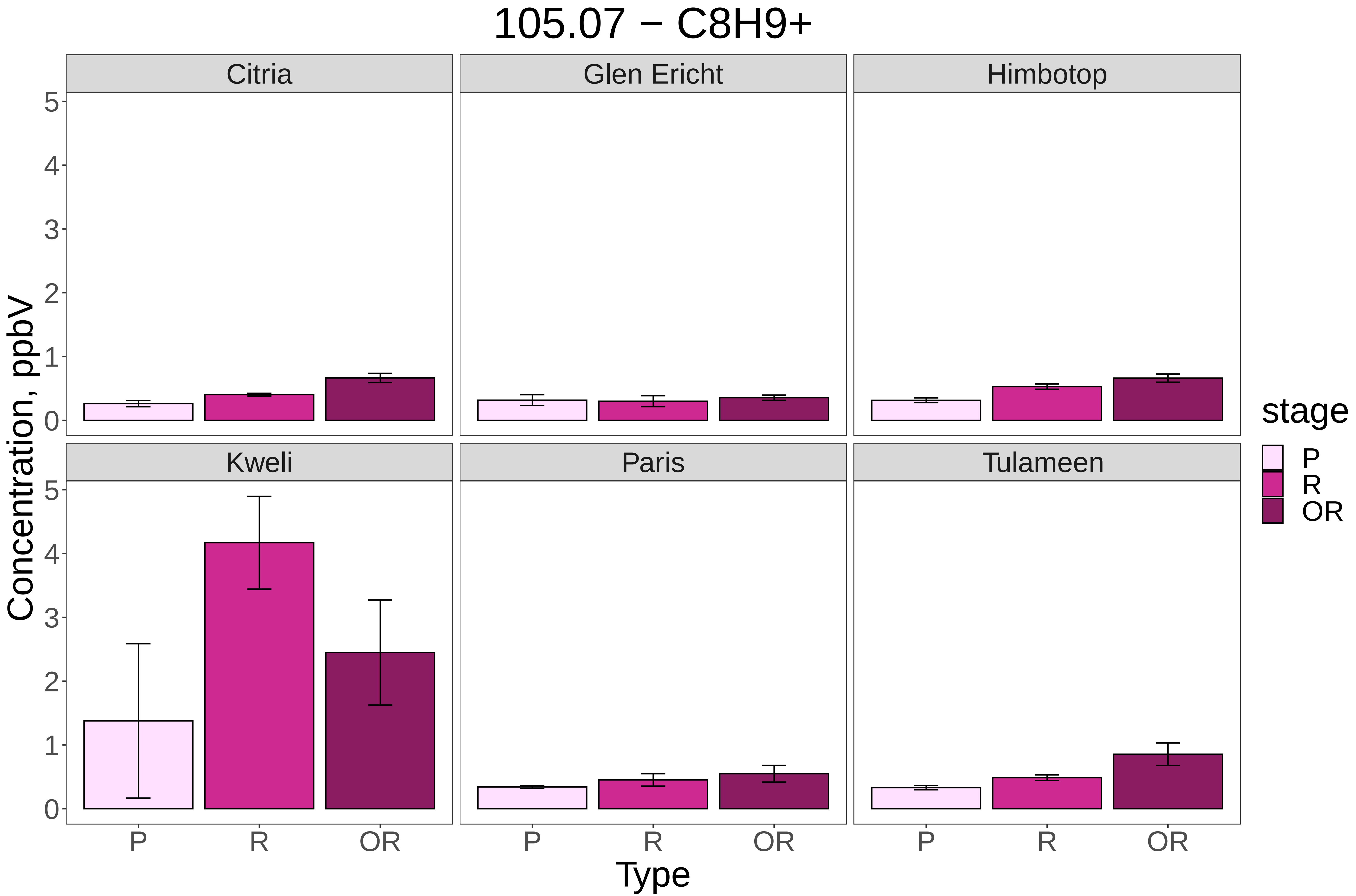

105.07 − C8H9+
Citria
Glen Ericht
Himbotop
Kweli
Paris
Tulameen
Concentration, ppbV
Type
P
R
OR
stage
P
R
OR
0
1
2
3
4
5

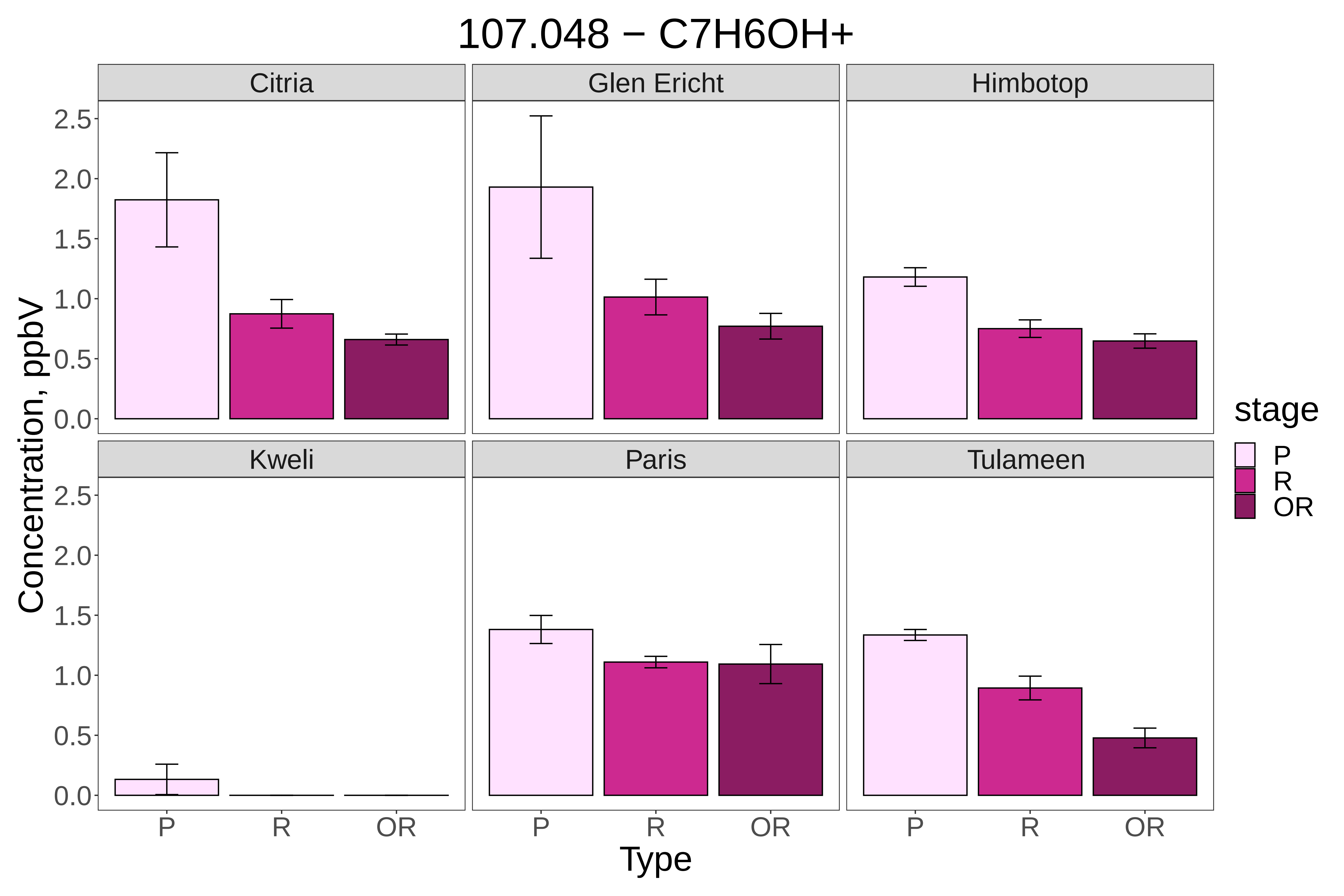

107.048 − C7H6OH+
Citria
Glen Ericht
Himbotop
Kweli
Paris
Tulameen
Concentration, ppbV
0.0
0.5
1.0
1.5
2.0
2.5
P
R
OR
Type
stage
P
R
OR

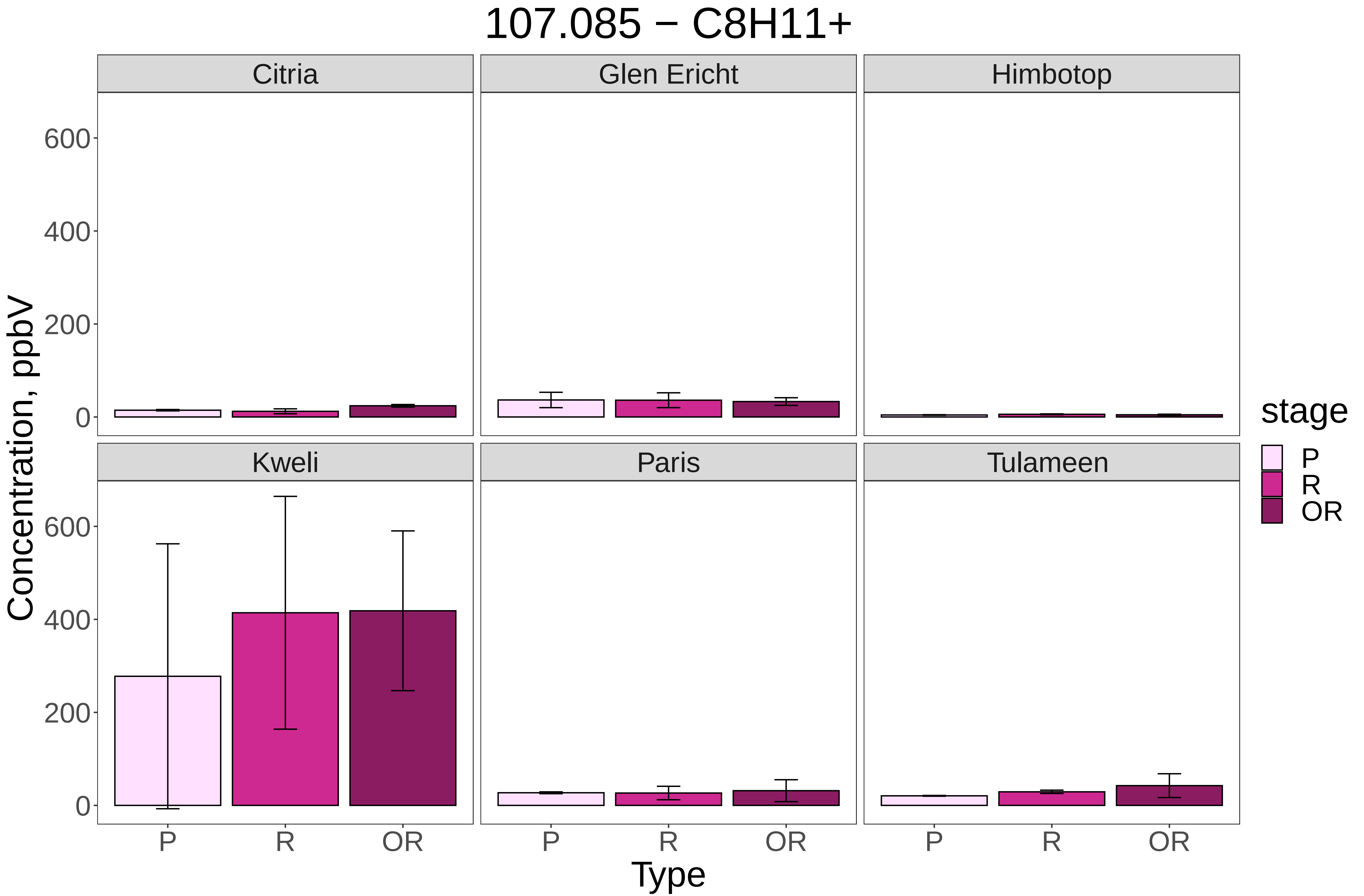
107.085 − C8H11+
Citria
Glen Ericht
Himbotop
Kweli
Paris
Tulameen
Concentration, ppbV
0
200
400
600
P
R
OR
Type
stage
P
R
OR

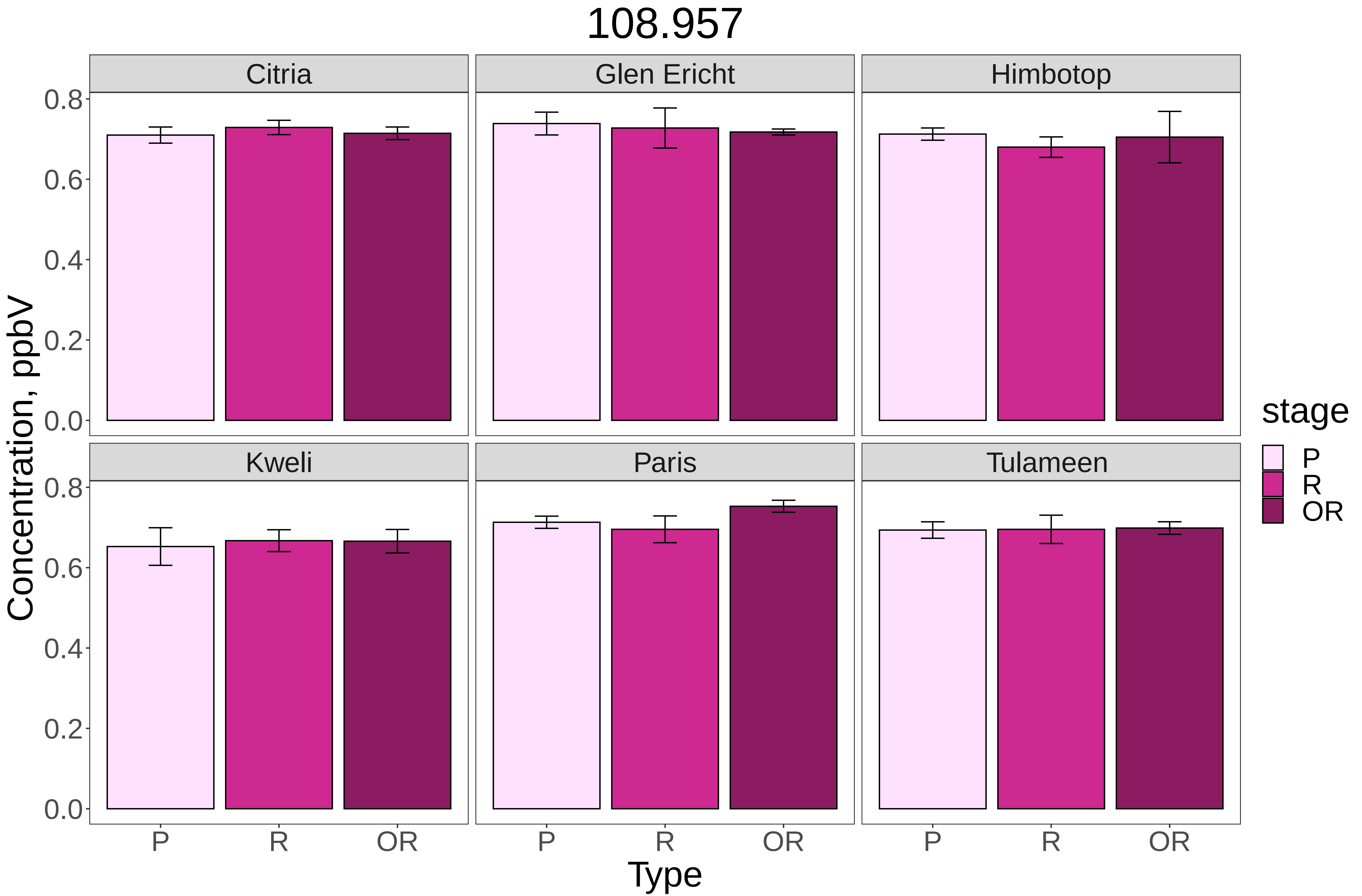

108.957
Citria
Glen Ericht
Himbotop
Kweli
Paris
Tulameen
Concentration, ppbV
0.0
0.2
0.4
0.6
0.8
P
R
OR
Type
stage
P
R
OR

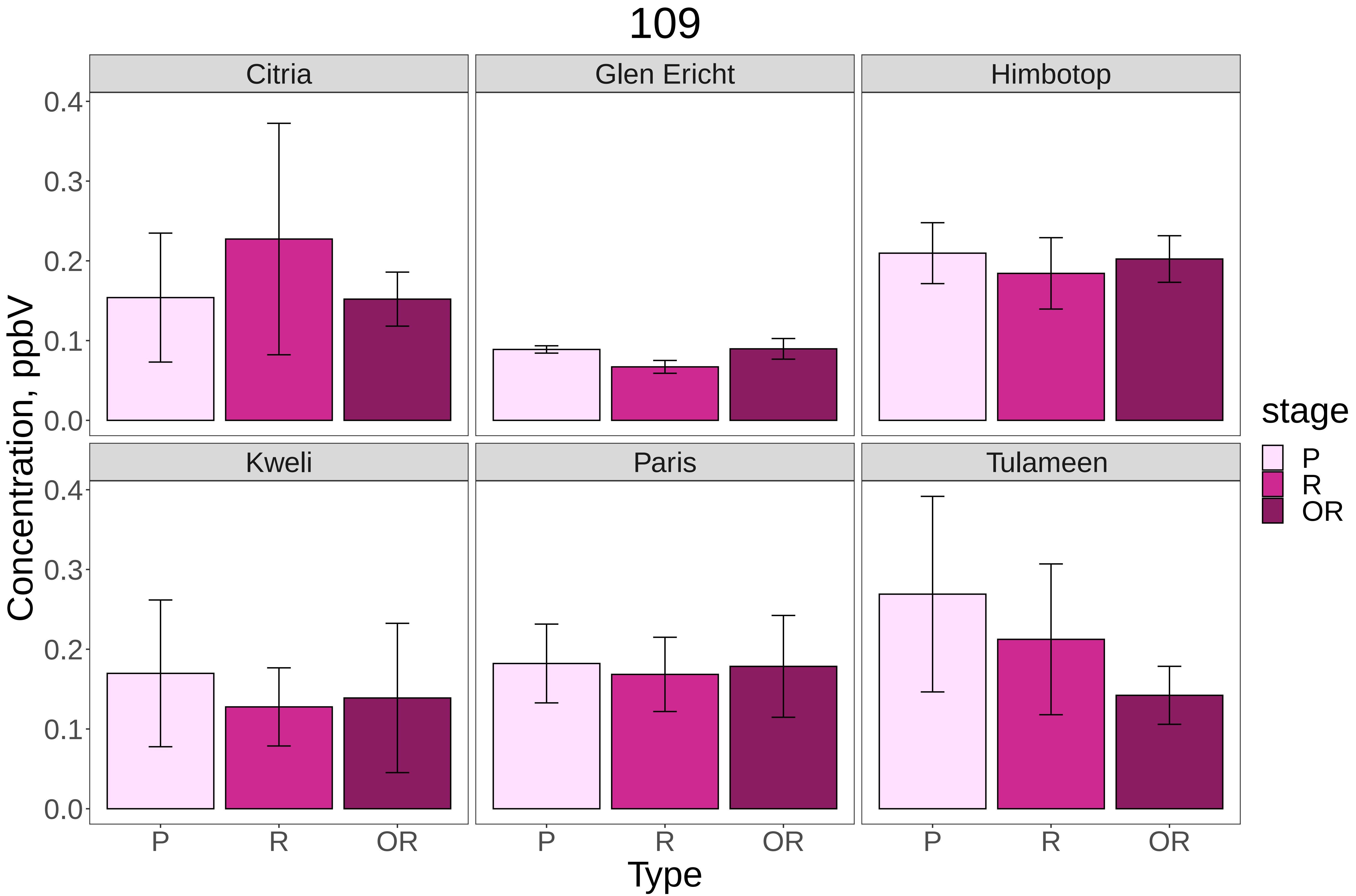
109
Citria
Glen Ericht
Himbotop
Kweli
Paris
Tulameen
0.0
0.1
0.2
0.3
0.4
Concentration, ppbV
P
R
OR
Type
stage
P
R
OR

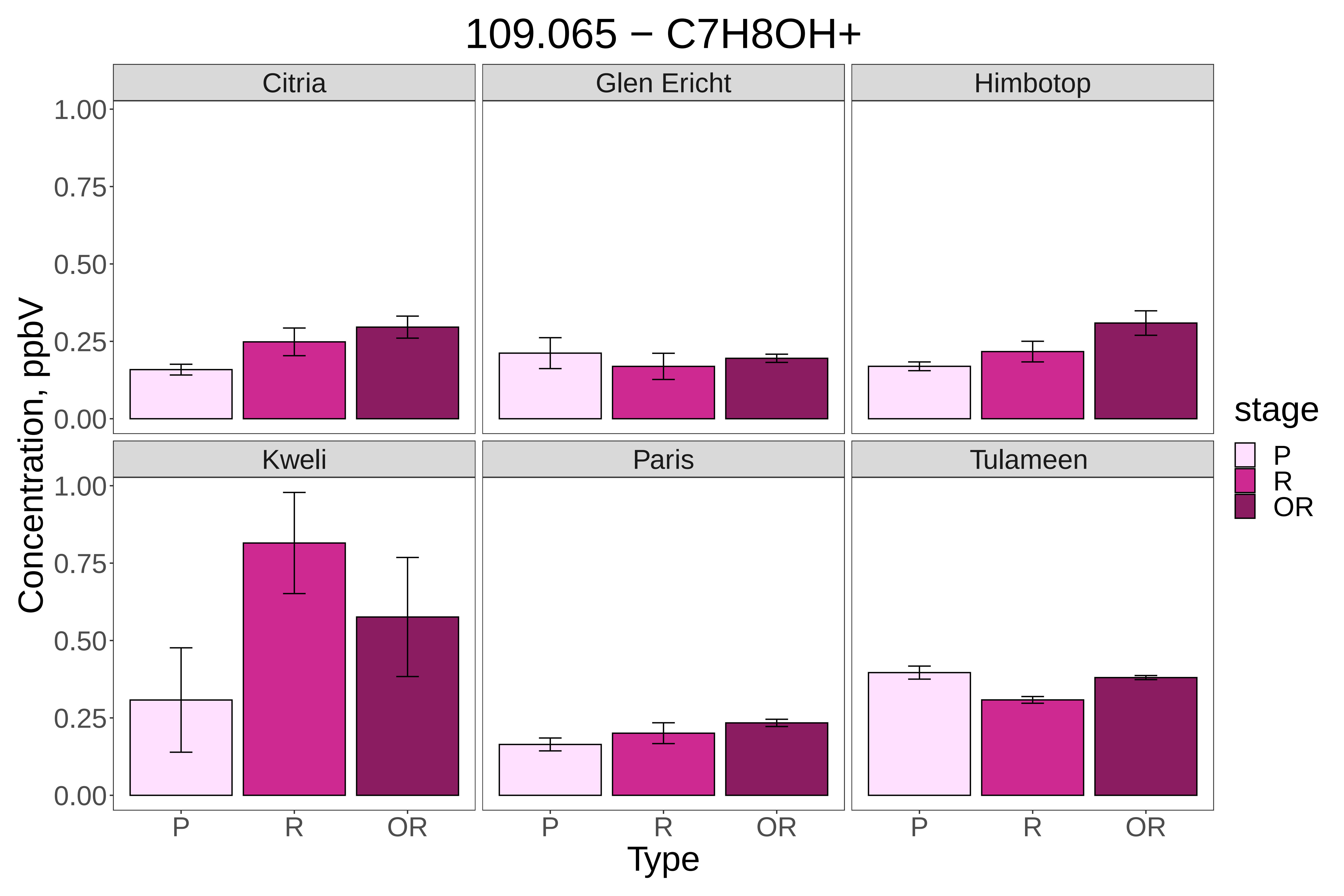
109.065 − C7H8OH+
Citria
Glen Ericht
Himbotop
Kweli
Paris
Tulameen
1.00
0.75
0.50
0.25
0.00
Concentration, ppbV
P
R
OR
Type
stage
P
R
OR

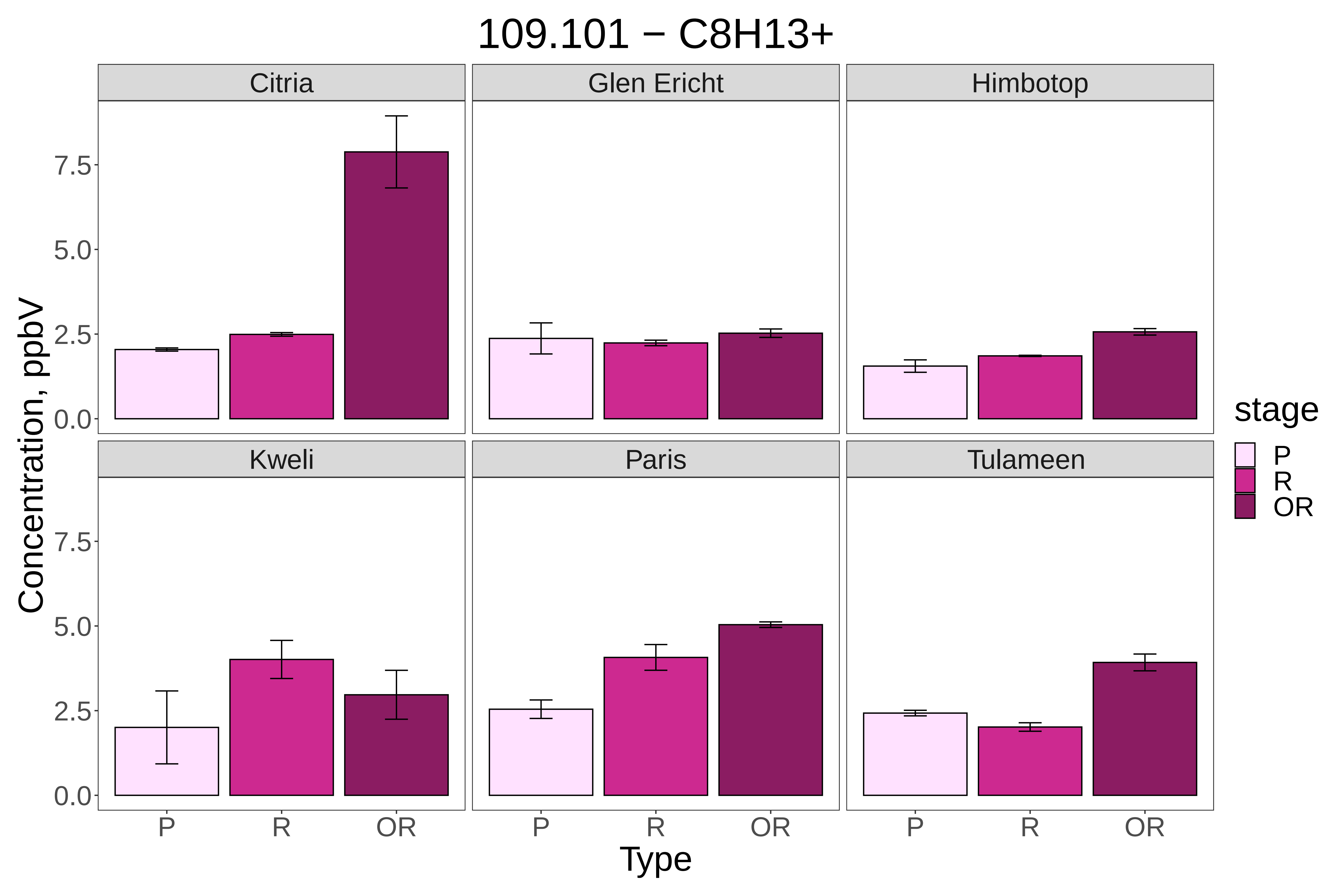
109.101 − C8H13+
Citria
Glen Ericht
Himbotop
Kweli
Paris
Tulameen
Concentration, ppbV
0.0
2.5
5.0
7.5
P
R
OR
Type
stage
P
R
OR

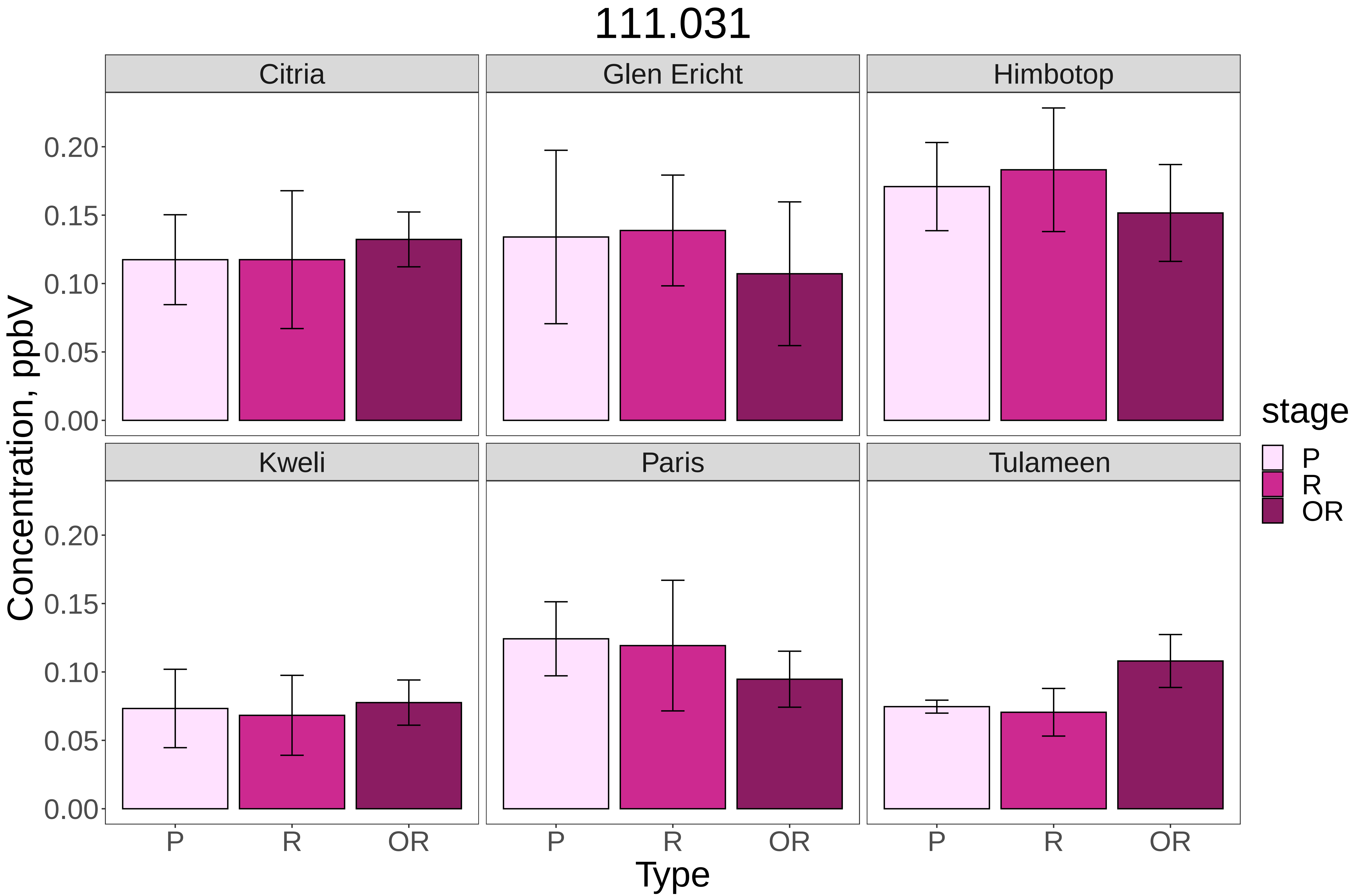

111.031
Citria
Glen Ericht
Himbotop
Kweli
Paris
Tulameen
Concentration, ppbV
0.00
0.05
0.10
0.15
0.20
Type
P
R
OR
stage
P
R
OR

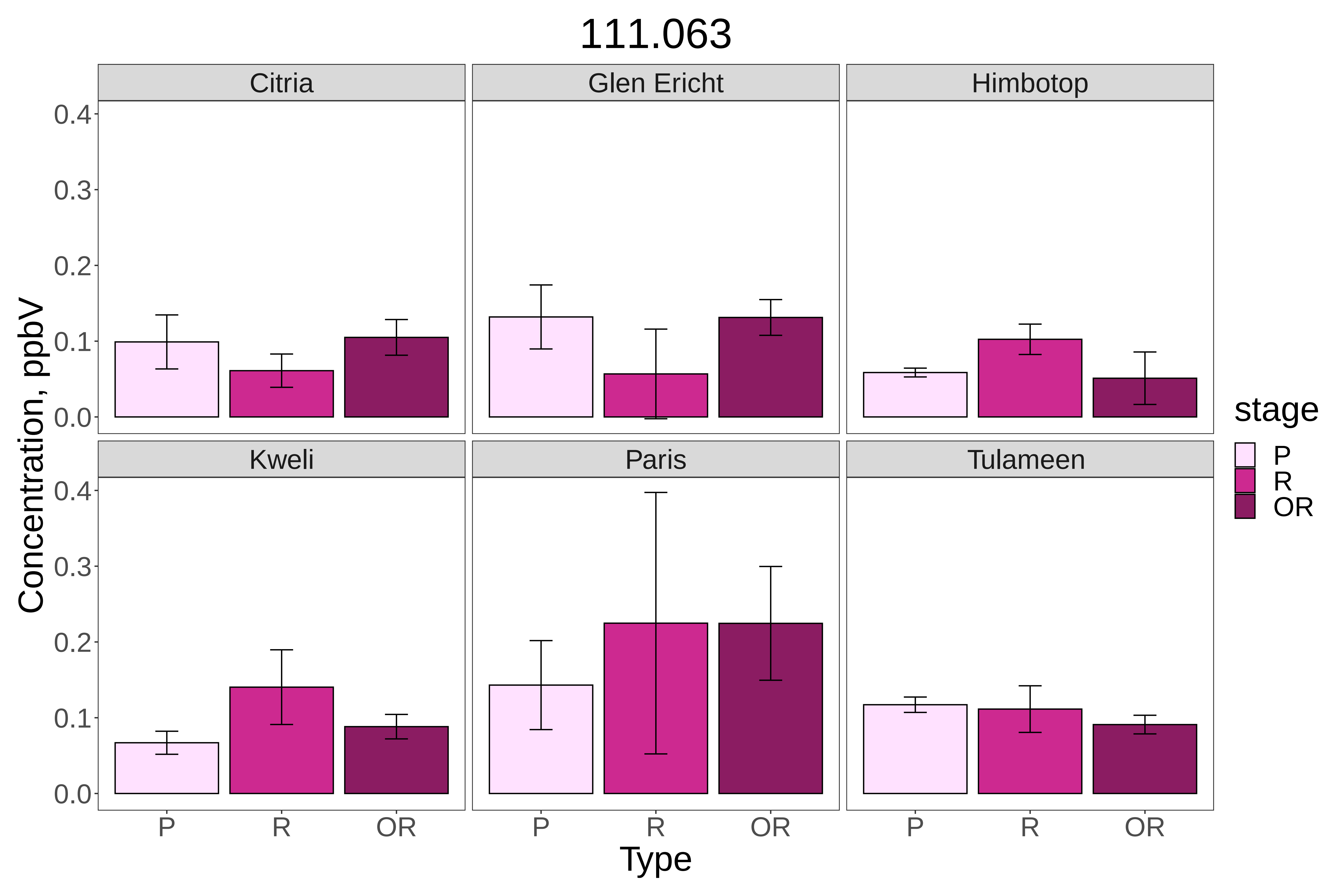
111.063
Citria
Glen Ericht
Himbotop
Kweli
Paris
Tulameen
Concentration, ppbV
0.0
0.1
0.2
0.3
0.4
Type
P
R
OR
stage
P
R
OR

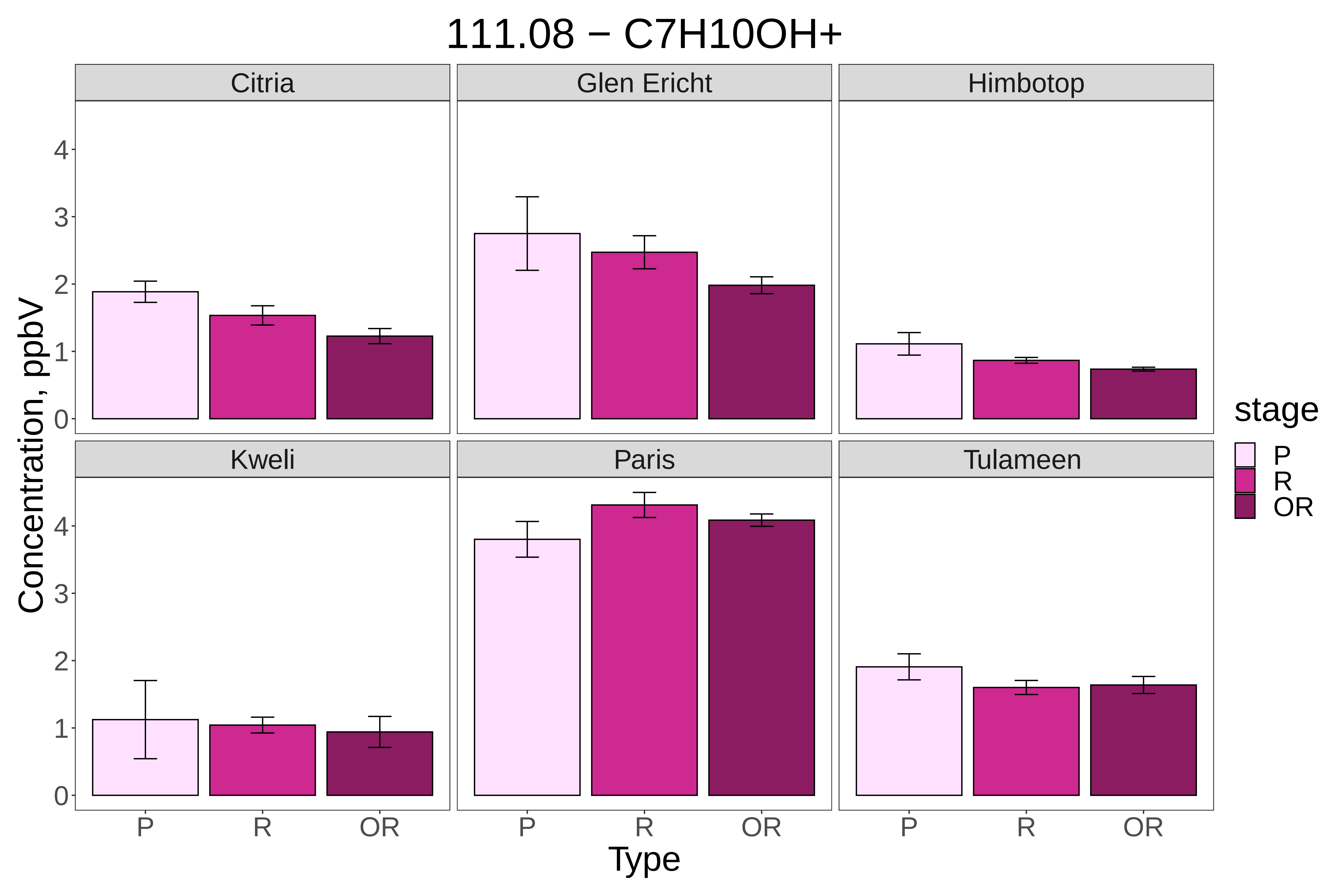

111.08 − C7H10OH+
Citria
Glen Ericht
Himbotop
Kweli
Paris
Tulameen
Concentration, ppbV
0
1
2
3
4
P
R
OR
Type
stage
P
R
OR

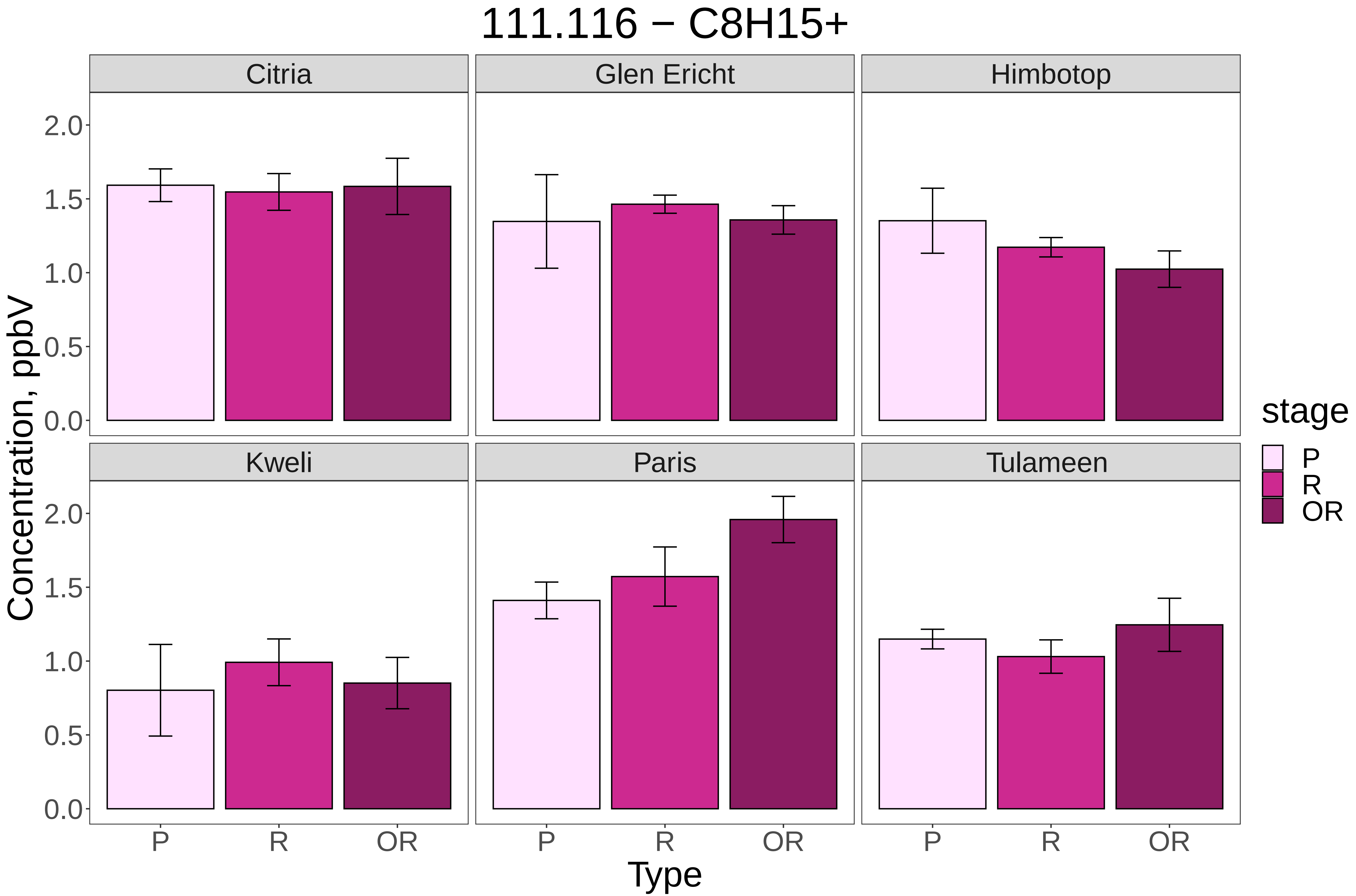
111.116 − C8H15+
Citria
Glen Ericht
Himbotop
Kweli
Paris
Tulameen
Concentration, ppbV
0.0
0.5
1.0
1.5
2.0
P
R
OR
Type
stage
P
R
OR

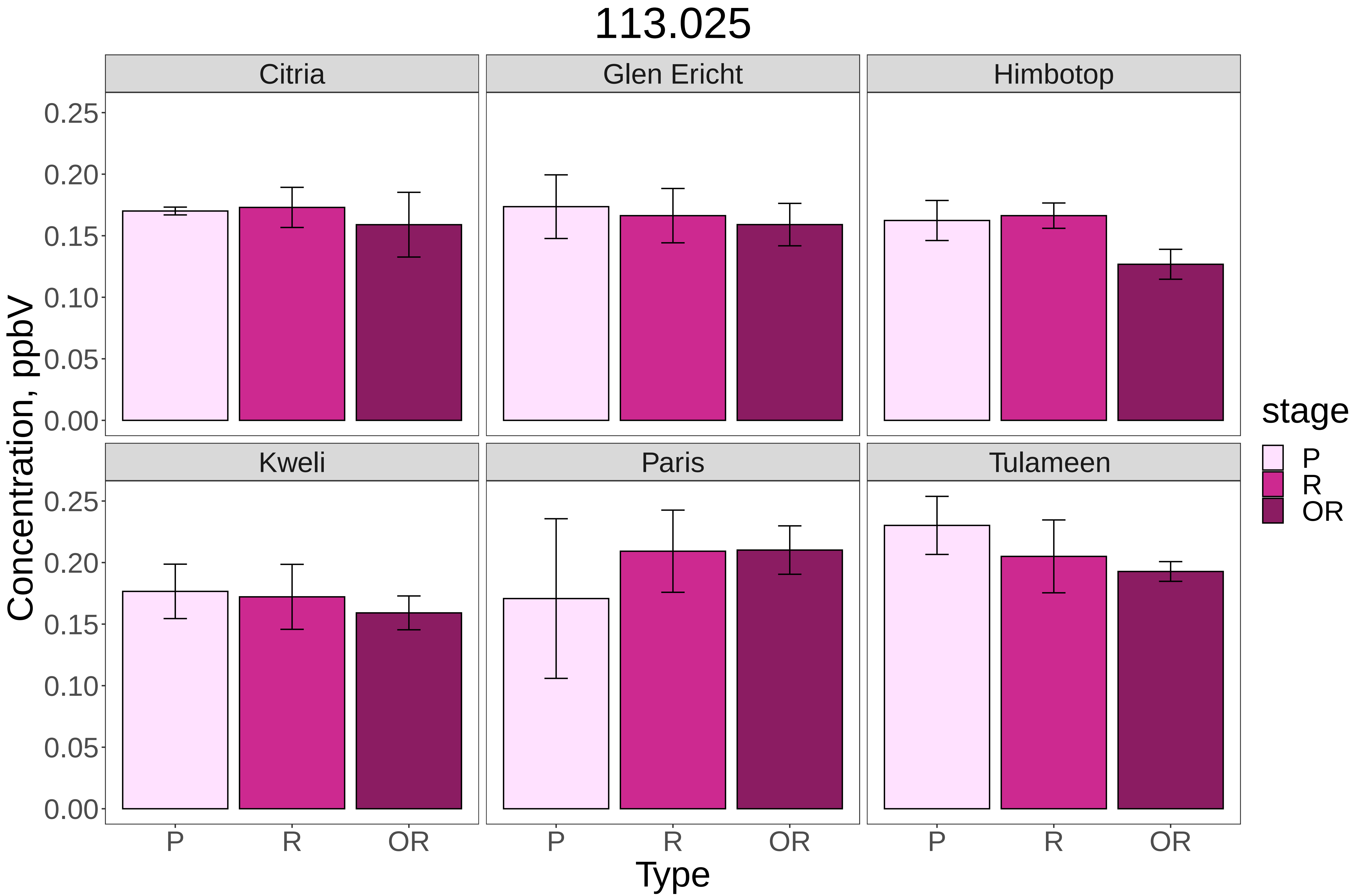

113.025
Citria
Glen Ericht
Himbotop
Kweli
Paris
Tulameen
0.25
0.20
0.15
0.10
0.05
0.00
Concentration, ppbV
P
R
OR
Type
stage
P
R
OR

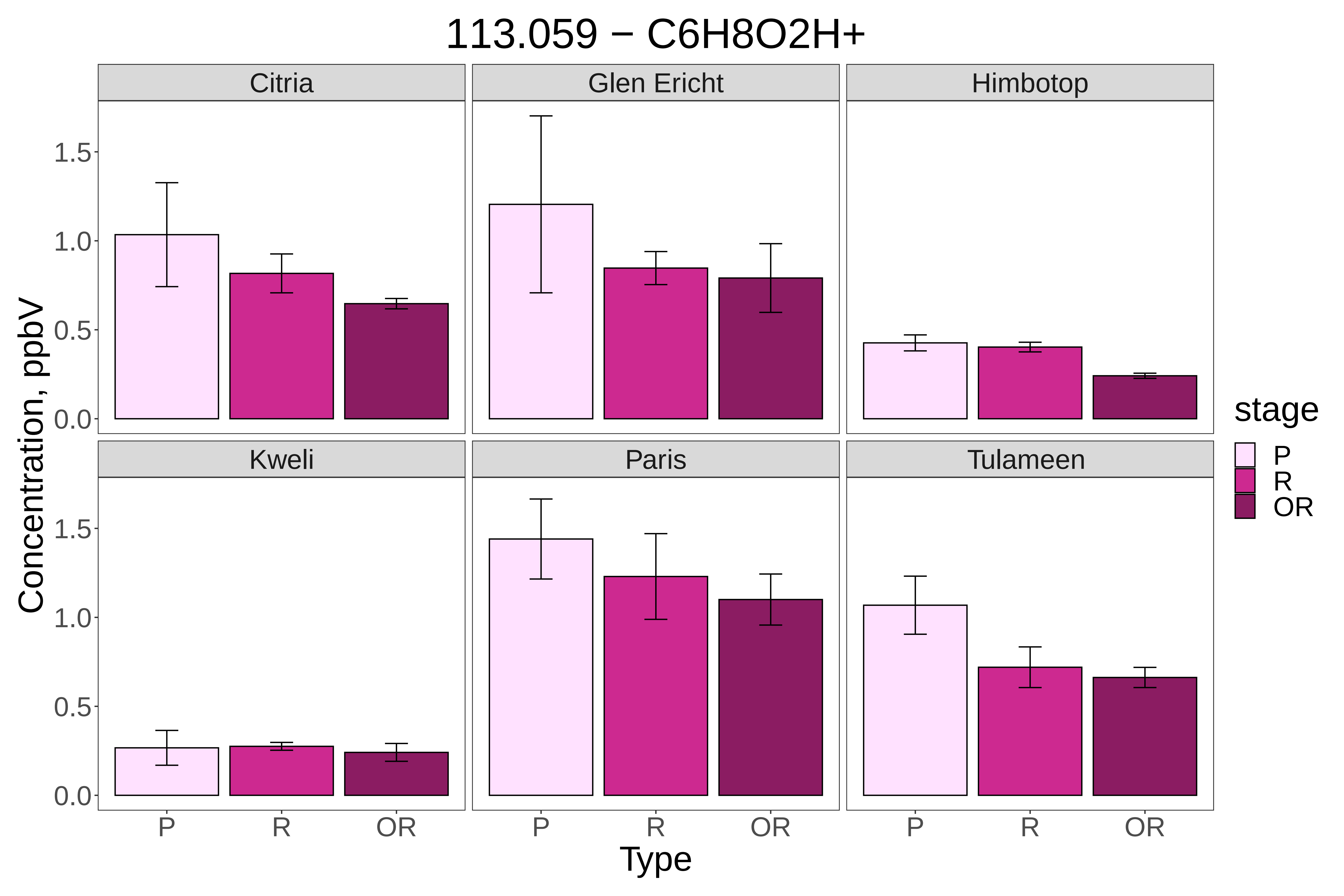
113.059 − C6H8O2H+
Citria
Glen Ericht
Himbotop
Kweli
Paris
Tulameen
Concentration, ppbV
0.0
0.5
1.0
1.5
P
R
OR
Type
stage
P
R
OR

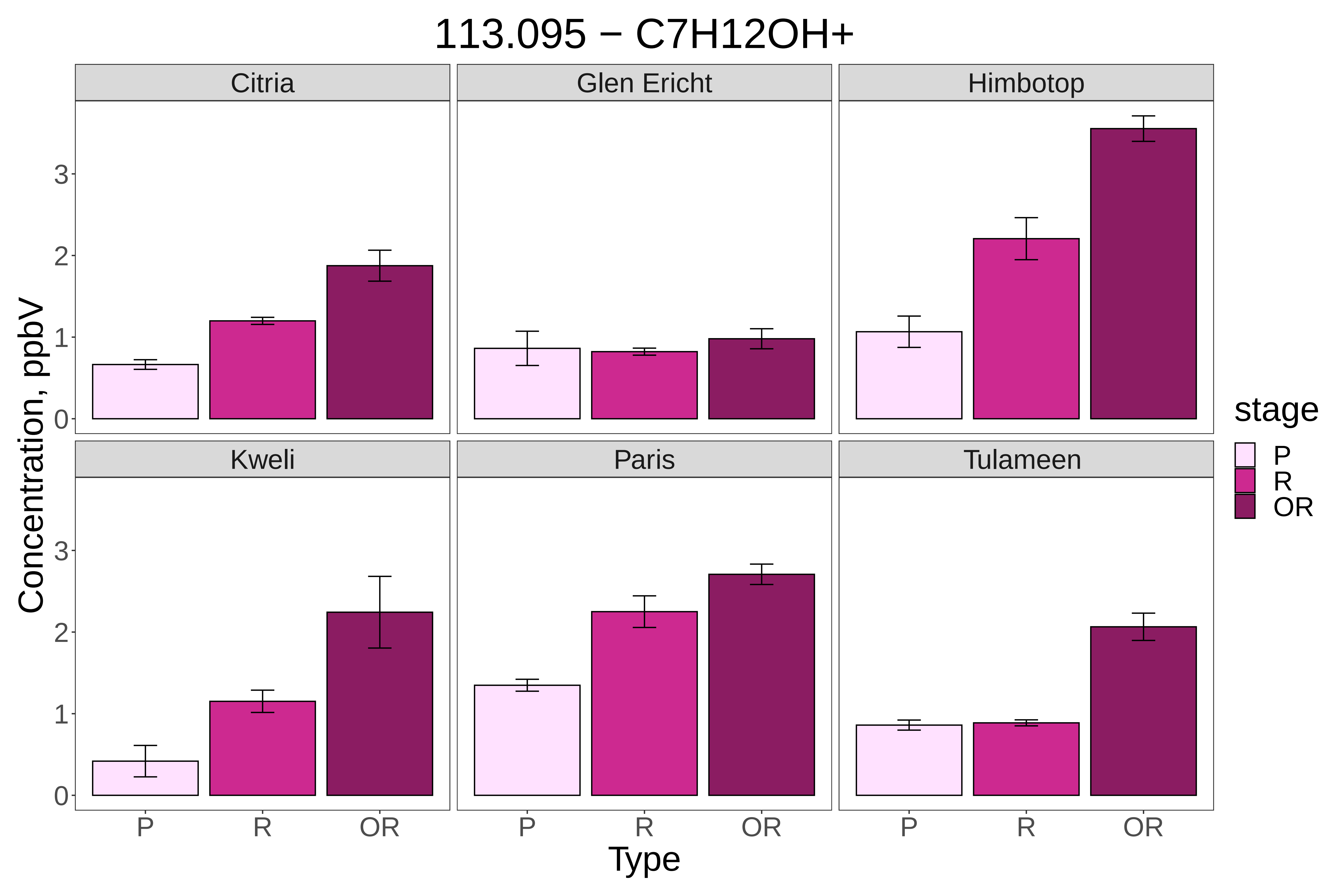

113.095 − C7H12OH+
Citria
Glen Ericht
Himbotop
Kweli
Paris
Tulameen
Concentration, ppbV
0
1
2
3
P
R
OR
Type
stage
P
R
OR

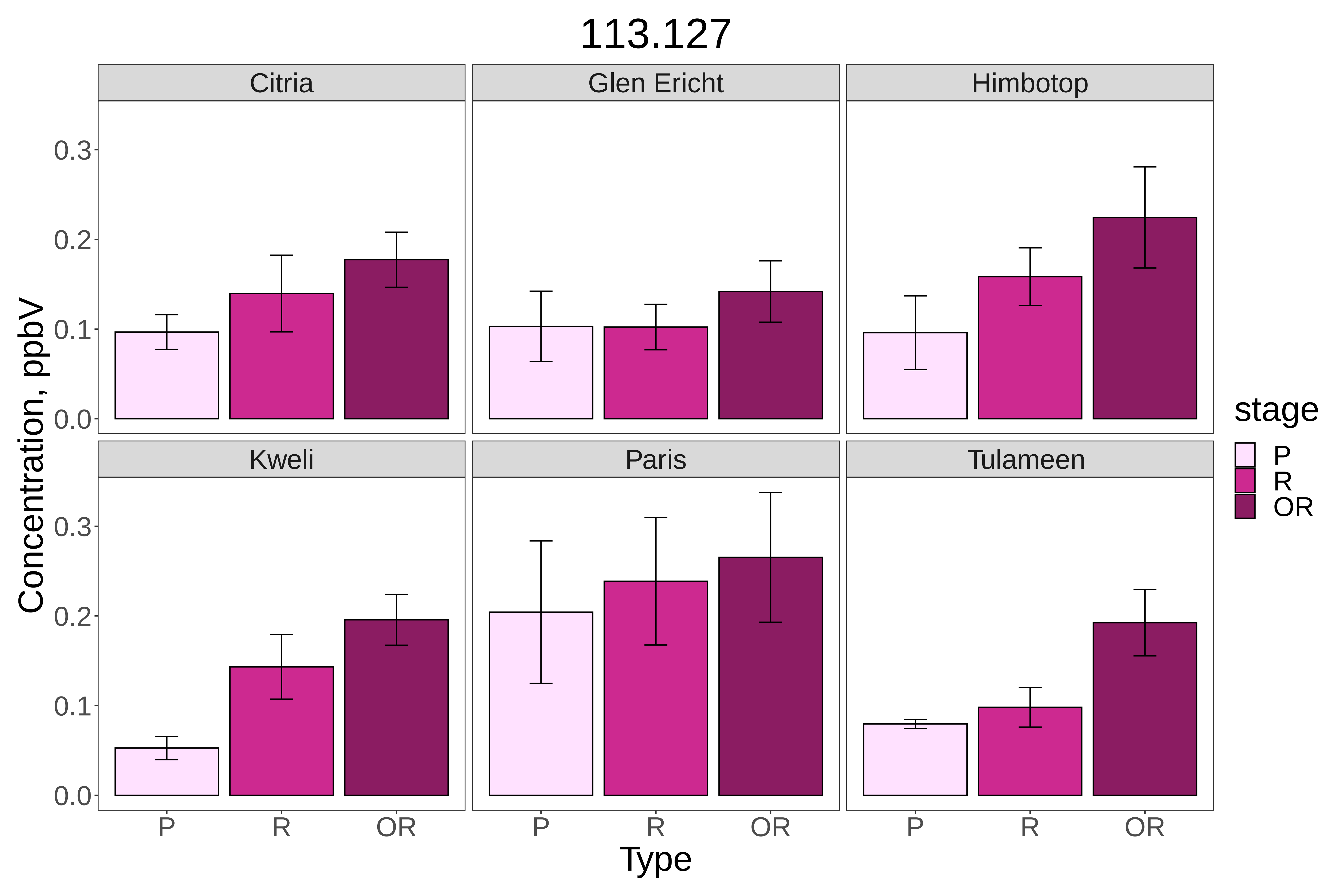

113.127
Citria
Glen Ericht
Himbotop
Kweli
Paris
Tulameen
Concentration, ppbV
0.0
0.1
0.2
0.3
P
R
OR
Type
stage
P
R
OR

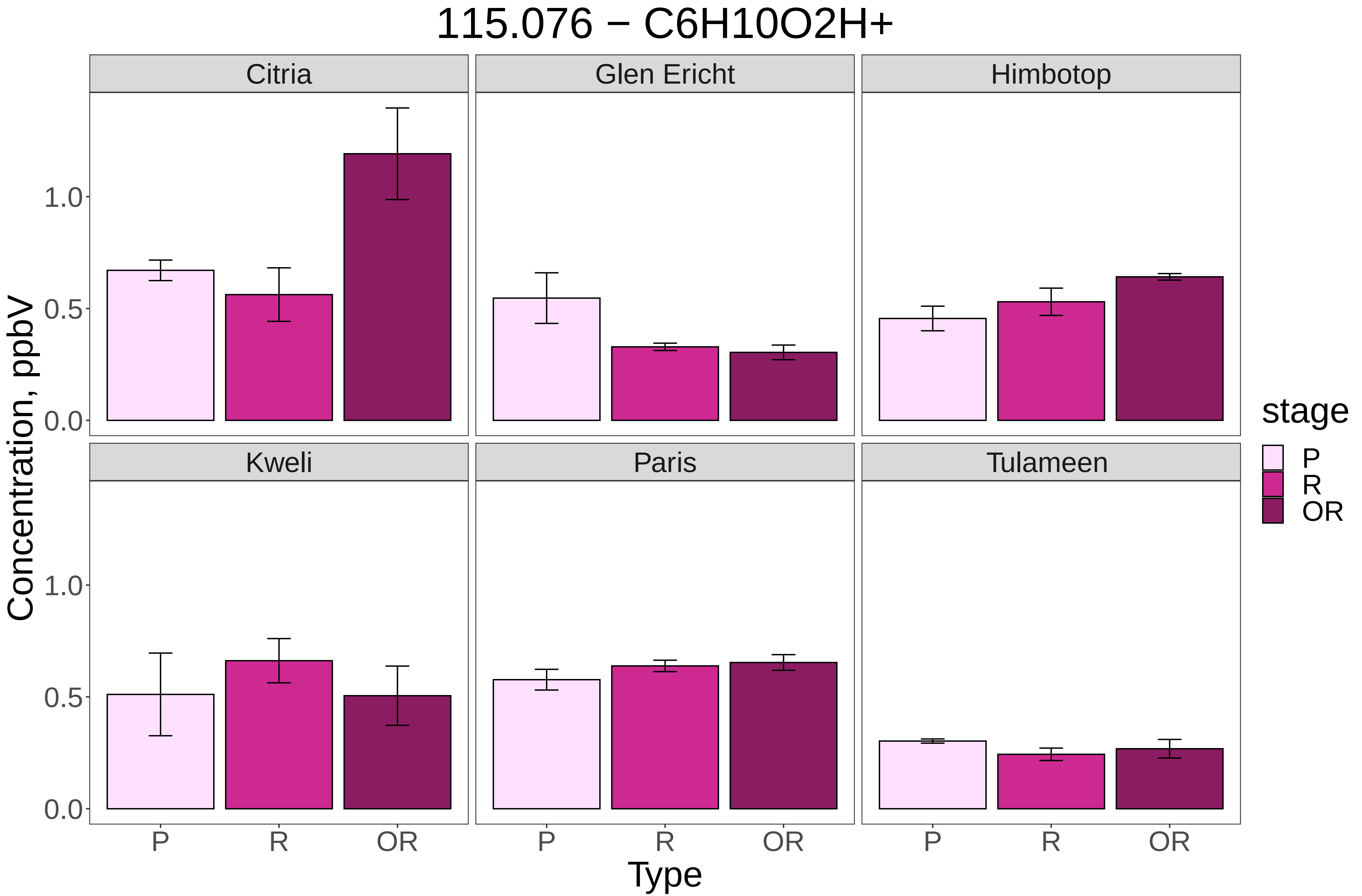

115.076 − C6H10O2H+
Citria
Glen Ericht
Himbotop
Kweli
Paris
Tulameen
Concentration, ppbV
0.0
0.5
1.0
P
R
OR
Type
stage
P
R
OR

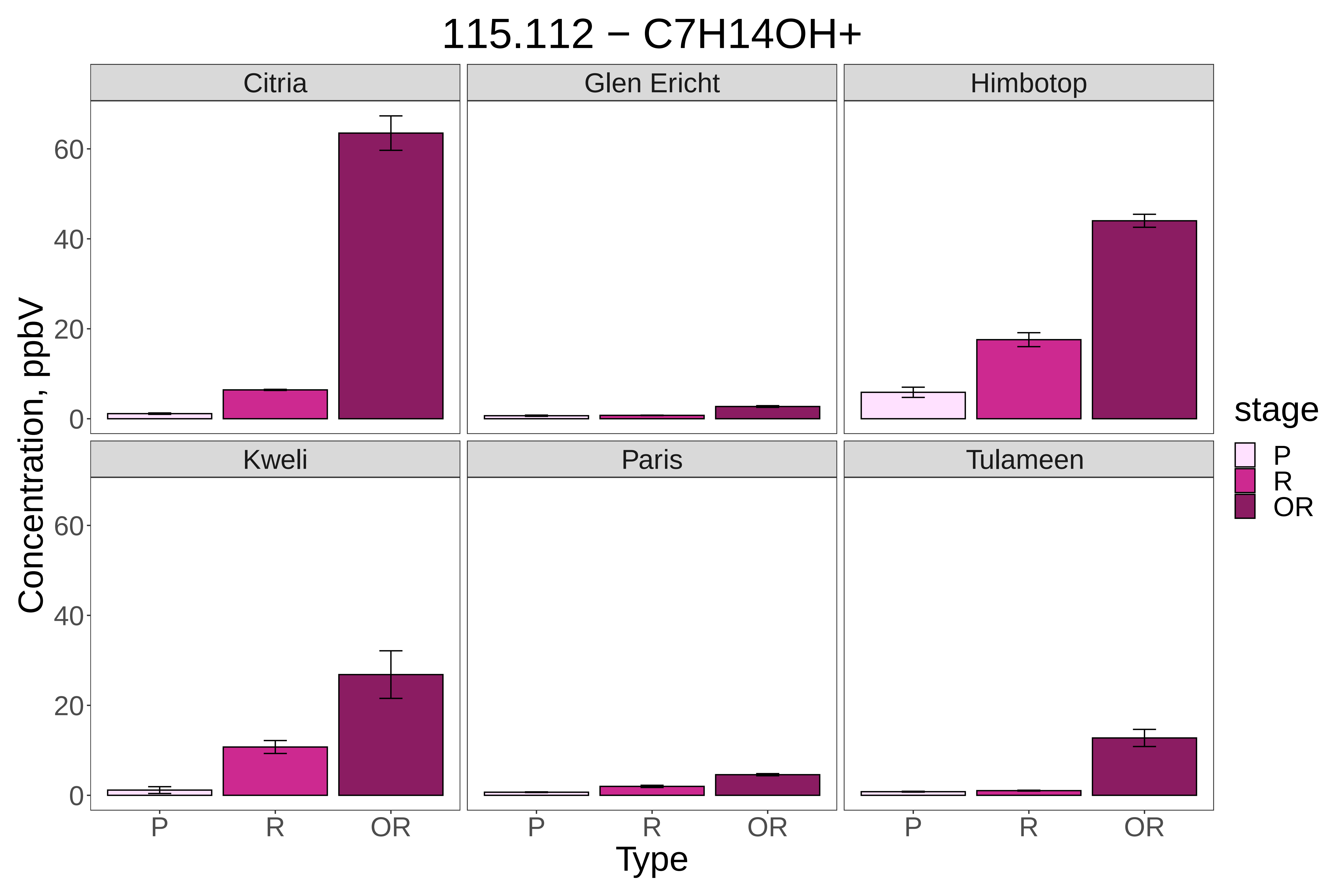

115.112 − C7H14OH+
Citria
Glen Ericht
Himbotop
Kweli
Paris
Tulameen
Concentration, ppbV
0
20
40
60
P
R
OR
Type
stage
P
R
OR

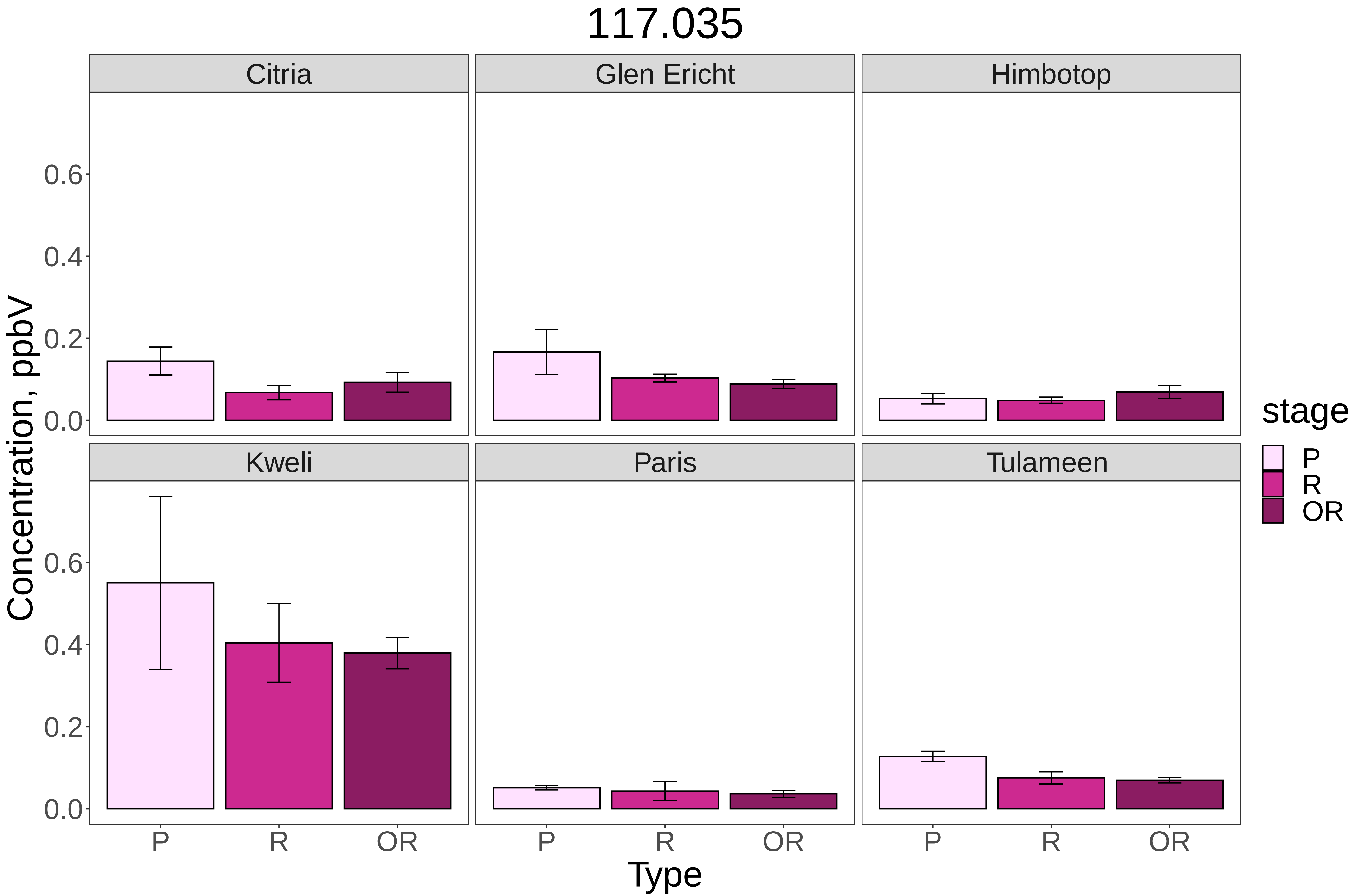
117.035
Citria
Glen Ericht
Himbotop
Kweli
Paris
Tulameen
Concentration, ppbV
0.0
0.2
0.4
0.6
P
R
OR
Type
stage
P
R
OR

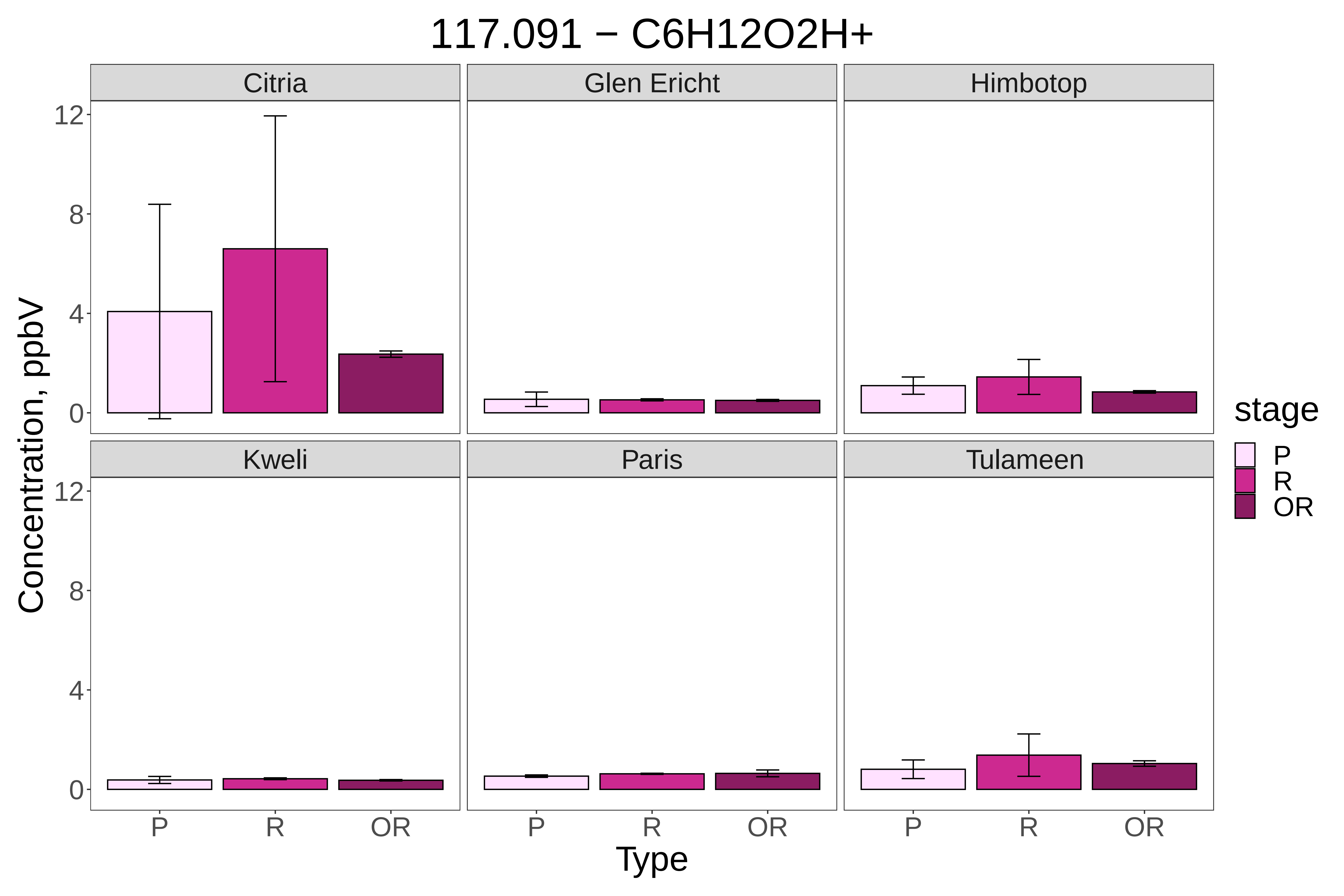

117.091 − C6H12O2H+
Citria
Glen Ericht
Himbotop
Kweli
Paris
Tulameen
Concentration, ppbV
0
4
8
12
P
R
OR
Type
stage

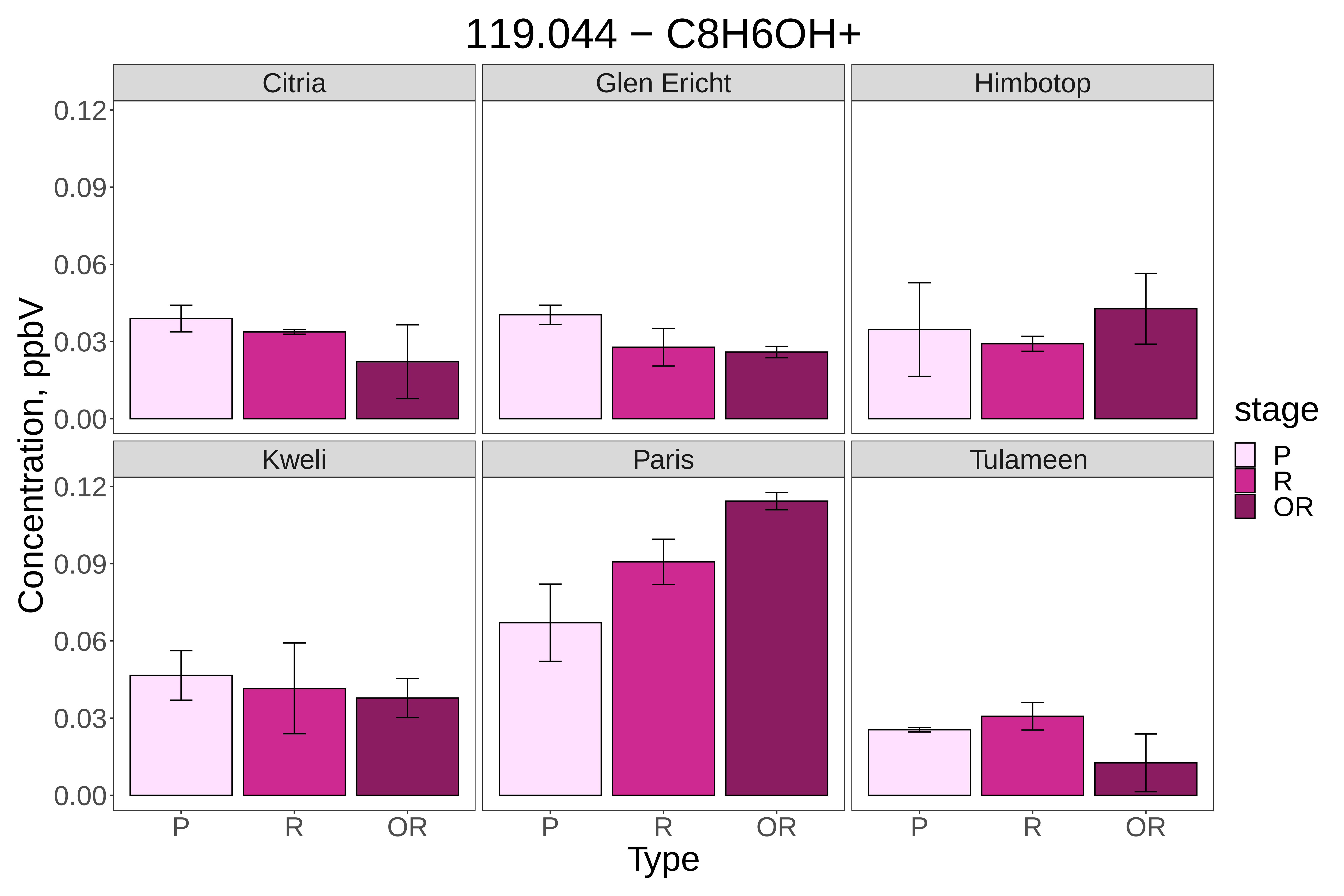

119.044 − C8H6OH+
Citria
Glen Ericht
Himbotop
Kweli
Paris
Tulameen
Concentration, ppbV
0.00
0.03
0.06
0.09
0.12
P
R
OR
Type
stage

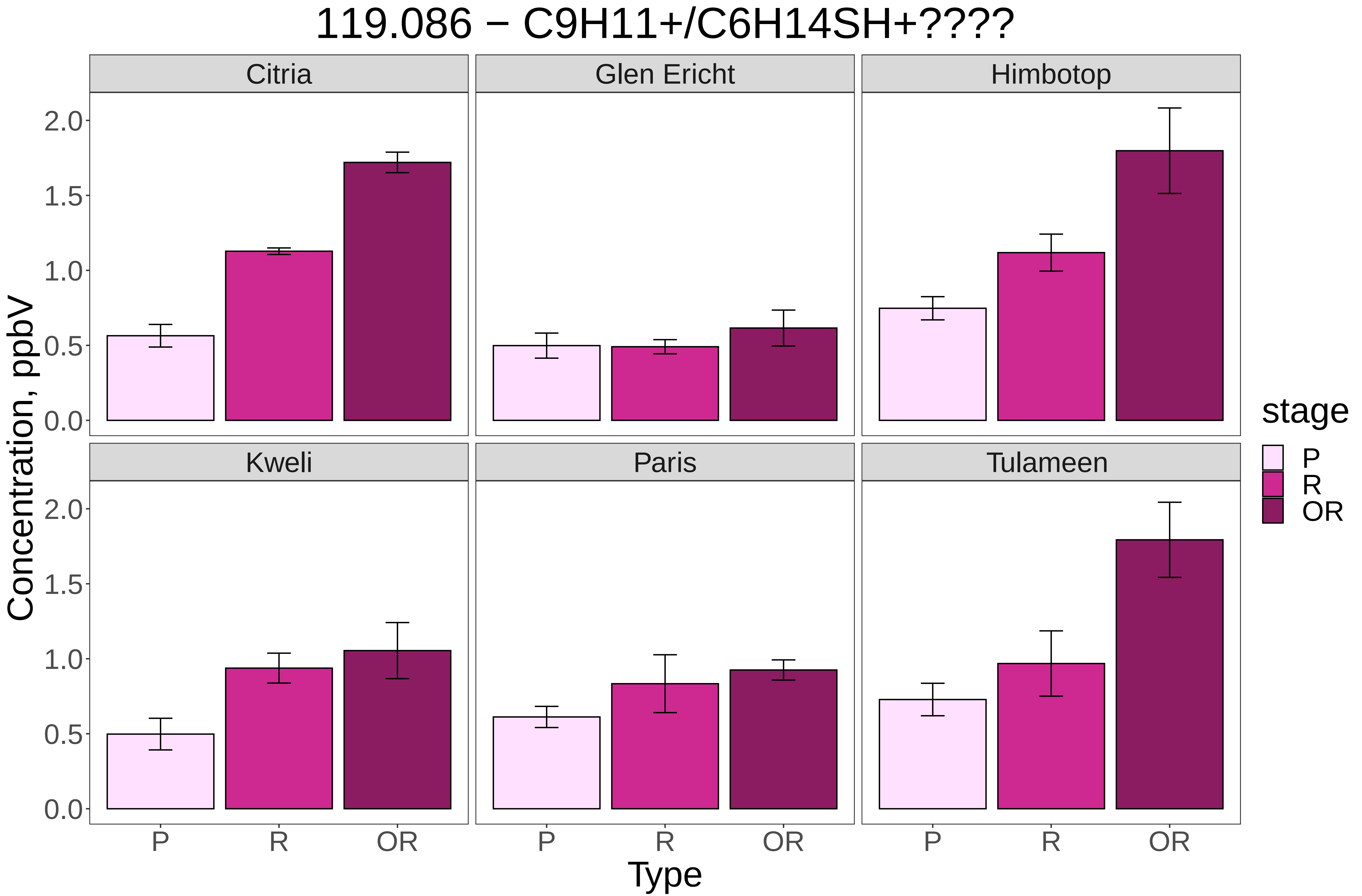

119.086 − C9H11+/C6H14SH+????
Citria
Glen Ericht
Himbotop
Kweli
Paris
Tulameen
Concentration, ppbV
0.0
0.5
1.0
1.5
2.0
P
R
OR
Type
stage
P
R
OR

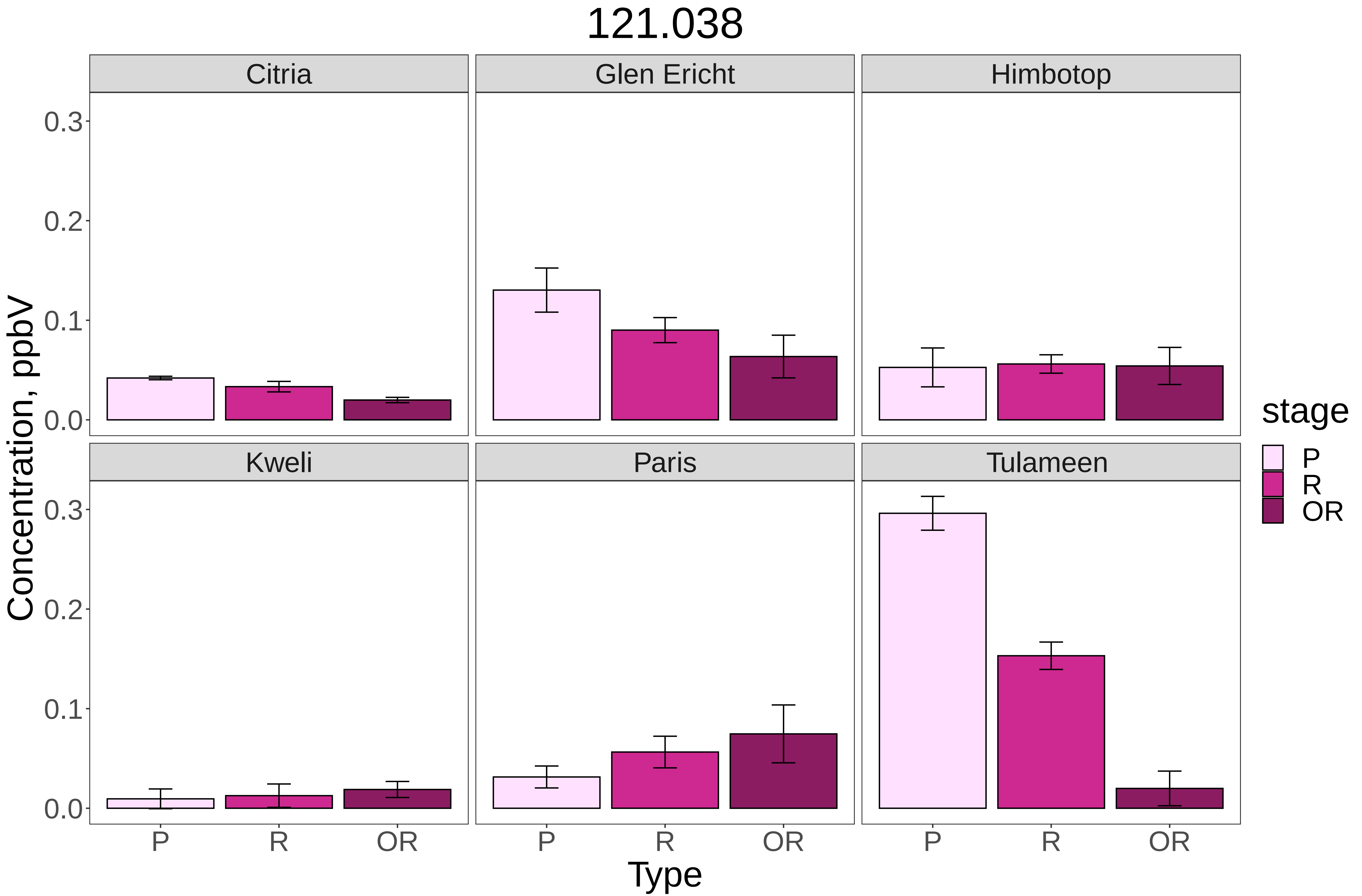
121.038
Citria
Glen Ericht
Himbotop
Kweli
Paris
Tulameen
Concentration, ppbV
0.0
0.1
0.2
0.3
P
R
OR
Type
stage
P
R
OR

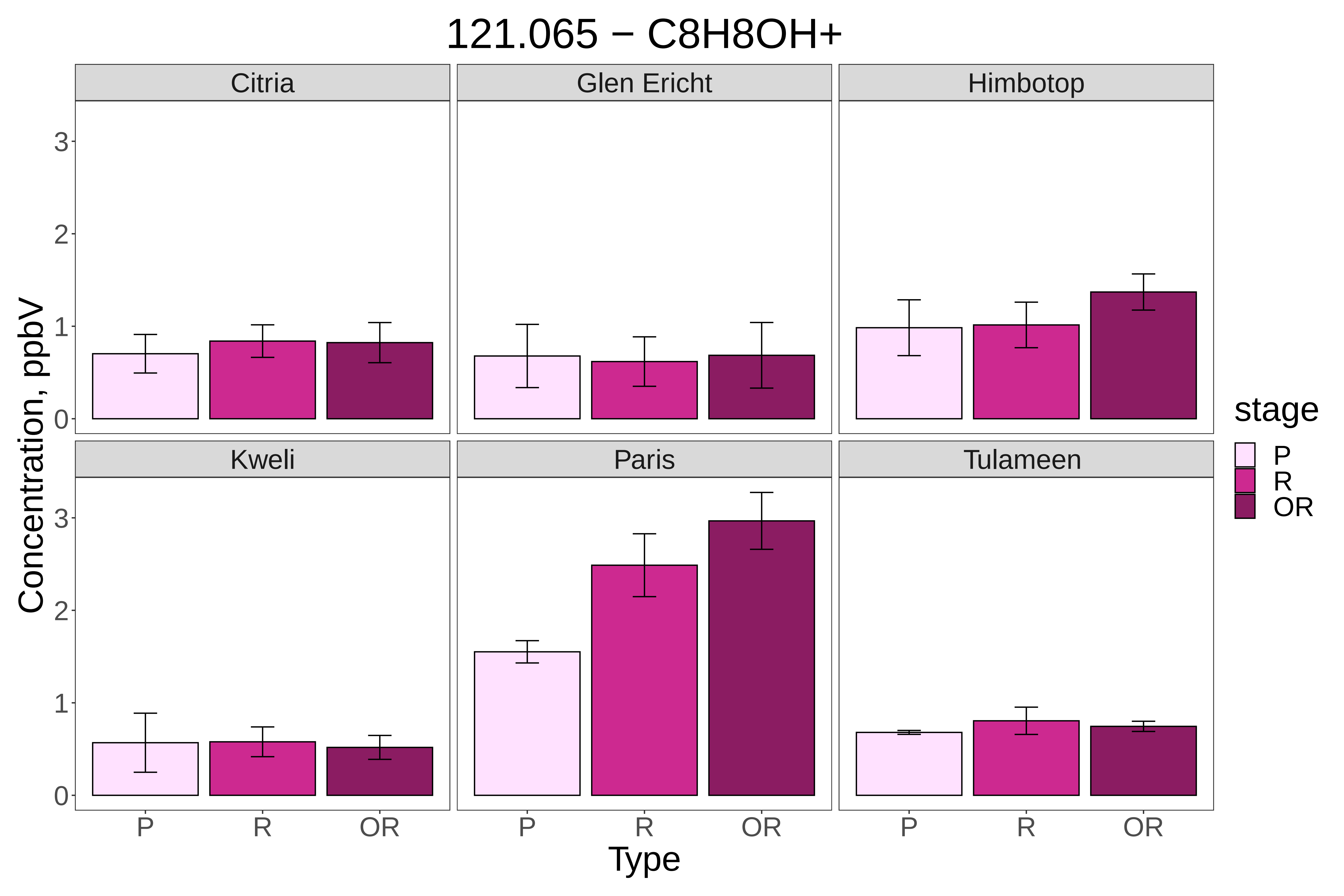

121.065 − C8H8OH+
Citria
Glen Ericht
Himbotop
Kweli
Paris
Tulameen
Concentration, ppbV
0
1
2
3
P
R
OR
Type
stage
P
R
OR

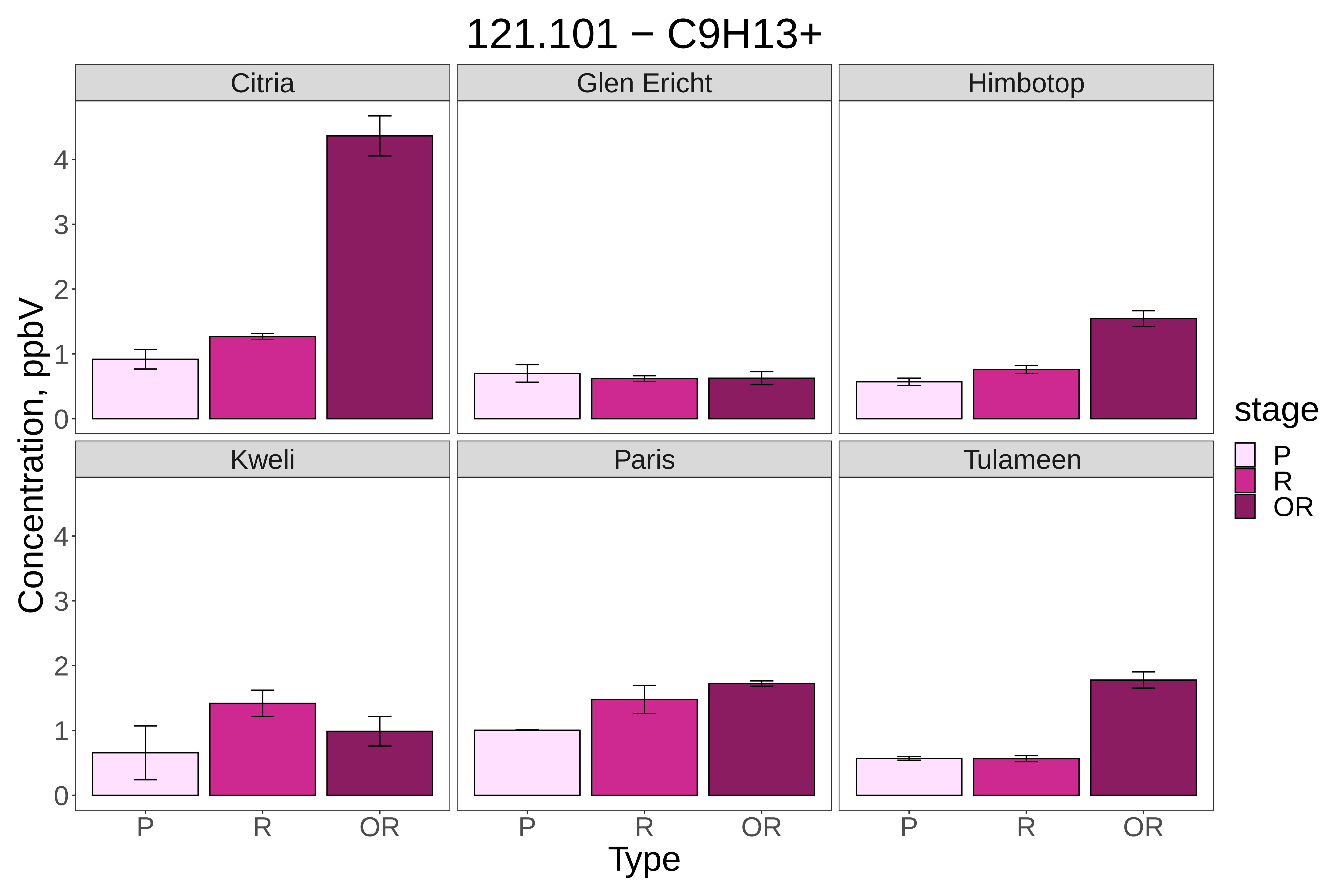

121.101 − C9H13+
Citria
Glen Ericht
Himbotop
Kweli
Paris
Tulameen
Concentration, ppbV
0
1
2
3
4
P
R
OR
Type
stage
P
R
OR

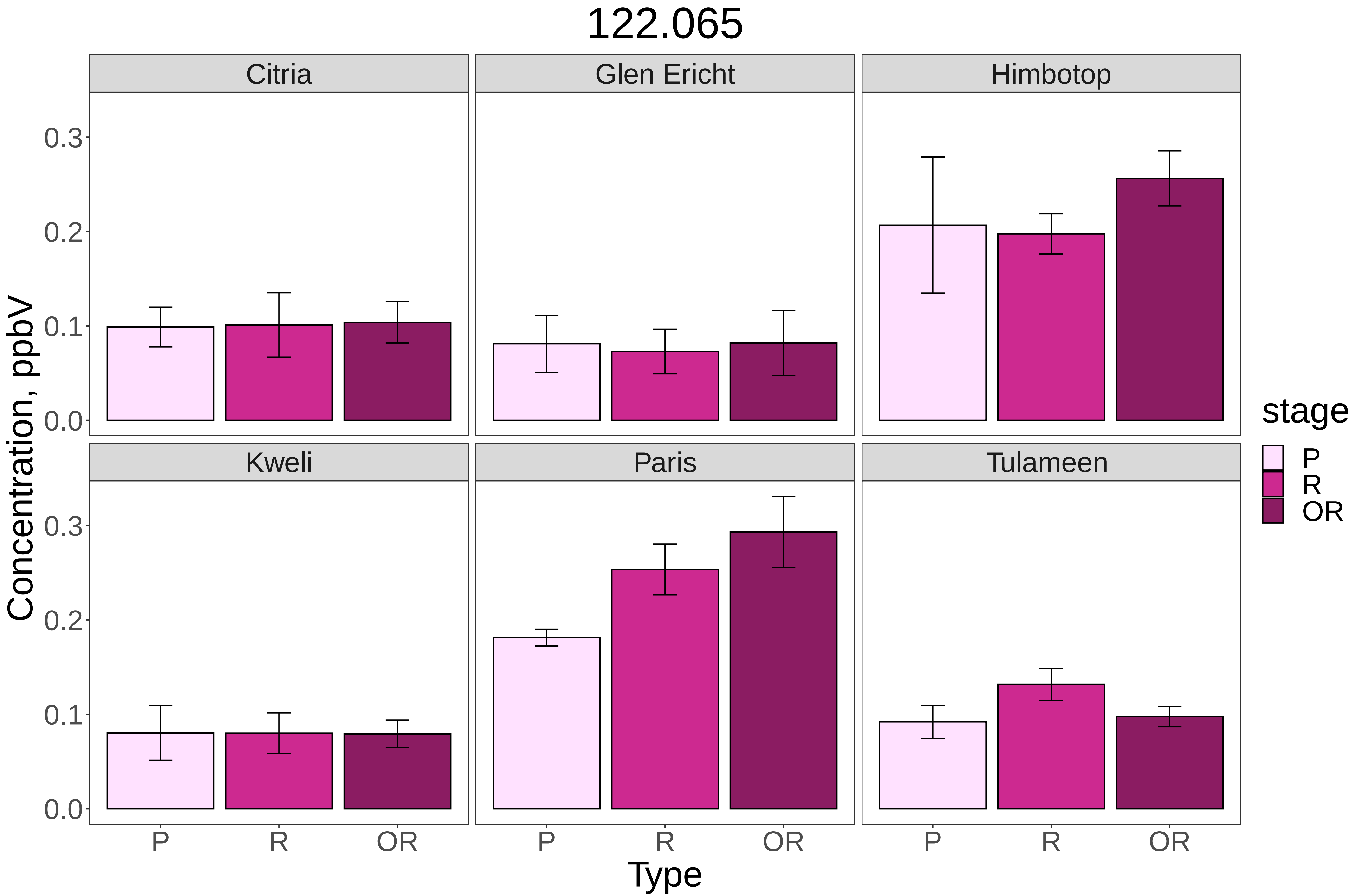

122.065
Citria
Glen Ericht
Himbotop
Kweli
Paris
Tulameen
Concentration, ppbV
0.0
0.1
0.2
0.3
P
R
OR
Type
stage
P
R
OR

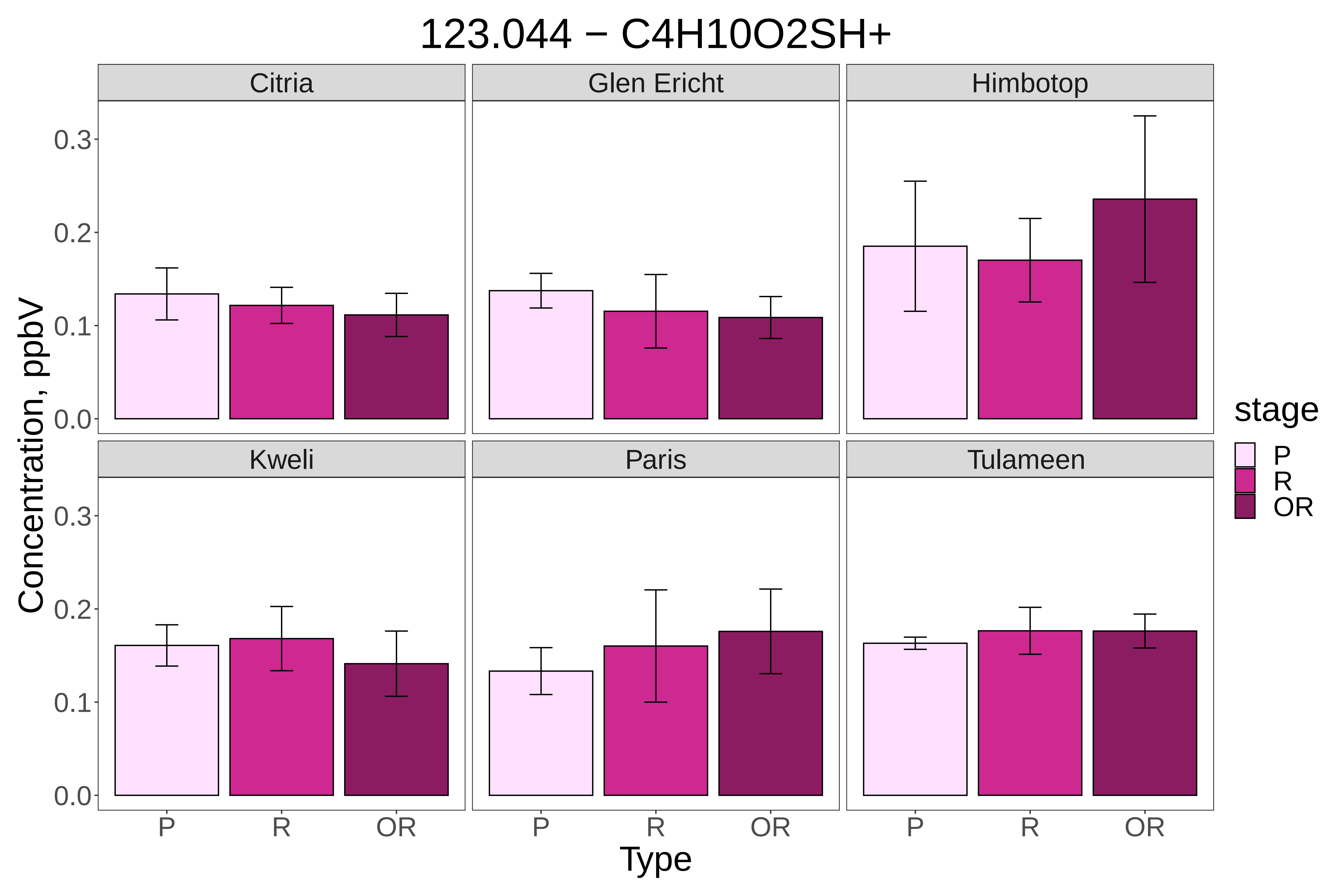

123.044 − C4H10O2SH+
Citria
Glen Ericht
Himbotop
Kweli
Paris
Tulameen
Concentration, ppbV
0.0
0.1
0.2
0.3
P
R
OR
Type
stage
P
R
OR

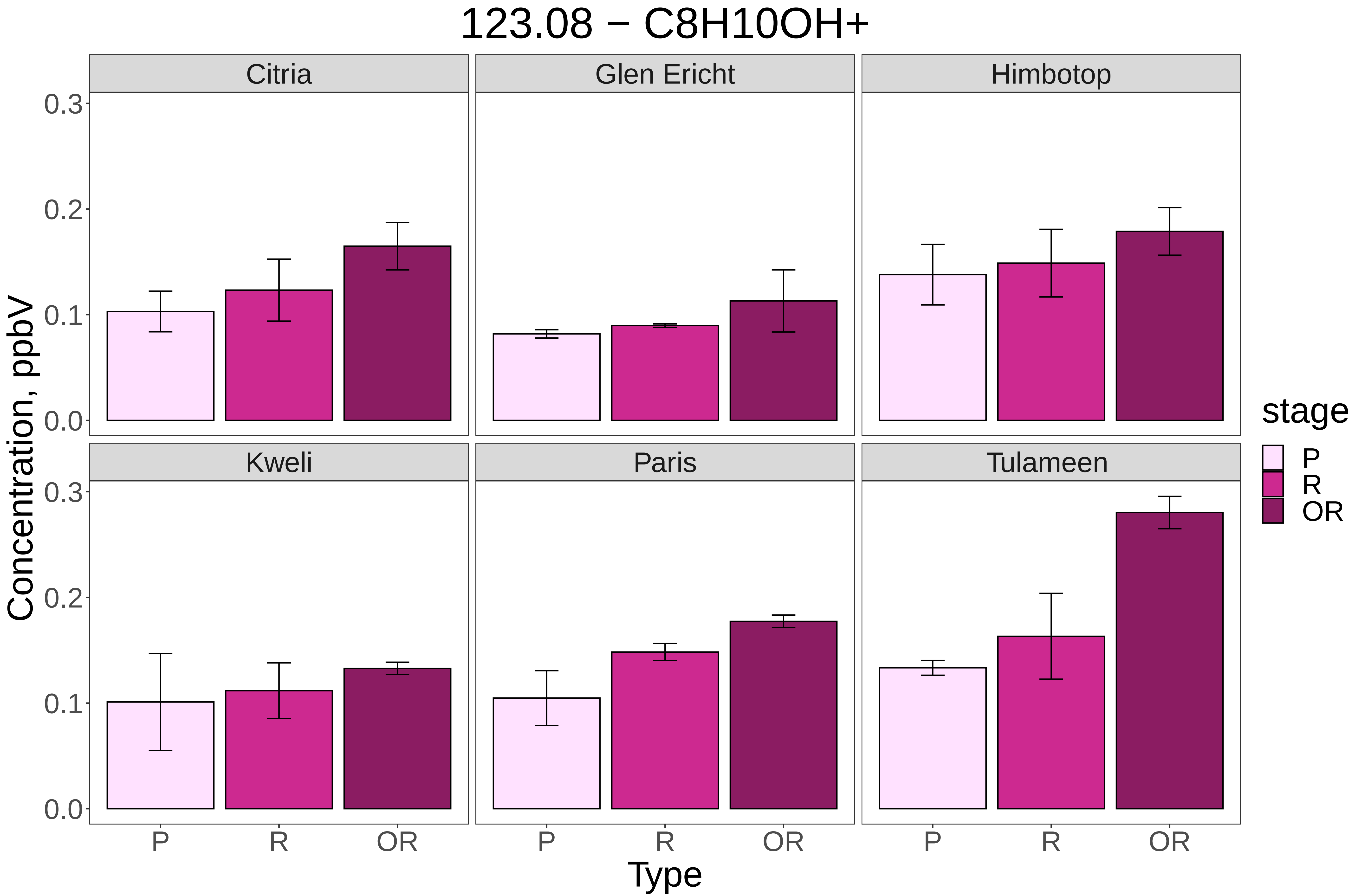
123.08 − C8H10OH+
Citria
Glen Ericht
Himbotop
Kweli
Paris
Tulameen
0.0
0.1
0.2
0.3
Concentration, ppbV
P
R
OR
Type
stage
P
R
OR

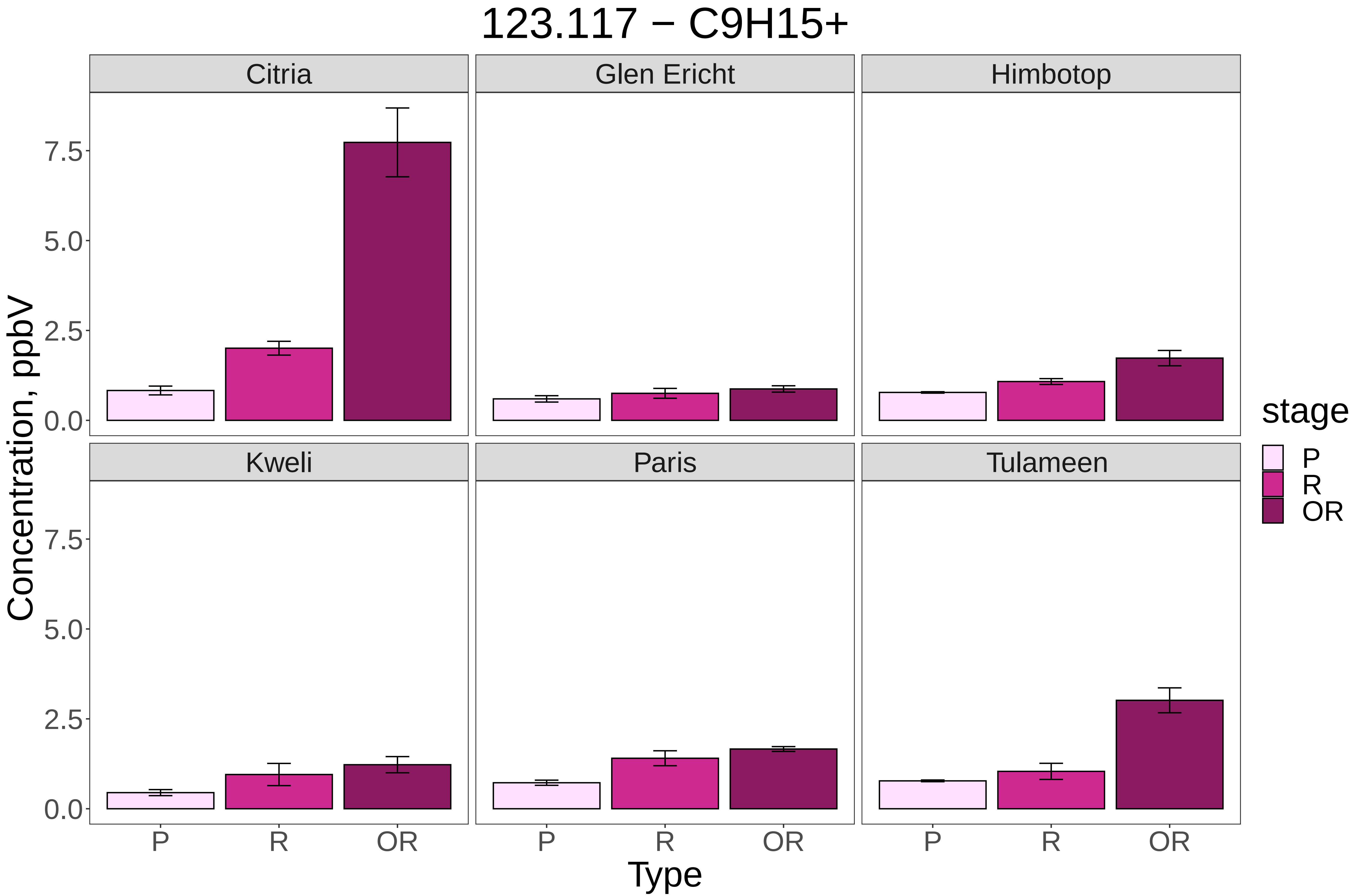

123.117 − C9H15+
Citria
Glen Ericht
Himbotop
Kweli
Paris
Tulameen
Concentration, ppbV
0.0
2.5
5.0
7.5
P
R
OR
Type
stage

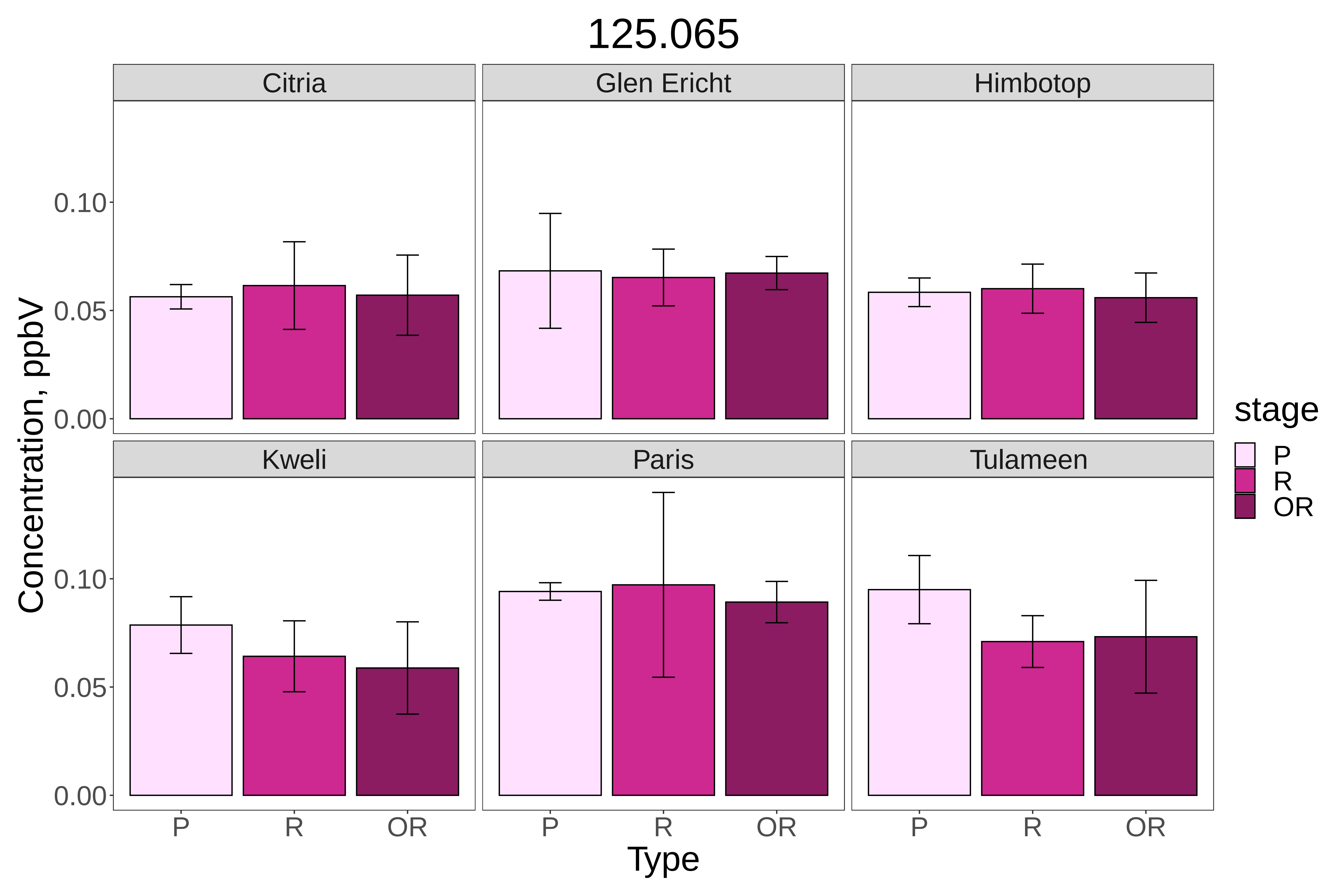
125.065
Citria
Glen Ericht
Himbotop
Kweli
Paris
Tulameen
Concentration, ppbV
0.00
0.05
0.10
Type
P
R
OR
stage
P
R
OR

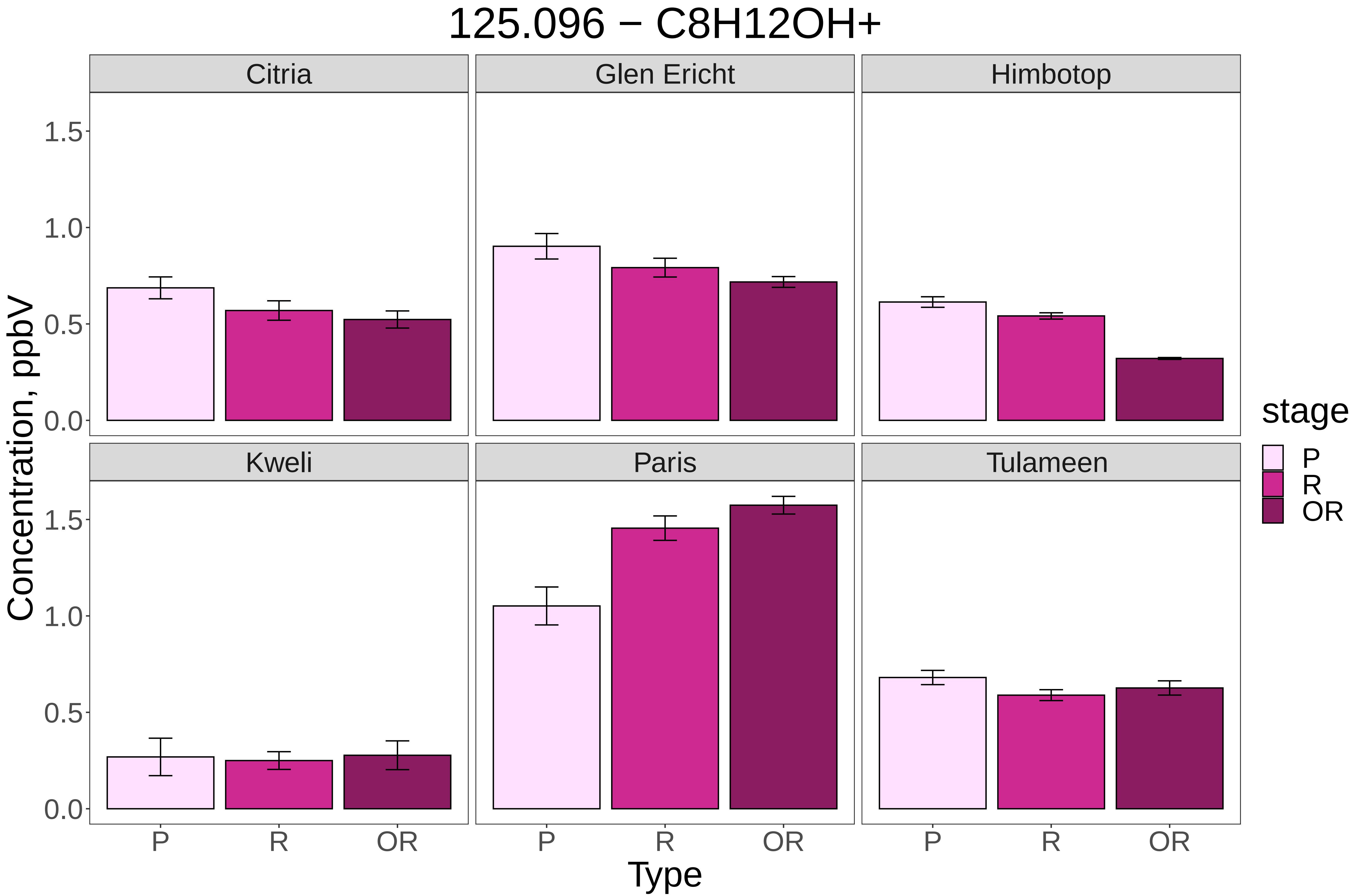

125.096 − C8H12OH+
Citria
Glen Ericht
Himbotop
Kweli
Paris
Tulameen
Concentration, ppbV
0.0
0.5
1.0
1.5
Type
P
R
OR
stage
P
R
OR

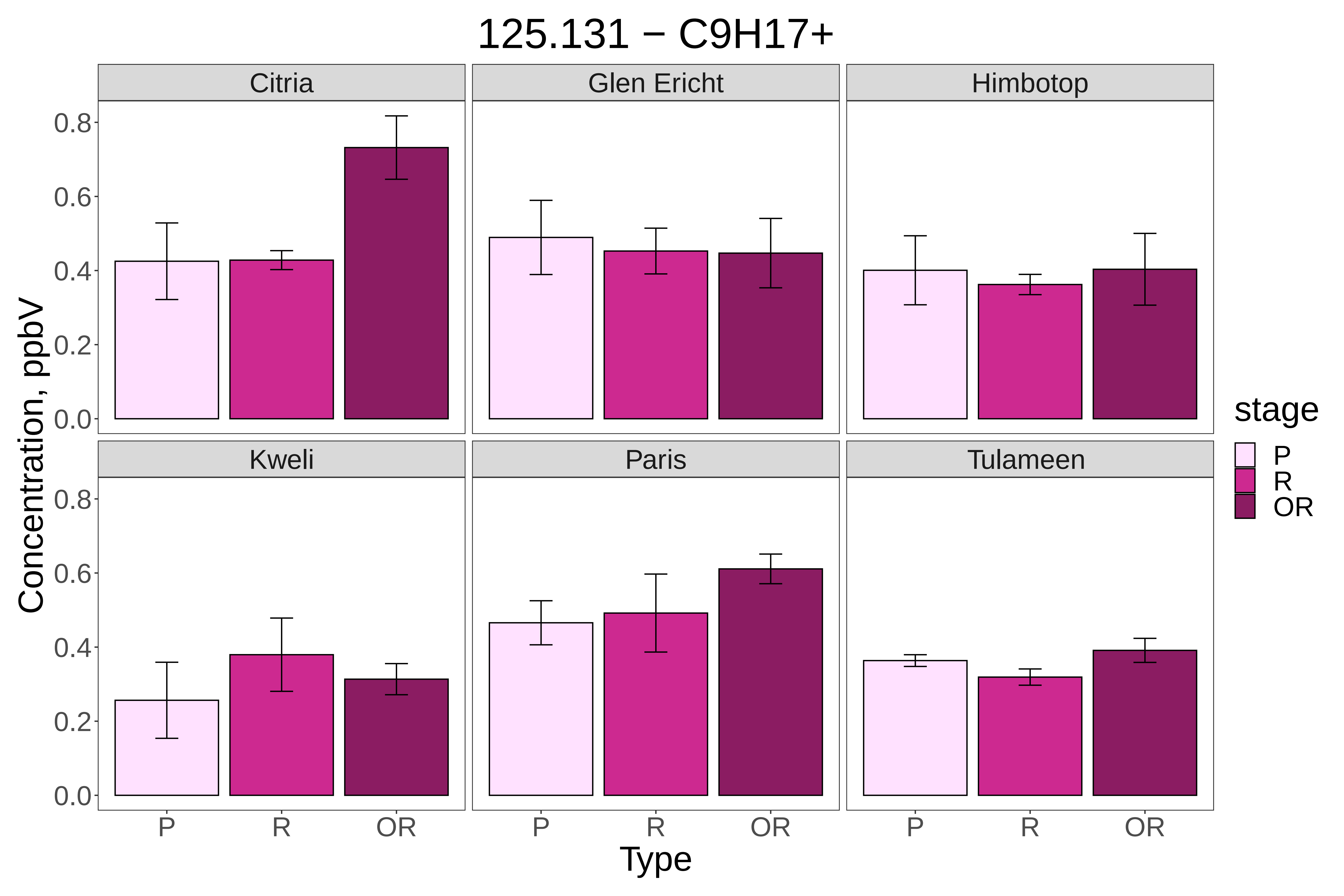

125.131 − C9H17+
Citria
Glen Ericht
Himbotop
Kweli
Paris
Tulameen
0.0
0.2
0.4
0.6
0.8
Concentration, ppbV
P
R
OR
Type
stage
P
R
OR

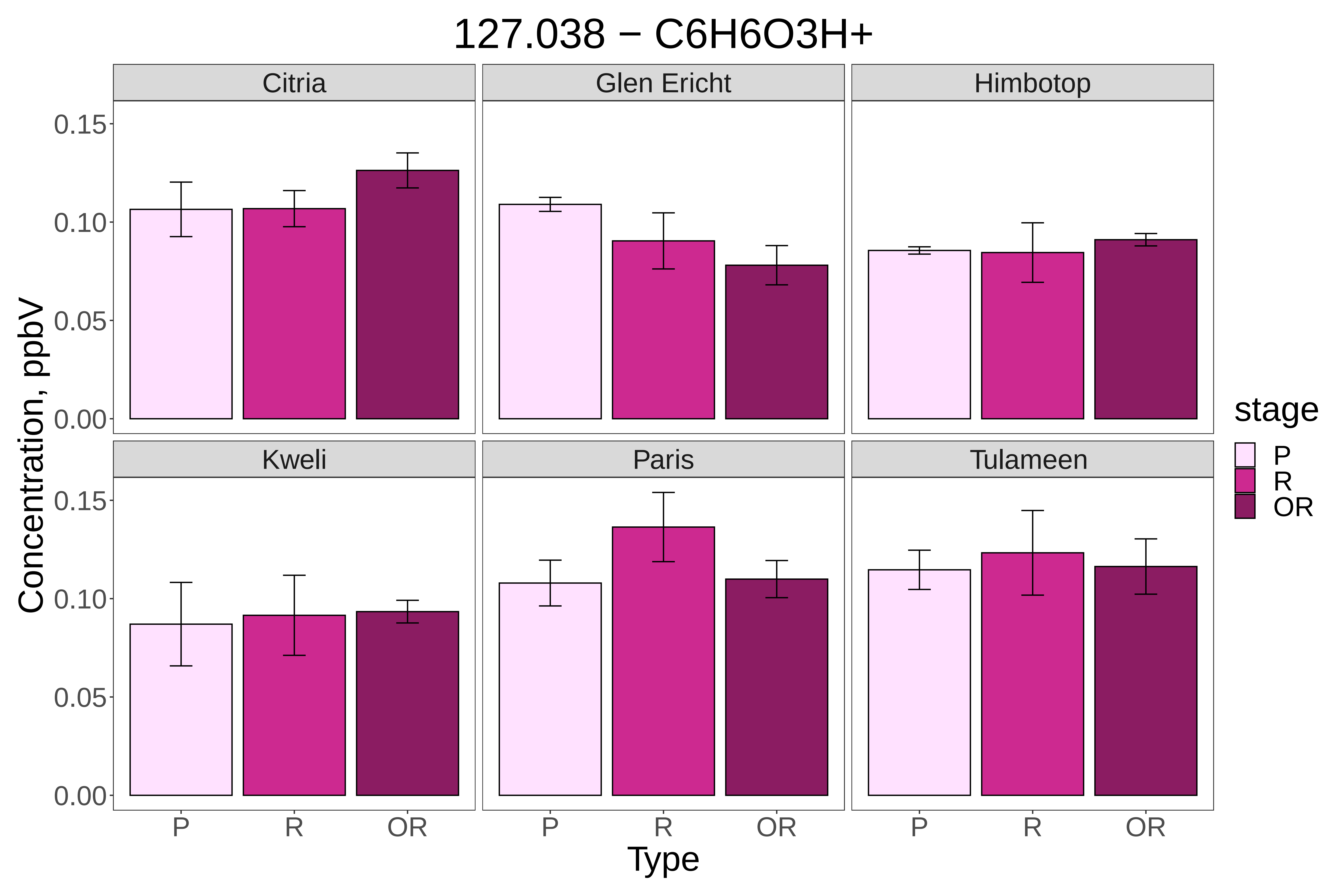
127.038 − C6H6O3H+
Citria
Glen Ericht
Himbotop
Kweli
Paris
Tulameen
Concentration, ppbV
0.00
0.05
0.10
0.15
P
R
OR
Type
stage

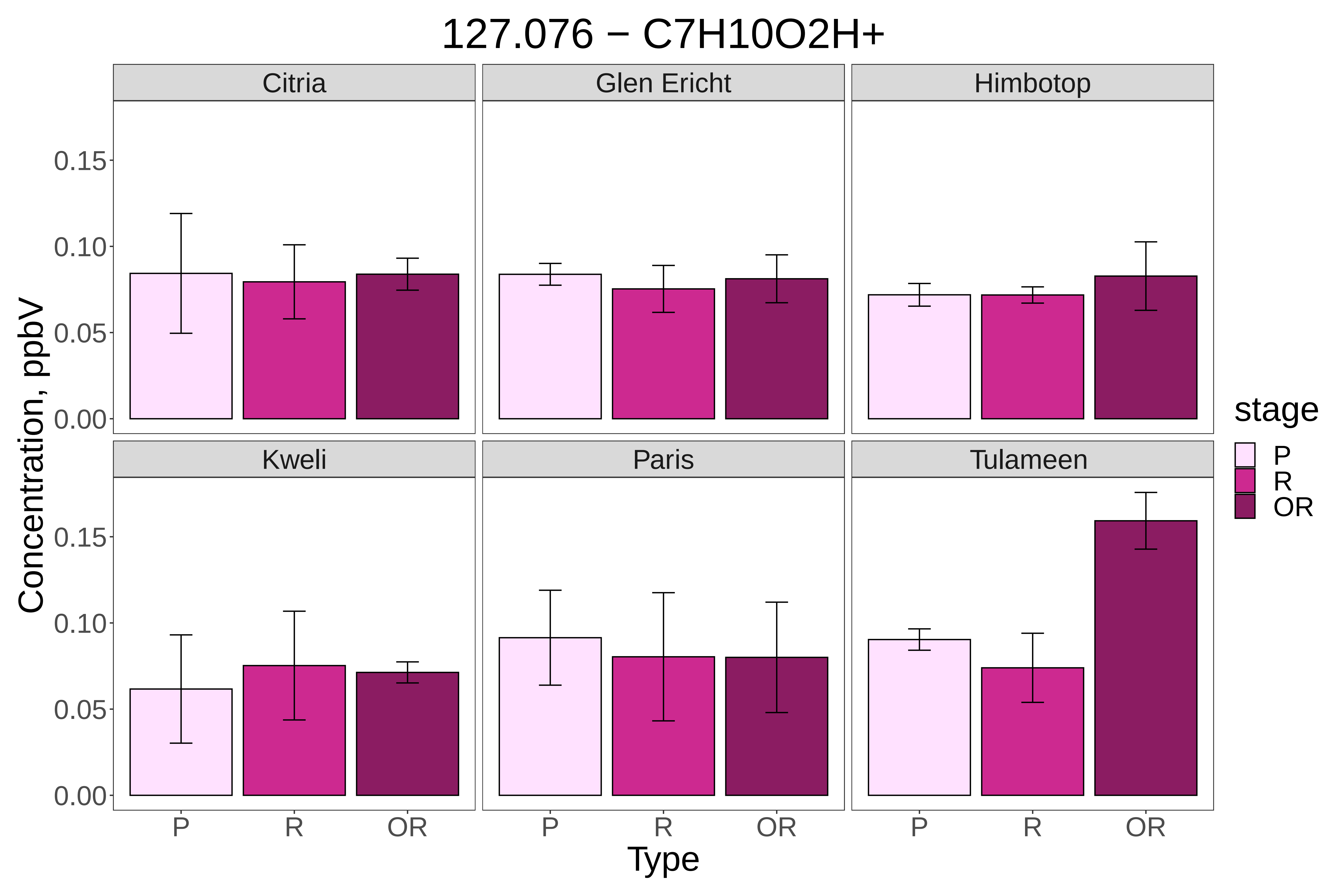

127.076 − C7H10O2H+
Citria
Glen Ericht
Himbotop
Kweli
Paris
Tulameen
0.00
0.05
0.10
0.15
Concentration, ppbV
P
R
OR
Type
stage
P
R
OR

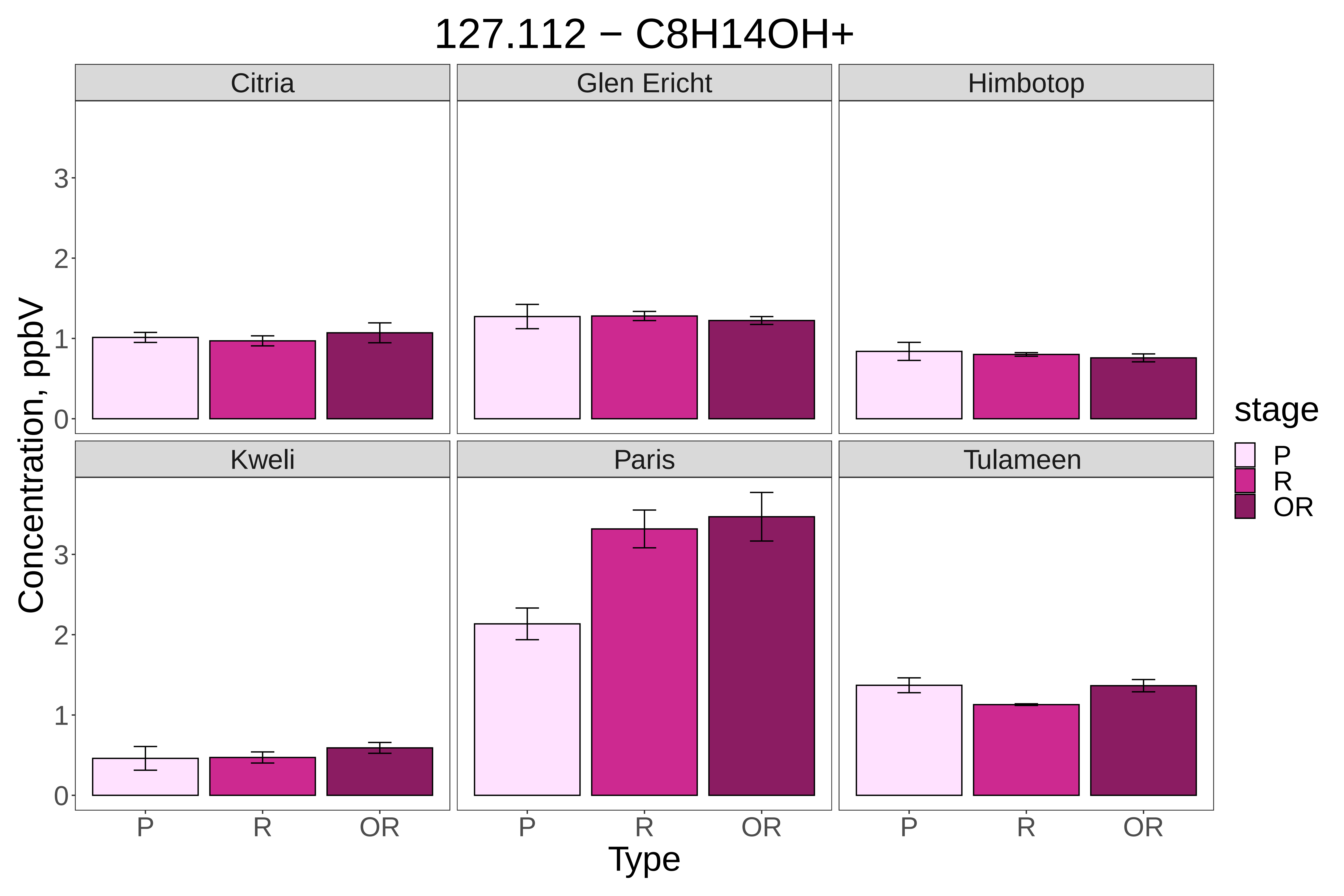
127.112 − C8H14OH+
Citria
Glen Ericht
Himbotop
Kweli
Paris
Tulameen
Concentration, ppbV
0
1
2
3
P
R
OR
Type
stage
P
R
OR

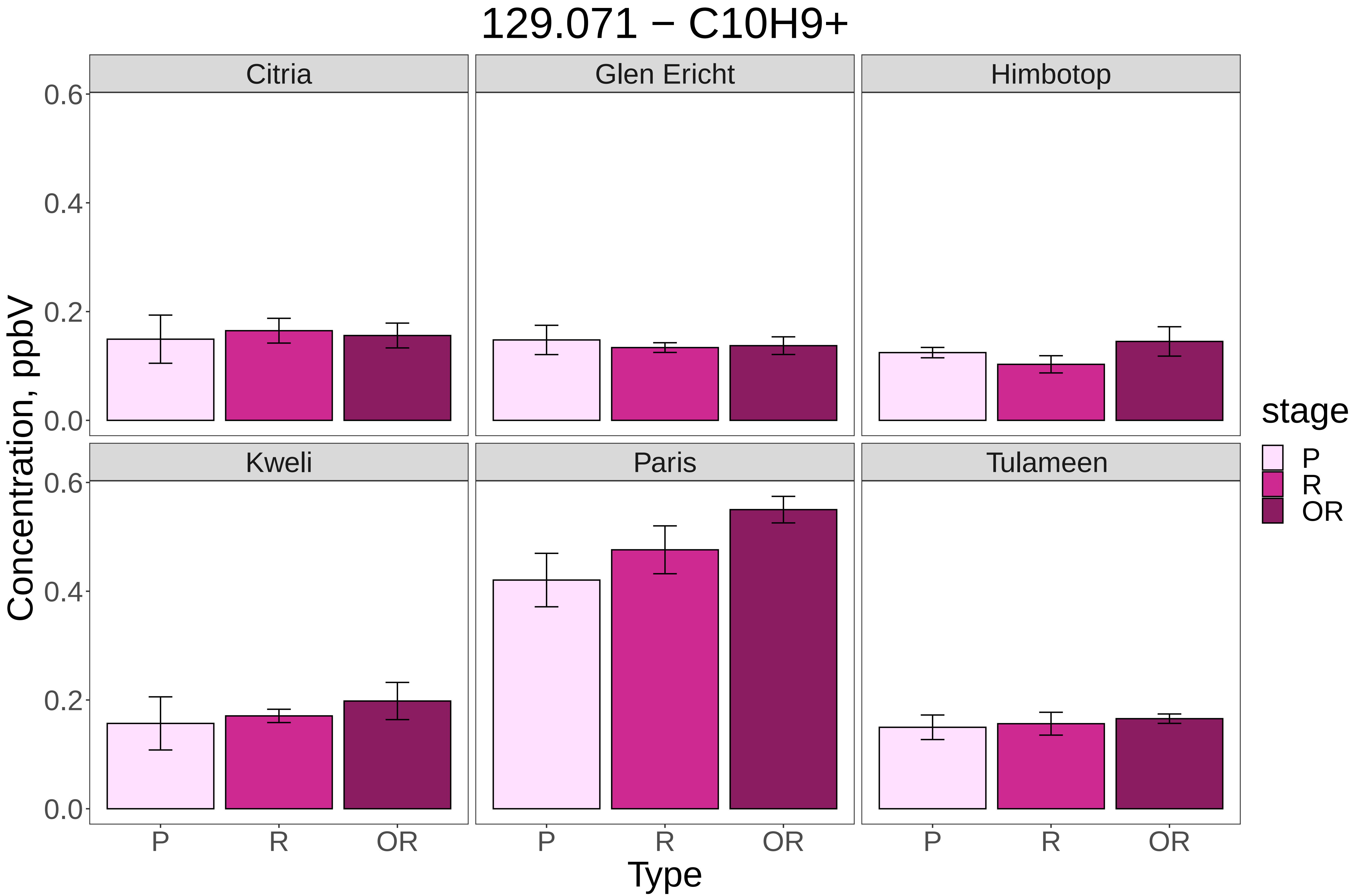

129.071 − C10H9+
Citria
Glen Ericht
Himbotop
Kweli
Paris
Tulameen
Concentration, ppbV
0.0
0.2
0.4
0.6
P
R
OR
Type
stage
P
R
OR

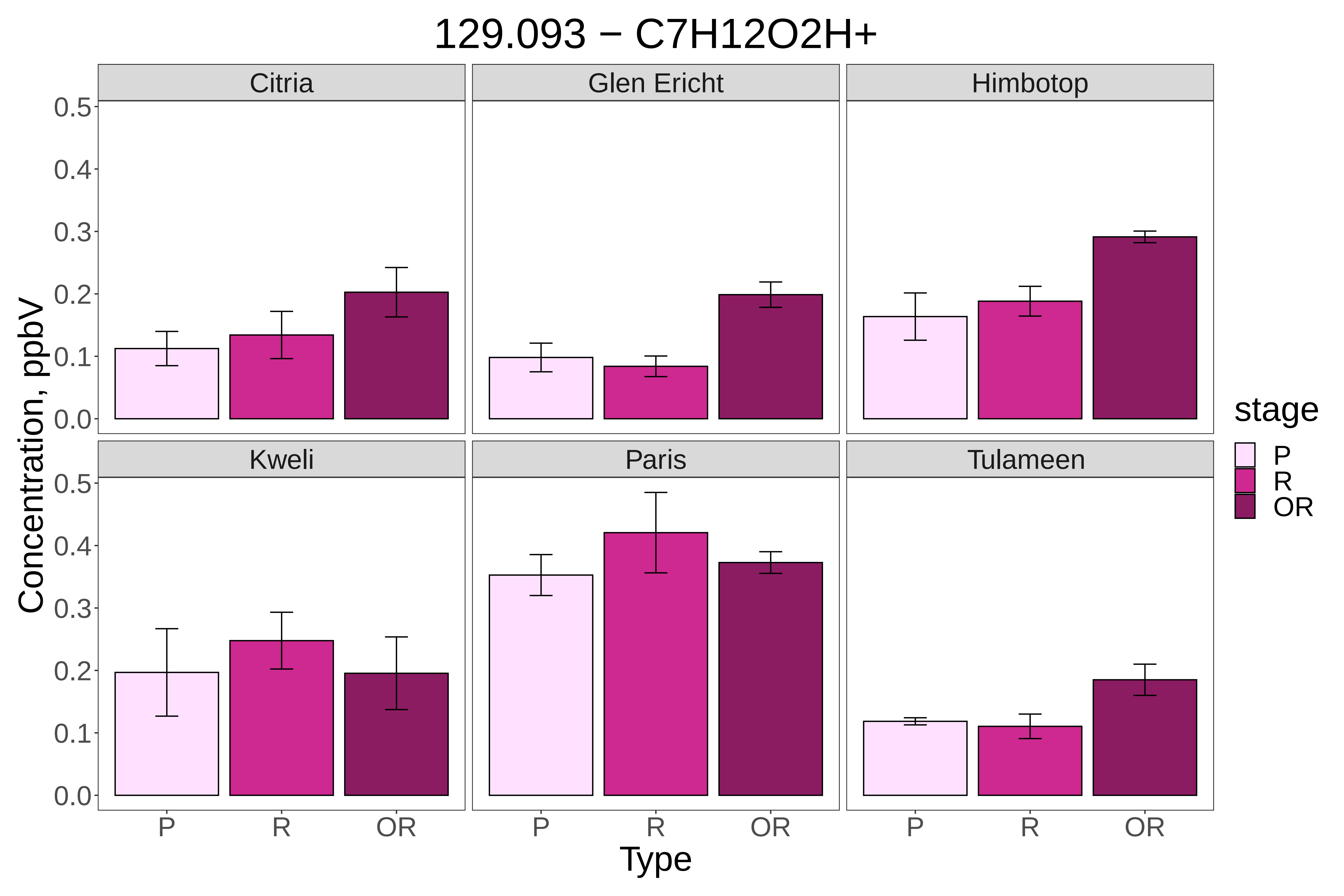

129.093 − C7H12O2H+
Citria
Glen Ericht
Himbotop
Kweli
Paris
Tulameen
Concentration, ppbV
0.0
0.1
0.2
0.3
0.4
0.5
P
R
OR
Type
stage
P
R
OR

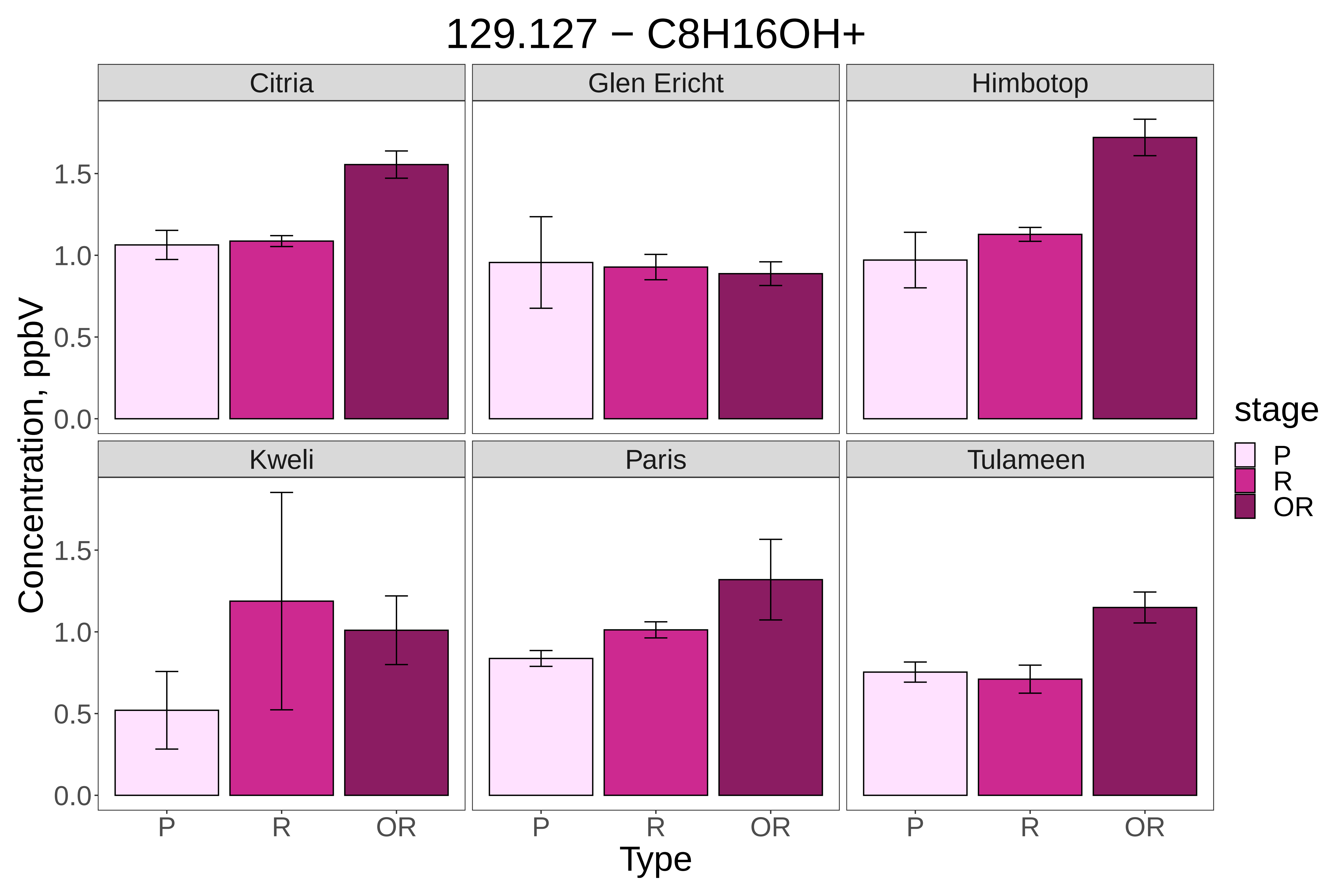
129.127 − C8H16OH+
Citria
Glen Ericht
Himbotop
Kweli
Paris
Tulameen
Concentration, ppbV
0.0
0.5
1.0
1.5
P
R
OR
Type
stage
P
R
OR

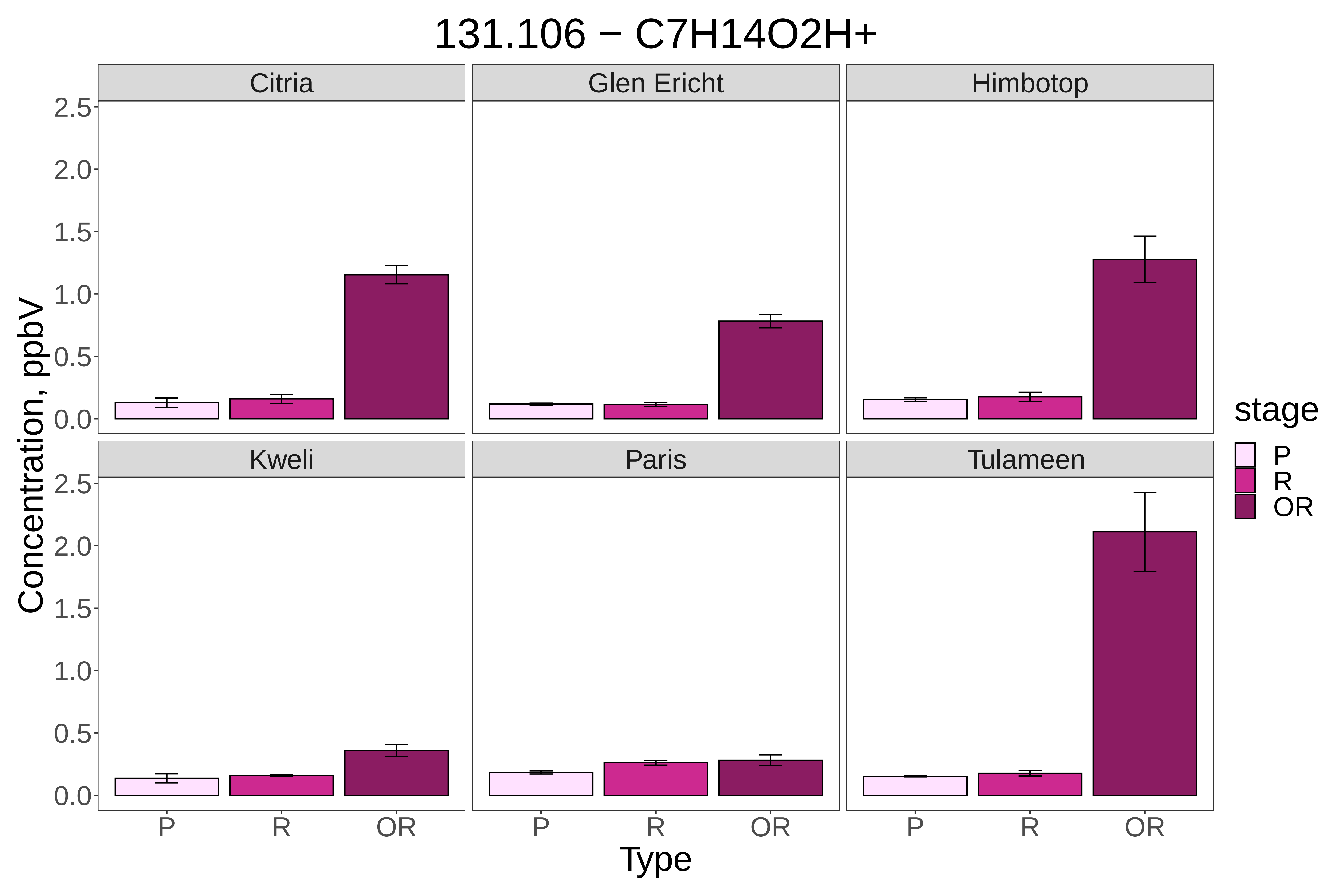
131.106 − C7H14O2H+
Citria
Glen Ericht
Himbotop
Kweli
Paris
Tulameen
Concentration, ppbV
0.0
0.5
1.0
1.5
2.0
2.5
P
R
OR
Type
stage
P
R
OR

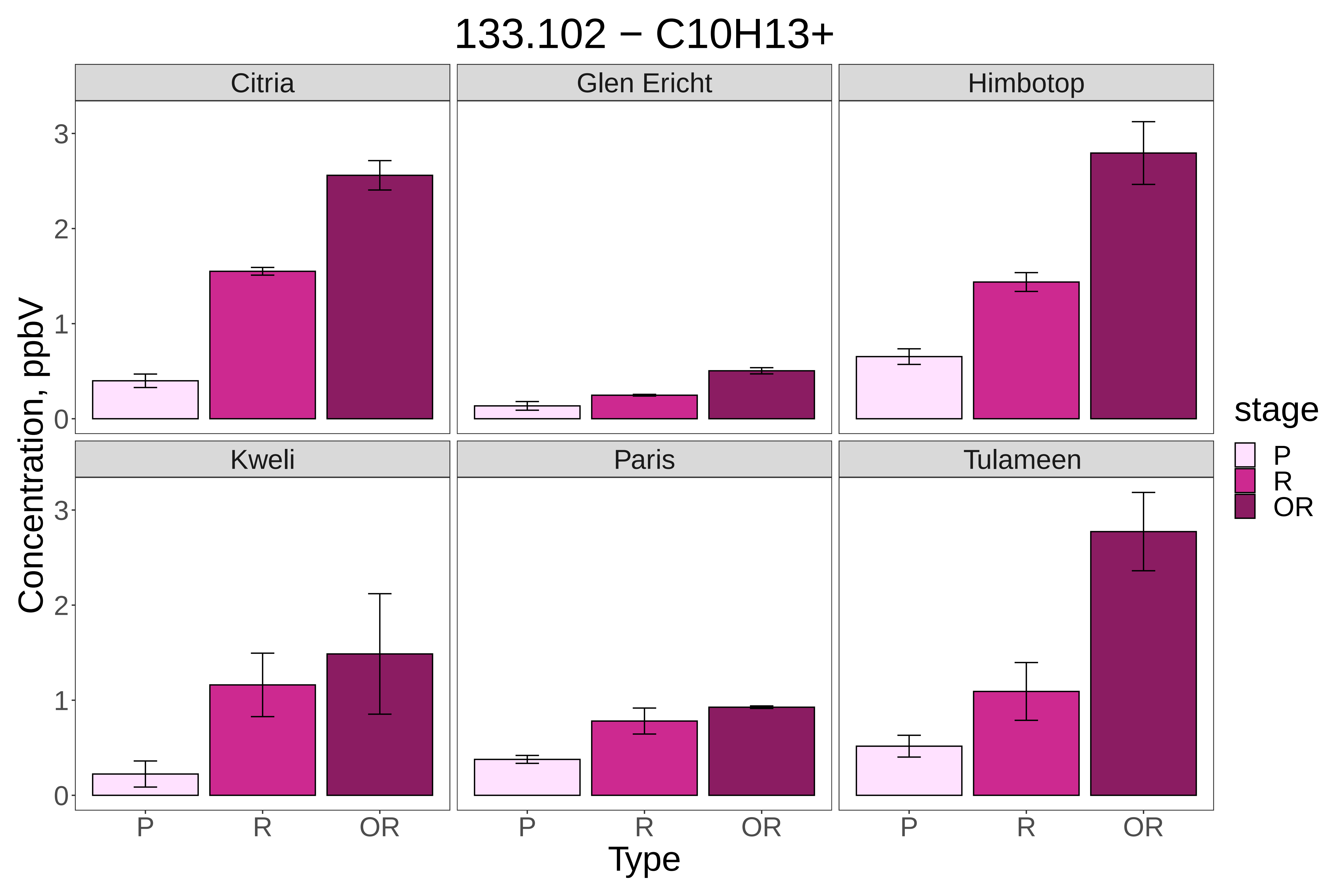

133.102 − C10H13+
Citria
Glen Ericht
Himbotop
Kweli
Paris
Tulameen
Concentration, ppbV
0
1
2
3
P
R
OR
Type
stage
P
R
OR

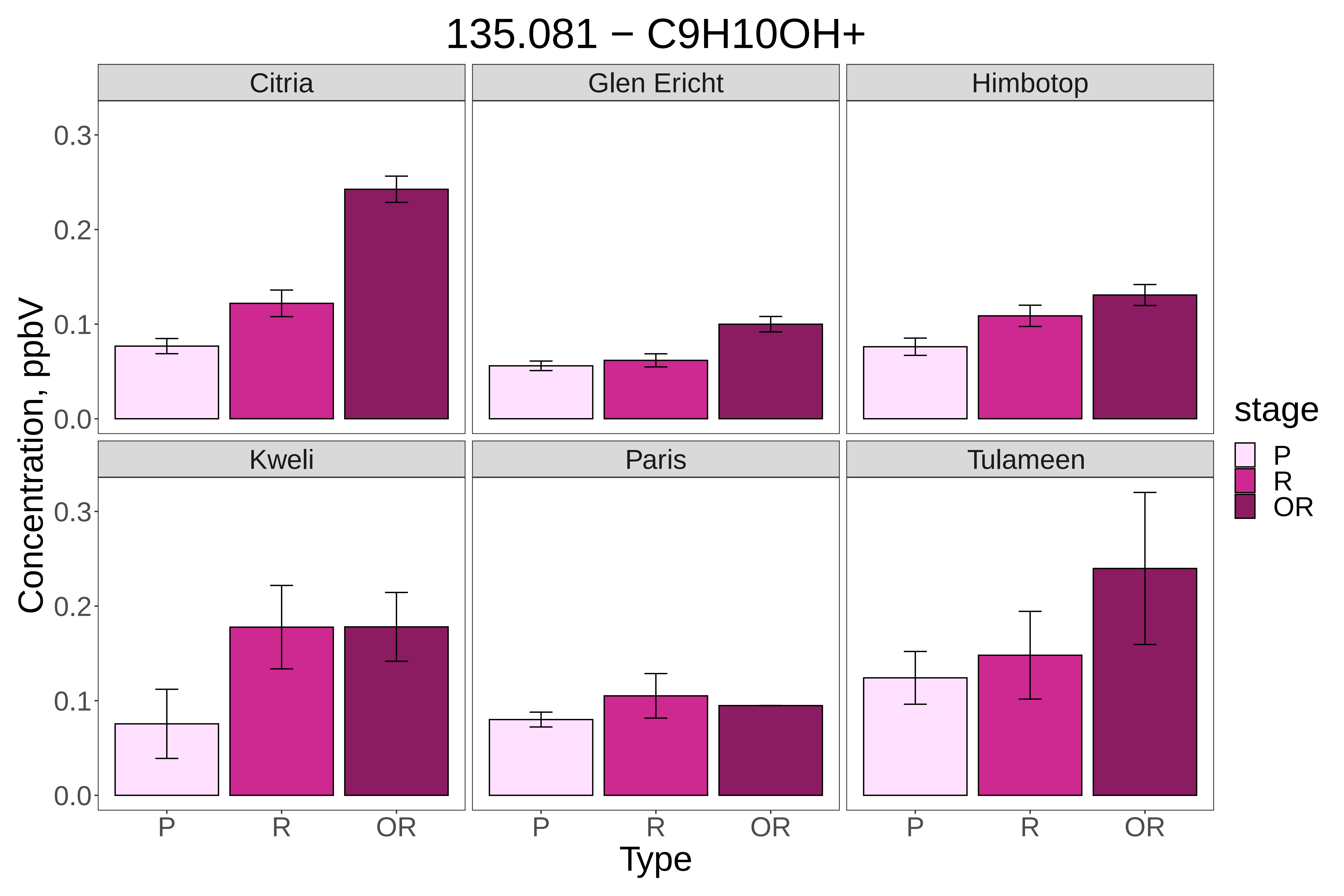

135.081 − C9H10OH+
Citria
Glen Ericht
Himbotop
Kweli
Paris
Tulameen
0.0
0.1
0.2
0.3
Concentration, ppbV
P
R
OR
Type
stage
P
R
OR

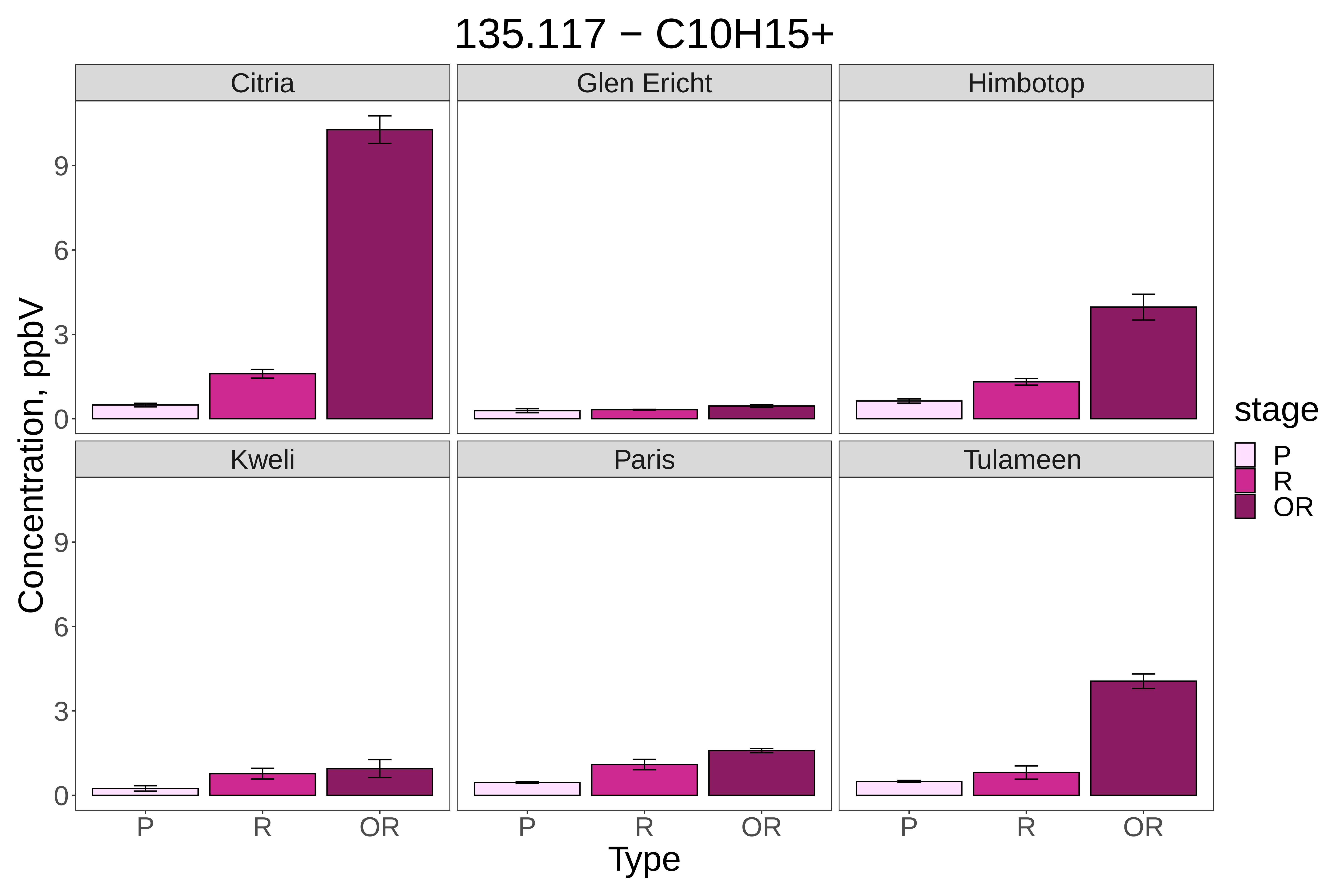

135.117 − C10H15+
Citria
Glen Ericht
Himbotop
Kweli
Paris
Tulameen
Concentration, ppbV
0
3
6
9
P
R
OR
Type
stage
P
R
OR

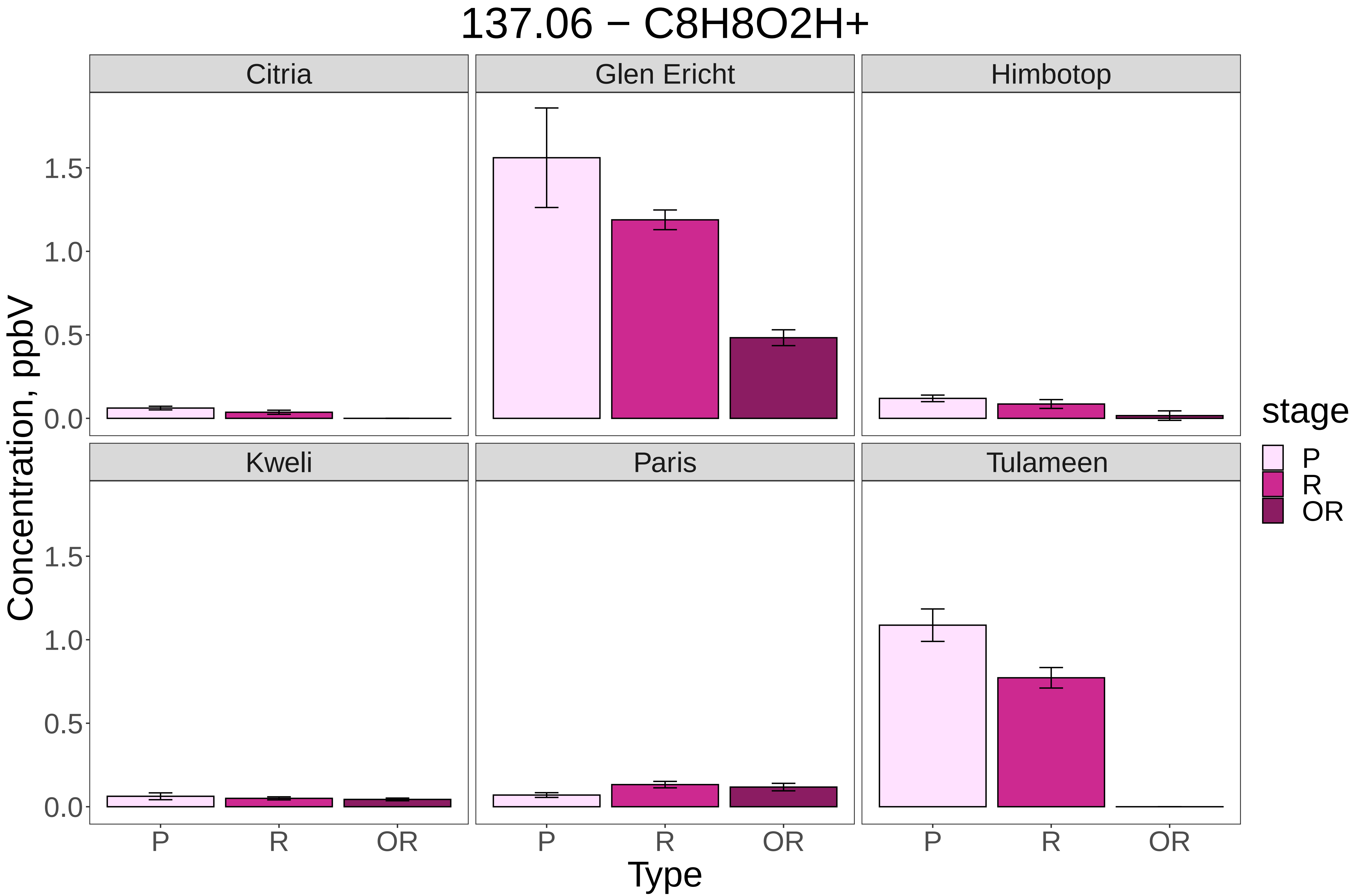

137.06 − C8H8O2H+
Citria
Glen Ericht
Himbotop
Kweli
Paris
Tulameen
Concentration, ppbV
0.0
0.5
1.0
1.5
Type
P
R
OR
stage
P
R
OR

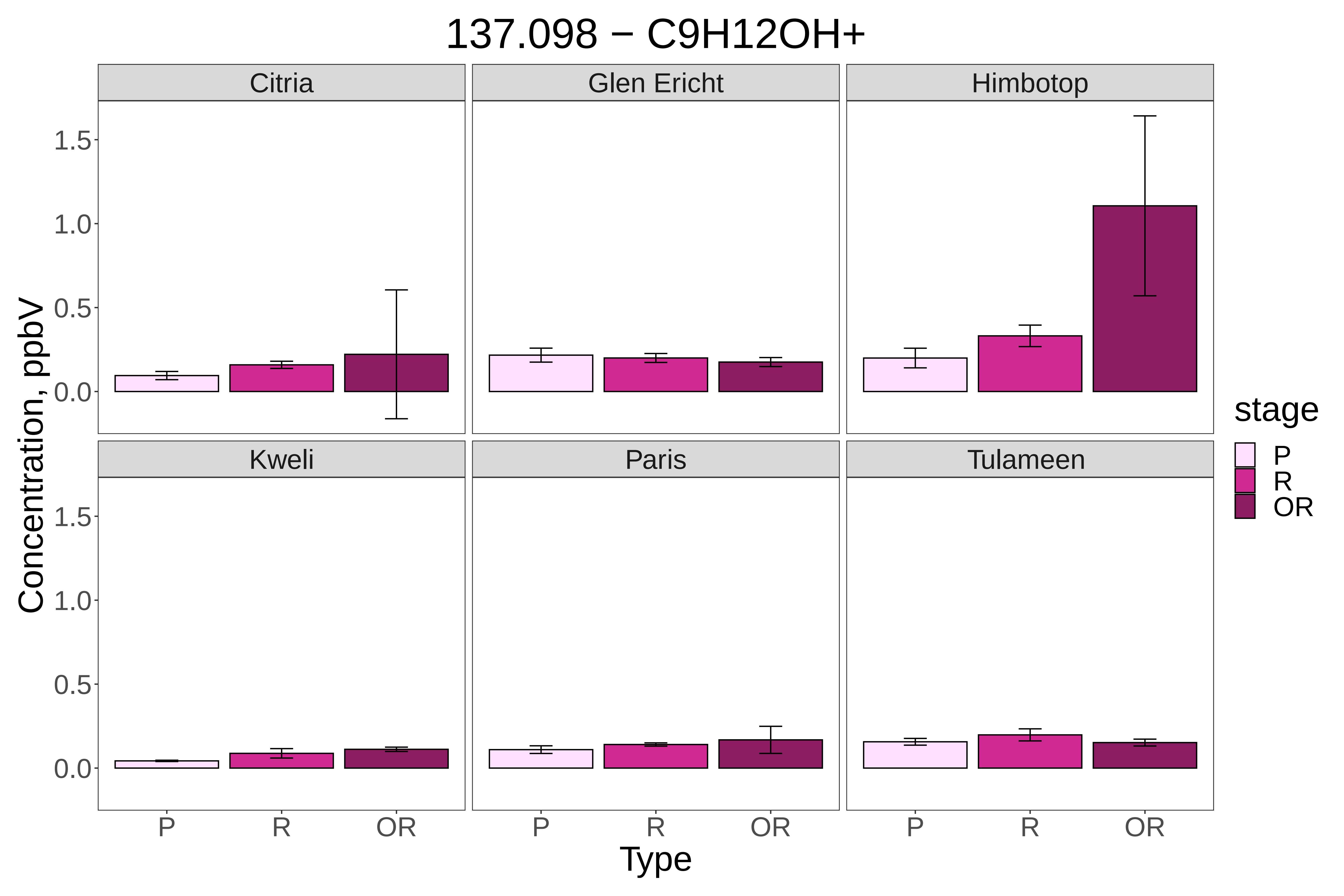

137.098 − C9H12OH+
Citria
Glen Ericht
Himbotop
Kweli
Paris
Tulameen
Concentration, ppbV
1.5
1.0
0.5
0.0
P
R
OR
Type
stage
P
R
OR

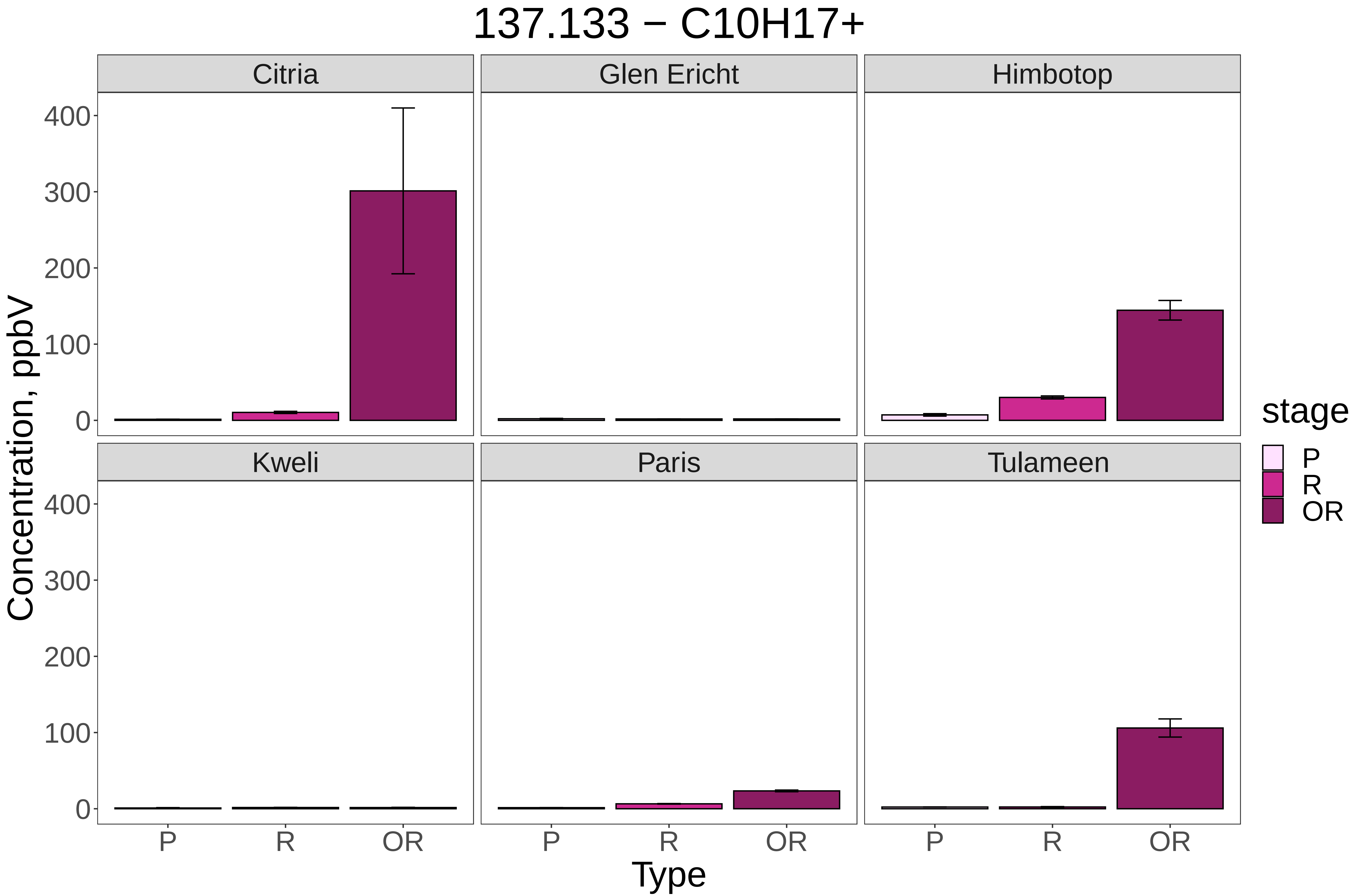

137.133 − C10H17+
Citria
Glen Ericht
Himbotop
Kweli
Paris
Tulameen
400
300
200
100
0
Concentration, ppbV
P
R
OR
Type
stage
P
R
OR

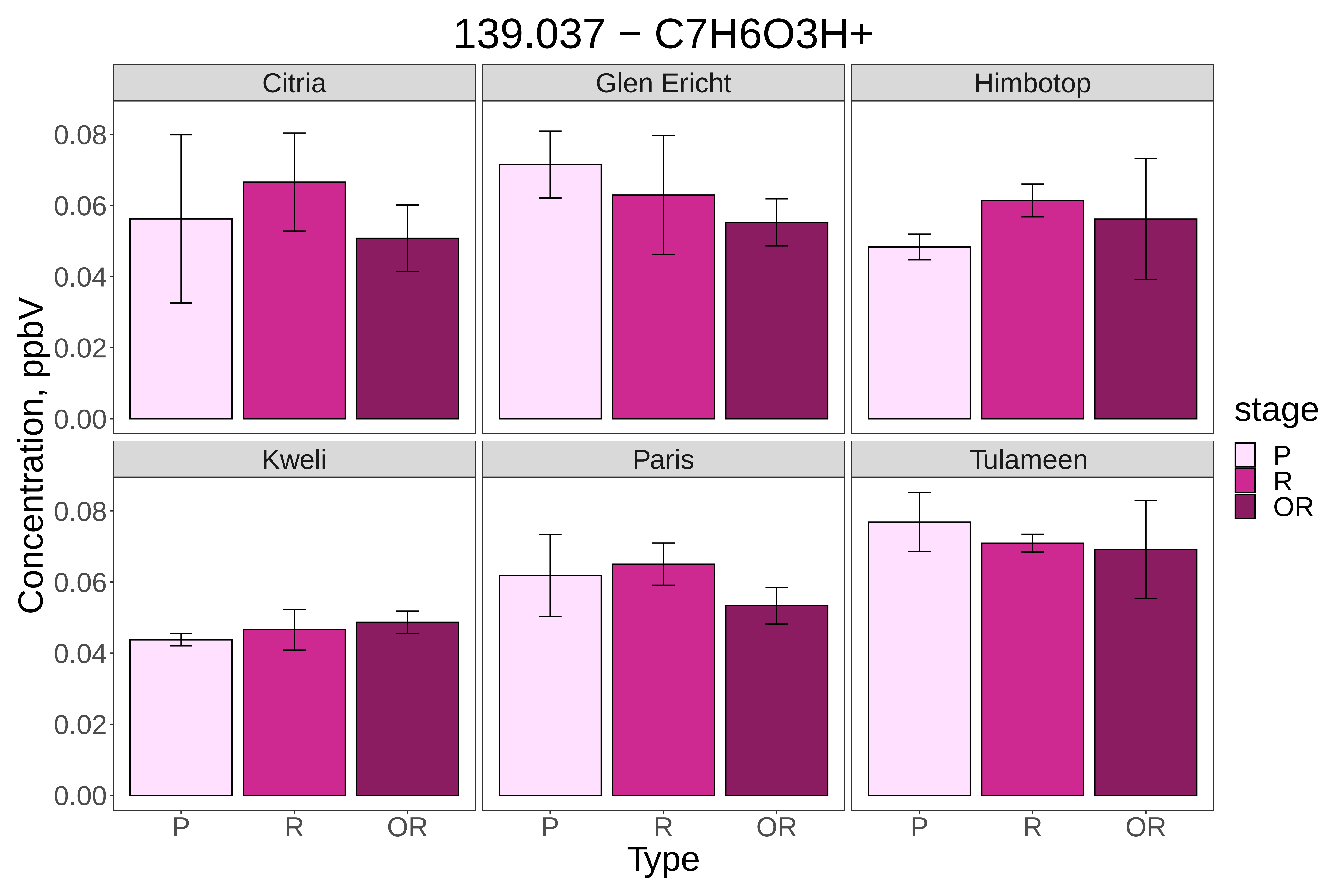
139.037 − C7H6O3H+
Citria
Glen Ericht
Himbotop
Kweli
Paris
Tulameen
Concentration, ppbV
0.00
0.02
0.04
0.06
0.08
P
R
OR
Type
stage
P
R
OR

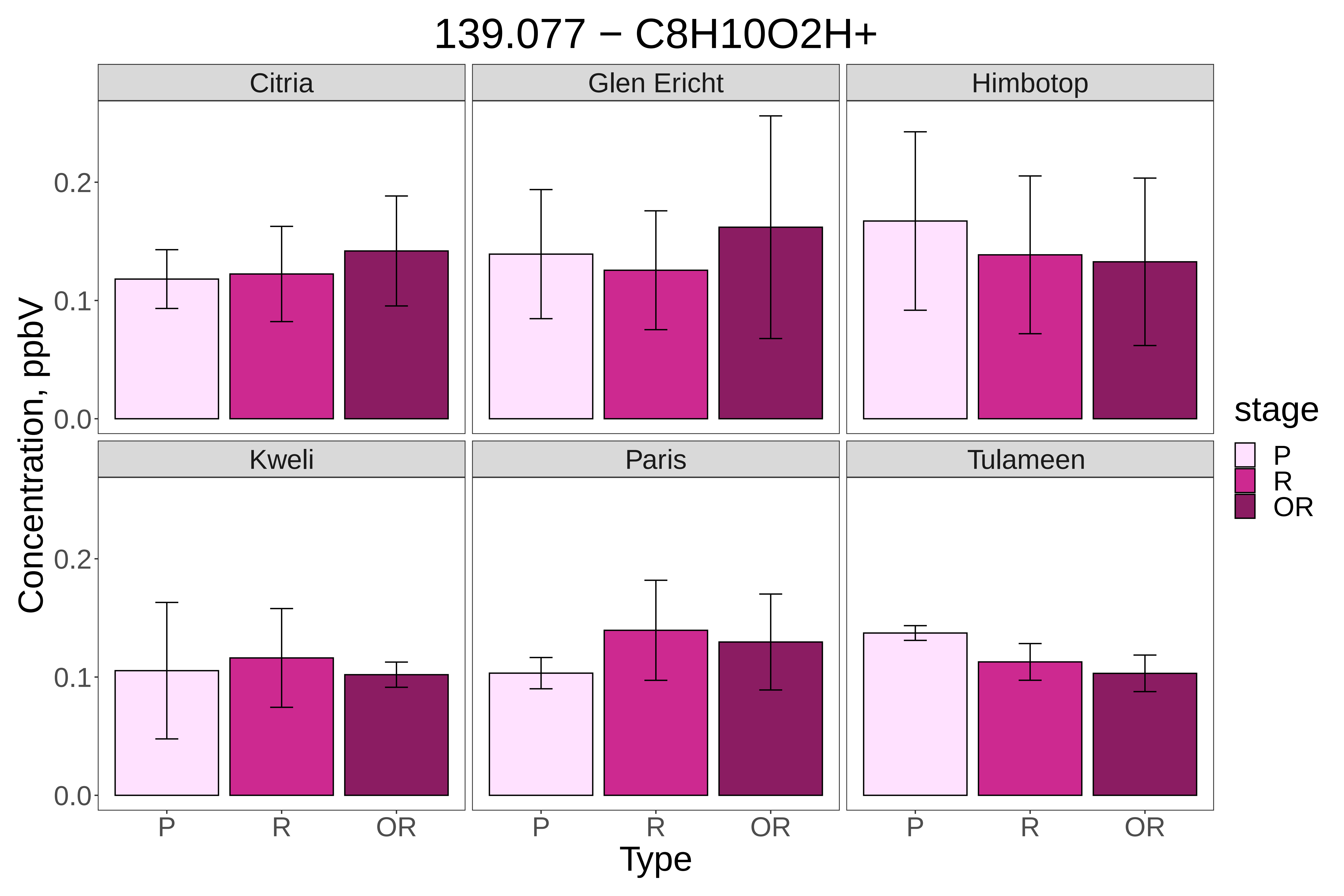
139.077 − C8H10O2H+
Citria
Glen Ericht
Himbotop
Kweli
Paris
Tulameen
Concentration, ppbV
0.0
0.1
0.2
Type
P
R
OR
stage
P
R
OR

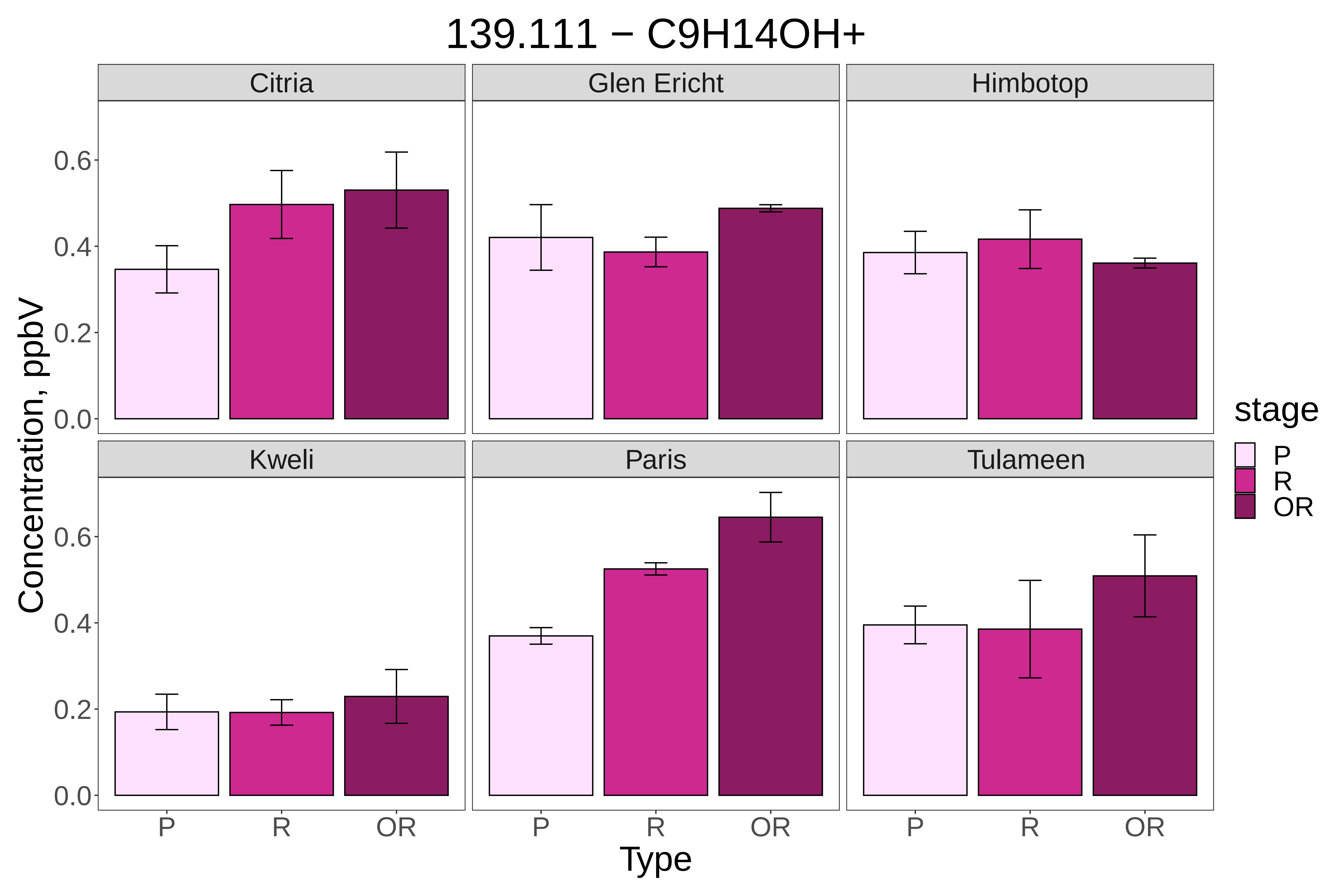

139.111 − C9H14OH+
Citria
Glen Ericht
Himbotop
Kweli
Paris
Tulameen
Concentration, ppbV
0.0
0.2
0.4
0.6
P
R
OR
Type
stage
P
R
OR

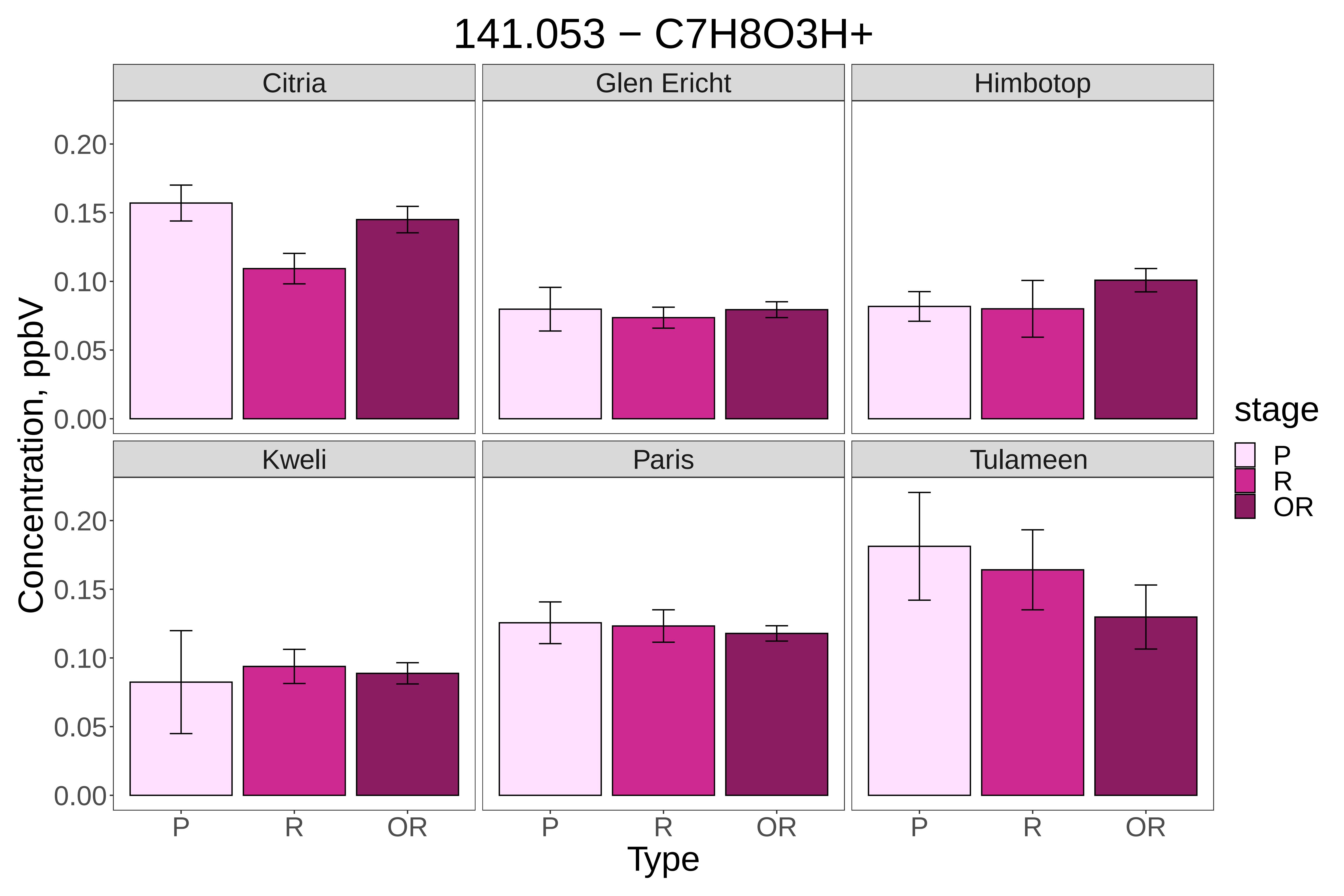

141.053 − C7H8O3H+
Citria
Glen Ericht
Himbotop
Kweli
Paris
Tulameen
Concentration, ppbV
0.00
0.05
0.10
0.15
0.20
P
R
OR
Type
stage
P
R
OR

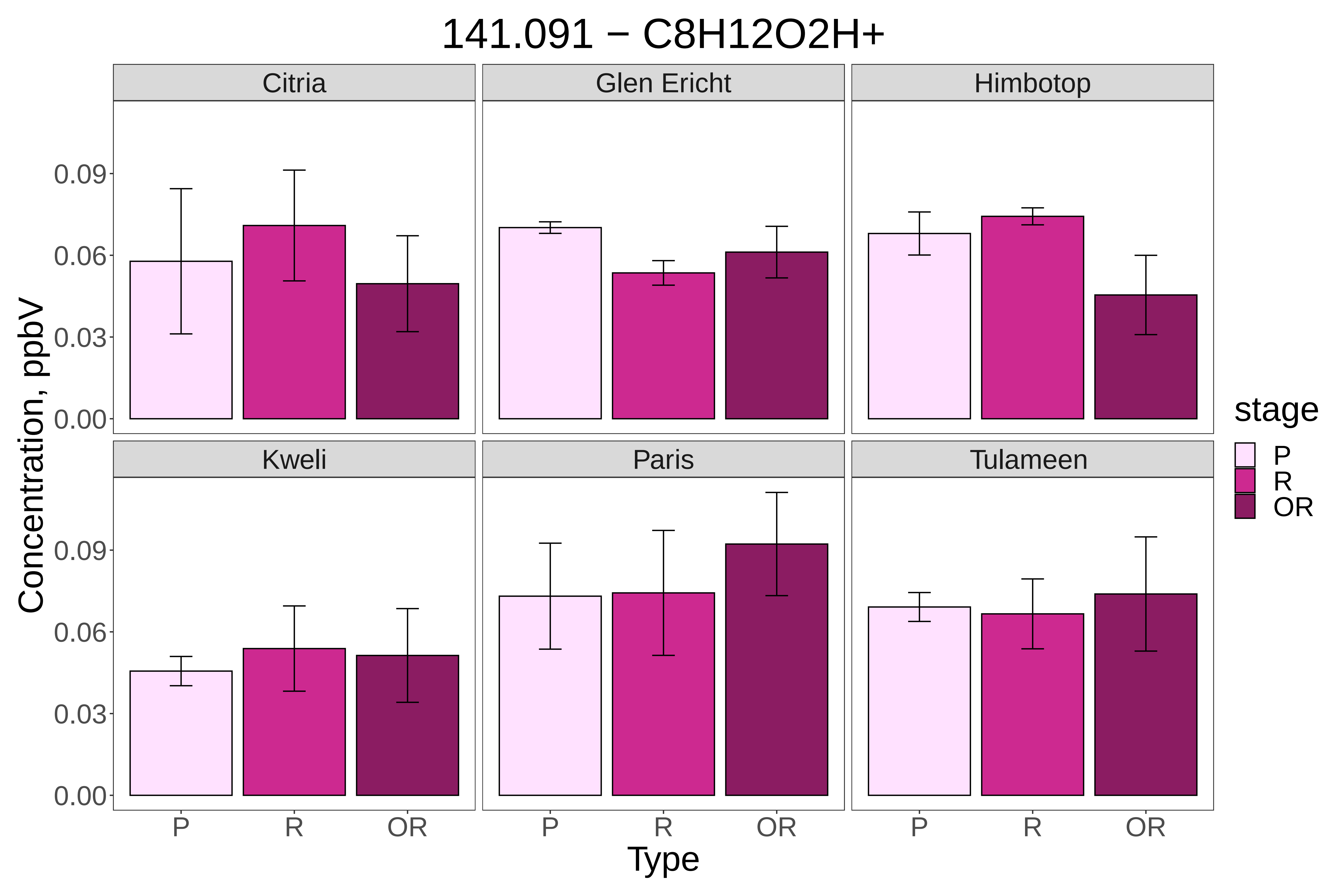

141.091 − C8H12O2H+
Citria
Glen Ericht
Himbotop
Kweli
Paris
Tulameen
Concentration, ppbV
0.00
0.03
0.06
0.09
P
R
OR
Type
stage

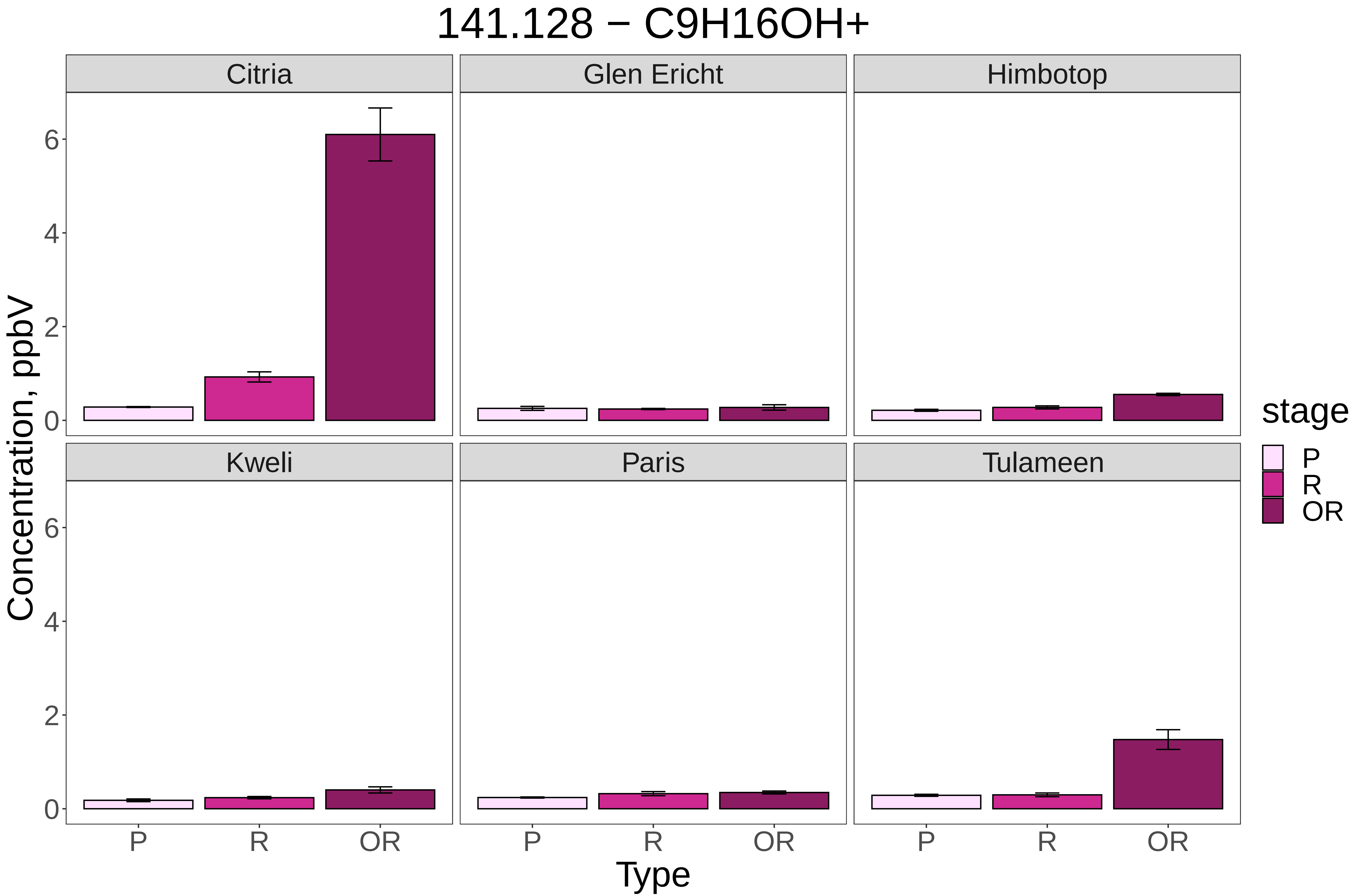
141.128 − C9H16OH+
Citria
Glen Ericht
Himbotop
Kweli
Paris
Tulameen
Concentration, ppbV
0
2
4
6
P
R
OR
Type
stage
P
R
OR

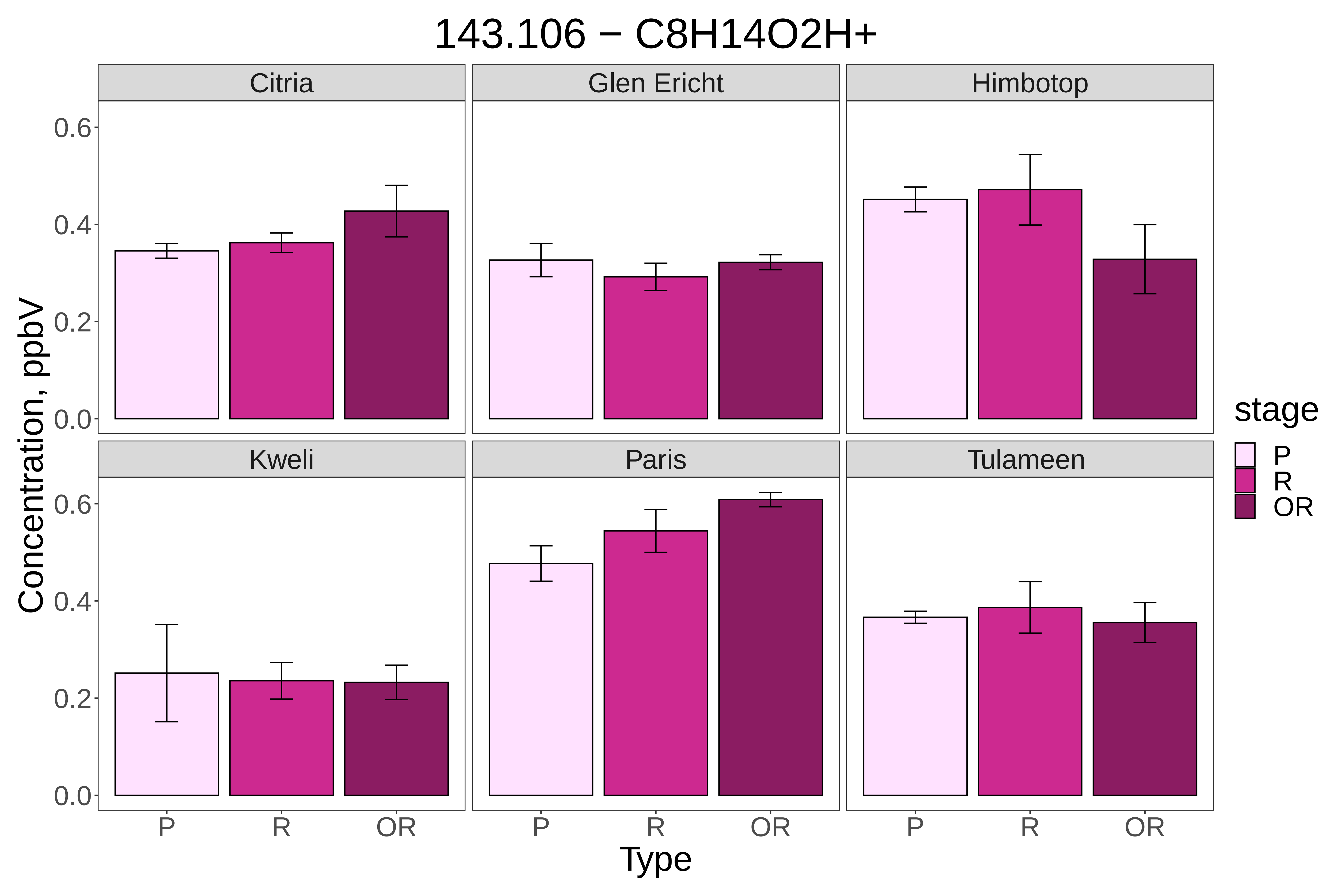
143.106 − C8H14O2H+
Citria
Glen Ericht
Himbotop
Kweli
Paris
Tulameen
Concentration, ppbV
0.0
0.2
0.4
0.6
Type
P
R
OR
stage
P
R
OR

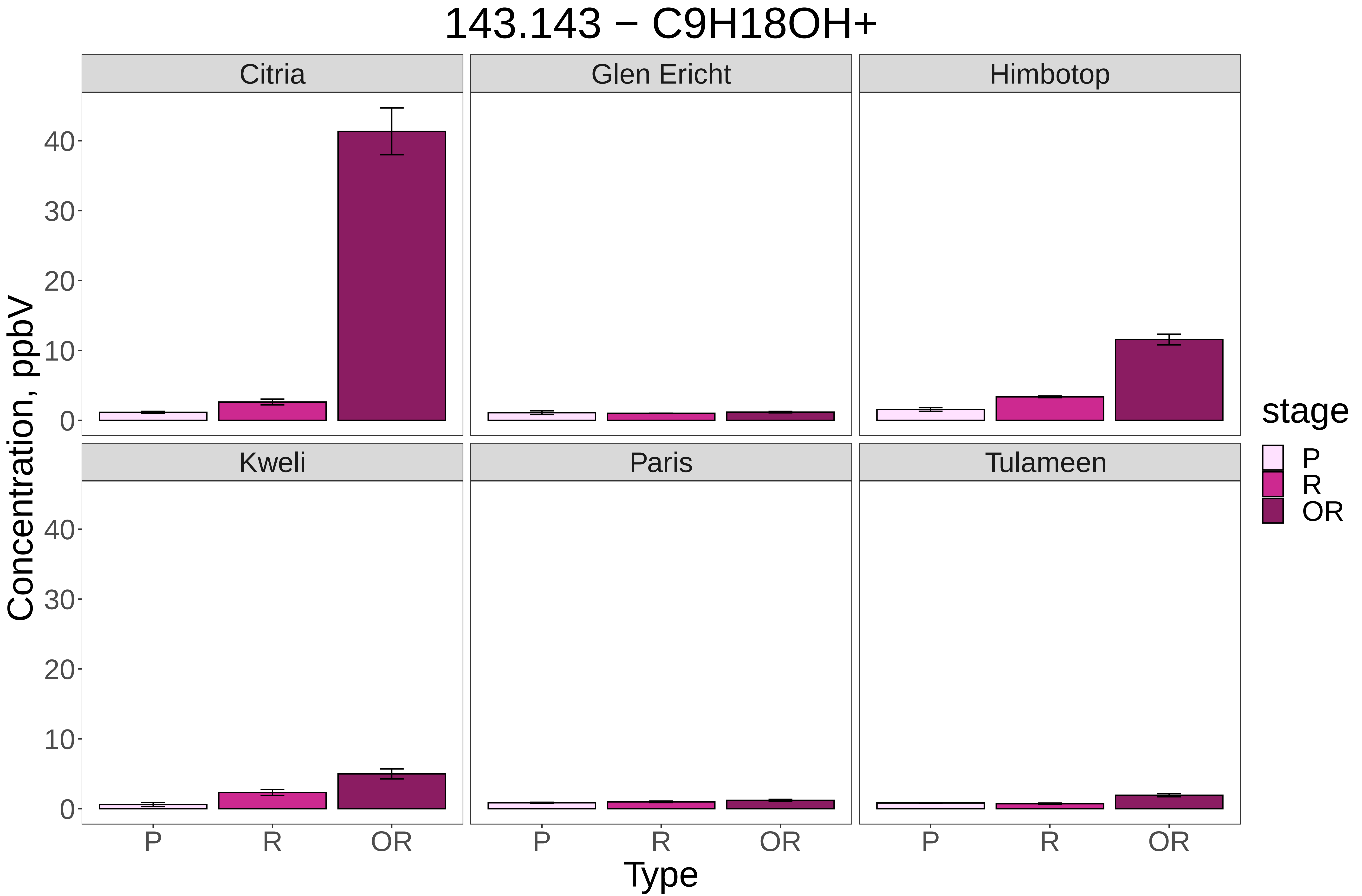

143.143 − C9H18OH+
Citria
Glen Ericht
Himbotop
Kweli
Paris
Tulameen
Concentration, ppbV
0
10
20
30
40
P
R
OR
Type
stage
P
R
OR

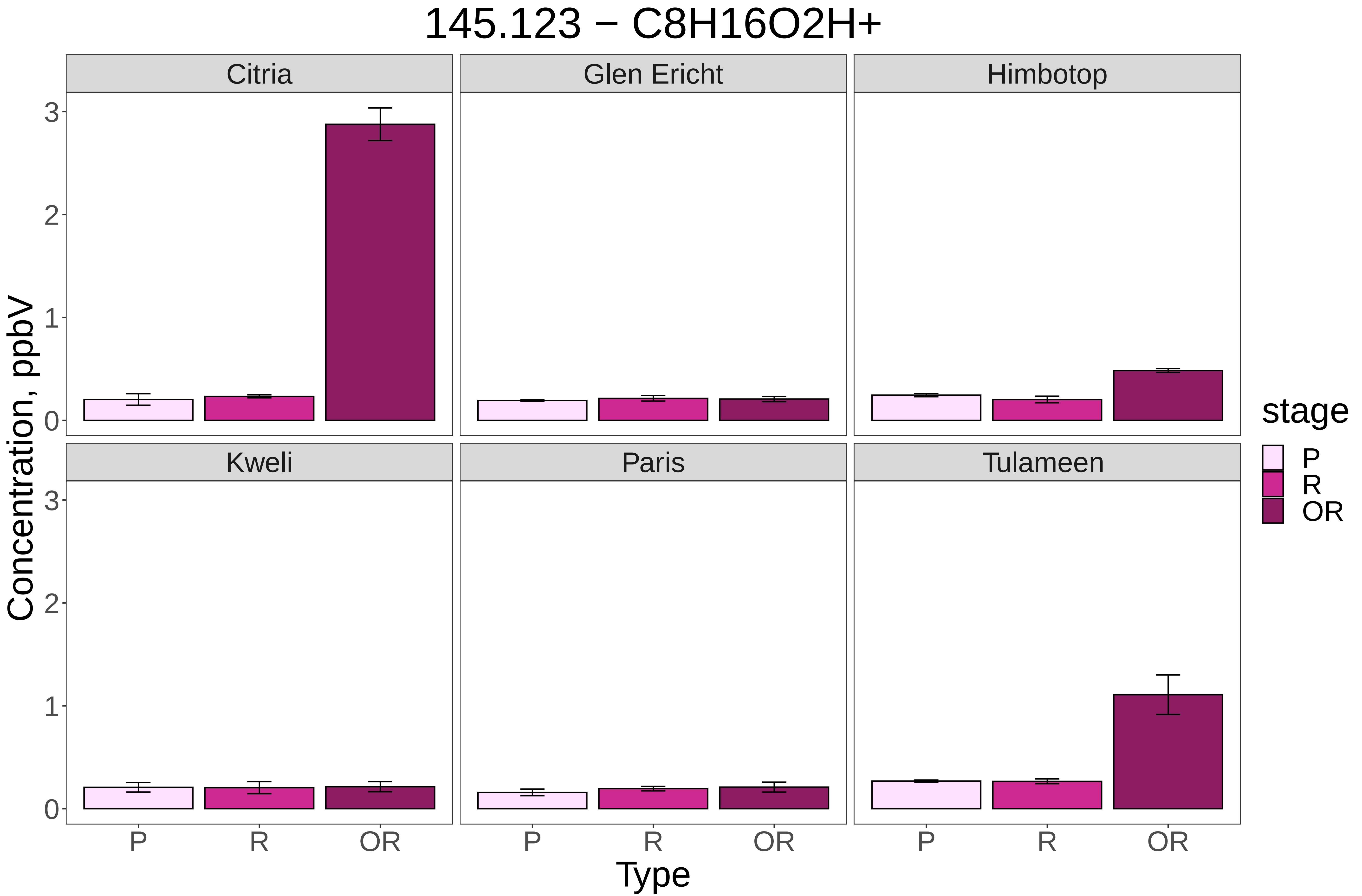
145.123 − C8H16O2H+
Citria
Glen Ericht
Himbotop
Kweli
Paris
Tulameen
Concentration, ppbV
0
1
2
3
P
R
OR
Type
stage
P
R
OR

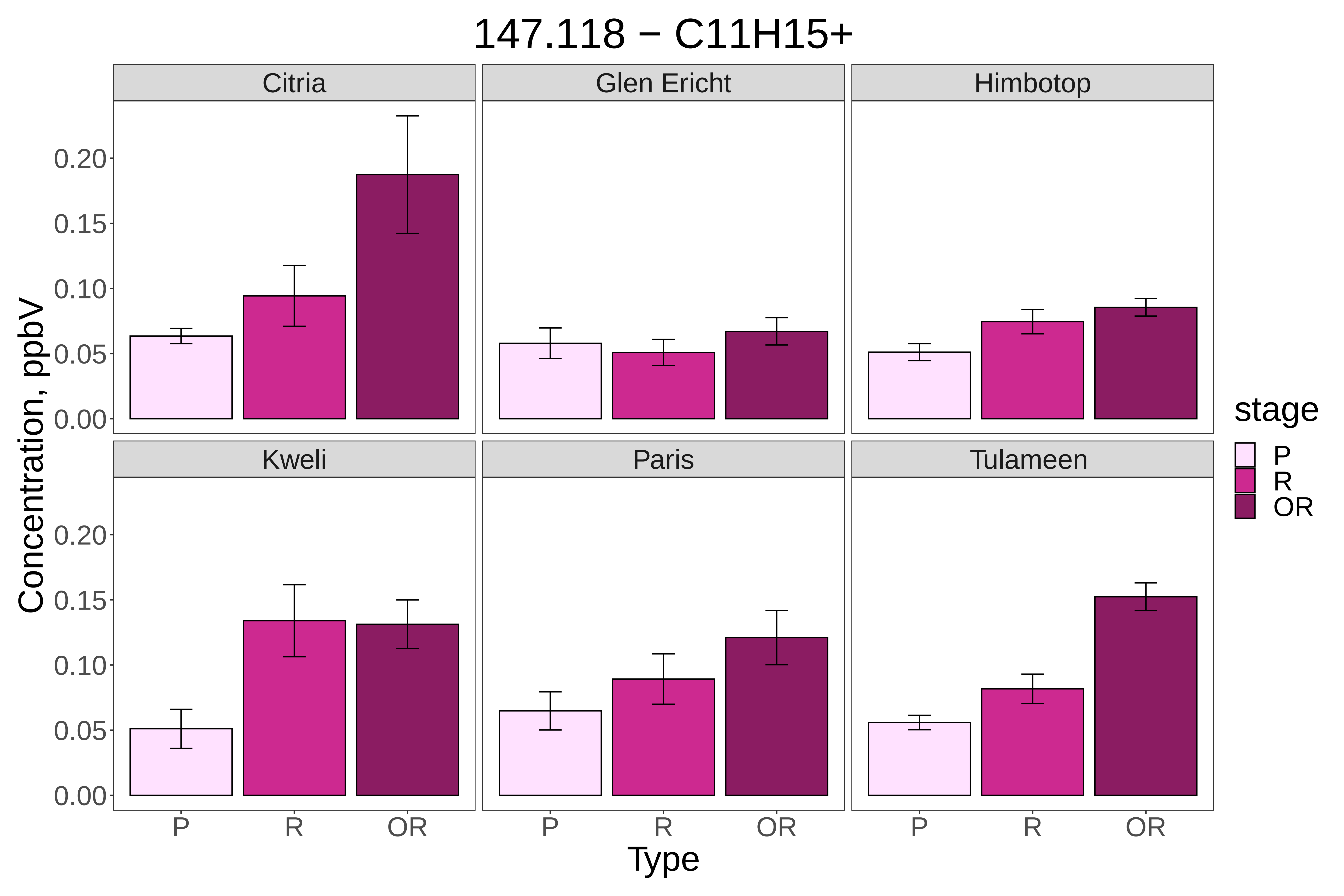

147.118 − C11H15+
Citria
Glen Ericht
Himbotop
Kweli
Paris
Tulameen
Concentration, ppbV
0.00
0.05
0.10
0.15
0.20
P
R
OR
Type
stage
P
R
OR

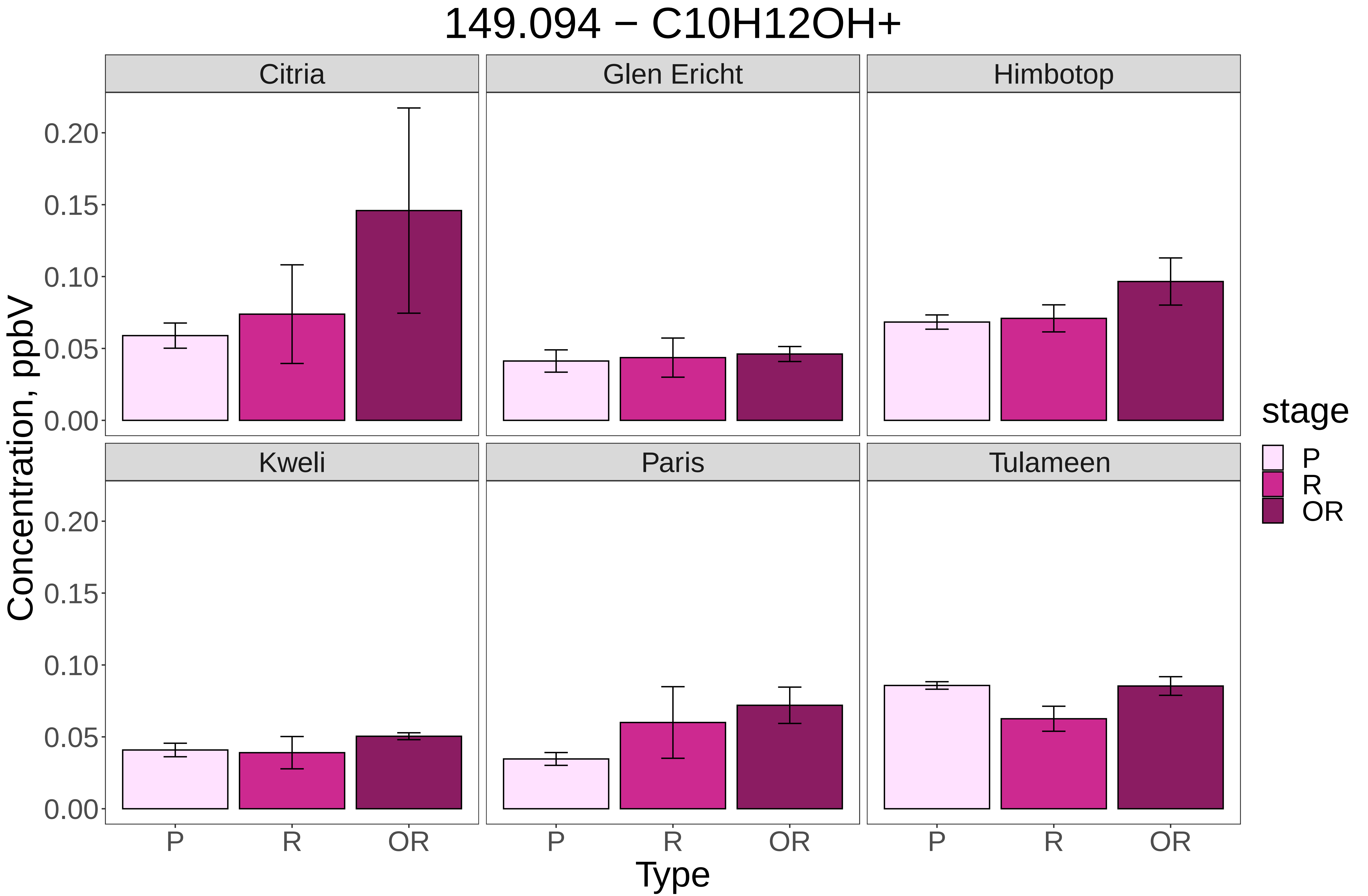

149.094 − C10H12OH+
Citria
Glen Ericht
Himbotop
Kweli
Paris
Tulameen
Concentration, ppbV
0.00
0.05
0.10
0.15
0.20
P
R
OR
Type
stage
P
R
OR

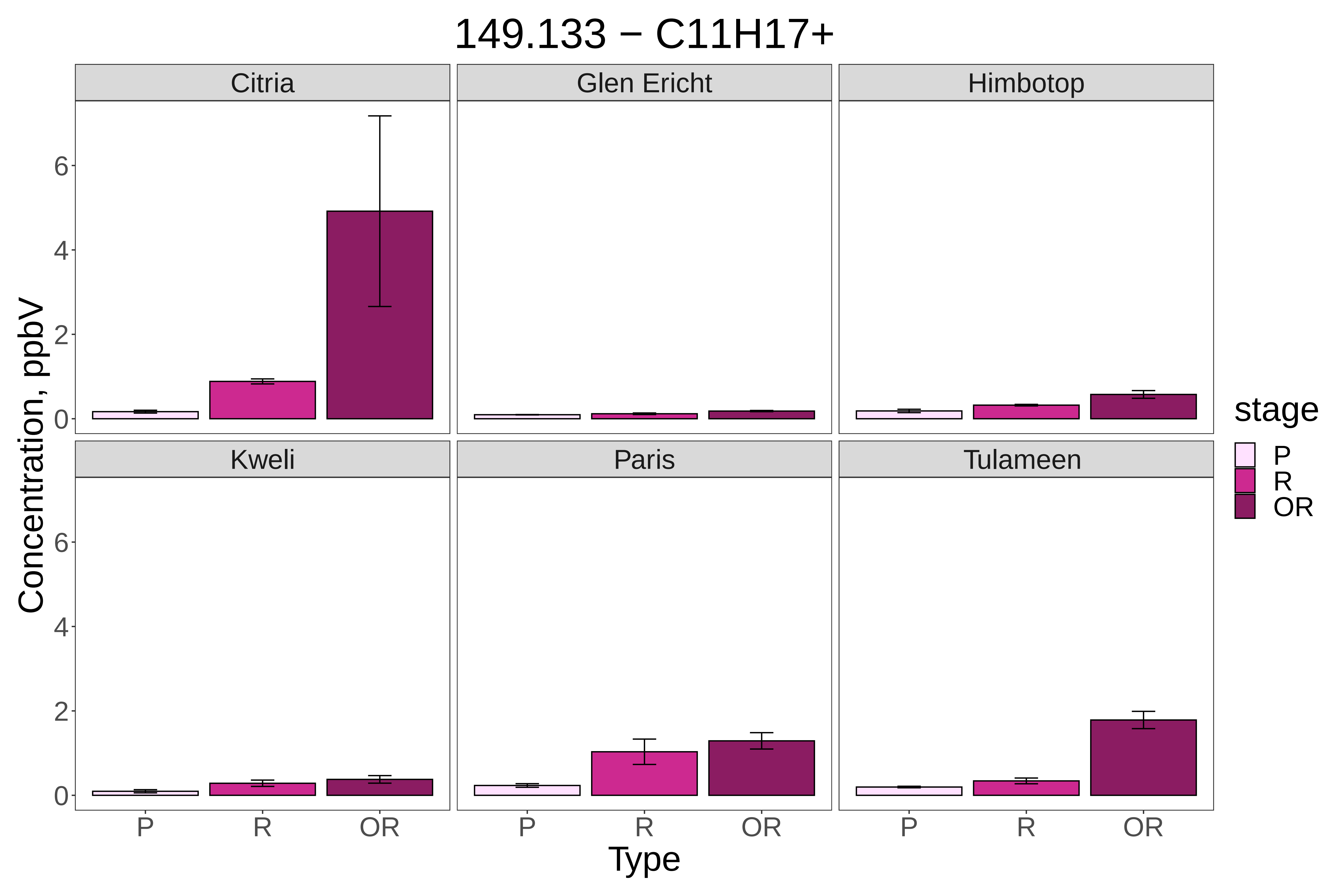

149.133 − C11H17+
Citria
Glen Ericht
Himbotop
Kweli
Paris
Tulameen
Concentration, ppbV
0
2
4
6
P
R
OR
Type
stage
P
R
OR

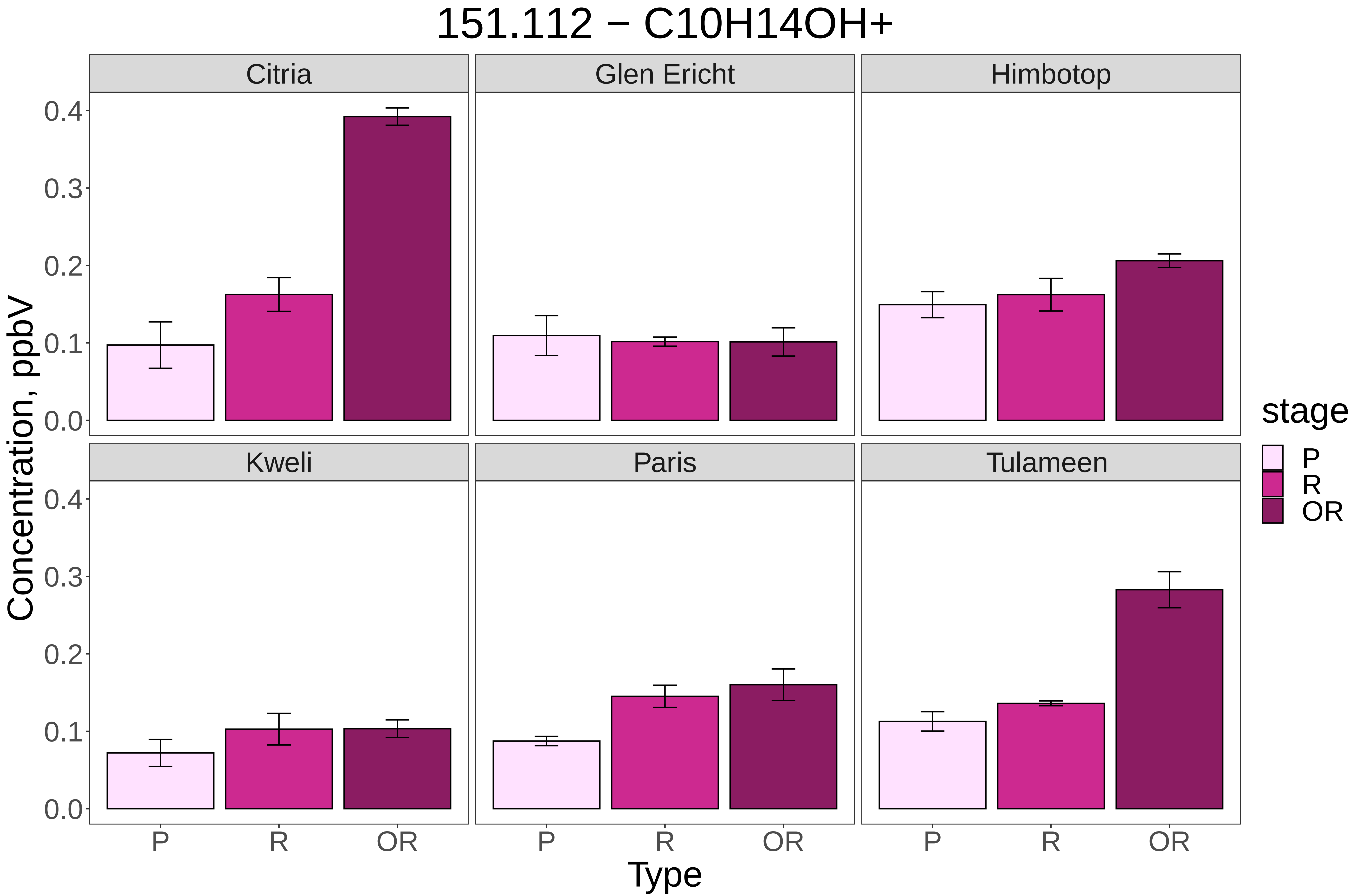
151.112 − C10H14OH+
Citria
Glen Ericht
Himbotop
Kweli
Paris
Tulameen
Concentration, ppbV
0.0
0.1
0.2
0.3
0.4
P
R
OR
Type
stage
P
R
OR

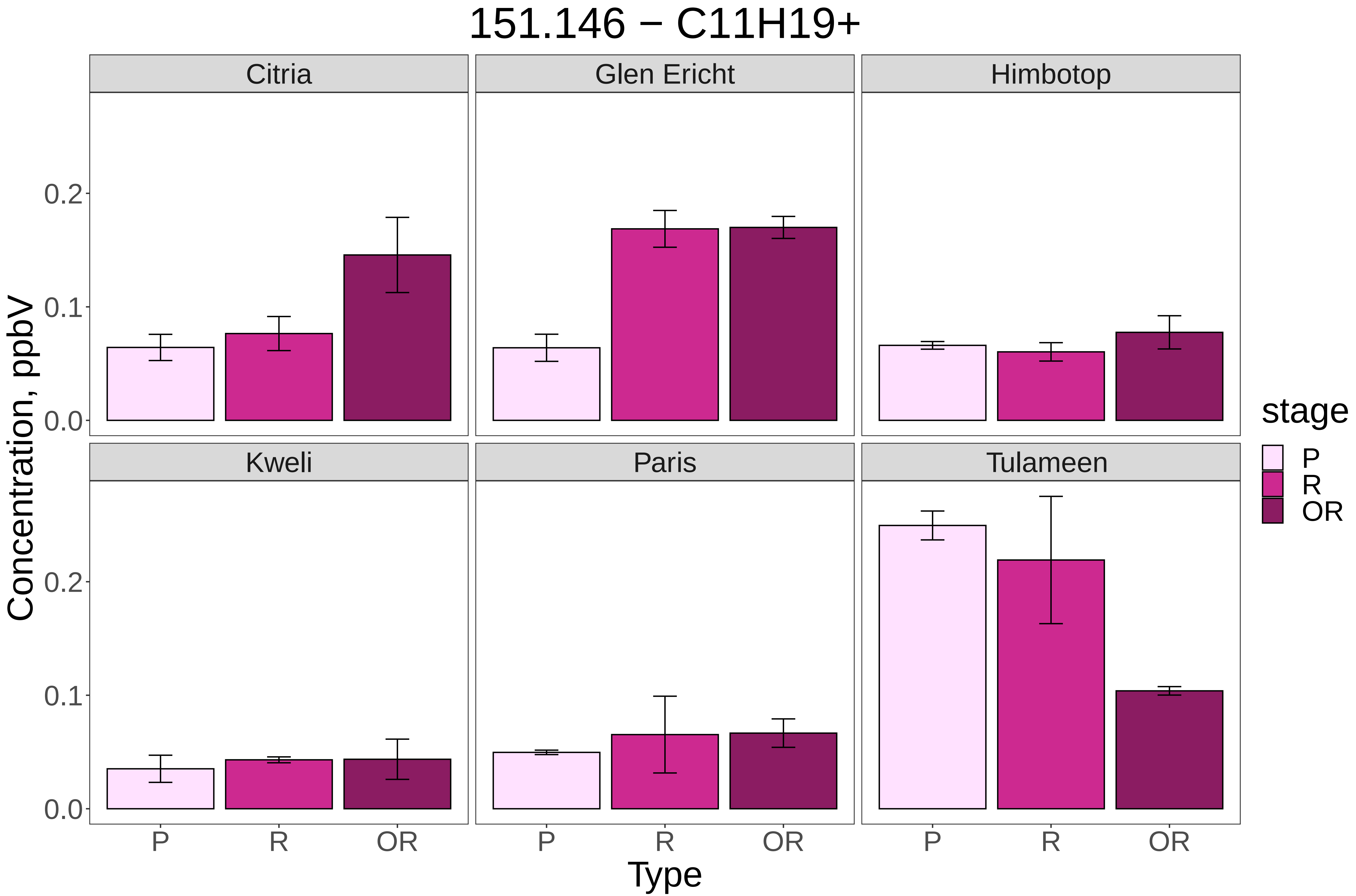

151.146 − C11H19+
Citria
Glen Ericht
Himbotop
Kweli
Paris
Tulameen
Concentration, ppbV
0.0
0.1
0.2
P
R
OR
Type
stage

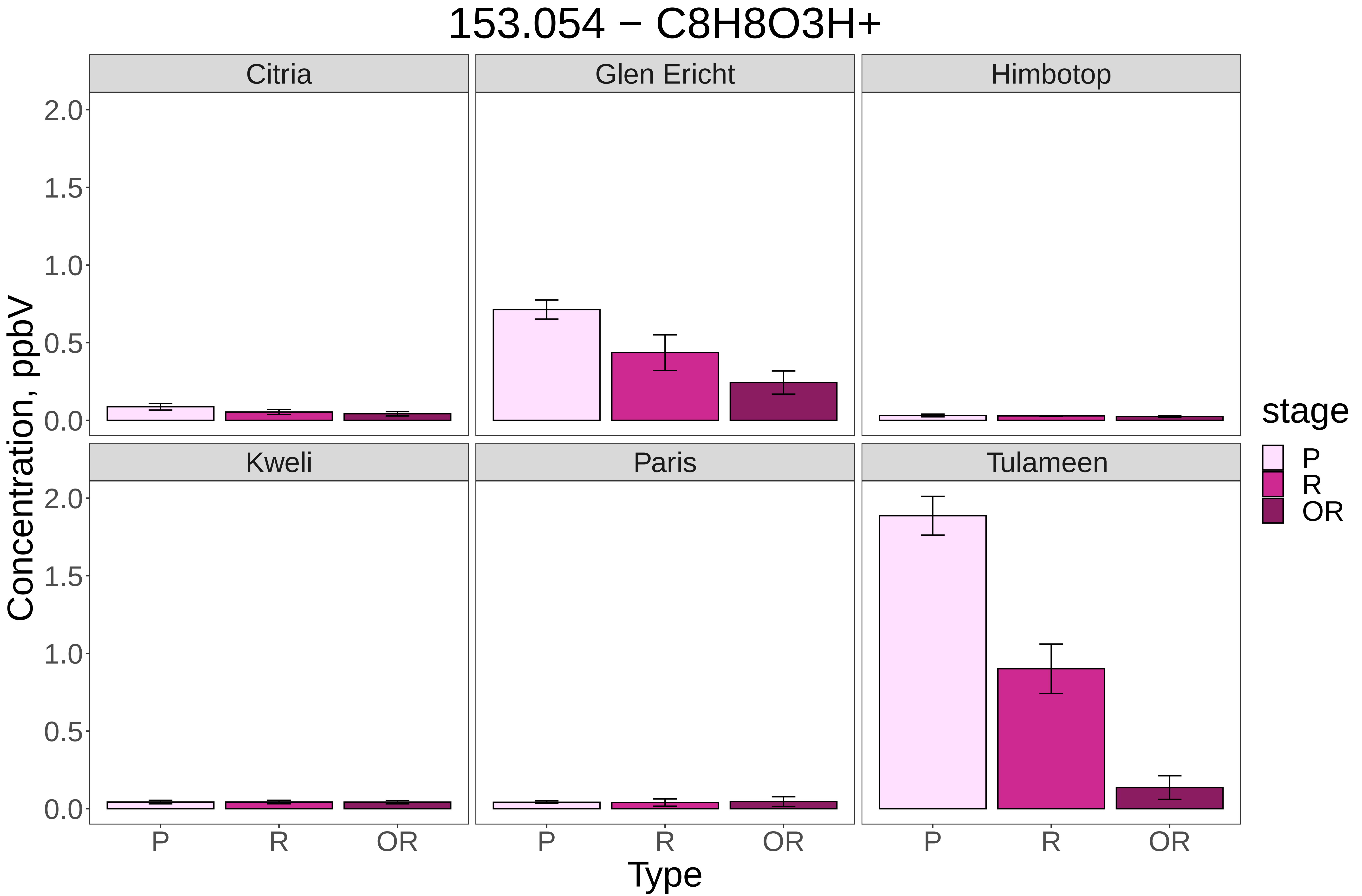

153.054 − C8H8O3H+
Citria
Glen Ericht
Himbotop
Kweli
Paris
Tulameen
Concentration, ppbV
0.0
0.5
1.0
1.5
2.0
P
R
OR
Type
stage

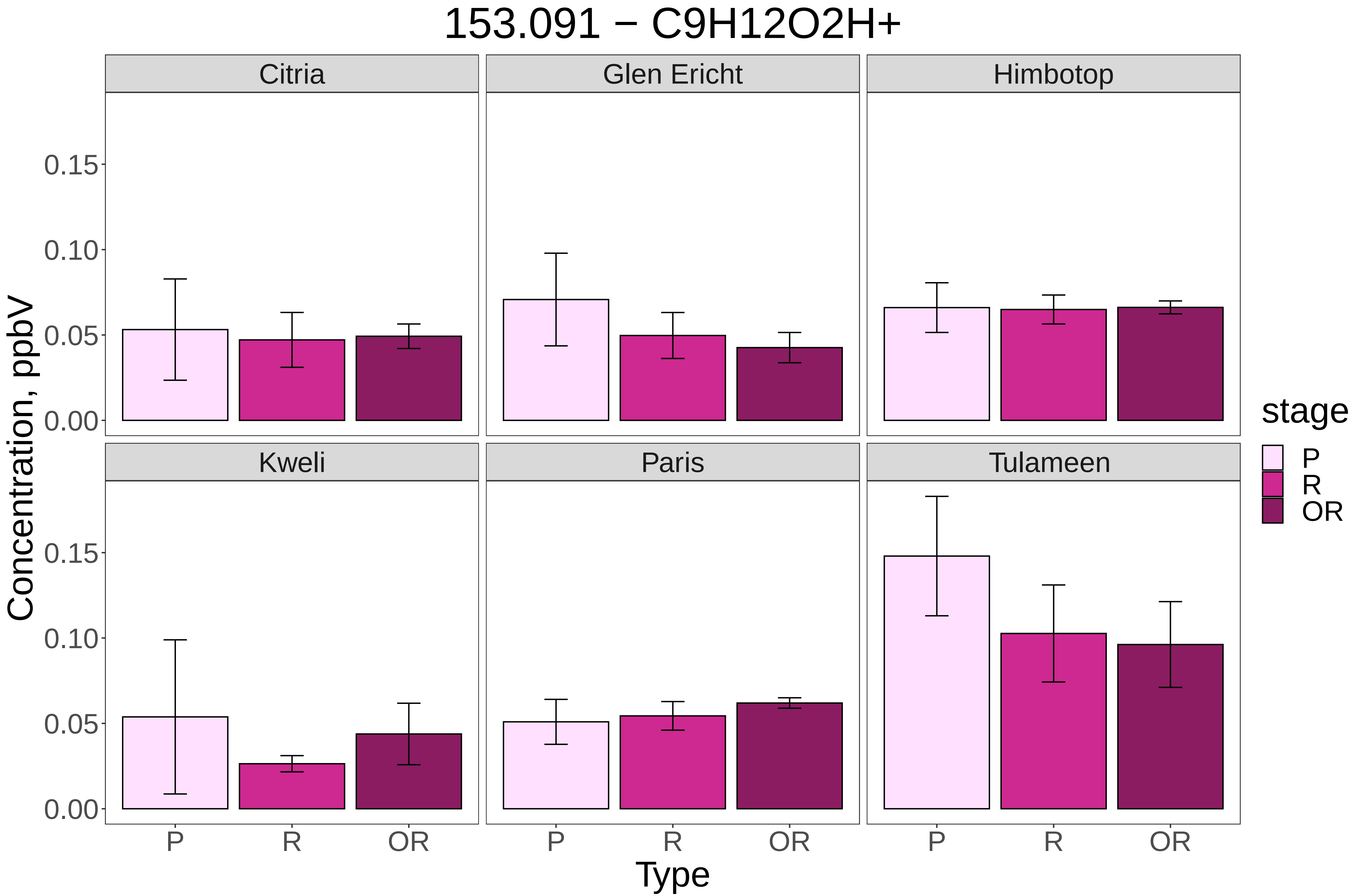

153.091 − C9H12O2H+
Citria
Glen Ericht
Himbotop
Kweli
Paris
Tulameen
Concentration, ppbV
0.00
0.05
0.10
0.15
P
R
OR
Type
stage

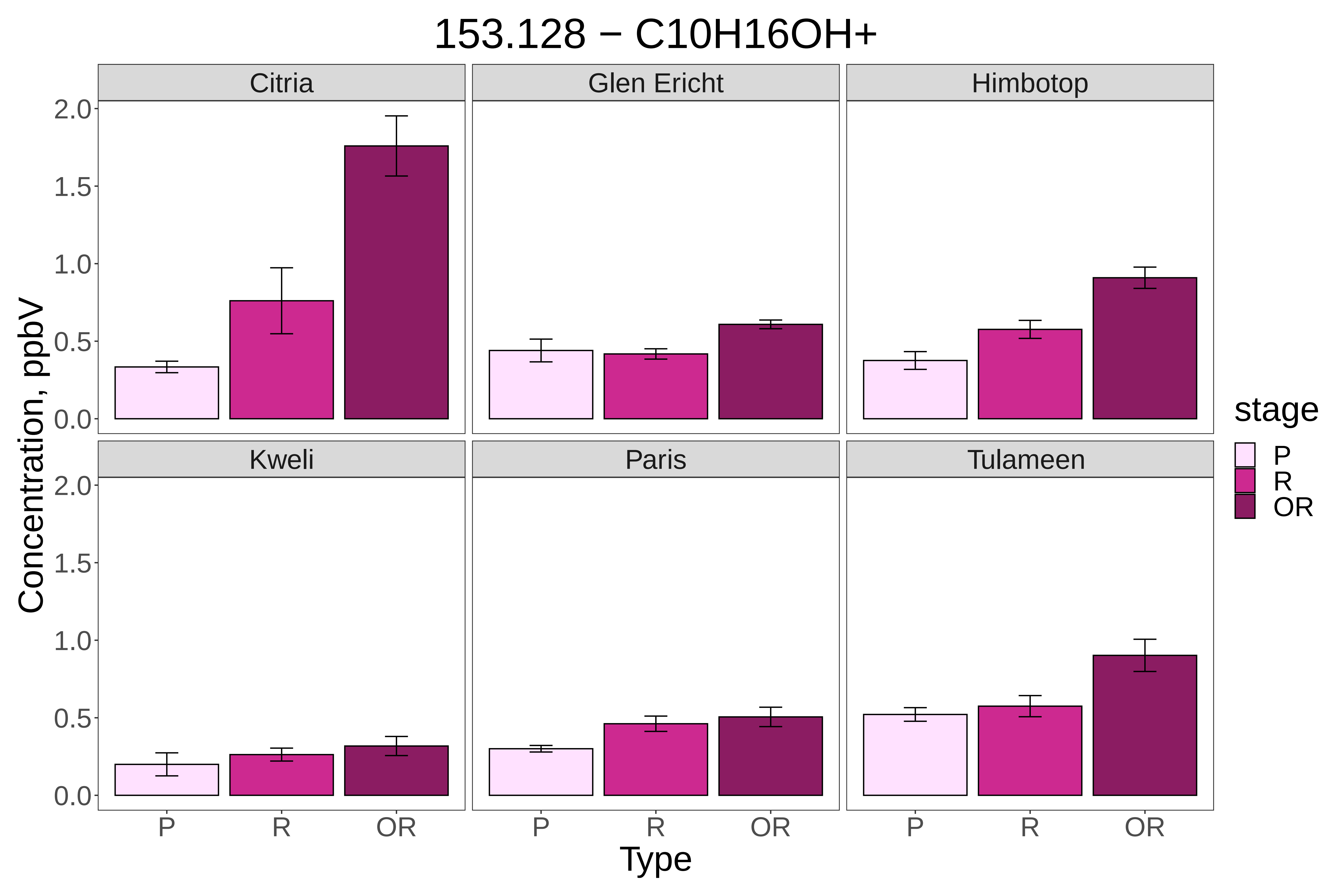

153.128 − C10H16OH+
Citria
Glen Ericht
Himbotop
Kweli
Paris
Tulameen
Concentration, ppbV
0.0
0.5
1.0
1.5
2.0
P
R
OR
Type
stage
P
R
OR

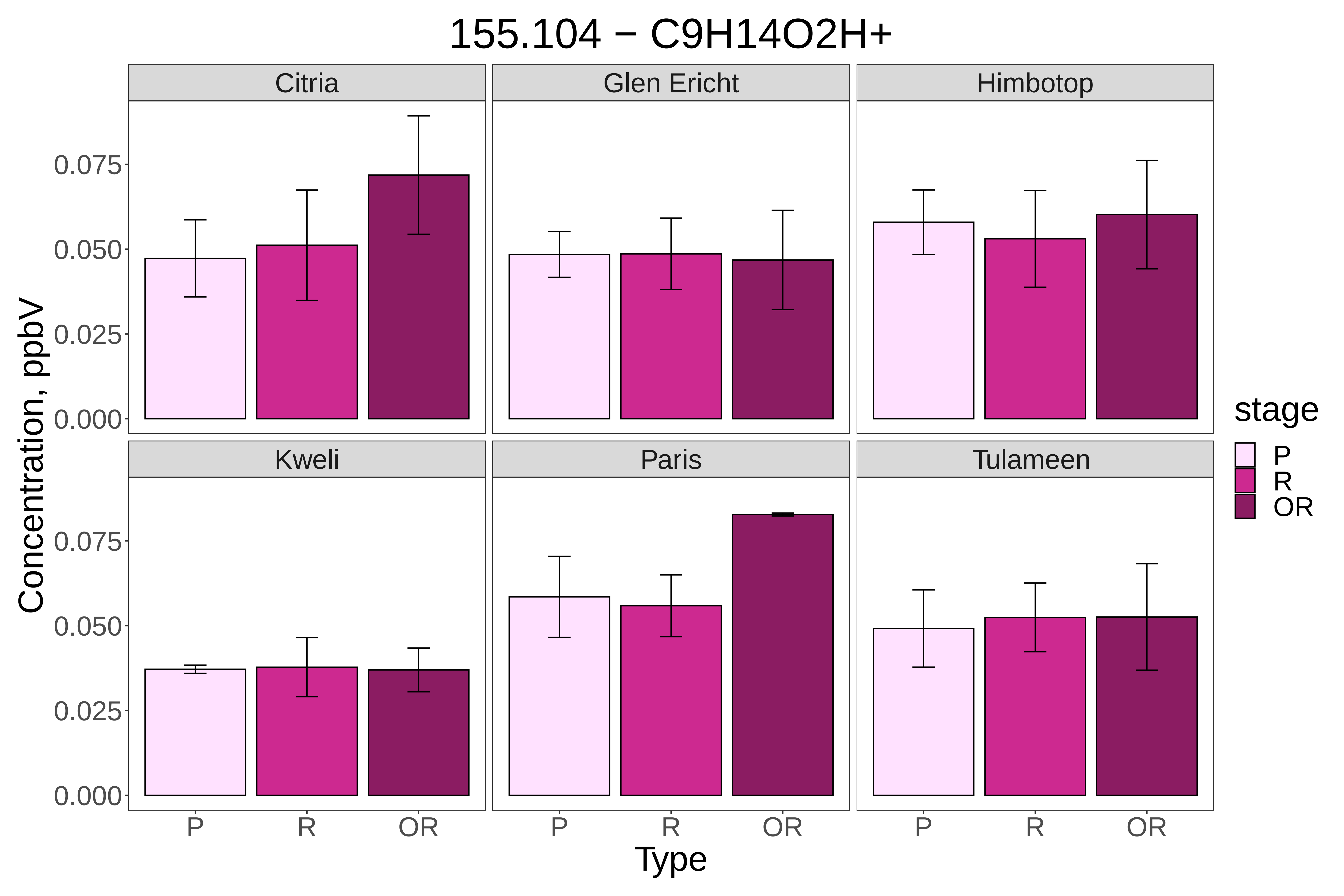
155.104 − C9H14O2H+
Citria
Glen Ericht
Himbotop
Kweli
Paris
Tulameen
0.000
0.025
0.050
0.075
Concentration, ppbV
P
R
OR
Type
stage
P
R
OR

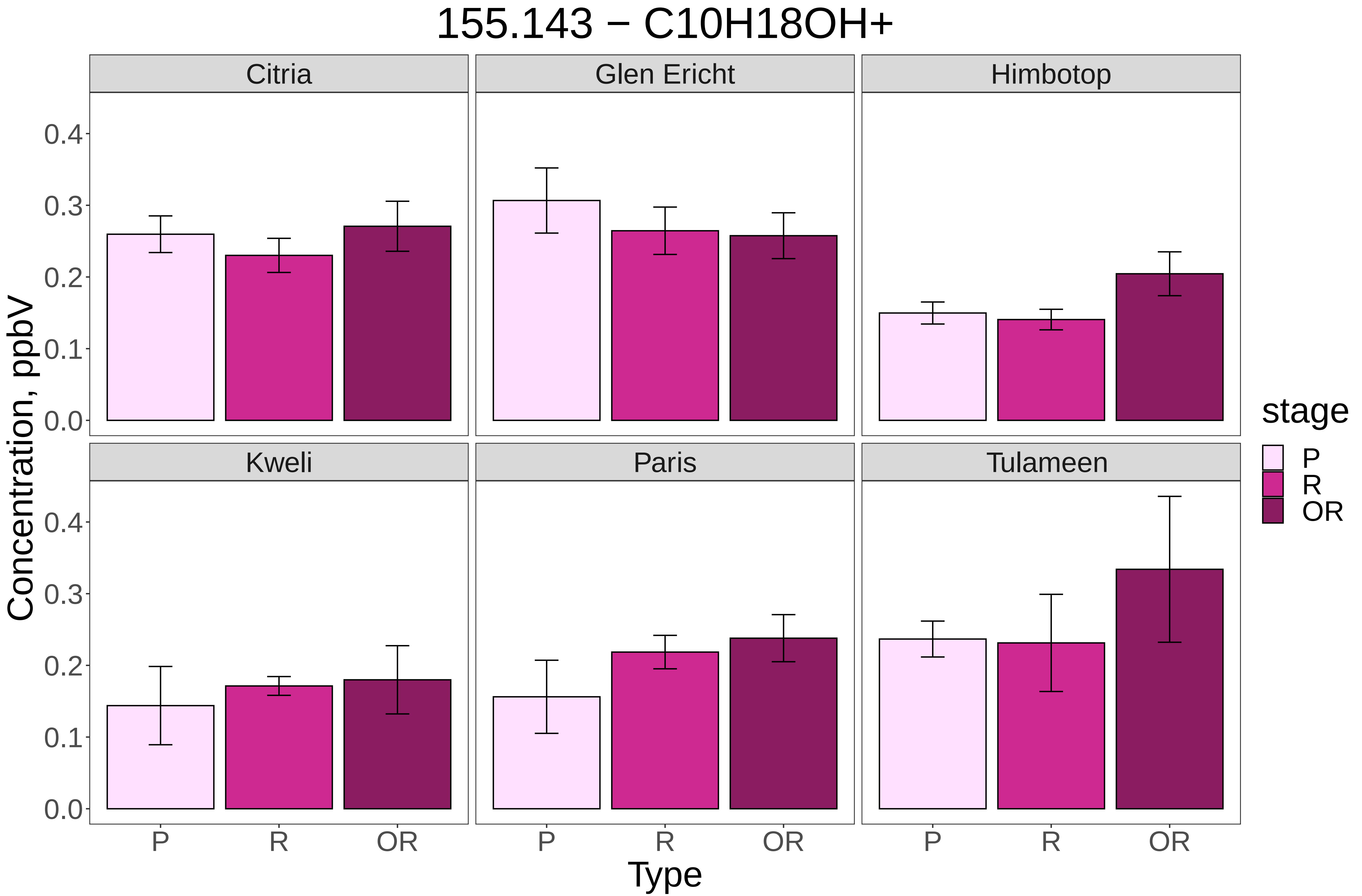
155.143 − C10H18OH+
Citria
Glen Ericht
Himbotop
Kweli
Paris
Tulameen
Concentration, ppbV
0.0
0.1
0.2
0.3
0.4
P
R
OR
Type
stage
P
R
OR

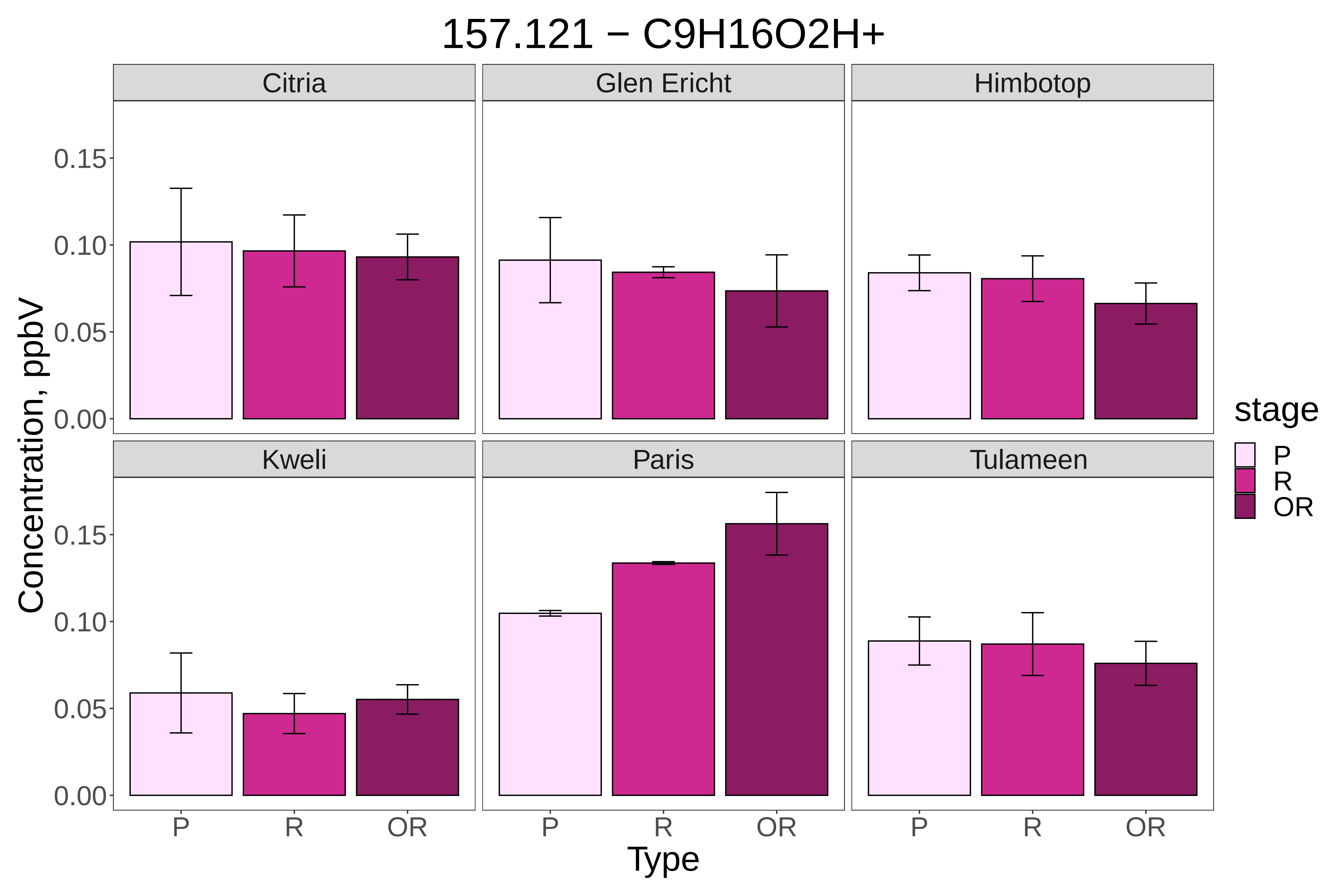

157.121 − C9H16O2H+
Citria
Glen Ericht
Himbotop
Kweli
Paris
Tulameen
0.00
0.05
0.10
0.15
Concentration, ppbV
P
R
OR
Type
stage
P
R
OR

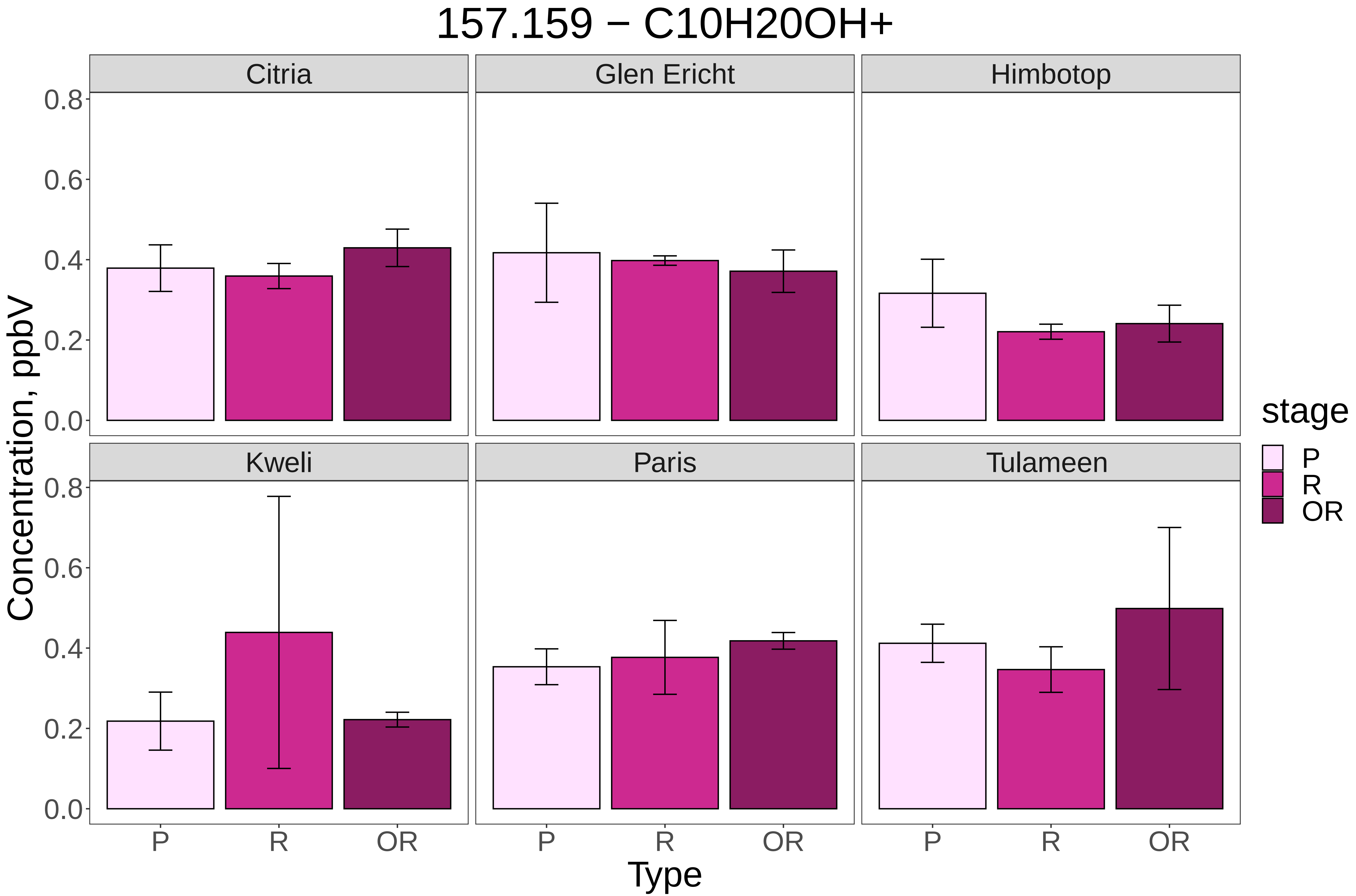
157.159 − C10H20OH+
Citria
Glen Ericht
Himbotop
Kweli
Paris
Tulameen
0.0
0.2
0.4
0.6
0.8
Concentration, ppbV
P
R
OR
Type
stage
P
R
OR

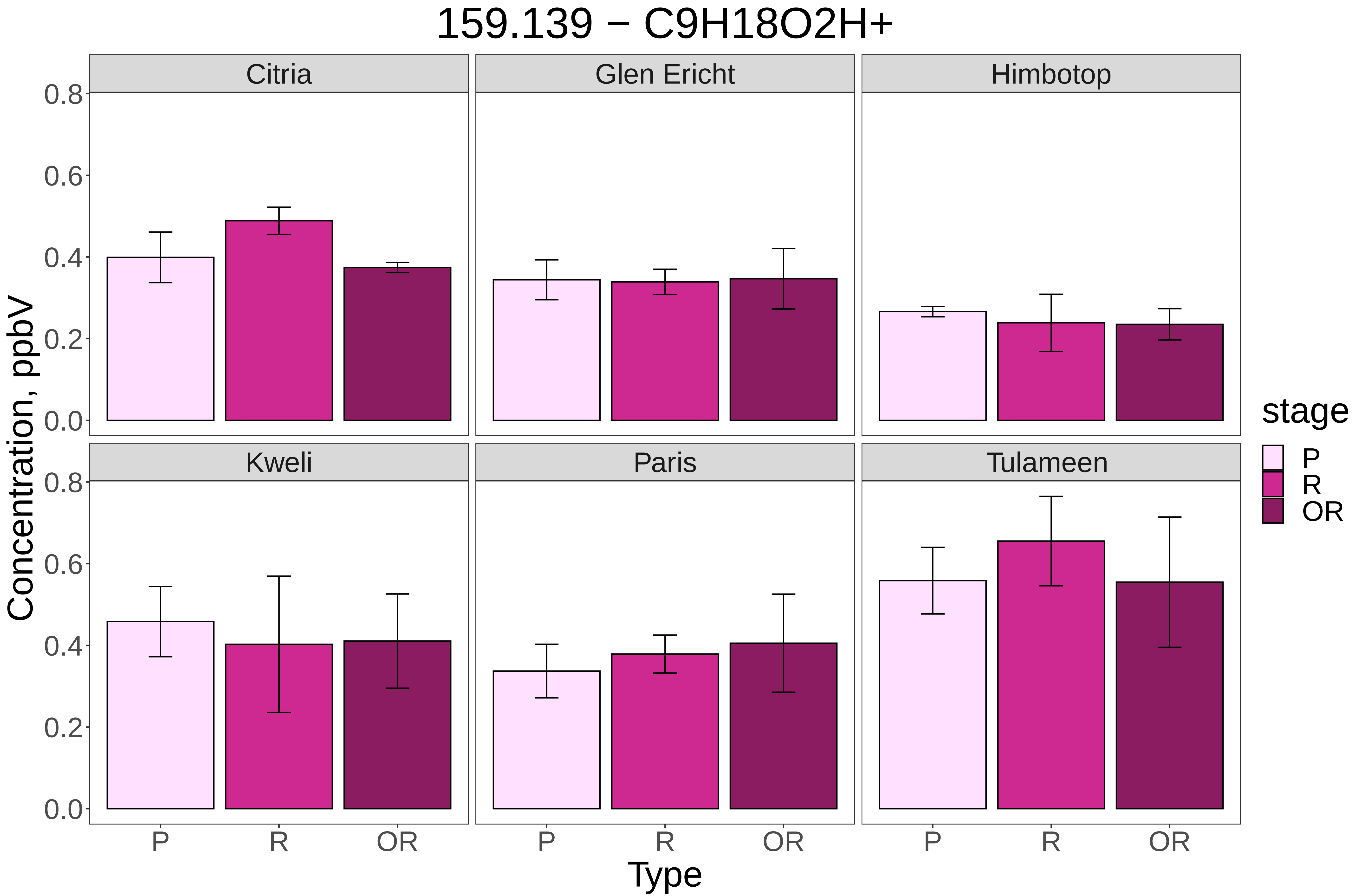

159.139 − C9H18O2H+
Citria
Glen Ericht
Himbotop
Kweli
Paris
Tulameen
Concentration, ppbV
0.0
0.2
0.4
0.6
0.8
P
R
OR
Type
stage
P
R
OR

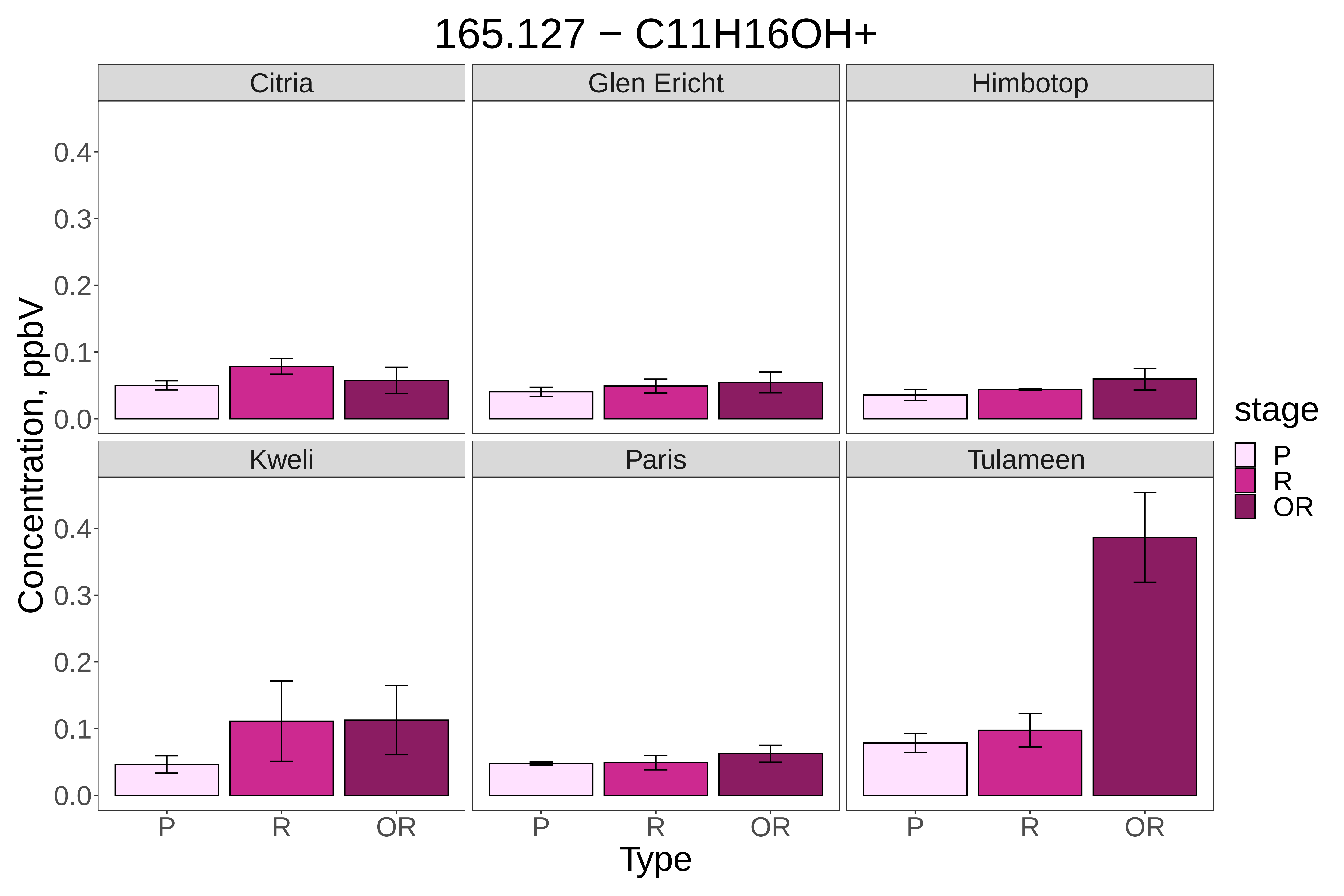
165.127 − C11H16OH+
Citria
Glen Ericht
Himbotop
Kweli
Paris
Tulameen
Concentration, ppbV
0.0
0.1
0.2
0.3
0.4
Type
P
R
OR
stage
P
R
OR

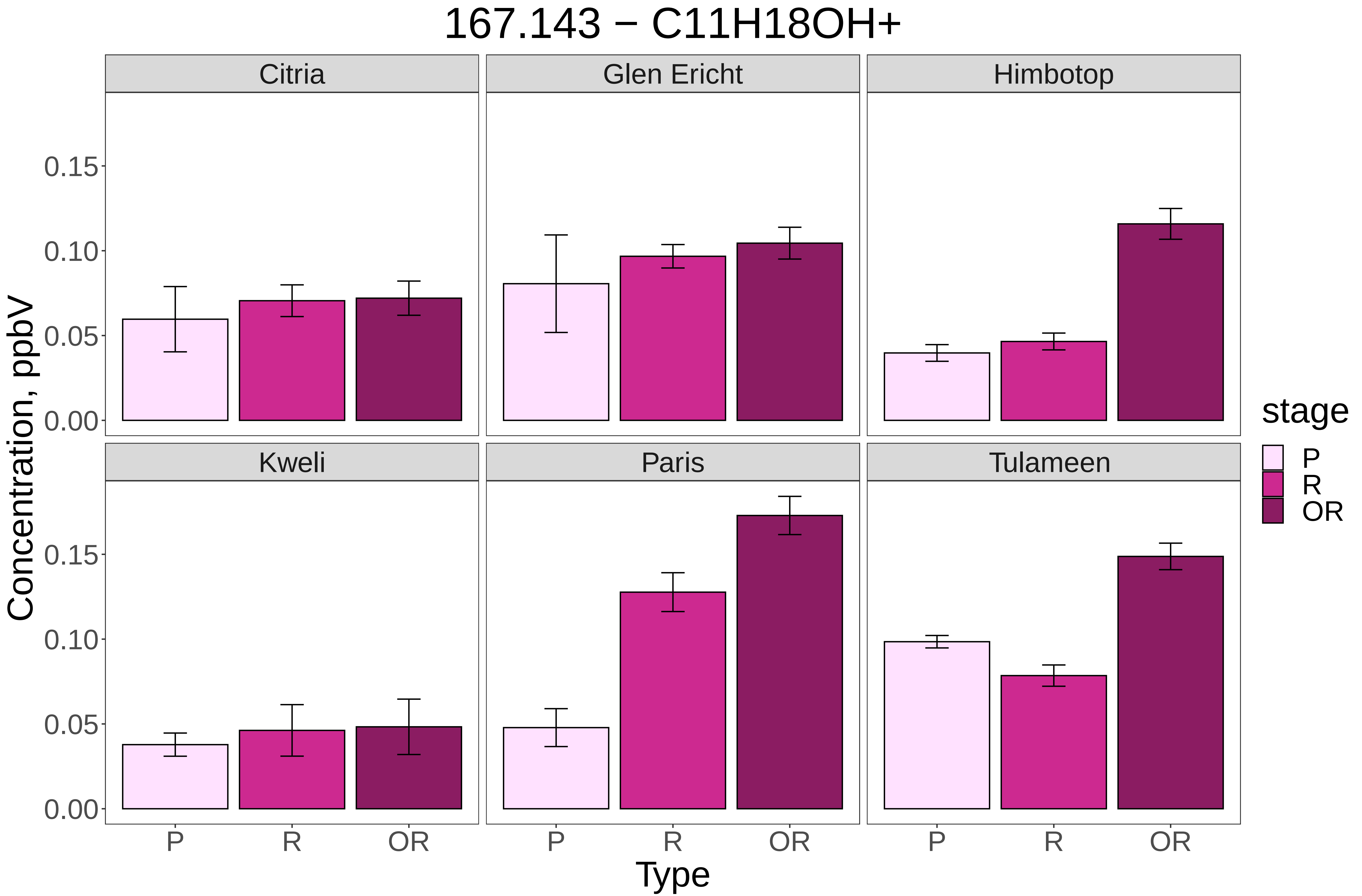

167.143 − C11H18OH+
Citria
Glen Ericht
Himbotop
Kweli
Paris
Tulameen
Concentration, ppbV
0.00
0.05
0.10
0.15
Type
P
R
OR
stage
P
R
OR

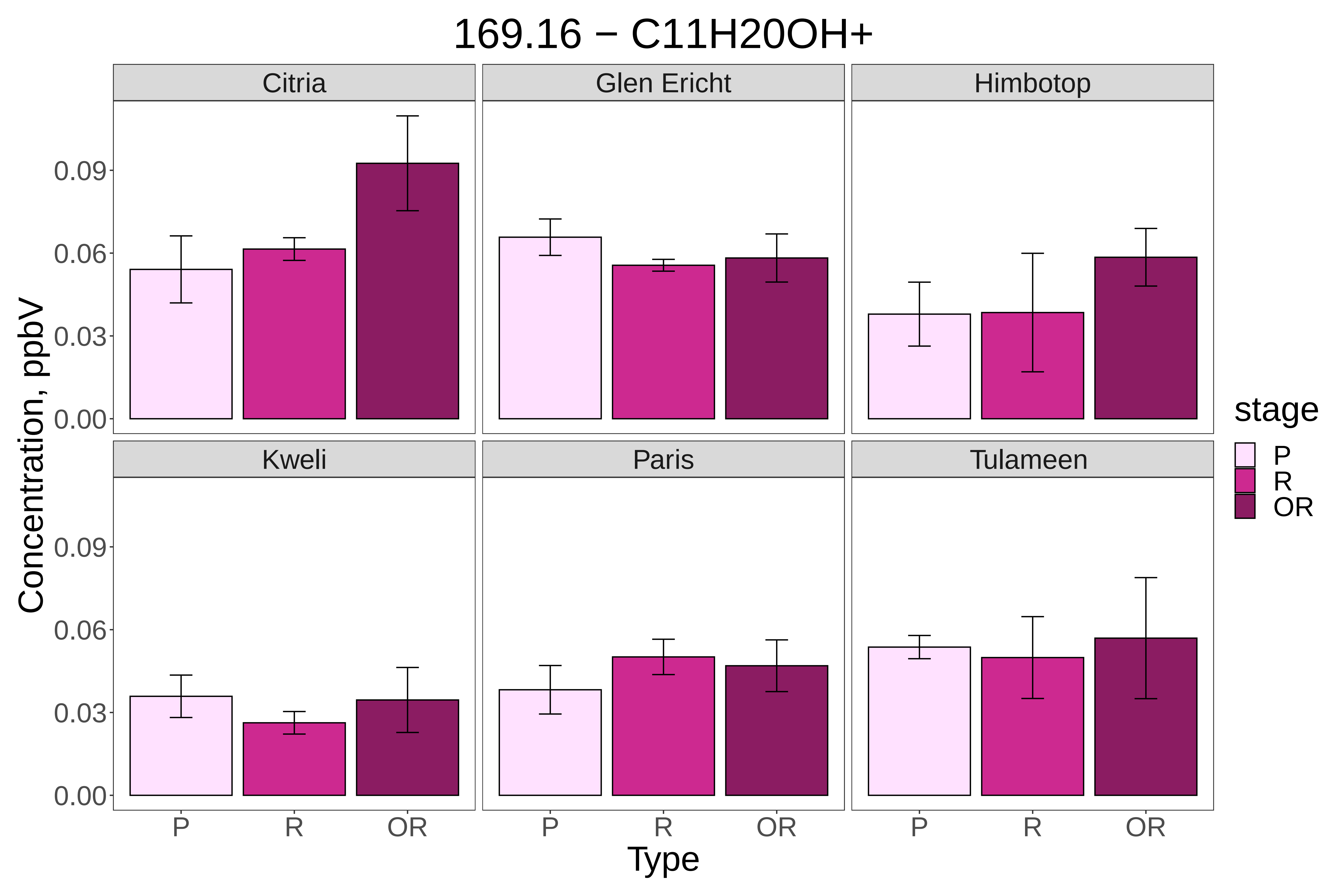
169.16 − C11H20OH+
Citria
Glen Ericht
Himbotop
Kweli
Paris
Tulameen
Concentration, ppbV
0.00
0.03
0.06
0.09
P
R
OR
Type
stage
P
R
OR

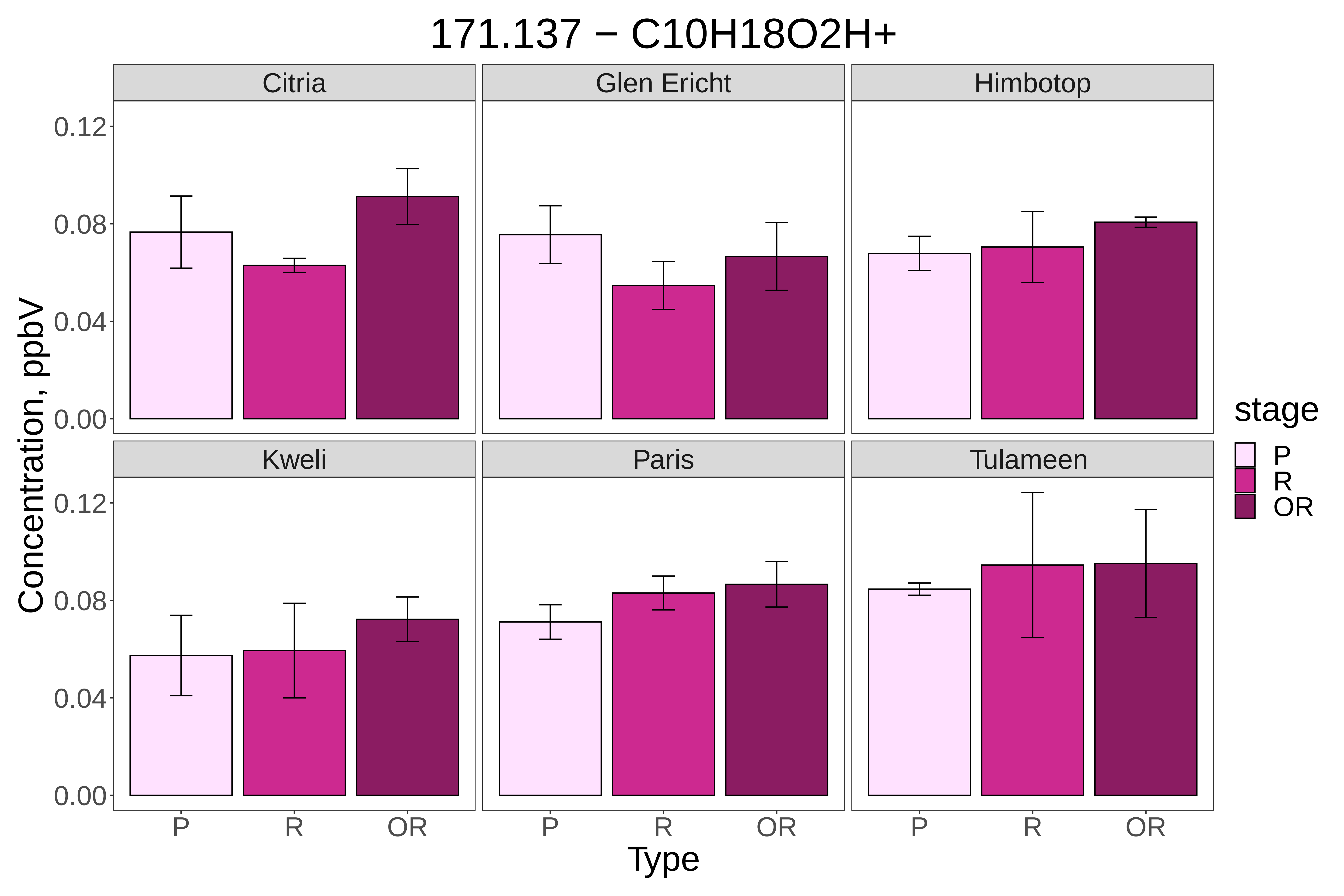
171.137 − C10H18O2H+
Citria
Glen Ericht
Himbotop
Kweli
Paris
Tulameen
Concentration, ppbV
0.00
0.04
0.08
0.12
P
R
OR
Type
stage
P
R
OR

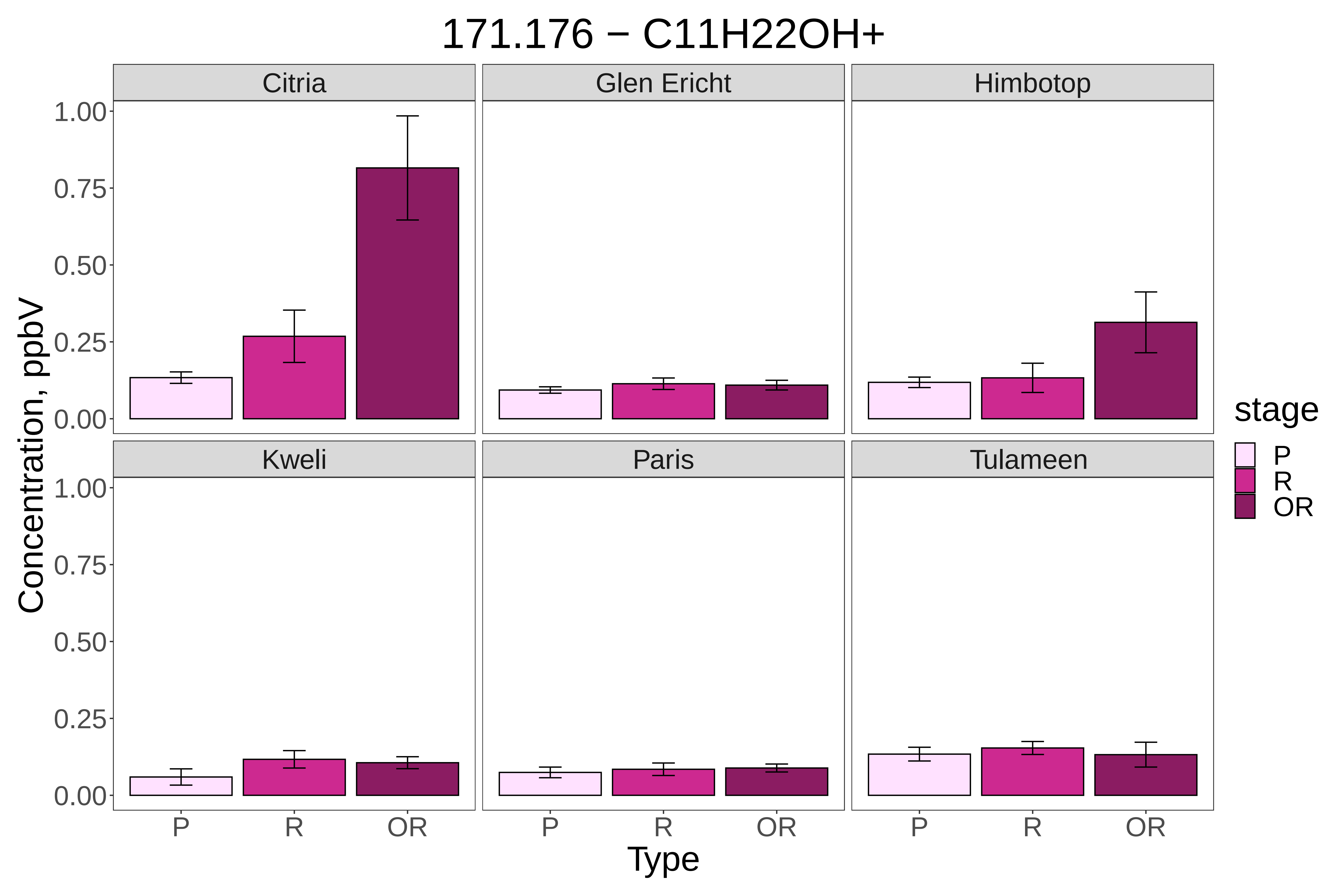
171.176 − C11H22OH+
Citria
Glen Ericht
Himbotop
Kweli
Paris
Tulameen
1.00
0.75
0.50
0.25
0.00
Concentration, ppbV
P
R
OR
Type
stage
P
R
OR

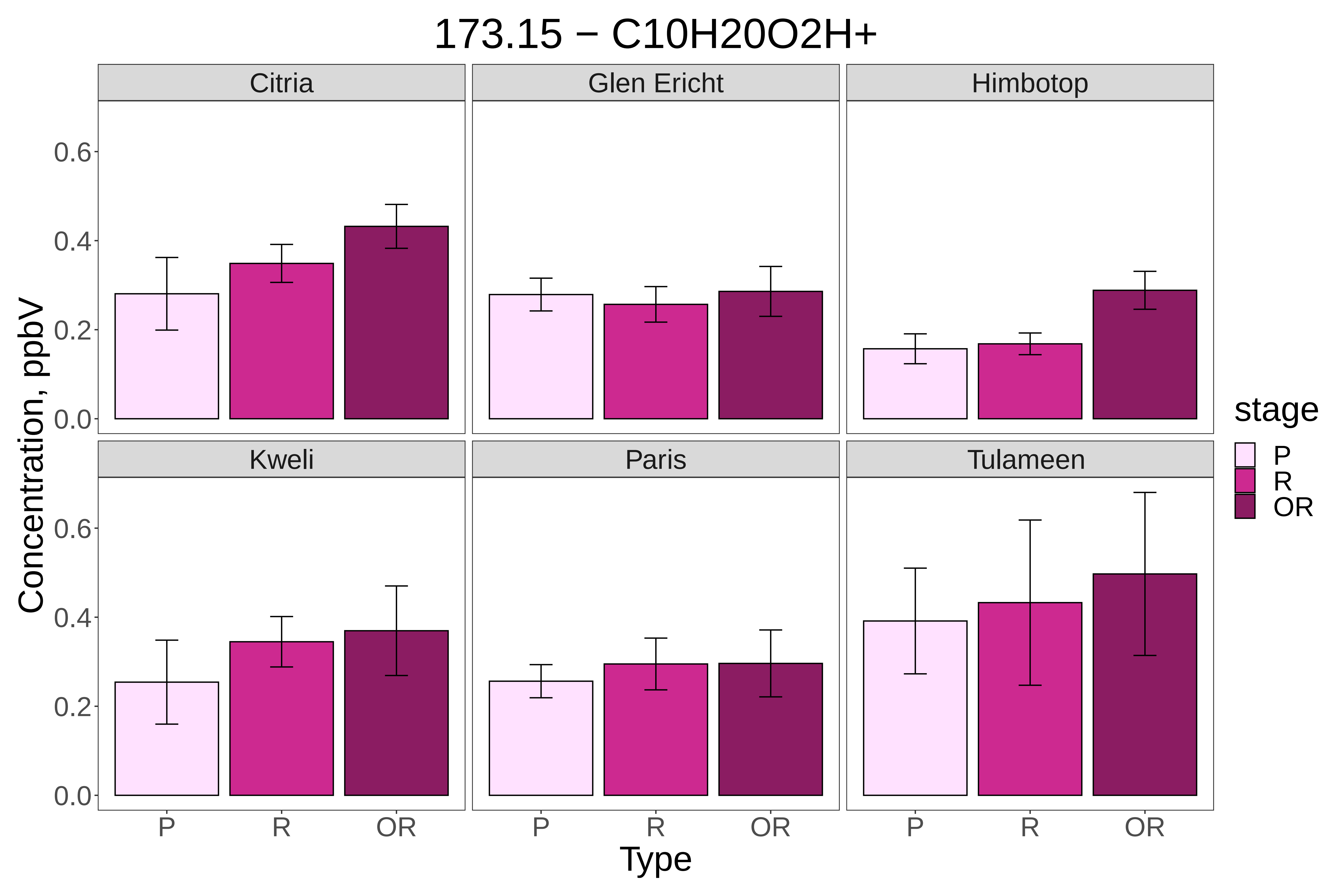

173.15 − C10H20O2H+
Citria
Glen Ericht
Himbotop
Kweli
Paris
Tulameen
Concentration, ppbV
0.0
0.2
0.4
0.6
P
R
OR
Type
stage
P
R
OR

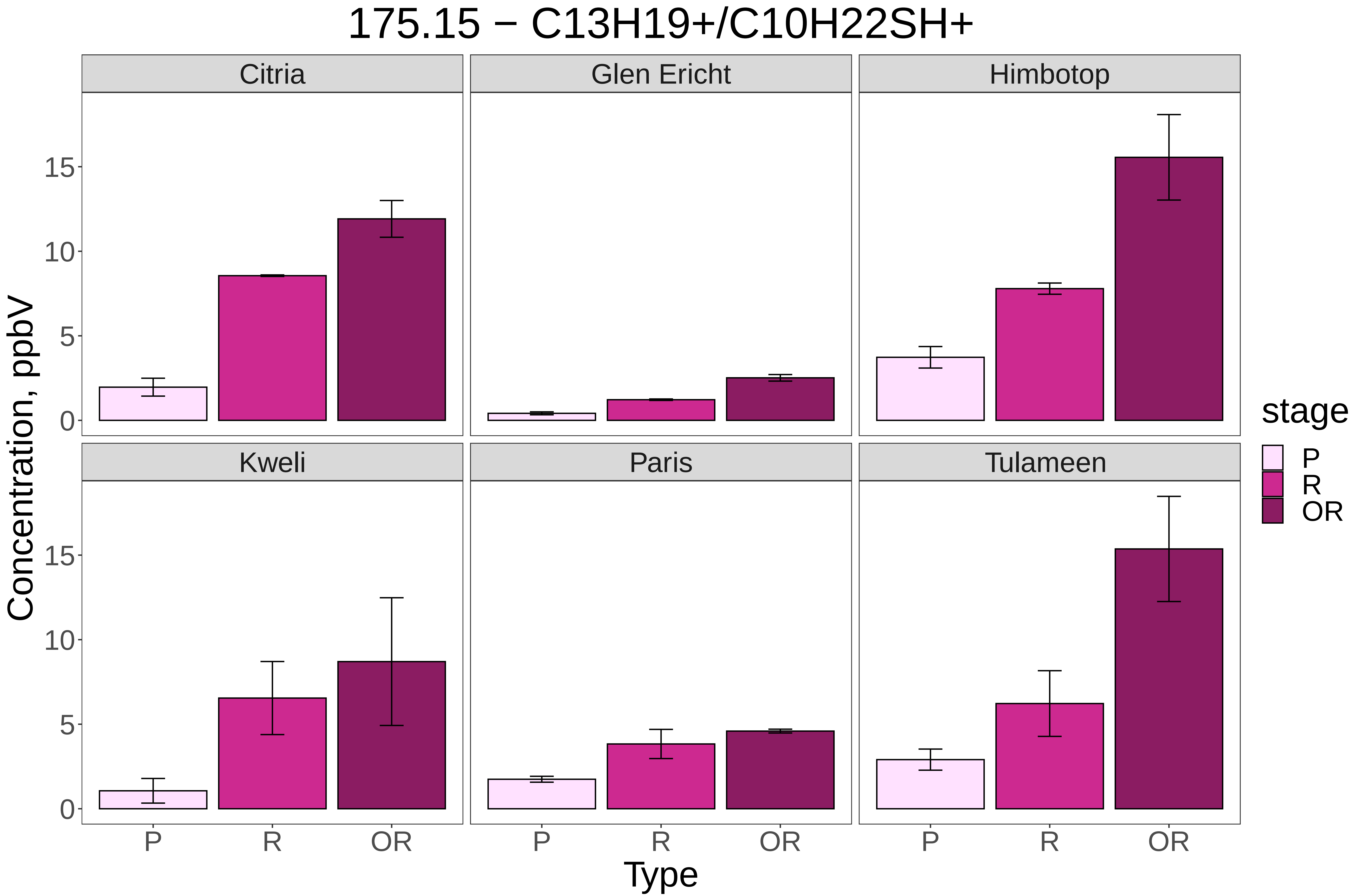
175.15 − C13H19+/C10H22SH+
Citria
Glen Ericht
Himbotop
Kweli
Paris
Tulameen
Concentration, ppbV
0
5
10
15
P
R
OR
Type
stage
P
R
OR

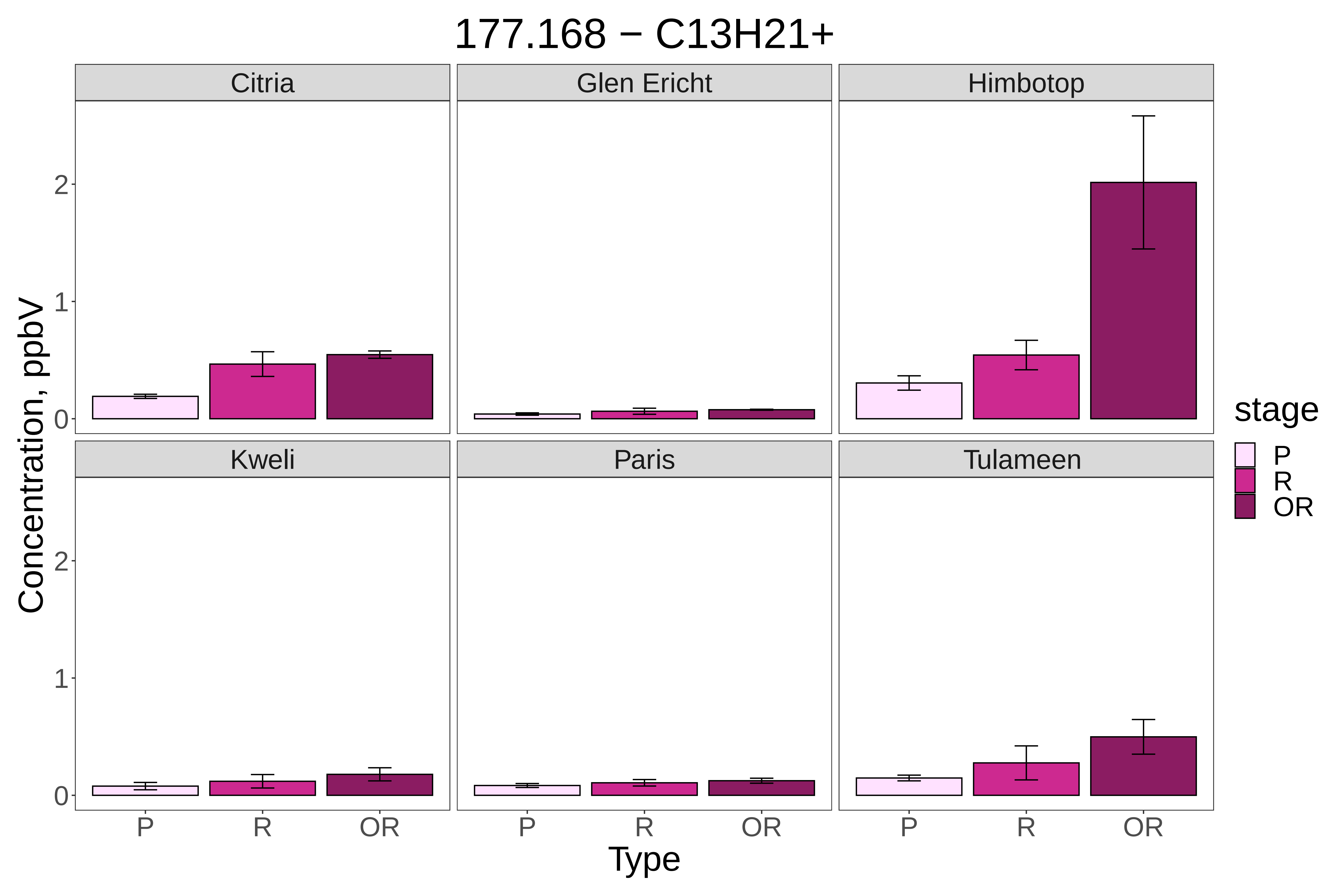

177.168 − C13H21+
Citria
Glen Ericht
Himbotop
Kweli
Paris
Tulameen
Concentration, ppbV
0
1
2
P
R
OR
Type
stage

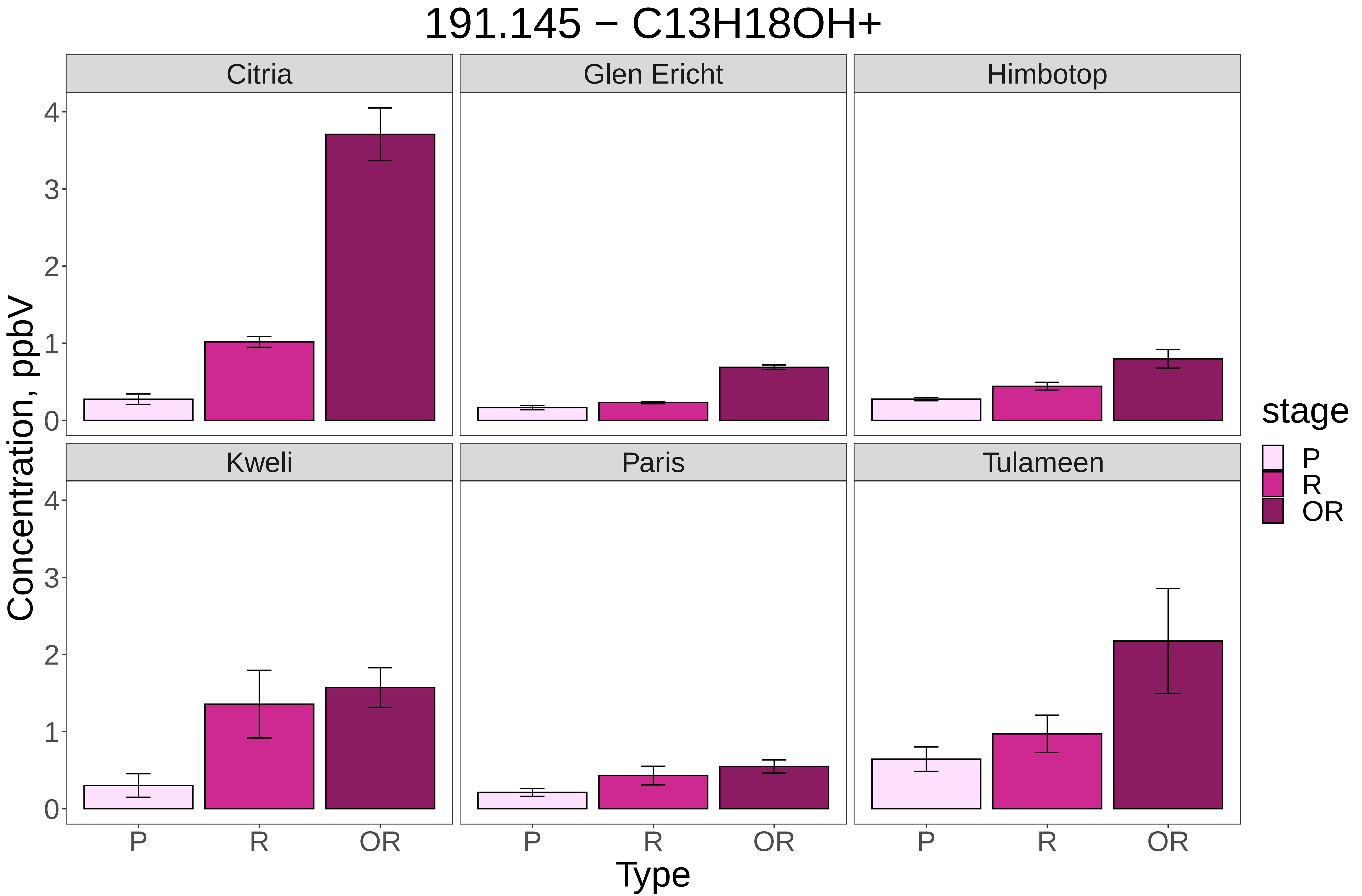
191.145 − C13H18OH+
Citria
Glen Ericht
Himbotop
Kweli
Paris
Tulameen
Concentration, ppbV
0
1
2
3
4
P
R
OR
Type
stage
P
R
OR

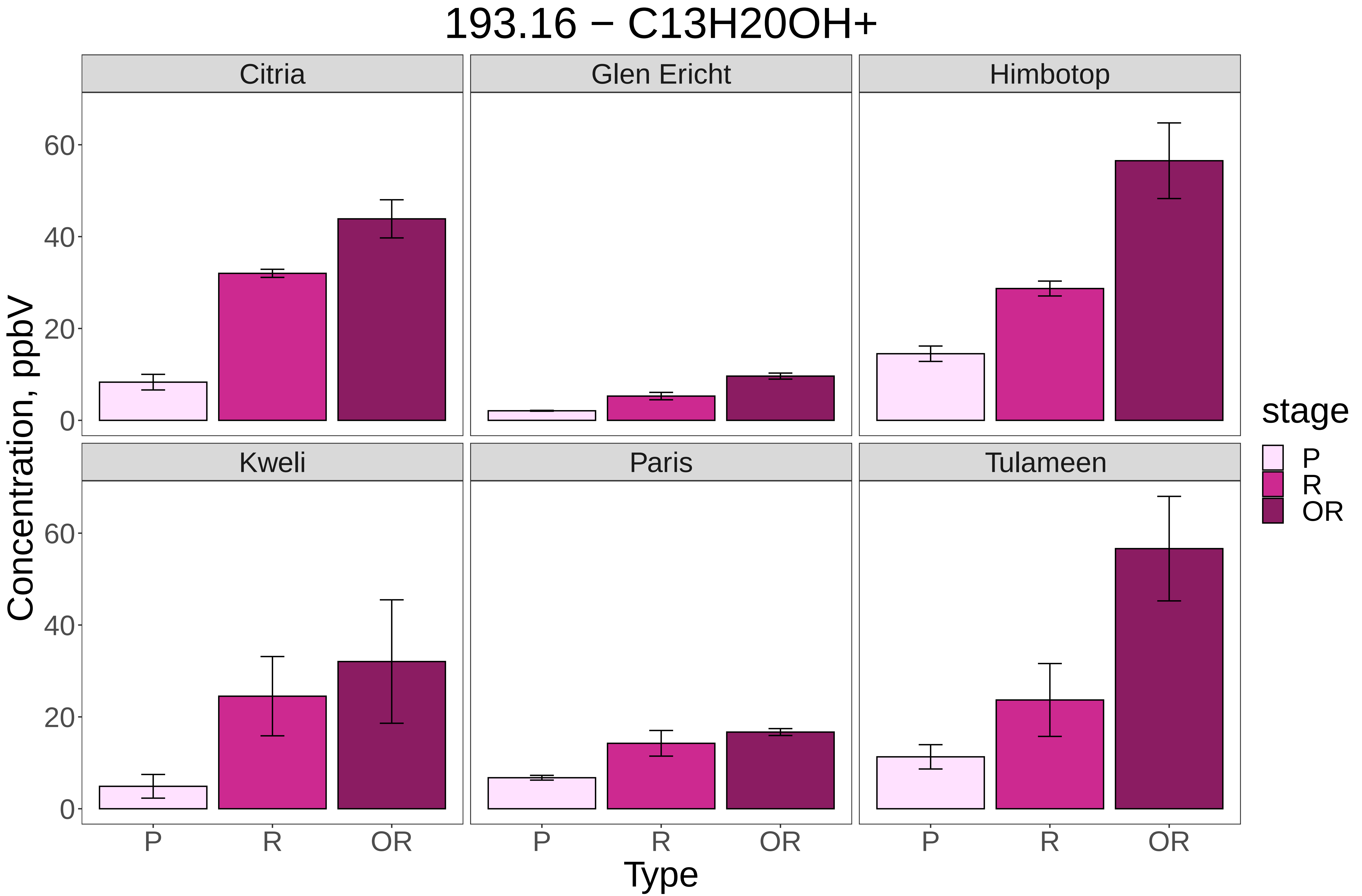

193.16 − C13H20OH+
Citria
Glen Ericht
Himbotop
Kweli
Paris
Tulameen
Concentration, ppbV
0
20
40
60
P
R
OR
Type
stage
P
R
OR

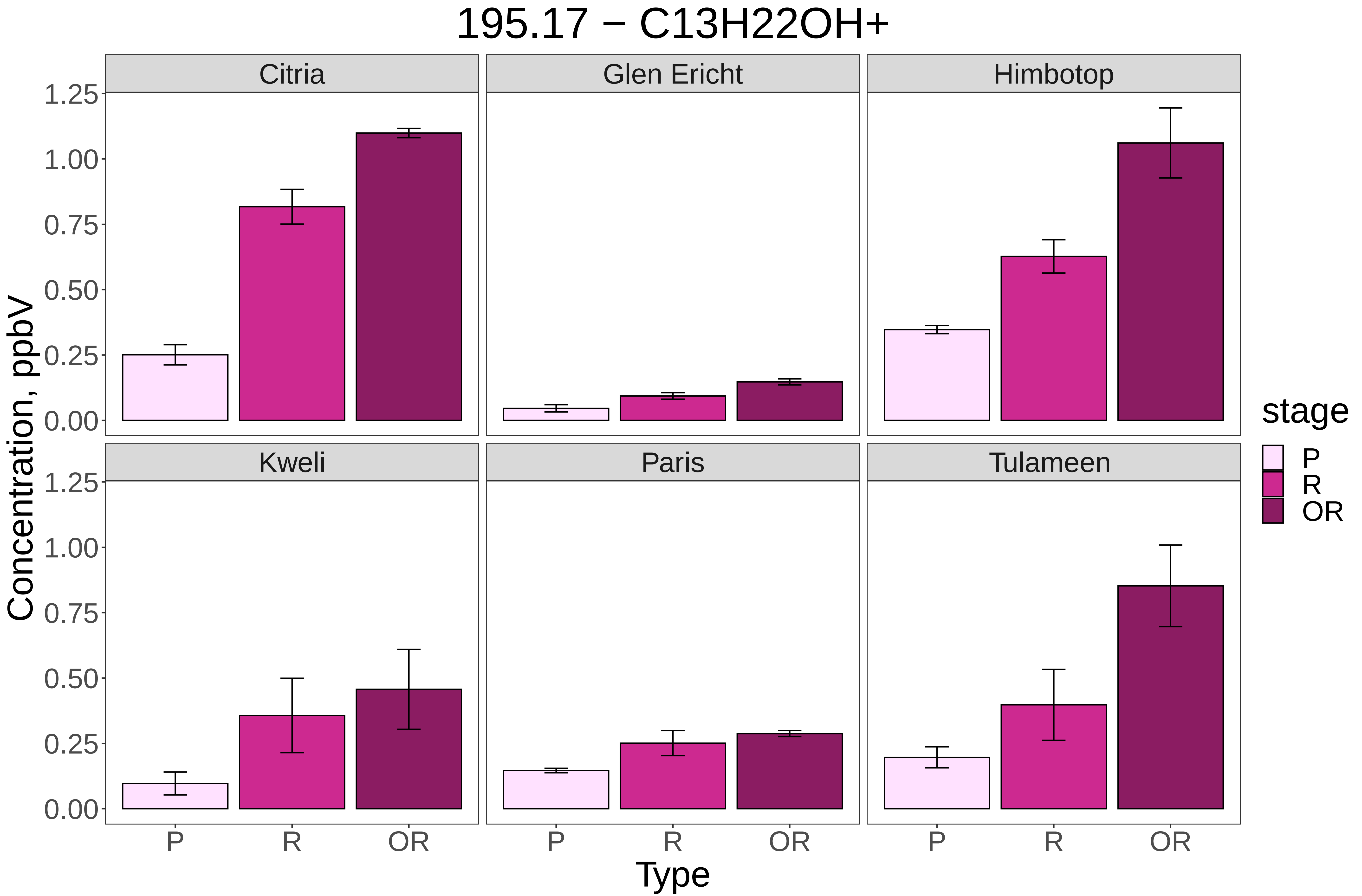

195.17 − C13H22OH+
Citria
Glen Ericht
Himbotop
Kweli
Paris
Tulameen
Concentration, ppbV
0.00
0.25
0.50
0.75
1.00
1.25
P
R
OR
Type
stage
P
R
OR

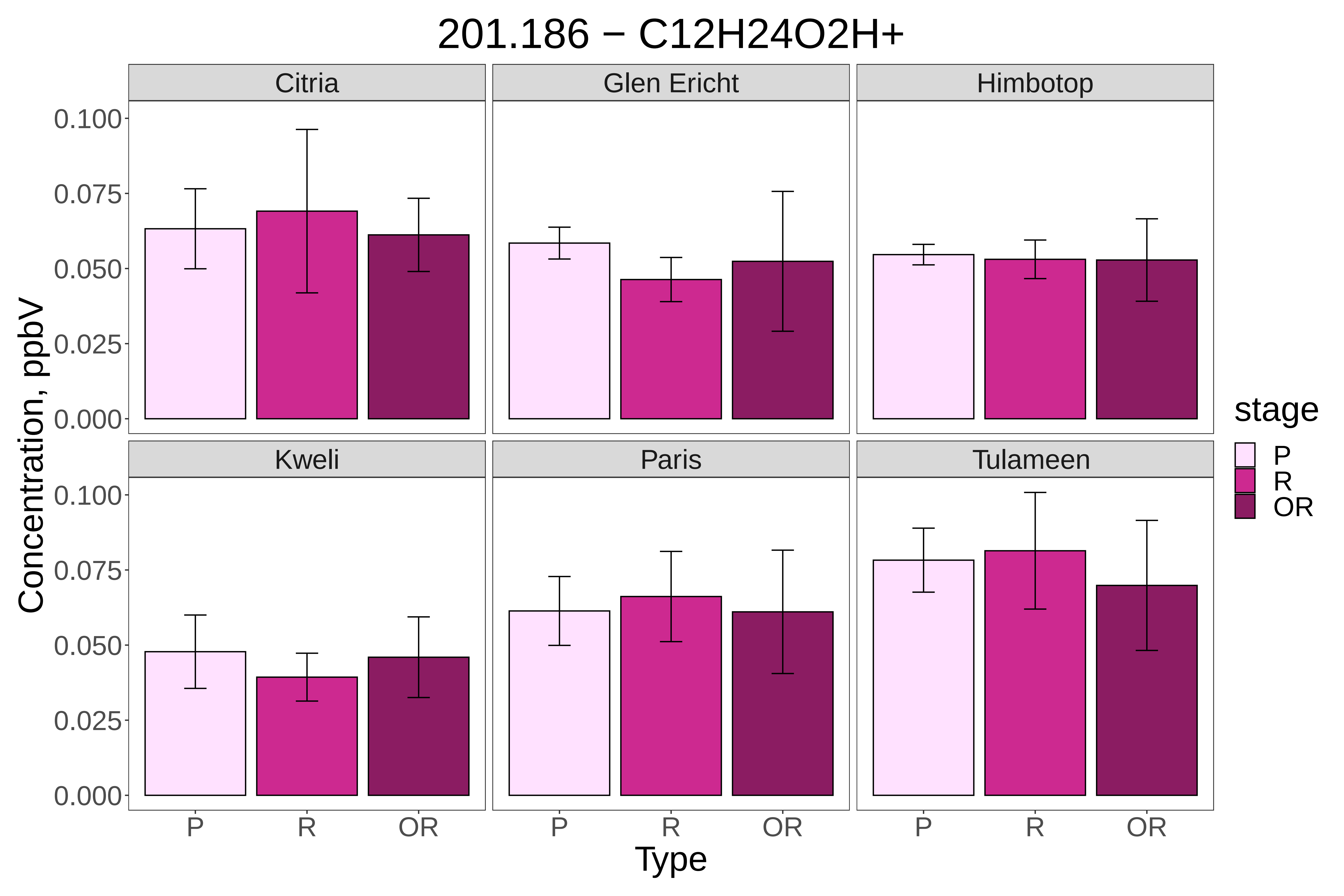
201.186 − C12H24O2H+
Citria
Glen Ericht
Himbotop
Kweli
Paris
Tulameen
Concentration, ppbV
0.000
0.025
0.050
0.075
0.100
P
R
OR
Type
stage
P
R
OR

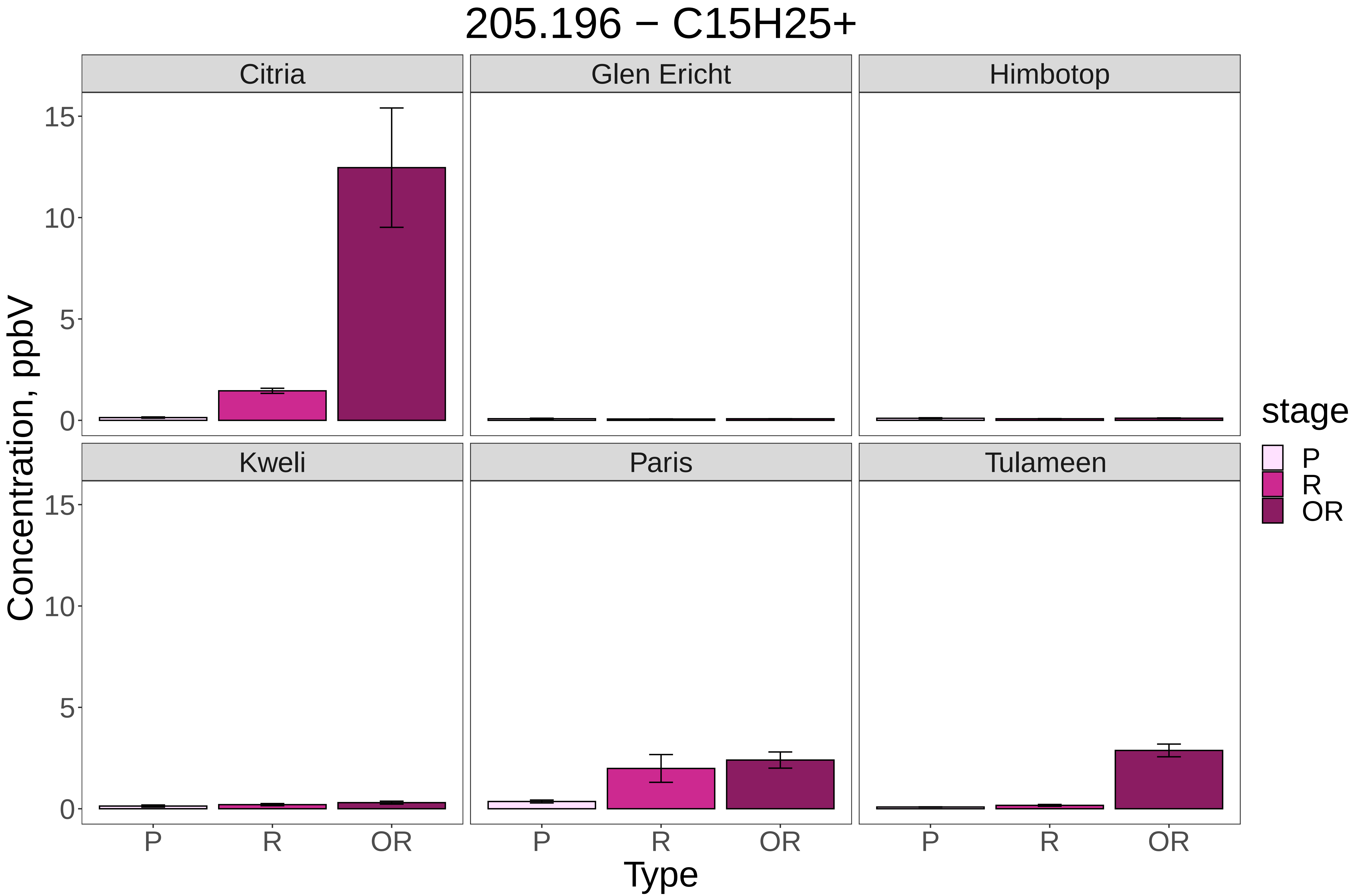
205.196 − C15H25+
Citria
Glen Ericht
Himbotop
Kweli
Paris
Tulameen
Concentration, ppbV
0
5
10
15
P
R
OR
Type
stage
P
R
OR

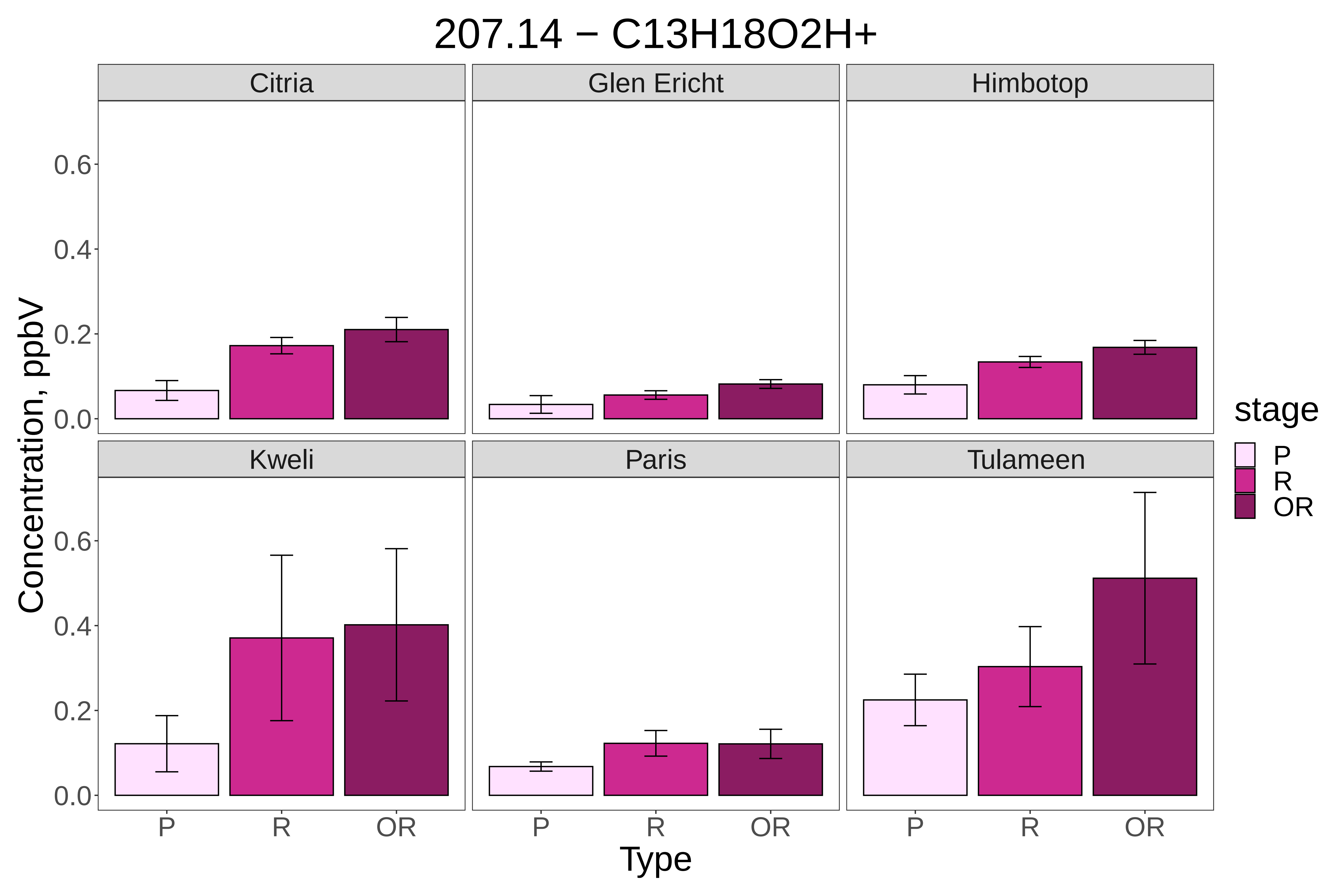

207.14 − C13H18O2H+
Citria
Glen Ericht
Himbotop
Kweli
Paris
Tulameen
Concentration, ppbV
0.0
0.2
0.4
0.6
P
R
OR
Type
stage
P
R
OR

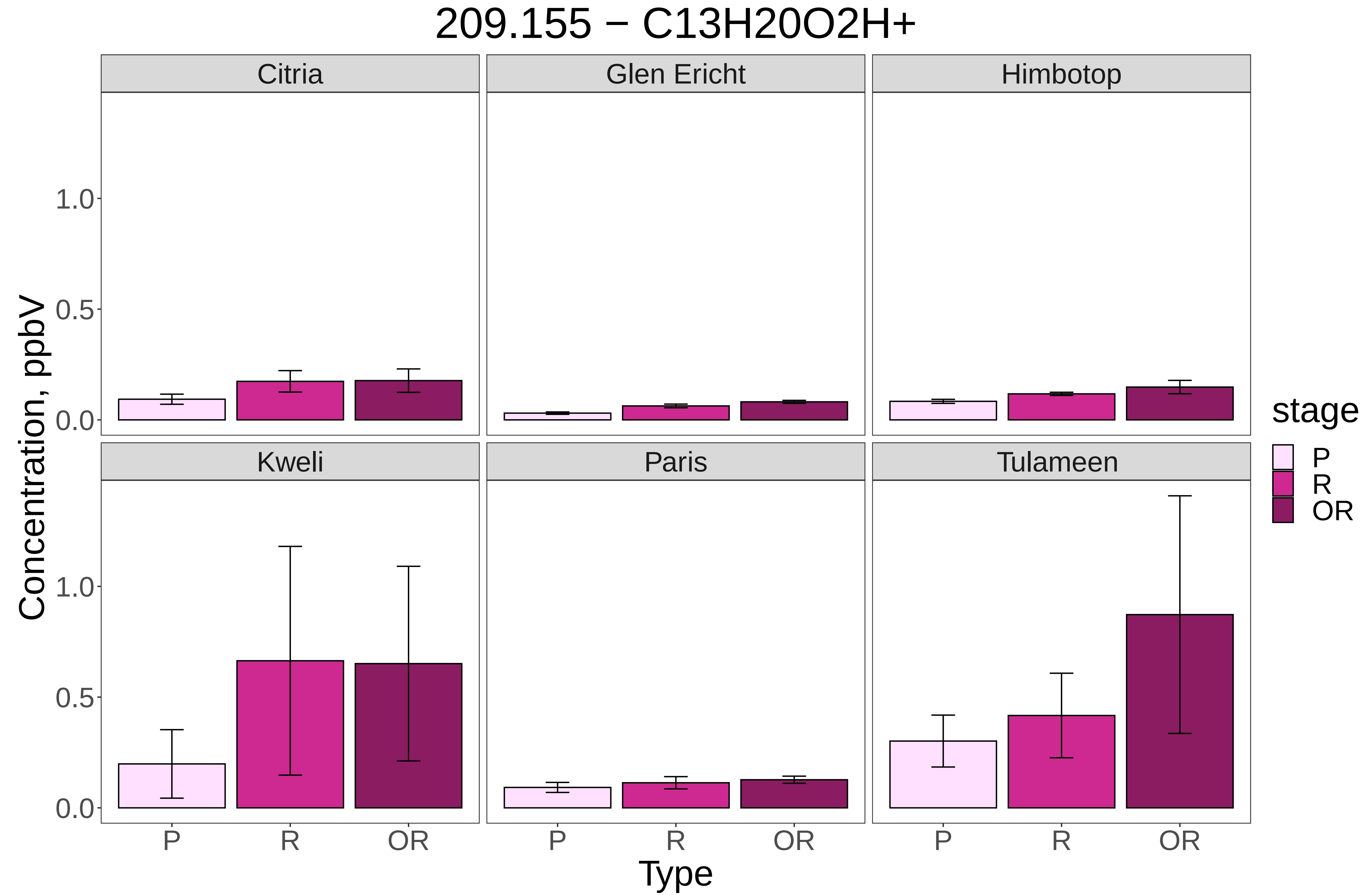
209.155 − C13H20O2H+
Citria
Glen Ericht
Himbotop
Kweli
Paris
Tulameen
0.0
0.5
1.0
Concentration, ppbV
P
R
OR
Type
stage
P
R
OR
