# Supplementary material for: Volatilomics of raspberry fruit germplasm by combining chromatographic and direct-injection mass spectrometric techniques
Source: Front Mol Biosci. 2023 Apr 13;10:1155564. doi: 10.3389/fmolb.2023.1155564 (PMC10133483; doi:10.3389/fmolb.2023.1155564)
Supplement: Supplementary file 4 [file Image6.PDF]

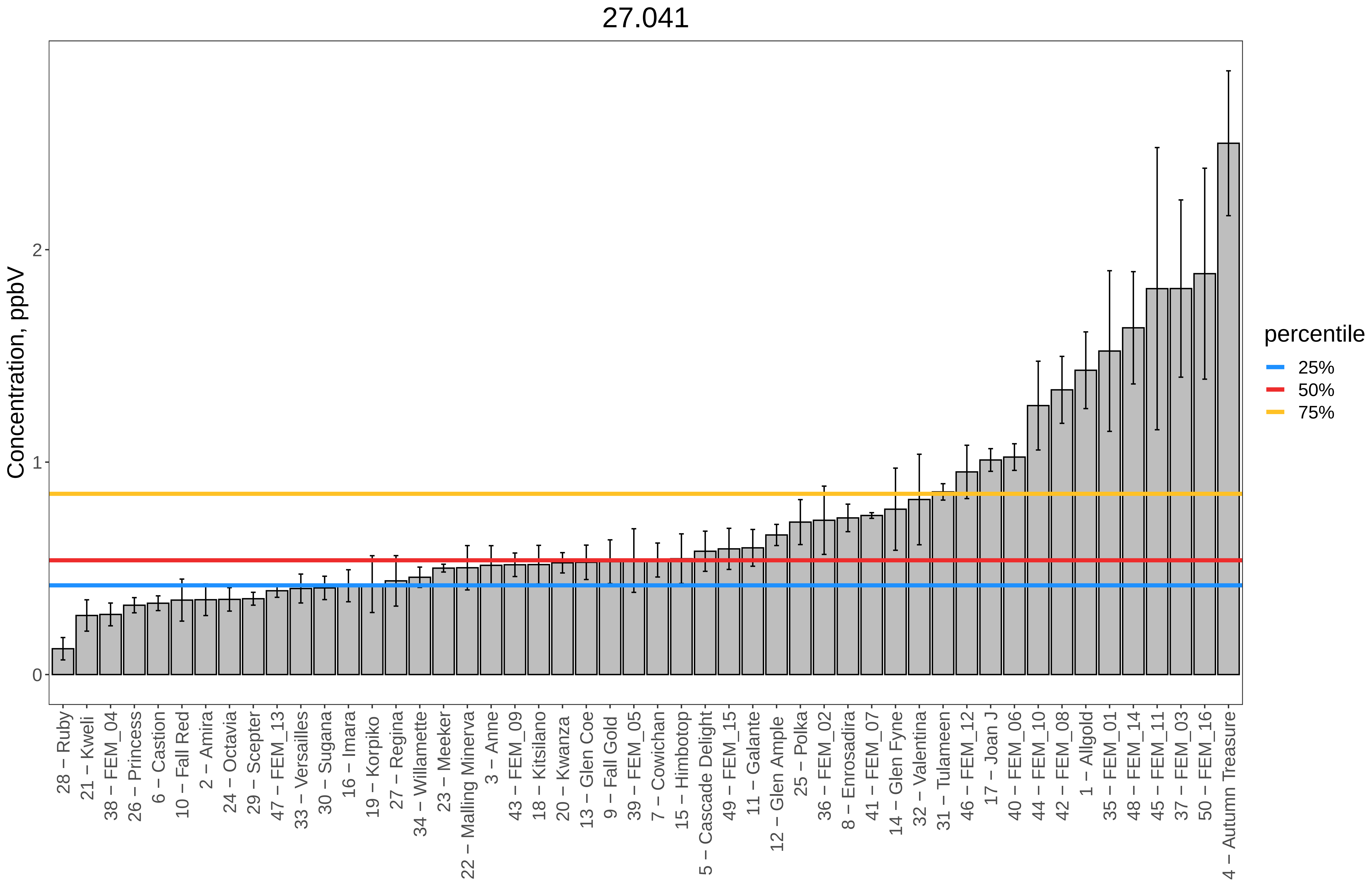
27.041
Concentration, ppbV
0
1
2
percentile
25%
50%
75%
28 – Ruby
21 – Kweli
38 – FEM_04
26 – Princess
6 – Castion
10 – Fall Red
2 – Amira
24 – Octavia
29 – Scepter
47 – FEM_13
33 – Versailles
30 – Sugana
16 – Imara
19 – Korpiko
27 – Regina
34 – Willamette
23 – Meeker
22 – Malling Minerva
3 – Anne
43 – FEM_09
18 – Kitsilano
20 – Kwanza
13 – Glen Coe
9 – Fall Gold
39 – FEM_05
7 – Cowichan
15 – Himbotop
5 – Cascade Delight
49 – FEM_15
11 – Galante
12 – Glen Ample
25 – Polka
36 – FEM_02
8 – Enrosadira
41 – FEM_07
14 – Glen Fyne
32 – Valentina
31 – Tulameen
46 – FEM_12
17 – Joan J
40 – FEM_06
44 – FEM_10
42 – FEM_08
1 – Allgold
35 – FEM_01
48 – FEM_14
45 – FEM_11
37 – FEM_03
50 – FEM_16
4 – Autumn Treasure

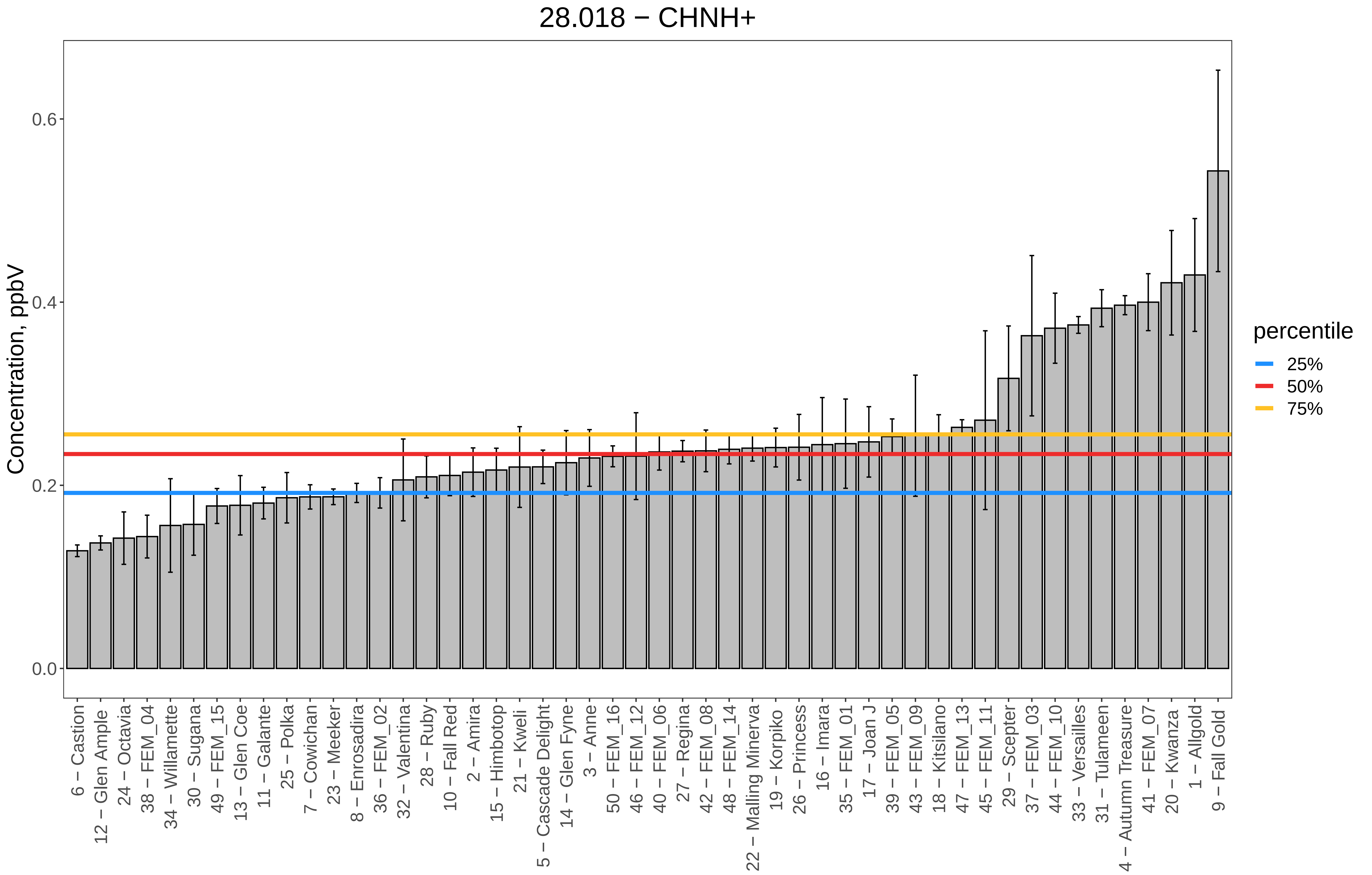

28.018 − CHNH+
Concentration, ppbV
0.0
0.2
0.4
0.6
percentile
25%
50%
75%
6 − Castion
12 − Glen Ample
24 − Octavia
38 − FEM_04
34 − Willamette
30 − Sugana
49 − FEM_15
13 − Glen Coe
11 − Galante
25 − Polka
7 − Cowichan
23 − Meeker
8 − Enrosadira
36 − FEM_02
32 − Valentina
28 − Ruby
10 − Fall Red
2 − Amira
15 − Himbotop
21 − Kweli
5 − Cascade Delight
14 − Glen Fyne
3 − Anne
50 − FEM_16
46 − FEM_12
40 − FEM_06
27 − Regina
42 − FEM_08
48 − FEM_14
22 − Malling Minerva
19 − Korpiko
26 − Princess
16 − Imara
35 − FEM_01
17 − Joan J
39 − FEM_05
43 − FEM_09
18 − Kitsilano
47 − FEM_13
45 − FEM_11
29 − Scepter
37 − FEM_03
44 − FEM_10
33 − Versailles
31 − Tulameen
4 − Autumn Treasure
41 − FEM_07
20 − Kwanza
1 − Allgold
9 − Fall Gold

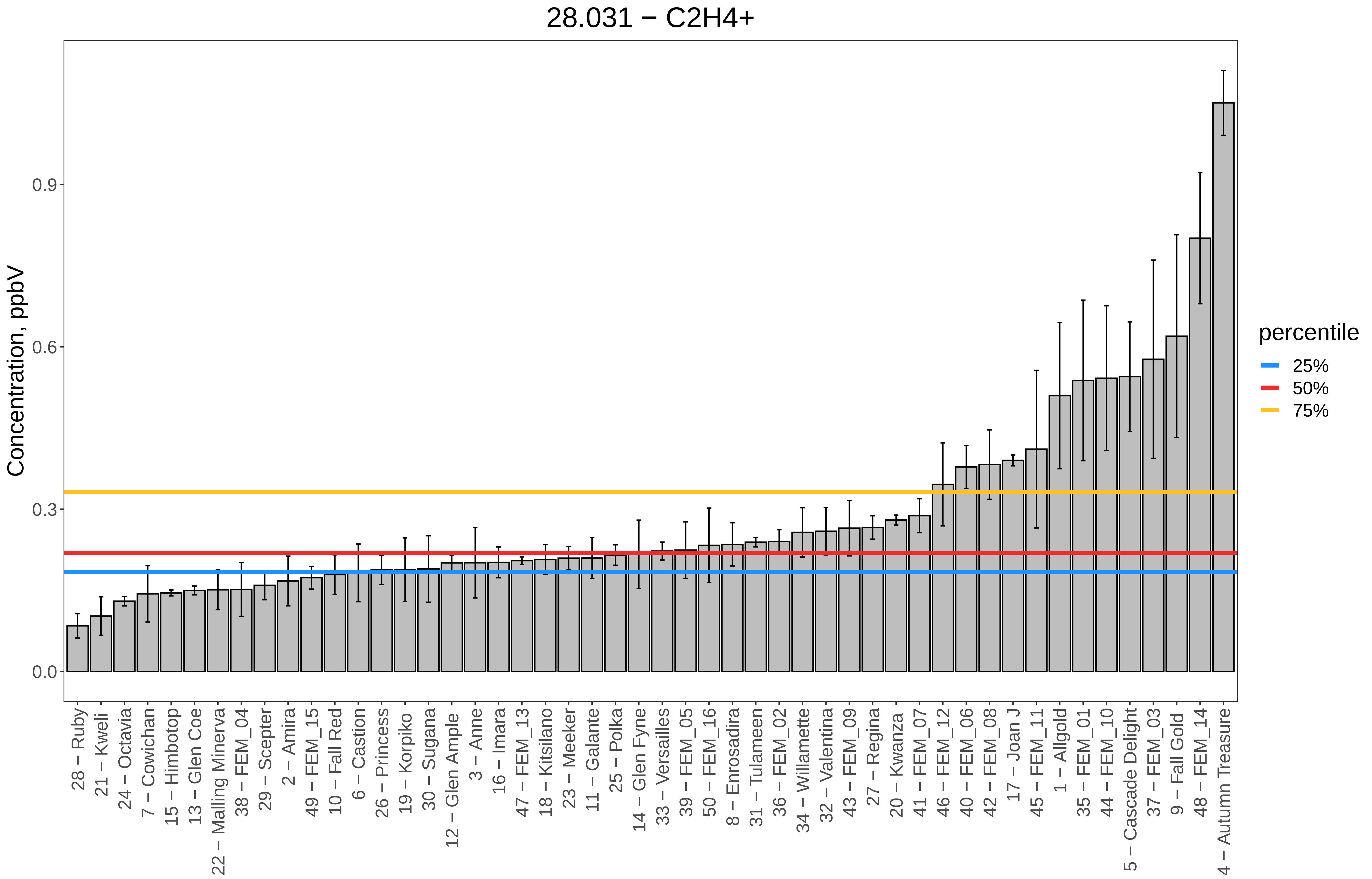

28.031 − C2H4+
Concentration, ppbV
0.0
0.3
0.6
0.9
percentile
25%
50%
75%
28 − Ruby
21 − Kweli
24 − Octavia
7 − Cowichan
15 − Himbotop
13 − Glen Coe
22 − Malling Minerva
38 − FEM_04
29 − Scepter
2 − Amira
49 − FEM_15
10 − Fall Red
6 − Castion
26 − Princess
19 − Korpiko
30 − Sugana
12 − Glen Ample
3 − Anne
16 − Imara
47 − FEM_13
18 − Kitsilano
23 − Meeker
11 − Galante
25 − Polka
14 − Glen Fyne
33 − Versailles
39 − FEM_05
50 − FEM_16
8 − Enrosadira
31 − Tulameen
36 − FEM_02
34 − Willamette
32 − Valentina
43 − FEM_09
27 − Regina
20 − Kwanza
41 − FEM_07
46 − FEM_12
40 − FEM_06
42 − FEM_08
17 − Joan J
45 − FEM_11
1 − Allgold
35 − FEM_01
44 − FEM_10
5 − Cascade Delight
37 − FEM_03
9 − Fall Gold
48 − FEM_14
4 − Autumn Treasure

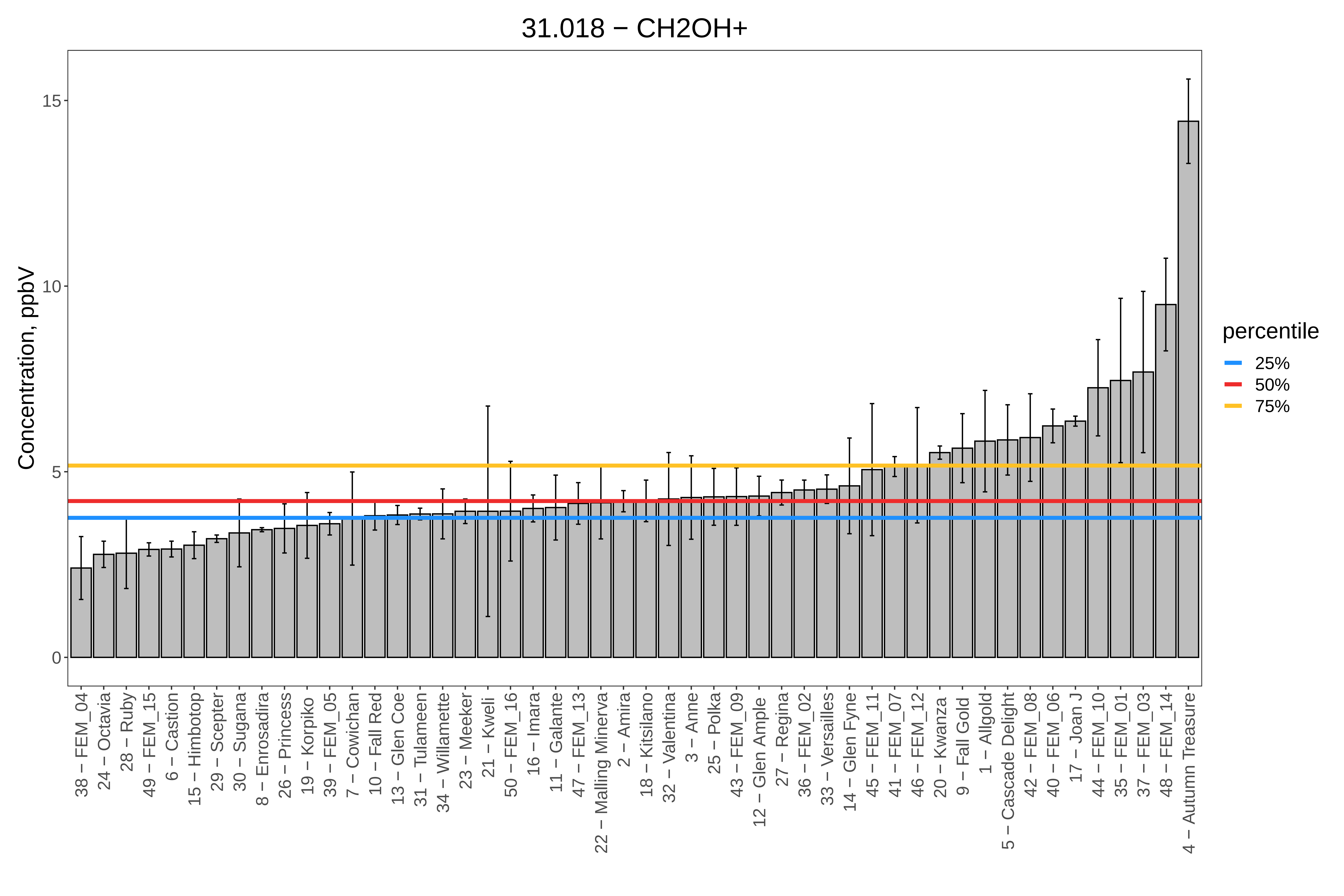

31.018 − CH2OH+
Concentration, ppbV
0
5
10
15
percentile
25%
50%
75%
38 − FEM_04
24 − Octavia
28 − Ruby
49 − FEM_15
6 − Castion
15 − Himbotop
29 − Scepter
30 − Sugana
8 − Enrosadira
26 − Princess
19 − Korpiko
39 − FEM_05
7 − Cowichan
10 − Fall Red
13 − Glen Coe
31 − Tulameen
34 − Willamette
23 − Meeker
21 − Kweli
50 − FEM_16
16 − Imara
11 − Galante
47 − FEM_13
22 − Malling Minerva
2 − Amira
18 − Kitsilano
32 − Valentina
3 − Anne
25 − Polka
43 − FEM_09
12 − Glen Ample
27 − Regina
36 − FEM_02
33 − Versailles
14 − Glen Fyne
45 − FEM_11
41 − FEM_07
46 − FEM_12
20 − Kwanza
9 − Fall Gold
1 − Allgold
5 − Cascade Delight
42 − FEM_08
40 − FEM_06
17 − Joan J
44 − FEM_10
35 − FEM_01
37 − FEM_03
48 − FEM_14
4 − Autumn Treasure

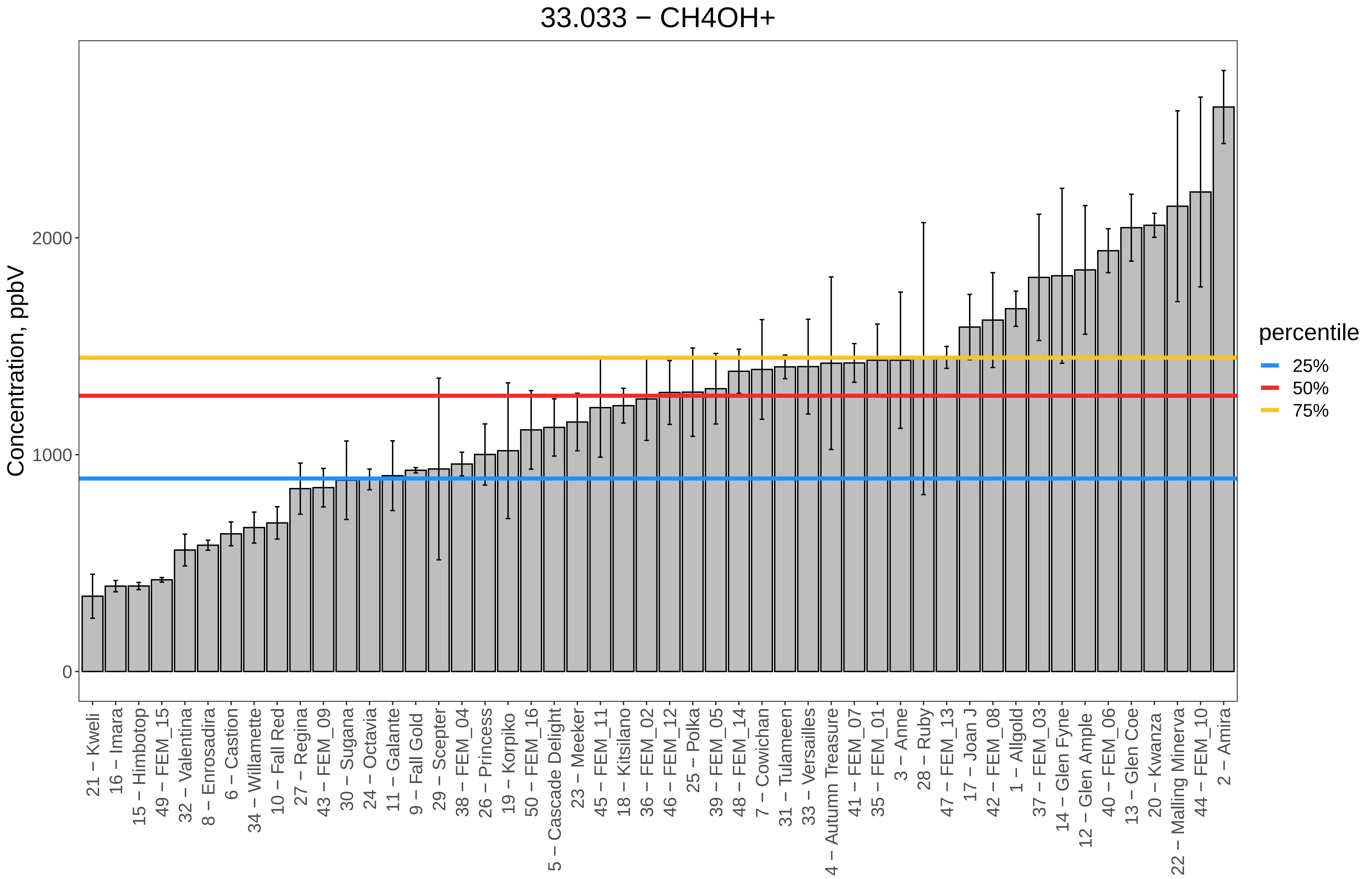

33.033 − CH4OH+
Concentration, ppbV
0
1000
2000
percentile
25%
50%
75%
21 − Kweli
16 − Imara
15 − Himbotop
49 − FEM_15
32 − Valentina
8 − Enrosadira
6 − Castion
34 − Willamette
10 − Fall Red
27 − Regina
43 − FEM_09
30 − Sugana
24 − Octavia
11 − Galante
9 − Fall Gold
29 − Scepter
38 − FEM_04
26 − Princess
19 − Korpiko
50 − FEM_16
5 − Cascade Delight
23 − Meeker
45 − FEM_11
18 − Kitsilano
36 − FEM_02
46 − FEM_12
25 − Polka
39 − FEM_05
48 − FEM_14
7 − Cowichan
31 − Tulameen
33 − Versailles
4 − Autumn Treasure
41 − FEM_07
35 − FEM_01
3 − Anne
28 − Ruby
47 − FEM_13
17 − Joan J
42 − FEM_08
1 − Allgold
37 − FEM_03
14 − Glen Fyne
12 − Glen Ample
40 − FEM_06
13 − Glen Coe
20 − Kwanza
22 − Malling Minerva
44 − FEM_10
2 − Amira

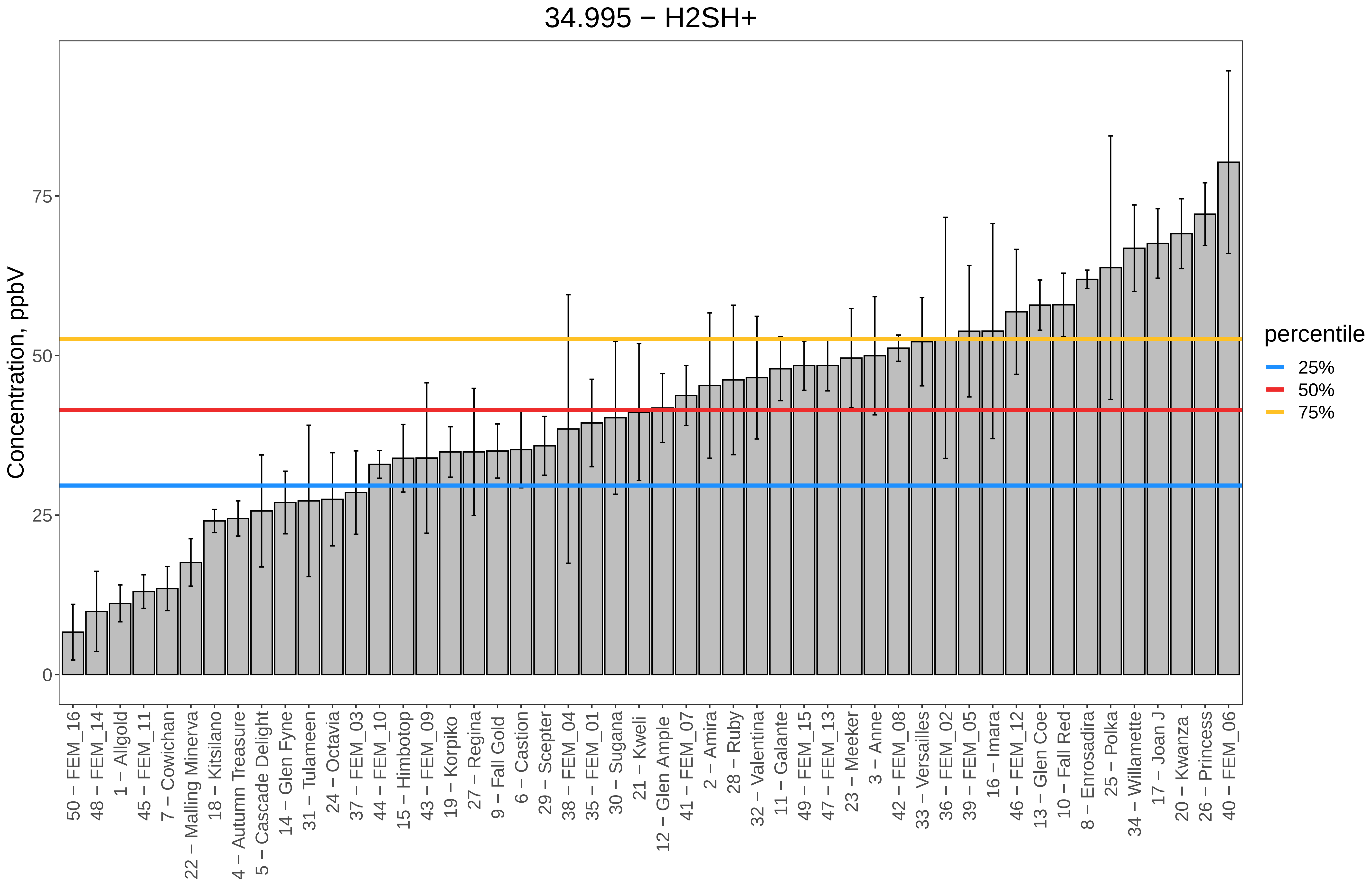
34.995 − H2SH+
Concentration, ppbV
0
25
50
75
percentile
25%
50%
75%
50 − FEM_16
48 − FEM_14
1 − Allgold
45 − FEM_11
7 − Cowichan
22 − Malling Minerva
18 − Kitsilano
4 − Autumn Treasure
5 − Cascade Delight
14 − Glen Fyne
31 − Tulameen
24 − Octavia
37 − FEM_03
44 − FEM_10
15 − Himbotop
43 − FEM_09
19 − Korpiko
27 − Regina
9 − Fall Gold
6 − Castion
29 − Scepter
38 − FEM_04
35 − FEM_01
30 − Sugana
21 − Kweli
12 − Glen Ample
41 − FEM_07
2 − Amira
28 − Ruby
32 − Valentina
11 − Galante
49 − FEM_15
47 − FEM_13
23 − Meeker
3 − Anne
42 − FEM_08
33 − Versailles
36 − FEM_02
39 − FEM_05
16 − Imara
46 − FEM_12
13 − Glen Coe
10 − Fall Red
8 − Enrosadira
25 − Polka
34 − Willamette
17 − Joan J
20 − Kwanza
26 − Princess
40 − FEM_06

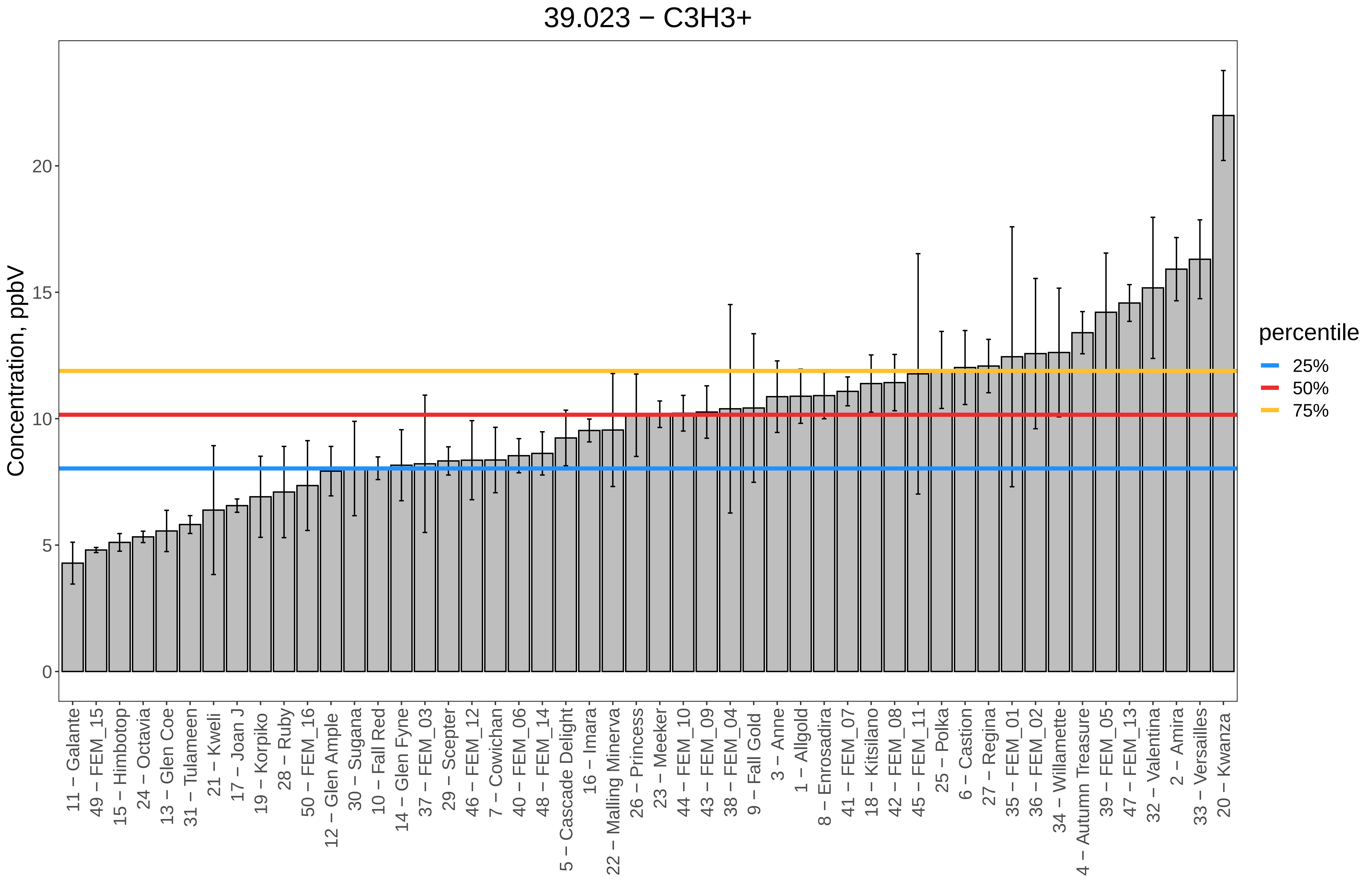

39.023 − C3H3+
Concentration, ppbV
0
5
10
15
20
percentile
25%
50%
75%
11 − Galante
49 − FEM_15
15 − Himbotop
24 − Octavia
13 − Glen Coe
31 − Tulameen
21 − Kweli
17 − Joan J
19 − Korpiko
28 − Ruby
50 − FEM_16
12 − Glen Ample
30 − Sugana
10 − Fall Red
14 − Glen Fyne
37 − FEM_03
29 − Scepter
46 − FEM_12
7 − Cowichan
40 − FEM_06
48 − FEM_14
5 − Cascade Delight
16 − Imara
22 − Malling Minerva
26 − Princess
23 − Meeker
44 − FEM_10
43 − FEM_09
38 − FEM_04
9 − Fall Gold
3 − Anne
1 − Allgold
8 − Enrosadira
41 − FEM_07
18 − Kitsilano
42 − FEM_08
45 − FEM_11
25 − Polka
6 − Castion
27 − Regina
35 − FEM_01
36 − FEM_02
34 − Willamette
4 − Autumn Treasure
39 − FEM_05
47 − FEM_13
32 − Valentina
2 − Amira
33 − Versailles
20 − Kwanza

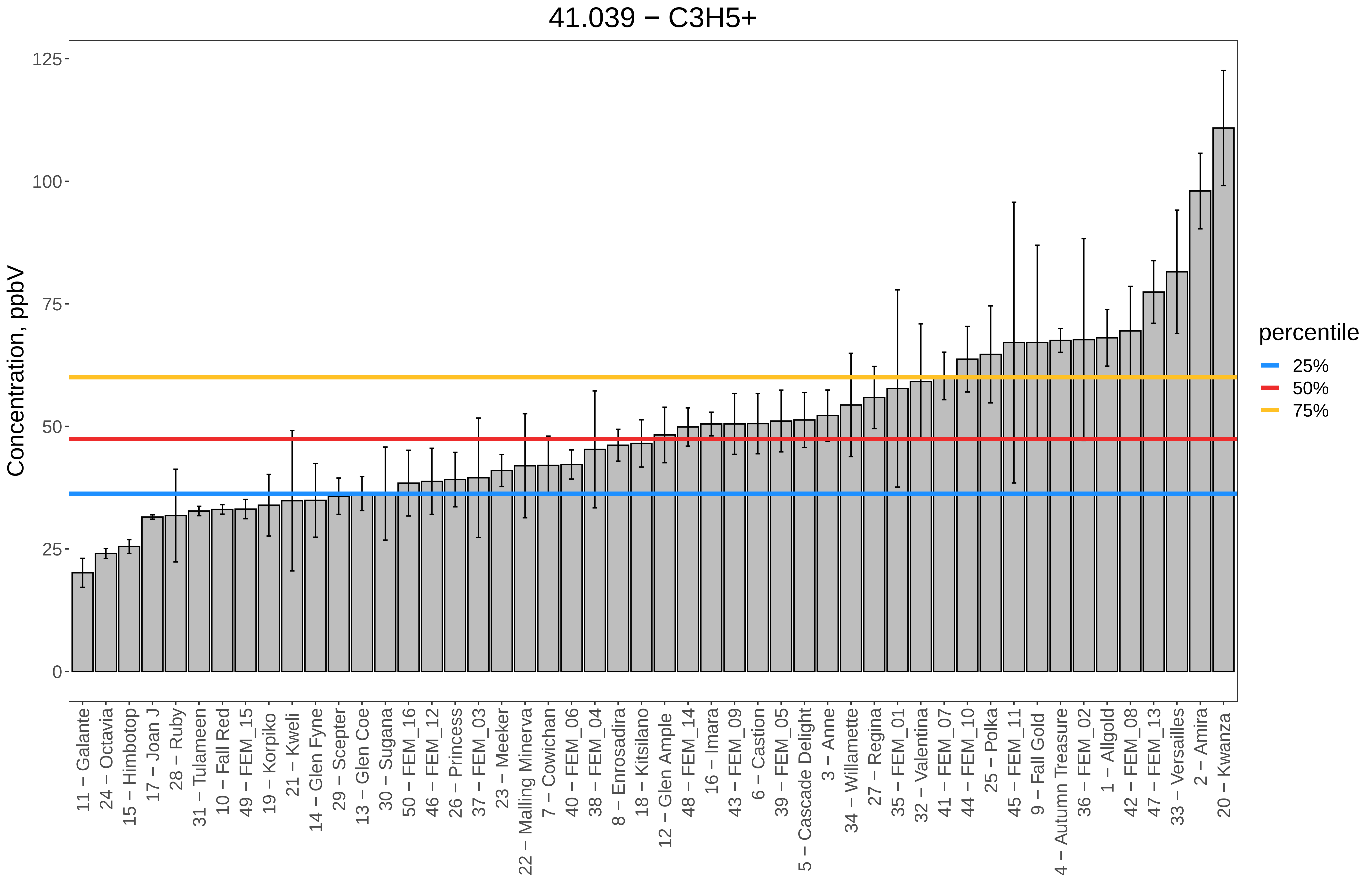
41.039 − C3H5+
Concentration, ppbV
0
25
50
75
100
125
percentile
25%
50%
75%
11 − Galante
24 − Octavia
15 − Himbotop
17 − Joan J
28 − Ruby
31 − Tulameen
10 − Fall Red
49 − FEM_15
19 − Korpiko
21 − Kweli
14 − Glen Fyne
29 − Scepter
13 − Glen Coe
30 − Sugana
50 − FEM_16
46 − FEM_12
26 − Princess
37 − FEM_03
23 − Meeker
22 − Malling Minerva
7 − Cowichan
40 − FEM_06
38 − FEM_04
8 − Enrosadira
18 − Kitsilano
12 − Glen Ample
48 − FEM_14
16 − Imara
43 − FEM_09
6 − Castion
39 − FEM_05
5 − Cascade Delight
3 − Anne
34 − Willamette
27 − Regina
35 − FEM_01
32 − Valentina
41 − FEM_07
44 − FEM_10
25 − Polka
45 − FEM_11
9 − Fall Gold
4 − Autumn Treasure
36 − FEM_02
1 − Allgold
42 − FEM_08
47 − FEM_13
33 − Versailles
2 − Amira
20 − Kwanza

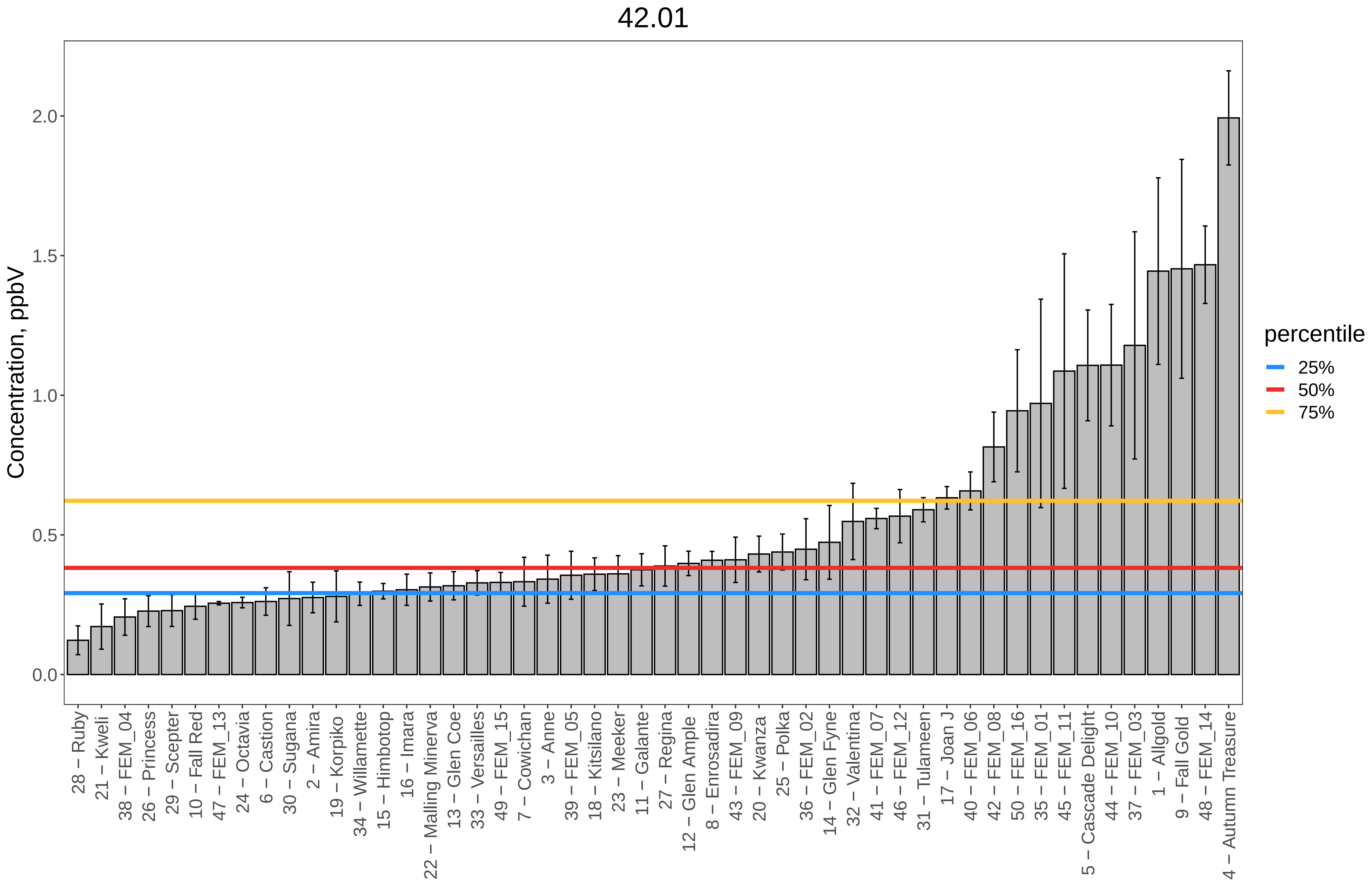

42.01
Concentration, ppbV
0.0
0.5
1.0
1.5
2.0
percentile
25%
50%
75%
28 – Ruby
21 – Kweli
38 – FEM_04
26 – Princess
29 – Scepter
10 – Fall Red
47 – FEM_13
24 – Octavia
6 – Castion
30 – Sugana
2 – Amira
19 – Korpiko
34 – Willamette
15 – Himbotop
16 – Imara
22 – Malling Minerva
13 – Glen Coe
33 – Versailles
49 – FEM_15
7 – Cowichan
3 – Anne
39 – FEM_05
18 – Kitsilano
23 – Meeker
11 – Galante
27 – Regina
12 – Glen Ample
8 – Enrosadira
43 – FEM_09
20 – Kwanza
25 – Polka
36 – FEM_02
14 – Glen Fyne
32 – Valentina
41 – FEM_07
46 – FEM_12
31 – Tulameen
17 – Joan J
40 – FEM_06
42 – FEM_08
50 – FEM_16
35 – FEM_01
45 – FEM_11
5 – Cascade Delight
44 – FEM_10
37 – FEM_03
1 – Allgold
9 – Fall Gold
48 – FEM_14
4 – Autumn Treasure

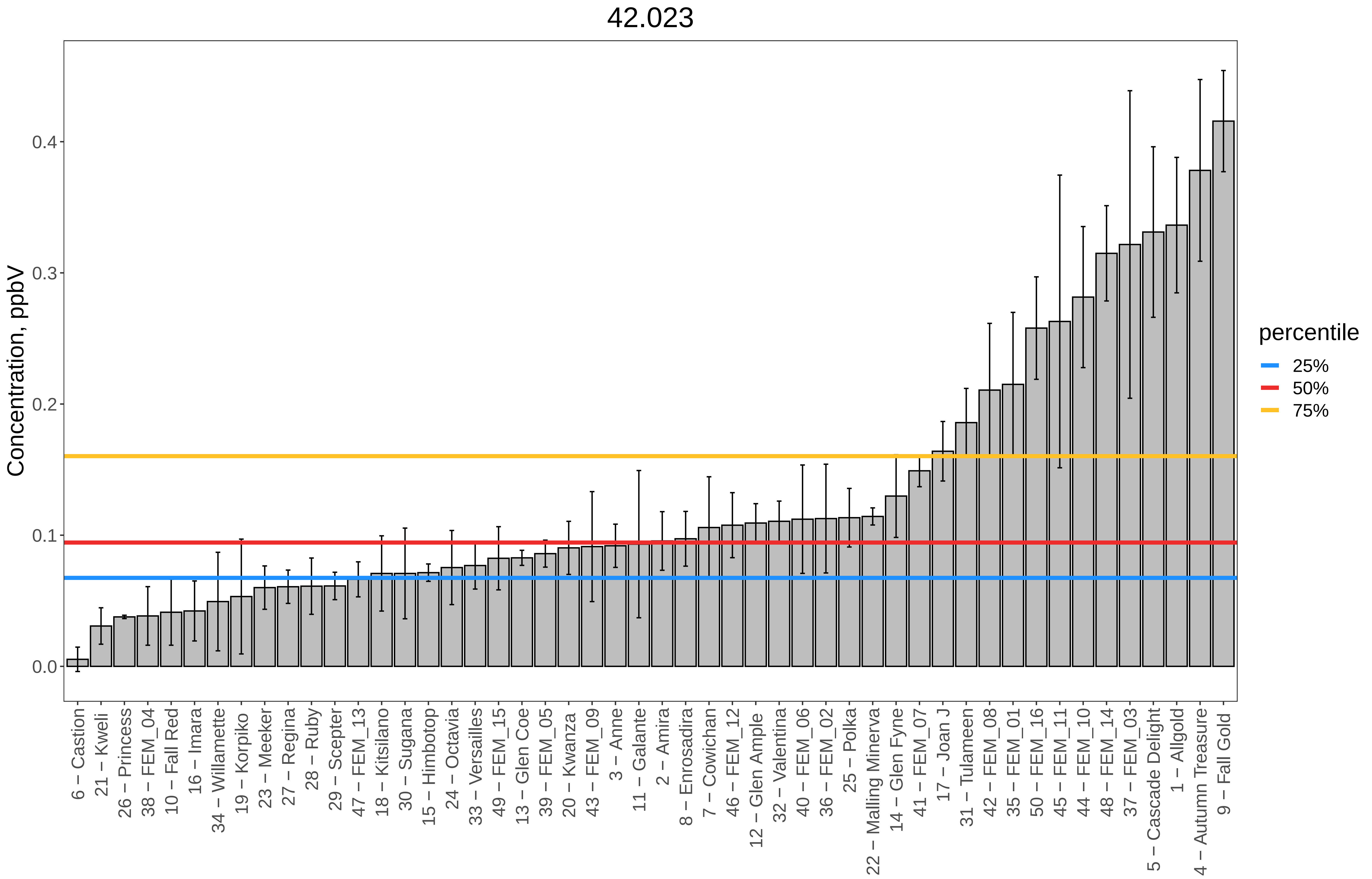
42.023
Concentration, ppbV
0.0
0.1
0.2
0.3
0.4
percentile
25%
50%
75%
6 – Castion
21 – Kweli
26 – Princess
38 – FEM_04
10 – Fall Red
16 – Imara
34 – Willamette
19 – Korpiko
23 – Meeker
27 – Regina
28 – Ruby
29 – Scepter
47 – FEM_13
18 – Kitsilano
30 – Sugana
15 – Himbotop
24 – Octavia
33 – Versailles
49 – FEM_15
13 – Glen Coe
39 – FEM_05
20 – Kwanza
43 – FEM_09
3 – Anne
11 – Galante
2 – Amira
8 – Enrosadira
7 – Cowichan
46 – FEM_12
12 – Glen Ample
32 – Valentina
40 – FEM_06
36 – FEM_02
25 – Polka
22 – Malling Minerva
14 – Glen Fyne
41 – FEM_07
17 – Joan J
31 – Tulameen
42 – FEM_08
35 – FEM_01
50 – FEM_16
45 – FEM_11
44 – FEM_10
48 – FEM_14
37 – FEM_03
5 – Cascade Delight
1 – Allgold
4 – Autumn Treasure
9 – Fall Gold

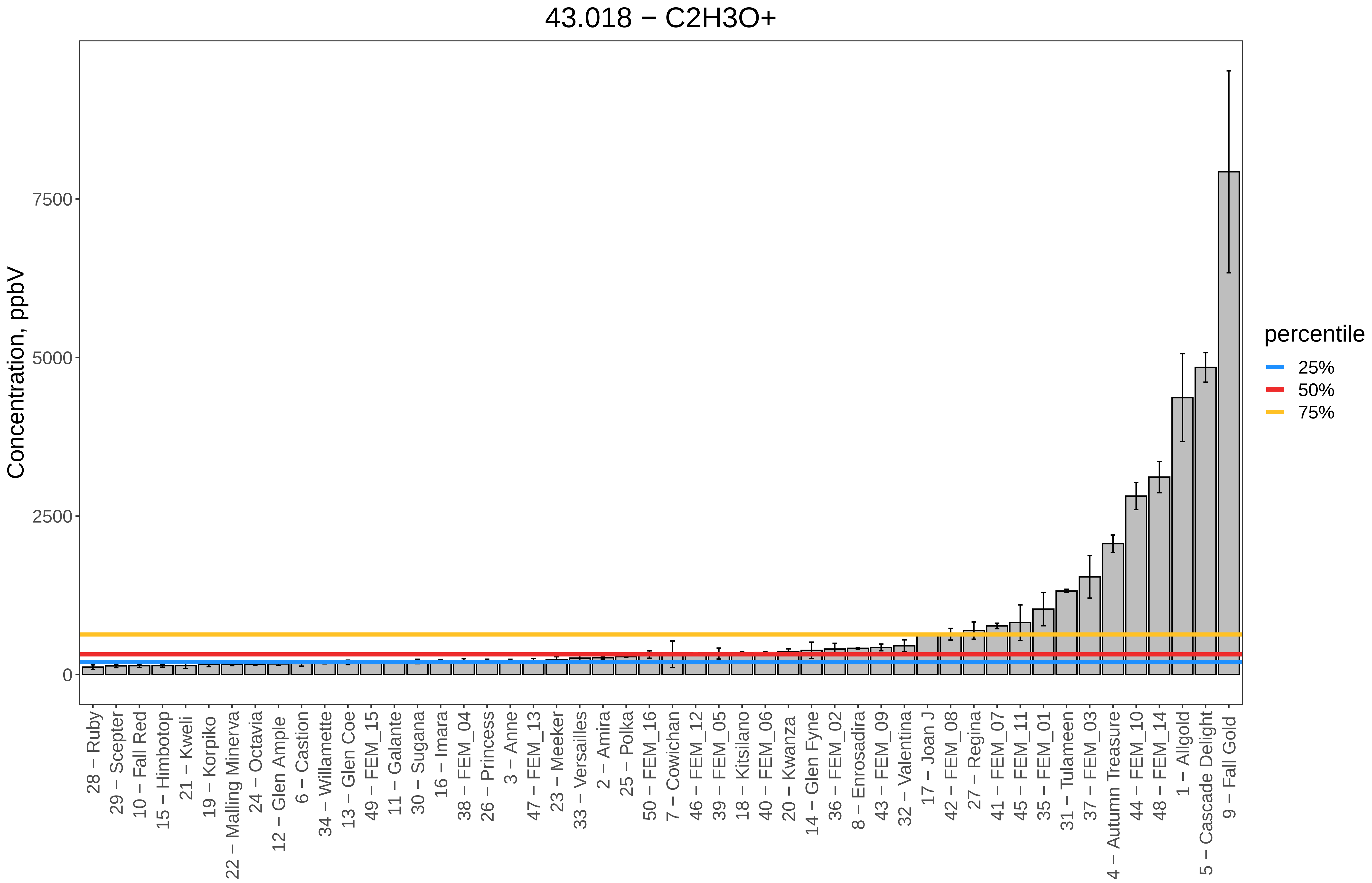

43.018 − C2H3O+
Concentration, ppbV
0
2500
5000
7500
percentile
25%
50%
75%
28 − Ruby
29 − Scepter
10 − Fall Red
15 − Himbotop
21 − Kweli
19 − Korpiko
22 − Malling Minerva
24 − Octavia
12 − Glen Ample
6 − Castion
34 − Willamette
13 − Glen Coe
49 − FEM_15
11 − Galante
30 − Sugana
16 − Imara
38 − FEM_04
26 − Princess
3 − Anne
47 − FEM_13
23 − Meeker
33 − Versailles
2 − Amira
25 − Polka
50 − FEM_16
7 − Cowichan
46 − FEM_12
39 − FEM_05
18 − Kitsilano
40 − FEM_06
20 − Kwanza
14 − Glen Fyne
36 − FEM_02
8 − Enrosadira
43 − FEM_09
32 − Valentina
17 − Joan J
42 − FEM_08
27 − Regina
41 − FEM_07
45 − FEM_11
35 − FEM_01
31 − Tulameen
37 − FEM_03
4 − Autumn Treasure
44 − FEM_10
48 − FEM_14
1 − Allgold
5 − Cascade Delight
9 − Fall Gold

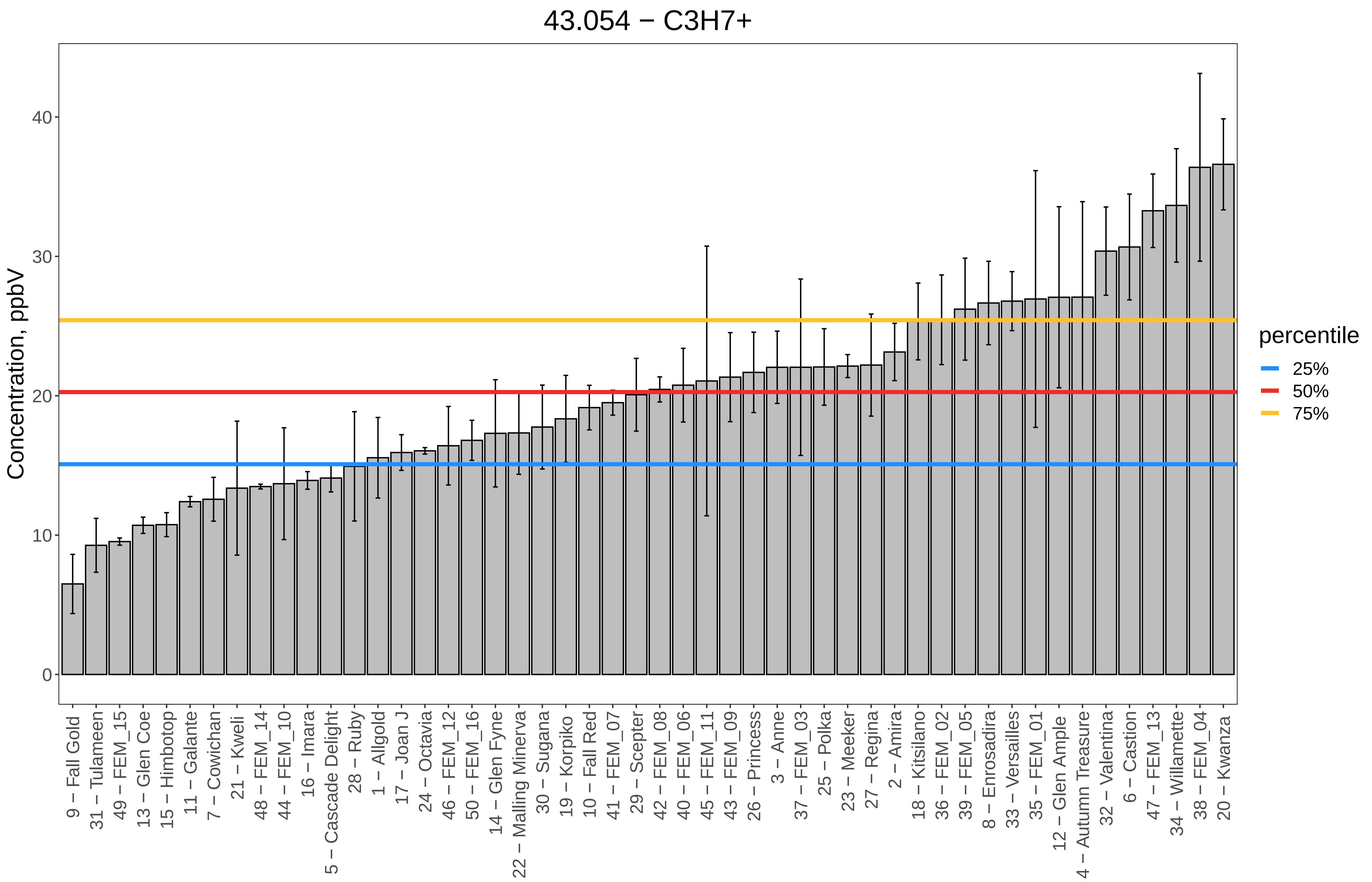

43.054 − C3H7+
Concentration, ppbV
0
10
20
30
40
percentile
25%
50%
75%
9 − Fall Gold
31 − Tulameen
49 − FEM_15
13 − Glen Coe
15 − Himbotop
11 − Galante
7 − Cowichan
21 − Kweli
48 − FEM_14
44 − FEM_10
16 − Imara
5 − Cascade Delight
28 − Ruby
1 − Allgold
17 − Joan J
24 − Octavia
46 − FEM_12
50 − FEM_16
14 − Glen Fyne
22 − Malling Minerva
30 − Sugana
19 − Korpiko
10 − Fall Red
41 − FEM_07
29 − Scepter
42 − FEM_08
40 − FEM_06
45 − FEM_11
43 − FEM_09
26 − Princess
3 − Anne
37 − FEM_03
25 − Polka
23 − Meeker
27 − Regina
2 − Amira
18 − Kitsilano
36 − FEM_02
39 − FEM_05
8 − Enrosadira
33 − Versailles
35 − FEM_01
12 − Glen Ample
4 − Autumn Treasure
32 − Valentina
6 − Castion
47 − FEM_13
34 − Willamette
38 − FEM_04
20 − Kwanza

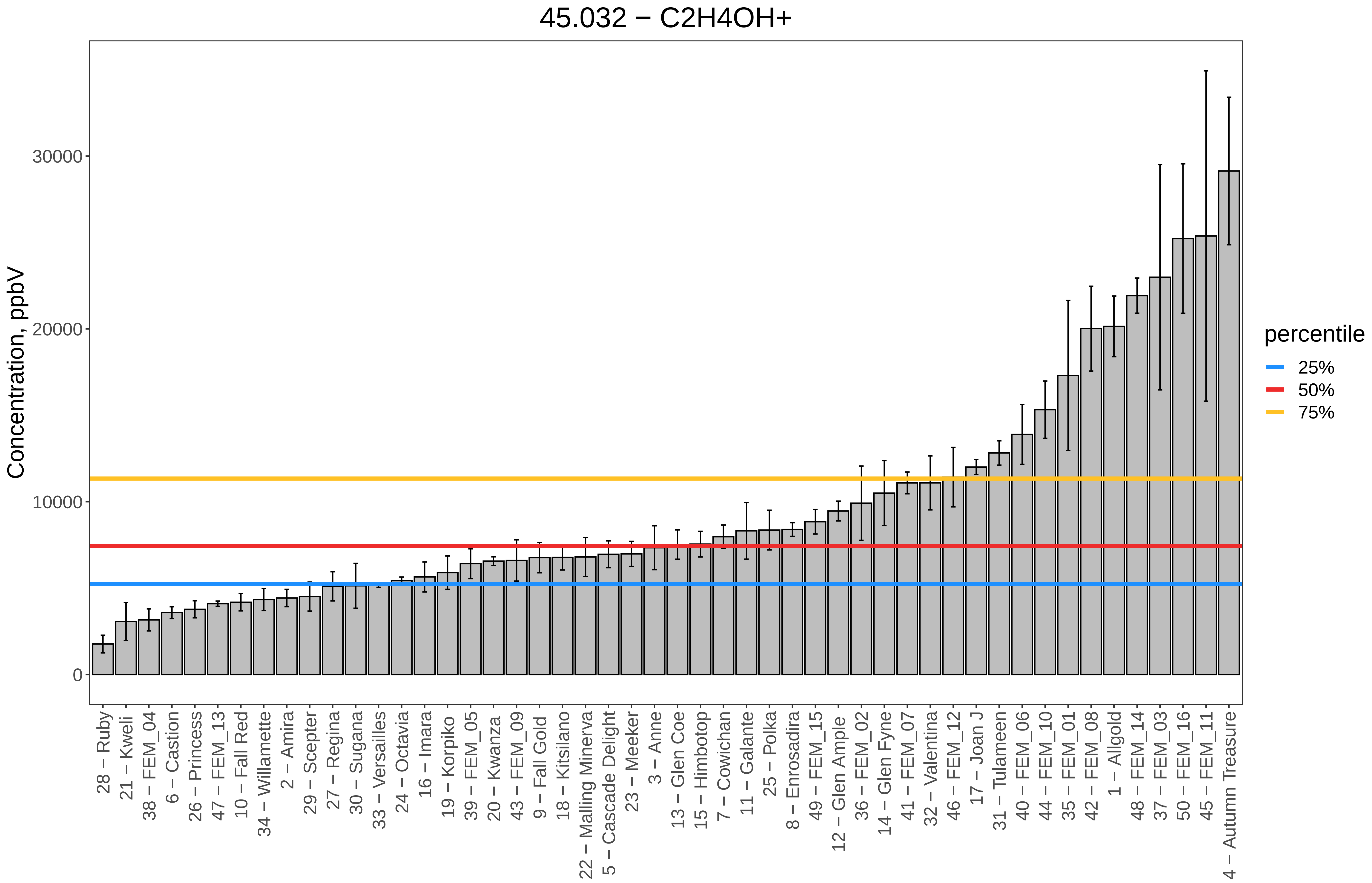
45.032 − C2H4OH+
Concentration, ppbV
0
10000
20000
30000
percentile
25%
50%
75%
28 − Ruby
21 − Kweli
38 − FEM_04
6 − Castion
26 − Princess
47 − FEM_13
10 − Fall Red
34 − Willamette
2 − Amira
29 − Scepter
27 − Regina
30 − Sugana
33 − Versailles
24 − Octavia
16 − Imara
19 − Korpiko
39 − FEM_05
20 − Kwanza
43 − FEM_09
9 − Fall Gold
18 − Kitsilano
22 − Malling Minerva
5 − Cascade Delight
23 − Meeker
3 − Anne
13 − Glen Coe
15 − Himbotop
7 − Cowichan
11 − Galante
25 − Polka
8 − Enrosadira
49 − FEM_15
12 − Glen Ample
36 − FEM_02
14 − Glen Fyne
41 − FEM_07
32 − Valentina
46 − FEM_12
17 − Joan J
31 − Tulameen
40 − FEM_06
44 − FEM_10
35 − FEM_01
42 − FEM_08
1 − Allgold
48 − FEM_14
37 − FEM_03
50 − FEM_16
45 − FEM_11
4 − Autumn Treasure

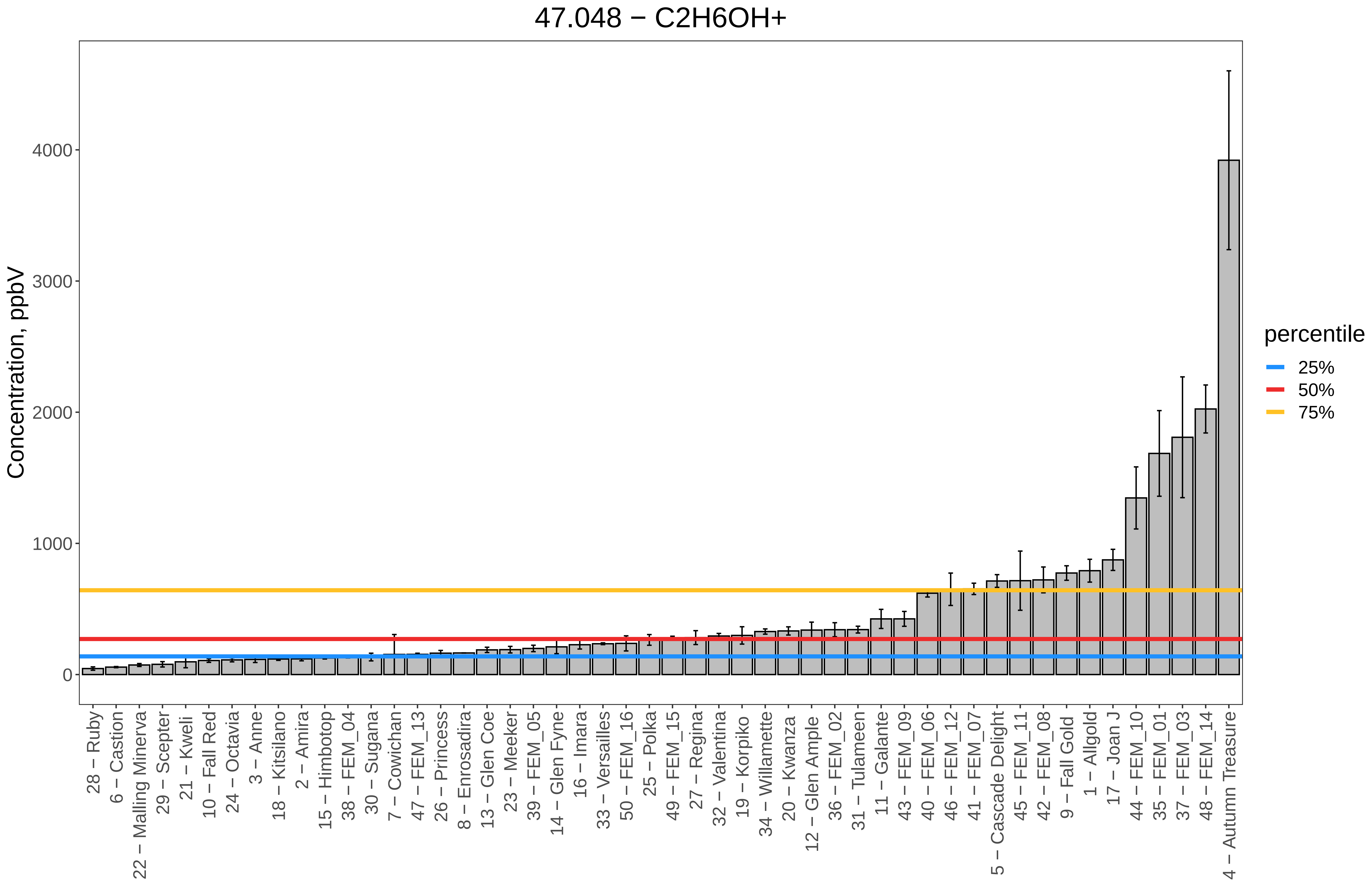

47.048 − C2H6OH+
Concentration, ppbV
0
1000
2000
3000
4000
percentile
25%
50%
75%
28 − Ruby
6 − Castion
22 − Malling Minerva
29 − Scepter
21 − Kweli
10 − Fall Red
24 − Octavia
3 − Anne
18 − Kitsilano
2 − Amira
15 − Himbotop
38 − FEM_04
30 − Sugana
7 − Cowichan
47 − FEM_13
26 − Princess
8 − Enrosadira
13 − Glen Coe
23 − Meeker
39 − FEM_05
14 − Glen Fyne
16 − Imara
33 − Versailles
50 − FEM_16
25 − Polka
49 − FEM_15
27 − Regina
32 − Valentina
19 − Korpiko
34 − Willamette
20 − Kwanza
12 − Glen Ample
36 − FEM_02
31 − Tulameen
11 − Galante
43 − FEM_09
40 − FEM_06
46 − FEM_12
41 − FEM_07
5 − Cascade Delight
45 − FEM_11
42 − FEM_08
9 − Fall Gold
1 − Allgold
17 − Joan J
44 − FEM_10
35 − FEM_01
37 − FEM_03
48 − FEM_14
4 − Autumn Treasure

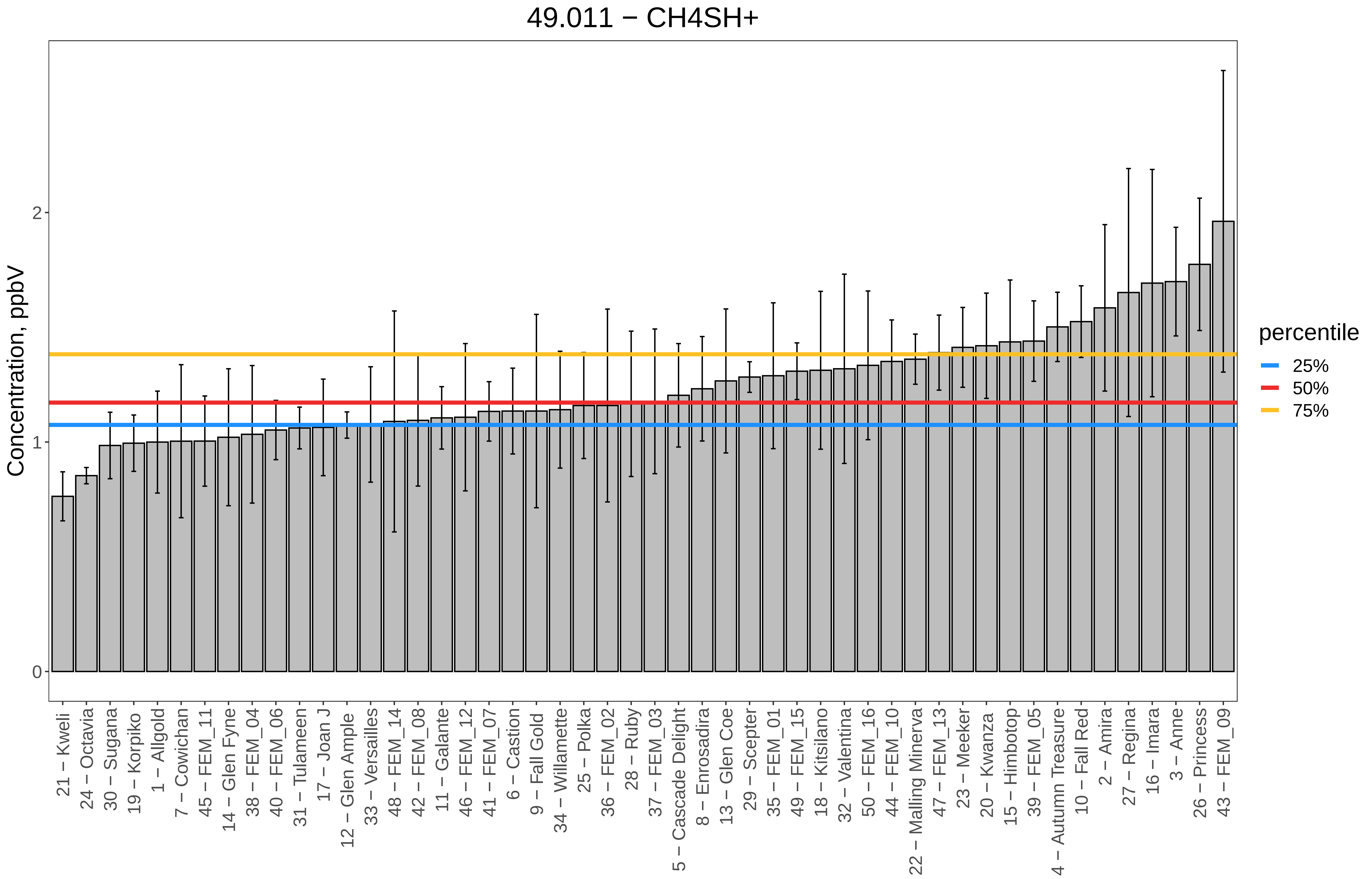

49.011 − CH4SH+
Concentration, ppbV
0
1
2
percentile
25%
50%
75%
21 − Kweli
24 − Octavia
30 − Sugana
19 − Korpiko
1 − Allgold
7 − Cowichan
45 − FEM_11
14 − Glen Fyne
38 − FEM_04
40 − FEM_06
31 − Tulameen
17 − Joan J
12 − Glen Ample
33 − Versailles
48 − FEM_14
42 − FEM_08
11 − Galante
46 − FEM_12
41 − FEM_07
6 − Castion
9 − Fall Gold
34 − Willamette
25 − Polka
36 − FEM_02
28 − Ruby
37 − FEM_03
5 − Cascade Delight
8 − Enrosadira
13 − Glen Coe
29 − Scepter
35 − FEM_01
49 − FEM_15
18 − Kitsilano
32 − Valentina
50 − FEM_16
44 − FEM_10
22 − Malling Minerva
47 − FEM_13
23 − Meeker
20 − Kwanza
15 − Himbotop
39 − FEM_05
4 − Autumn Treasure
10 − Fall Red
2 − Amira
27 − Regina
16 − Imara
3 − Anne
26 − Princess
43 − FEM_09

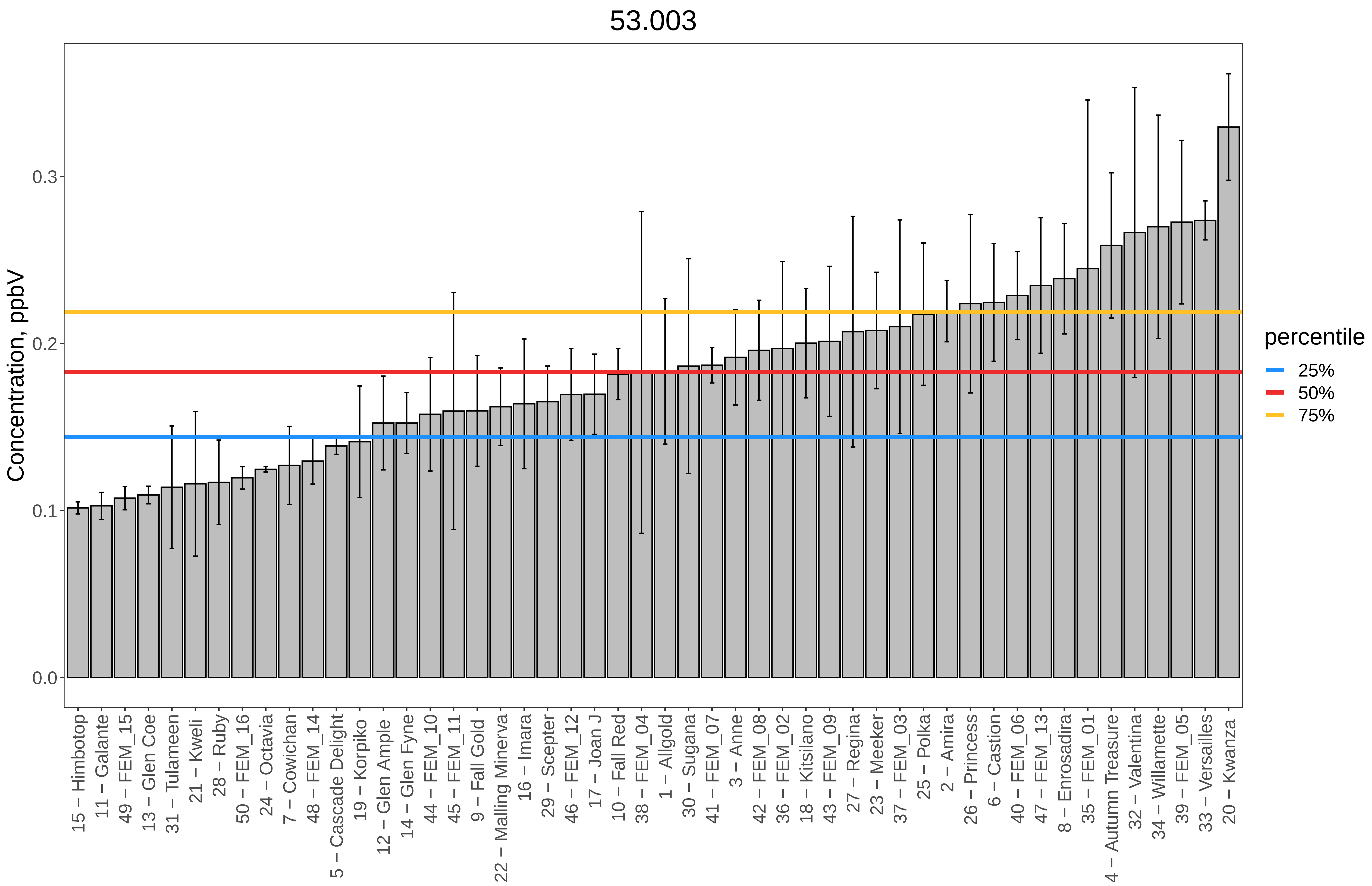

53.003
Concentration, ppbV
0.0
0.1
0.2
0.3
percentile
25%
50%
75%
15 – Himbotop
11 – Galante
49 – FEM_15
13 – Glen Coe
31 – Tulameen
21 – Kweli
28 – Ruby
50 – FEM_16
24 – Octavia
7 – Cowichan
48 – FEM_14
5 – Cascade Delight
19 – Korpiko
12 – Glen Ample
14 – Glen Fyne
44 – FEM_10
45 – FEM_11
9 – Fall Gold
22 – Malling Minerva
16 – Imara
29 – Scepter
46 – FEM_12
17 – Joan J
10 – Fall Red
38 – FEM_04
1 – Allgold
30 – Sugana
41 – FEM_07
3 – Anne
42 – FEM_08
36 – FEM_02
18 – Kitsilano
43 – FEM_09
27 – Regina
23 – Meeker
37 – FEM_03
25 – Polka
2 – Amira
26 – Princess
6 – Castion
40 – FEM_06
47 – FEM_13
8 – Enrosadira
35 – FEM_01
4 – Autumn Treasure
32 – Valentina
34 – Willamette
39 – FEM_05
33 – Versailles
20 – Kwanza

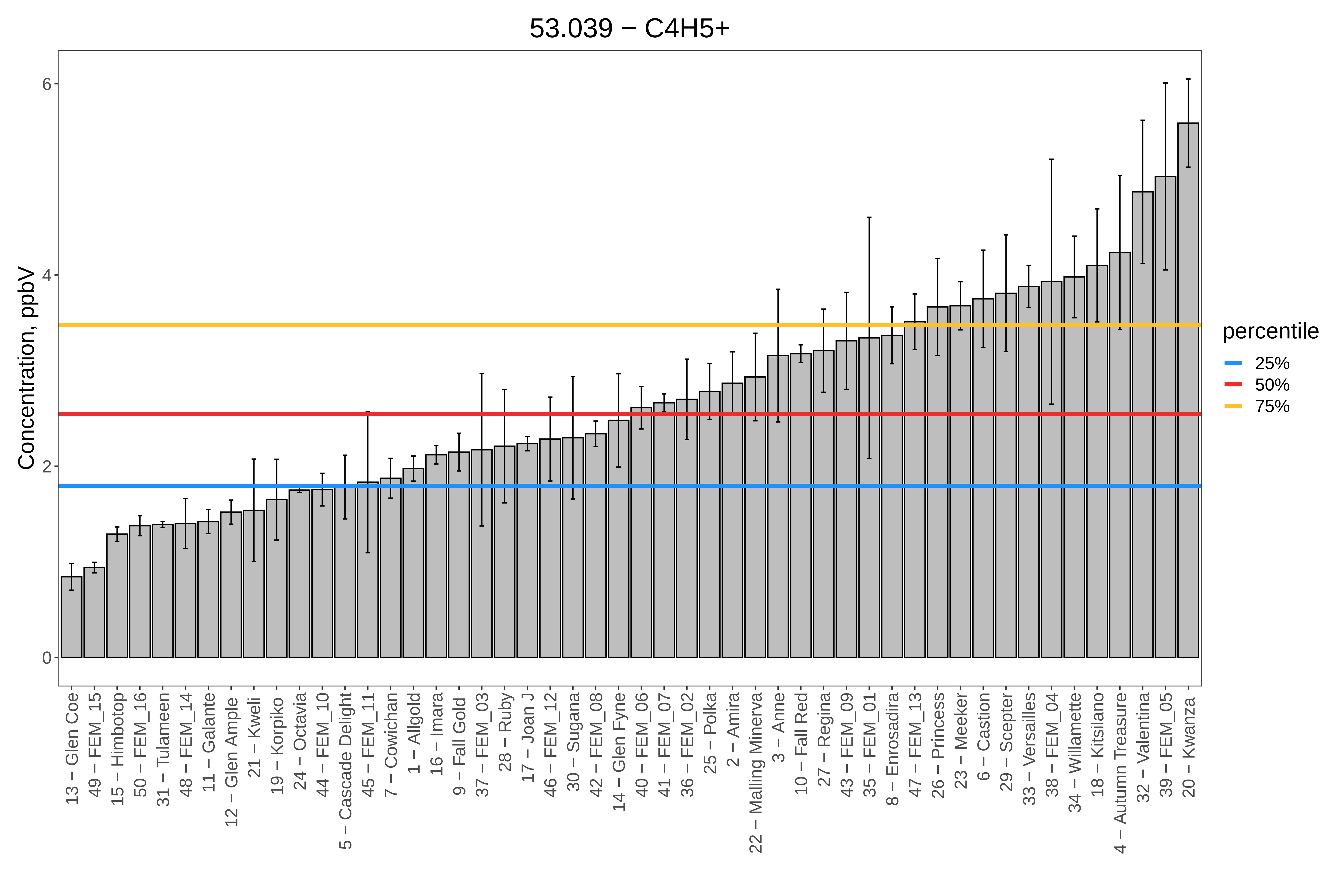
53.039 − C4H5+
Concentration, ppbV
0
2
4
6
percentile
25%
50%
75%
13 − Glen Coe
49 − FEM_15
15 − Himbotop
50 − FEM_16
31 − Tulameen
48 − FEM_14
11 − Galante
12 − Glen Ample
21 − Kweli
19 − Korpiko
24 − Octavia
44 − FEM_10
5 − Cascade Delight
45 − FEM_11
7 − Cowichan
1 − Allgold
16 − Imara
9 − Fall Gold
37 − FEM_03
28 − Ruby
17 − Joan J
46 − FEM_12
30 − Sugana
42 − FEM_08
14 − Glen Fyne
40 − FEM_06
41 − FEM_07
36 − FEM_02
25 − Polka
2 − Amira
22 − Malling Minerva
3 − Anne
10 − Fall Red
27 − Regina
43 − FEM_09
35 − FEM_01
8 − Enrosadira
47 − FEM_13
26 − Princess
23 − Meeker
6 − Castion
29 − Scepter
33 − Versailles
38 − FEM_04
34 − Willamette
18 − Kitsilano
4 − Autumn Treasure
32 − Valentina
39 − FEM_05
20 − Kwanza

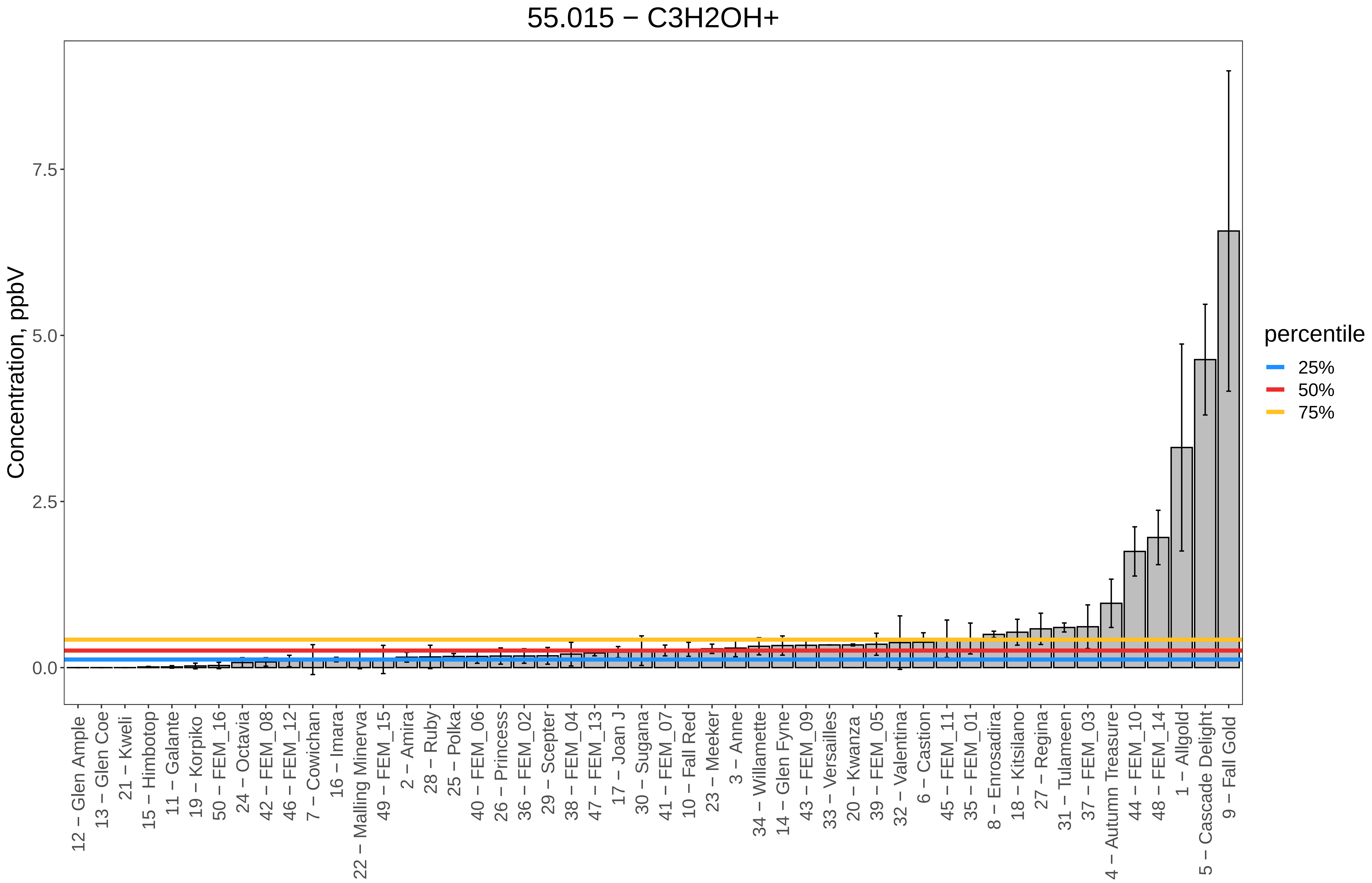
55.015 − C3H2OH+
Concentration, ppbV
0.0
2.5
5.0
7.5
percentile
25%
50%
75%
12 − Glen Ample
13 − Glen Coe
21 − Kweli
15 − Himbotop
11 − Galante
19 − Korpiko
50 − FEM_16
24 − Octavia
42 − FEM_08
46 − FEM_12
7 − Cowichan
16 − Imara
22 − Malling Minerva
49 − FEM_15
2 − Amira
28 − Ruby
25 − Polka
40 − FEM_06
26 − Princess
36 − FEM_02
29 − Scepter
38 − FEM_04
47 − FEM_13
17 − Joan J
30 − Sugana
41 − FEM_07
10 − Fall Red
23 − Meeker
3 − Anne
34 − Willamette
14 − Glen Fyne
43 − FEM_09
33 − Versailles
20 − Kwanza
39 − FEM_05
32 − Valentina
6 − Castion
45 − FEM_11
35 − FEM_01
8 − Enrosadira
18 − Kitsilano
27 − Regina
31 − Tulameen
37 − FEM_03
4 − Autumn Treasure
44 − FEM_10
48 − FEM_14
1 − Allgold
5 − Cascade Delight
9 − Fall Gold

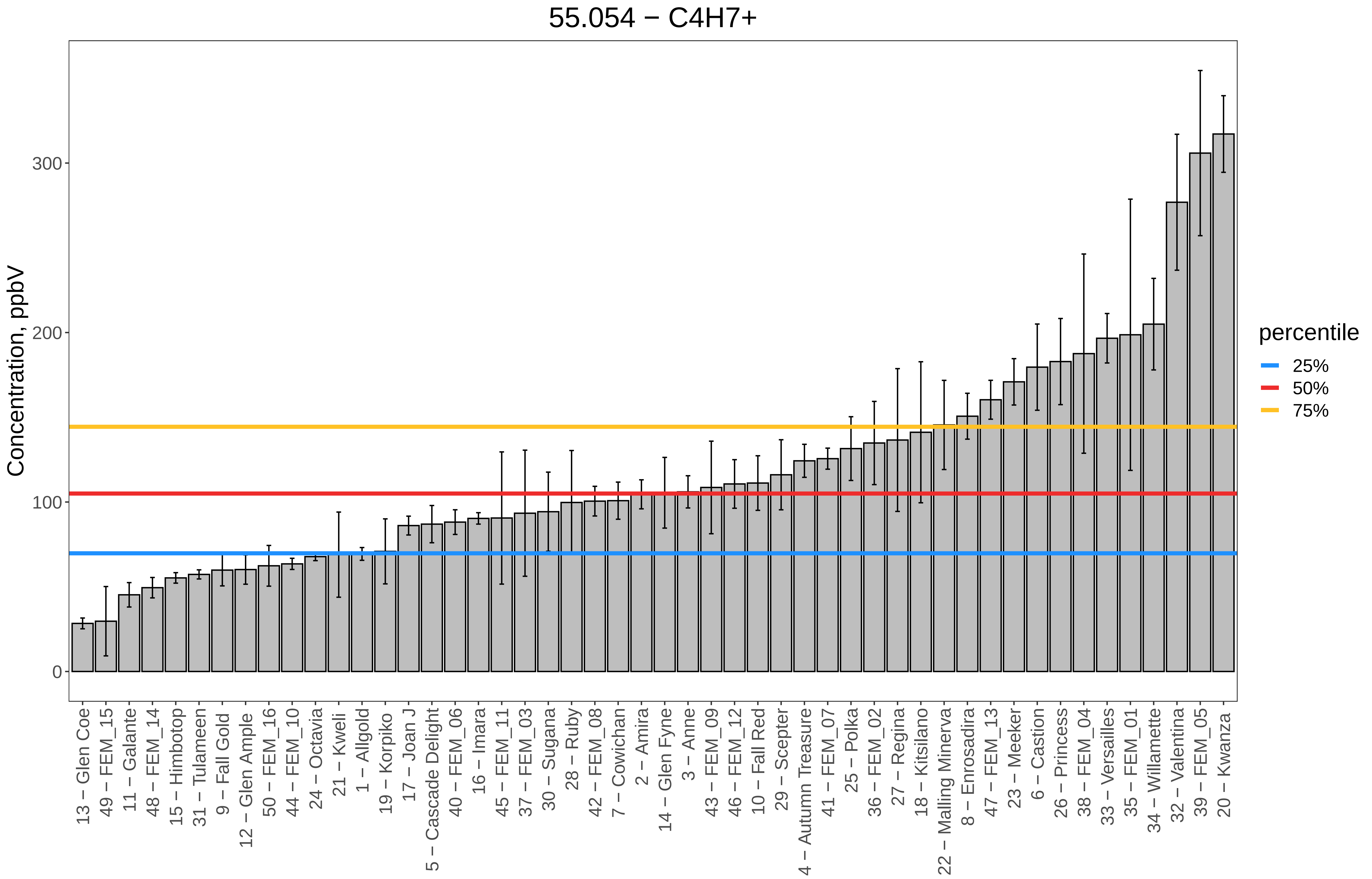

55.054 − C4H7+
Concentration, ppbV
0
100
200
300
percentile
25%
50%
75%
13 − Glen Coe
49 − FEM_15
11 − Galante
48 − FEM_14
15 − Himbotop
31 − Tulameen
9 − Fall Gold
12 − Glen Ample
50 − FEM_16
44 − FEM_10
24 − Octavia
21 − Kweli
1 − Allgold
19 − Korpiko
17 − Joan J
5 − Cascade Delight
40 − FEM_06
16 − Imara
45 − FEM_11
37 − FEM_03
30 − Sugana
28 − Ruby
42 − FEM_08
7 − Cowichan
2 − Amira
14 − Glen Fyne
3 − Anne
43 − FEM_09
46 − FEM_12
10 − Fall Red
29 − Scepter
4 − Autumn Treasure
41 − FEM_07
25 − Polka
36 − FEM_02
27 − Regina
18 − Kitsilano
22 − Malling Minerva
8 − Enrosadira
47 − FEM_13
23 − Meeker
6 − Castion
26 − Princess
38 − FEM_04
33 − Versailles
35 − FEM_01
34 − Willamette
32 − Valentina
39 − FEM_05
20 − Kwanza

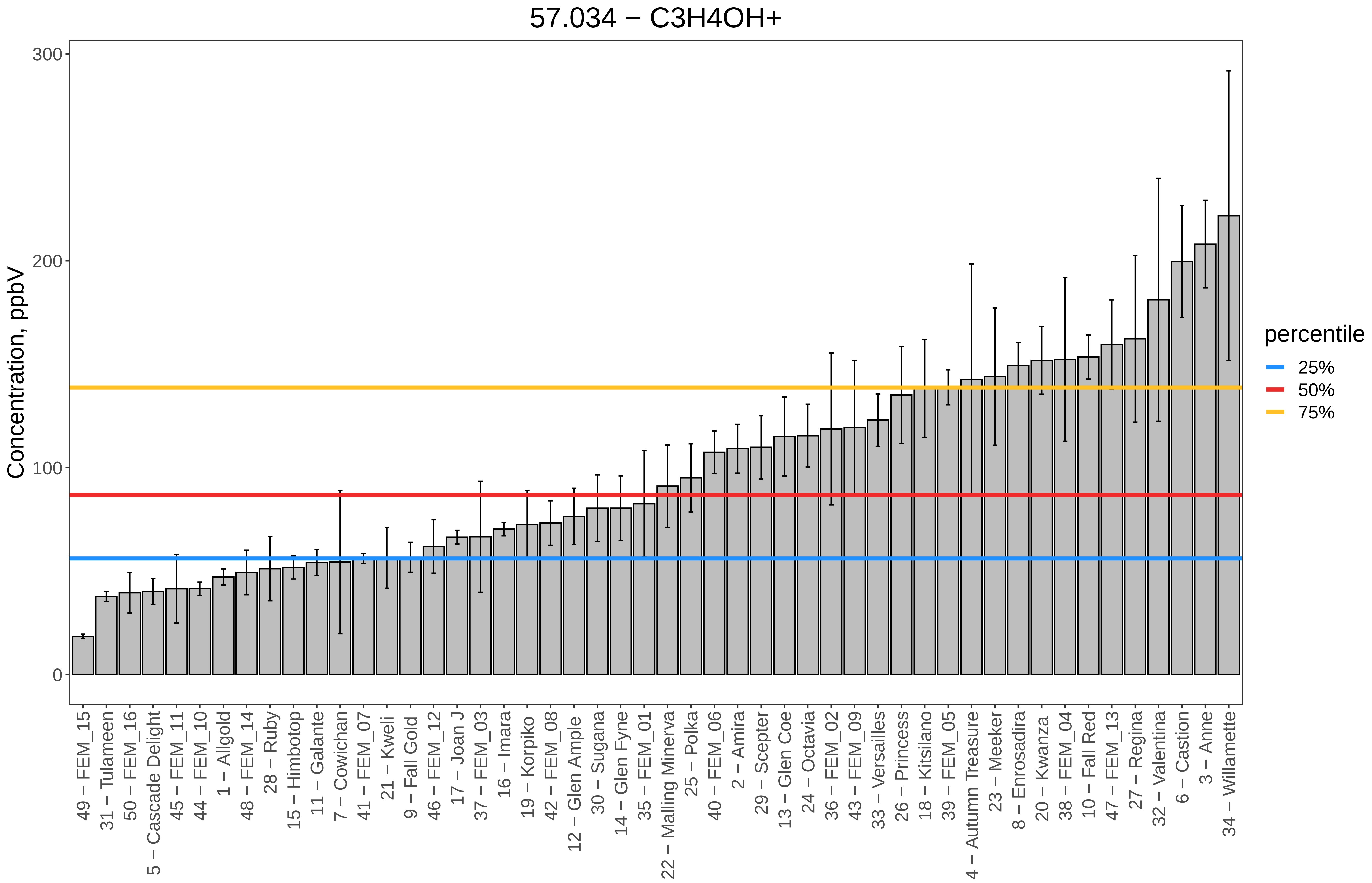

57.034 − C3H4OH+
Concentration, ppbV
0
100
200
300
percentile
25%
50%
75%
49 − FEM_15
31 − Tulameen
50 − FEM_16
5 − Cascade Delight
45 − FEM_11
44 − FEM_10
1 − Allgold
48 − FEM_14
28 − Ruby
15 − Himbotop
11 − Galante
7 − Cowichan
41 − FEM_07
21 − Kweli
9 − Fall Gold
46 − FEM_12
17 − Joan J
37 − FEM_03
16 − Imara
19 − Korpiko
42 − FEM_08
12 − Glen Ample
30 − Sugana
14 − Glen Fyne
35 − FEM_01
22 − Malling Minerva
25 − Polka
40 − FEM_06
2 − Amira
29 − Scepter
13 − Glen Coe
24 − Octavia
36 − FEM_02
43 − FEM_09
33 − Versailles
26 − Princess
18 − Kitsilano
39 − FEM_05
4 − Autumn Treasure
23 − Meeker
8 − Enrosadira
20 − Kwanza
38 − FEM_04
10 − Fall Red
47 − FEM_13
27 − Regina
32 − Valentina
6 − Castion
3 − Anne
34 − Willamette

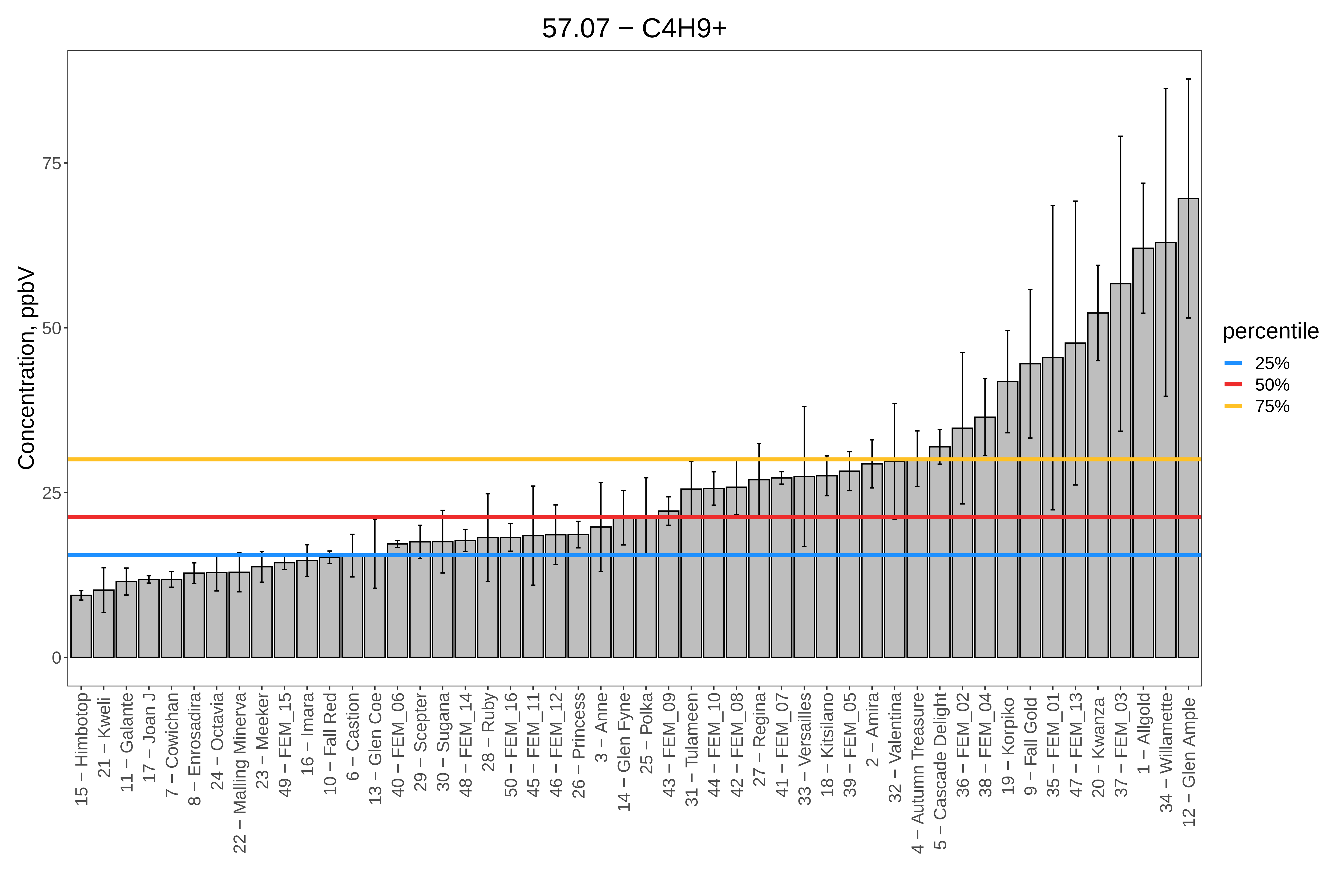
57.07 − C4H9+
Concentration, ppbV
0
25
50
75
percentile
25%
50%
75%
15 − Himbotop
21 − Kweli
11 − Galante
17 − Joan J
7 − Cowichan
8 − Enrosadira
24 − Octavia
22 − Malling Minerva
23 − Meeker
49 − FEM_15
16 − Imara
10 − Fall Red
6 − Castion
13 − Glen Coe
40 − FEM_06
29 − Scepter
30 − Sugana
48 − FEM_14
28 − Ruby
50 − FEM_16
45 − FEM_11
46 − FEM_12
26 − Princess
3 − Anne
14 − Glen Fyne
25 − Polka
43 − FEM_09
31 − Tulameen
44 − FEM_10
42 − FEM_08
27 − Regina
41 − FEM_07
33 − Versailles
18 − Kitsilano
39 − FEM_05
2 − Amira
32 − Valentina
4 − Autumn Treasure
5 − Cascade Delight
36 − FEM_02
38 − FEM_04
19 − Korpiko
9 − Fall Gold
35 − FEM_01
47 − FEM_13
20 − Kwanza
37 − FEM_03
1 − Allgold
34 − Willamette
12 − Glen Ample

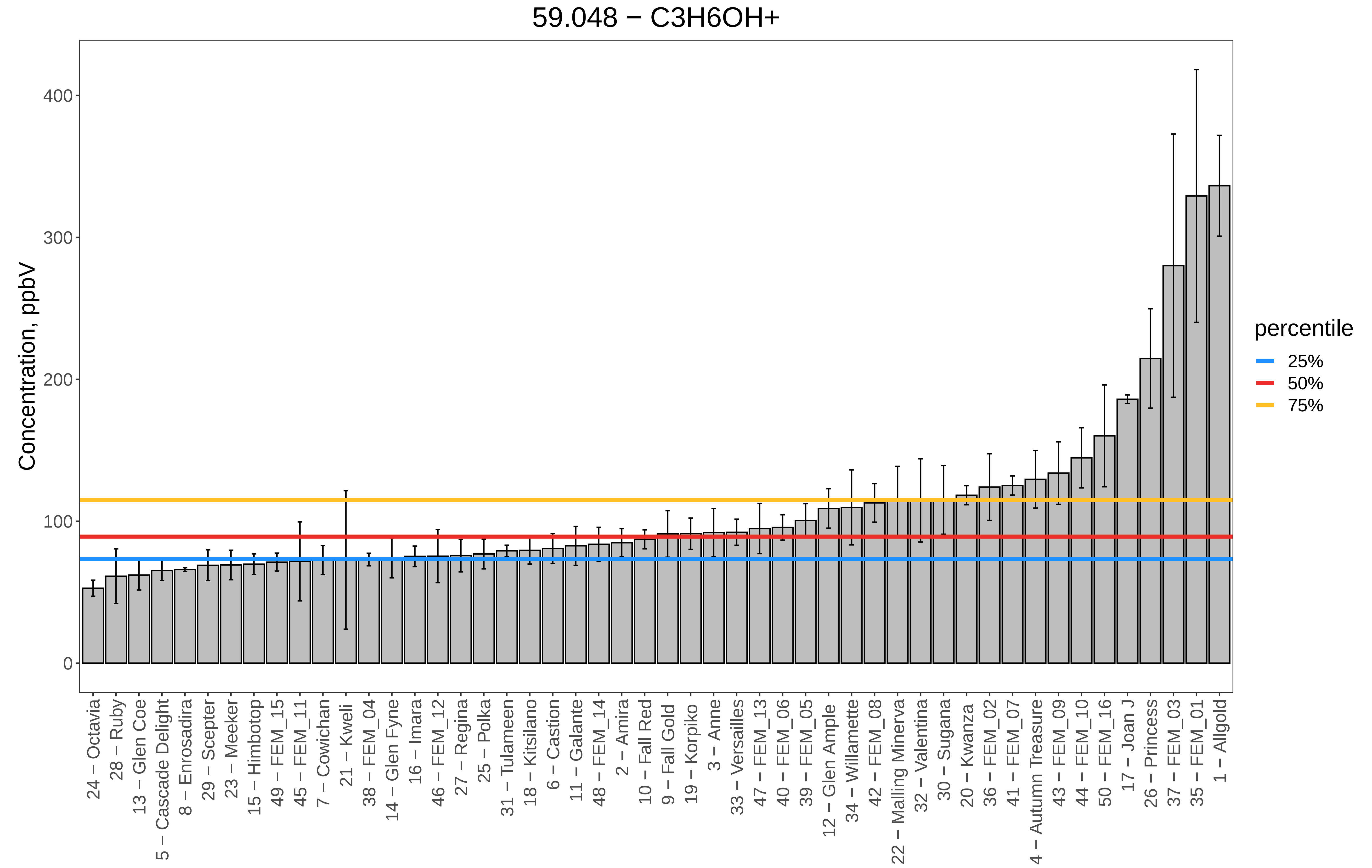

59.048 − C3H6OH+
Concentration, ppbV
0
100
200
300
400
percentile
25%
50%
75%
24 − Octavia
28 − Ruby
13 − Glen Coe
5 − Cascade Delight
8 − Enrosadira
29 − Scepter
23 − Meeker
15 − Himbotop
49 − FEM_15
45 − FEM_11
7 − Cowichan
21 − Kweli
38 − FEM_04
14 − Glen Fyne
16 − Imara
46 − FEM_12
27 − Regina
25 − Polka
31 − Tulameen
18 − Kitsilano
6 − Castion
11 − Galante
48 − FEM_14
2 − Amira
10 − Fall Red
9 − Fall Gold
19 − Korpiko
3 − Anne
33 − Versailles
47 − FEM_13
40 − FEM_06
39 − FEM_05
12 − Glen Ample
34 − Willamette
42 − FEM_08
22 − Malling Minerva
32 − Valentina
30 − Sugana
20 − Kwanza
36 − FEM_02
41 − FEM_07
4 − Autumn Treasure
43 − FEM_09
44 − FEM_10
50 − FEM_16
17 − Joan J
26 − Princess
37 − FEM_03
35 − FEM_01
1 − Allgold

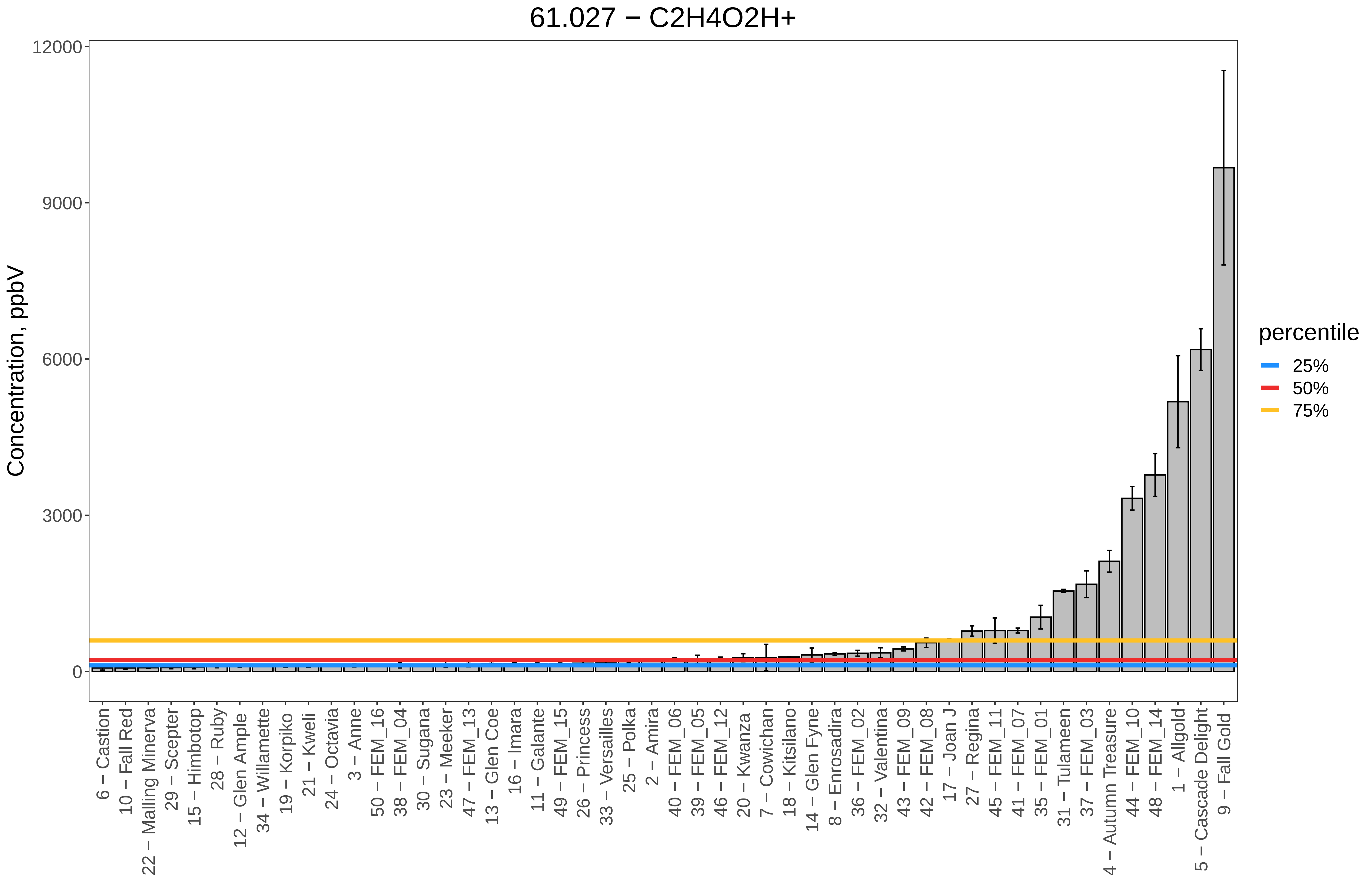

61.027 − C2H4O2H+
Concentration, ppbV
12000
9000
6000
3000
0
percentile
25%
50%
75%
6 − Castion
10 − Fall Red
22 − Malling Minerva
29 − Scepter
15 − Himbotop
28 − Ruby
12 − Glen Ample
34 − Willamette
19 − Korpiko
21 − Kweli
24 − Octavia
3 − Anne
50 − FEM_16
38 − FEM_04
30 − Sugana
23 − Meeker
47 − FEM_13
13 − Glen Coe
16 − Imara
11 − Galante
49 − FEM_15
26 − Princess
33 − Versailles
25 − Polka
2 − Amira
40 − FEM_06
39 − FEM_05
46 − FEM_12
20 − Kwanza
7 − Cowichan
18 − Kitsilano
14 − Glen Fyne
8 − Enrosadira
36 − FEM_02
32 − Valentina
43 − FEM_09
42 − FEM_08
17 − Joan J
27 − Regina
45 − FEM_11
41 − FEM_07
35 − FEM_01
31 − Tulameen
37 − FEM_03
4 − Autumn Treasure
44 − FEM_10
48 − FEM_14
1 − Allgold
5 − Cascade Delight
9 − Fall Gold

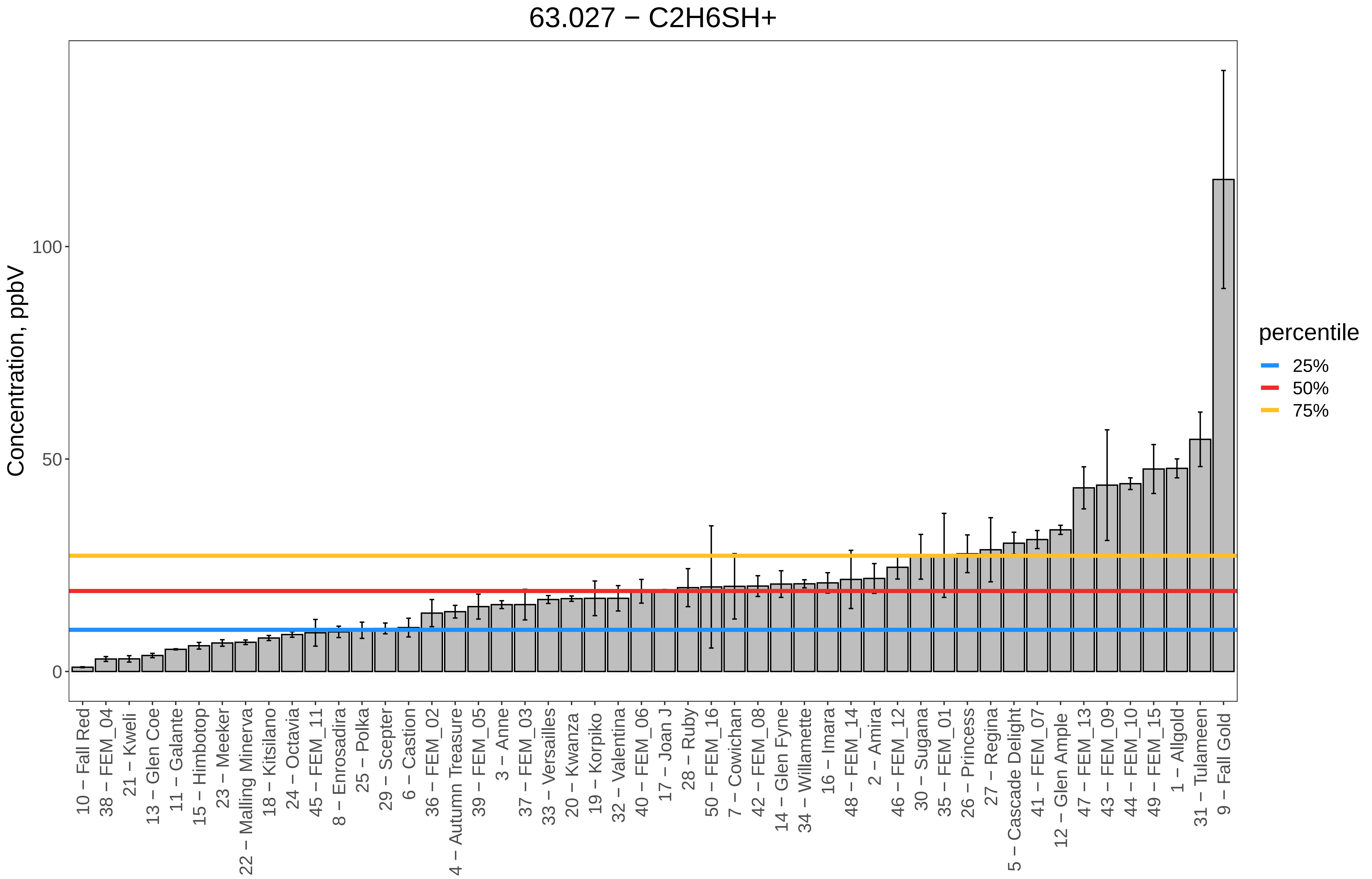

63.027 − C2H6SH+
Concentration, ppbV
0
50
100
percentile
25%
50%
75%
10 − Fall Red
38 − FEM_04
21 − Kweli
13 − Glen Coe
11 − Galante
15 − Himbotop
23 − Meeker
22 − Malling Minerva
18 − Kitsilano
24 − Octavia
45 − FEM_11
8 − Enrosadira
25 − Polka
29 − Scepter
6 − Castion
36 − FEM_02
4 − Autumn Treasure
39 − FEM_05
3 − Anne
37 − FEM_03
33 − Versailles
20 − Kwanza
19 − Korpiko
32 − Valentina
40 − FEM_06
17 − Joan J
28 − Ruby
50 − FEM_16
7 − Cowichan
42 − FEM_08
14 − Glen Fyne
34 − Willamette
16 − Imara
48 − FEM_14
2 − Amira
46 − FEM_12
30 − Sugana
35 − FEM_01
26 − Princess
27 − Regina
5 − Cascade Delight
41 − FEM_07
12 − Glen Ample
47 − FEM_13
43 − FEM_09
44 − FEM_10
49 − FEM_15
1 − Allgold
31 − Tulameen
9 − Fall Gold

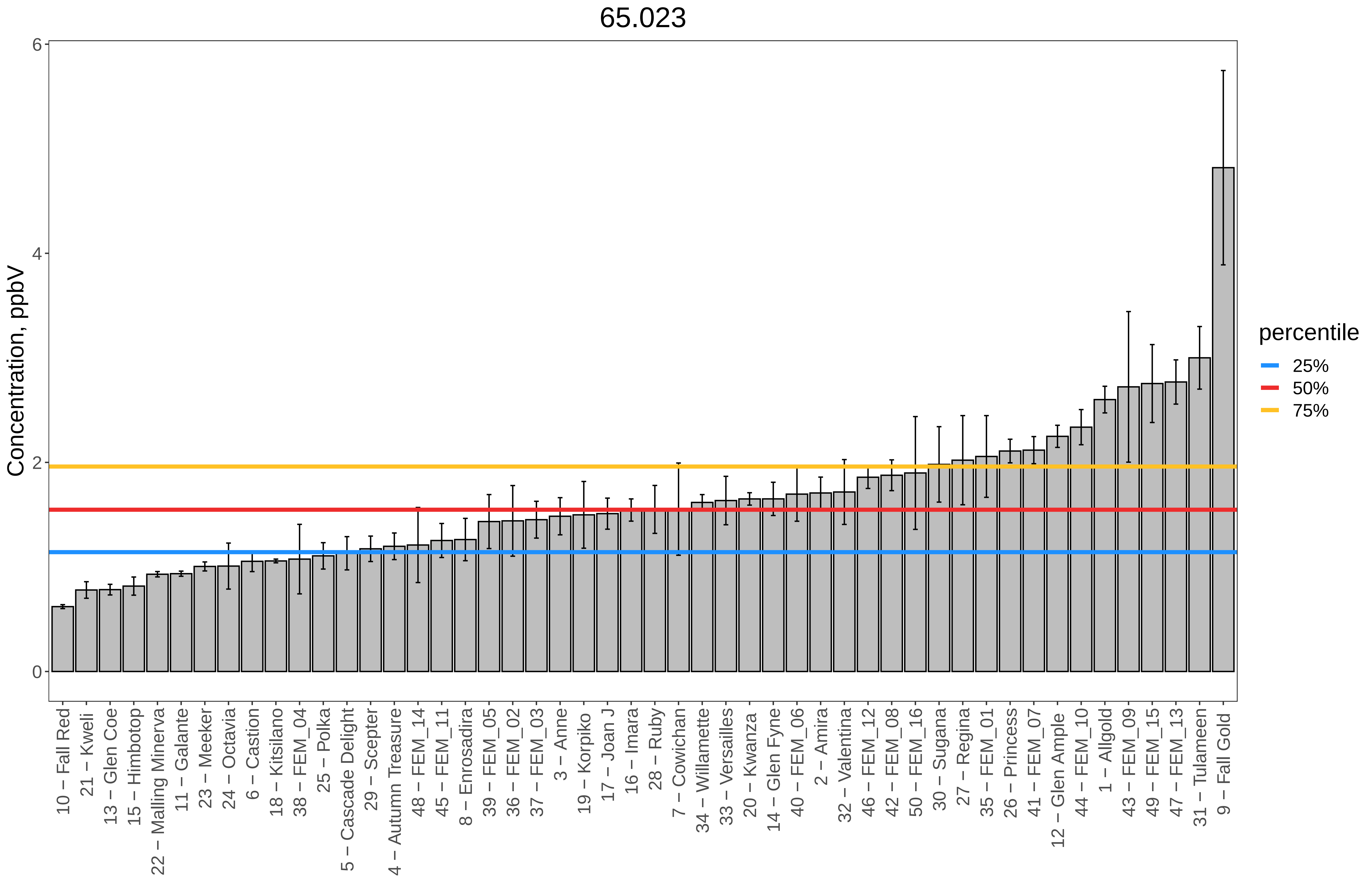

65.023
Concentration, ppbV
0
2
4
6
percentile
25%
50%
75%
10 – Fall Red
21 – Kweli
13 – Glen Coe
15 – Himbotop
22 – Malling Minerva
11 – Galante
23 – Meeker
24 – Octavia
6 – Castion
18 – Kitsilano
38 – FEM_04
25 – Polka
5 – Cascade Delight
29 – Scepter
4 – Autumn Treasure
48 – FEM_14
45 – FEM_11
8 – Enrosadira
39 – FEM_05
36 – FEM_02
37 – FEM_03
3 – Anne
19 – Korpiko
17 – Joan J
16 – Imara
28 – Ruby
7 – Cowichan
34 – Willamette
33 – Versailles
20 – Kwanza
14 – Glen Fyne
40 – FEM_06
2 – Amira
32 – Valentina
46 – FEM_12
42 – FEM_08
50 – FEM_16
30 – Sugana
27 – Regina
35 – FEM_01
26 – Princess
41 – FEM_07
12 – Glen Ample
44 – FEM_10
1 – Allgold
43 – FEM_09
49 – FEM_15
47 – FEM_13
31 – Tulameen
9 – Fall Gold

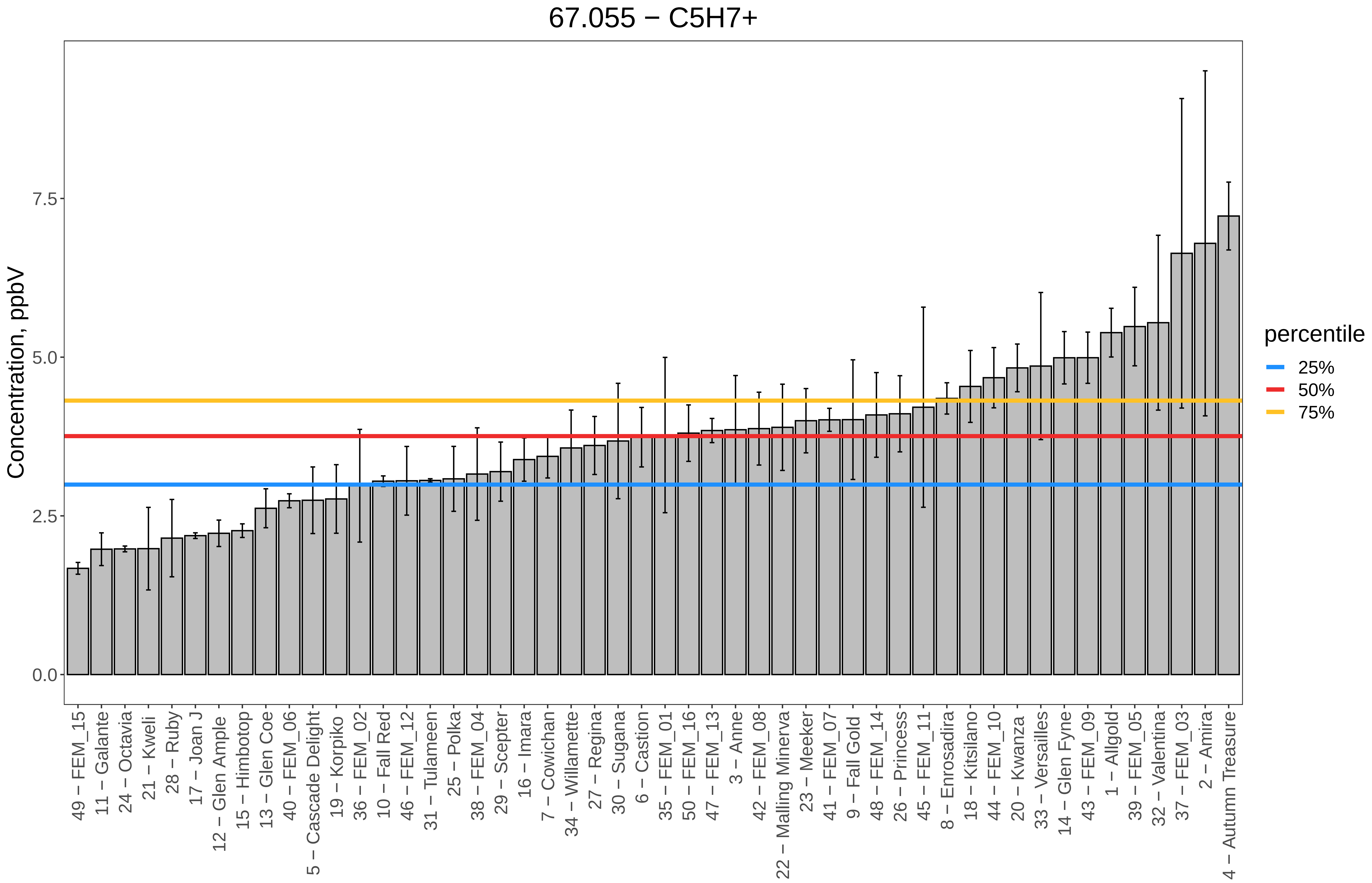

67.055 − C5H7+
Concentration, ppbV
0.0
2.5
5.0
7.5
percentile
25%
50%
75%
49 − FEM_15
11 − Galante
24 − Octavia
21 − Kweli
28 − Ruby
17 − Joan J
12 − Glen Ample
15 − Himbotop
13 − Glen Coe
40 − FEM_06
5 − Cascade Delight
19 − Korpiko
36 − FEM_02
10 − Fall Red
46 − FEM_12
31 − Tulameen
25 − Polka
38 − FEM_04
29 − Scepter
16 − Imara
7 − Cowichan
34 − Willamette
27 − Regina
30 − Sugana
6 − Castion
35 − FEM_01
50 − FEM_16
47 − FEM_13
3 − Anne
42 − FEM_08
22 − Malling Minerva
23 − Meeker
41 − FEM_07
9 − Fall Gold
48 − FEM_14
26 − Princess
45 − FEM_11
8 − Enrosadira
18 − Kitsilano
44 − FEM_10
20 − Kwanza
33 − Versailles
14 − Glen Fyne
43 − FEM_09
1 − Allgold
39 − FEM_05
32 − Valentina
37 − FEM_03
2 − Amira
4 − Autumn Treasure

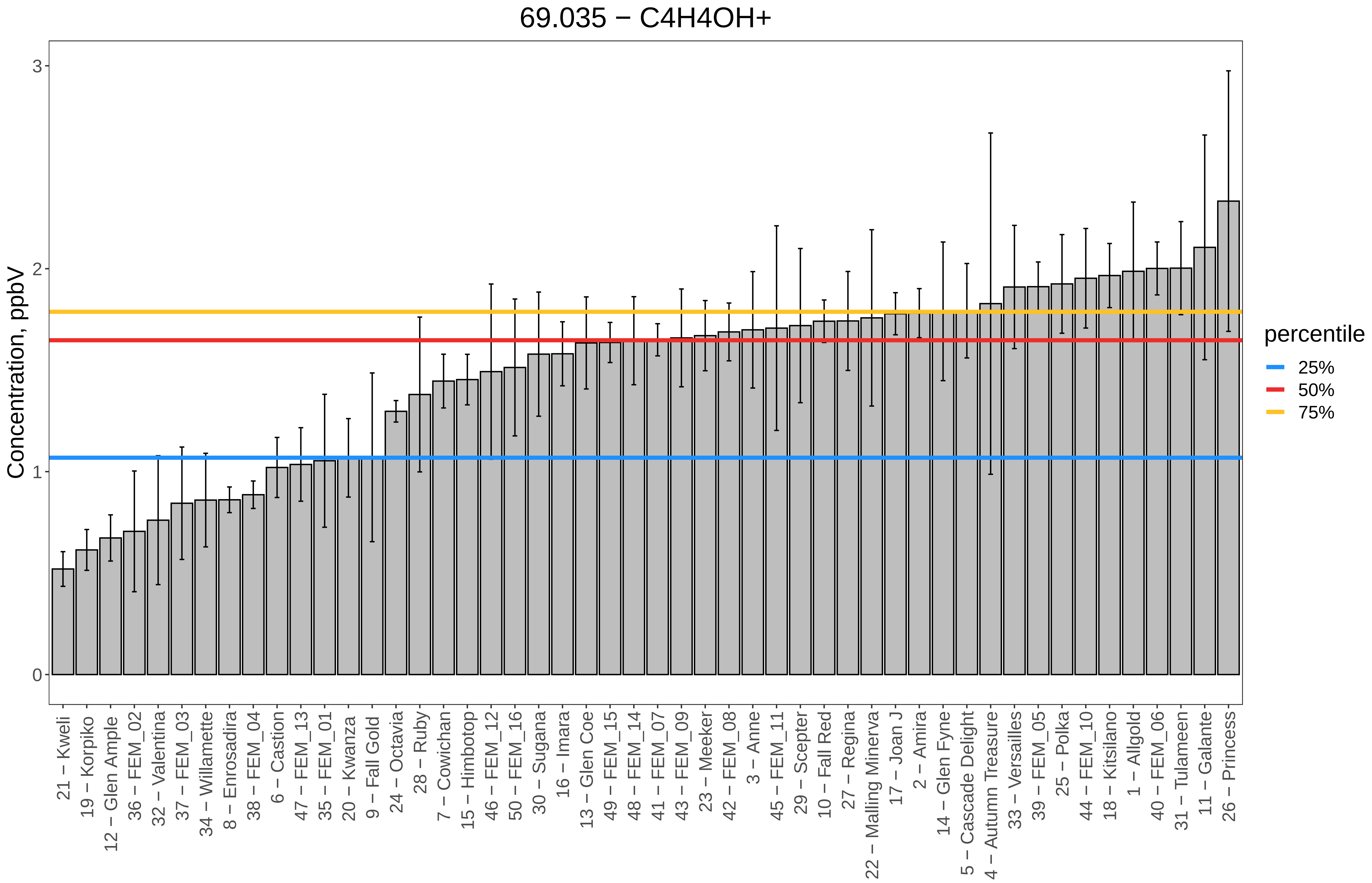
69.035 − C4H4OH+
Concentration, ppbV
percentile
25%
50%
75%
21 − Kweli
19 − Korpiko
12 − Glen Ample
36 − FEM_02
32 − Valentina
37 − FEM_03
34 − Willamette
8 − Enrosadira
38 − FEM_04
6 − Castion
47 − FEM_13
35 − FEM_01
20 − Kwanza
9 − Fall Gold
24 − Octavia
28 − Ruby
7 − Cowichan
15 − Himbotop
46 − FEM_12
50 − FEM_16
30 − Sugana
16 − Imara
13 − Glen Coe
49 − FEM_15
48 − FEM_14
41 − FEM_07
43 − FEM_09
23 − Meeker
42 − FEM_08
3 − Anne
45 − FEM_11
29 − Scepter
10 − Fall Red
27 − Regina
22 − Malling Minerva
17 − Joan J
2 − Amira
14 − Glen Fyne
5 − Cascade Delight
4 − Autumn Treasure
33 − Versailles
39 − FEM_05
25 − Polka
44 − FEM_10
18 − Kitsilano
1 − Allgold
40 − FEM_06
31 − Tulameen
11 − Galante
26 − Princess

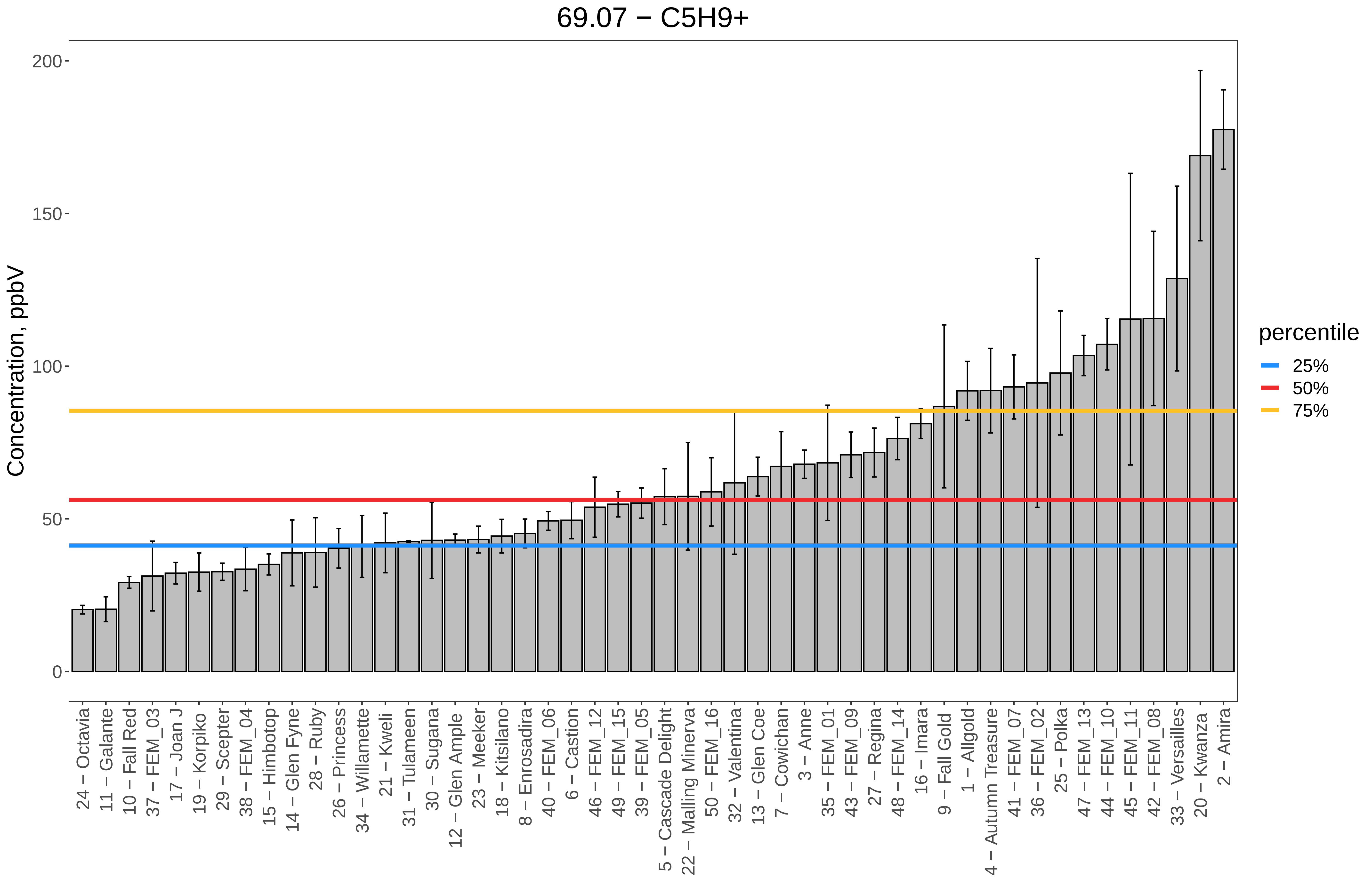

69.07 − C5H9+
Concentration, ppbV
0
50
100
150
200
percentile
25%
50%
75%
24 − Octavia
11 − Galante
10 − Fall Red
37 − FEM_03
17 − Joan J
19 − Korpiko
29 − Scepter
38 − FEM_04
15 − Himbotop
14 − Glen Fyne
28 − Ruby
26 − Princess
34 − Willamette
21 − Kweli
31 − Tulameen
30 − Sugana
12 − Glen Ample
23 − Meeker
18 − Kitsilano
8 − Enrosadira
40 − FEM_06
6 − Castion
46 − FEM_12
49 − FEM_15
39 − FEM_05
5 − Cascade Delight
22 − Malling Minerva
50 − FEM_16
32 − Valentina
13 − Glen Coe
7 − Cowichan
3 − Anne
35 − FEM_01
43 − FEM_09
27 − Regina
48 − FEM_14
16 − Imara
9 − Fall Gold
1 − Allgold
4 − Autumn Treasure
41 − FEM_07
36 − FEM_02
25 − Polka
47 − FEM_13
44 − FEM_10
45 − FEM_11
42 − FEM_08
33 − Versailles
20 − Kwanza
2 − Amira

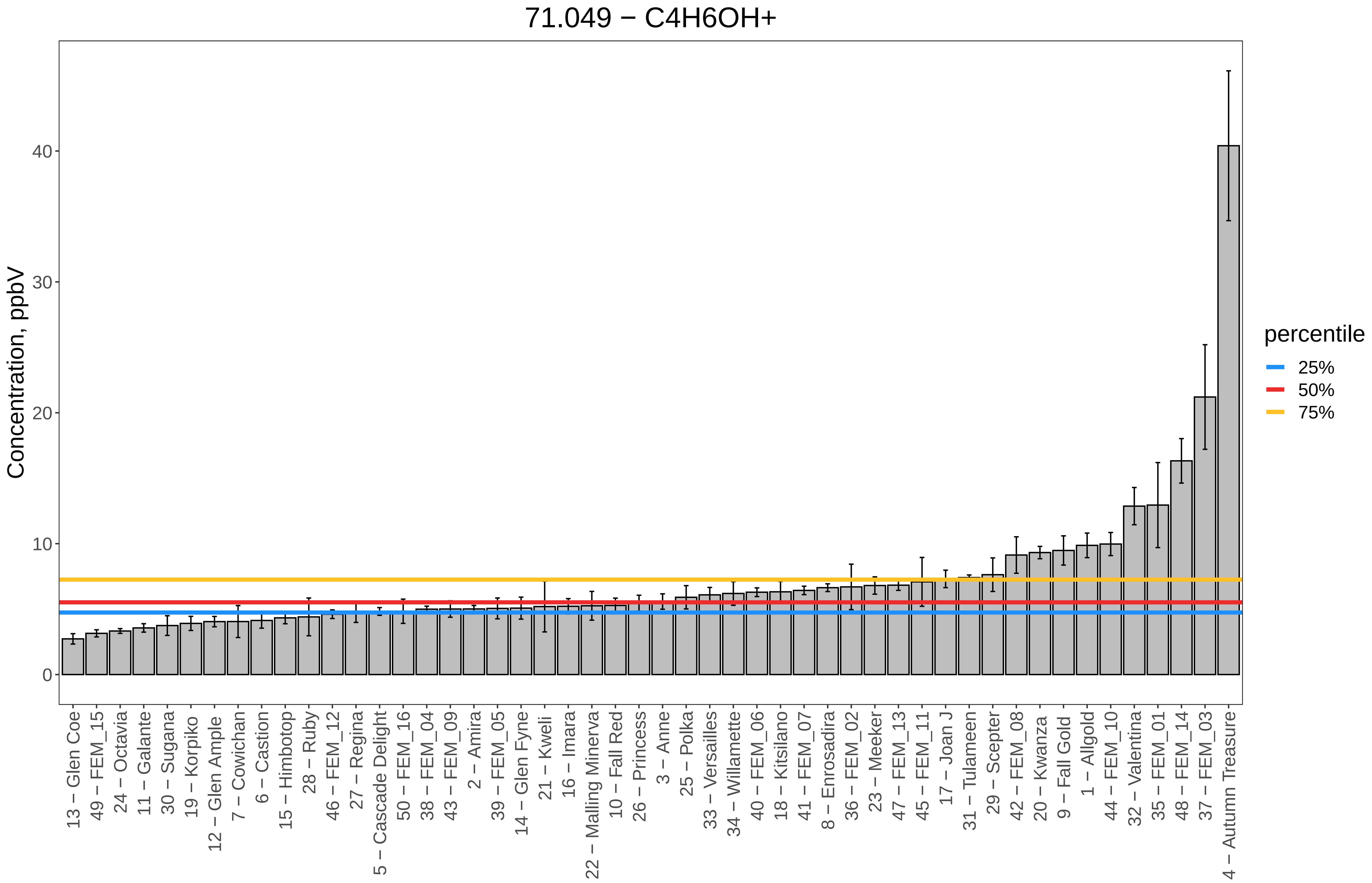

71.049 − C4H6OH+
Concentration, ppbV
0
10
20
30
40
percentile
25%
50%
75%
13 − Glen Coe
49 − FEM_15
24 − Octavia
11 − Galante
30 − Sugana
19 − Korpiko
12 − Glen Ample
7 − Cowichan
6 − Castion
15 − Himbotop
28 − Ruby
46 − FEM_12
27 − Regina
5 − Cascade Delight
50 − FEM_16
38 − FEM_04
43 − FEM_09
2 − Amira
39 − FEM_05
14 − Glen Fyne
21 − Kweli
16 − Imara
22 − Malling Minerva
10 − Fall Red
26 − Princess
3 − Anne
25 − Polka
33 − Versailles
34 − Willamette
40 − FEM_06
18 − Kitsilano
41 − FEM_07
8 − Enrosadira
36 − FEM_02
23 − Meeker
47 − FEM_13
45 − FEM_11
17 − Joan J
31 − Tulameen
29 − Scepter
42 − FEM_08
20 − Kwanza
9 − Fall Gold
1 − Allgold
44 − FEM_10
32 − Valentina
35 − FEM_01
48 − FEM_14
37 − FEM_03
4 − Autumn Treasure

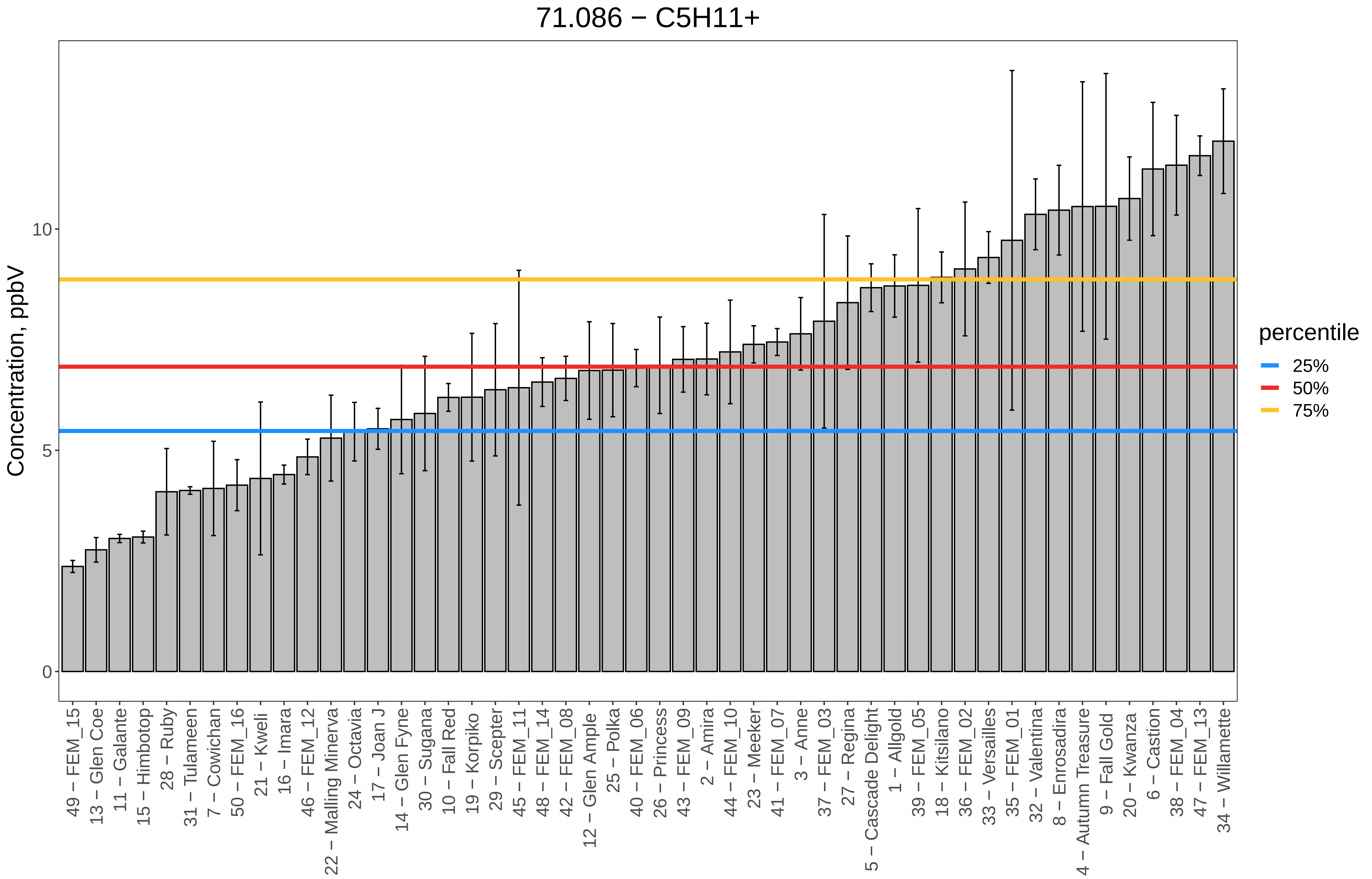

71.086 − C5H11+
Concentration, ppbV
0
5
10
percentile
25%
50%
75%
49 – FEM_15
13 – Glen Coe
11 – Galante
15 – Himbotop
28 – Ruby
31 – Tulameen
7 – Cowichan
50 – FEM_16
21 – Kweli
16 – Imara
46 – FEM_12
22 – Malling Minerva
24 – Octavia
17 – Joan J
14 – Glen Fyne
30 – Sugana
10 – Fall Red
19 – Korpiko
29 – Scepter
45 – FEM_11
48 – FEM_14
42 – FEM_08
12 – Glen Ample
25 – Polka
40 – FEM_06
26 – Princess
43 – FEM_09
2 – Amira
44 – FEM_10
23 – Meeker
41 – FEM_07
3 – Anne
37 – FEM_03
27 – Regina
5 – Cascade Delight
1 – Allgold
39 – FEM_05
18 – Kitsilano
36 – FEM_02
33 – Versailles
35 – FEM_01
32 – Valentina
8 – Enrosadira
4 – Autumn Treasure
9 – Fall Gold
20 – Kwanza
6 – Castion
38 – FEM_04
47 – FEM_13
34 – Willamette

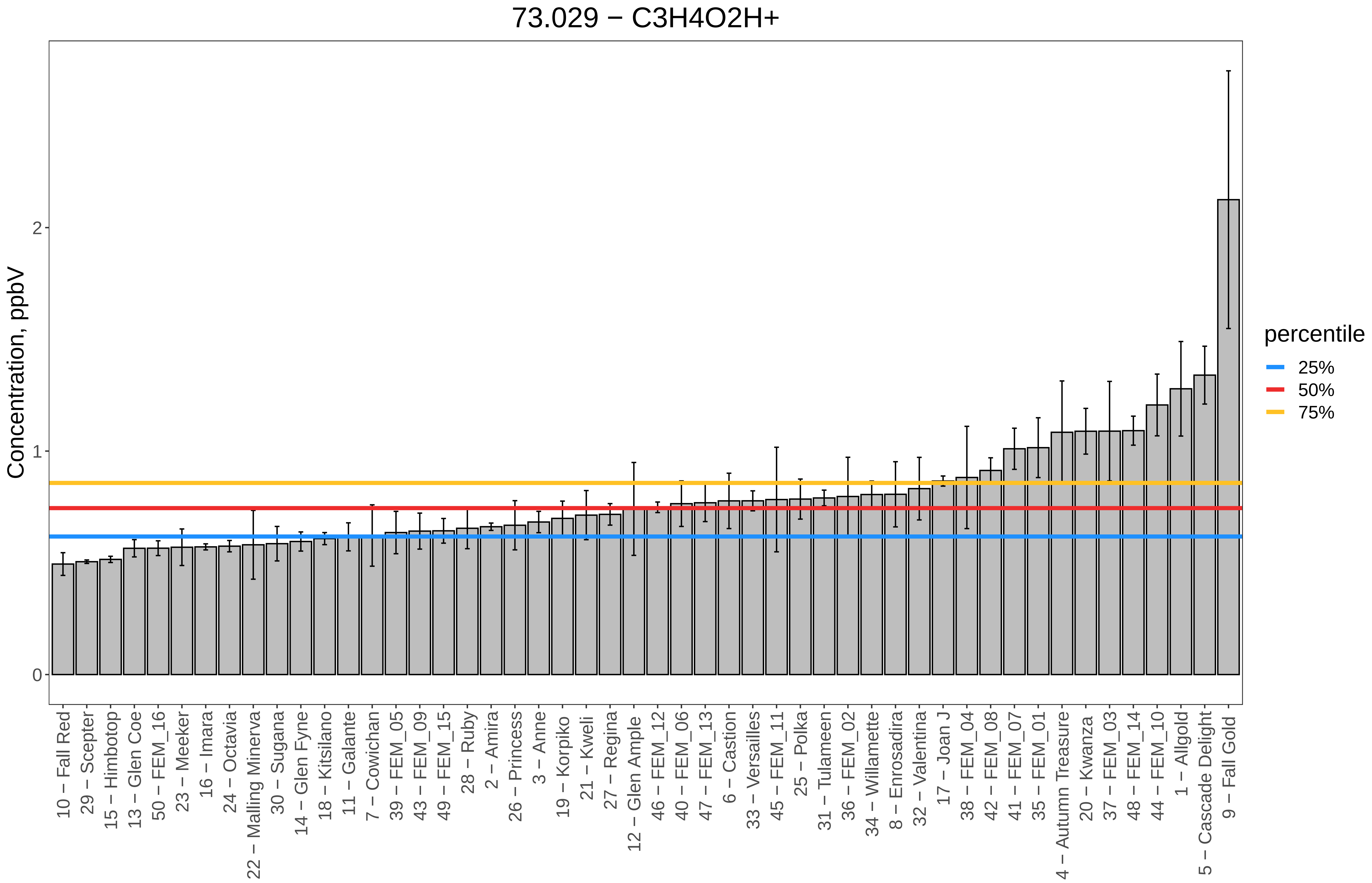

73.029 − C3H4O2H+
Concentration, ppbV
0
1
2
percentile
25%
50%
75%
10 − Fall Red
29 − Scepter
15 − Himbotop
13 − Glen Coe
50 − FEM_16
23 − Meeker
16 − Imara
24 − Octavia
22 − Malling Minerva
30 − Sugana
14 − Glen Fyne
18 − Kitsilano
11 − Galante
7 − Cowichan
39 − FEM_05
43 − FEM_09
49 − FEM_15
28 − Ruby
2 − Amira
26 − Princess
3 − Anne
19 − Korpiko
21 − Kweli
27 − Regina
12 − Glen Ample
46 − FEM_12
40 − FEM_06
47 − FEM_13
6 − Castion
33 − Versailles
45 − FEM_11
25 − Polka
31 − Tulameen
36 − FEM_02
34 − Willamette
8 − Enrosadira
32 − Valentina
17 − Joan J
38 − FEM_04
42 − FEM_08
41 − FEM_07
35 − FEM_01
4 − Autumn Treasure
20 − Kwanza
37 − FEM_03
48 − FEM_14
44 − FEM_10
1 − Allgold
5 − Cascade Delight
9 − Fall Gold

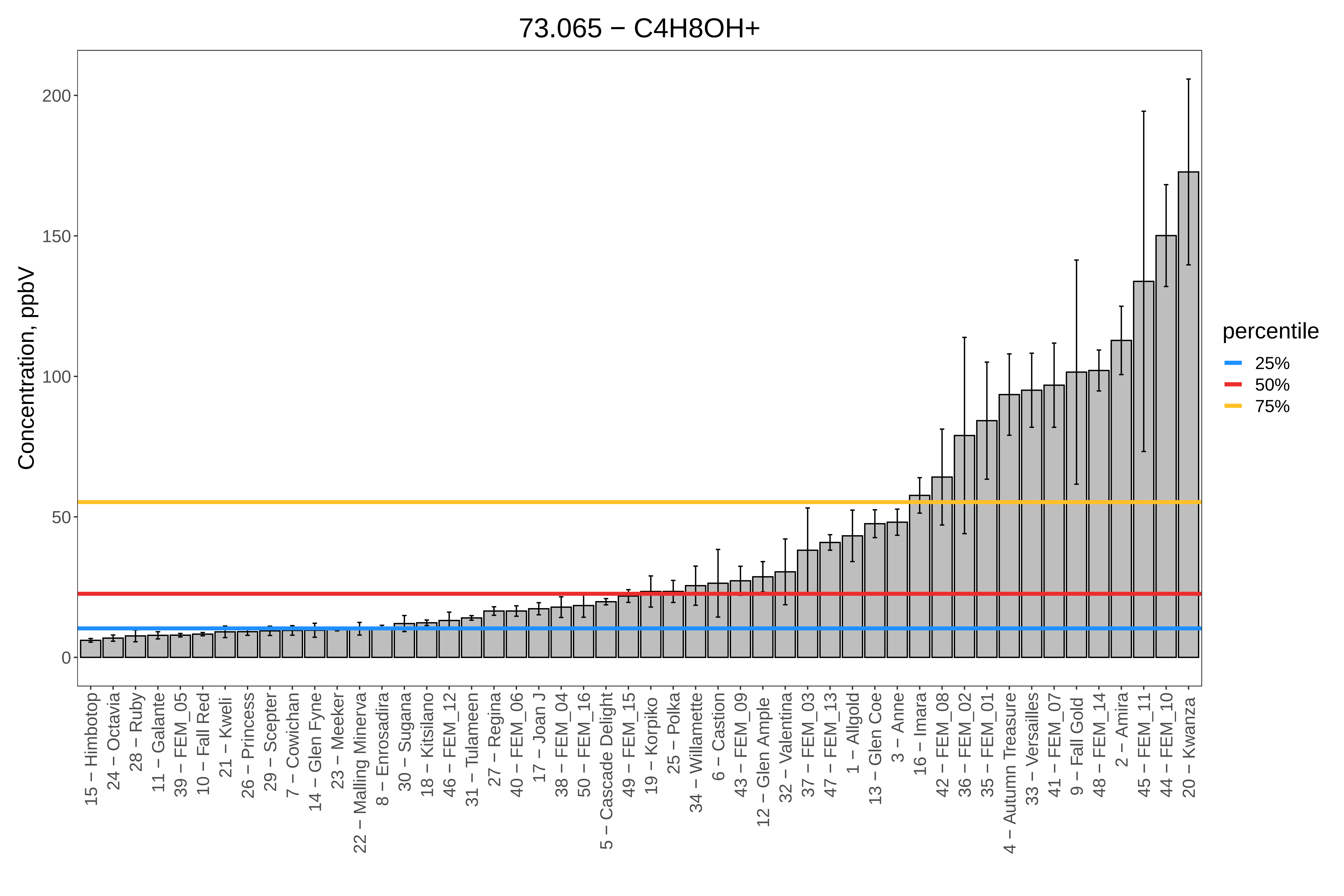
73.065 − C4H8OH+
Concentration, ppbV
0
50
100
150
200
percentile
25%
50%
75%
15 − Himbotop
24 − Octavia
28 − Ruby
11 − Galante
39 − FEM_05
10 − Fall Red
21 − Kweli
26 − Princess
29 − Scepter
7 − Cowichan
14 − Glen Fyne
23 − Meeker
22 − Malling Minerva
8 − Enrosadira
30 − Sugana
18 − Kitsilano
46 − FEM_12
31 − Tulameen
27 − Regina
40 − FEM_06
17 − Joan J
38 − FEM_04
50 − FEM_16
5 − Cascade Delight
49 − FEM_15
19 − Korpiko
25 − Polka
34 − Willamette
6 − Castion
43 − FEM_09
12 − Glen Ample
32 − Valentina
37 − FEM_03
47 − FEM_13
1 − Allgold
13 − Glen Coe
3 − Anne
16 − Imara
42 − FEM_08
36 − FEM_02
35 − FEM_01
4 − Autumn Treasure
33 − Versailles
41 − FEM_07
9 − Fall Gold
48 − FEM_14
2 − Amira
45 − FEM_11
44 − FEM_10
20 − Kwanza

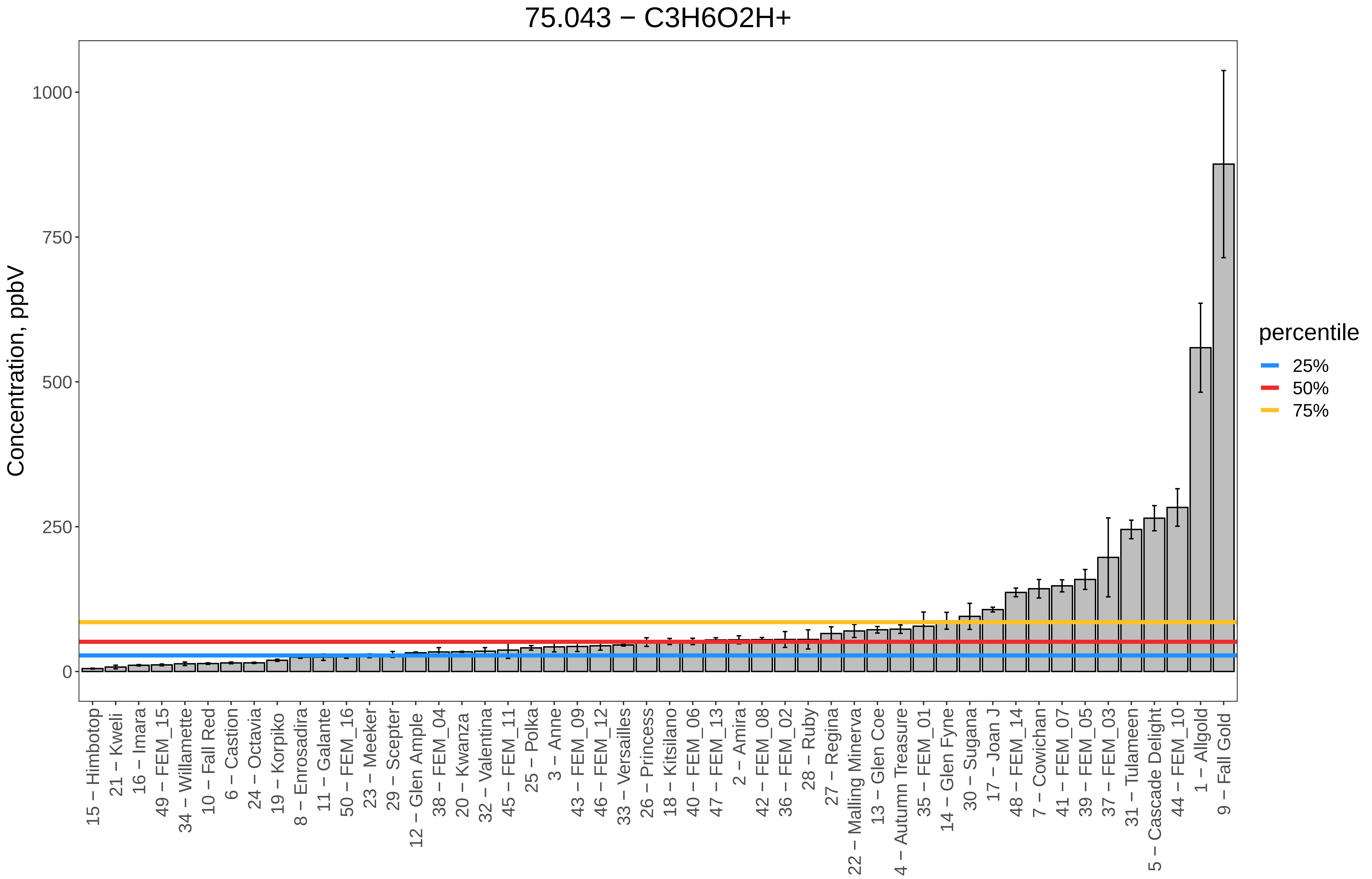

75.043 − C3H6O2H+
Concentration, ppbV
1000
750
500
250
0
percentile
25%
50%
75%
15 − Himbotop
21 − Kweli
16 − Imara
49 − FEM_15
34 − Willamette
10 − Fall Red
6 − Castion
24 − Octavia
19 − Korpiko
8 − Enrosadira
11 − Galante
50 − FEM_16
23 − Meeker
29 − Scepter
12 − Glen Ample
38 − FEM_04
20 − Kwanza
32 − Valentina
45 − FEM_11
25 − Polka
3 − Anne
43 − FEM_09
46 − FEM_12
33 − Versailles
26 − Princess
18 − Kitsilano
40 − FEM_06
47 − FEM_13
2 − Amira
42 − FEM_08
36 − FEM_02
28 − Ruby
27 − Regina
22 − Malling Minerva
13 − Glen Coe
4 − Autumn Treasure
35 − FEM_01
14 − Glen Fyne
30 − Sugana
17 − Joan J
48 − FEM_14
7 − Cowichan
41 − FEM_07
39 − FEM_05
37 − FEM_03
31 − Tulameen
5 − Cascade Delight
44 − FEM_10
1 − Allgold
9 − Fall Gold

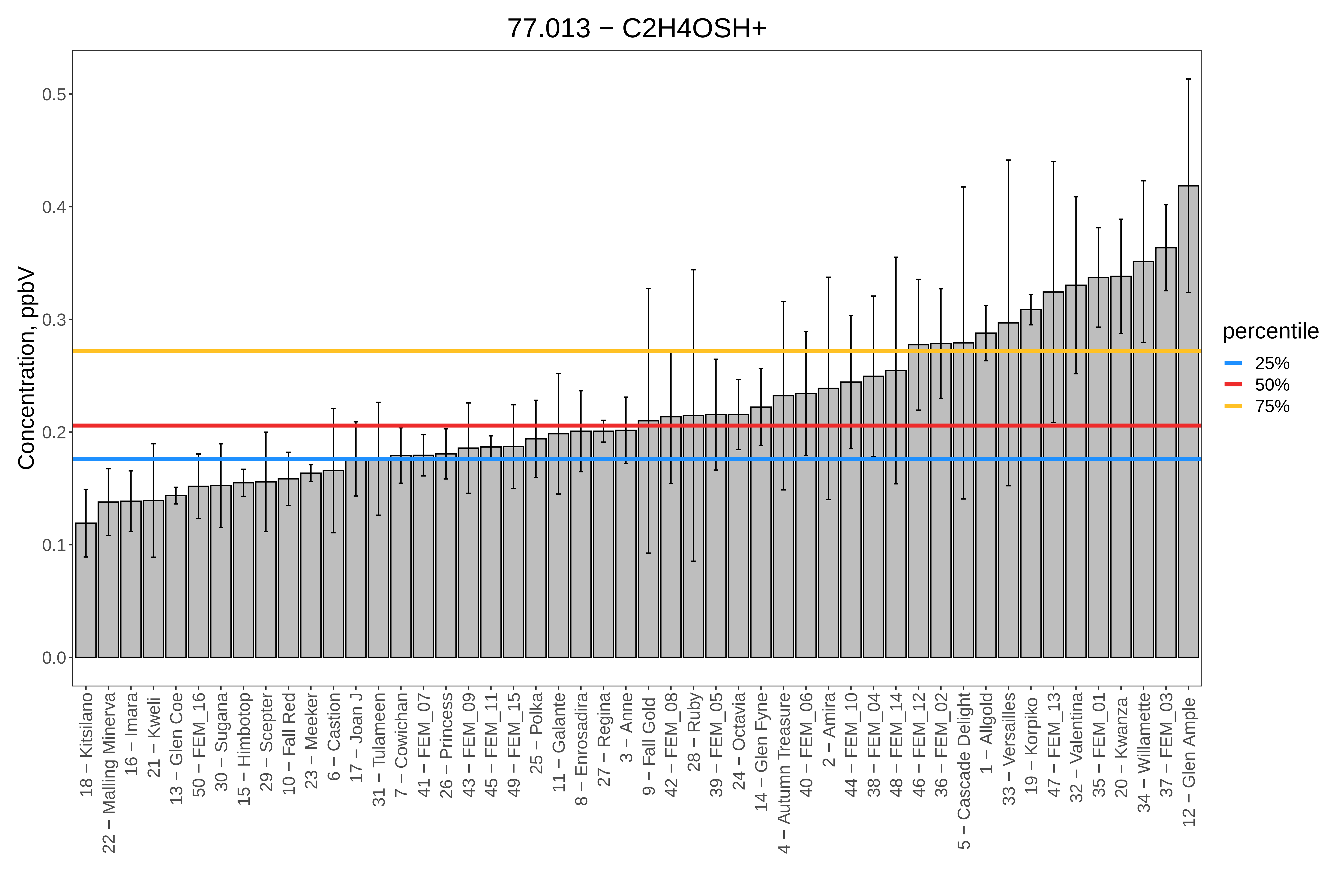
77.013 − C2H4OSH+
Concentration, ppbV
0.0
0.1
0.2
0.3
0.4
0.5
percentile
25%
50%
75%
18 − Kitsilano
22 − Malling Minerva
16 − Imara
21 − Kweli
13 − Glen Coe
50 − FEM_16
30 − Sugana
15 − Himbotop
29 − Scepter
10 − Fall Red
23 − Meeker
6 − Castion
17 − Joan J
31 − Tulameen
7 − Cowichan
41 − FEM_07
26 − Princess
43 − FEM_09
45 − FEM_11
49 − FEM_15
25 − Polka
11 − Galante
8 − Enrosadira
27 − Regina
3 − Anne
9 − Fall Gold
42 − FEM_08
28 − Ruby
39 − FEM_05
24 − Octavia
14 − Glen Fyne
4 − Autumn Treasure
40 − FEM_06
2 − Amira
44 − FEM_10
38 − FEM_04
48 − FEM_14
46 − FEM_12
36 − FEM_02
5 − Cascade Delight
1 − Allgold
33 − Versailles
19 − Korpiko
47 − FEM_13
32 − Valentina
35 − FEM_01
20 − Kwanza
34 − Willamette
37 − FEM_03
12 − Glen Ample

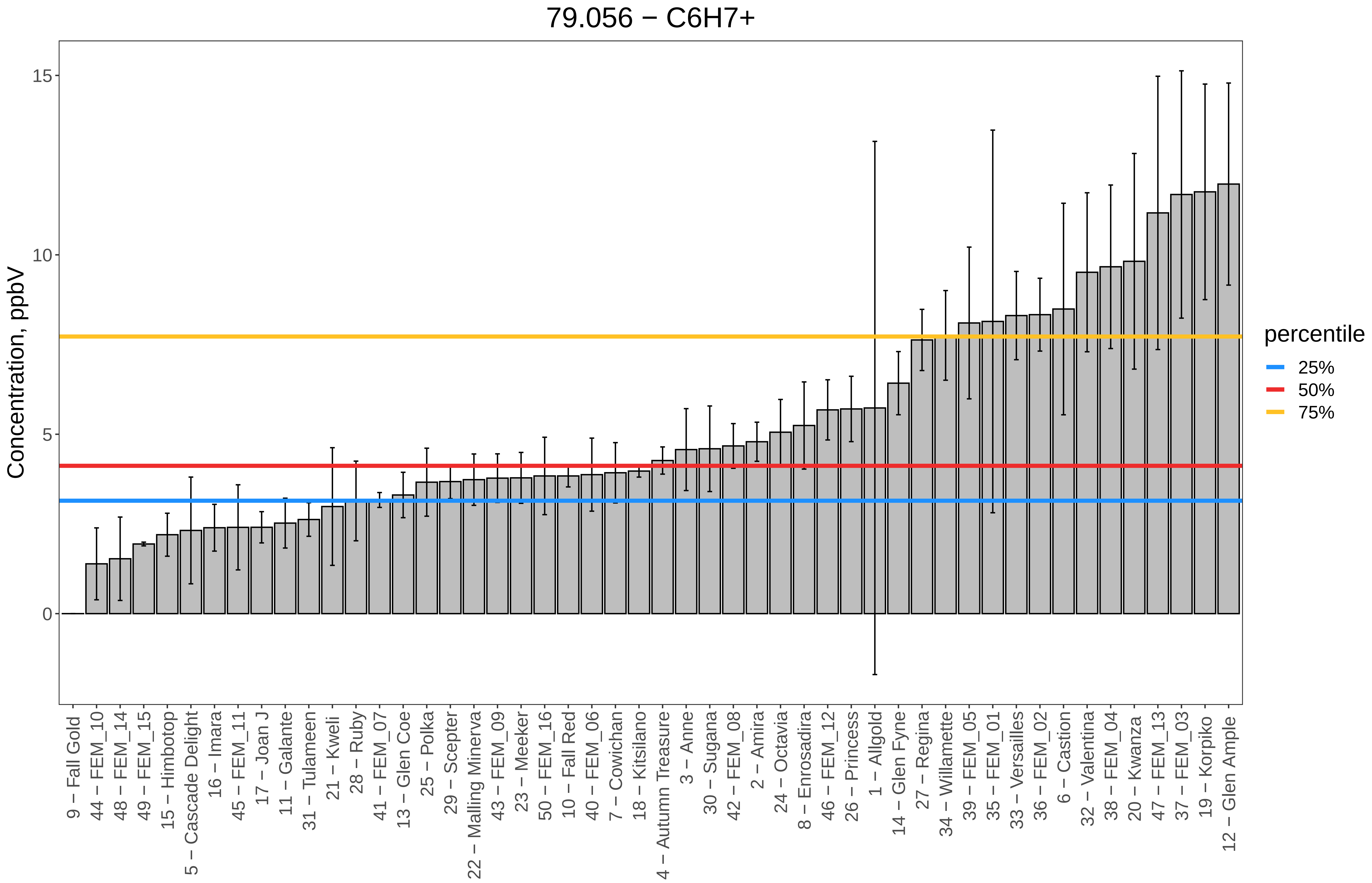
79.056 − C6H7+
Concentration, ppbV
0
5
10
15
percentile
25%
50%
75%
9 − Fall Gold
44 − FEM_10
48 − FEM_14
49 − FEM_15
15 − Himbotop
5 − Cascade Delight
16 − Imara
45 − FEM_11
17 − Joan J
11 − Galante
31 − Tulameen
21 − Kweli
28 − Ruby
41 − FEM_07
13 − Glen Coe
25 − Polka
29 − Scepter
22 − Malling Minerva
43 − FEM_09
23 − Meeker
50 − FEM_16
10 − Fall Red
40 − FEM_06
7 − Cowichan
18 − Kitsilano
4 − Autumn Treasure
3 − Anne
30 − Sugana
42 − FEM_08
2 − Amira
24 − Octavia
8 − Enrosadira
46 − FEM_12
26 − Princess
1 − Allgold
14 − Glen Fyne
27 − Regina
34 − Willamette
39 − FEM_05
35 − FEM_01
33 − Versailles
36 − FEM_02
6 − Castion
32 − Valentina
38 − FEM_04
20 − Kwanza
47 − FEM_13
37 − FEM_03
19 − Korpiko
12 − Glen Ample

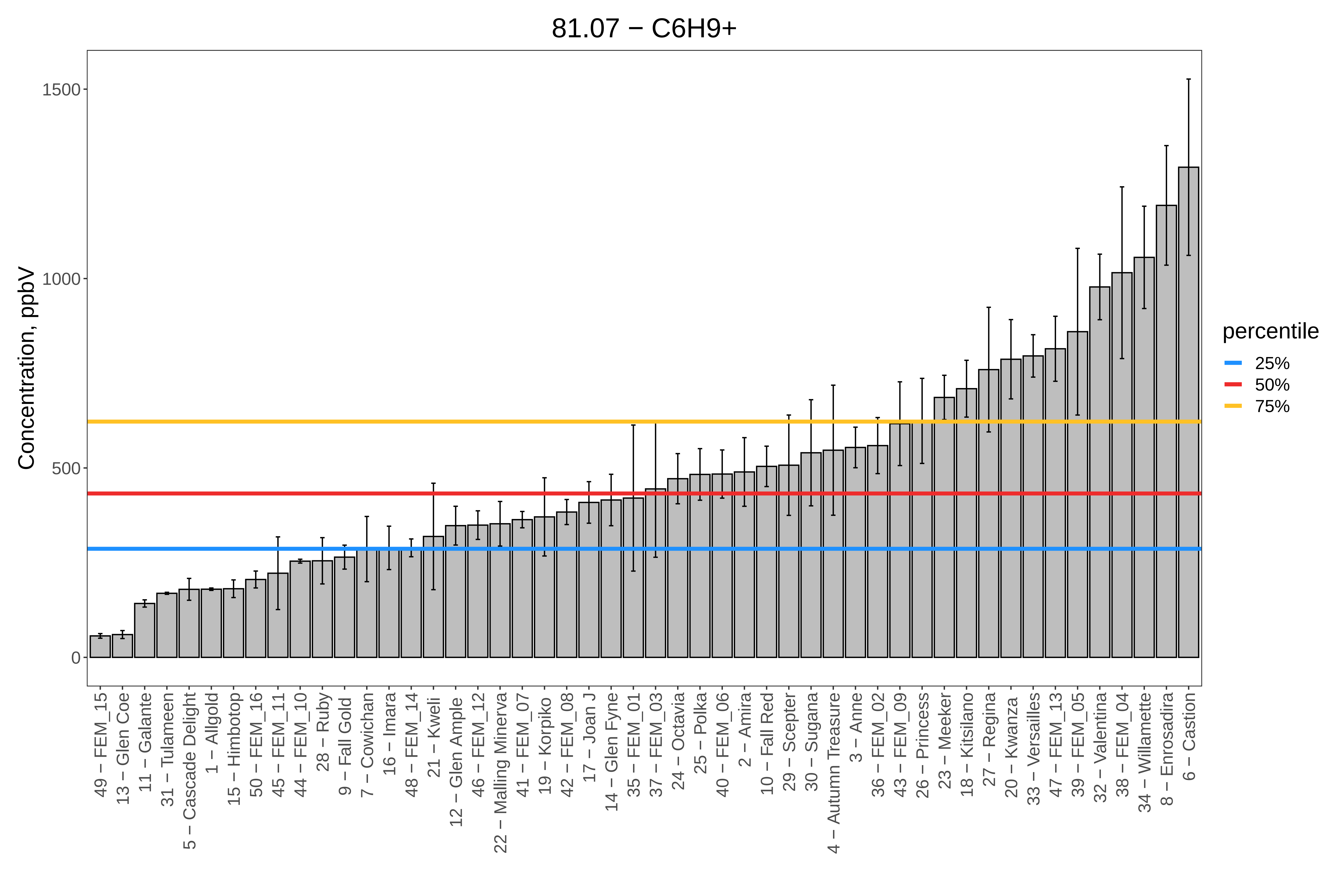

81.07 − C6H9+
Concentration, ppbV
1500
1000
500
0
percentile
25%
50%
75%
49 − FEM_15
13 − Glen Coe
11 − Galante
31 − Tulameen
5 − Cascade Delight
1 − Allgold
15 − Himbotop
50 − FEM_16
45 − FEM_11
44 − FEM_10
28 − Ruby
9 − Fall Gold
7 − Cowichan
16 − Imara
48 − FEM_14
21 − Kweli
12 − Glen Ample
46 − FEM_12
22 − Malling Minerva
41 − FEM_07
19 − Korpiko
42 − FEM_08
17 − Joan J
14 − Glen Fyne
35 − FEM_01
37 − FEM_03
24 − Octavia
25 − Polka
40 − FEM_06
2 − Amira
10 − Fall Red
29 − Scepter
30 − Sugana
4 − Autumn Treasure
3 − Anne
36 − FEM_02
43 − FEM_09
26 − Princess
23 − Meeker
18 − Kitsilano
27 − Regina
20 − Kwanza
33 − Versailles
47 − FEM_13
39 − FEM_05
32 − Valentina
38 − FEM_04
34 − Willamette
8 − Enrosadira
6 − Castion

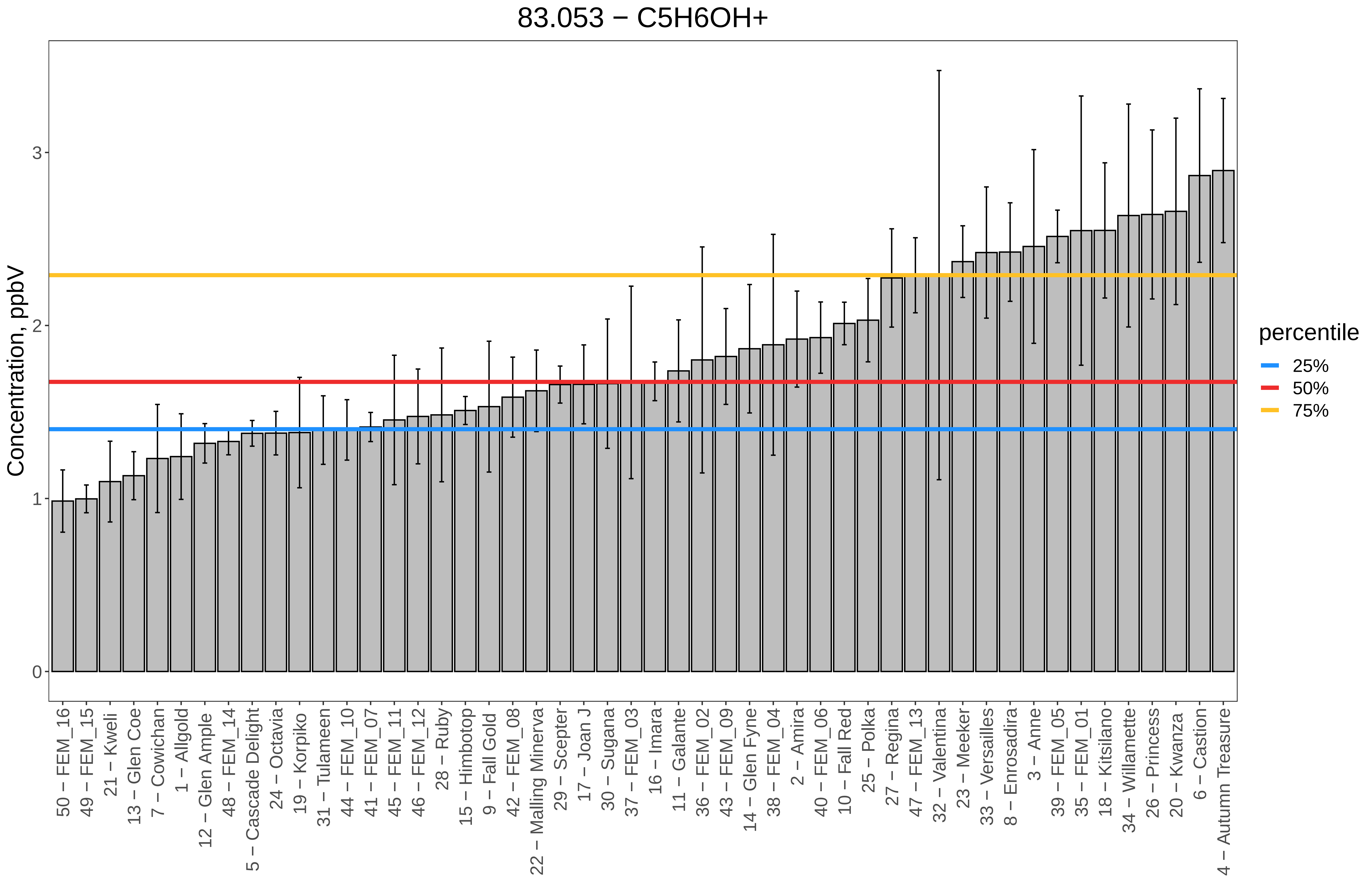
83.053 − C5H6OH+
Concentration, ppbV
0
1
2
3
percentile
25%
50%
75%
50 − FEM_16
49 − FEM_15
21 − Kweli
13 − Glen Coe
7 − Cowichan
1 − Allgold
12 − Glen Ample
48 − FEM_14
5 − Cascade Delight
24 − Octavia
19 − Korpiko
31 − Tulameen
44 − FEM_10
41 − FEM_07
45 − FEM_11
46 − FEM_12
28 − Ruby
15 − Himbotop
9 − Fall Gold
42 − FEM_08
22 − Malling Minerva
29 − Scepter
17 − Joan J
30 − Sugana
37 − FEM_03
16 − Imara
11 − Galante
36 − FEM_02
43 − FEM_09
14 − Glen Fyne
38 − FEM_04
2 − Amira
40 − FEM_06
10 − Fall Red
25 − Polka
27 − Regina
47 − FEM_13
32 − Valentina
23 − Meeker
33 − Versailles
8 − Enrosadira
3 − Anne
39 − FEM_05
35 − FEM_01
18 − Kitsilano
34 − Willamette
26 − Princess
20 − Kwanza
6 − Castion
4 − Autumn Treasure

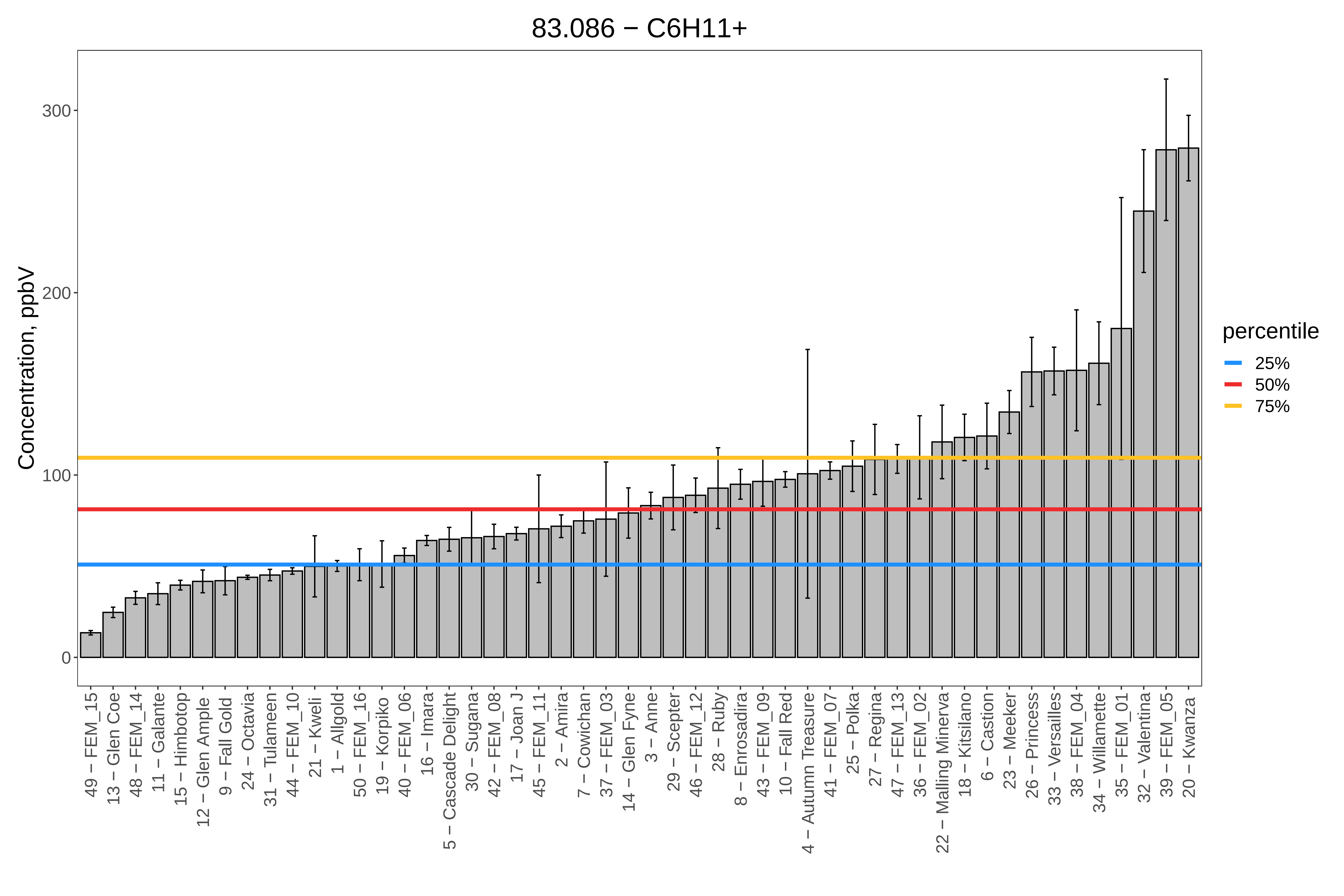

83.086 − C6H11+
Concentration, ppbV
0
100
200
300
percentile
25%
50%
75%
49 − FEM_15
13 − Glen Coe
48 − FEM_14
11 − Galante
15 − Himbotop
12 − Glen Ample
9 − Fall Gold
24 − Octavia
31 − Tulameen
44 − FEM_10
21 − Kweli
1 − Allgold
50 − FEM_16
19 − Korpiko
40 − FEM_06
16 − Imara
5 − Cascade Delight
30 − Sugana
42 − FEM_08
17 − Joan J
45 − FEM_11
2 − Amira
7 − Cowichan
37 − FEM_03
14 − Glen Fyne
3 − Anne
29 − Scepter
46 − FEM_12
28 − Ruby
8 − Enrosadira
43 − FEM_09
10 − Fall Red
4 − Autumn Treasure
41 − FEM_07
25 − Polka
27 − Regina
47 − FEM_13
36 − FEM_02
22 − Malling Minerva
18 − Kitsilano
6 − Castion
23 − Meeker
26 − Princess
33 − Versailles
38 − FEM_04
34 − Willamette
35 − FEM_01
32 − Valentina
39 − FEM_05
20 − Kwanza

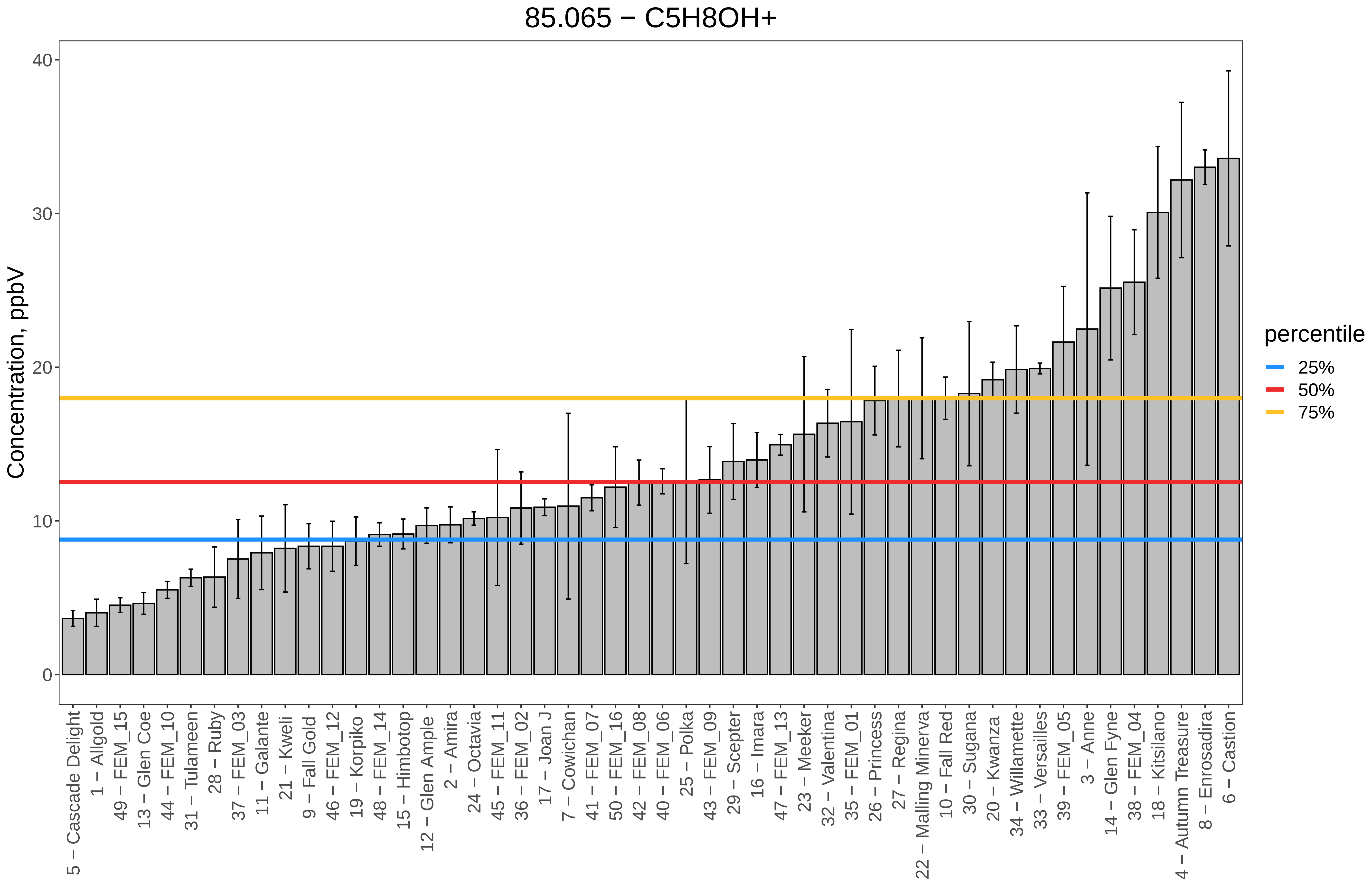

85.065 − C5H8OH+
Concentration, ppbV
0
10
20
30
40
percentile
25%
50%
75%
5 − Cascade Delight
1 − Allgold
49 − FEM_15
13 − Glen Coe
44 − FEM_10
31 − Tulameen
28 − Ruby
37 − FEM_03
11 − Galante
21 − Kweli
9 − Fall Gold
46 − FEM_12
19 − Korpiko
48 − FEM_14
15 − Himbotop
12 − Glen Ample
2 − Amira
24 − Octavia
45 − FEM_11
36 − FEM_02
17 − Joan J
7 − Cowichan
41 − FEM_07
50 − FEM_16
42 − FEM_08
40 − FEM_06
25 − Polka
43 − FEM_09
29 − Scepter
16 − Imara
47 − FEM_13
23 − Meeker
32 − Valentina
35 − FEM_01
26 − Princess
27 − Regina
22 − Malling Minerva
10 − Fall Red
30 − Sugana
20 − Kwanza
34 − Willamette
33 − Versailles
39 − FEM_05
3 − Anne
14 − Glen Fyne
38 − FEM_04
18 − Kitsilano
4 − Autumn Treasure
8 − Enrosadira
6 − Castion

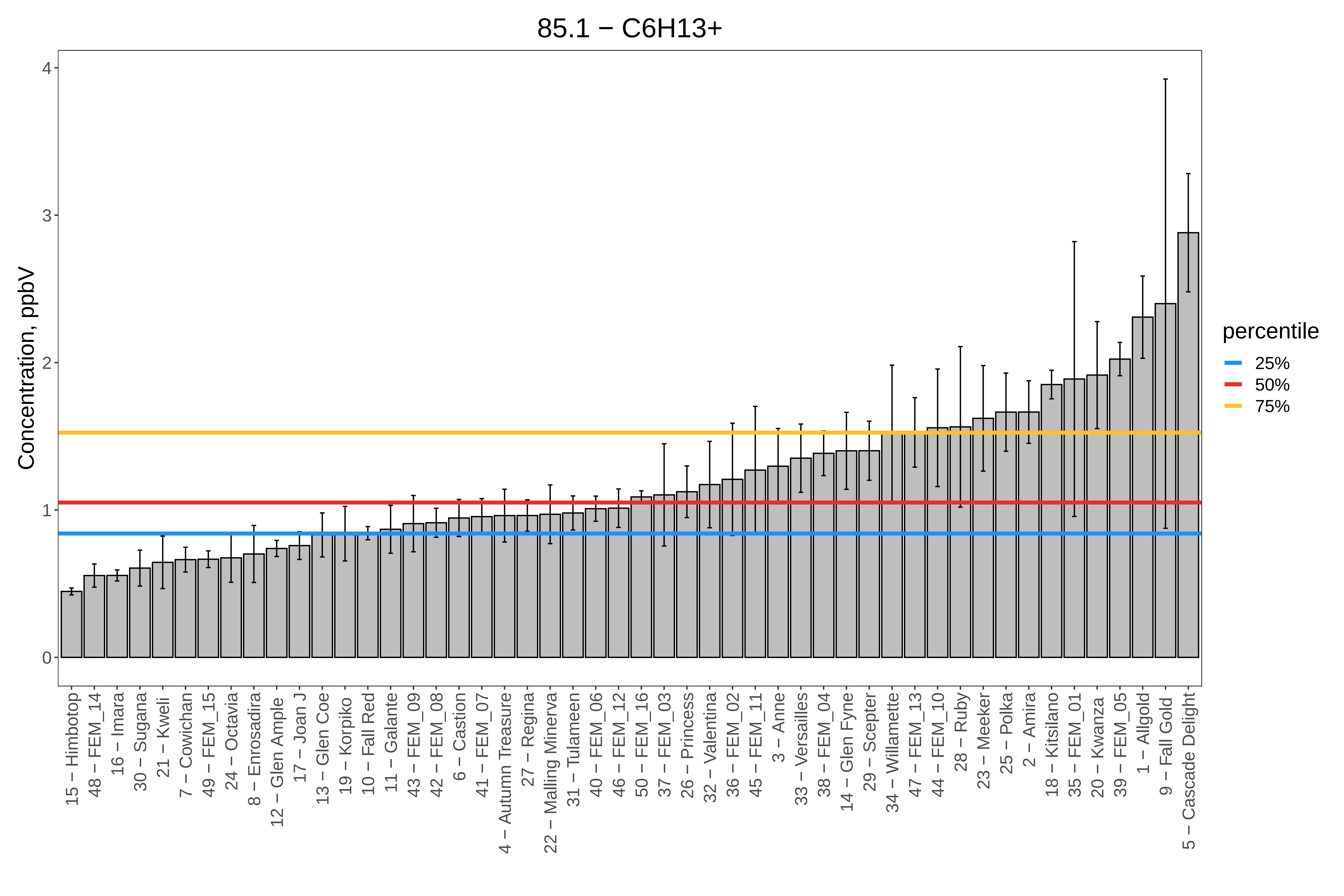

85.1 − C6H13+
Concentration, ppbV
0
1
2
3
4
percentile
25%
50%
75%
15 − Himbotop
48 − FEM_14
16 − Imara
30 − Sugana
21 − Kweli
7 − Cowichan
49 − FEM_15
24 − Octavia
8 − Enrosadira
12 − Glen Ample
17 − Joan J
13 − Glen Coe
19 − Korpiko
10 − Fall Red
11 − Galante
43 − FEM_09
42 − FEM_08
6 − Castion
41 − FEM_07
4 − Autumn Treasure
27 − Regina
22 − Malling Minerva
31 − Tulameen
40 − FEM_06
46 − FEM_12
50 − FEM_16
37 − FEM_03
26 − Princess
32 − Valentina
36 − FEM_02
45 − FEM_11
3 − Anne
33 − Versailles
38 − FEM_04
14 − Glen Fyne
29 − Scepter
34 − Willamette
47 − FEM_13
44 − FEM_10
28 − Ruby
23 − Meeker
25 − Polka
2 − Amira
18 − Kitsilano
35 − FEM_01
20 − Kwanza
39 − FEM_05
1 − Allgold
9 − Fall Gold
5 − Cascade Delight

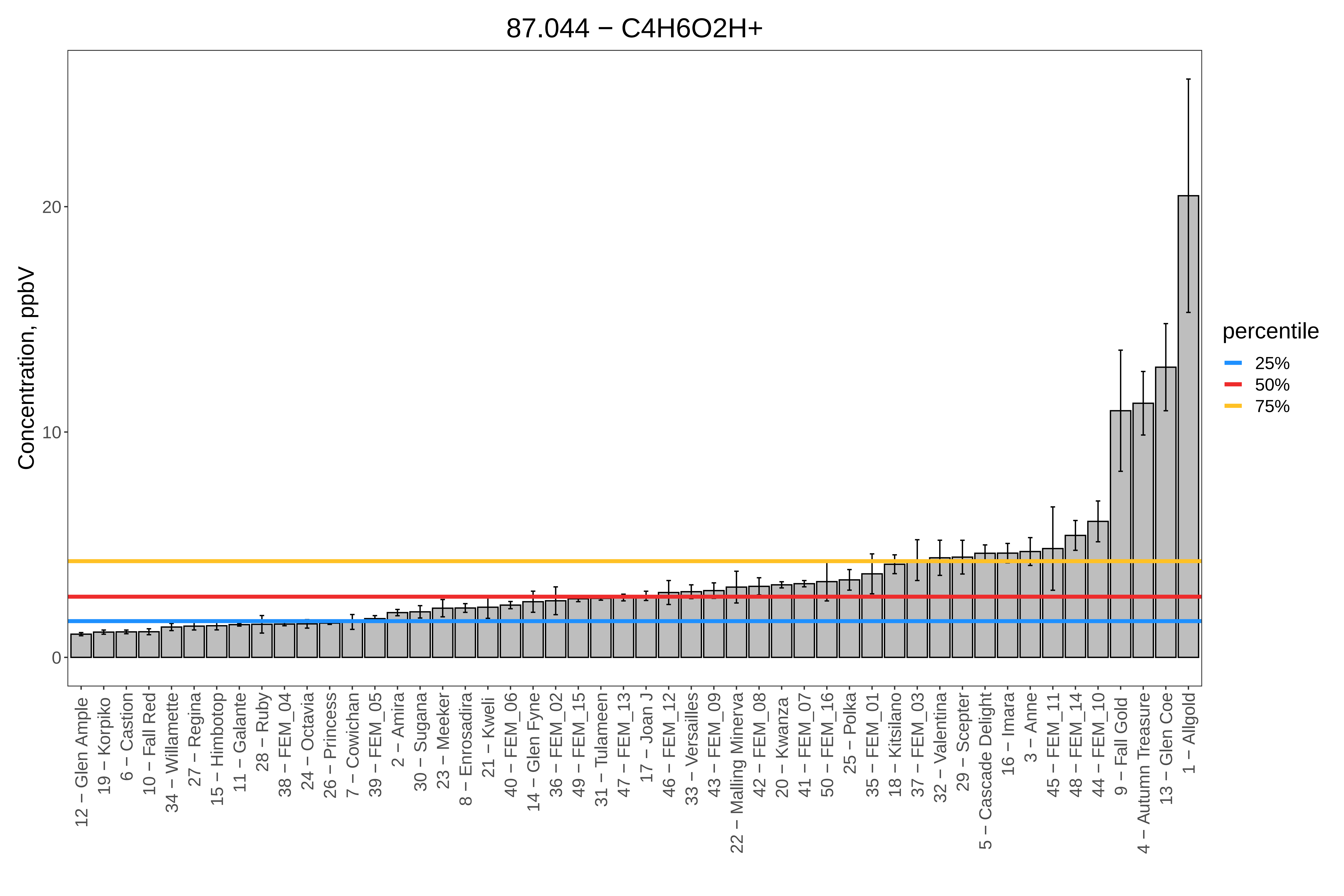
87.044 − C4H6O2H+
Concentration, ppbV
0
10
20
percentile
25%
50%
75%
12 − Glen Ample
19 − Korpiko
6 − Castion
10 − Fall Red
34 − Willamette
27 − Regina
15 − Himbotop
11 − Galante
28 − Ruby
38 − FEM_04
24 − Octavia
26 − Princess
7 − Cowichan
39 − FEM_05
2 − Amira
30 − Sugana
23 − Meeker
8 − Enrosadira
21 − Kweli
40 − FEM_06
14 − Glen Fyne
36 − FEM_02
49 − FEM_15
31 − Tulameen
47 − FEM_13
17 − Joan J
46 − FEM_12
33 − Versailles
43 − FEM_09
22 − Malling Minerva
42 − FEM_08
20 − Kwanza
41 − FEM_07
50 − FEM_16
25 − Polka
35 − FEM_01
18 − Kitsilano
37 − FEM_03
32 − Valentina
29 − Scepter
5 − Cascade Delight
16 − Imara
3 − Anne
45 − FEM_11
48 − FEM_14
44 − FEM_10
9 − Fall Gold
4 − Autumn Treasure
13 − Glen Coe
1 − Allgold

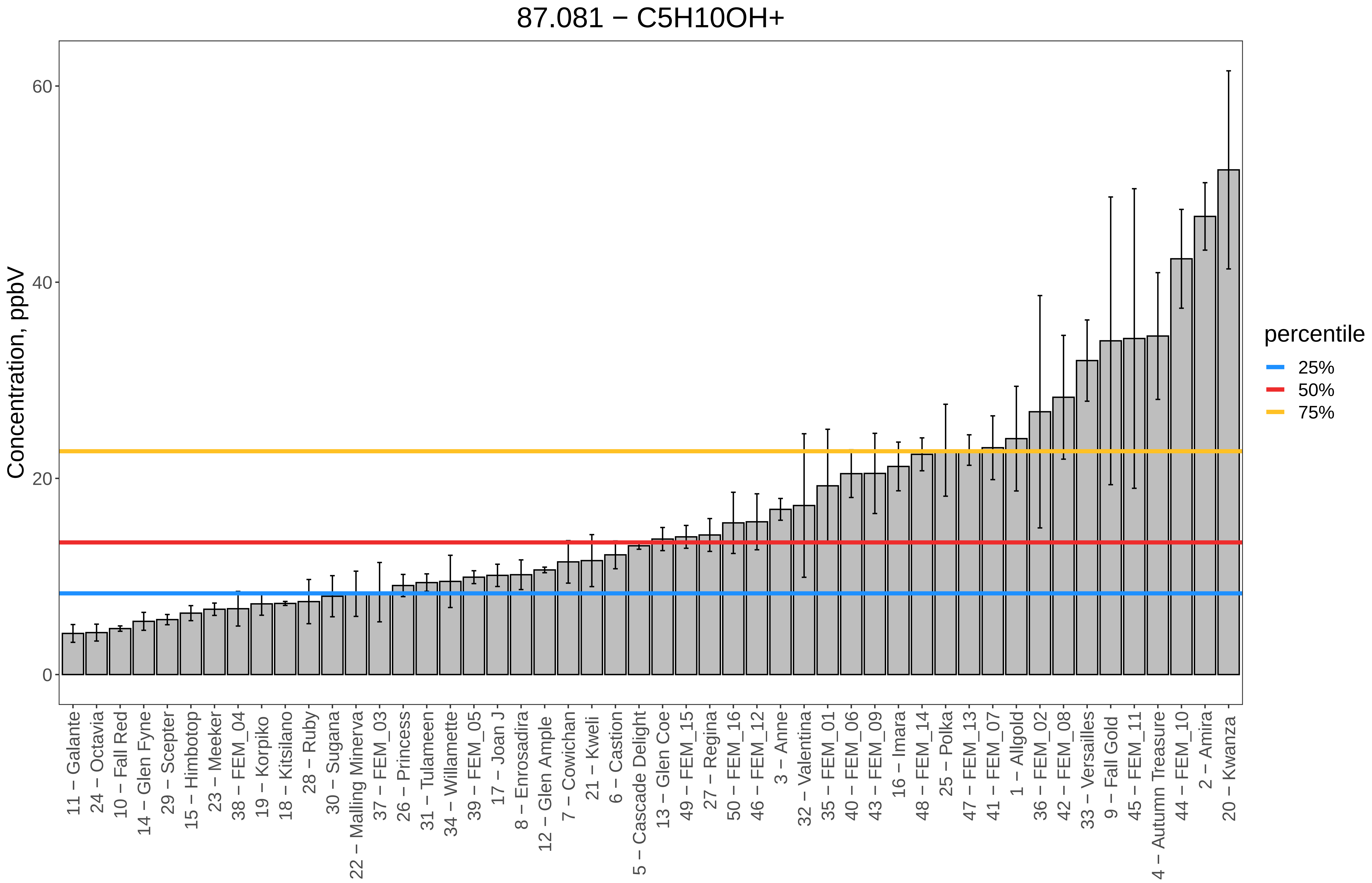

87.081 − C5H10OH+
Concentration, ppbV
0
20
40
60
percentile
25%
50%
75%
11 − Galante
24 − Octavia
10 − Fall Red
14 − Glen Fyne
29 − Scepter
15 − Himbotop
23 − Meeker
38 − FEM_04
19 − Korpiko
18 − Kitsilano
28 − Ruby
30 − Sugana
22 − Malling Minerva
37 − FEM_03
26 − Princess
31 − Tulameen
34 − Willamette
39 − FEM_05
17 − Joan J
8 − Enrosadira
12 − Glen Ample
7 − Cowichan
21 − Kweli
6 − Castion
5 − Cascade Delight
13 − Glen Coe
49 − FEM_15
27 − Regina
50 − FEM_16
46 − FEM_12
3 − Anne
32 − Valentina
35 − FEM_01
40 − FEM_06
43 − FEM_09
16 − Imara
48 − FEM_14
25 − Polka
47 − FEM_13
41 − FEM_07
1 − Allgold
36 − FEM_02
42 − FEM_08
33 − Versailles
9 − Fall Gold
45 − FEM_11
4 − Autumn Treasure
44 − FEM_10
2 − Amira
20 − Kwanza

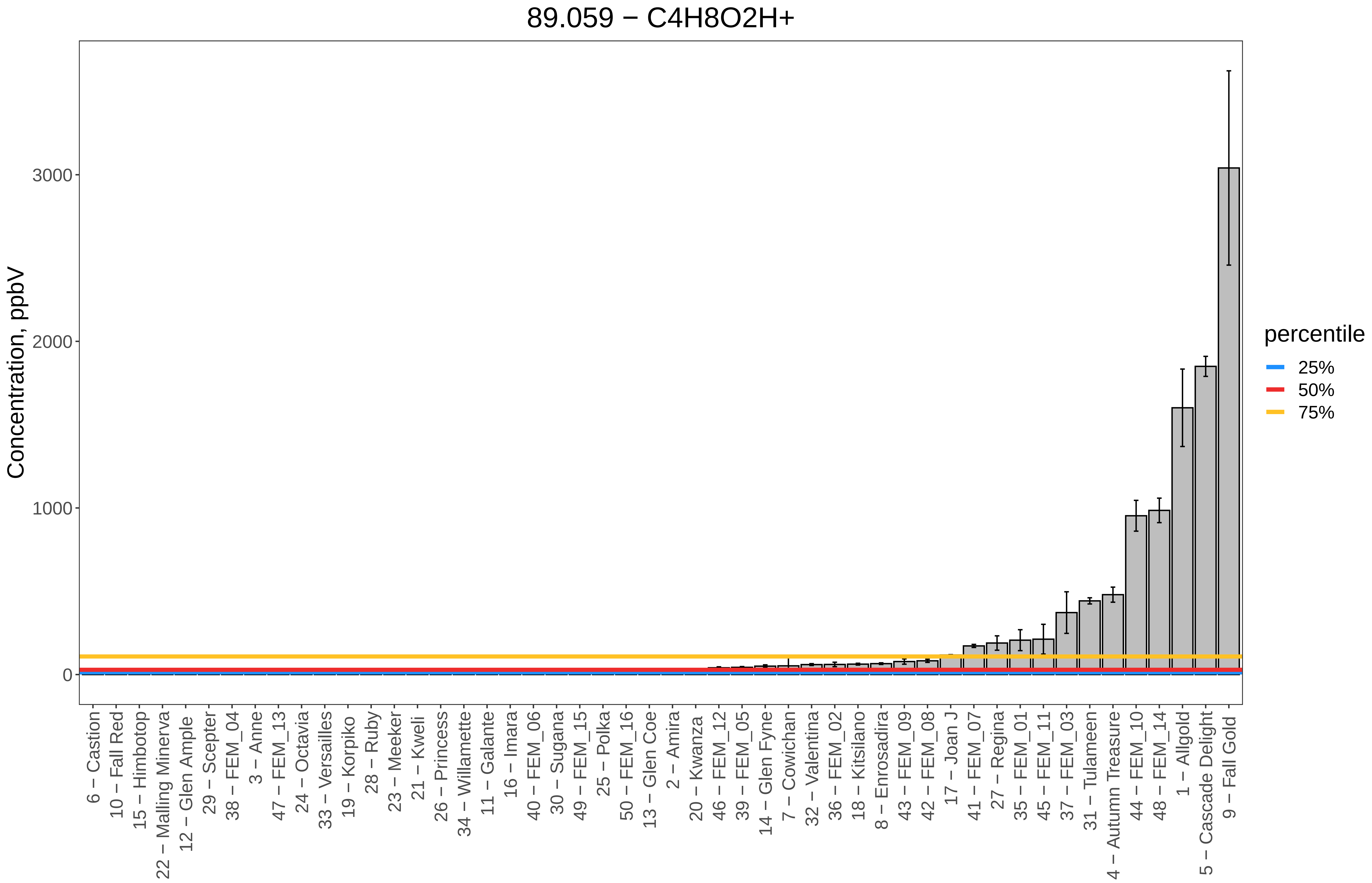
89.059 − C4H8O2H+
Concentration, ppbV
3000
2000
1000
0
percentile
25%
50%
75%
6 − Castion
10 − Fall Red
15 − Himbotop
22 − Malling Minerva
12 − Glen Ample
29 − Scepter
38 − FEM_04
3 − Anne
47 − FEM_13
24 − Octavia
33 − Versailles
19 − Korpiko
28 − Ruby
23 − Meeker
21 − Kweli
26 − Princess
34 − Willamette
11 − Galante
16 − Imara
40 − FEM_06
30 − Sugana
49 − FEM_15
25 − Polka
50 − FEM_16
13 − Glen Coe
2 − Amira
20 − Kwanza
46 − FEM_12
39 − FEM_05
14 − Glen Fyne
7 − Cowichan
32 − Valentina
36 − FEM_02
18 − Kitsilano
8 − Enrosadira
43 − FEM_09
42 − FEM_08
17 − Joan J
41 − FEM_07
27 − Regina
35 − FEM_01
45 − FEM_11
37 − FEM_03
31 − Tulameen
4 − Autumn Treasure
44 − FEM_10
48 − FEM_14
1 − Allgold
5 − Cascade Delight
9 − Fall Gold

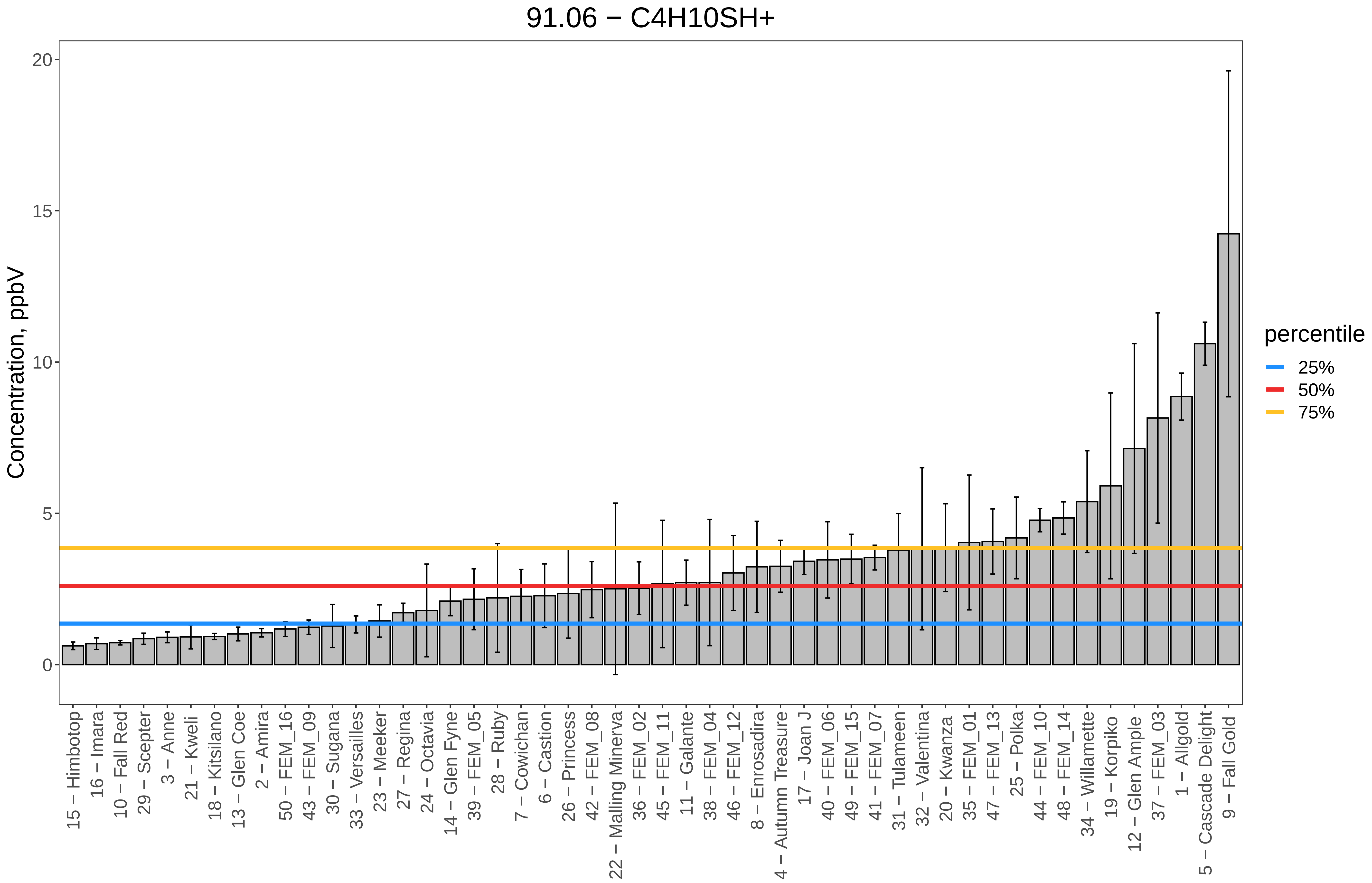
91.06 − C4H10SH+
Concentration, ppbV
0
5
10
15
20
percentile
25%
50%
75%
15 − Himbotop
16 − Imara
10 − Fall Red
29 − Scepter
3 − Anne
21 − Kweli
18 − Kitsilano
13 − Glen Coe
2 − Amira
50 − FEM_16
43 − FEM_09
30 − Sugana
33 − Versailles
23 − Meeker
27 − Regina
24 − Octavia
14 − Glen Fyne
39 − FEM_05
28 − Ruby
7 − Cowichan
6 − Castion
26 − Princess
42 − FEM_08
22 − Malling Minerva
36 − FEM_02
45 − FEM_11
11 − Galante
38 − FEM_04
46 − FEM_12
8 − Enrosadira
4 − Autumn Treasure
17 − Joan J
40 − FEM_06
49 − FEM_15
41 − FEM_07
31 − Tulameen
32 − Valentina
20 − Kwanza
35 − FEM_01
47 − FEM_13
25 − Polka
44 − FEM_10
48 − FEM_14
34 − Willamette
19 − Korpiko
12 − Glen Ample
37 − FEM_03
1 − Allgold
5 − Cascade Delight
9 − Fall Gold

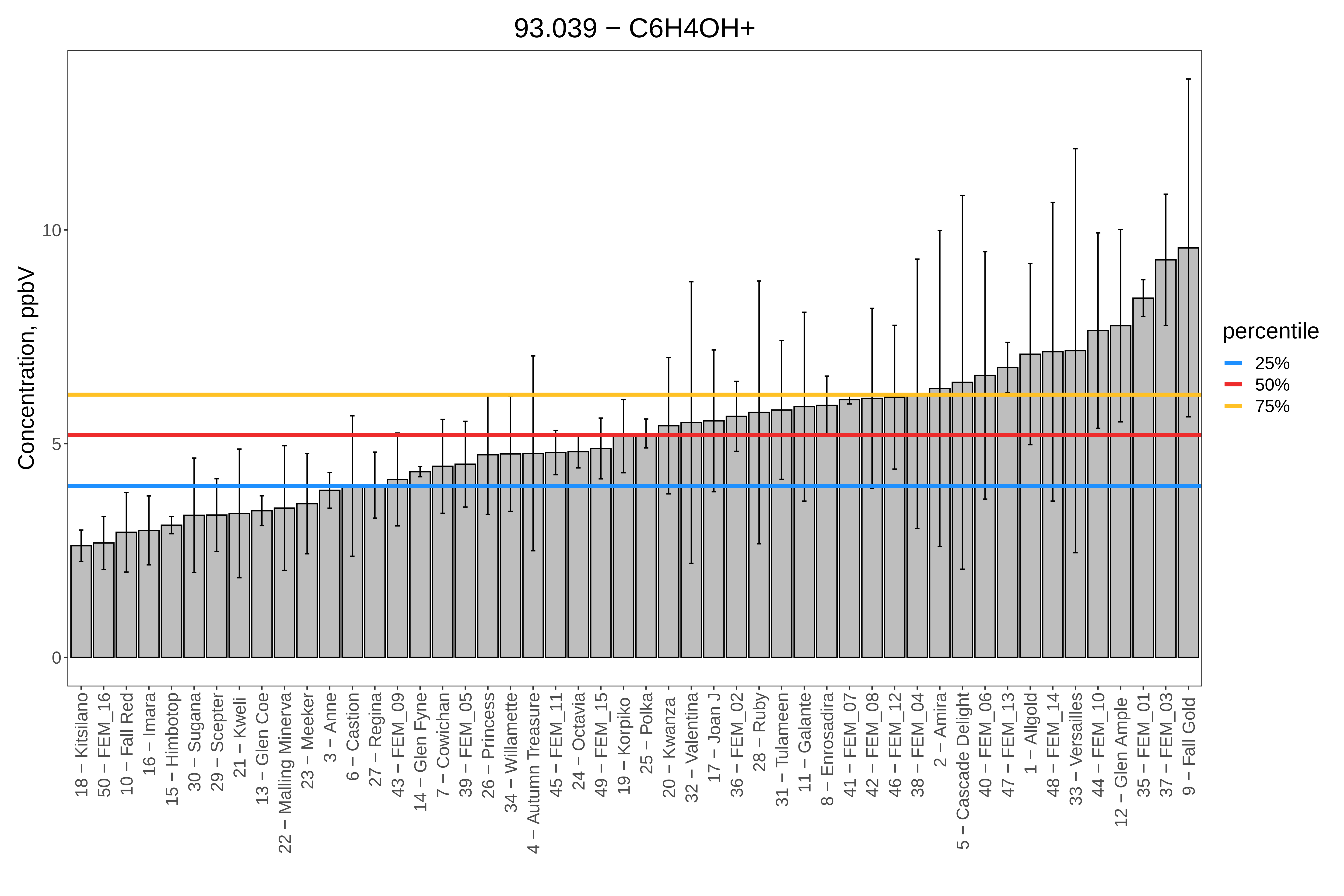

93.039 − C6H4OH+
Concentration, ppbV
0
5
10
percentile
25%
50%
75%
18 − Kitsilano
50 − FEM_16
10 − Fall Red
16 − Imara
15 − Himbotop
30 − Sugana
29 − Scepter
21 − Kweli
13 − Glen Coe
22 − Malling Minerva
23 − Meeker
3 − Anne
6 − Castion
27 − Regina
43 − FEM_09
14 − Glen Fyne
7 − Cowichan
39 − FEM_05
26 − Princess
34 − Willamette
4 − Autumn Treasure
45 − FEM_11
24 − Octavia
49 − FEM_15
19 − Korpiko
25 − Polka
20 − Kwanza
32 − Valentina
17 − Joan J
36 − FEM_02
28 − Ruby
31 − Tulameen
11 − Galante
8 − Enrosadira
41 − FEM_07
42 − FEM_08
46 − FEM_12
38 − FEM_04
2 − Amira
5 − Cascade Delight
40 − FEM_06
47 − FEM_13
1 − Allgold
48 − FEM_14
33 − Versailles
44 − FEM_10
12 − Glen Ample
35 − FEM_01
37 − FEM_03
9 − Fall Gold

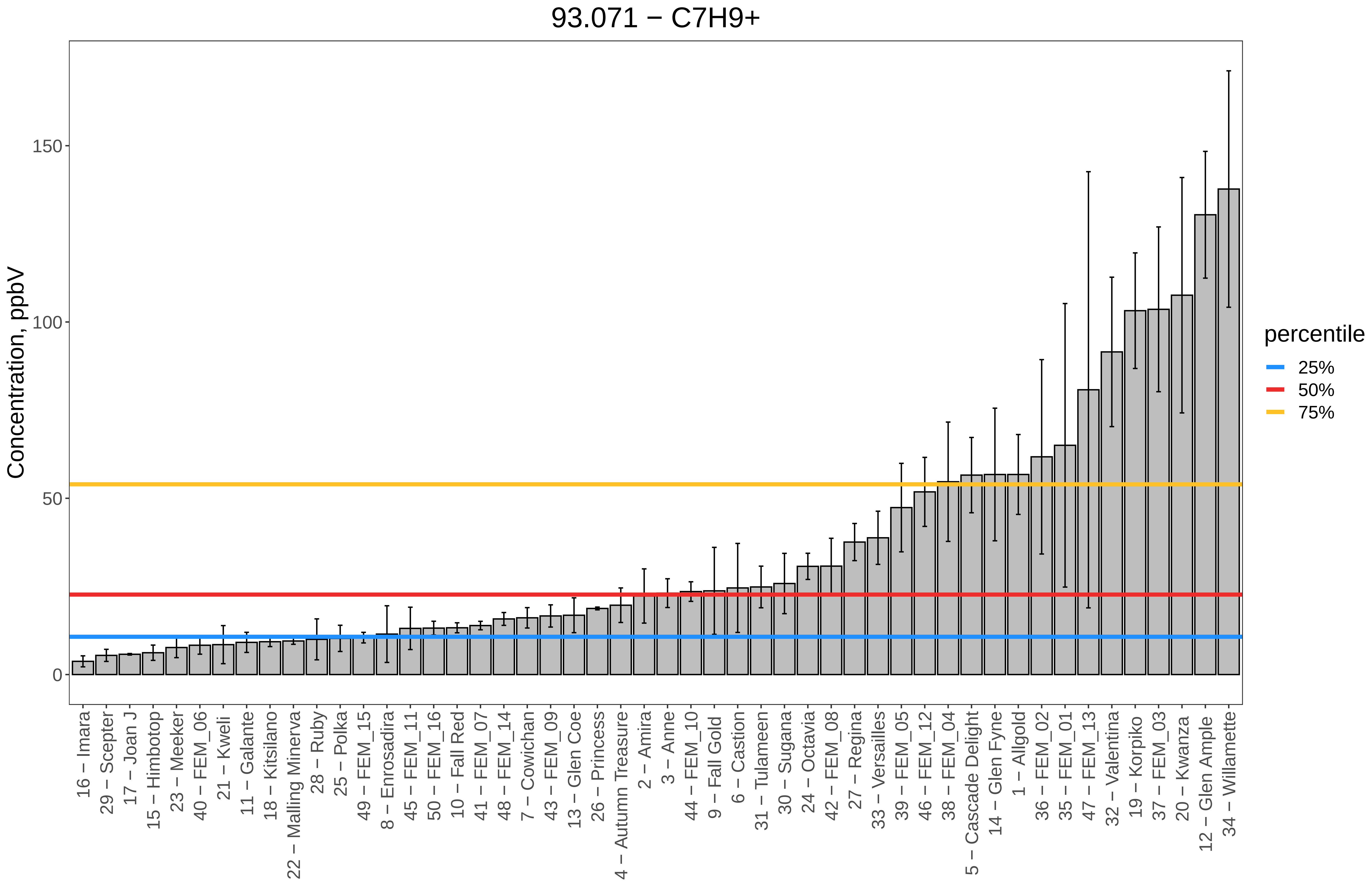

93.071 − C7H9+
Concentration, ppbV
0
50
100
150
percentile
25%
50%
75%
16 − Imara
29 − Scepter
17 − Joan J
15 − Himbotop
23 − Meeker
40 − FEM_06
21 − Kweli
11 − Galante
18 − Kitsilano
22 − Malling Minerva
28 − Ruby
25 − Polka
49 − FEM_15
8 − Enrosadira
45 − FEM_11
50 − FEM_16
10 − Fall Red
41 − FEM_07
48 − FEM_14
7 − Cowichan
43 − FEM_09
13 − Glen Coe
26 − Princess
4 − Autumn Treasure
2 − Amira
3 − Anne
44 − FEM_10
9 − Fall Gold
6 − Castion
31 − Tulameen
30 − Sugana
24 − Octavia
42 − FEM_08
27 − Regina
33 − Versailles
39 − FEM_05
46 − FEM_12
38 − FEM_04
5 − Cascade Delight
14 − Glen Fyne
1 − Allgold
36 − FEM_02
35 − FEM_01
47 − FEM_13
32 − Valentina
19 − Korpiko
37 − FEM_03
20 − Kwanza
12 − Glen Ample
34 − Willamette

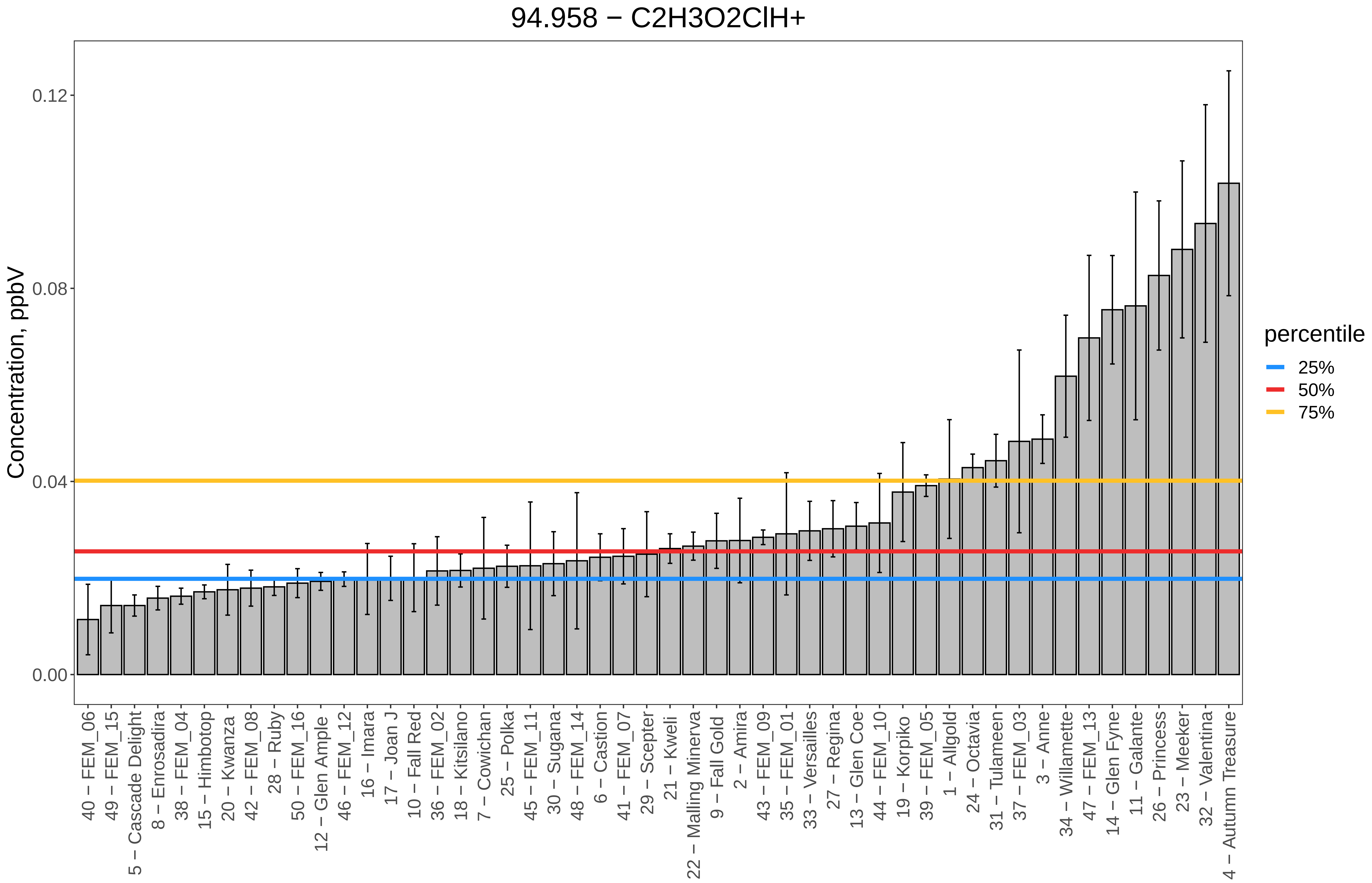

94.958 − C2H3O2ClH+
Concentration, ppbV
0.00
0.04
0.08
0.12
percentile
25%
50%
75%
40 − FEM_06
49 − FEM_15
5 − Cascade Delight
8 − Enrosadira
38 − FEM_04
15 − Himbotop
20 − Kwanza
42 − FEM_08
28 − Ruby
50 − FEM_16
12 − Glen Ample
46 − FEM_12
16 − Imara
17 − Joan J
10 − Fall Red
36 − FEM_02
18 − Kitsilano
7 − Cowichan
25 − Polka
45 − FEM_11
30 − Sugana
48 − FEM_14
6 − Castion
41 − FEM_07
29 − Scepter
21 − Kweli
22 − Malling Minerva
9 − Fall Gold
2 − Amira
43 − FEM_09
35 − FEM_01
33 − Versailles
27 − Regina
13 − Glen Coe
44 − FEM_10
19 − Korpiko
39 − FEM_05
1 − Allgold
24 − Octavia
31 − Tulameen
37 − FEM_03
3 − Anne
34 − Willamette
47 − FEM_13
14 − Glen Fyne
11 − Galante
26 − Princess
23 − Meeker
32 − Valentina
4 − Autumn Treasure

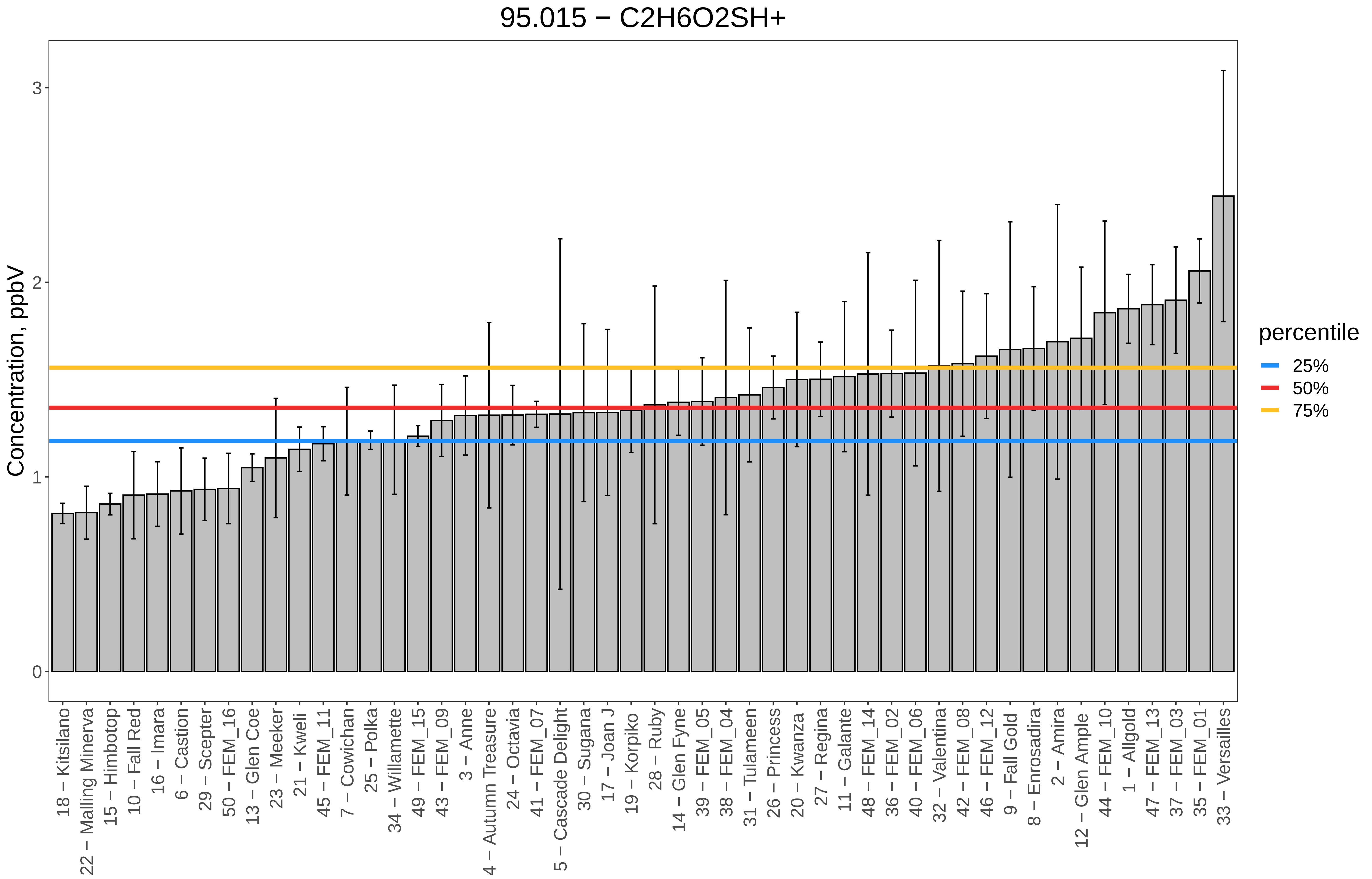

95.015 − C2H6O2SH+
Concentration, ppbV
0
1
2
3
percentile
25%
50%
75%
18 − Kitsilano
22 − Malling Minerva
15 − Himbotop
10 − Fall Red
16 − Imara
6 − Castion
29 − Scepter
50 − FEM_16
13 − Glen Coe
23 − Meeker
21 − Kweli
45 − FEM_11
7 − Cowichan
25 − Polka
34 − Willamette
49 − FEM_15
43 − FEM_09
3 − Anne
4 − Autumn Treasure
24 − Octavia
41 − FEM_07
5 − Cascade Delight
30 − Sugana
17 − Joan J
19 − Korpiko
28 − Ruby
14 − Glen Fyne
39 − FEM_05
38 − FEM_04
31 − Tulameen
26 − Princess
20 − Kwanza
27 − Regina
11 − Galante
48 − FEM_14
36 − FEM_02
40 − FEM_06
32 − Valentina
42 − FEM_08
46 − FEM_12
9 − Fall Gold
8 − Enrosadira
2 − Amira
12 − Glen Ample
44 − FEM_10
1 − Allgold
47 − FEM_13
37 − FEM_03
35 − FEM_01
33 − Versailles

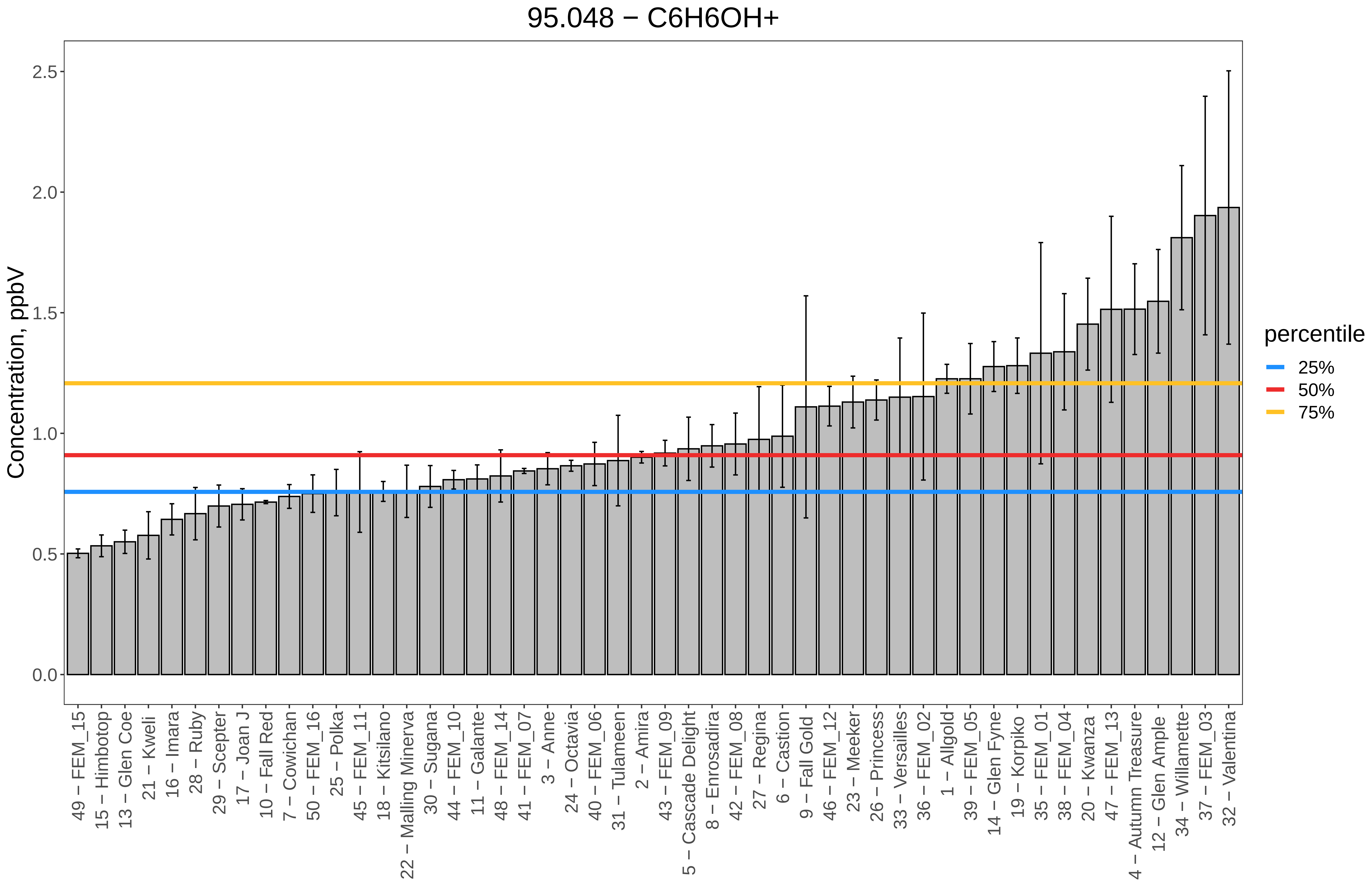

95.048 − C6H6OH+
Concentration, ppbV
0.0
0.5
1.0
1.5
2.0
2.5
percentile
25%
50%
75%
49 − FEM_15
15 − Himbotop
13 − Glen Coe
21 − Kweli
16 − Imara
28 − Ruby
29 − Scepter
17 − Joan J
10 − Fall Red
7 − Cowichan
50 − FEM_16
25 − Polka
45 − FEM_11
18 − Kitsilano
22 − Malling Minerva
30 − Sugana
44 − FEM_10
11 − Galante
48 − FEM_14
41 − FEM_07
3 − Anne
24 − Octavia
40 − FEM_06
31 − Tulameen
2 − Amira
43 − FEM_09
5 − Cascade Delight
8 − Enrosadira
42 − FEM_08
27 − Regina
6 − Castion
9 − Fall Gold
46 − FEM_12
23 − Meeker
26 − Princess
33 − Versailles
36 − FEM_02
1 − Allgold
39 − FEM_05
14 − Glen Fyne
19 − Korpiko
35 − FEM_01
38 − FEM_04
20 − Kwanza
47 − FEM_13
4 − Autumn Treasure
12 − Glen Ample
34 − Willamette
37 − FEM_03
32 − Valentina

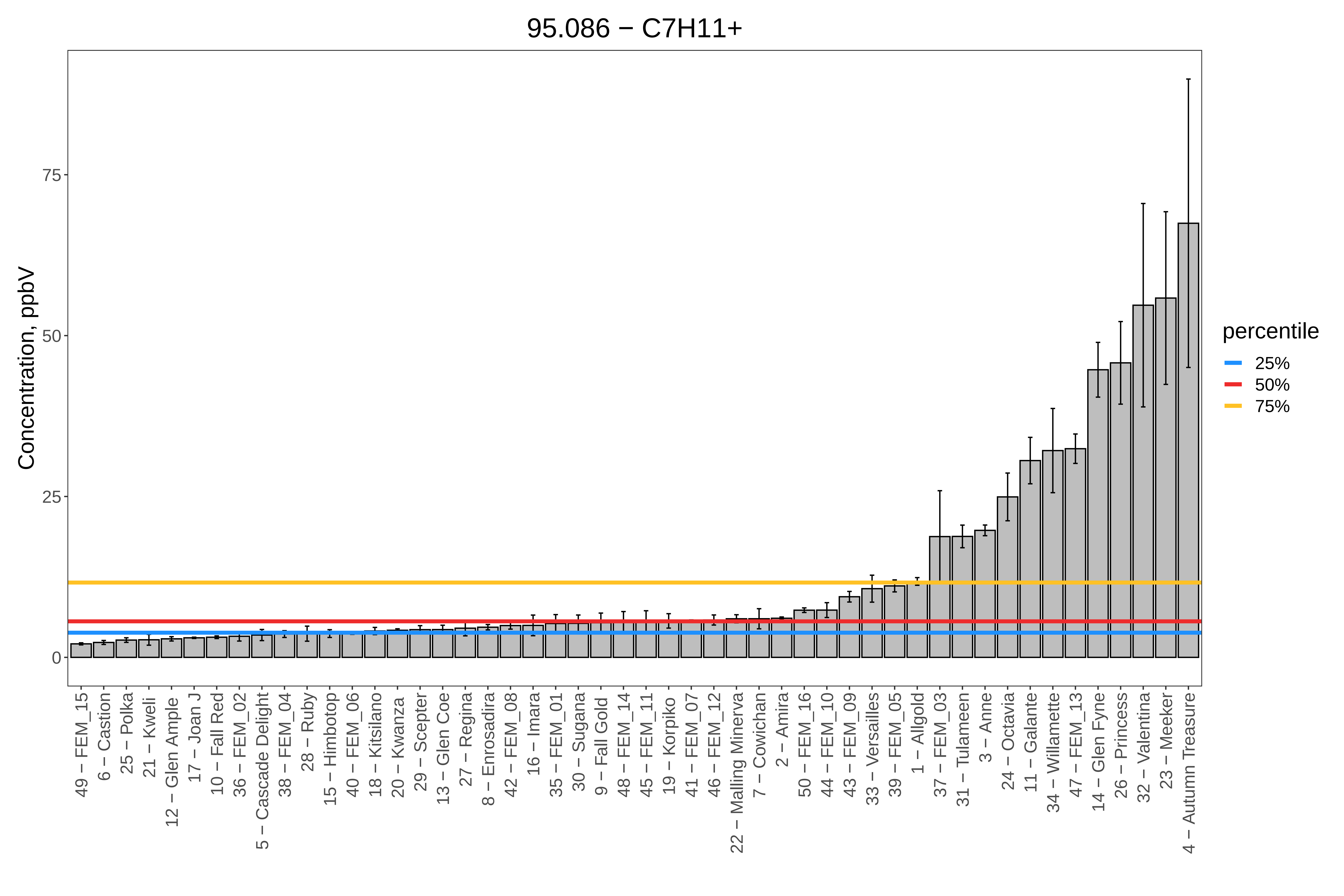
95.086 − C7H11+
Concentration, ppbV
0
25
50
75
percentile
25%
50%
75%
49 − FEM_15
6 − Castion
25 − Polka
21 − Kweli
12 − Glen Ample
17 − Joan J
10 − Fall Red
36 − FEM_02
5 − Cascade Delight
38 − FEM_04
28 − Ruby
15 − Himbotop
40 − FEM_06
18 − Kitsilano
20 − Kwanza
29 − Scepter
13 − Glen Coe
27 − Regina
8 − Enrosadira
42 − FEM_08
16 − Imara
35 − FEM_01
30 − Sugana
9 − Fall Gold
48 − FEM_14
45 − FEM_11
19 − Korpiko
41 − FEM_07
46 − FEM_12
22 − Malling Minerva
7 − Cowichan
2 − Amira
50 − FEM_16
44 − FEM_10
43 − FEM_09
33 − Versailles
39 − FEM_05
1 − Allgold
37 − FEM_03
31 − Tulameen
3 − Anne
24 − Octavia
11 − Galante
34 − Willamette
47 − FEM_13
14 − Glen Fyne
26 − Princess
32 − Valentina
23 − Meeker
4 − Autumn Treasure

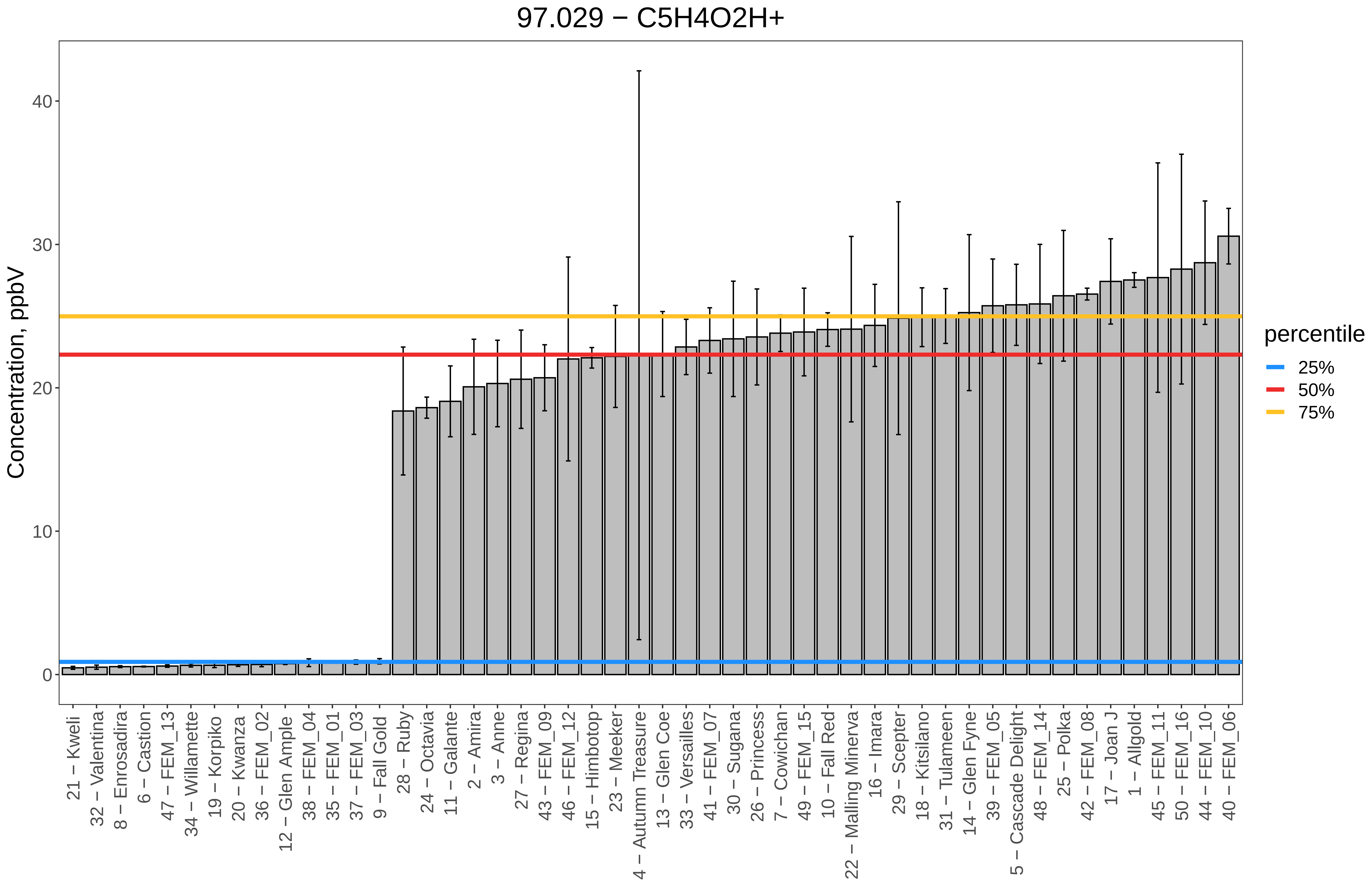

97.029 − C5H4O2H+
Concentration, ppbV
0
10
20
30
40
percentile
25%
50%
75%
21 − Kweli
32 − Valentina
8 − Enrosadira
6 − Castion
47 − FEM_13
34 − Willamette
19 − Korpiko
20 − Kwanza
36 − FEM_02
12 − Glen Ample
38 − FEM_04
35 − FEM_01
37 − FEM_03
9 − Fall Gold
28 − Ruby
24 − Octavia
11 − Galante
2 − Amira
3 − Anne
27 − Regina
43 − FEM_09
46 − FEM_12
15 − Himbotop
23 − Meeker
4 − Autumn Treasure
13 − Glen Coe
33 − Versailles
41 − FEM_07
30 − Sugana
26 − Princess
7 − Cowichan
49 − FEM_15
10 − Fall Red
22 − Malling Minerva
16 − Imara
29 − Scepter
18 − Kitsilano
31 − Tulameen
14 − Glen Fyne
39 − FEM_05
5 − Cascade Delight
48 − FEM_14
25 − Polka
42 − FEM_08
17 − Joan J
1 − Allgold
45 − FEM_11
50 − FEM_16
44 − FEM_10
40 − FEM_06

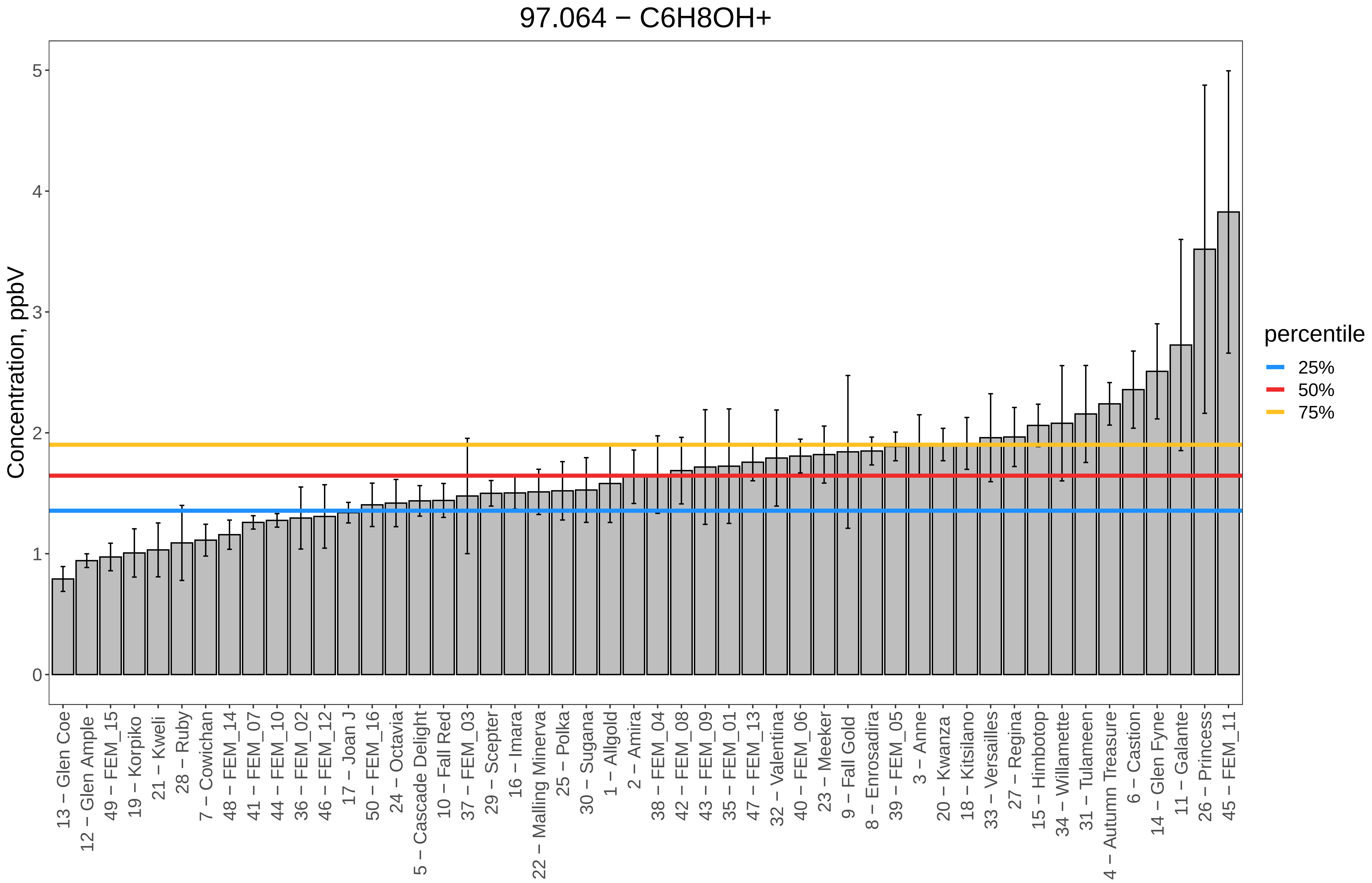

97.064 − C6H8OH+
Concentration, ppbV
0
1
2
3
4
5
percentile
25%
50%
75%
13 − Glen Coe
12 − Glen Ample
49 − FEM_15
19 − Korpiko
21 − Kweli
28 − Ruby
7 − Cowichan
48 − FEM_14
41 − FEM_07
44 − FEM_10
36 − FEM_02
46 − FEM_12
17 − Joan J
50 − FEM_16
24 − Octavia
5 − Cascade Delight
10 − Fall Red
37 − FEM_03
29 − Scepter
16 − Imara
22 − Malling Minerva
25 − Polka
30 − Sugana
1 − Allgold
2 − Amira
38 − FEM_04
42 − FEM_08
43 − FEM_09
35 − FEM_01
47 − FEM_13
32 − Valentina
40 − FEM_06
23 − Meeker
9 − Fall Gold
8 − Enrosadira
39 − FEM_05
3 − Anne
20 − Kwanza
18 − Kitsilano
33 − Versailles
27 − Regina
15 − Himbotop
34 − Willamette
31 − Tulameen
4 − Autumn Treasure
6 − Castion
14 − Glen Fyne
11 − Galante
26 − Princess
45 − FEM_11

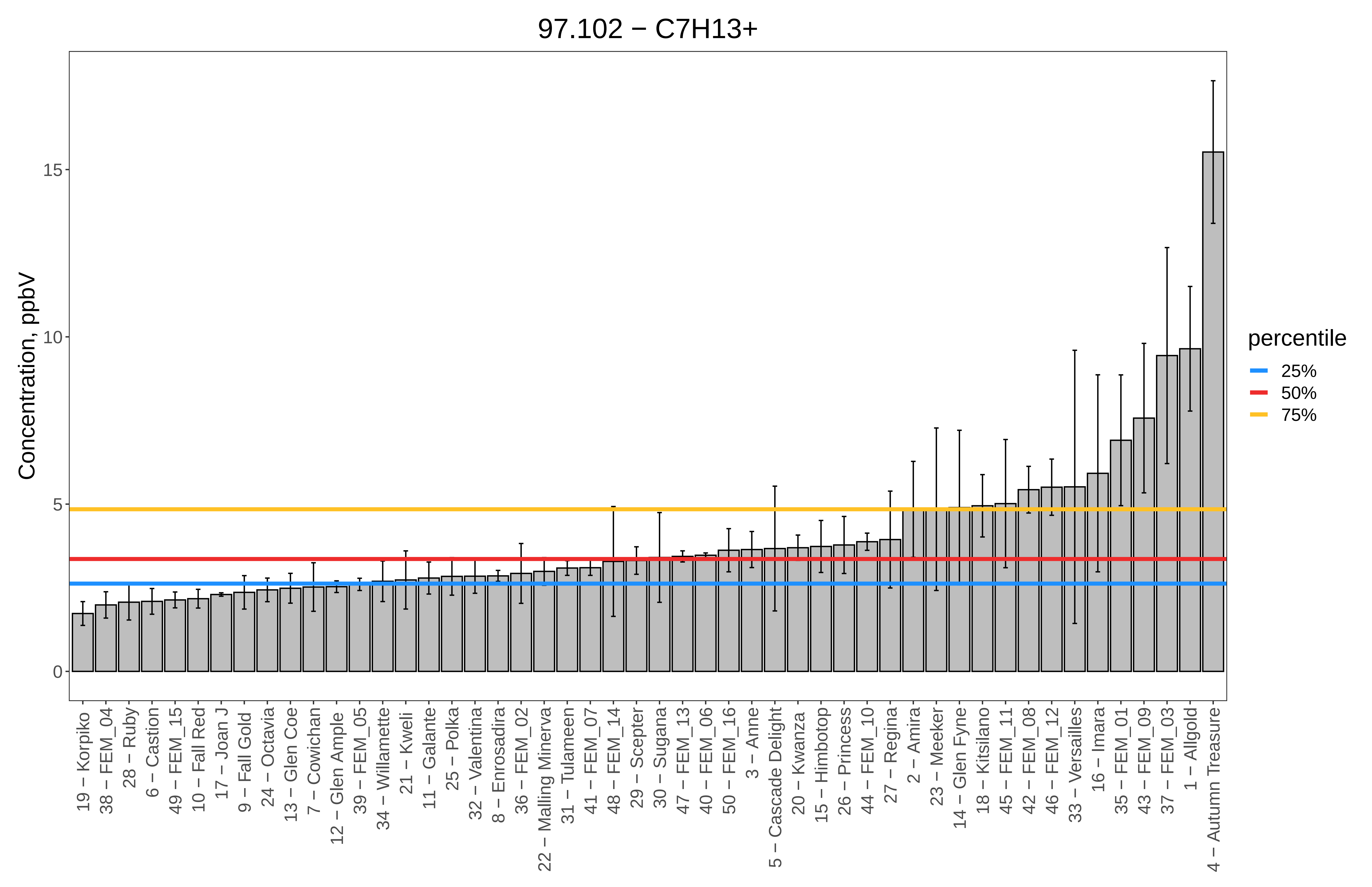
97.102 − C7H13+
Concentration, ppbV
0
5
10
15
percentile
25%
50%
75%
19 − Korpiko
38 − FEM_04
28 − Ruby
6 − Castion
49 − FEM_15
10 − Fall Red
17 − Joan J
9 − Fall Gold
24 − Octavia
13 − Glen Coe
7 − Cowichan
12 − Glen Ample
39 − FEM_05
34 − Willamette
21 − Kweli
11 − Galante
25 − Polka
32 − Valentina
8 − Enrosadira
36 − FEM_02
22 − Malling Minerva
31 − Tulameen
41 − FEM_07
48 − FEM_14
29 − Scepter
30 − Sugana
47 − FEM_13
40 − FEM_06
50 − FEM_16
3 − Anne
5 − Cascade Delight
20 − Kwanza
15 − Himbotop
26 − Princess
44 − FEM_10
27 − Regina
2 − Amira
23 − Meeker
14 − Glen Fyne
18 − Kitsilano
45 − FEM_11
42 − FEM_08
46 − FEM_12
33 − Versailles
16 − Imara
35 − FEM_01
43 − FEM_09
37 − FEM_03
1 − Allgold
4 − Autumn Treasure

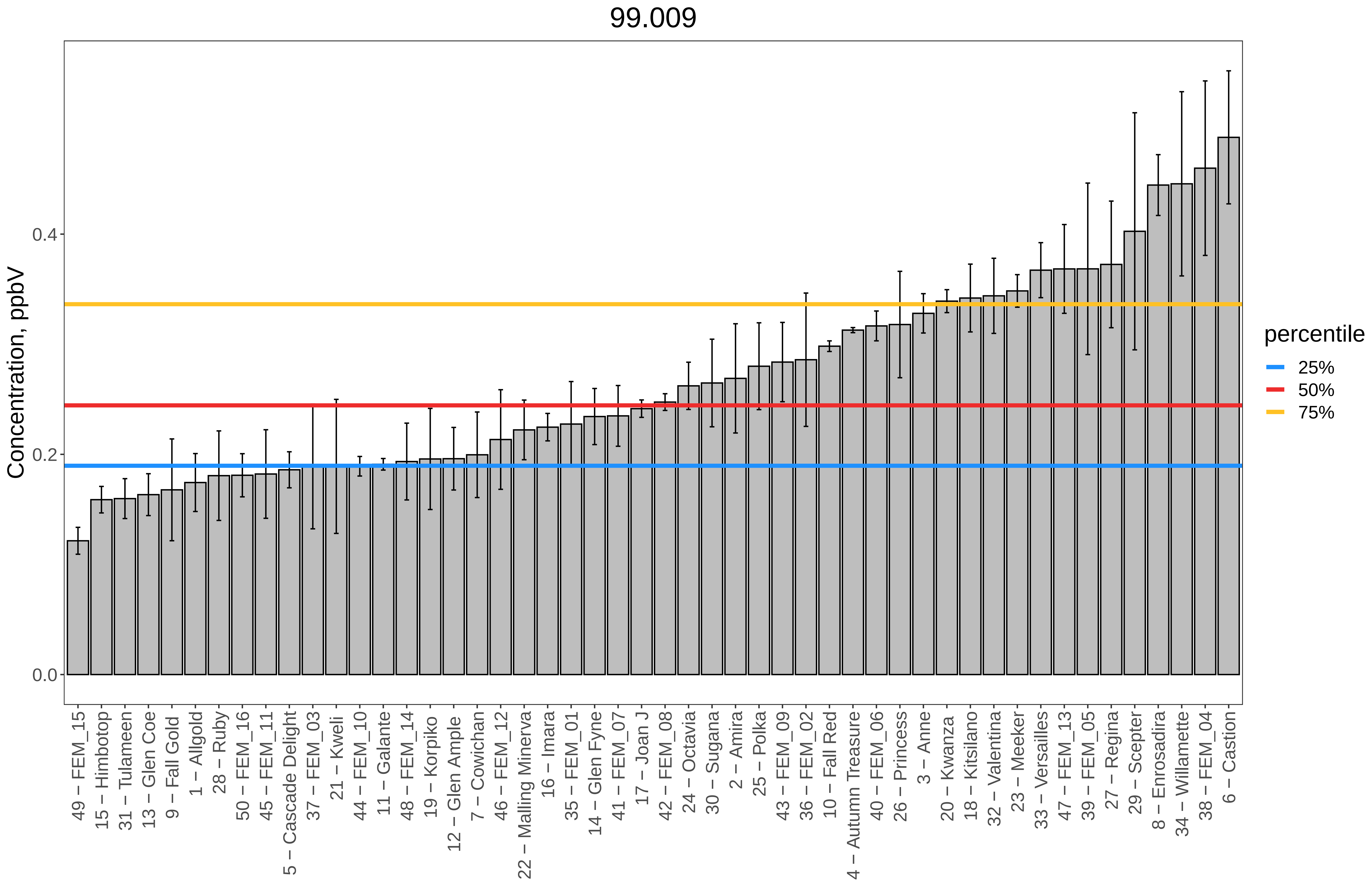

99.009
Concentration, ppbV
0.0
0.2
0.4
percentile
25%
50%
75%
49 – FEM_15
15 – Himbotop
31 – Tulameen
13 – Glen Coe
9 – Fall Gold
1 – Allgold
28 – Ruby
50 – FEM_16
45 – FEM_11
5 – Cascade Delight
37 – FEM_03
21 – Kweli
44 – FEM_10
11 – Galante
48 – FEM_14
19 – Korpiko
12 – Glen Ample
7 – Cowichan
46 – FEM_12
22 – Malling Minerva
16 – Imara
35 – FEM_01
14 – Glen Fyne
41 – FEM_07
17 – Joan J
42 – FEM_08
24 – Octavia
30 – Sugana
2 – Amira
25 – Polka
43 – FEM_09
36 – FEM_02
10 – Fall Red
4 – Autumn Treasure
40 – FEM_06
26 – Princess
3 – Anne
20 – Kwanza
18 – Kitsilano
32 – Valentina
23 – Meeker
33 – Versailles
47 – FEM_13
39 – FEM_05
27 – Regina
29 – Scepter
8 – Enrosadira
34 – Willamette
38 – FEM_04
6 – Castion

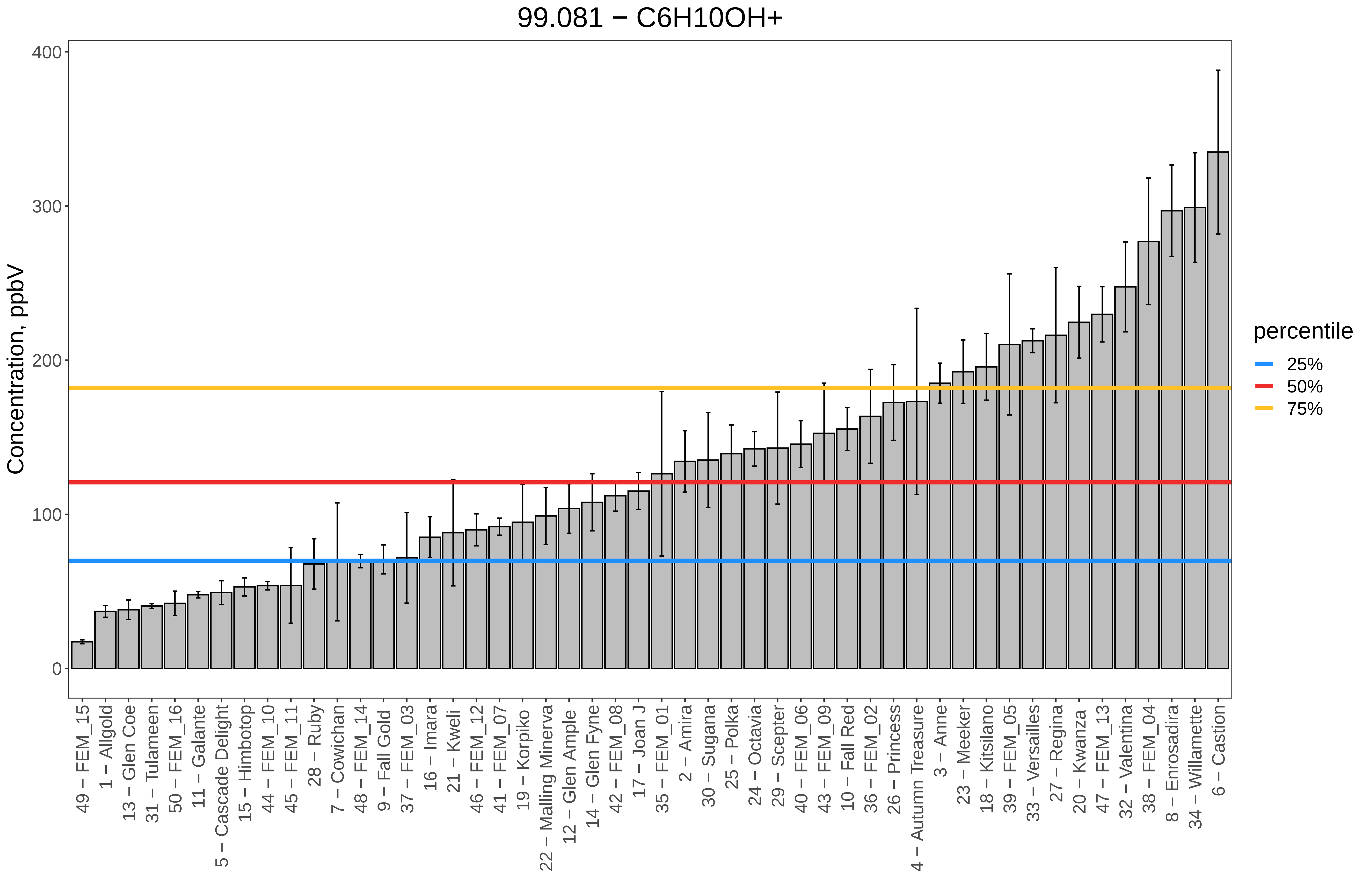

99.081 − C6H10OH+
Concentration, ppbV
0
100
200
300
400
percentile
25%
50%
75%
49 − FEM_15
1 − Allgold
13 − Glen Coe
31 − Tulameen
50 − FEM_16
11 − Galante
5 − Cascade Delight
15 − Himbotop
44 − FEM_10
45 − FEM_11
28 − Ruby
7 − Cowichan
48 − FEM_14
9 − Fall Gold
37 − FEM_03
16 − Imara
21 − Kweli
46 − FEM_12
41 − FEM_07
19 − Korpiko
22 − Malling Minerva
12 − Glen Ample
14 − Glen Fyne
42 − FEM_08
17 − Joan J
35 − FEM_01
2 − Amira
30 − Sugana
25 − Polka
24 − Octavia
29 − Scepter
40 − FEM_06
43 − FEM_09
10 − Fall Red
36 − FEM_02
26 − Princess
4 − Autumn Treasure
3 − Anne
23 − Meeker
18 − Kitsilano
39 − FEM_05
33 − Versailles
27 − Regina
20 − Kwanza
47 − FEM_13
32 − Valentina
38 − FEM_04
8 − Enrosadira
34 − Willamette
6 − Castion

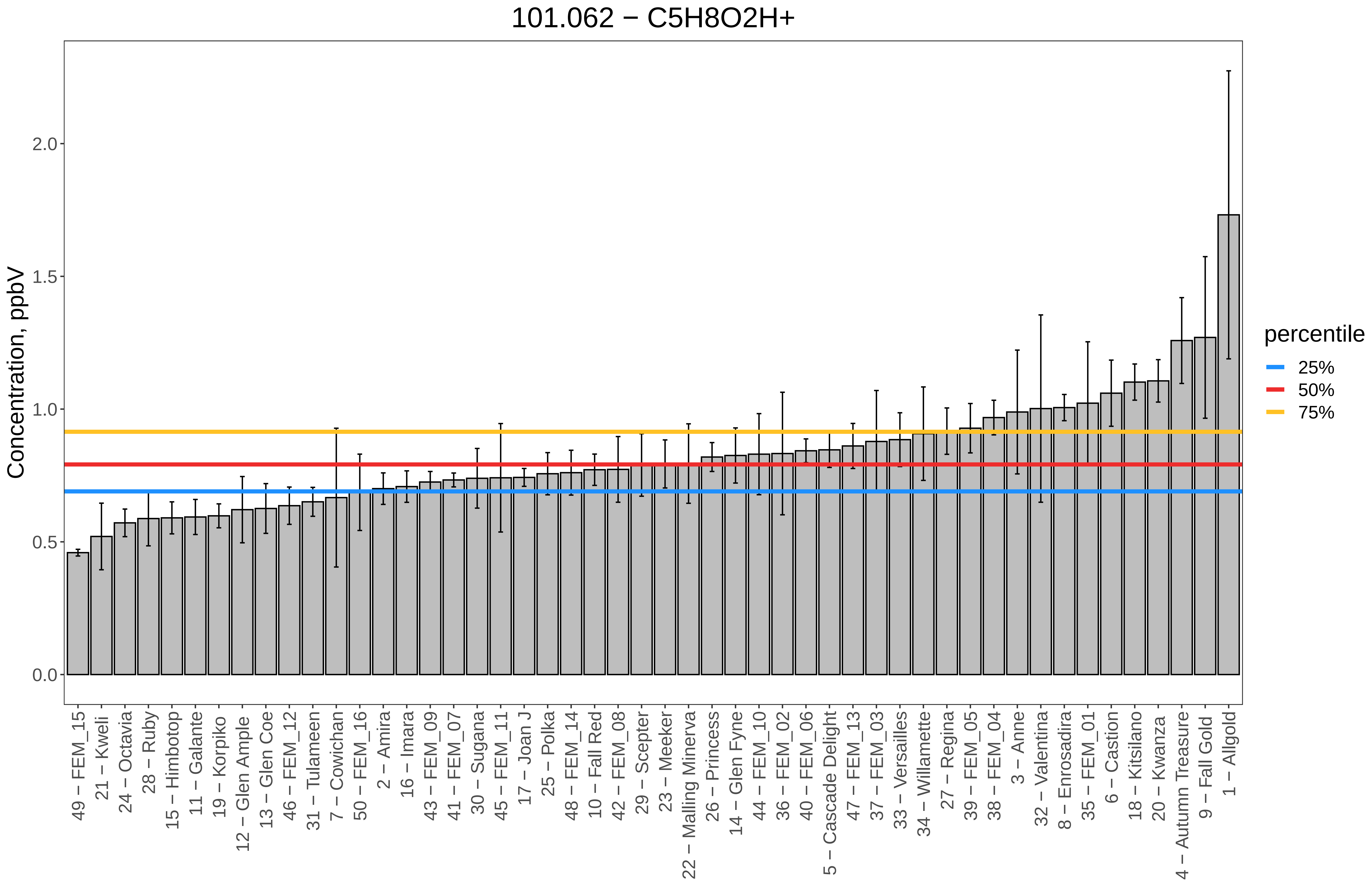

101.062 − C5H8O2H+
Concentration, ppbV
0.0
0.5
1.0
1.5
2.0
percentile
25%
50%
75%
49 − FEM_15
21 − Kweli
24 − Octavia
28 − Ruby
15 − Himbotop
11 − Galante
19 − Korpiko
12 − Glen Ample
13 − Glen Coe
46 − FEM_12
31 − Tulameen
7 − Cowichan
50 − FEM_16
2 − Amira
16 − Imara
43 − FEM_09
41 − FEM_07
30 − Sugana
45 − FEM_11
17 − Joan J
25 − Polka
48 − FEM_14
10 − Fall Red
42 − FEM_08
29 − Scepter
23 − Meeker
22 − Malling Minerva
26 − Princess
14 − Glen Fyne
44 − FEM_10
36 − FEM_02
40 − FEM_06
5 − Cascade Delight
47 − FEM_13
37 − FEM_03
33 − Versailles
34 − Willamette
27 − Regina
39 − FEM_05
38 − FEM_04
3 − Anne
32 − Valentina
8 − Enrosadira
35 − FEM_01
6 − Castion
18 − Kitsilano
20 − Kwanza
4 − Autumn Treasure
9 − Fall Gold
1 − Allgold

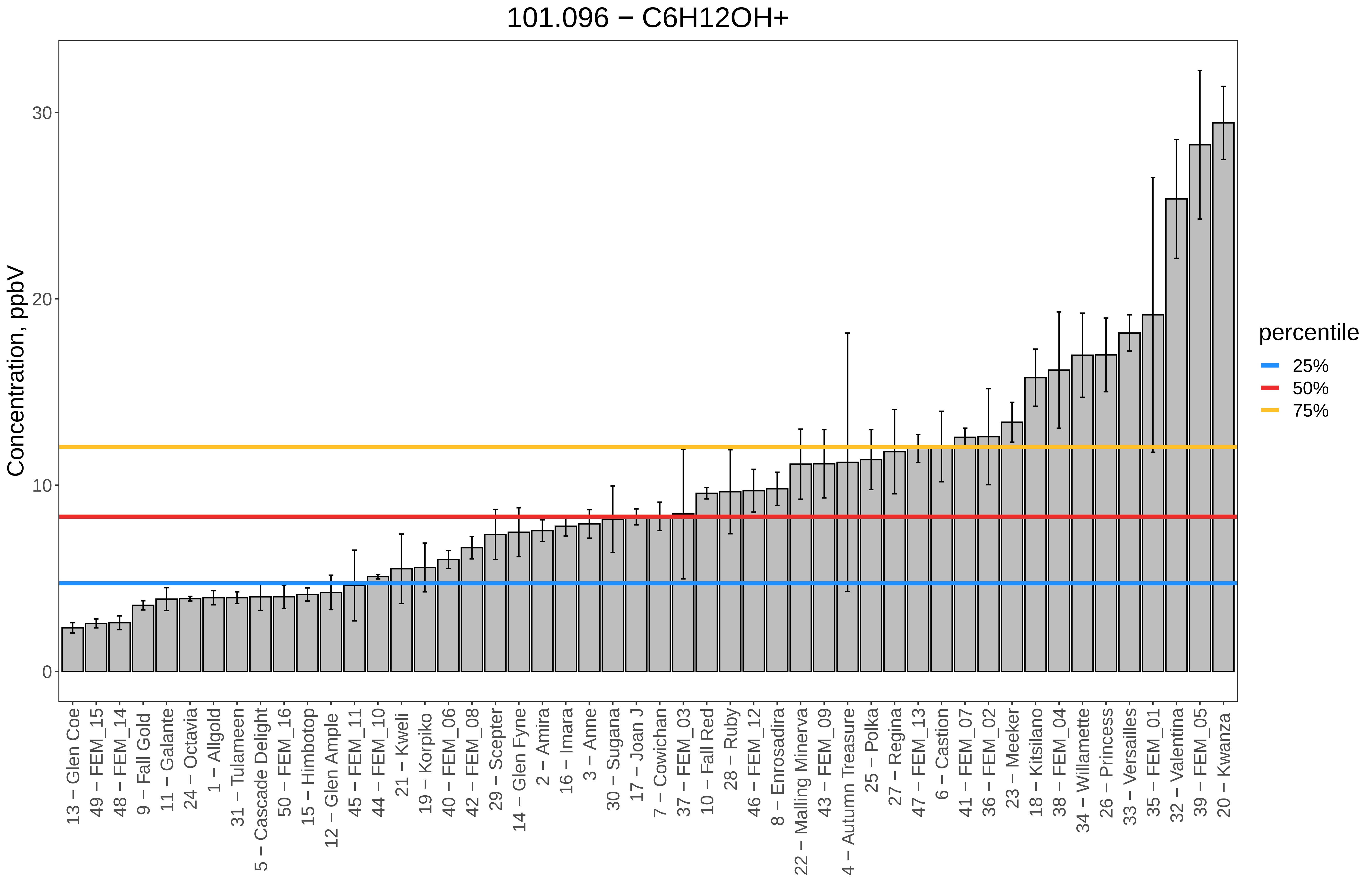

101.096 − C6H12OH+
Concentration, ppbV
0
10
20
30
percentile
25%
50%
75%
13 − Glen Coe
49 − FEM_15
48 − FEM_14
9 − Fall Gold
11 − Galante
24 − Octavia
1 − Allgold
31 − Tulameen
5 − Cascade Delight
50 − FEM_16
15 − Himbotop
12 − Glen Ample
45 − FEM_11
44 − FEM_10
21 − Kweli
19 − Korpiko
40 − FEM_06
42 − FEM_08
29 − Scepter
14 − Glen Fyne
2 − Amira
16 − Imara
3 − Anne
30 − Sugana
17 − Joan J
7 − Cowichan
37 − FEM_03
10 − Fall Red
28 − Ruby
46 − FEM_12
8 − Enrosadira
22 − Malling Minerva
43 − FEM_09
4 − Autumn Treasure
25 − Polka
27 − Regina
47 − FEM_13
6 − Castion
41 − FEM_07
36 − FEM_02
23 − Meeker
18 − Kitsilano
38 − FEM_04
34 − Willamette
26 − Princess
33 − Versailles
35 − FEM_01
32 − Valentina
39 − FEM_05
20 − Kwanza

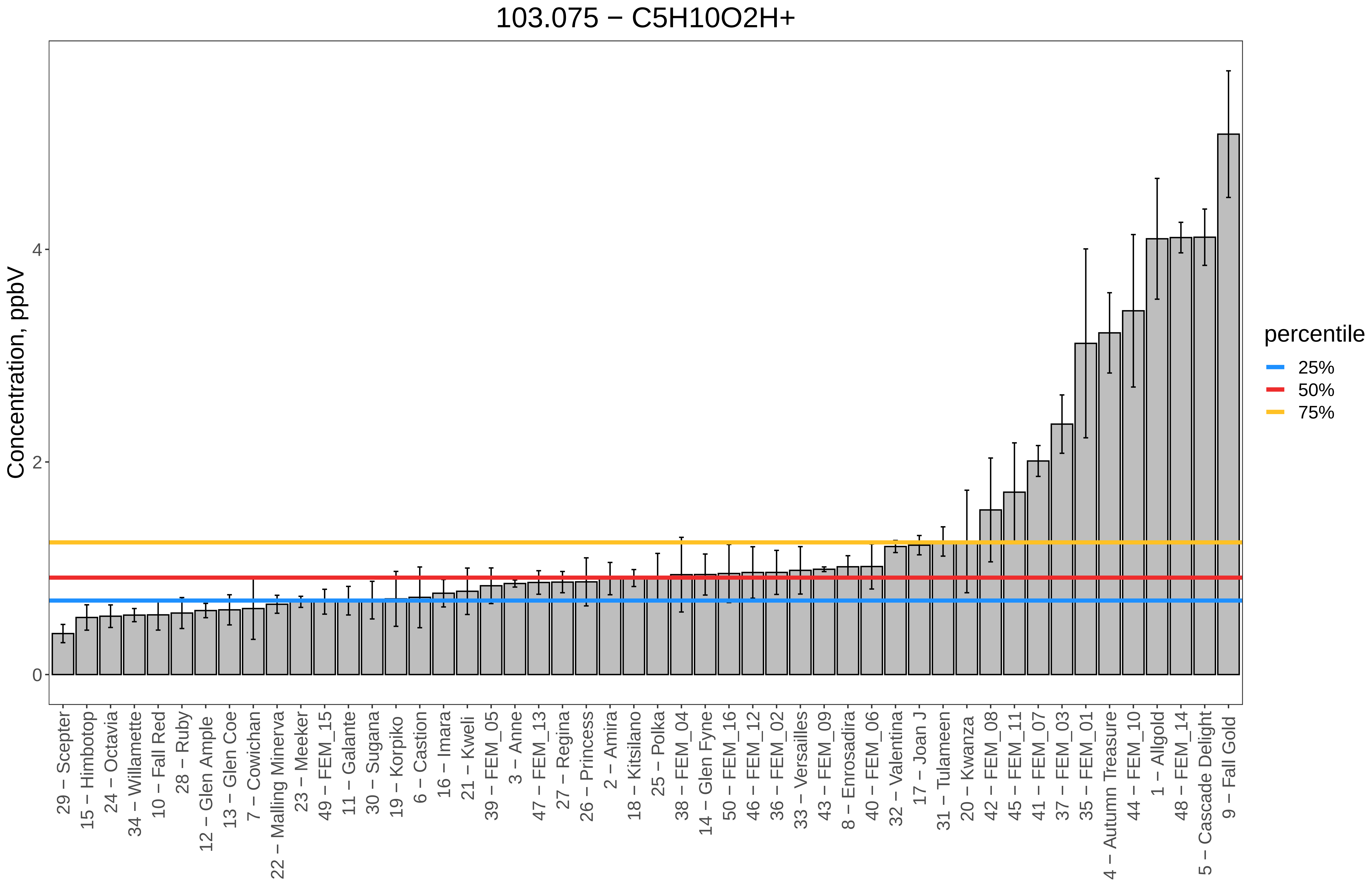

103.075 − C5H10O2H+
Concentration, ppbV
0
2
4
percentile
25%
50%
75%
29 − Scepter
15 − Himbotop
24 − Octavia
34 − Willamette
10 − Fall Red
28 − Ruby
12 − Glen Ample
13 − Glen Coe
7 − Cowichan
22 − Malling Minerva
23 − Meeker
49 − FEM_15
11 − Galante
30 − Sugana
19 − Korpiko
6 − Castion
16 − Imara
21 − Kweli
39 − FEM_05
3 − Anne
47 − FEM_13
27 − Regina
26 − Princess
2 − Amira
18 − Kitsilano
25 − Polka
38 − FEM_04
14 − Glen Fyne
50 − FEM_16
46 − FEM_12
36 − FEM_02
33 − Versailles
43 − FEM_09
8 − Enrosadira
40 − FEM_06
32 − Valentina
17 − Joan J
31 − Tulameen
20 − Kwanza
42 − FEM_08
45 − FEM_11
41 − FEM_07
37 − FEM_03
35 − FEM_01
4 − Autumn Treasure
44 − FEM_10
1 − Allgold
48 − FEM_14
5 − Cascade Delight
9 − Fall Gold

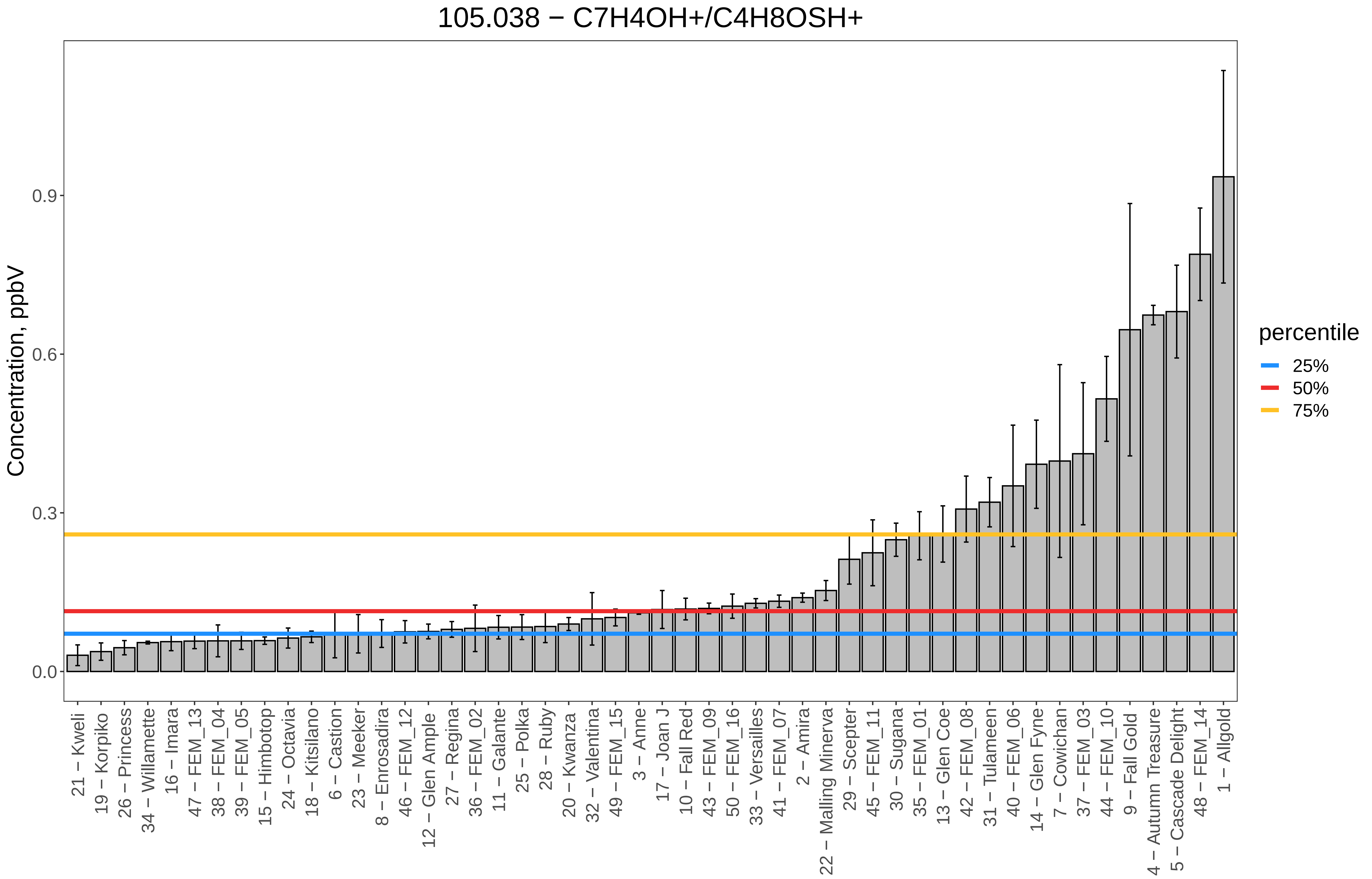

105.038 − C7H4OH+/C4H8OSH+
Concentration, ppbV
0.0
0.3
0.6
0.9
percentile
25%
50%
75%
21 − Kweli
19 − Korpiko
26 − Princess
34 − Willamette
16 − Imara
47 − FEM_13
38 − FEM_04
39 − FEM_05
15 − Himbotop
24 − Octavia
18 − Kitsilano
6 − Castion
23 − Meeker
8 − Enrosadira
46 − FEM_12
12 − Glen Ample
27 − Regina
36 − FEM_02
11 − Galante
25 − Polka
28 − Ruby
20 − Kwanza
32 − Valentina
49 − FEM_15
3 − Anne
17 − Joan J
10 − Fall Red
43 − FEM_09
50 − FEM_16
33 − Versailles
41 − FEM_07
2 − Amira
22 − Malling Minerva
29 − Scepter
45 − FEM_11
30 − Sugana
35 − FEM_01
13 − Glen Coe
42 − FEM_08
31 − Tulameen
40 − FEM_06
14 − Glen Fyne
7 − Cowichan
37 − FEM_03
44 − FEM_10
9 − Fall Gold
4 − Autumn Treasure
5 − Cascade Delight
48 − FEM_14
1 − Allgold

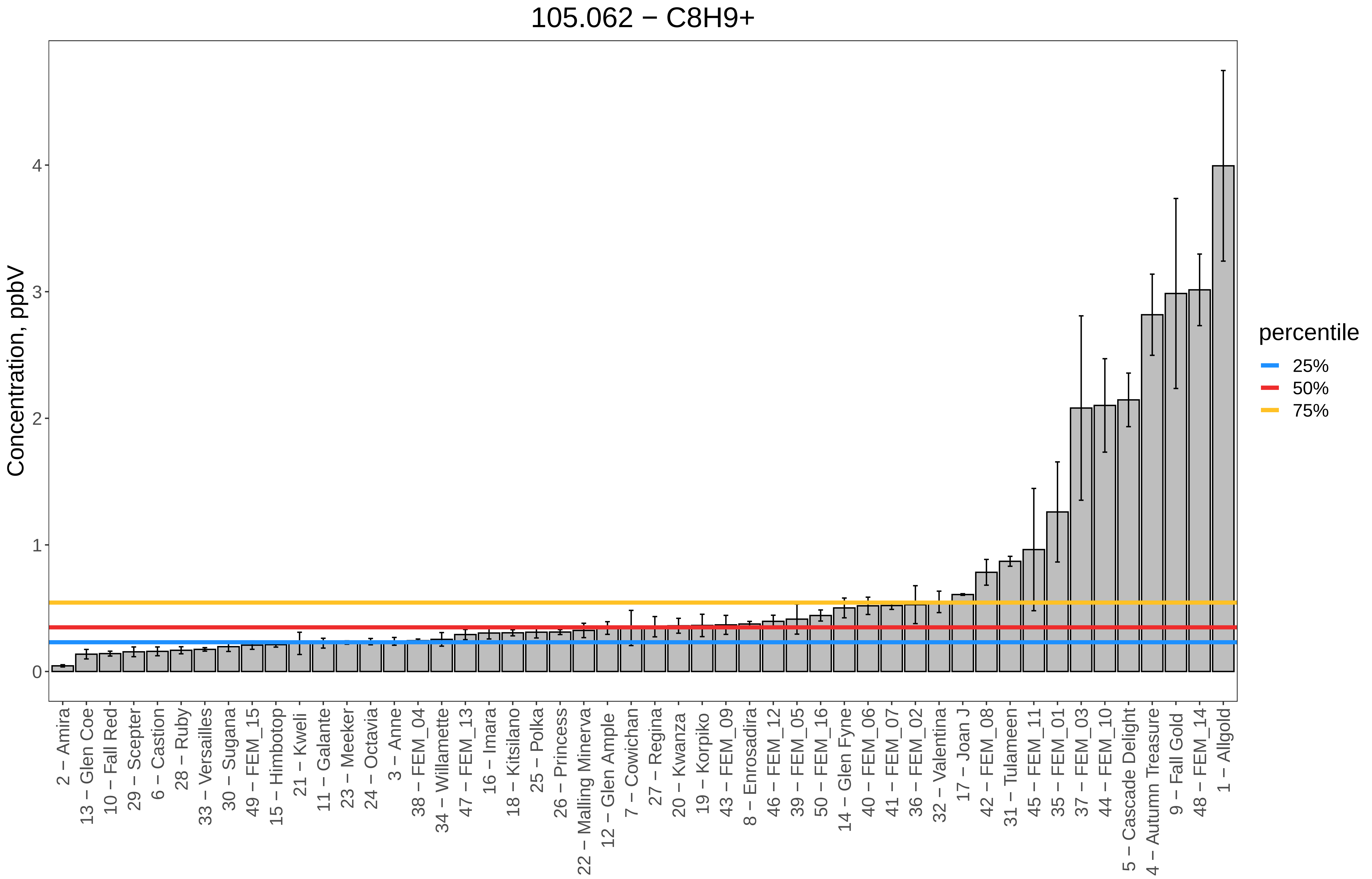

105.062 − C8H9+
Concentration, ppbV
0
1
2
3
4
percentile
25%
50%
75%
2 − Amira
13 − Glen Coe
10 − Fall Red
29 − Scepter
6 − Castion
28 − Ruby
33 − Versailles
30 − Sugana
49 − FEM_15
15 − Himbotop
21 − Kweli
11 − Galante
23 − Meeker
24 − Octavia
3 − Anne
38 − FEM_04
34 − Willamette
47 − FEM_13
16 − Imara
18 − Kitsilano
25 − Polka
26 − Princess
22 − Malling Minerva
12 − Glen Ample
7 − Cowichan
27 − Regina
20 − Kwanza
19 − Korpiko
43 − FEM_09
8 − Enrosadira
46 − FEM_12
39 − FEM_05
50 − FEM_16
14 − Glen Fyne
40 − FEM_06
41 − FEM_07
36 − FEM_02
32 − Valentina
17 − Joan J
42 − FEM_08
31 − Tulameen
45 − FEM_11
35 − FEM_01
37 − FEM_03
44 − FEM_10
5 − Cascade Delight
4 − Autumn Treasure
9 − Fall Gold
48 − FEM_14
1 − Allgold

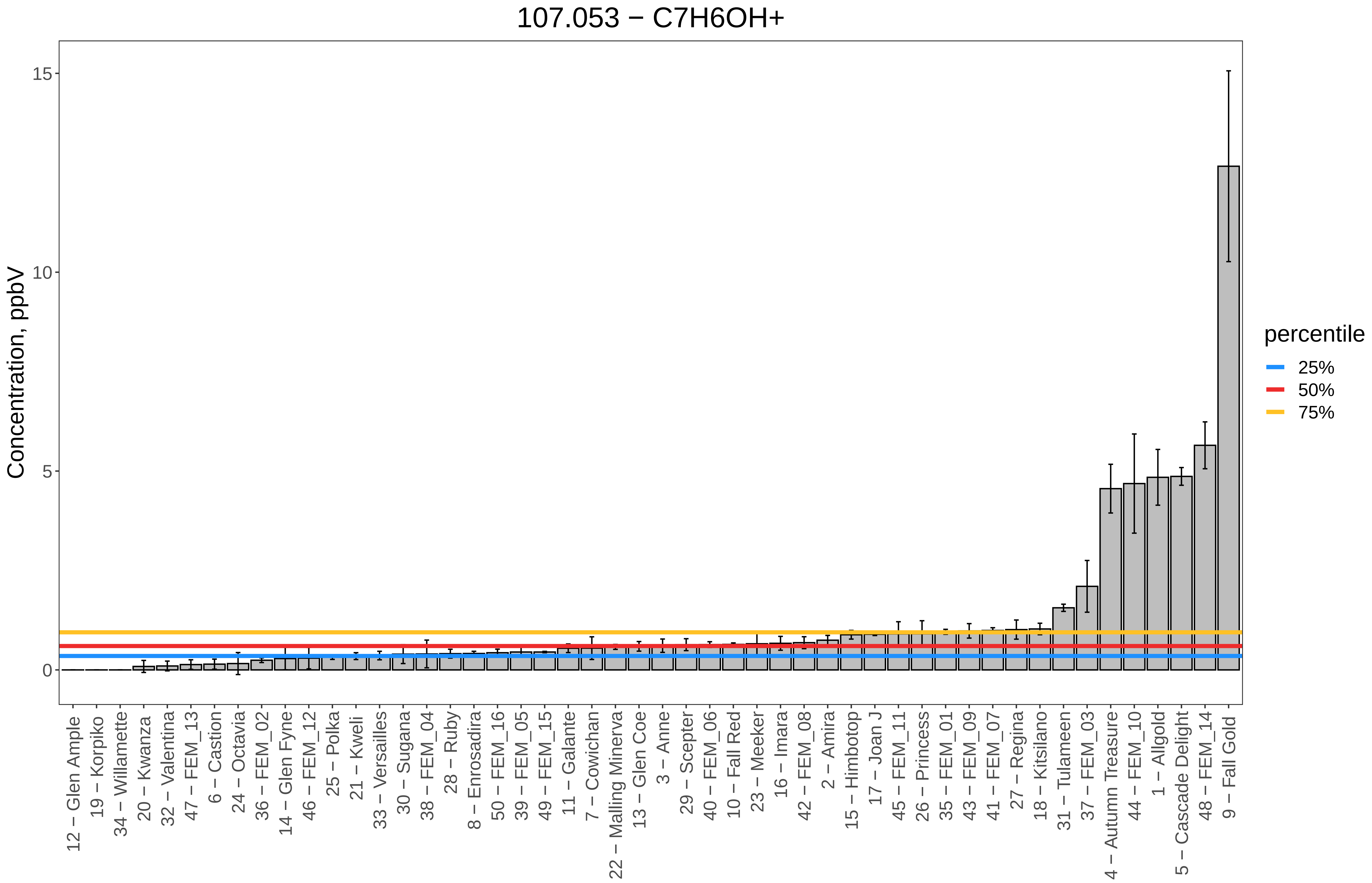

107.053 − C7H6OH+
Concentration, ppbV
0
5
10
15
percentile
25%
50%
75%
12 − Glen Ample
19 − Korpiko
34 − Willamette
20 − Kwanza
32 − Valentina
47 − FEM_13
6 − Castion
24 − Octavia
36 − FEM_02
14 − Glen Fyne
46 − FEM_12
25 − Polka
21 − Kweli
33 − Versailles
30 − Sugana
38 − FEM_04
28 − Ruby
8 − Enrosadira
50 − FEM_16
39 − FEM_05
49 − FEM_15
11 − Galante
7 − Cowichan
22 − Malling Minerva
13 − Glen Coe
3 − Anne
29 − Scepter
40 − FEM_06
10 − Fall Red
23 − Meeker
16 − Imara
42 − FEM_08
2 − Amira
15 − Himbotop
17 − Joan J
45 − FEM_11
26 − Princess
35 − FEM_01
43 − FEM_09
41 − FEM_07
27 − Regina
18 − Kitsilano
31 − Tulameen
37 − FEM_03
4 − Autumn Treasure
44 − FEM_10
1 − Allgold
5 − Cascade Delight
48 − FEM_14
9 − Fall Gold

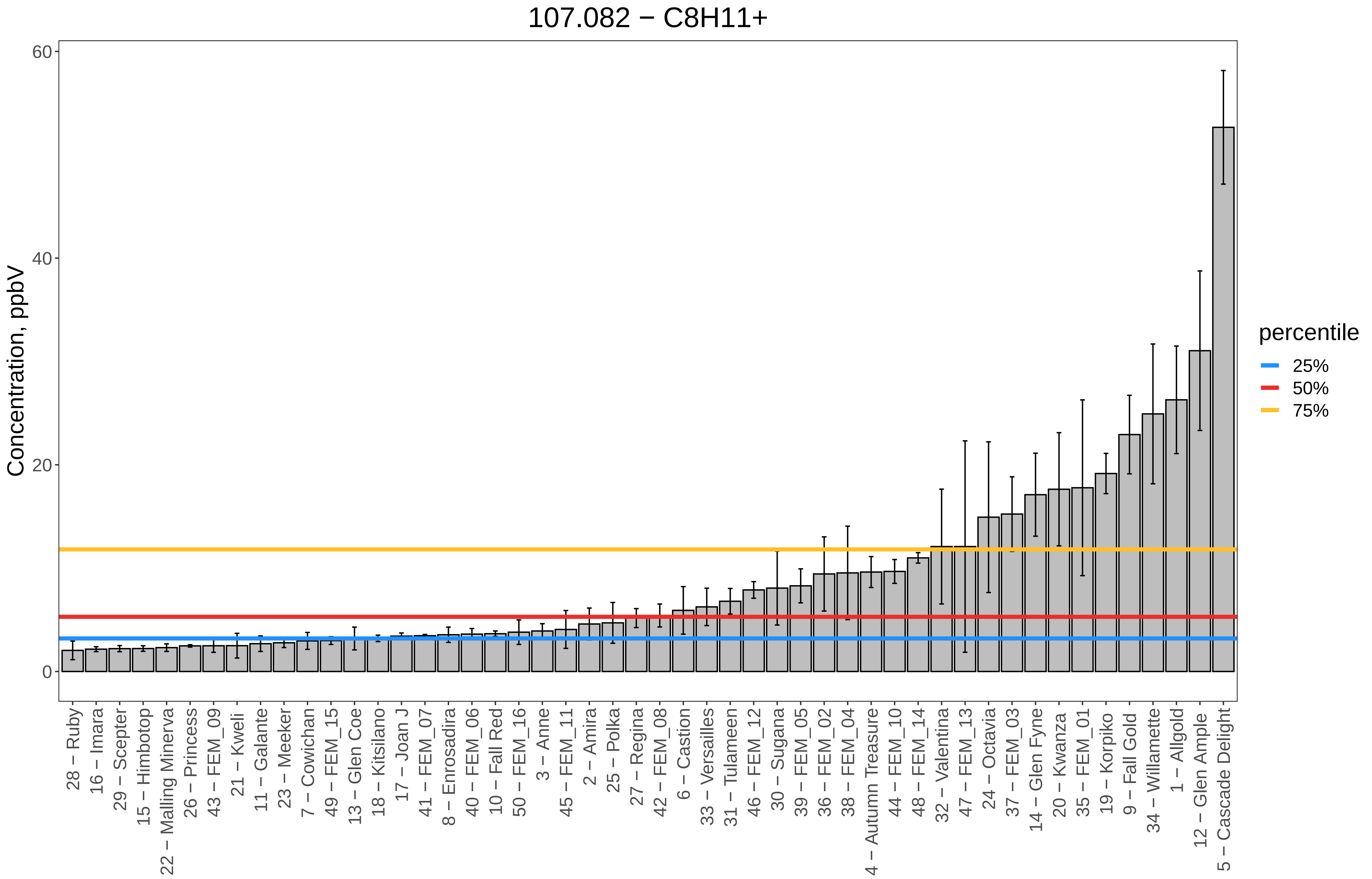

107.082 − C8H11+
Concentration, ppbV
0
20
40
60
percentile
25%
50%
75%
28 − Ruby
16 − Imara
29 − Scepter
15 − Himbotop
22 − Malling Minerva
26 − Princess
43 − FEM_09
21 − Kweli
11 − Galante
23 − Meeker
7 − Cowichan
49 − FEM_15
13 − Glen Coe
18 − Kitsilano
17 − Joan J
41 − FEM_07
8 − Enrosadira
40 − FEM_06
10 − Fall Red
50 − FEM_16
3 − Anne
45 − FEM_11
2 − Amira
25 − Polka
27 − Regina
42 − FEM_08
6 − Castion
33 − Versailles
31 − Tulameen
46 − FEM_12
30 − Sugana
39 − FEM_05
36 − FEM_02
38 − FEM_04
4 − Autumn Treasure
44 − FEM_10
48 − FEM_14
32 − Valentina
47 − FEM_13
24 − Octavia
37 − FEM_03
14 − Glen Fyne
20 − Kwanza
35 − FEM_01
19 − Korpiko
9 − Fall Gold
34 − Willamette
1 − Allgold
12 − Glen Ample
5 − Cascade Delight

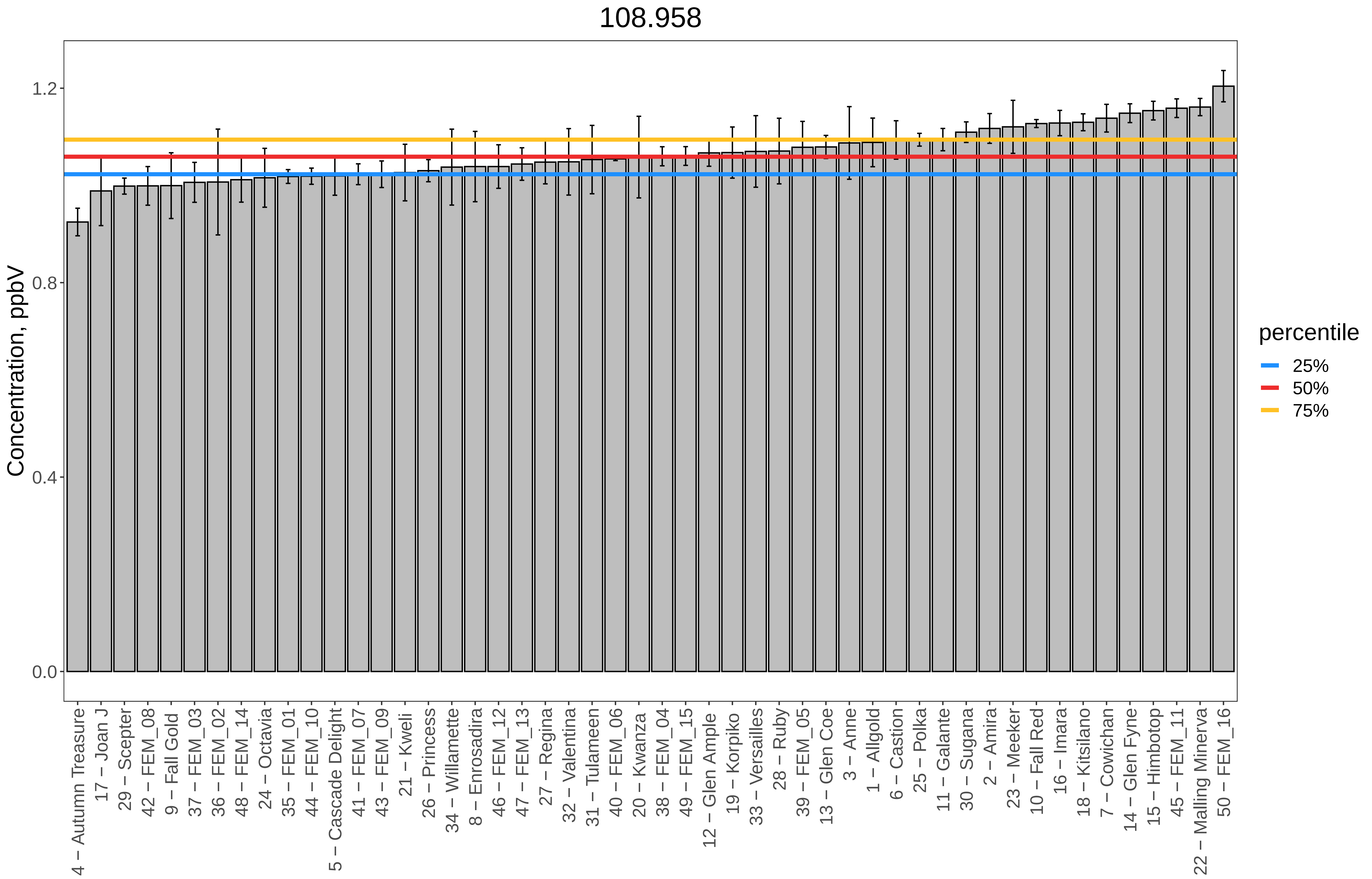

108.958
Concentration, ppbV
1.2
0.8
0.4
0.0
percentile
25%
50%
75%
4 – Autumn Treasure
17 – Joan J
29 – Scepter
42 – FEM_08
9 – Fall Gold
37 – FEM_03
36 – FEM_02
48 – FEM_14
24 – Octavia
35 – FEM_01
44 – FEM_10
5 – Cascade Delight
41 – FEM_07
43 – FEM_09
21 – Kweli
26 – Princess
34 – Willamette
8 – Enrosadira
46 – FEM_12
47 – FEM_13
27 – Regina
32 – Valentina
31 – Tulameen
40 – FEM_06
20 – Kwanza
38 – FEM_04
49 – FEM_15
12 – Glen Ample
19 – Korpiko
33 – Versailles
28 – Ruby
39 – FEM_05
13 – Glen Coe
3 – Anne
1 – Allgold
6 – Castion
25 – Polka
11 – Galante
30 – Sugana
2 – Amira
23 – Meeker
10 – Fall Red
16 – Imara
18 – Kitsilano
7 – Cowichan
14 – Glen Fyne
15 – Himbotop
45 – FEM_11
22 – Malling Minerva
50 – FEM_16

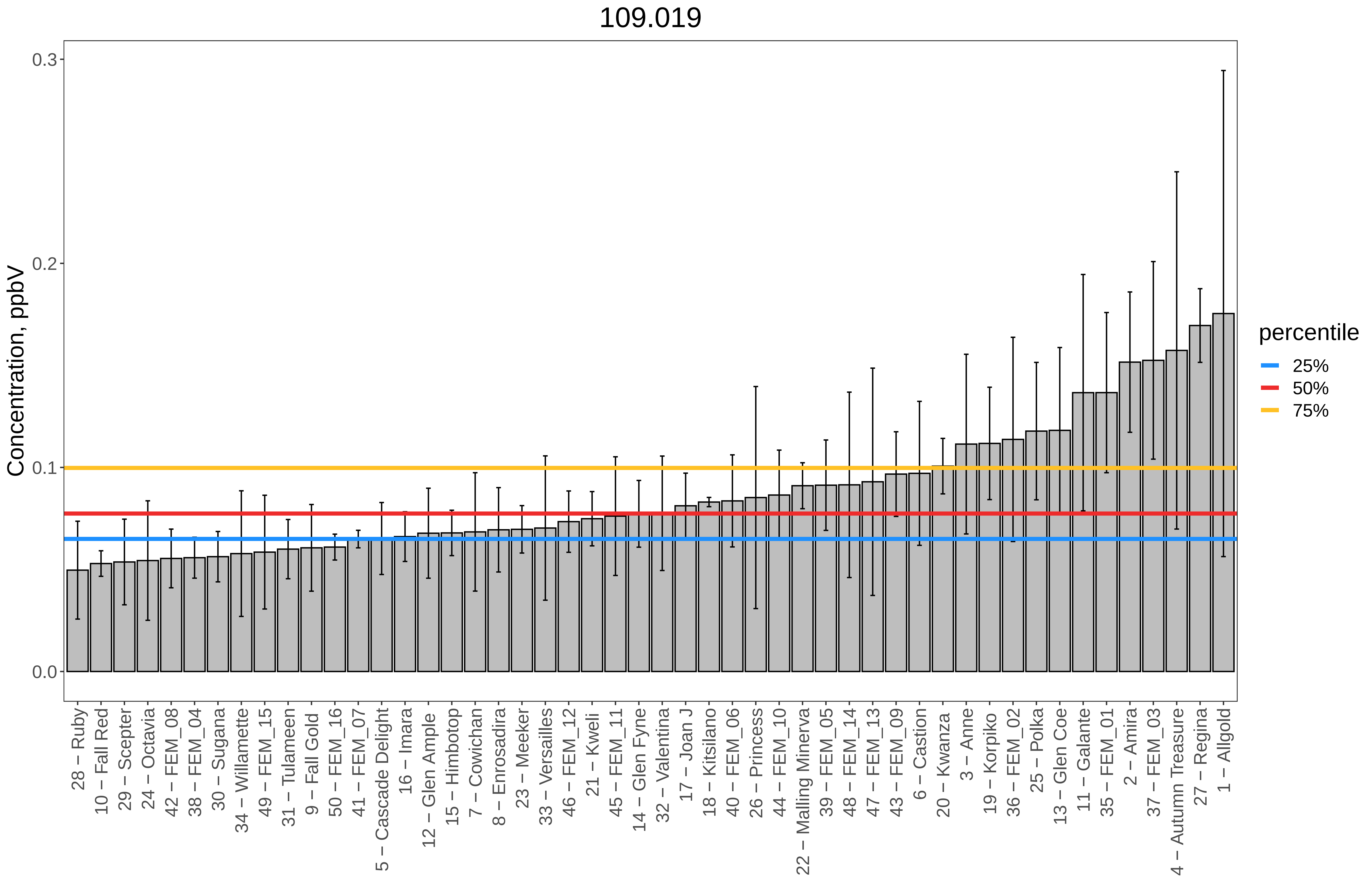

109.019
Concentration, ppbV
0.0
0.1
0.2
0.3
percentile
25%
50%
75%
28 – Ruby
10 – Fall Red
29 – Scepter
24 – Octavia
42 – FEM_08
38 – FEM_04
30 – Sugana
34 – Willamette
49 – FEM_15
31 – Tulameen
9 – Fall Gold
50 – FEM_16
41 – FEM_07
5 – Cascade Delight
16 – Imara
12 – Glen Ample
15 – Himbotop
7 – Cowichan
8 – Enrosadira
23 – Meeker
33 – Versailles
46 – FEM_12
21 – Kweli
45 – FEM_11
14 – Glen Fyne
32 – Valentina
17 – Joan J
18 – Kitsilano
40 – FEM_06
26 – Princess
44 – FEM_10
22 – Malling Minerva
39 – FEM_05
48 – FEM_14
47 – FEM_13
43 – FEM_09
6 – Castion
20 – Kwanza
3 – Anne
19 – Korpiko
36 – FEM_02
25 – Polka
13 – Glen Coe
11 – Galante
35 – FEM_01
2 – Amira
37 – FEM_03
4 – Autumn Treasure
27 – Regina
1 – Allgold

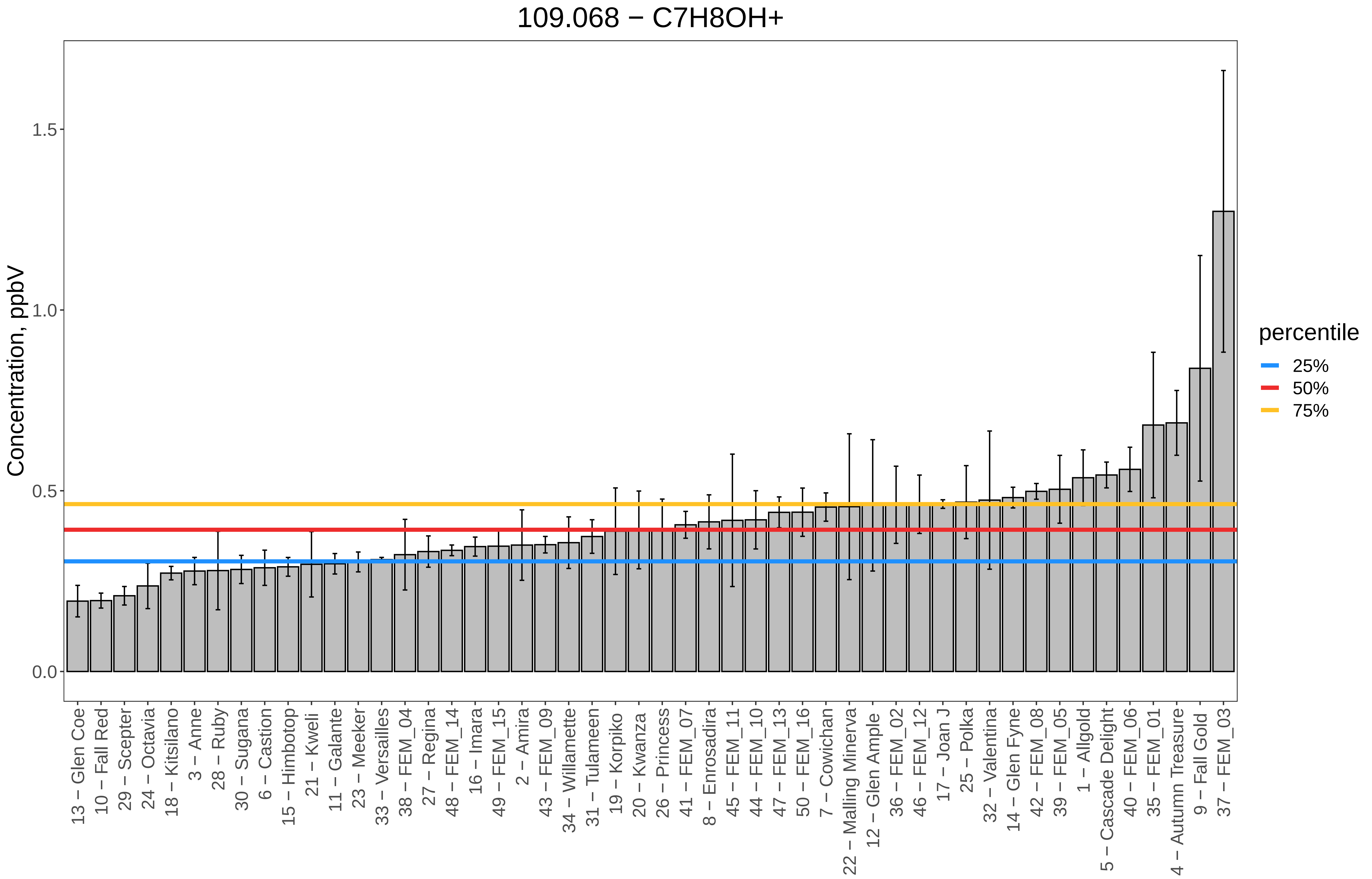

109.068 − C7H8OH+
Concentration, ppbV
0.0
0.5
1.0
1.5
percentile
25%
50%
75%
13 − Glen Coe
10 − Fall Red
29 − Scepter
24 − Octavia
18 − Kitsilano
3 − Anne
28 − Ruby
30 − Sugana
6 − Castion
15 − Himbotop
21 − Kweli
11 − Galante
23 − Meeker
33 − Versailles
38 − FEM_04
27 − Regina
48 − FEM_14
16 − Imara
49 − FEM_15
2 − Amira
43 − FEM_09
34 − Willamette
31 − Tulameen
19 − Korpiko
20 − Kwanza
26 − Princess
41 − FEM_07
8 − Enrosadira
45 − FEM_11
44 − FEM_10
47 − FEM_13
50 − FEM_16
7 − Cowichan
22 − Malling Minerva
12 − Glen Ample
36 − FEM_02
46 − FEM_12
17 − Joan J
25 − Polka
32 − Valentina
14 − Glen Fyne
42 − FEM_08
39 − FEM_05
1 − Allgold
5 − Cascade Delight
40 − FEM_06
35 − FEM_01
4 − Autumn Treasure
9 − Fall Gold
37 − FEM_03

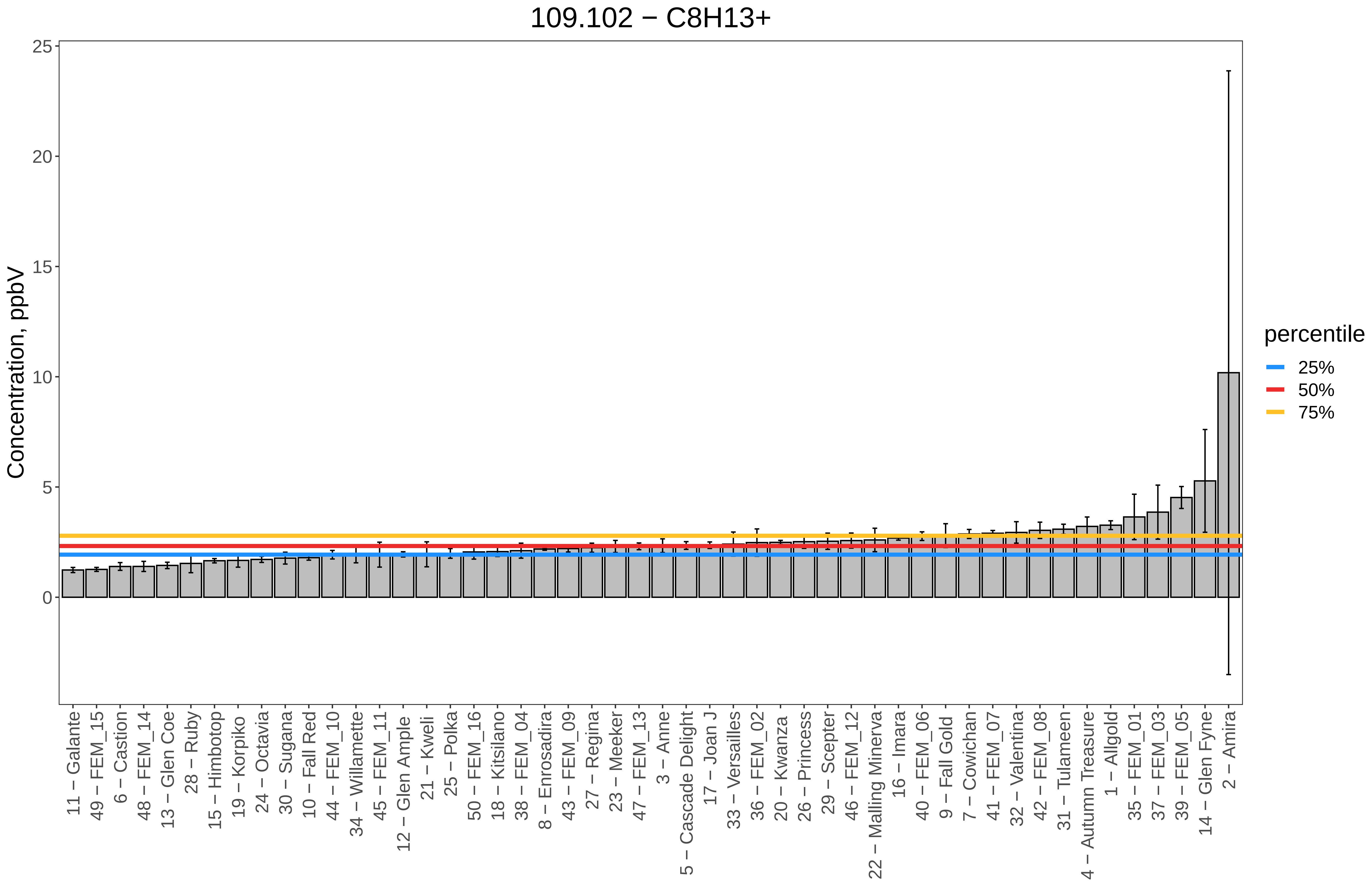

109.102 − C8H13+
Concentration, ppbV
0
5
10
15
20
25
percentile
25%
50%
75%
11 − Galante
49 − FEM_15
6 − Castion
48 − FEM_14
13 − Glen Coe
28 − Ruby
15 − Himbotop
19 − Korpiko
24 − Octavia
30 − Sugana
10 − Fall Red
44 − FEM_10
34 − Willamette
45 − FEM_11
12 − Glen Ample
21 − Kweli
25 − Polka
50 − FEM_16
18 − Kitsilano
38 − FEM_04
8 − Enrosadira
43 − FEM_09
27 − Regina
23 − Meeker
47 − FEM_13
3 − Anne
5 − Cascade Delight
17 − Joan J
33 − Versailles
36 − FEM_02
20 − Kwanza
26 − Princess
29 − Scepter
46 − FEM_12
22 − Malling Minerva
16 − Imara
40 − FEM_06
9 − Fall Gold
7 − Cowichan
41 − FEM_07
32 − Valentina
42 − FEM_08
31 − Tulameen
4 − Autumn Treasure
1 − Allgold
35 − FEM_01
37 − FEM_03
39 − FEM_05
14 − Glen Fyne
2 − Amira

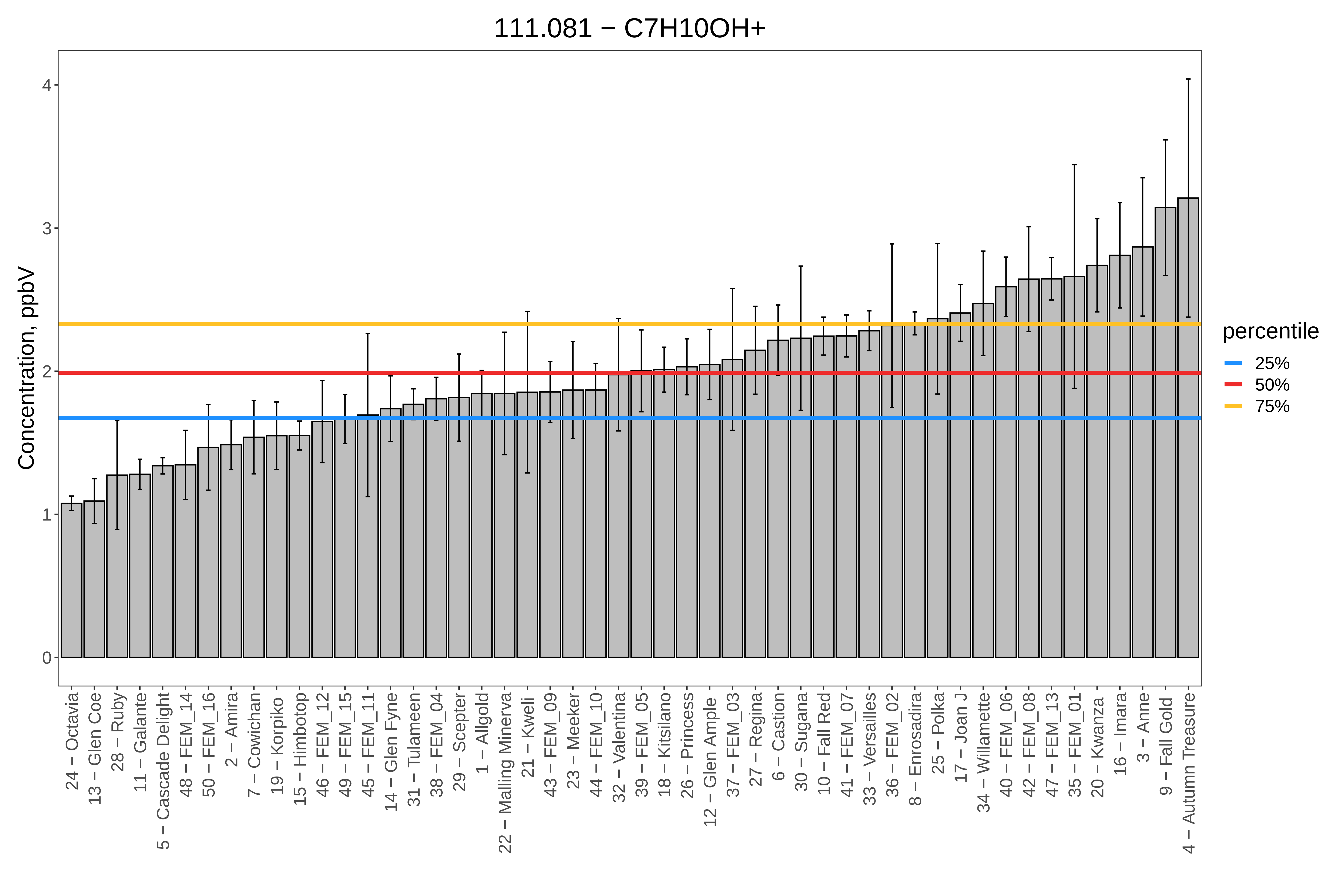

111.081 − C7H10OH+
Concentration, ppbV
0
1
2
3
4
percentile
25%
50%
75%
24 − Octavia
13 − Glen Coe
28 − Ruby
11 − Galante
5 − Cascade Delight
48 − FEM_14
50 − FEM_16
2 − Amira
7 − Cowichan
19 − Korpiko
15 − Himbotop
46 − FEM_12
49 − FEM_15
45 − FEM_11
14 − Glen Fyne
31 − Tulameen
38 − FEM_04
29 − Scepter
1 − Allgold
22 − Malling Minerva
21 − Kweli
43 − FEM_09
23 − Meeker
44 − FEM_10
32 − Valentina
39 − FEM_05
18 − Kitsilano
26 − Princess
12 − Glen Ample
37 − FEM_03
27 − Regina
6 − Castion
30 − Sugana
10 − Fall Red
41 − FEM_07
33 − Versailles
36 − FEM_02
8 − Enrosadira
25 − Polka
17 − Joan J
34 − Willamette
40 − FEM_06
42 − FEM_08
47 − FEM_13
35 − FEM_01
20 − Kwanza
16 − Imara
3 − Anne
9 − Fall Gold
4 − Autumn Treasure

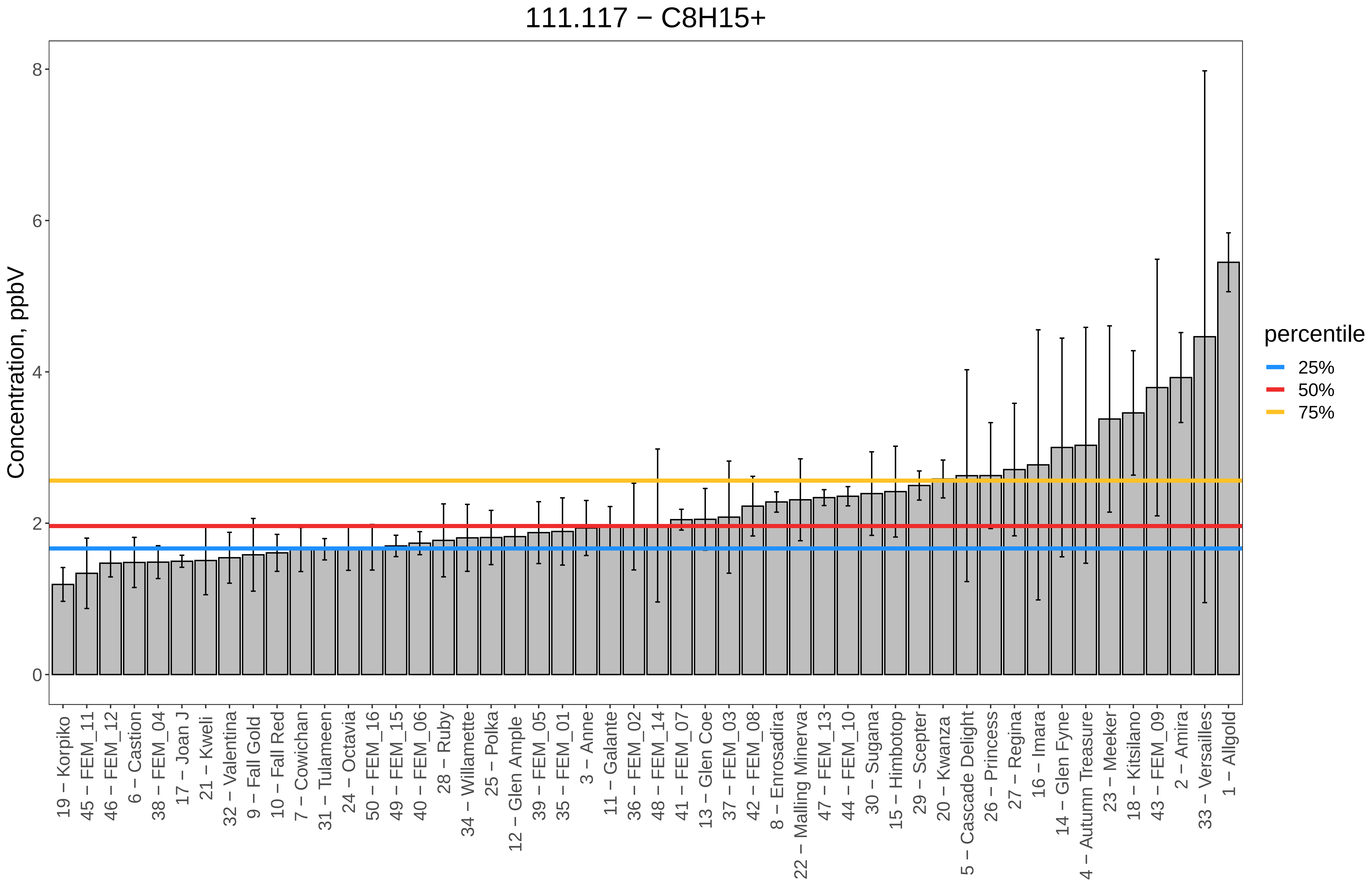

111.117 − C8H15+
Concentration, ppbV
0
2
4
6
8
percentile
25%
50%
75%
19 − Korpiko
45 − FEM_11
46 − FEM_12
6 − Castion
38 − FEM_04
17 − Joan J
21 − Kweli
32 − Valentina
9 − Fall Gold
10 − Fall Red
7 − Cowichan
31 − Tulameen
24 − Octavia
50 − FEM_16
49 − FEM_15
40 − FEM_06
28 − Ruby
34 − Willamette
25 − Polka
12 − Glen Ample
39 − FEM_05
35 − FEM_01
3 − Anne
11 − Galante
36 − FEM_02
48 − FEM_14
41 − FEM_07
13 − Glen Coe
37 − FEM_03
42 − FEM_08
8 − Enrosadira
22 − Malling Minerva
47 − FEM_13
44 − FEM_10
30 − Sugana
15 − Himbotop
29 − Scepter
20 − Kwanza
5 − Cascade Delight
26 − Princess
27 − Regina
16 − Imara
14 − Glen Fyne
4 − Autumn Treasure
23 − Meeker
18 − Kitsilano
43 − FEM_09
2 − Amira
33 − Versailles
1 − Allgold

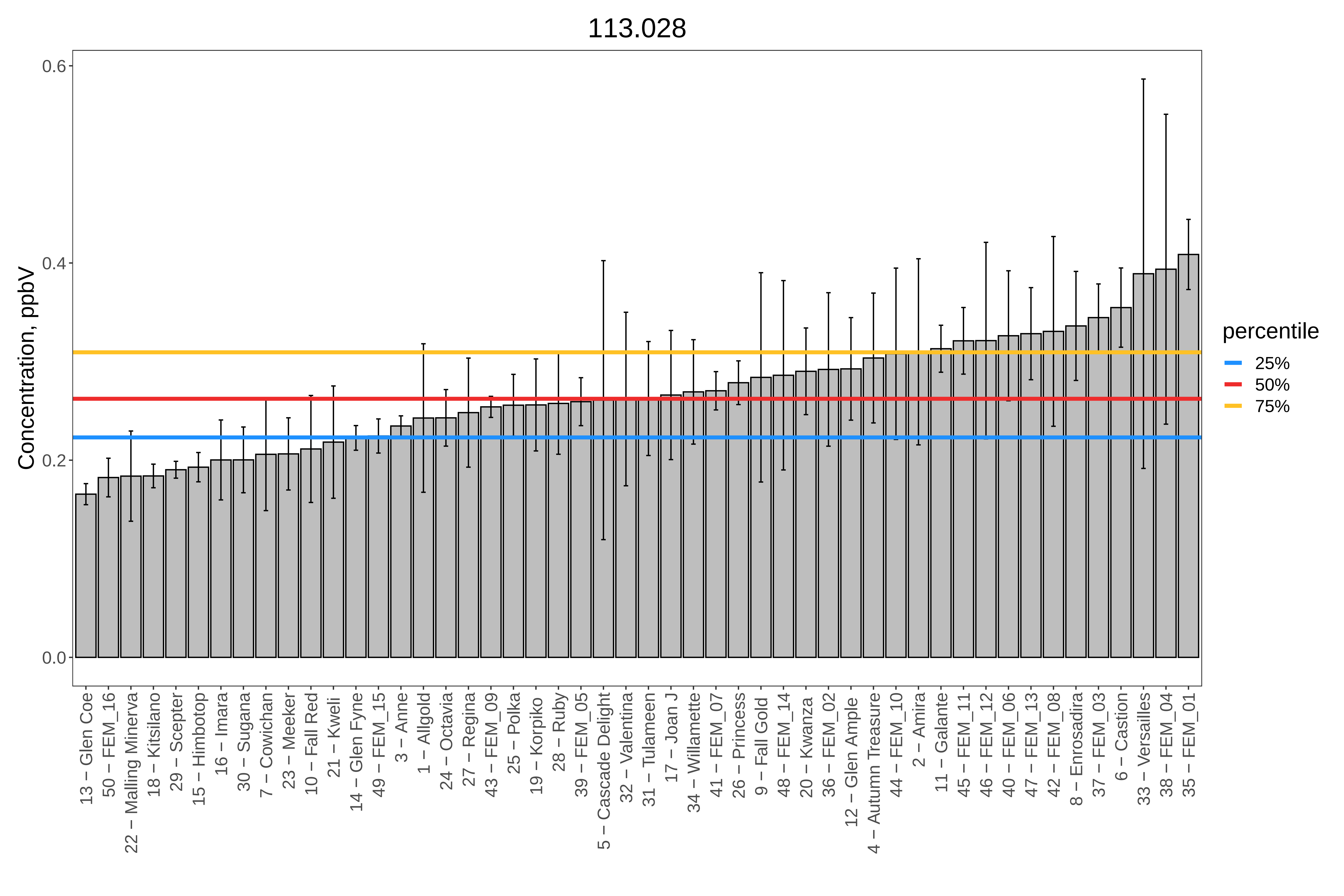

113.028
Concentration, ppbV
0.0
0.2
0.4
0.6
percentile
25%
50%
75%
13 – Glen Coe
50 – FEM_16
22 – Malling Minerva
18 – Kitsilano
29 – Scepter
15 – Himbotop
16 – Imara
30 – Sugana
7 – Cowichan
23 – Meeker
10 – Fall Red
21 – Kweli
14 – Glen Fyne
49 – FEM_15
3 – Anne
1 – Allgold
24 – Octavia
27 – Regina
43 – FEM_09
25 – Polka
19 – Korpiko
28 – Ruby
39 – FEM_05
5 – Cascade Delight
32 – Valentina
31 – Tulameen
17 – Joan J
34 – Willamette
41 – FEM_07
26 – Princess
9 – Fall Gold
48 – FEM_14
20 – Kwanza
36 – FEM_02
12 – Glen Ample
4 – Autumn Treasure
44 – FEM_10
2 – Amira
11 – Galante
45 – FEM_11
46 – FEM_12
40 – FEM_06
47 – FEM_13
42 – FEM_08
8 – Enrosadira
37 – FEM_03
6 – Castion
33 – Versailles
38 – FEM_04
35 – FEM_01

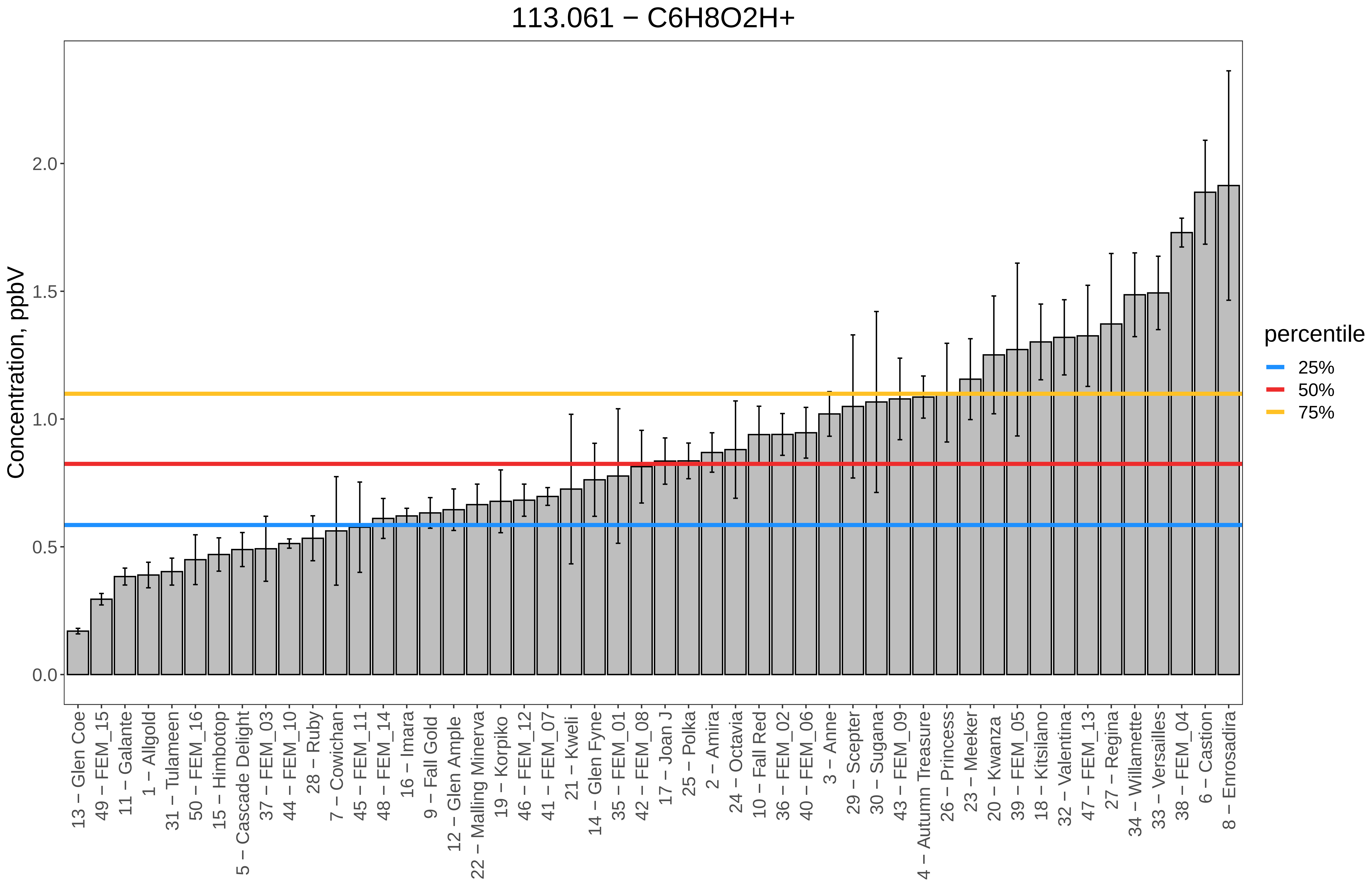
113.061 − C6H8O2H+
Concentration, ppbV
0.0
0.5
1.0
1.5
2.0
percentile
25%
50%
75%
13 − Glen Coe
49 − FEM_15
11 − Galante
1 − Allgold
31 − Tulameen
50 − FEM_16
15 − Himbotop
5 − Cascade Delight
37 − FEM_03
44 − FEM_10
28 − Ruby
7 − Cowichan
45 − FEM_11
48 − FEM_14
16 − Imara
9 − Fall Gold
12 − Glen Ample
22 − Malling Minerva
19 − Korpiko
46 − FEM_12
41 − FEM_07
21 − Kweli
14 − Glen Fyne
35 − FEM_01
42 − FEM_08
17 − Joan J
25 − Polka
2 − Amira
24 − Octavia
10 − Fall Red
36 − FEM_02
40 − FEM_06
3 − Anne
29 − Scepter
30 − Sugana
43 − FEM_09
4 − Autumn Treasure
26 − Princess
23 − Meeker
20 − Kwanza
39 − FEM_05
18 − Kitsilano
32 − Valentina
47 − FEM_13
27 − Regina
34 − Willamette
33 − Versailles
38 − FEM_04
6 − Castion
8 − Enrosadira

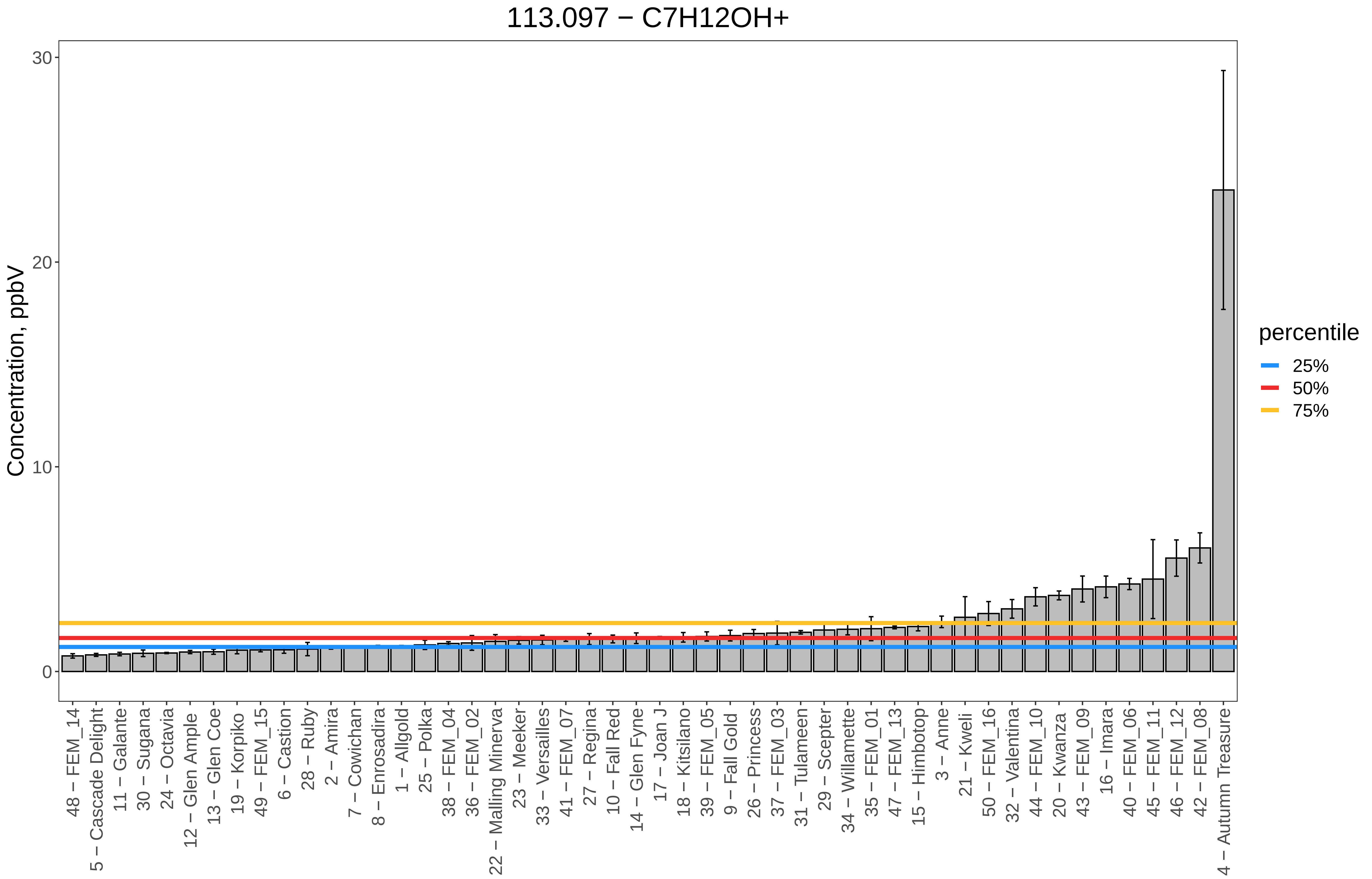

113.097 − C7H12OH+
Concentration, ppbV
0
10
20
30
percentile
25%
50%
75%
48 − FEM_14
5 − Cascade Delight
11 − Galante
30 − Sugana
24 − Octavia
12 − Glen Ample
13 − Glen Coe
19 − Korpiko
49 − FEM_15
6 − Castion
28 − Ruby
2 − Amira
7 − Cowichan
8 − Enrosadira
1 − Allgold
25 − Polka
38 − FEM_04
36 − FEM_02
22 − Malling Minerva
23 − Meeker
33 − Versailles
41 − FEM_07
27 − Regina
10 − Fall Red
14 − Glen Fyne
17 − Joan J
18 − Kitsilano
39 − FEM_05
9 − Fall Gold
26 − Princess
37 − FEM_03
31 − Tulameen
29 − Scepter
34 − Willamette
35 − FEM_01
47 − FEM_13
15 − Himbotop
3 − Anne
21 − Kweli
50 − FEM_16
32 − Valentina
44 − FEM_10
20 − Kwanza
43 − FEM_09
16 − Imara
40 − FEM_06
45 − FEM_11
46 − FEM_12
42 − FEM_08
4 − Autumn Treasure

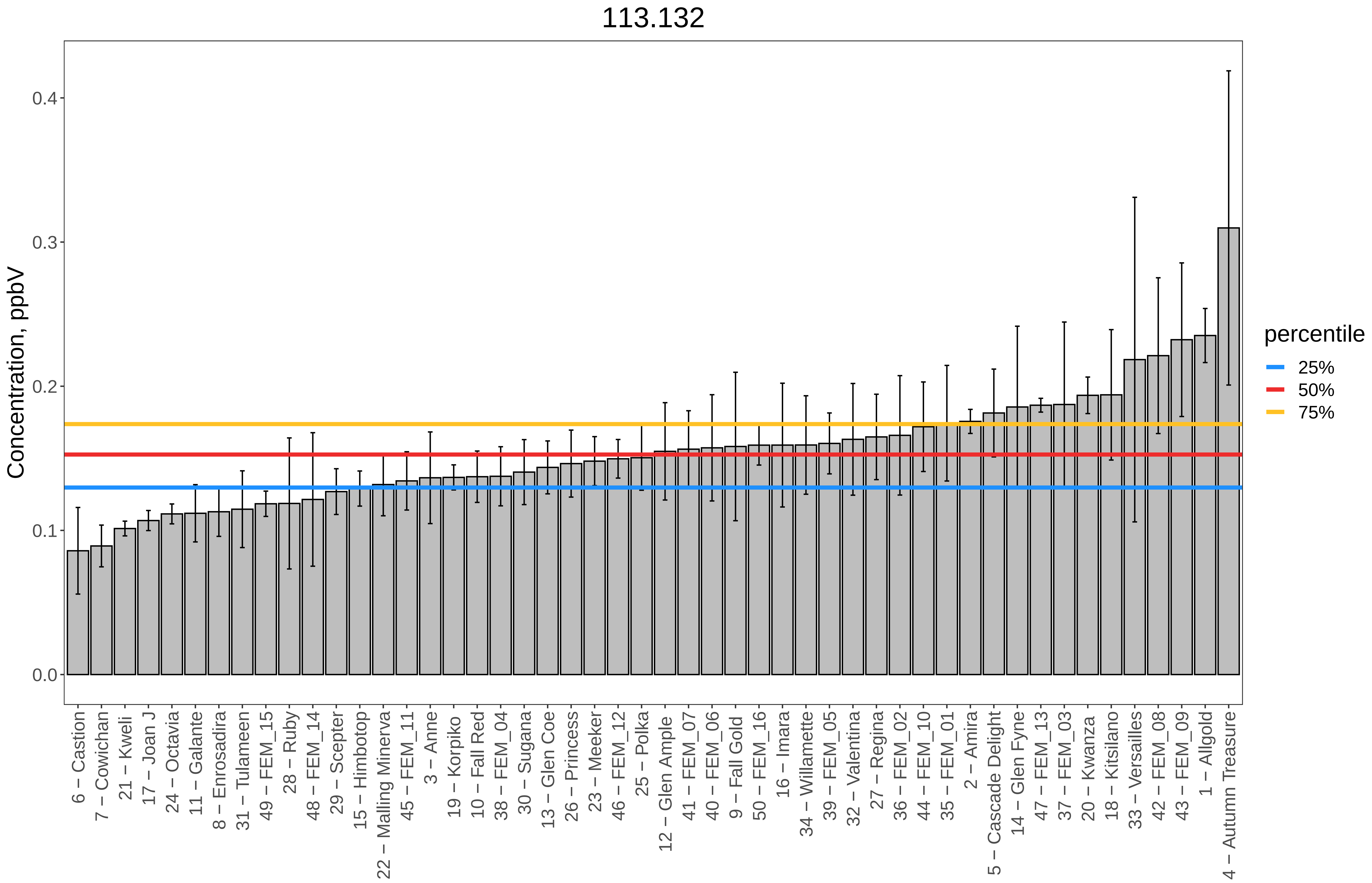

113.132
Concentration, ppbV
0.0
0.1
0.2
0.3
0.4
percentile
25%
50%
75%
6 – Castion
7 – Cowichan
21 – Kweli
17 – Joan J
24 – Octavia
11 – Galante
8 – Enrosadira
31 – Tulameen
49 – FEM_15
28 – Ruby
48 – FEM_14
29 – Scepter
15 – Himbotop
22 – Malling Minerva
45 – FEM_11
3 – Anne
19 – Korpiko
10 – Fall Red
38 – FEM_04
30 – Sugana
13 – Glen Coe
26 – Princess
23 – Meeker
46 – FEM_12
25 – Polka
12 – Glen Ample
41 – FEM_07
40 – FEM_06
9 – Fall Gold
50 – FEM_16
16 – Imara
34 – Willamette
39 – FEM_05
32 – Valentina
27 – Regina
36 – FEM_02
44 – FEM_10
35 – FEM_01
2 – Amira
5 – Cascade Delight
14 – Glen Fyne
47 – FEM_13
37 – FEM_03
20 – Kwanza
18 – Kitsilano
33 – Versailles
42 – FEM_08
43 – FEM_09
1 – Allgold
4 – Autumn Treasure

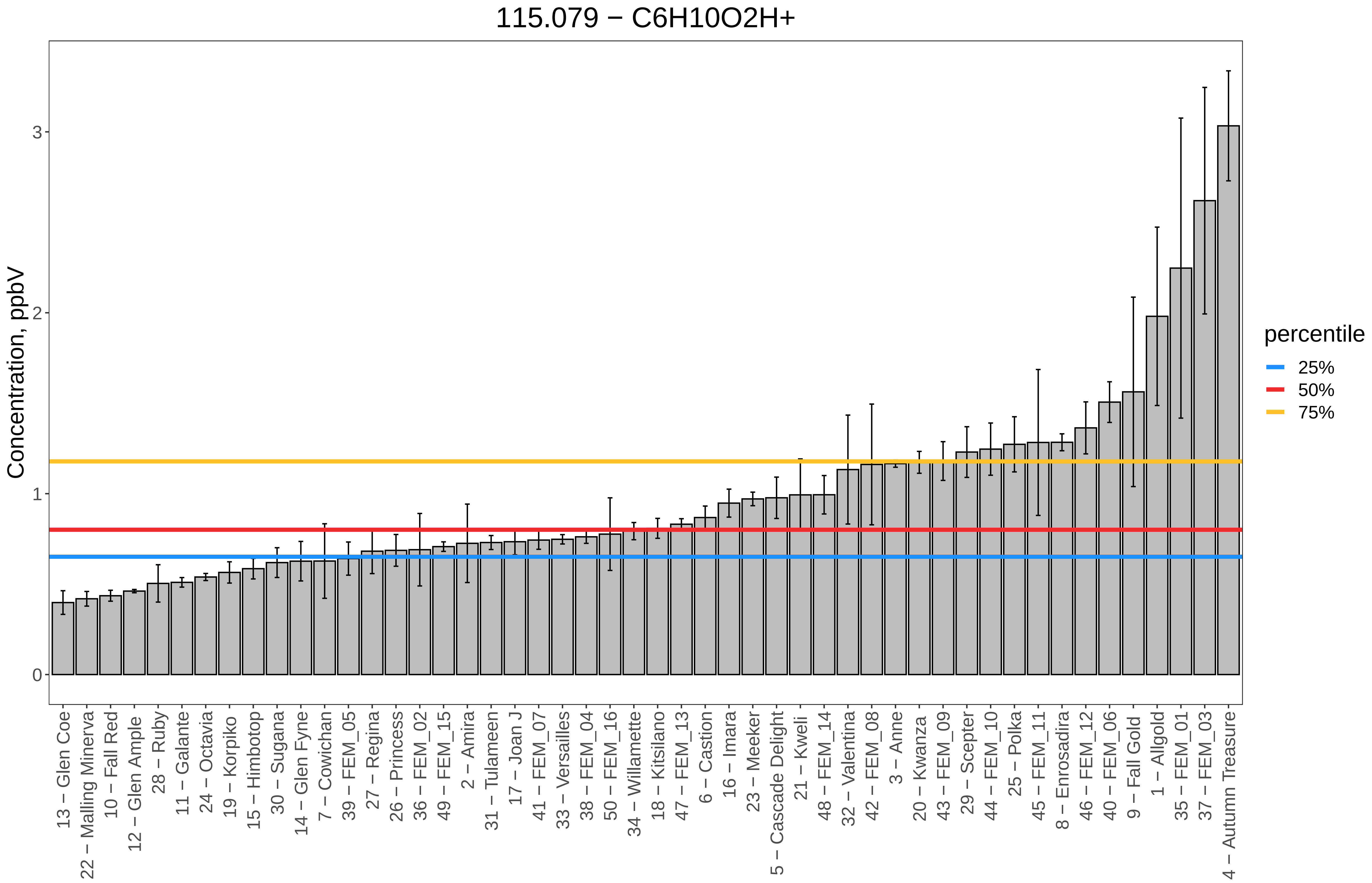

115.079 − C6H10O2H+
Concentration, ppbV
0
1
2
3
percentile
25%
50%
75%
13 − Glen Coe
22 − Malling Minerva
10 − Fall Red
12 − Glen Ample
28 − Ruby
11 − Galante
24 − Octavia
19 − Korpiko
15 − Himbotop
30 − Sugana
14 − Glen Fyne
7 − Cowichan
39 − FEM_05
27 − Regina
26 − Princess
36 − FEM_02
49 − FEM_15
2 − Amira
31 − Tulameen
17 − Joan J
41 − FEM_07
33 − Versailles
38 − FEM_04
50 − FEM_16
34 − Willamette
18 − Kitsilano
47 − FEM_13
6 − Castion
16 − Imara
23 − Meeker
5 − Cascade Delight
21 − Kweli
48 − FEM_14
32 − Valentina
42 − FEM_08
3 − Anne
20 − Kwanza
43 − FEM_09
29 − Scepter
44 − FEM_10
25 − Polka
45 − FEM_11
8 − Enrosadira
46 − FEM_12
40 − FEM_06
9 − Fall Gold
1 − Allgold
35 − FEM_01
37 − FEM_03
4 − Autumn Treasure

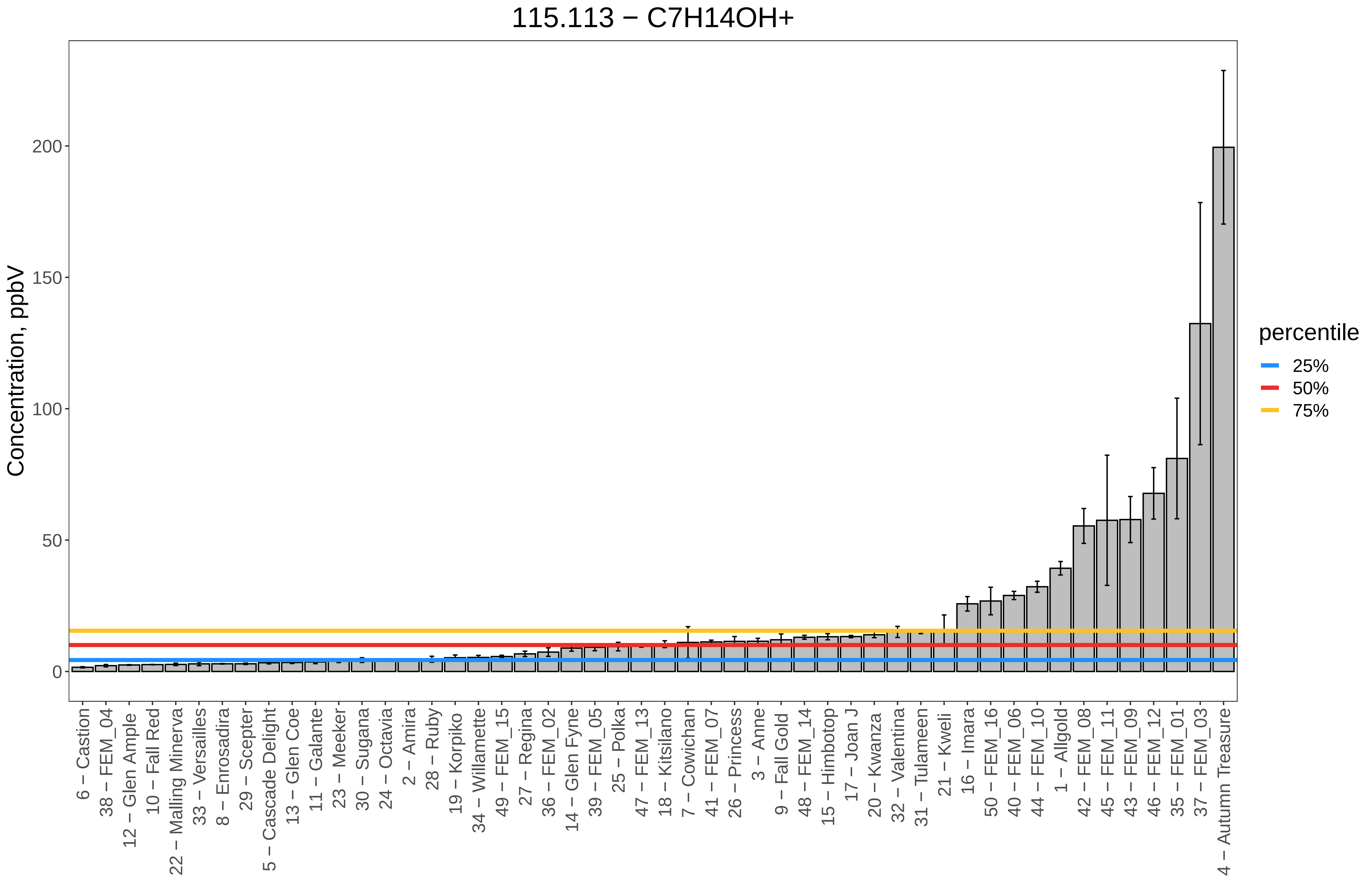

115.113 − C7H14OH+
Concentration, ppbV
0
50
100
150
200
percentile
25%
50%
75%
6 − Castion
38 − FEM_04
12 − Glen Ample
10 − Fall Red
22 − Malling Minerva
33 − Versailles
8 − Enrosadira
29 − Scepter
5 − Cascade Delight
13 − Glen Coe
11 − Galante
23 − Meeker
30 − Sugana
24 − Octavia
2 − Amira
28 − Ruby
19 − Korpiko
34 − Willamette
49 − FEM_15
27 − Regina
36 − FEM_02
14 − Glen Fyne
39 − FEM_05
25 − Polka
47 − FEM_13
18 − Kitsilano
7 − Cowichan
41 − FEM_07
26 − Princess
3 − Anne
9 − Fall Gold
48 − FEM_14
15 − Himbotop
17 − Joan J
20 − Kwanza
32 − Valentina
31 − Tulameen
21 − Kweli
16 − Imara
50 − FEM_16
40 − FEM_06
44 − FEM_10
1 − Allgold
42 − FEM_08
45 − FEM_11
43 − FEM_09
46 − FEM_12
35 − FEM_01
37 − FEM_03
4 − Autumn Treasure

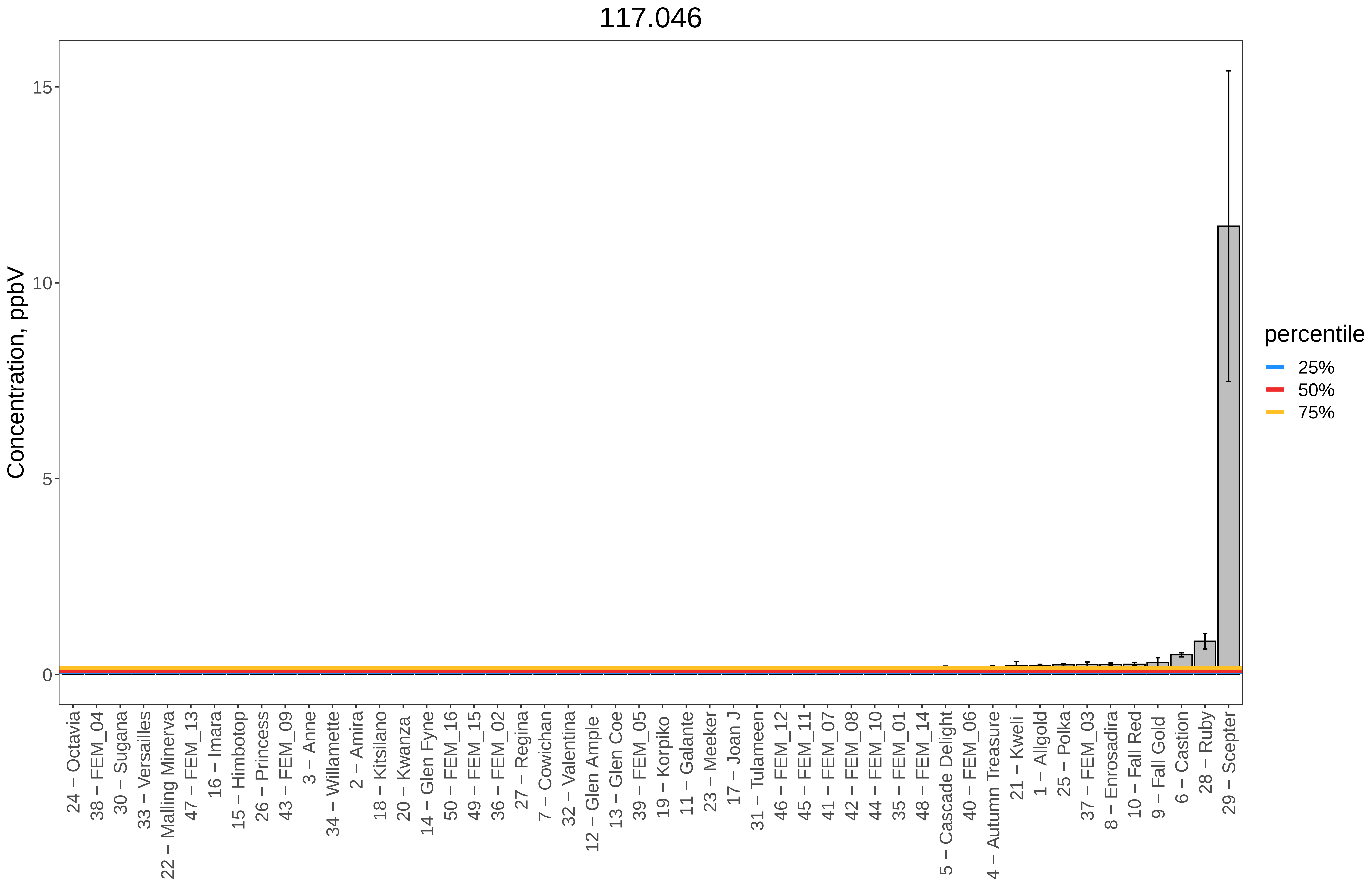
117.046
Concentration, ppbV
15
10
5
0
percentile
25%
50%
75%
24 – Octavia
38 – FEM_04
30 – Sugana
33 – Versailles
22 – Malling Minerva
47 – FEM_13
16 – Imara
15 – Himbotop
26 – Princess
43 – FEM_09
3 – Anne
34 – Willamette
2 – Amira
18 – Kitsilano
20 – Kwanza
14 – Glen Fyne
50 – FEM_16
49 – FEM_15
36 – FEM_02
27 – Regina
7 – Cowichan
32 – Valentina
12 – Glen Ample
13 – Glen Coe
39 – FEM_05
19 – Korpiko
11 – Galante
23 – Meeker
17 – Joan J
31 – Tulameen
46 – FEM_12
45 – FEM_11
41 – FEM_07
42 – FEM_08
44 – FEM_10
35 – FEM_01
48 – FEM_14
5 – Cascade Delight
40 – FEM_06
4 – Autumn Treasure
21 – Kweli
1 – Allgold
25 – Polka
37 – FEM_03
8 – Enrosadira
10 – Fall Red
9 – Fall Gold
6 – Castion
28 – Ruby
29 – Scepter

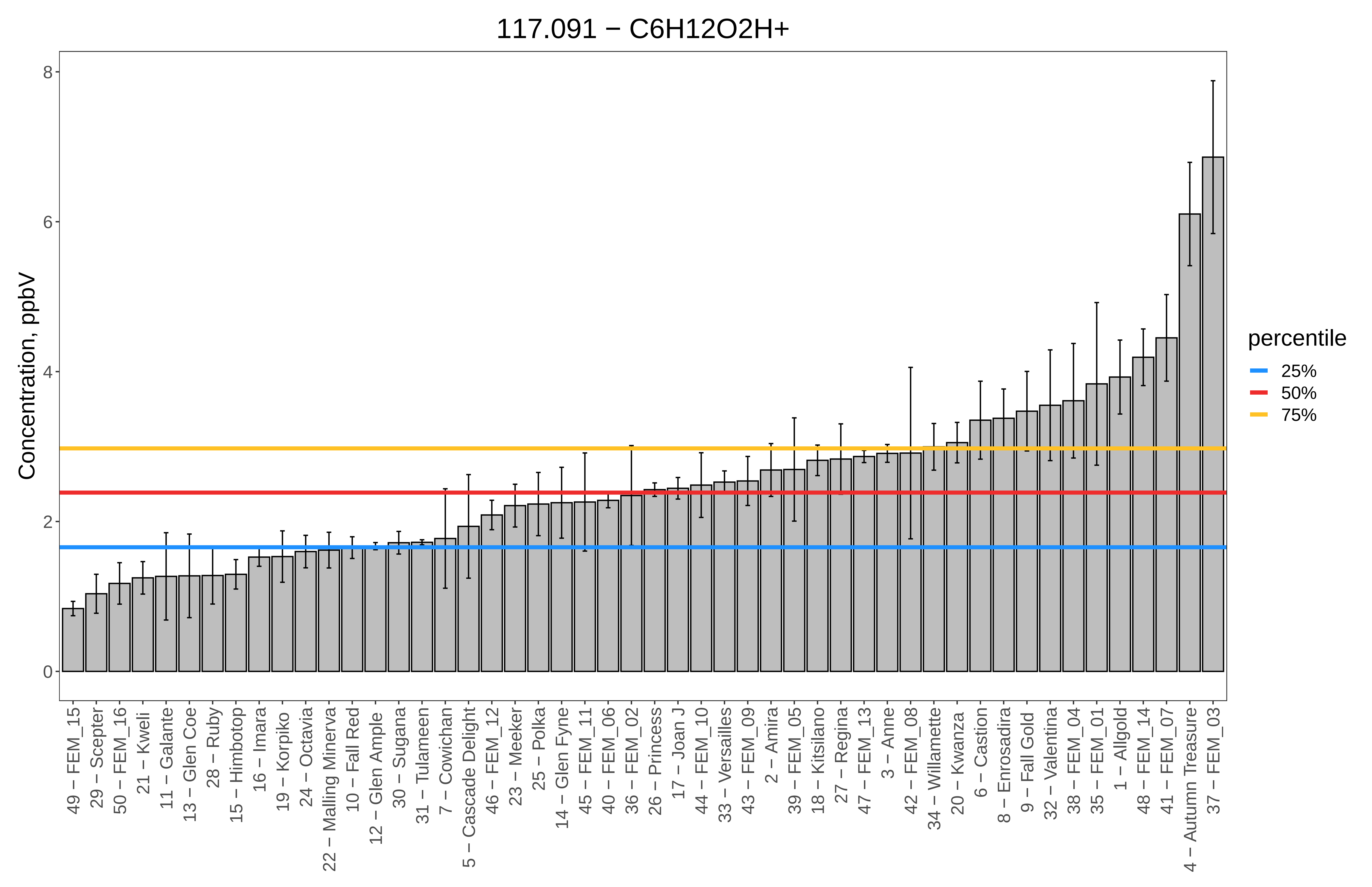
117.091 − C6H12O2H+
Concentration, ppbV
0
2
4
6
8
percentile
25%
50%
75%
49 − FEM_15
29 − Scepter
50 − FEM_16
21 − Kweli
11 − Galante
13 − Glen Coe
28 − Ruby
15 − Himbotop
16 − Imara
19 − Korpiko
24 − Octavia
22 − Malling Minerva
10 − Fall Red
12 − Glen Ample
30 − Sugana
31 − Tulameen
7 − Cowichan
5 − Cascade Delight
46 − FEM_12
23 − Meeker
25 − Polka
14 − Glen Fyne
45 − FEM_11
40 − FEM_06
36 − FEM_02
26 − Princess
17 − Joan J
44 − FEM_10
33 − Versailles
43 − FEM_09
2 − Amira
39 − FEM_05
18 − Kitsilano
27 − Regina
47 − FEM_13
3 − Anne
42 − FEM_08
34 − Willamette
20 − Kwanza
6 − Castion
8 − Enrosadira
9 − Fall Gold
32 − Valentina
38 − FEM_04
35 − FEM_01
1 − Allgold
48 − FEM_14
41 − FEM_07
4 − Autumn Treasure
37 − FEM_03

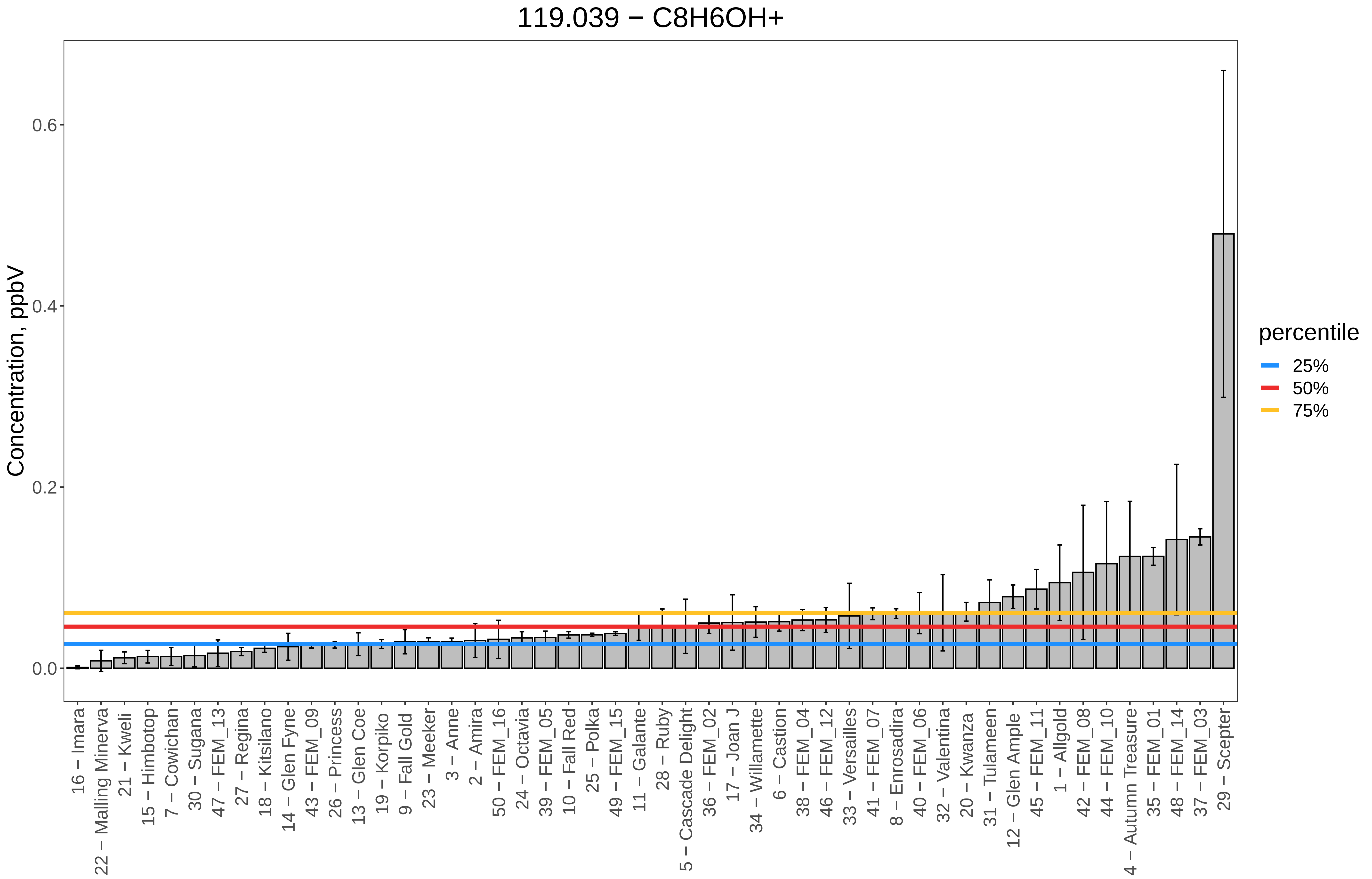

119.039 − C8H6OH+
Concentration, ppbV
0.0
0.2
0.4
0.6
percentile
25%
50%
75%
16 − Imara
22 − Malling Minerva
21 − Kweli
15 − Himbotop
7 − Cowichan
30 − Sugana
47 − FEM_13
27 − Regina
18 − Kitsilano
14 − Glen Fyne
43 − FEM_09
26 − Princess
13 − Glen Coe
19 − Korpiko
9 − Fall Gold
23 − Meeker
3 − Anne
2 − Amira
50 − FEM_16
24 − Octavia
39 − FEM_05
10 − Fall Red
25 − Polka
49 − FEM_15
11 − Galante
28 − Ruby
5 − Cascade Delight
36 − FEM_02
17 − Joan J
34 − Willamette
6 − Castion
38 − FEM_04
46 − FEM_12
33 − Versailles
41 − FEM_07
8 − Enrosadira
40 − FEM_06
32 − Valentina
20 − Kwanza
31 − Tulameen
12 − Glen Ample
45 − FEM_11
1 − Allgold
42 − FEM_08
44 − FEM_10
4 − Autumn Treasure
35 − FEM_01
48 − FEM_14
37 − FEM_03
29 − Scepter

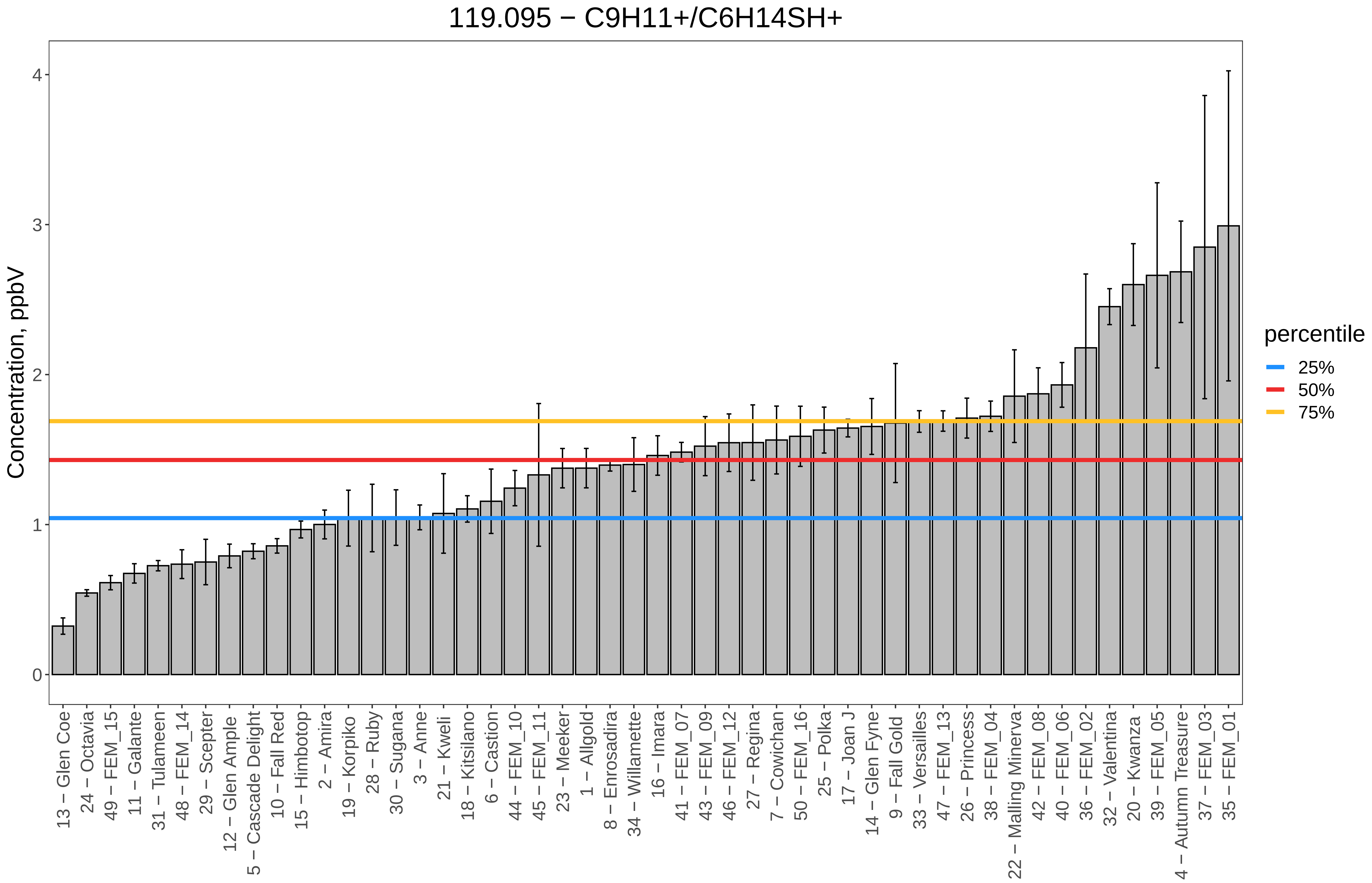

119.095 − C9H11+/C6H14SH+
Concentration, ppbV
0
1
2
3
4
percentile
25%
50%
75%
13 − Glen Coe
24 − Octavia
49 − FEM_15
11 − Galante
31 − Tulameen
48 − FEM_14
29 − Scepter
12 − Glen Ample
5 − Cascade Delight
10 − Fall Red
15 − Himbotop
2 − Amira
19 − Korpiko
28 − Ruby
30 − Sugana
3 − Anne
21 − Kweli
18 − Kitsilano
6 − Castion
44 − FEM_10
45 − FEM_11
23 − Meeker
1 − Allgold
8 − Enrosadira
34 − Willamette
16 − Imara
41 − FEM_07
43 − FEM_09
46 − FEM_12
27 − Regina
7 − Cowichan
50 − FEM_16
25 − Polka
17 − Joan J
14 − Glen Fyne
9 − Fall Gold
33 − Versailles
47 − FEM_13
26 − Princess
38 − FEM_04
22 − Malling Minerva
42 − FEM_08
40 − FEM_06
36 − FEM_02
32 − Valentina
20 − Kwanza
39 − FEM_05
4 − Autumn Treasure
37 − FEM_03
35 − FEM_01

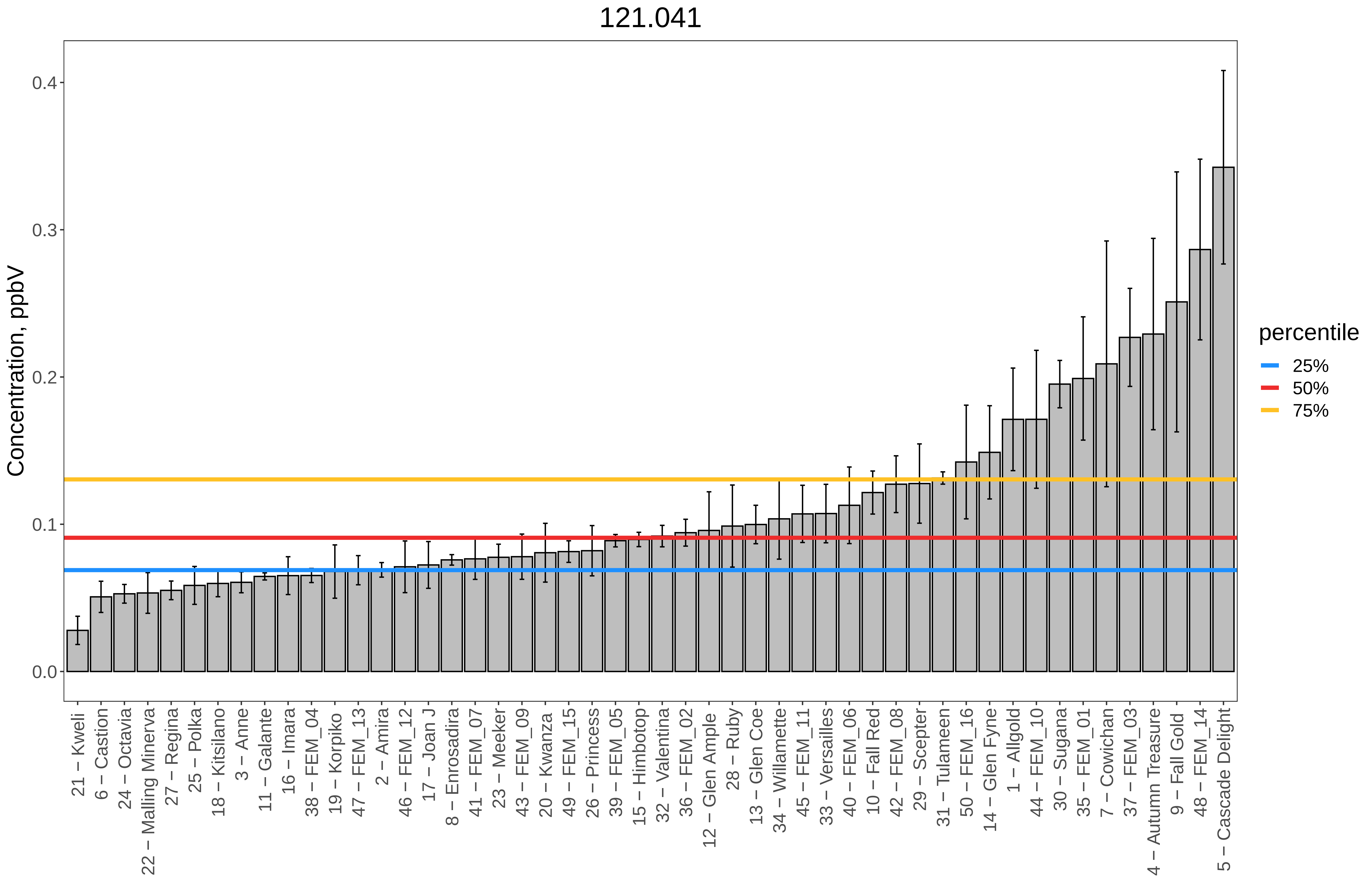

121.041
Concentration, ppbV
0.0
0.1
0.2
0.3
0.4
percentile
25%
50%
75%
21 – Kweli
6 – Castion
24 – Octavia
22 – Malling Minerva
27 – Regina
25 – Polka
18 – Kitsilano
3 – Anne
11 – Galante
16 – Imara
38 – FEM_04
19 – Korpiko
47 – FEM_13
2 – Amira
46 – FEM_12
17 – Joan J
8 – Enrosadira
41 – FEM_07
23 – Meeker
43 – FEM_09
20 – Kwanza
49 – FEM_15
26 – Princess
39 – FEM_05
15 – Himbotop
32 – Valentina
36 – FEM_02
12 – Glen Ample
28 – Ruby
13 – Glen Coe
34 – Willamette
45 – FEM_11
33 – Versailles
40 – FEM_06
10 – Fall Red
42 – FEM_08
29 – Scepter
31 – Tulameen
50 – FEM_16
14 – Glen Fyne
1 – Allgold
44 – FEM_10
30 – Sugana
35 – FEM_01
7 – Cowichan
37 – FEM_03
4 – Autumn Treasure
9 – Fall Gold
48 – FEM_14
5 – Cascade Delight

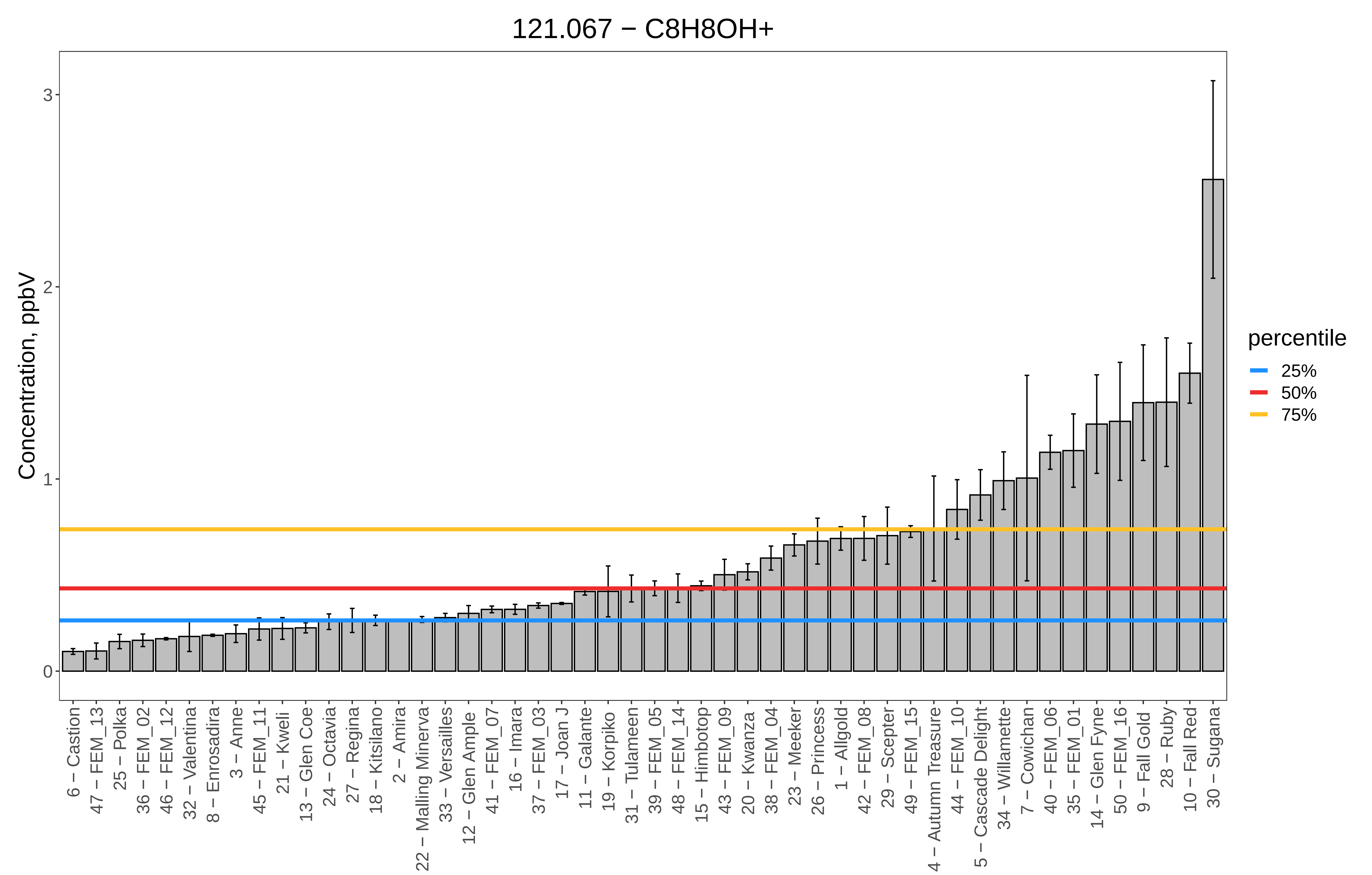

121.067 − C8H8OH+
Concentration, ppbV
0
1
2
3
percentile
25%
50%
75%
6 − Castion
47 − FEM_13
25 − Polka
36 − FEM_02
46 − FEM_12
32 − Valentina
8 − Enrosadira
3 − Anne
45 − FEM_11
21 − Kweli
13 − Glen Coe
24 − Octavia
27 − Regina
18 − Kitsilano
2 − Amira
22 − Malling Minerva
33 − Versailles
12 − Glen Ample
41 − FEM_07
16 − Imara
37 − FEM_03
17 − Joan J
11 − Galante
19 − Korpiko
31 − Tulameen
39 − FEM_05
48 − FEM_14
15 − Himbotop
43 − FEM_09
20 − Kwanza
38 − FEM_04
23 − Meeker
26 − Princess
1 − Allgold
42 − FEM_08
29 − Scepter
49 − FEM_15
4 − Autumn Treasure
44 − FEM_10
5 − Cascade Delight
34 − Willamette
7 − Cowichan
40 − FEM_06
35 − FEM_01
14 − Glen Fyne
50 − FEM_16
9 − Fall Gold
28 − Ruby
10 − Fall Red
30 − Sugana

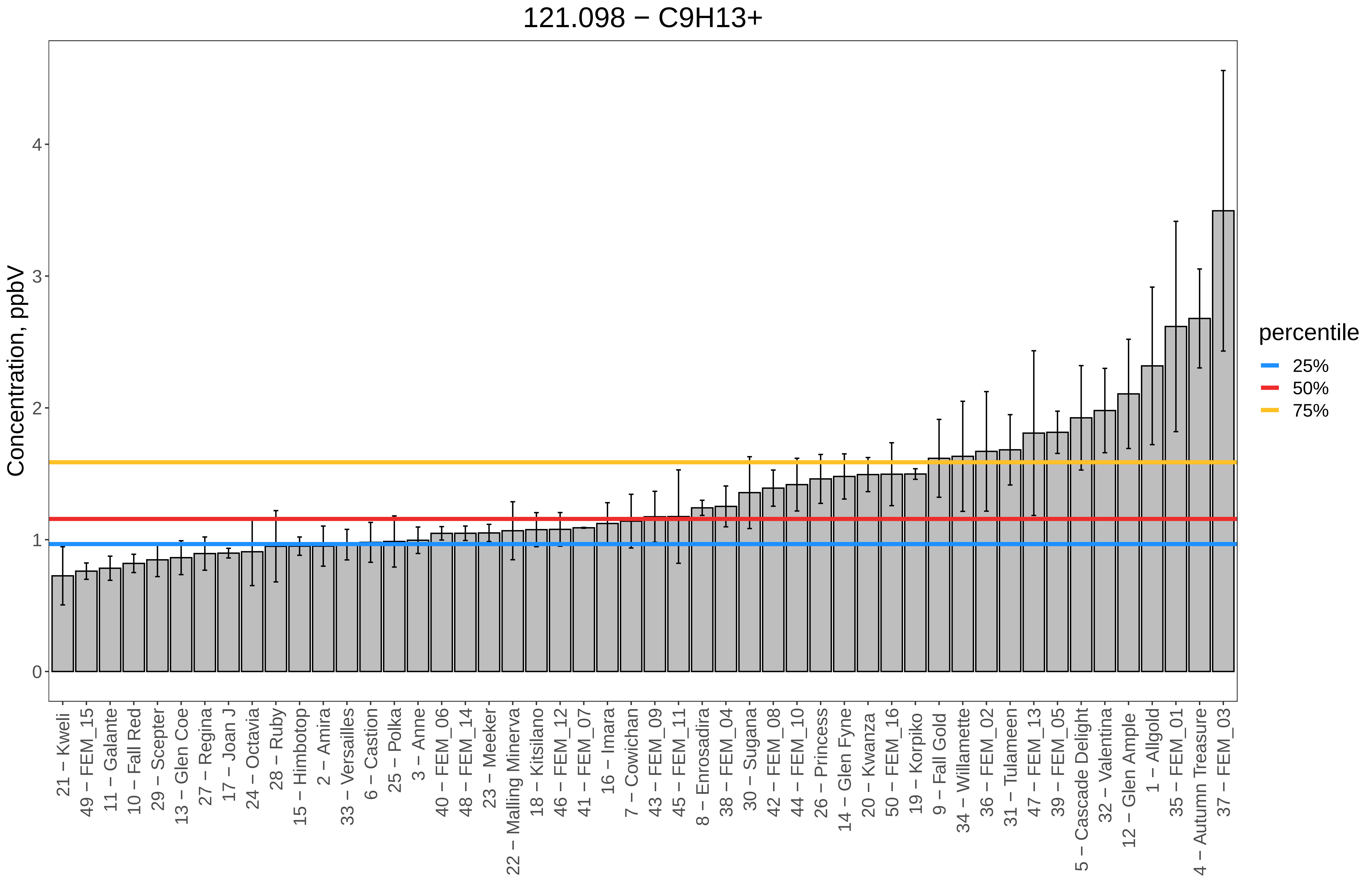

121.098 − C9H13+
Concentration, ppbV
0
1
2
3
4
percentile
25%
50%
75%
21 − Kweli
49 − FEM_15
11 − Galante
10 − Fall Red
29 − Scepter
13 − Glen Coe
27 − Regina
17 − Joan J
24 − Octavia
28 − Ruby
15 − Himbotop
2 − Amira
33 − Versailles
6 − Castion
25 − Polka
3 − Anne
40 − FEM_06
48 − FEM_14
23 − Meeker
22 − Malling Minerva
18 − Kitsilano
46 − FEM_12
41 − FEM_07
16 − Imara
7 − Cowichan
43 − FEM_09
45 − FEM_11
8 − Enrosadira
38 − FEM_04
30 − Sugana
42 − FEM_08
44 − FEM_10
26 − Princess
14 − Glen Fyne
20 − Kwanza
50 − FEM_16
19 − Korpiko
9 − Fall Gold
34 − Willamette
36 − FEM_02
31 − Tulameen
47 − FEM_13
39 − FEM_05
5 − Cascade Delight
32 − Valentina
12 − Glen Ample
1 − Allgold
35 − FEM_01
4 − Autumn Treasure
37 − FEM_03

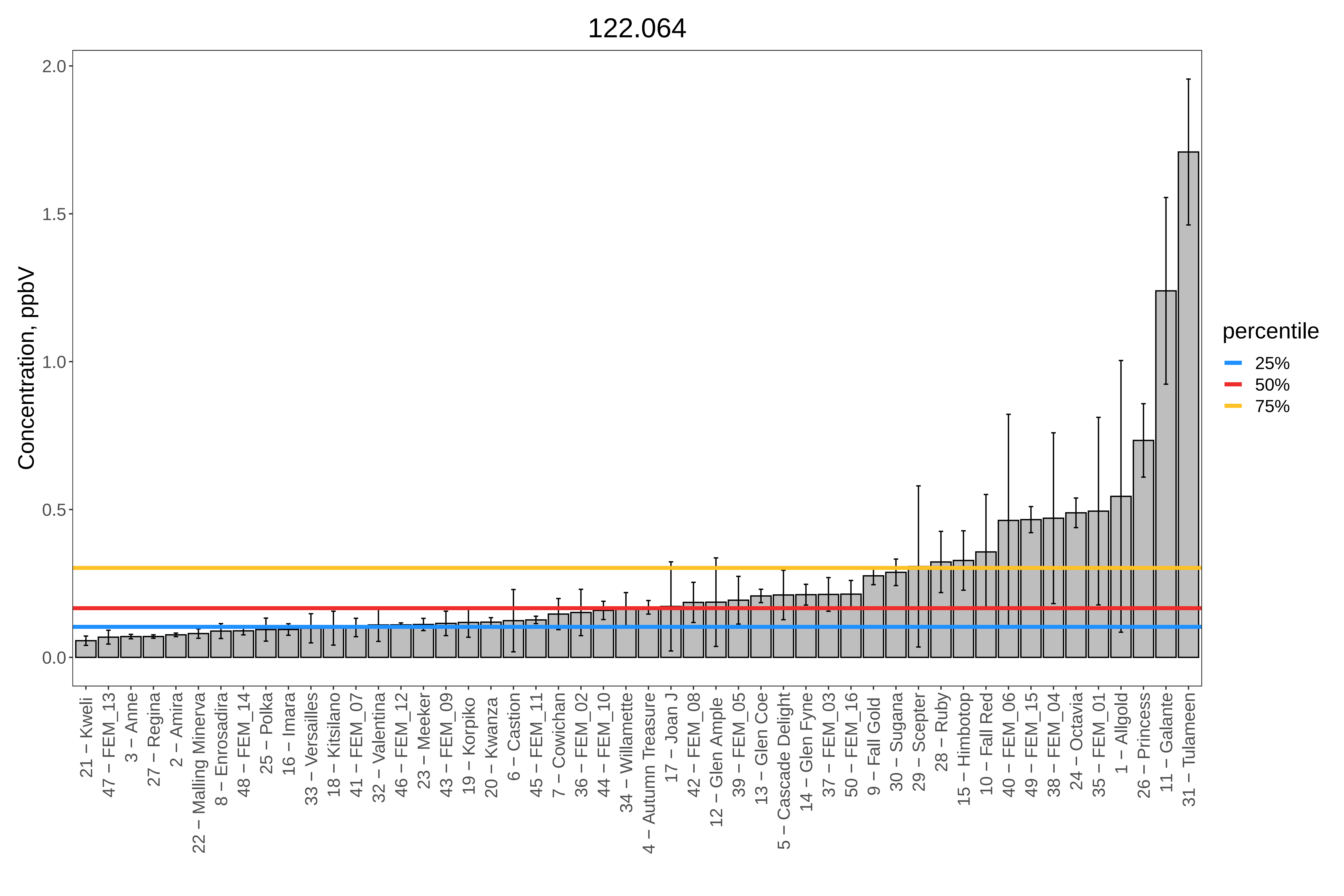

122.064
Concentration, ppbV
0.0
0.5
1.0
1.5
2.0
percentile
25%
50%
75%
21 – Kweli
47 – FEM_13
3 – Anne
27 – Regina
2 – Amira
22 – Malling Minerva
8 – Enrosadira
48 – FEM_14
25 – Polka
16 – Imara
33 – Versailles
18 – Kitsilano
41 – FEM_07
32 – Valentina
46 – FEM_12
23 – Meeker
43 – FEM_09
19 – Korpiko
20 – Kwanza
6 – Castion
45 – FEM_11
7 – Cowichan
36 – FEM_02
44 – FEM_10
34 – Willamette
4 – Autumn Treasure
17 – Joan J
42 – FEM_08
12 – Glen Ample
39 – FEM_05
13 – Glen Coe
5 – Cascade Delight
14 – Glen Fyne
37 – FEM_03
50 – FEM_16
9 – Fall Gold
30 – Sugana
29 – Scepter
28 – Ruby
15 – Himbotop
10 – Fall Red
40 – FEM_06
49 – FEM_15
38 – FEM_04
24 – Octavia
35 – FEM_01
1 – Allgold
26 – Princess
11 – Galante
31 – Tulameen

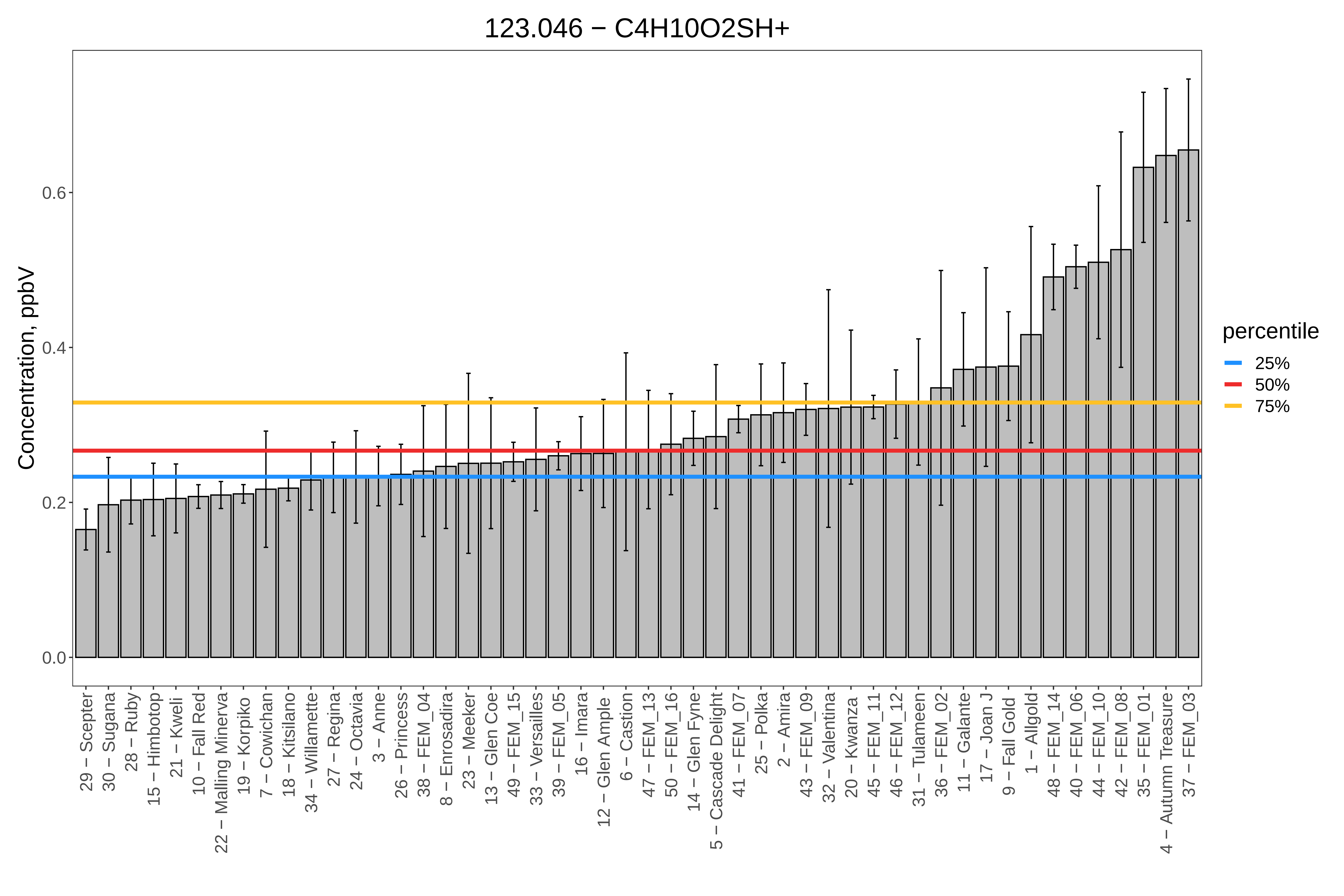
123.046 − C4H10O2SH+
Concentration, ppbV
0.0
0.2
0.4
0.6
percentile
25%
50%
75%
29 − Scepter
30 − Sugana
28 − Ruby
15 − Himbotop
21 − Kweli
10 − Fall Red
22 − Malling Minerva
19 − Korpiko
7 − Cowichan
18 − Kitsilano
34 − Willamette
27 − Regina
24 − Octavia
3 − Anne
26 − Princess
38 − FEM_04
8 − Enrosadira
23 − Meeker
13 − Glen Coe
49 − FEM_15
33 − Versailles
39 − FEM_05
16 − Imara
12 − Glen Ample
6 − Castion
47 − FEM_13
50 − FEM_16
14 − Glen Fyne
5 − Cascade Delight
41 − FEM_07
25 − Polka
2 − Amira
43 − FEM_09
32 − Valentina
20 − Kwanza
45 − FEM_11
46 − FEM_12
31 − Tulameen
36 − FEM_02
11 − Galante
17 − Joan J
9 − Fall Gold
1 − Allgold
48 − FEM_14
40 − FEM_06
44 − FEM_10
42 − FEM_08
35 − FEM_01
4 − Autumn Treasure
37 − FEM_03

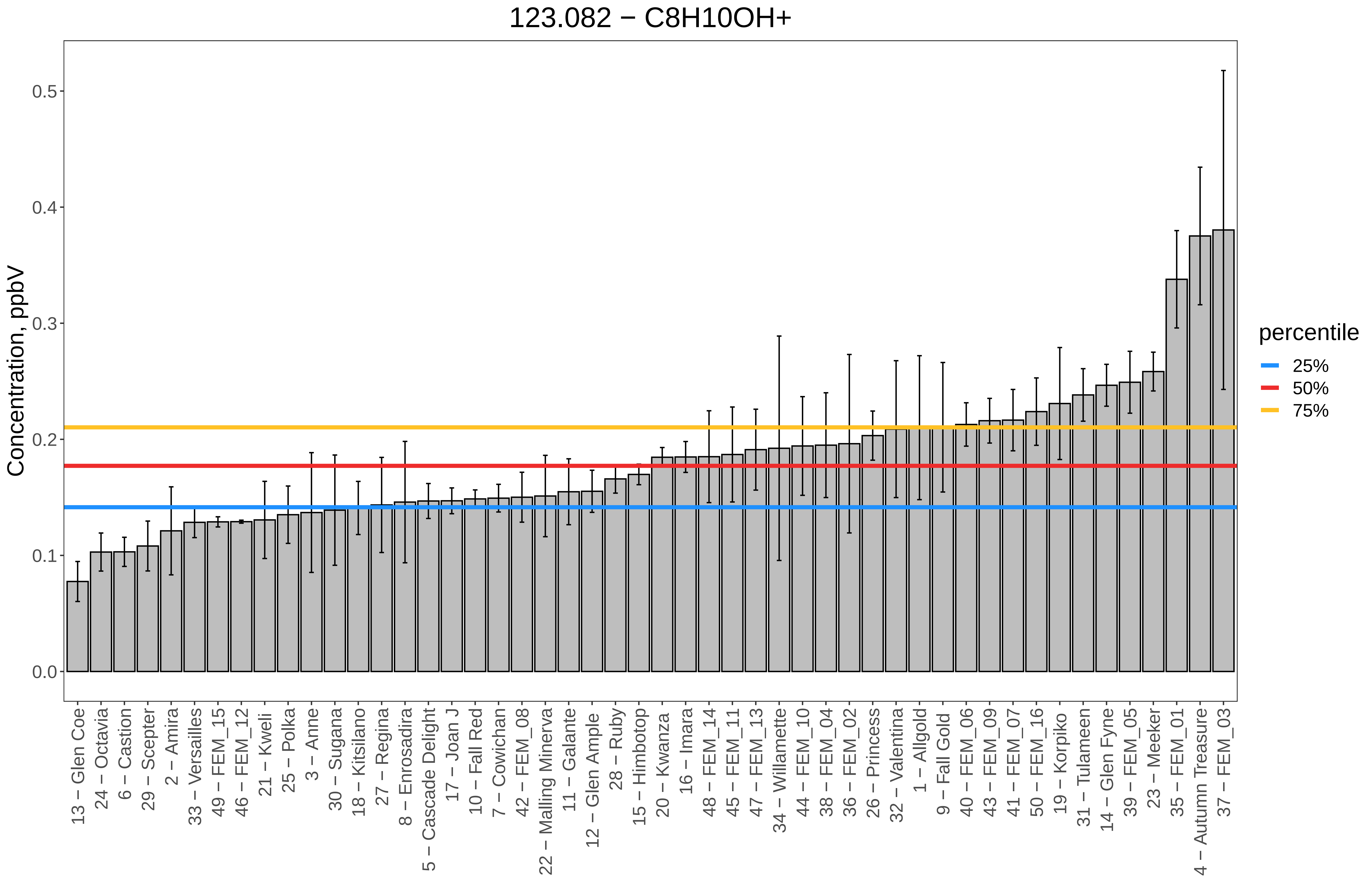

123.082 − C8H10OH+
Concentration, ppbV
0.0
0.1
0.2
0.3
0.4
0.5
percentile
25%
50%
75%
13 − Glen Coe
24 − Octavia
6 − Castion
29 − Scepter
2 − Amira
33 − Versailles
49 − FEM_15
46 − FEM_12
21 − Kweli
25 − Polka
3 − Anne
30 − Sugana
18 − Kitsilano
27 − Regina
8 − Enrosadira
5 − Cascade Delight
17 − Joan J
10 − Fall Red
7 − Cowichan
42 − FEM_08
22 − Malling Minerva
11 − Galante
12 − Glen Ample
28 − Ruby
15 − Himbotop
20 − Kwanza
16 − Imara
48 − FEM_14
45 − FEM_11
47 − FEM_13
34 − Willamette
44 − FEM_10
38 − FEM_04
36 − FEM_02
26 − Princess
32 − Valentina
1 − Allgold
9 − Fall Gold
40 − FEM_06
43 − FEM_09
41 − FEM_07
50 − FEM_16
19 − Korpiko
31 − Tulameen
14 − Glen Fyne
39 − FEM_05
23 − Meeker
35 − FEM_01
4 − Autumn Treasure
37 − FEM_03

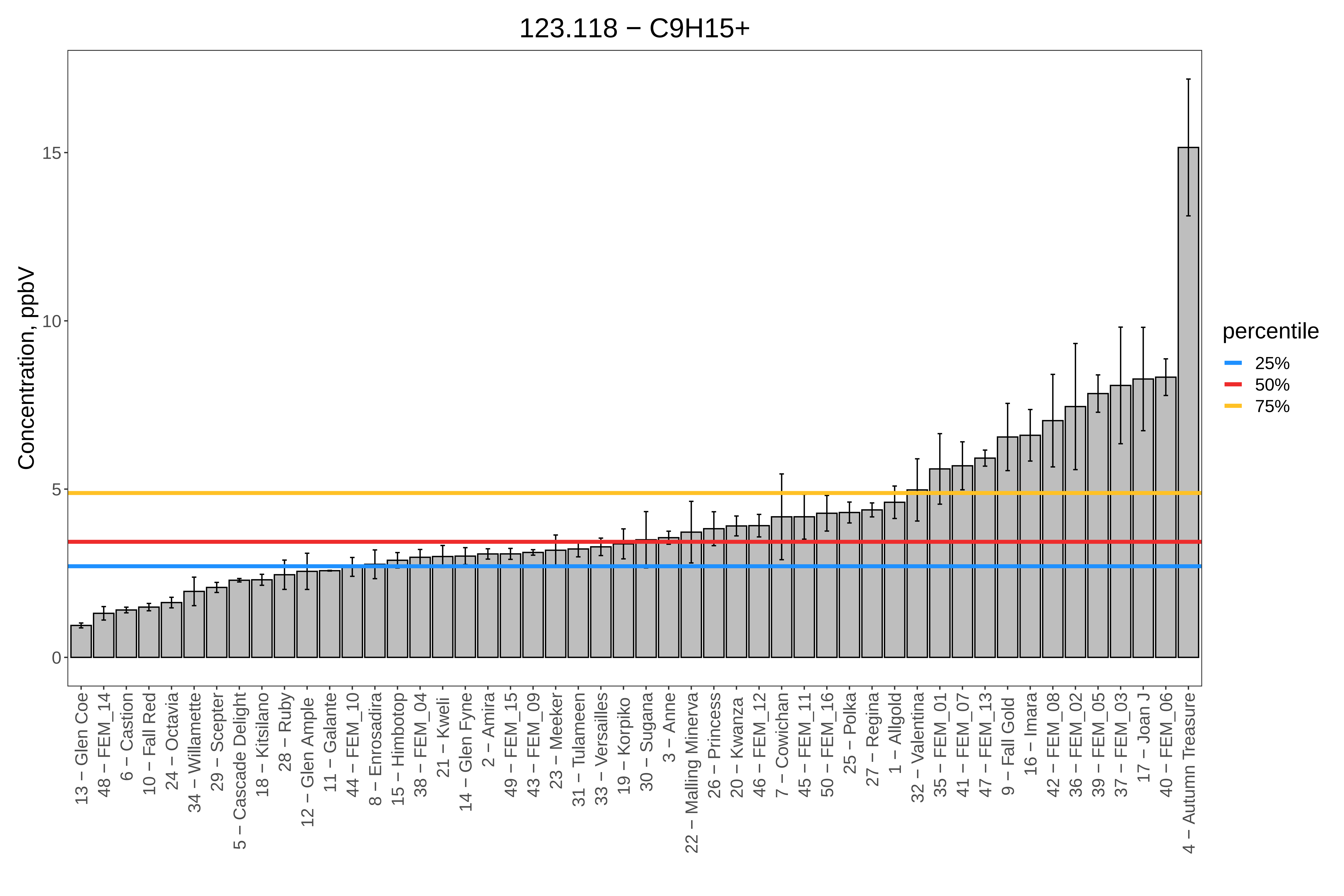

123.118 − C9H15+
Concentration, ppbV
0
5
10
15
percentile
25%
50%
75%
13 − Glen Coe
48 − FEM_14
6 − Castion
10 − Fall Red
24 − Octavia
34 − Willamette
29 − Scepter
5 − Cascade Delight
18 − Kitsilano
28 − Ruby
12 − Glen Ample
11 − Galante
44 − FEM_10
8 − Enrosadira
15 − Himbotop
38 − FEM_04
21 − Kweli
14 − Glen Fyne
2 − Amira
49 − FEM_15
43 − FEM_09
23 − Meeker
31 − Tulameen
33 − Versailles
19 − Korpiko
30 − Sugana
3 − Anne
22 − Malling Minerva
26 − Princess
20 − Kwanza
46 − FEM_12
7 − Cowichan
45 − FEM_11
50 − FEM_16
25 − Polka
27 − Regina
1 − Allgold
32 − Valentina
35 − FEM_01
41 − FEM_07
47 − FEM_13
9 − Fall Gold
16 − Imara
42 − FEM_08
36 − FEM_02
39 − FEM_05
37 − FEM_03
17 − Joan J
40 − FEM_06
4 − Autumn Treasure

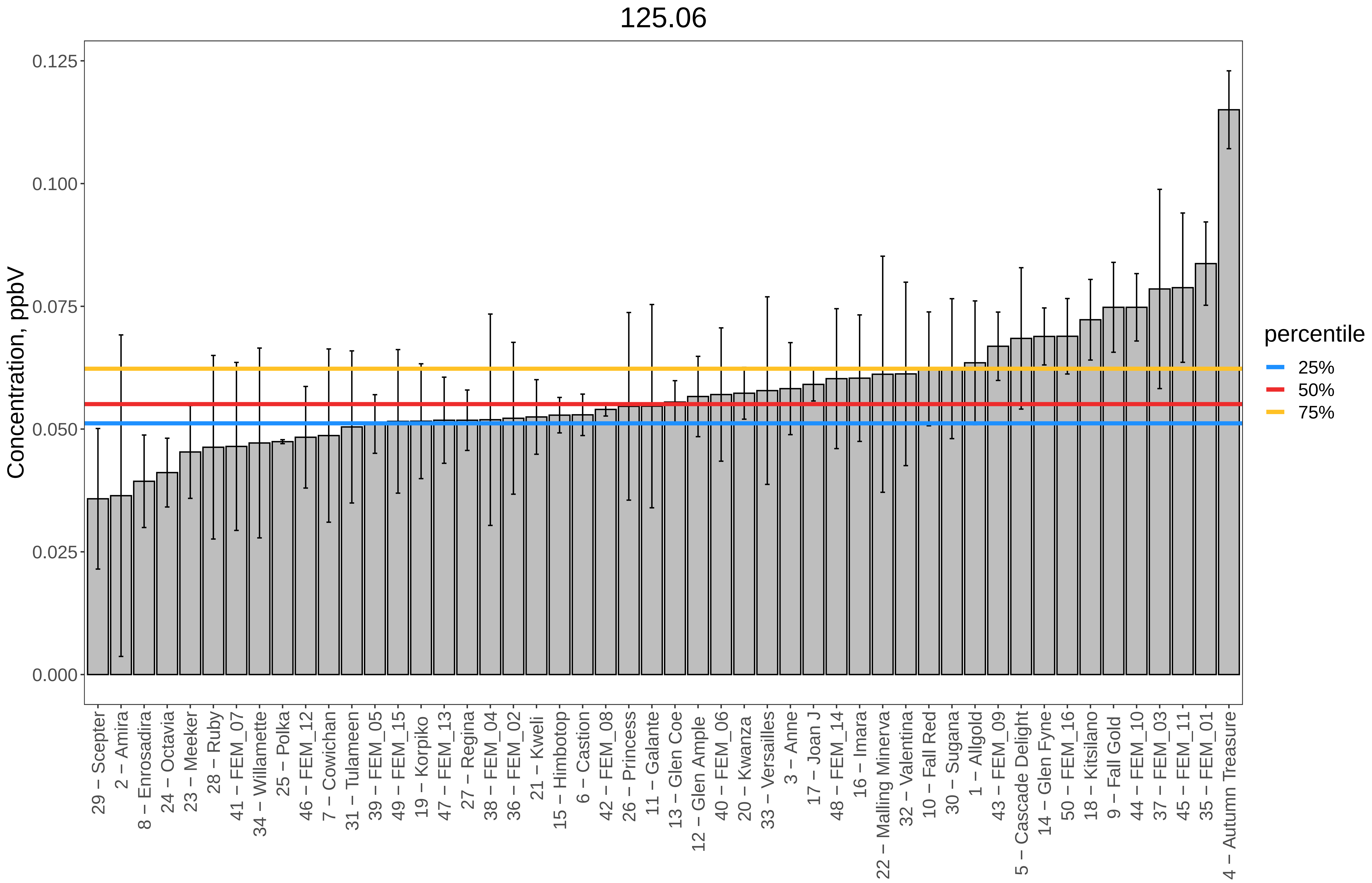
125.06
Concentration, ppbV
0.125
0.100
0.075
0.050
0.025
0.000
percentile
25%
50%
75%
29 – Scepter
2 – Amira
8 – Enrosadira
24 – Octavia
23 – Meeker
28 – Ruby
41 – FEM_07
34 – Willamette
25 – Polka
46 – FEM_12
7 – Cowichan
31 – Tulameen
39 – FEM_05
49 – FEM_15
19 – Korpiko
47 – FEM_13
27 – Regina
38 – FEM_04
36 – FEM_02
21 – Kweli
15 – Himbotop
6 – Castion
42 – FEM_08
26 – Princess
11 – Galante
13 – Glen Coe
12 – Glen Ample
40 – FEM_06
20 – Kwanza
33 – Versailles
3 – Anne
17 – Joan J
48 – FEM_14
16 – Imara
22 – Malling Minerva
32 – Valentina
10 – Fall Red
30 – Sugana
1 – Allgold
43 – FEM_09
5 – Cascade Delight
14 – Glen Fyne
50 – FEM_16
18 – Kitsilano
9 – Fall Gold
44 – FEM_10
37 – FEM_03
45 – FEM_11
35 – FEM_01
4 – Autumn Treasure

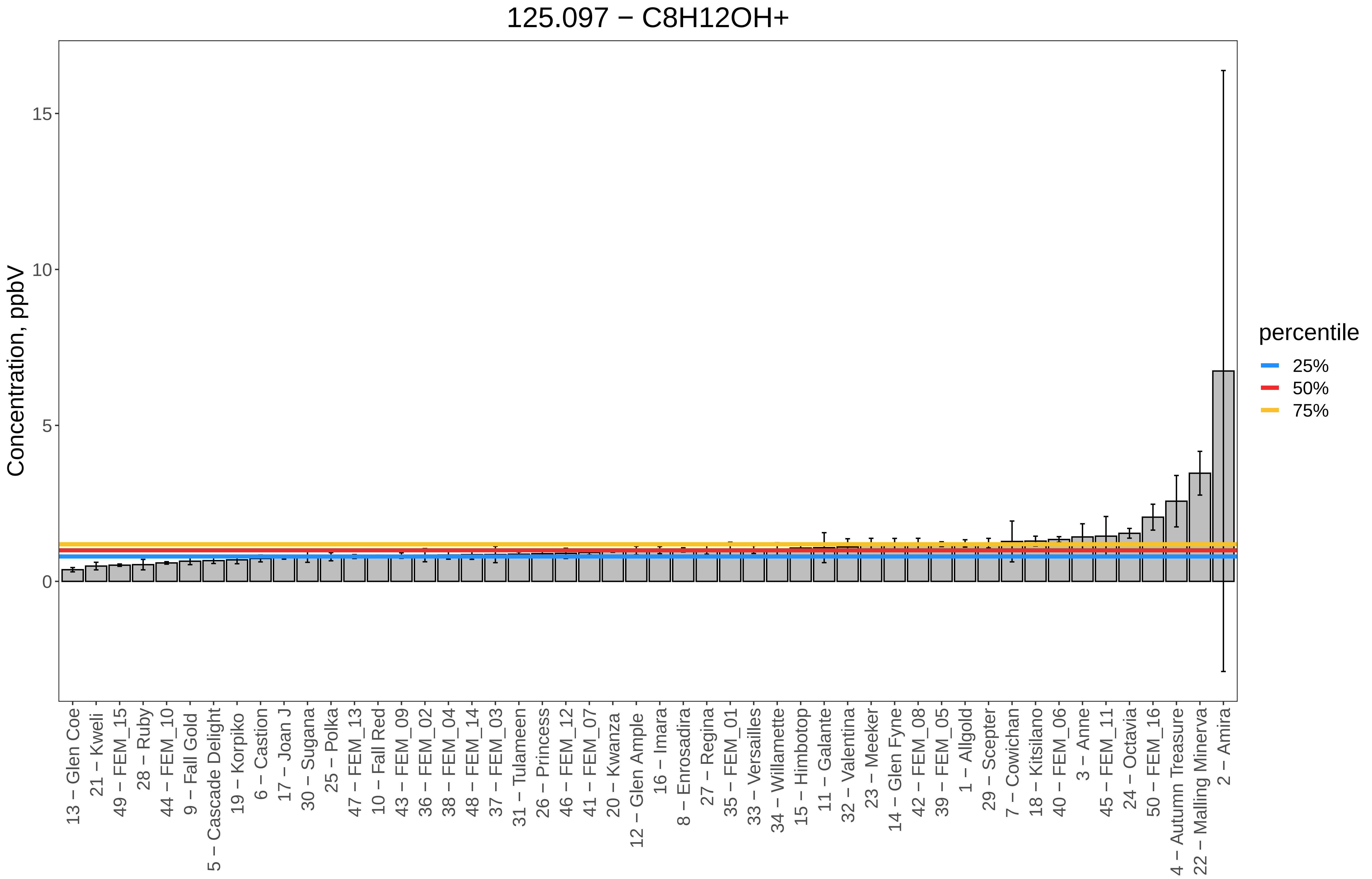
125.097 − C8H12OH+
Concentration, ppbV
0
5
10
15
percentile
25%
50%
75%
13 − Glen Coe
21 − Kweli
49 − FEM_15
28 − Ruby
44 − FEM_10
9 − Fall Gold
5 − Cascade Delight
19 − Korpiko
6 − Castion
17 − Joan J
30 − Sugana
25 − Polka
47 − FEM_13
10 − Fall Red
43 − FEM_09
36 − FEM_02
38 − FEM_04
48 − FEM_14
37 − FEM_03
31 − Tulameen
26 − Princess
46 − FEM_12
41 − FEM_07
20 − Kwanza
12 − Glen Ample
16 − Imara
8 − Enrosadira
27 − Regina
35 − FEM_01
33 − Versailles
34 − Willamette
15 − Himbotop
11 − Galante
32 − Valentina
23 − Meeker
14 − Glen Fyne
42 − FEM_08
39 − FEM_05
1 − Allgold
29 − Scepter
7 − Cowichan
18 − Kitsilano
40 − FEM_06
3 − Anne
45 − FEM_11
24 − Octavia
50 − FEM_16
4 − Autumn Treasure
22 − Malling Minerva
2 − Amira

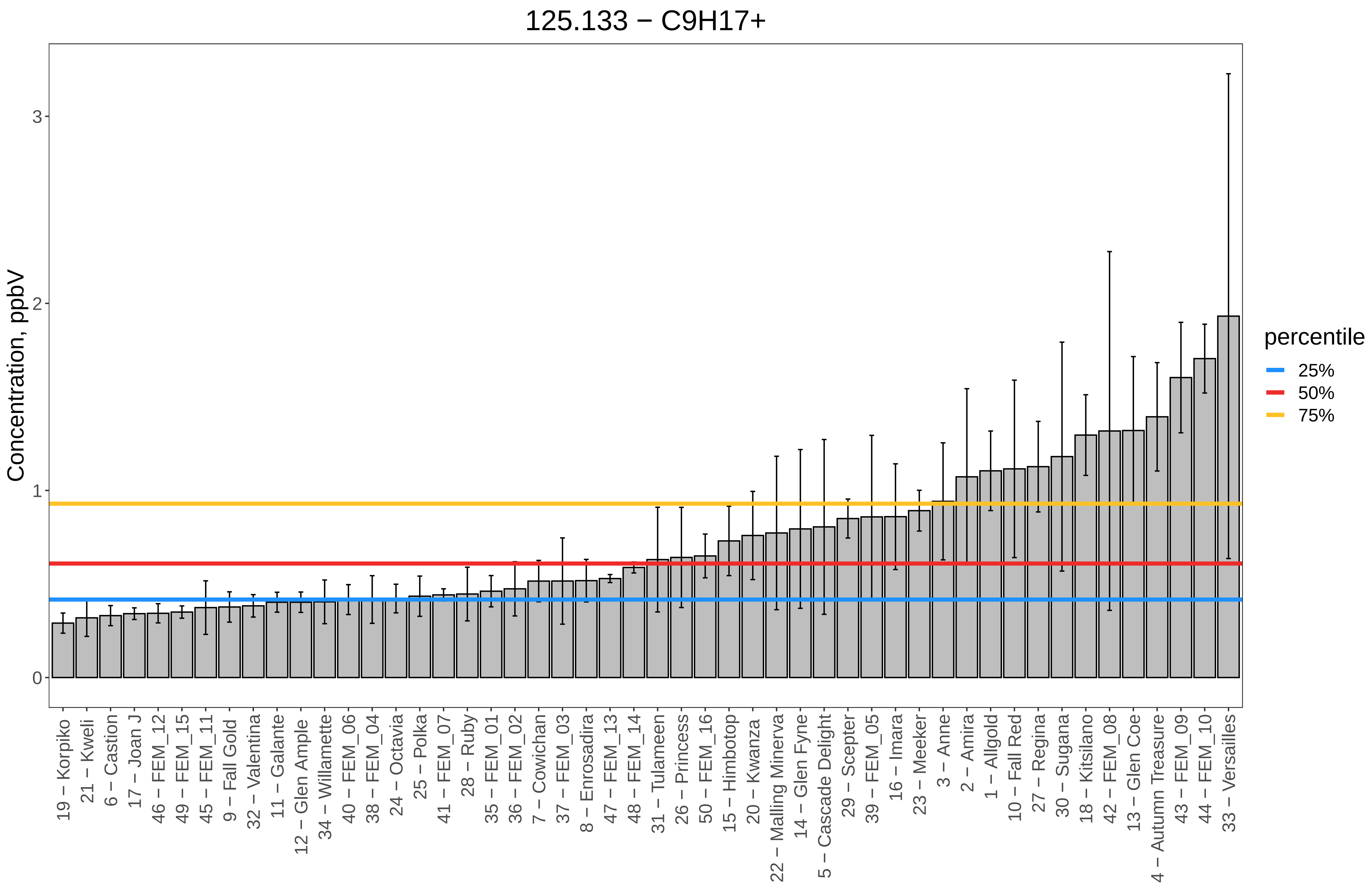

125.133 − C9H17+
Concentration, ppbV
0
1
2
3
percentile
25%
50%
75%
19 − Korpiko
21 − Kweli
6 − Castion
17 − Joan J
46 − FEM_12
49 − FEM_15
45 − FEM_11
9 − Fall Gold
32 − Valentina
11 − Galante
12 − Glen Ample
34 − Willamette
40 − FEM_06
38 − FEM_04
24 − Octavia
25 − Polka
41 − FEM_07
28 − Ruby
35 − FEM_01
36 − FEM_02
7 − Cowichan
37 − FEM_03
8 − Enrosadira
47 − FEM_13
48 − FEM_14
31 − Tulameen
26 − Princess
50 − FEM_16
15 − Himbotop
20 − Kwanza
22 − Malling Minerva
14 − Glen Fyne
5 − Cascade Delight
29 − Scepter
39 − FEM_05
16 − Imara
23 − Meeker
3 − Anne
2 − Amira
1 − Allgold
10 − Fall Red
27 − Regina
30 − Sugana
18 − Kitsilano
42 − FEM_08
13 − Glen Coe
4 − Autumn Treasure
43 − FEM_09
44 − FEM_10
33 − Versailles

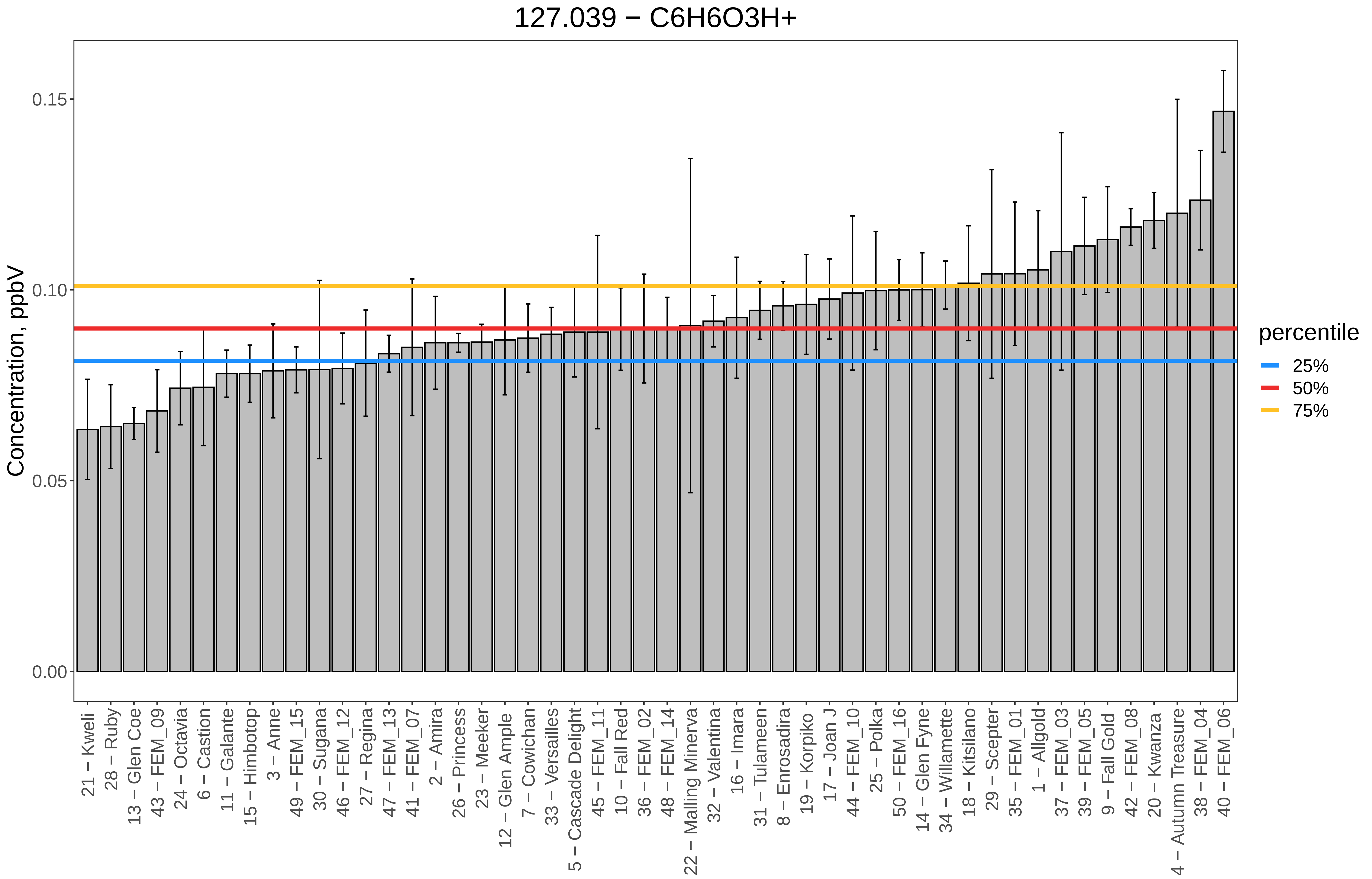
127.039 − C6H6O3H+
Concentration, ppbV
0.00
0.05
0.10
0.15
percentile
25%
50%
75%
21 − Kweli
28 − Ruby
13 − Glen Coe
43 − FEM_09
24 − Octavia
6 − Castion
11 − Galante
15 − Himbotop
3 − Anne
49 − FEM_15
30 − Sugana
46 − FEM_12
27 − Regina
47 − FEM_13
41 − FEM_07
2 − Amira
26 − Princess
23 − Meeker
12 − Glen Ample
7 − Cowichan
33 − Versailles
5 − Cascade Delight
45 − FEM_11
10 − Fall Red
36 − FEM_02
48 − FEM_14
22 − Malling Minerva
32 − Valentina
16 − Imara
31 − Tulameen
8 − Enrosadira
19 − Korpiko
17 − Joan J
44 − FEM_10
25 − Polka
50 − FEM_16
14 − Glen Fyne
34 − Willamette
18 − Kitsilano
29 − Scepter
35 − FEM_01
1 − Allgold
37 − FEM_03
39 − FEM_05
9 − Fall Gold
42 − FEM_08
20 − Kwanza
4 − Autumn Treasure
38 − FEM_04
40 − FEM_06

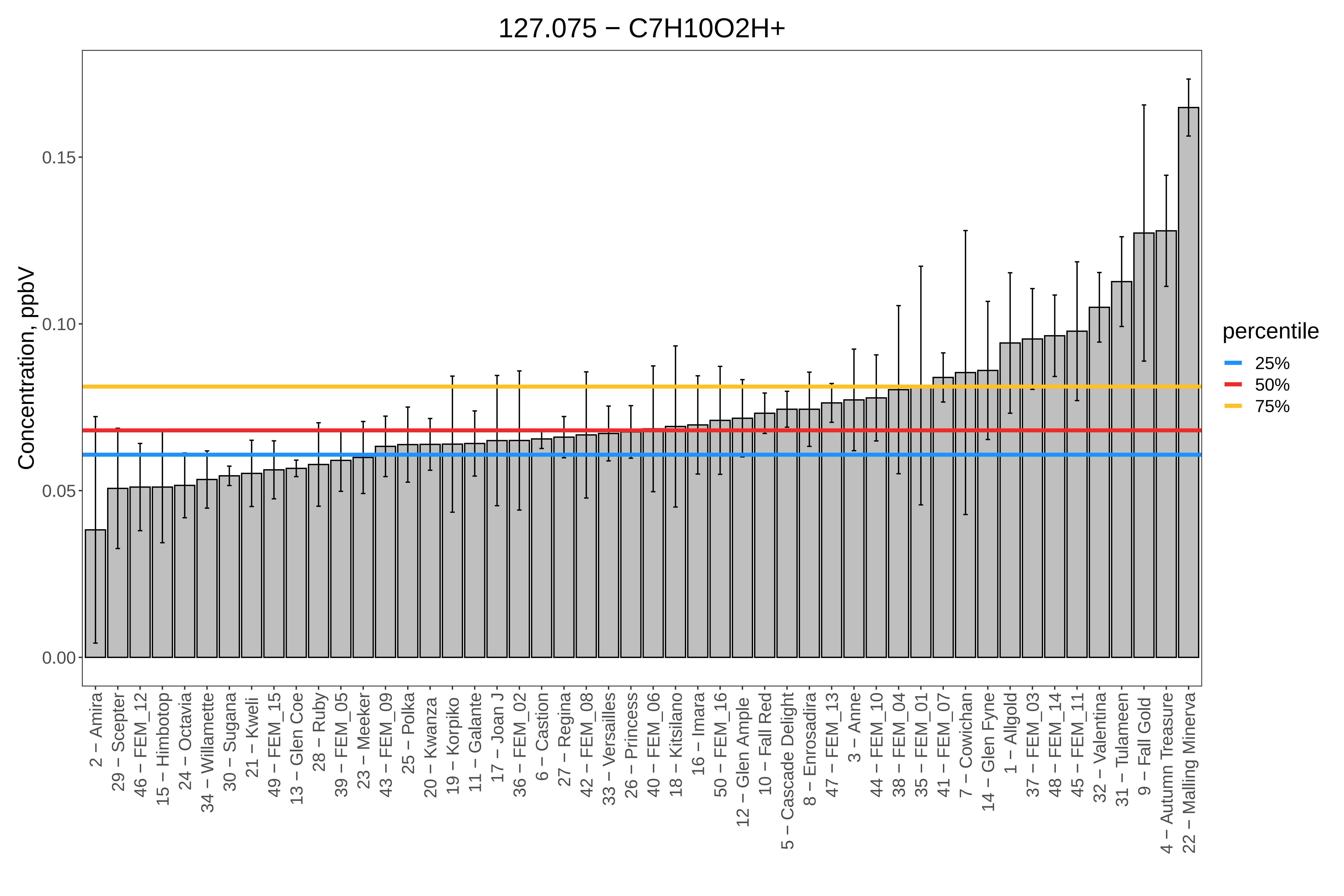
127.075 − C7H10O2H+
Concentration, ppbV
0.00
0.05
0.10
0.15
percentile
25%
50%
75%
2 − Amira
29 − Scepter
46 − FEM_12
15 − Himbotop
24 − Octavia
34 − Willamette
30 − Sugana
21 − Kweli
49 − FEM_15
13 − Glen Coe
28 − Ruby
39 − FEM_05
23 − Meeker
43 − FEM_09
25 − Polka
20 − Kwanza
19 − Korpiko
11 − Galante
17 − Joan J
36 − FEM_02
6 − Castion
27 − Regina
42 − FEM_08
33 − Versailles
26 − Princess
40 − FEM_06
18 − Kitsilano
16 − Imara
50 − FEM_16
12 − Glen Ample
10 − Fall Red
5 − Cascade Delight
8 − Enrosadira
47 − FEM_13
3 − Anne
44 − FEM_10
38 − FEM_04
35 − FEM_01
41 − FEM_07
7 − Cowichan
14 − Glen Fyne
1 − Allgold
37 − FEM_03
48 − FEM_14
45 − FEM_11
32 − Valentina
31 − Tulameen
9 − Fall Gold
4 − Autumn Treasure
22 − Malling Minerva

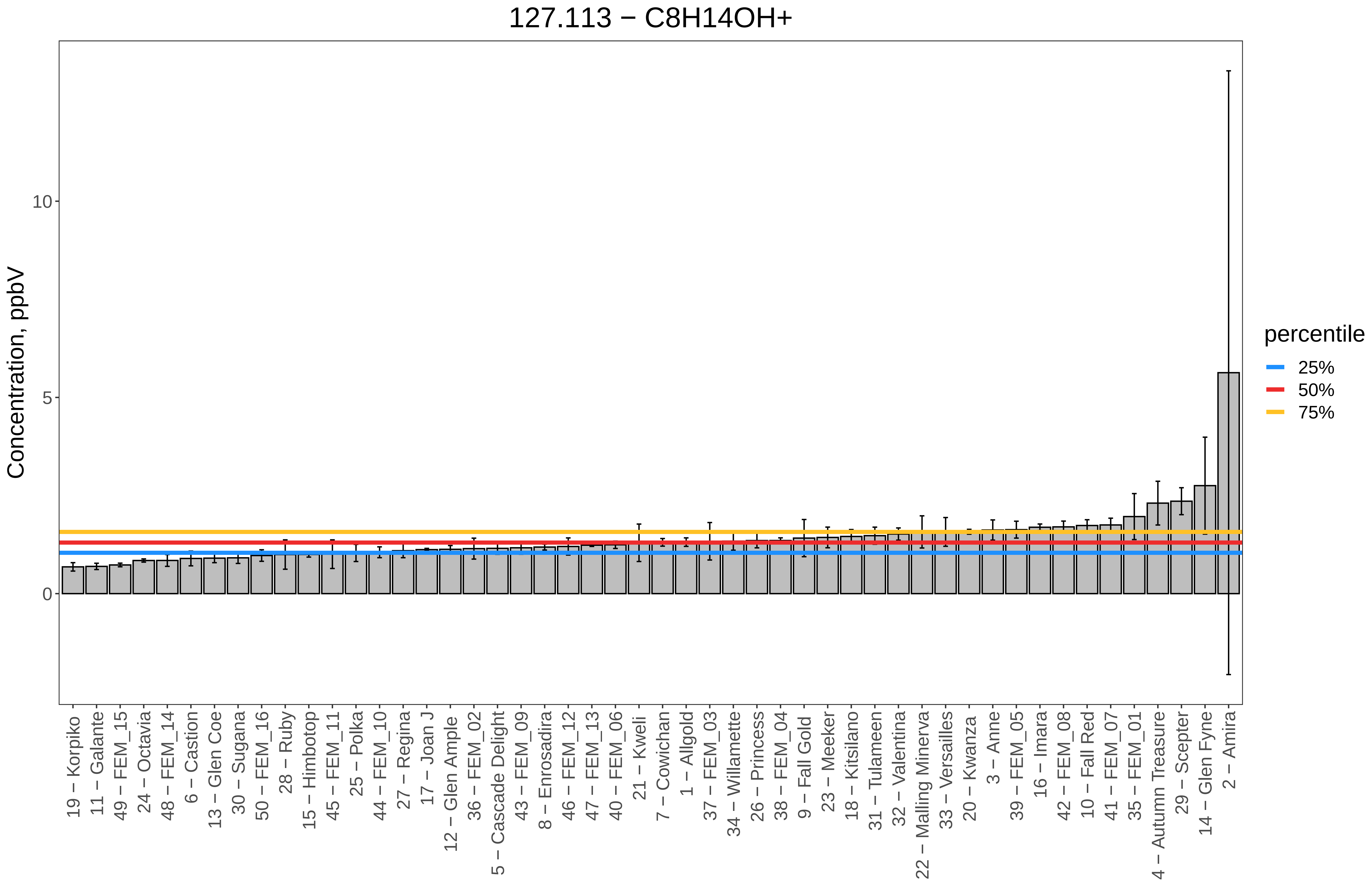

127.113 − C8H14OH+
Concentration, ppbV
0
5
10
percentile
25%
50%
75%
19 − Korpiko
11 − Galante
49 − FEM_15
24 − Octavia
48 − FEM_14
6 − Castion
13 − Glen Coe
30 − Sugana
50 − FEM_16
28 − Ruby
15 − Himbotop
45 − FEM_11
25 − Polka
44 − FEM_10
27 − Regina
17 − Joan J
12 − Glen Ample
36 − FEM_02
5 − Cascade Delight
43 − FEM_09
8 − Enrosadira
46 − FEM_12
47 − FEM_13
40 − FEM_06
21 − Kweli
7 − Cowichan
1 − Allgold
37 − FEM_03
34 − Willamette
26 − Princess
38 − FEM_04
9 − Fall Gold
23 − Meeker
18 − Kitsilano
31 − Tulameen
32 − Valentina
22 − Malling Minerva
33 − Versailles
20 − Kwanza
3 − Anne
39 − FEM_05
16 − Imara
42 − FEM_08
10 − Fall Red
41 − FEM_07
35 − FEM_01
4 − Autumn Treasure
29 − Scepter
14 − Glen Fyne
2 − Amira

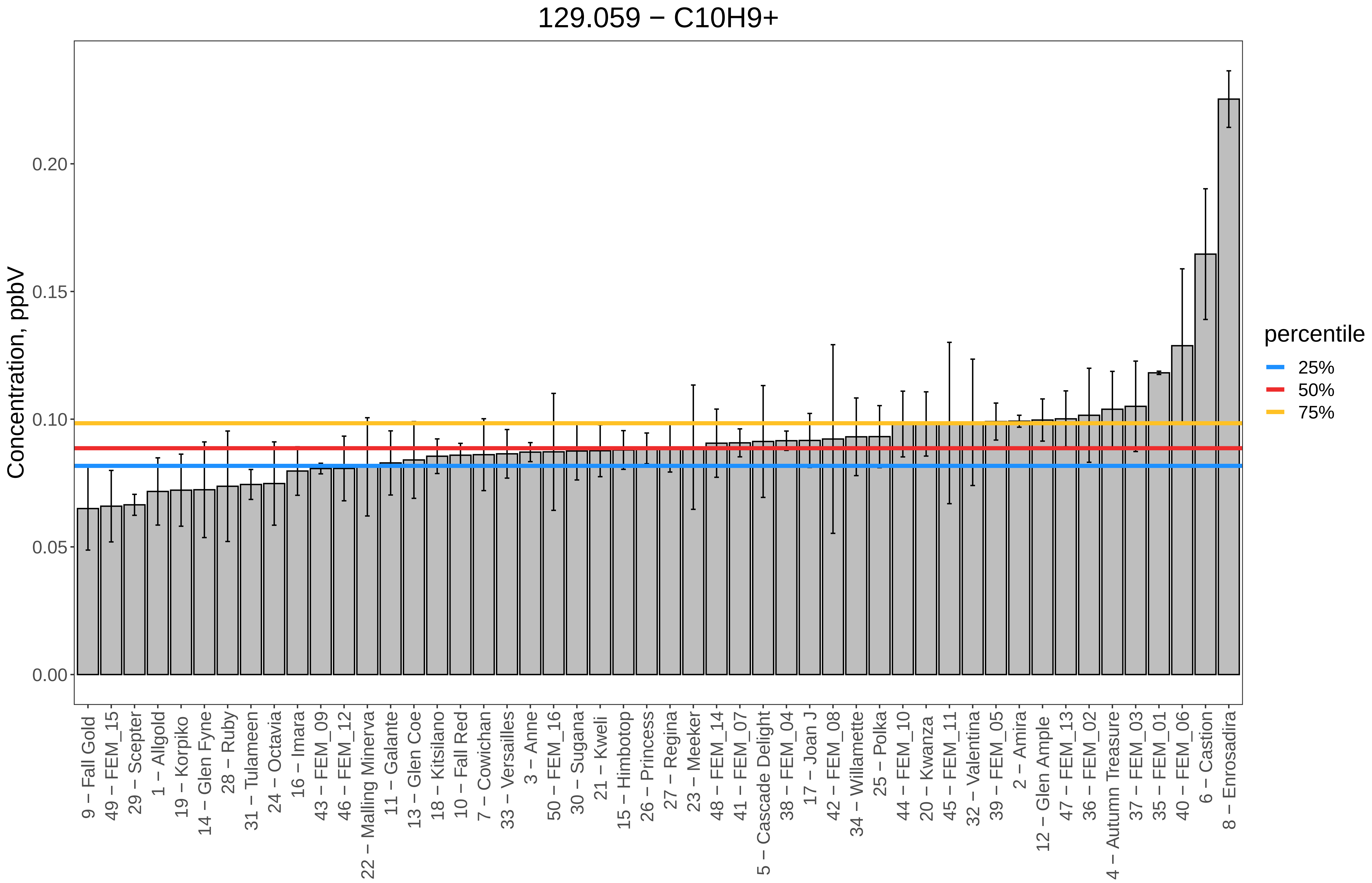
129.059 − C10H9+
Concentration, ppbV
0.00
0.05
0.10
0.15
0.20
percentile
25%
50%
75%
9 − Fall Gold
49 − FEM_15
29 − Scepter
1 − Allgold
19 − Korpiko
14 − Glen Fyne
28 − Ruby
31 − Tulameen
24 − Octavia
16 − Imara
43 − FEM_09
46 − FEM_12
22 − Malling Minerva
11 − Galante
13 − Glen Coe
18 − Kitsilano
10 − Fall Red
7 − Cowichan
33 − Versailles
3 − Anne
50 − FEM_16
30 − Sugana
21 − Kweli
15 − Himbotop
26 − Princess
27 − Regina
23 − Meeker
48 − FEM_14
41 − FEM_07
5 − Cascade Delight
38 − FEM_04
17 − Joan J
42 − FEM_08
34 − Willamette
25 − Polka
44 − FEM_10
20 − Kwanza
45 − FEM_11
32 − Valentina
39 − FEM_05
2 − Amira
12 − Glen Ample
47 − FEM_13
36 − FEM_02
4 − Autumn Treasure
37 − FEM_03
35 − FEM_01
40 − FEM_06
6 − Castion
8 − Enrosadira

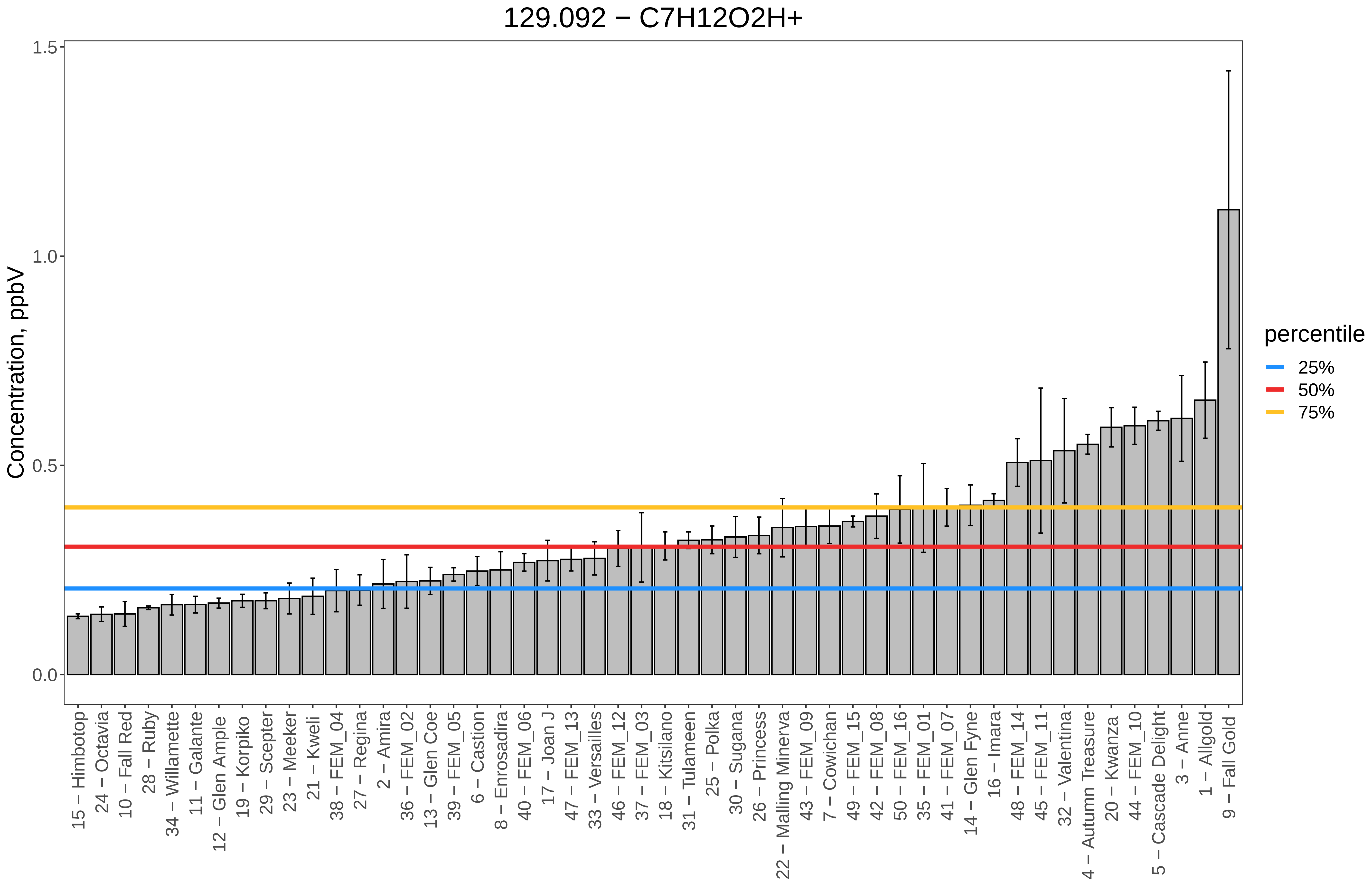

129.092 − C7H12O2H+
Concentration, ppbV
0.0
0.5
1.0
1.5
percentile
25%
50%
75%
15 − Himbotop
24 − Octavia
10 − Fall Red
28 − Ruby
34 − Willamette
11 − Galante
12 − Glen Ample
19 − Korpiko
29 − Scepter
23 − Meeker
21 − Kweli
38 − FEM_04
27 − Regina
2 − Amira
36 − FEM_02
13 − Glen Coe
39 − FEM_05
6 − Castion
8 − Enrosadira
40 − FEM_06
17 − Joan J
47 − FEM_13
33 − Versailles
46 − FEM_12
37 − FEM_03
18 − Kitsilano
31 − Tulameen
25 − Polka
30 − Sugana
26 − Princess
22 − Malling Minerva
43 − FEM_09
7 − Cowichan
49 − FEM_15
42 − FEM_08
50 − FEM_16
35 − FEM_01
41 − FEM_07
14 − Glen Fyne
16 − Imara
48 − FEM_14
45 − FEM_11
32 − Valentina
4 − Autumn Treasure
20 − Kwanza
44 − FEM_10
5 − Cascade Delight
3 − Anne
1 − Allgold
9 − Fall Gold

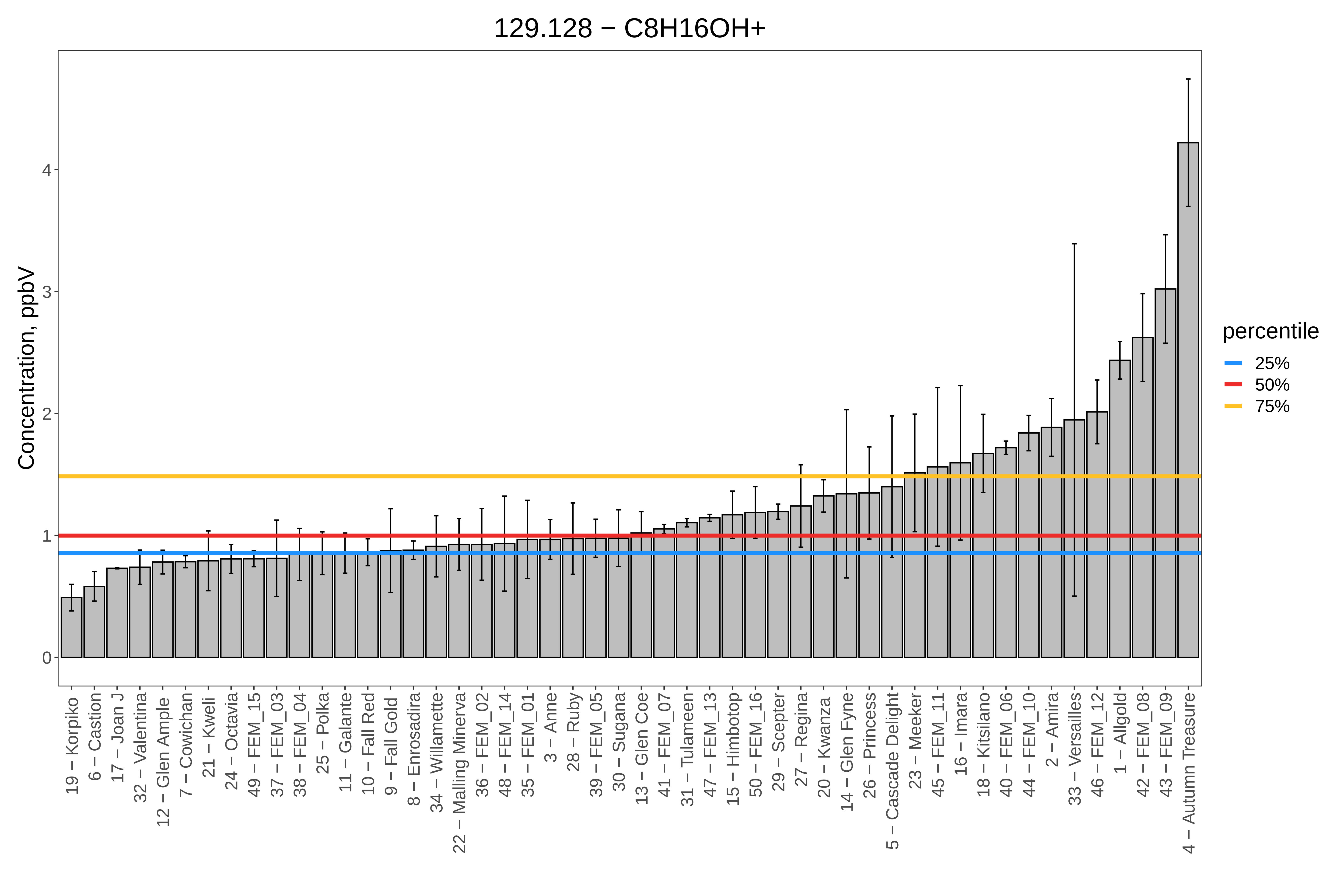

129.128 − C8H16OH+
Concentration, ppbV
0
1
2
3
4
percentile
25%
50%
75%
19 − Korpiko
6 − Castion
17 − Joan J
32 − Valentina
12 − Glen Ample
7 − Cowichan
21 − Kweli
24 − Octavia
49 − FEM_15
37 − FEM_03
38 − FEM_04
25 − Polka
11 − Galante
10 − Fall Red
9 − Fall Gold
8 − Enrosadira
34 − Willamette
22 − Malling Minerva
36 − FEM_02
48 − FEM_14
35 − FEM_01
3 − Anne
28 − Ruby
39 − FEM_05
30 − Sugana
13 − Glen Coe
41 − FEM_07
31 − Tulameen
47 − FEM_13
15 − Himbotop
50 − FEM_16
29 − Scepter
27 − Regina
20 − Kwanza
14 − Glen Fyne
26 − Princess
5 − Cascade Delight
23 − Meeker
45 − FEM_11
16 − Imara
18 − Kitsilano
40 − FEM_06
44 − FEM_10
2 − Amira
33 − Versailles
46 − FEM_12
1 − Allgold
42 − FEM_08
43 − FEM_09
4 − Autumn Treasure

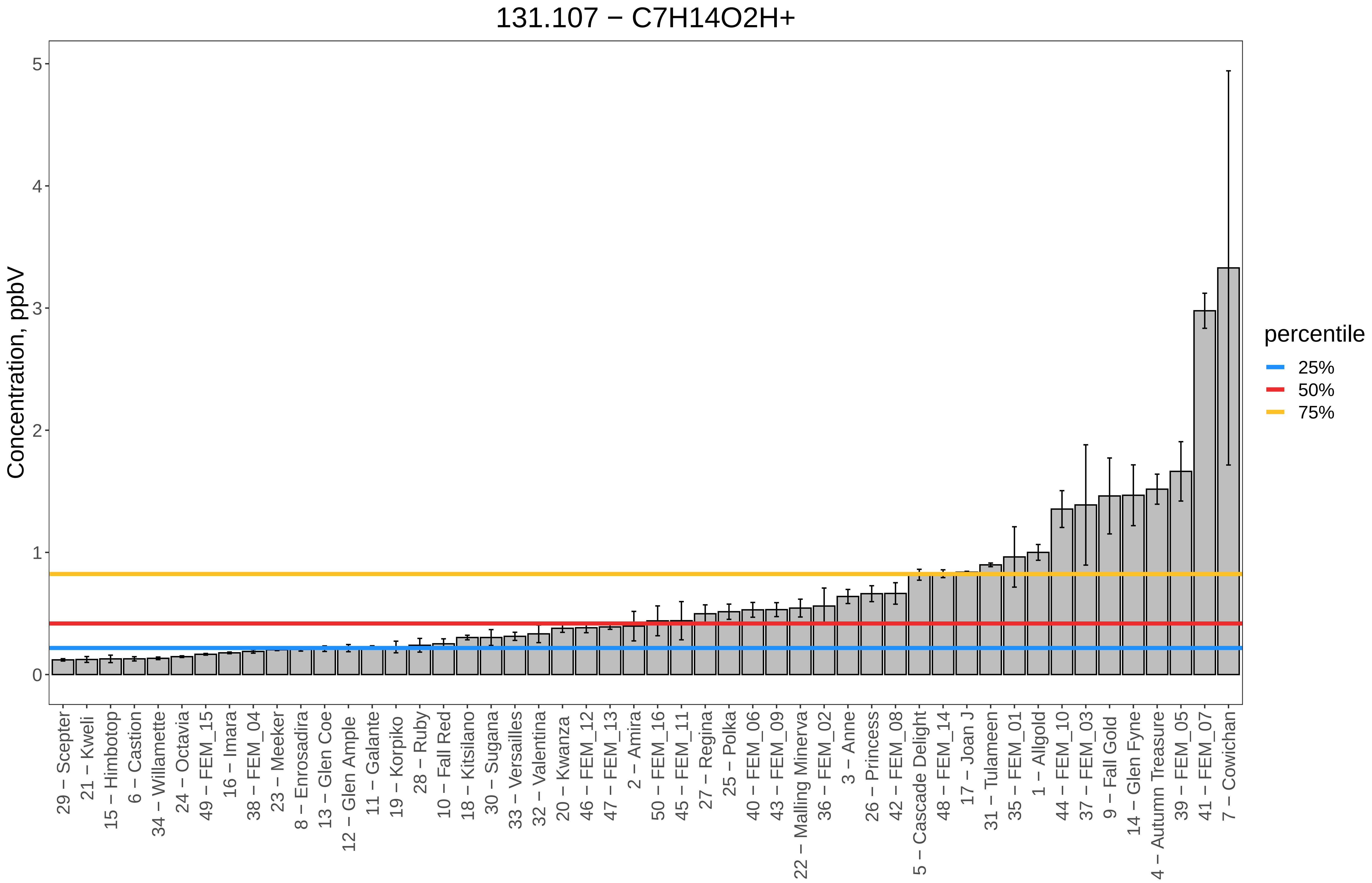

131.107 − C7H14O2H+
Concentration, ppbV
percentile
25%
50%
75%
29 − Scepter
21 − Kweli
15 − Himbotop
6 − Castion
34 − Willamette
24 − Octavia
49 − FEM_15
16 − Imara
38 − FEM_04
23 − Meeker
8 − Enrosadira
13 − Glen Coe
12 − Glen Ample
11 − Galante
19 − Korpiko
28 − Ruby
10 − Fall Red
18 − Kitsilano
30 − Sugana
33 − Versailles
32 − Valentina
20 − Kwanza
46 − FEM_12
47 − FEM_13
2 − Amira
50 − FEM_16
45 − FEM_11
27 − Regina
25 − Polka
40 − FEM_06
43 − FEM_09
22 − Malling Minerva
36 − FEM_02
3 − Anne
26 − Princess
42 − FEM_08
5 − Cascade Delight
48 − FEM_14
17 − Joan J
31 − Tulameen
35 − FEM_01
1 − Allgold
44 − FEM_10
37 − FEM_03
9 − Fall Gold
14 − Glen Fyne
4 − Autumn Treasure
39 − FEM_05
41 − FEM_07
7 − Cowichan

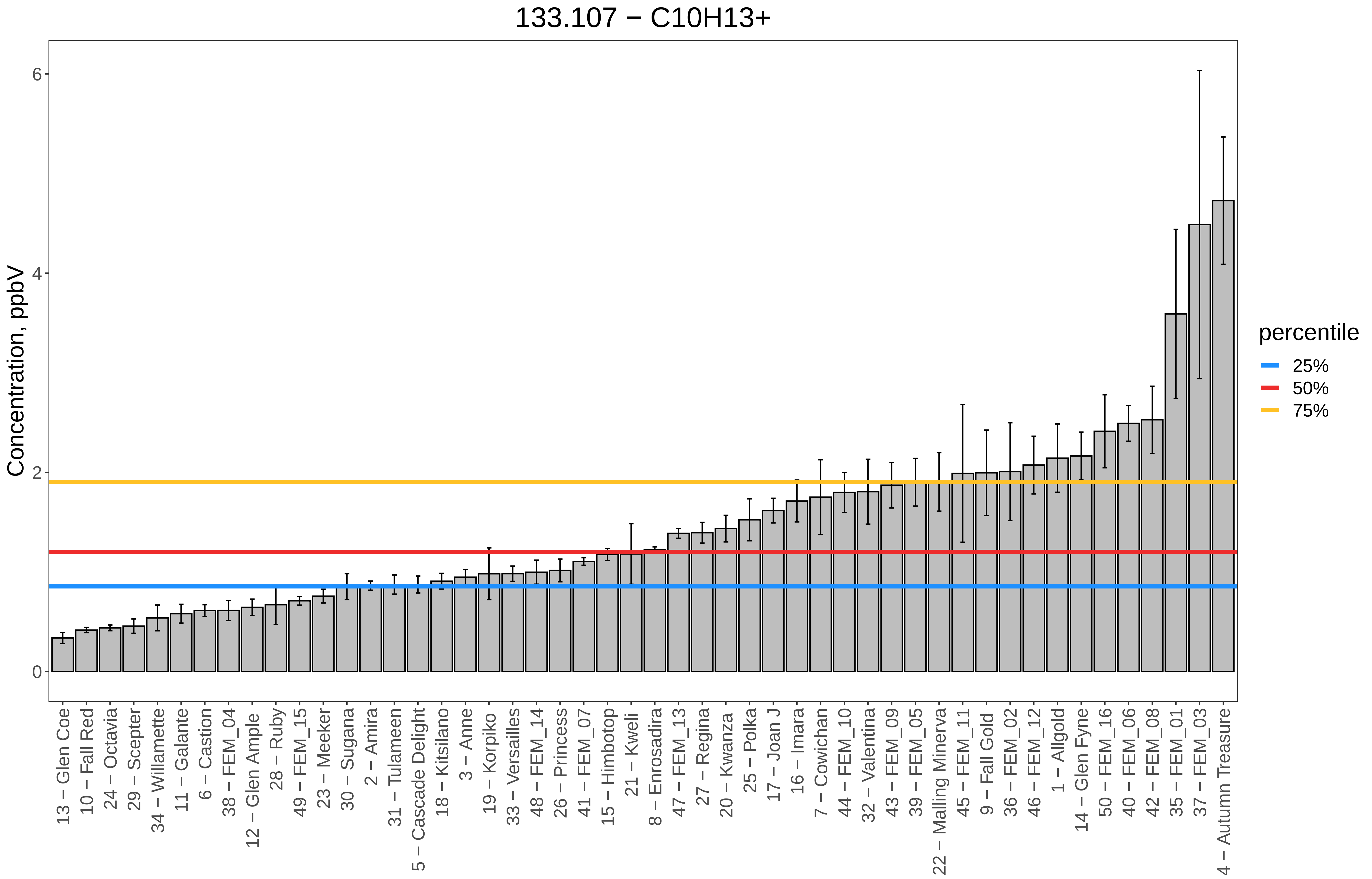
133.107 − C10H13+
Concentration, ppbV
0
2
4
6
percentile
25%
50%
75%
13 − Glen Coe
10 − Fall Red
24 − Octavia
29 − Scepter
34 − Willamette
11 − Galante
6 − Castion
38 − FEM_04
12 − Glen Ample
28 − Ruby
49 − FEM_15
23 − Meeker
30 − Sugana
2 − Amira
31 − Tulameen
5 − Cascade Delight
18 − Kitsilano
3 − Anne
19 − Korpiko
33 − Versailles
48 − FEM_14
26 − Princess
41 − FEM_07
15 − Himbotop
21 − Kweli
8 − Enrosadira
47 − FEM_13
27 − Regina
20 − Kwanza
25 − Polka
17 − Joan J
16 − Imara
7 − Cowichan
44 − FEM_10
32 − Valentina
43 − FEM_09
39 − FEM_05
22 − Malling Minerva
45 − FEM_11
9 − Fall Gold
36 − FEM_02
46 − FEM_12
1 − Allgold
14 − Glen Fyne
50 − FEM_16
40 − FEM_06
42 − FEM_08
35 − FEM_01
37 − FEM_03
4 − Autumn Treasure

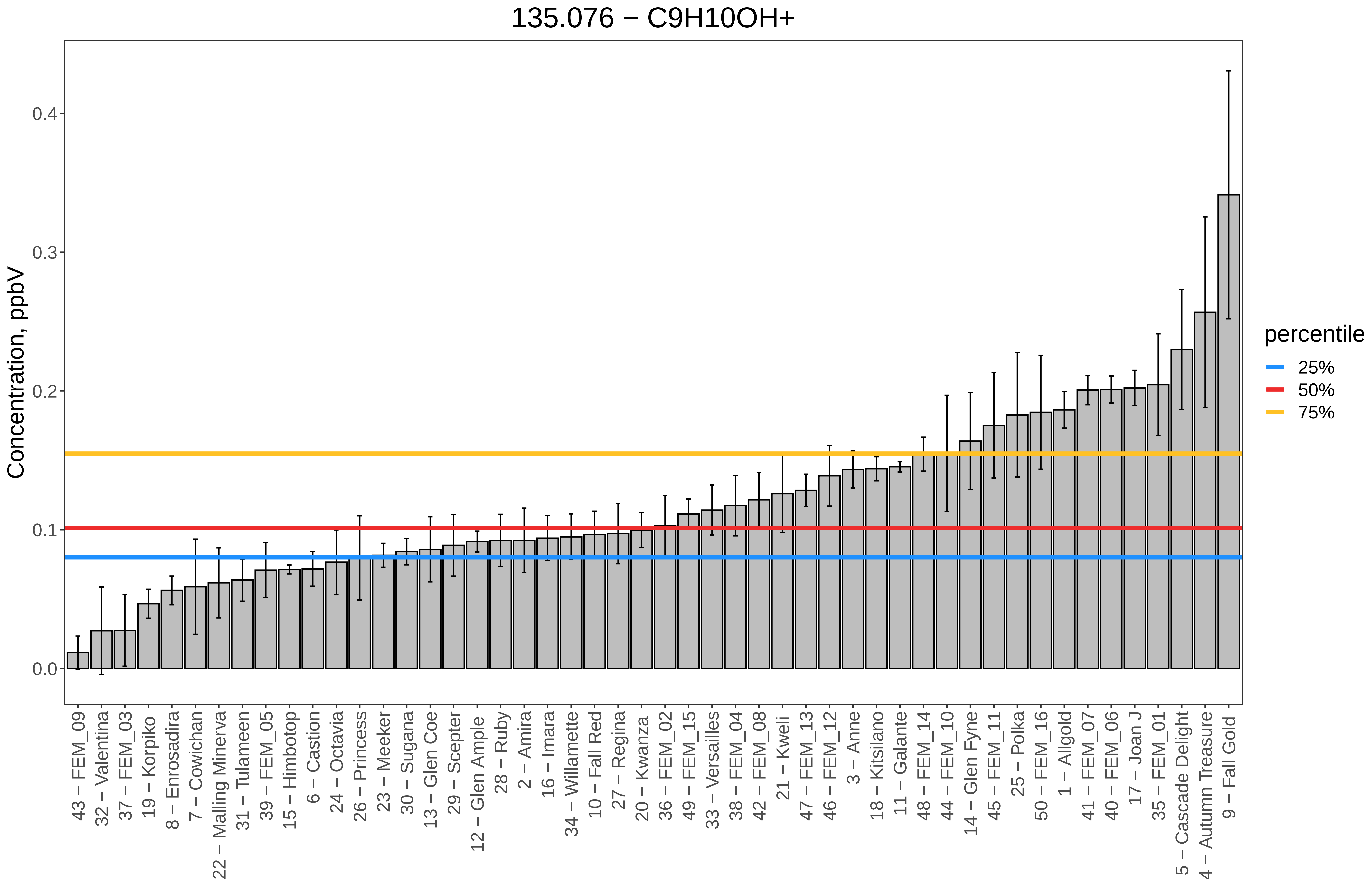
135.076 − C9H10OH+
Concentration, ppbV
0.0
0.1
0.2
0.3
0.4
percentile
25%
50%
75%
43 − FEM_09
32 − Valentina
37 − FEM_03
19 − Korpiko
8 − Enrosadira
7 − Cowichan
22 − Malling Minerva
31 − Tulameen
39 − FEM_05
15 − Himbotop
6 − Castion
24 − Octavia
26 − Princess
23 − Meeker
30 − Sugana
13 − Glen Coe
29 − Scepter
12 − Glen Ample
28 − Ruby
2 − Amira
16 − Imara
34 − Willamette
10 − Fall Red
27 − Regina
20 − Kwanza
36 − FEM_02
49 − FEM_15
33 − Versailles
38 − FEM_04
42 − FEM_08
21 − Kweli
47 − FEM_13
46 − FEM_12
3 − Anne
18 − Kitsilano
11 − Galante
48 − FEM_14
44 − FEM_10
14 − Glen Fyne
45 − FEM_11
25 − Polka
50 − FEM_16
1 − Allgold
41 − FEM_07
40 − FEM_06
17 − Joan J
35 − FEM_01
5 − Cascade Delight
4 − Autumn Treasure
9 − Fall Gold

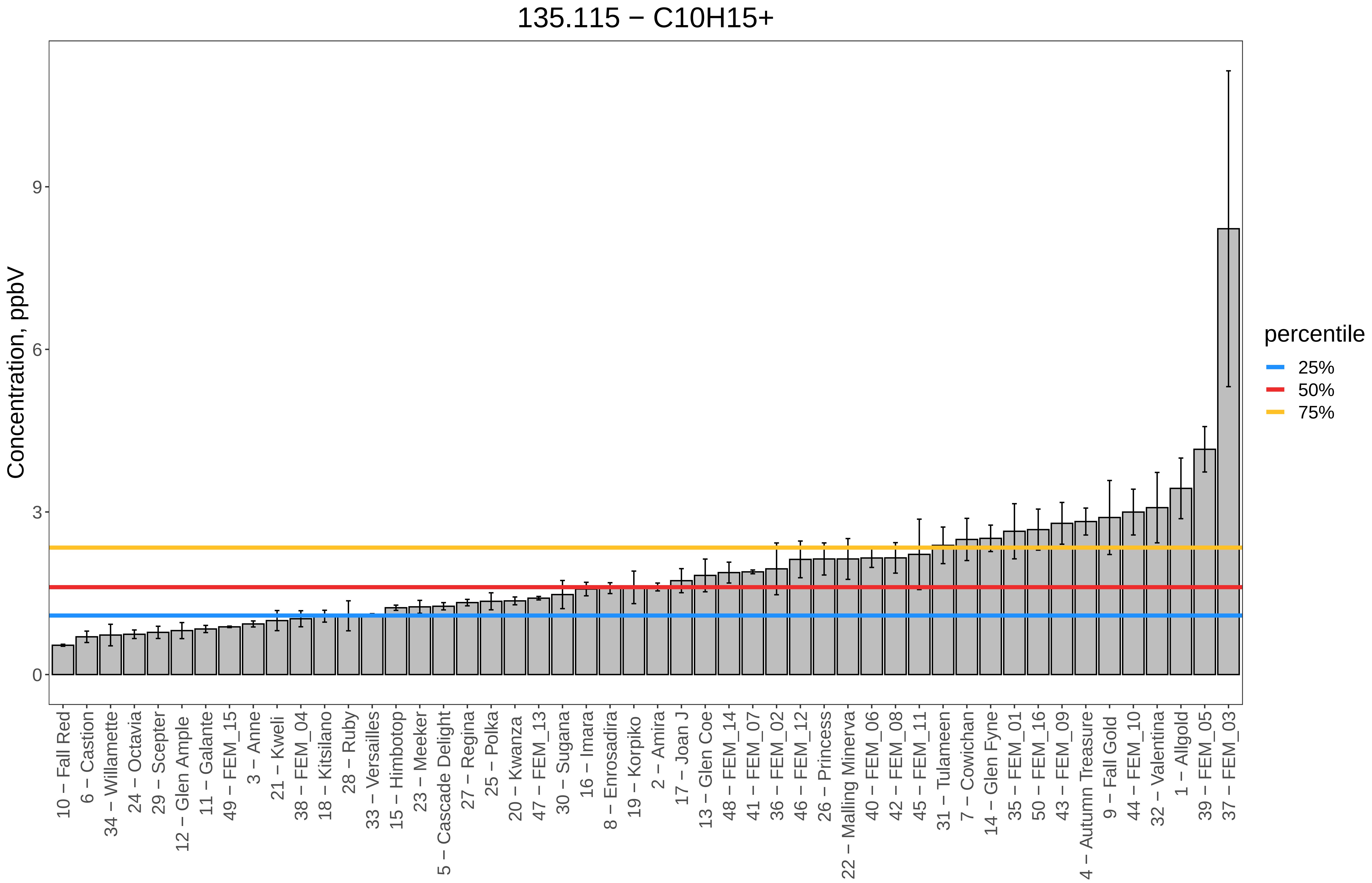
135.115 − C10H15+
Concentration, ppbV
0
3
6
9
percentile
25%
50%
75%
10 − Fall Red
6 − Castion
34 − Willamette
24 − Octavia
29 − Scepter
12 − Glen Ample
11 − Galante
49 − FEM_15
3 − Anne
21 − Kweli
38 − FEM_04
18 − Kitsilano
28 − Ruby
33 − Versailles
15 − Himbotop
23 − Meeker
5 − Cascade Delight
27 − Regina
25 − Polka
20 − Kwanza
47 − FEM_13
30 − Sugana
16 − Imara
8 − Enrosadira
19 − Korpiko
2 − Amira
17 − Joan J
13 − Glen Coe
48 − FEM_14
41 − FEM_07
36 − FEM_02
46 − FEM_12
26 − Princess
22 − Malling Minerva
40 − FEM_06
42 − FEM_08
45 − FEM_11
31 − Tulameen
7 − Cowichan
14 − Glen Fyne
35 − FEM_01
50 − FEM_16
43 − FEM_09
4 − Autumn Treasure
9 − Fall Gold
44 − FEM_10
32 − Valentina
1 − Allgold
39 − FEM_05
37 − FEM_03

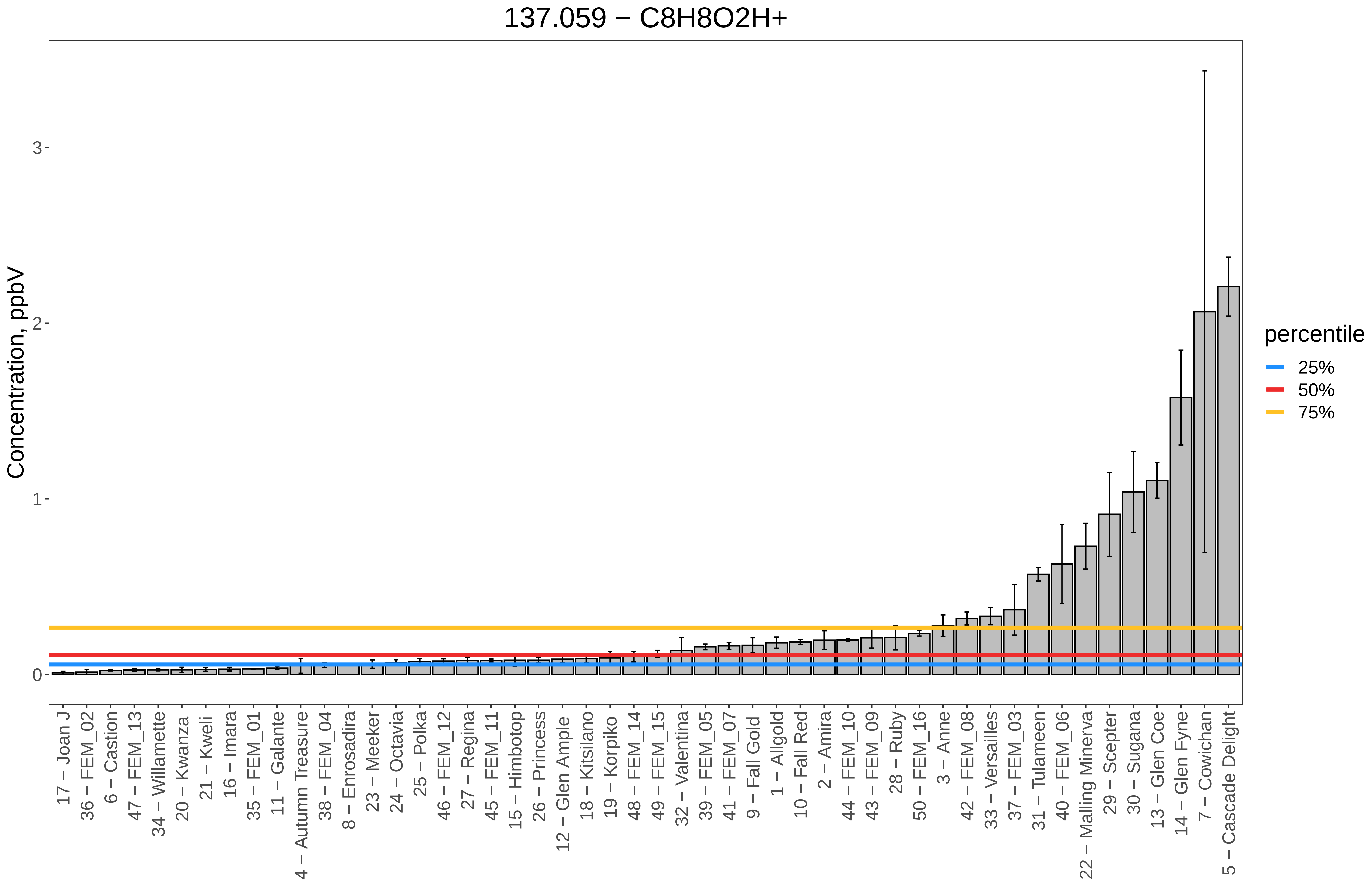
137.059 − C8H8O2H+
Concentration, ppbV
0
1
2
3
percentile
25%
50%
75%
17 − Joan J
36 − FEM_02
6 − Castion
47 − FEM_13
34 − Willamette
20 − Kwanza
21 − Kweli
16 − Imara
35 − FEM_01
11 − Galante
4 − Autumn Treasure
38 − FEM_04
8 − Enrosadira
23 − Meeker
24 − Octavia
25 − Polka
46 − FEM_12
27 − Regina
45 − FEM_11
15 − Himbotop
26 − Princess
12 − Glen Ample
18 − Kitsilano
19 − Korpiko
48 − FEM_14
49 − FEM_15
32 − Valentina
39 − FEM_05
41 − FEM_07
9 − Fall Gold
1 − Allgold
10 − Fall Red
2 − Amira
44 − FEM_10
43 − FEM_09
28 − Ruby
50 − FEM_16
3 − Anne
42 − FEM_08
33 − Versailles
37 − FEM_03
31 − Tulameen
40 − FEM_06
22 − Malling Minerva
29 − Scepter
30 − Sugana
13 − Glen Coe
14 − Glen Fyne
7 − Cowichan
5 − Cascade Delight

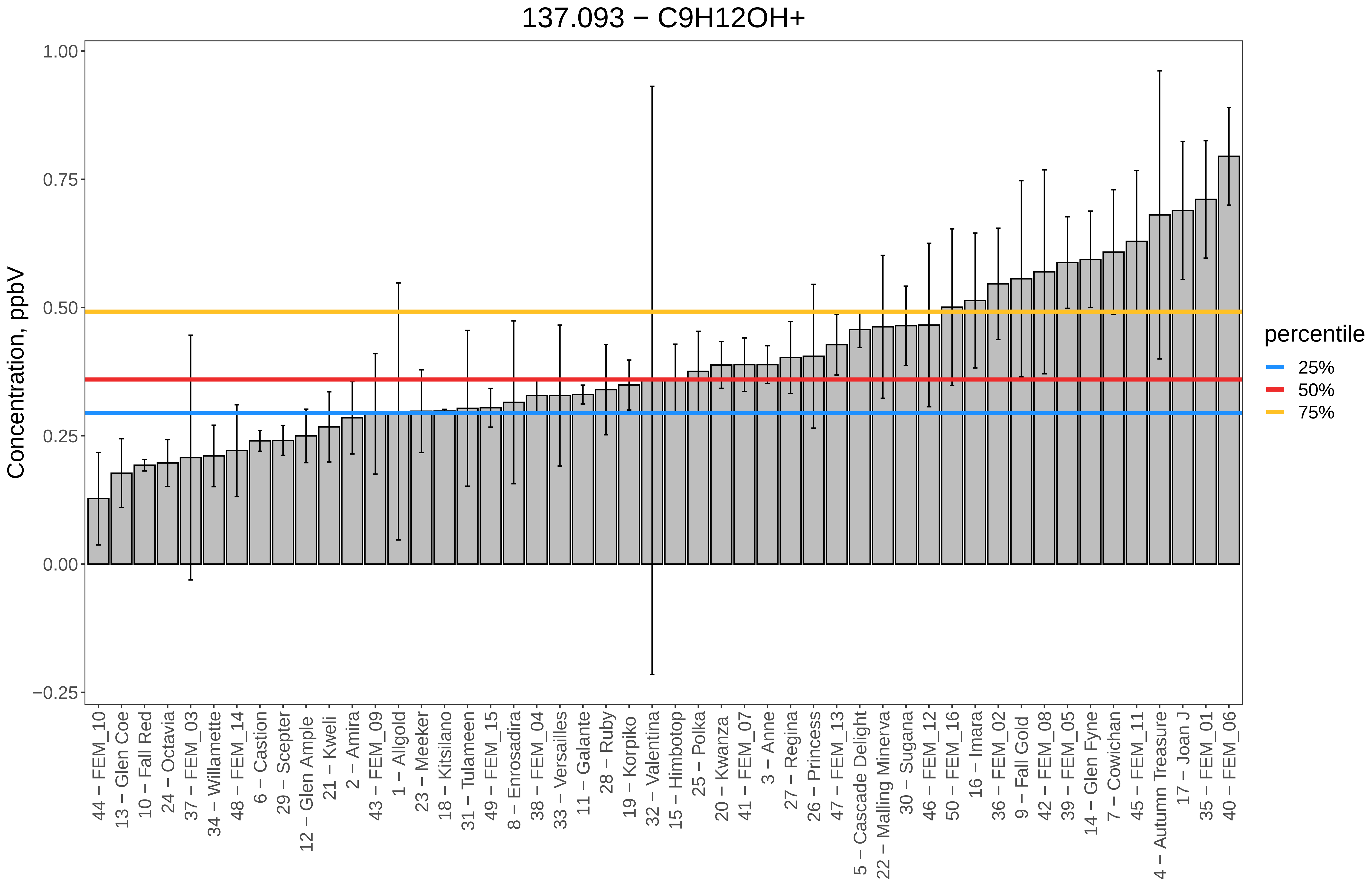

137.093 − C9H12OH+
Concentration, ppbV
1.00
0.75
0.50
0.25
0.00
−0.25
percentile
25%
50%
75%
44 − FEM_10
13 − Glen Coe
10 − Fall Red
24 − Octavia
37 − FEM_03
34 − Willamette
48 − FEM_14
6 − Castion
29 − Scepter
12 − Glen Ample
21 − Kweli
2 − Amira
43 − FEM_09
1 − Allgold
23 − Meeker
18 − Kitsilano
31 − Tulameen
49 − FEM_15
8 − Enrosadira
38 − FEM_04
33 − Versailles
11 − Galante
28 − Ruby
19 − Korpiko
32 − Valentina
15 − Himbotop
25 − Polka
20 − Kwanza
41 − FEM_07
3 − Anne
27 − Regina
26 − Princess
47 − FEM_13
5 − Cascade Delight
22 − Malling Minerva
30 − Sugana
46 − FEM_12
50 − FEM_16
16 − Imara
36 − FEM_02
9 − Fall Gold
42 − FEM_08
39 − FEM_05
14 − Glen Fyne
7 − Cowichan
45 − FEM_11
4 − Autumn Treasure
17 − Joan J
35 − FEM_01
40 − FEM_06

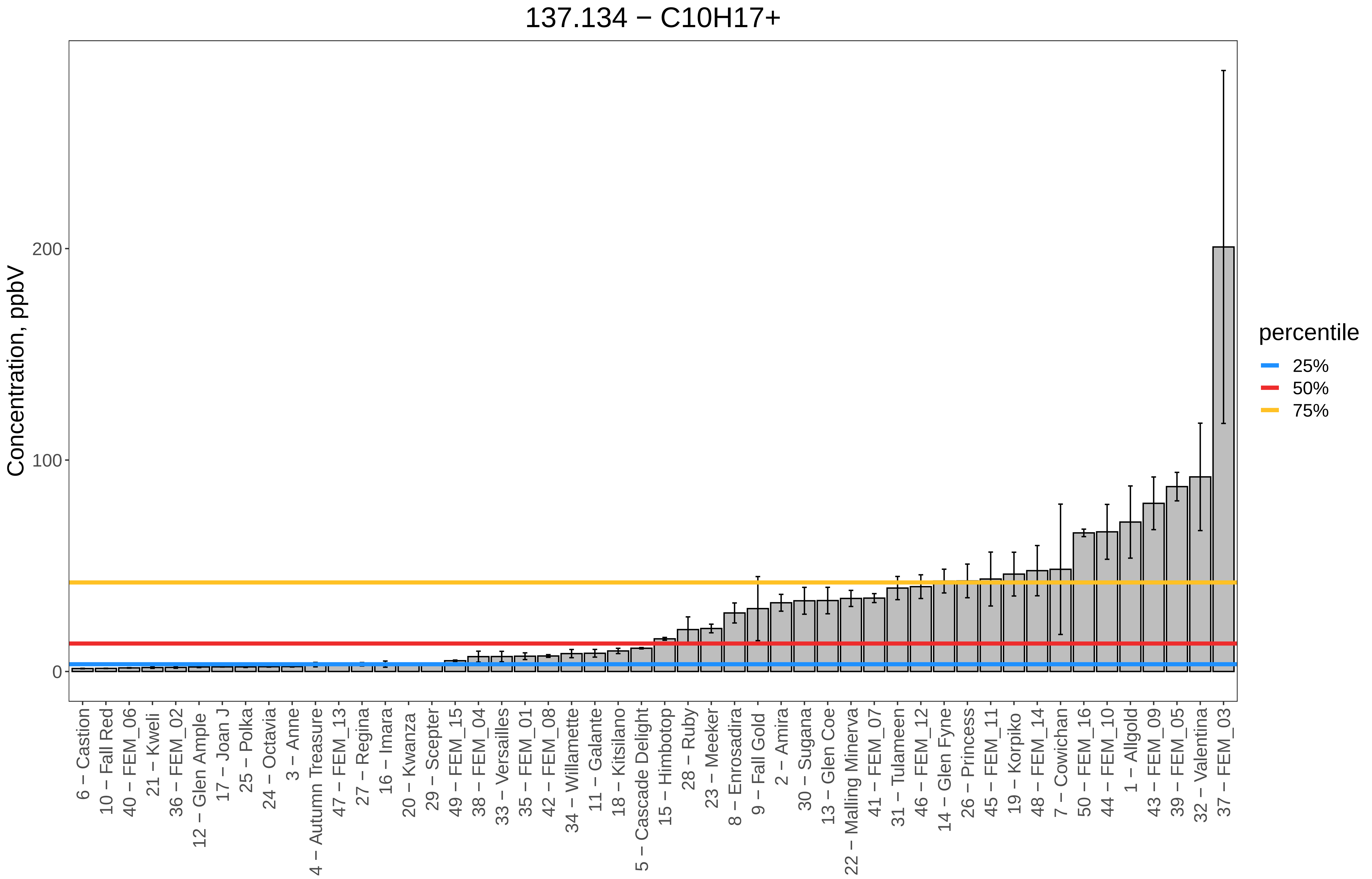

137.134 − C10H17+
Concentration, ppbV
0
100
200
percentile
25%
50%
75%
6 − Castion
10 − Fall Red
40 − FEM_06
21 − Kweli
36 − FEM_02
12 − Glen Ample
17 − Joan J
25 − Polka
24 − Octavia
3 − Anne
4 − Autumn Treasure
47 − FEM_13
27 − Regina
16 − Imara
20 − Kwanza
29 − Scepter
49 − FEM_15
38 − FEM_04
33 − Versailles
35 − FEM_01
42 − FEM_08
34 − Willamette
11 − Galante
18 − Kitsilano
5 − Cascade Delight
15 − Himbotop
28 − Ruby
23 − Meeker
8 − Enrosadira
9 − Fall Gold
2 − Amira
30 − Sugana
13 − Glen Coe
22 − Malling Minerva
41 − FEM_07
31 − Tulameen
46 − FEM_12
14 − Glen Fyne
26 − Princess
45 − FEM_11
19 − Korpiko
48 − FEM_14
7 − Cowichan
50 − FEM_16
44 − FEM_10
1 − Allgold
43 − FEM_09
39 − FEM_05
32 − Valentina
37 − FEM_03

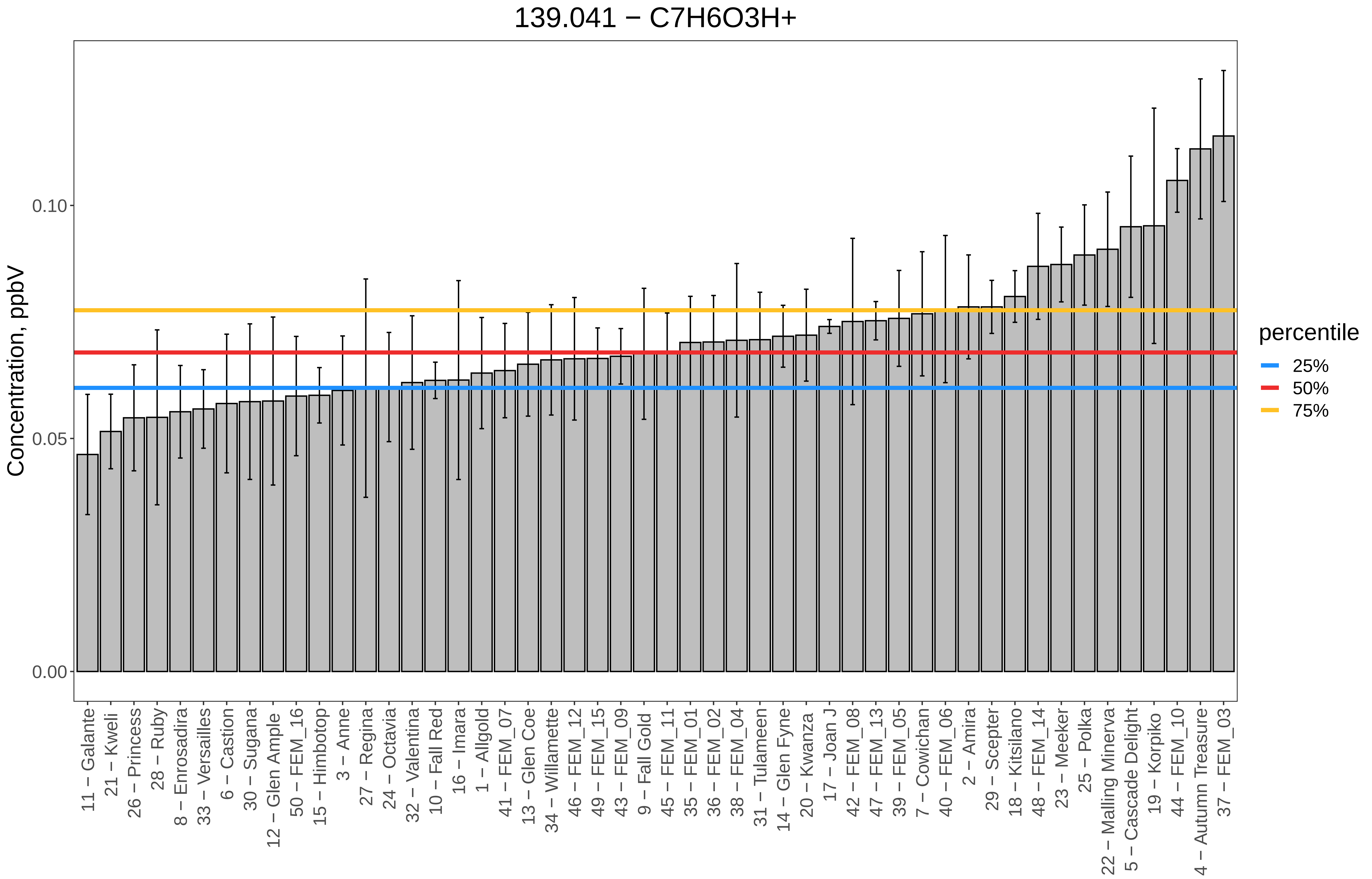
139.041 − C7H6O3H+
Concentration, ppbV
0.00
0.05
0.10
percentile
25%
50%
75%
11 − Galante
21 − Kweli
26 − Princess
28 − Ruby
8 − Enrosadira
33 − Versailles
6 − Castion
30 − Sugana
12 − Glen Ample
50 − FEM_16
15 − Himbotop
3 − Anne
27 − Regina
24 − Octavia
32 − Valentina
10 − Fall Red
16 − Imara
1 − Allgold
41 − FEM_07
13 − Glen Coe
34 − Willamette
46 − FEM_12
49 − FEM_15
43 − FEM_09
9 − Fall Gold
45 − FEM_11
35 − FEM_01
36 − FEM_02
38 − FEM_04
31 − Tulameen
14 − Glen Fyne
20 − Kwanza
17 − Joan J
42 − FEM_08
47 − FEM_13
39 − FEM_05
7 − Cowichan
40 − FEM_06
2 − Amira
29 − Scepter
18 − Kitsilano
48 − FEM_14
23 − Meeker
25 − Polka
22 − Malling Minerva
5 − Cascade Delight
19 − Korpiko
44 − FEM_10
4 − Autumn Treasure
37 − FEM_03

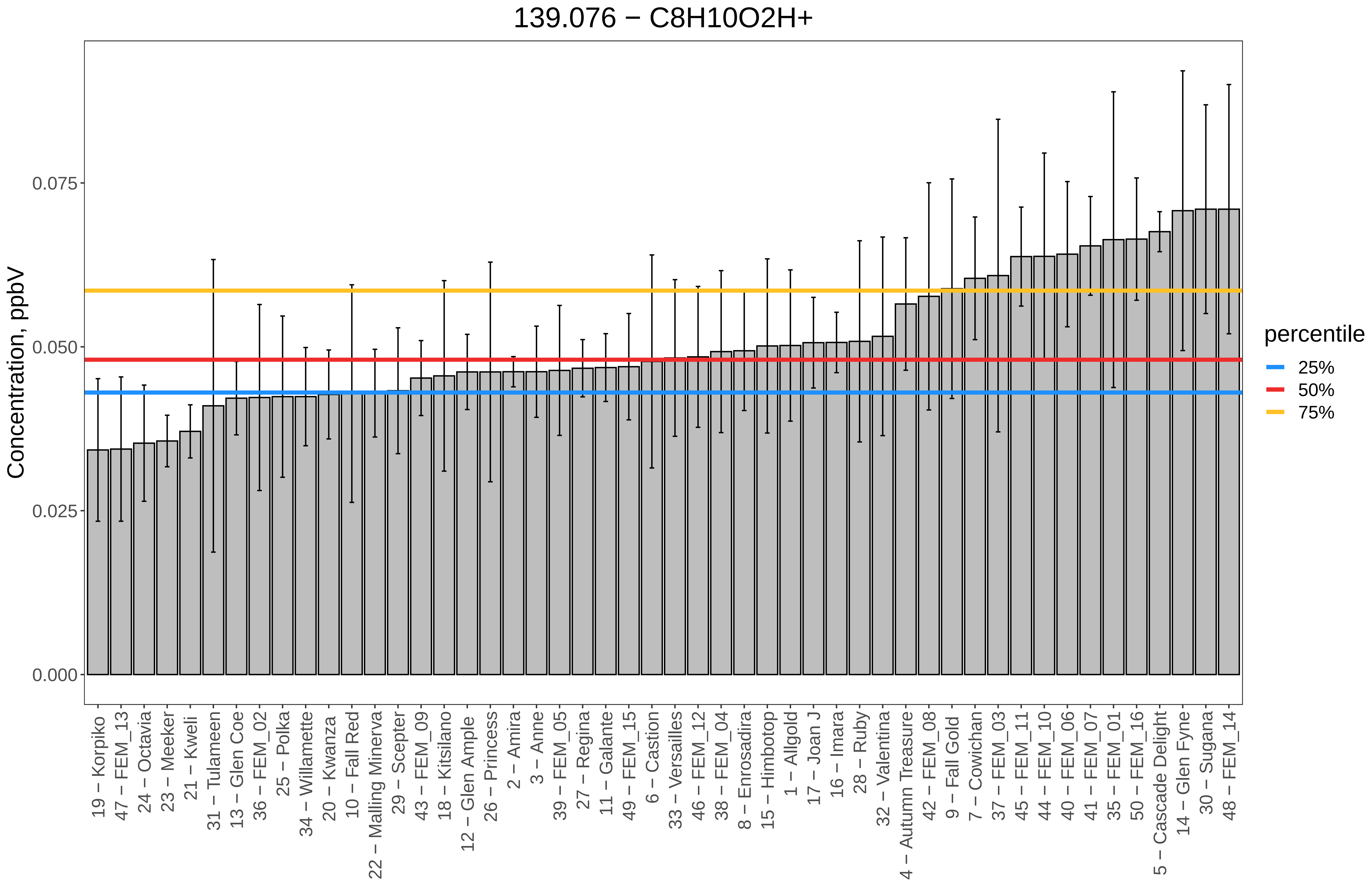

139.076 − C8H10O2H+
Concentration, ppbV
0.000
0.025
0.050
0.075
percentile
25%
50%
75%
19 − Korpiko
47 − FEM_13
24 − Octavia
23 − Meeker
21 − Kweli
31 − Tulameen
13 − Glen Coe
36 − FEM_02
25 − Polka
34 − Willamette
20 − Kwanza
10 − Fall Red
22 − Malling Minerva
29 − Scepter
43 − FEM_09
18 − Kitsilano
12 − Glen Ample
26 − Princess
2 − Amira
3 − Anne
39 − FEM_05
27 − Regina
11 − Galante
49 − FEM_15
6 − Castion
33 − Versailles
46 − FEM_12
38 − FEM_04
8 − Enrosadira
15 − Himbotop
1 − Allgold
17 − Joan J
16 − Imara
28 − Ruby
32 − Valentina
4 − Autumn Treasure
42 − FEM_08
9 − Fall Gold
7 − Cowichan
37 − FEM_03
45 − FEM_11
44 − FEM_10
40 − FEM_06
41 − FEM_07
35 − FEM_01
50 − FEM_16
5 − Cascade Delight
14 − Glen Fyne
30 − Sugana
48 − FEM_14

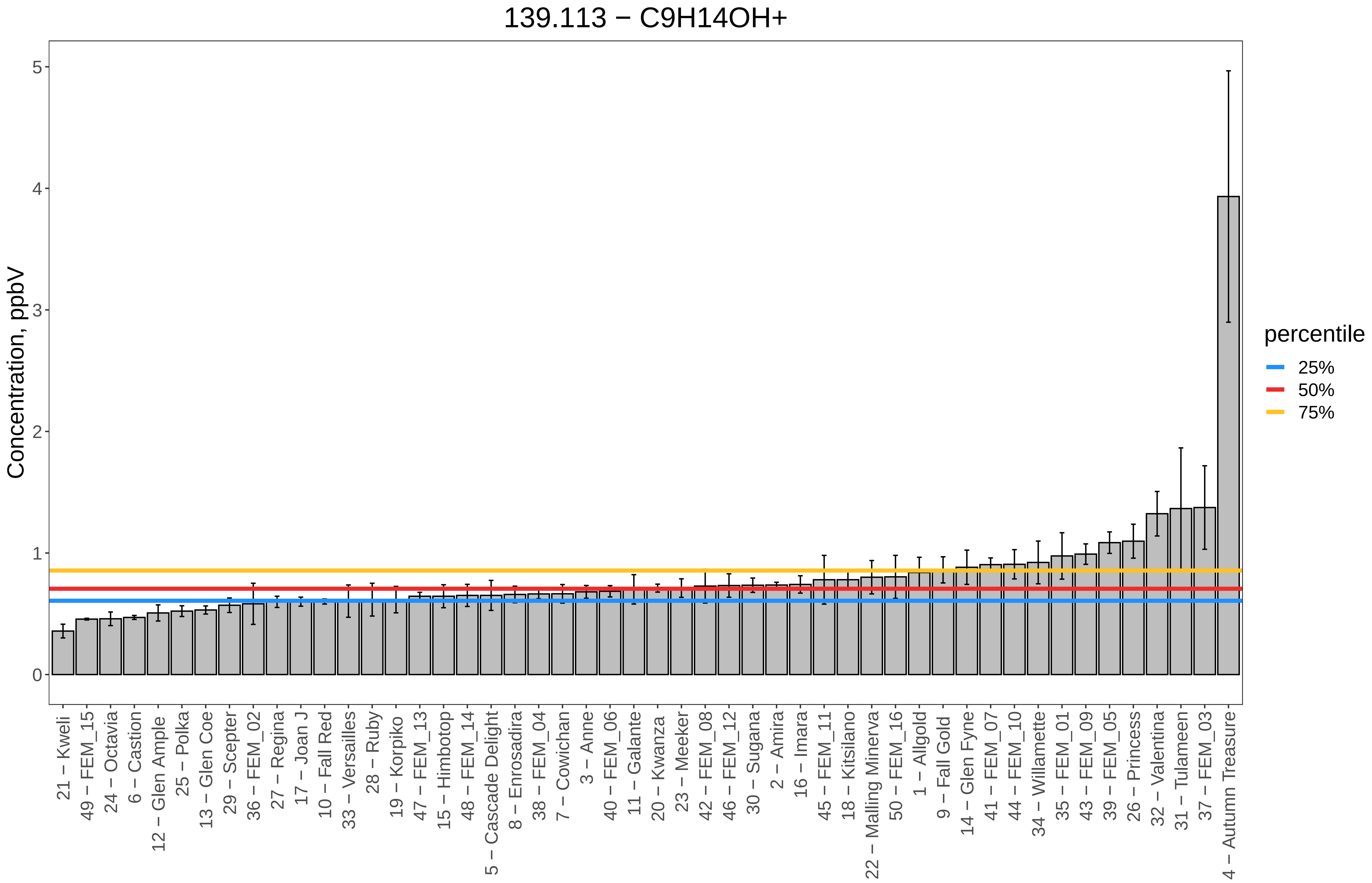

139.113 − C9H14OH+
Concentration, ppbV
0
1
2
3
4
5
percentile
25%
50%
75%
21 − Kweli
49 − FEM_15
24 − Octavia
6 − Castion
12 − Glen Ample
25 − Polka
13 − Glen Coe
29 − Scepter
36 − FEM_02
27 − Regina
17 − Joan J
10 − Fall Red
33 − Versailles
28 − Ruby
19 − Korpiko
47 − FEM_13
15 − Himbotop
48 − FEM_14
5 − Cascade Delight
8 − Enrosadira
38 − FEM_04
7 − Cowichan
3 − Anne
40 − FEM_06
11 − Galante
20 − Kwanza
23 − Meeker
42 − FEM_08
46 − FEM_12
30 − Sugana
2 − Amira
16 − Imara
45 − FEM_11
18 − Kitsilano
22 − Malling Minerva
50 − FEM_16
1 − Allgold
9 − Fall Gold
14 − Glen Fyne
41 − FEM_07
44 − FEM_10
34 − Willamette
35 − FEM_01
43 − FEM_09
39 − FEM_05
26 − Princess
32 − Valentina
31 − Tulameen
37 − FEM_03
4 − Autumn Treasure

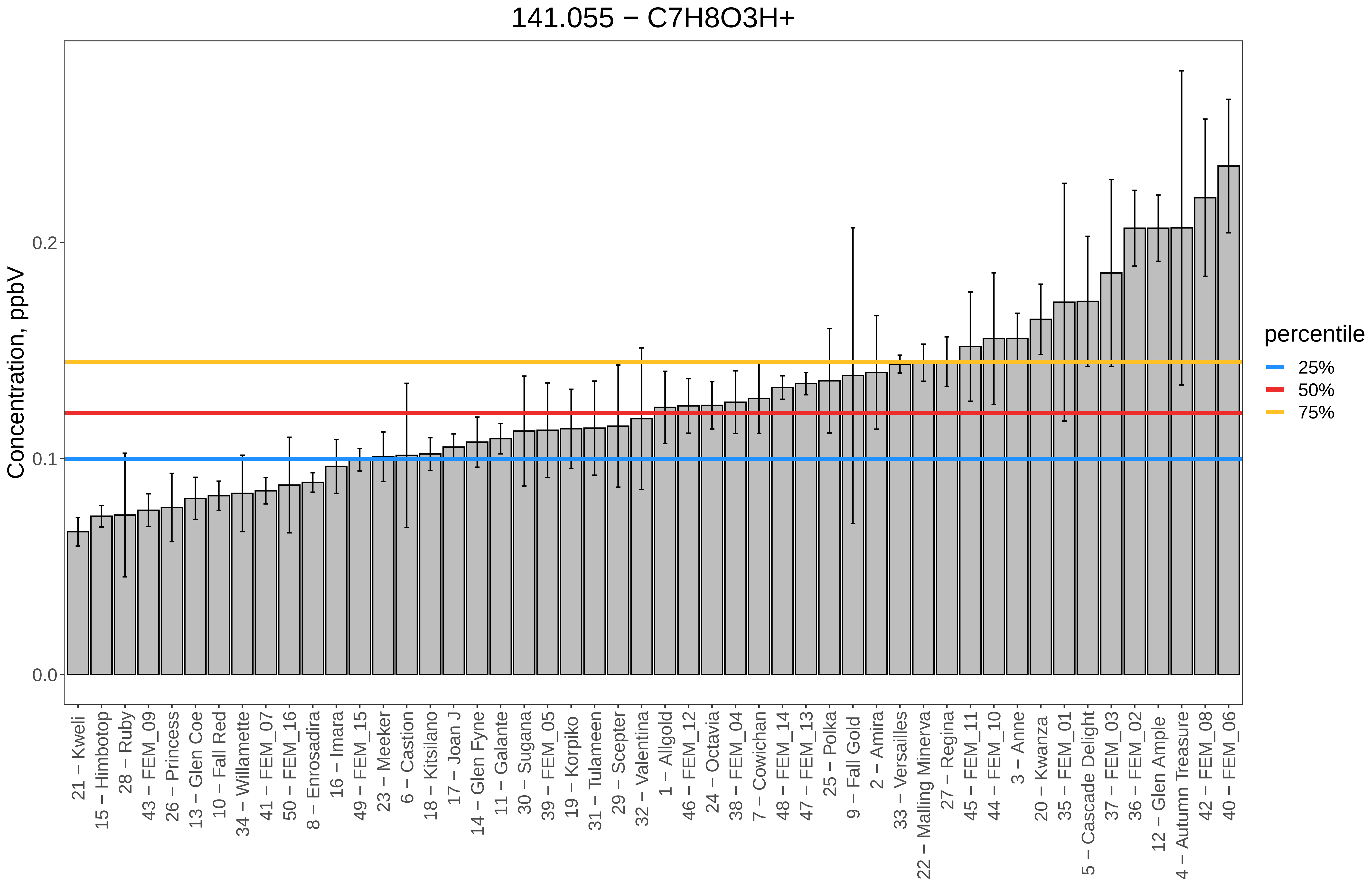

141.055 − C7H8O3H+
Concentration, ppbV
0.0
0.1
0.2
percentile
25%
50%
75%
21 − Kweli
15 − Himbotop
28 − Ruby
43 − FEM_09
26 − Princess
13 − Glen Coe
10 − Fall Red
34 − Willamette
41 − FEM_07
50 − FEM_16
8 − Enrosadira
16 − Imara
49 − FEM_15
23 − Meeker
6 − Castion
18 − Kitsilano
17 − Joan J
14 − Glen Fyne
11 − Galante
30 − Sugana
39 − FEM_05
19 − Korpiko
31 − Tulameen
29 − Scepter
32 − Valentina
1 − Allgold
46 − FEM_12
24 − Octavia
38 − FEM_04
7 − Cowichan
48 − FEM_14
47 − FEM_13
25 − Polka
9 − Fall Gold
2 − Amira
33 − Versailles
22 − Malling Minerva
27 − Regina
45 − FEM_11
44 − FEM_10
3 − Anne
20 − Kwanza
35 − FEM_01
5 − Cascade Delight
37 − FEM_03
36 − FEM_02
12 − Glen Ample
4 − Autumn Treasure
42 − FEM_08
40 − FEM_06

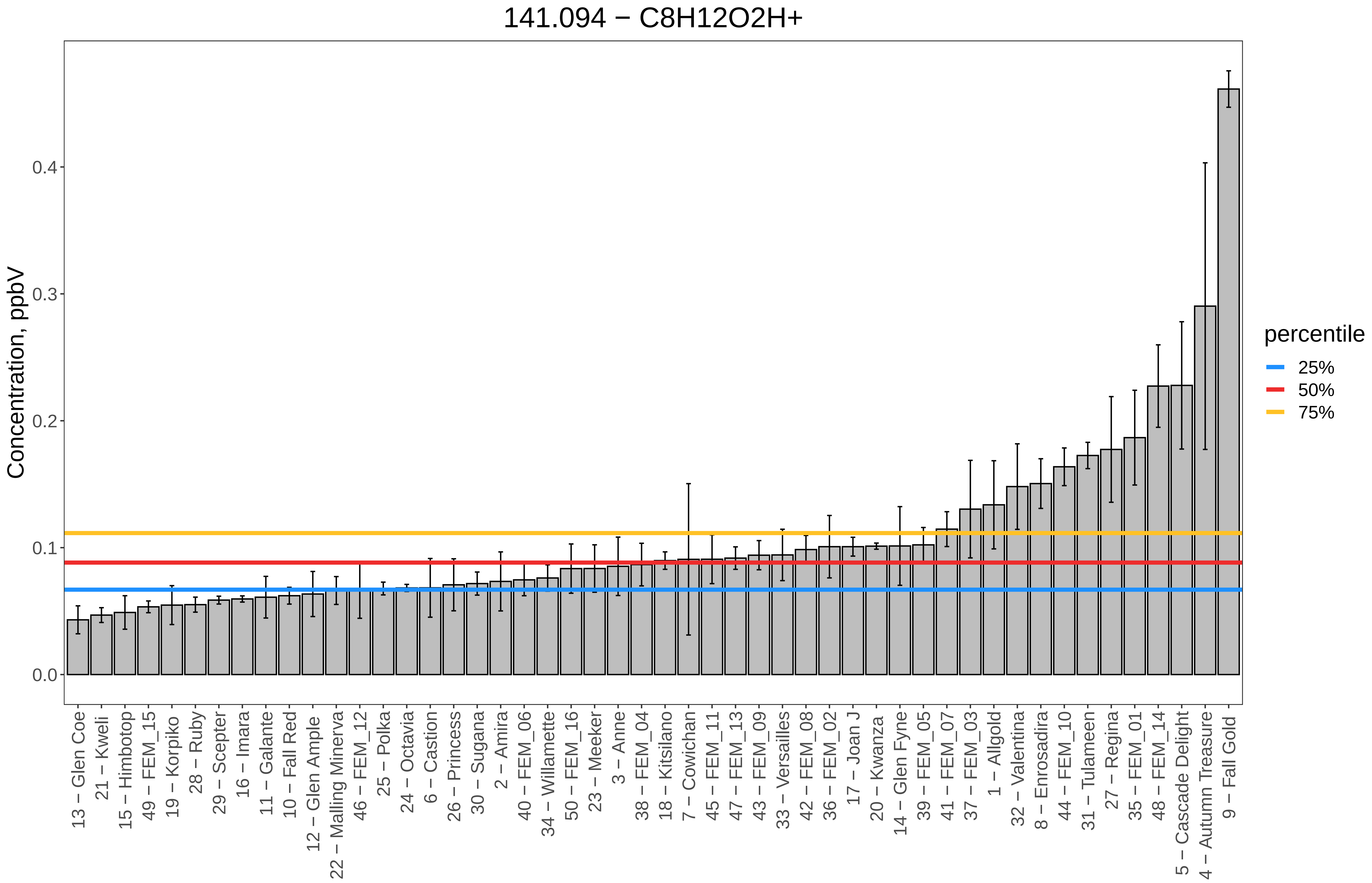

141.094 − C8H12O2H+
Concentration, ppbV
0.0
0.1
0.2
0.3
0.4
percentile
25%
50%
75%
13 − Glen Coe
21 − Kweli
15 − Himbotop
49 − FEM_15
19 − Korpiko
28 − Ruby
29 − Scepter
16 − Imara
11 − Galante
10 − Fall Red
12 − Glen Ample
22 − Malling Minerva
46 − FEM_12
25 − Polka
24 − Octavia
6 − Castion
26 − Princess
30 − Sugana
2 − Amira
40 − FEM_06
34 − Willamette
50 − FEM_16
23 − Meeker
3 − Anne
38 − FEM_04
18 − Kitsilano
7 − Cowichan
45 − FEM_11
47 − FEM_13
43 − FEM_09
33 − Versailles
42 − FEM_08
36 − FEM_02
17 − Joan J
20 − Kwanza
14 − Glen Fyne
39 − FEM_05
41 − FEM_07
37 − FEM_03
1 − Allgold
32 − Valentina
8 − Enrosadira
44 − FEM_10
31 − Tulameen
27 − Regina
35 − FEM_01
48 − FEM_14
5 − Cascade Delight
4 − Autumn Treasure
9 − Fall Gold

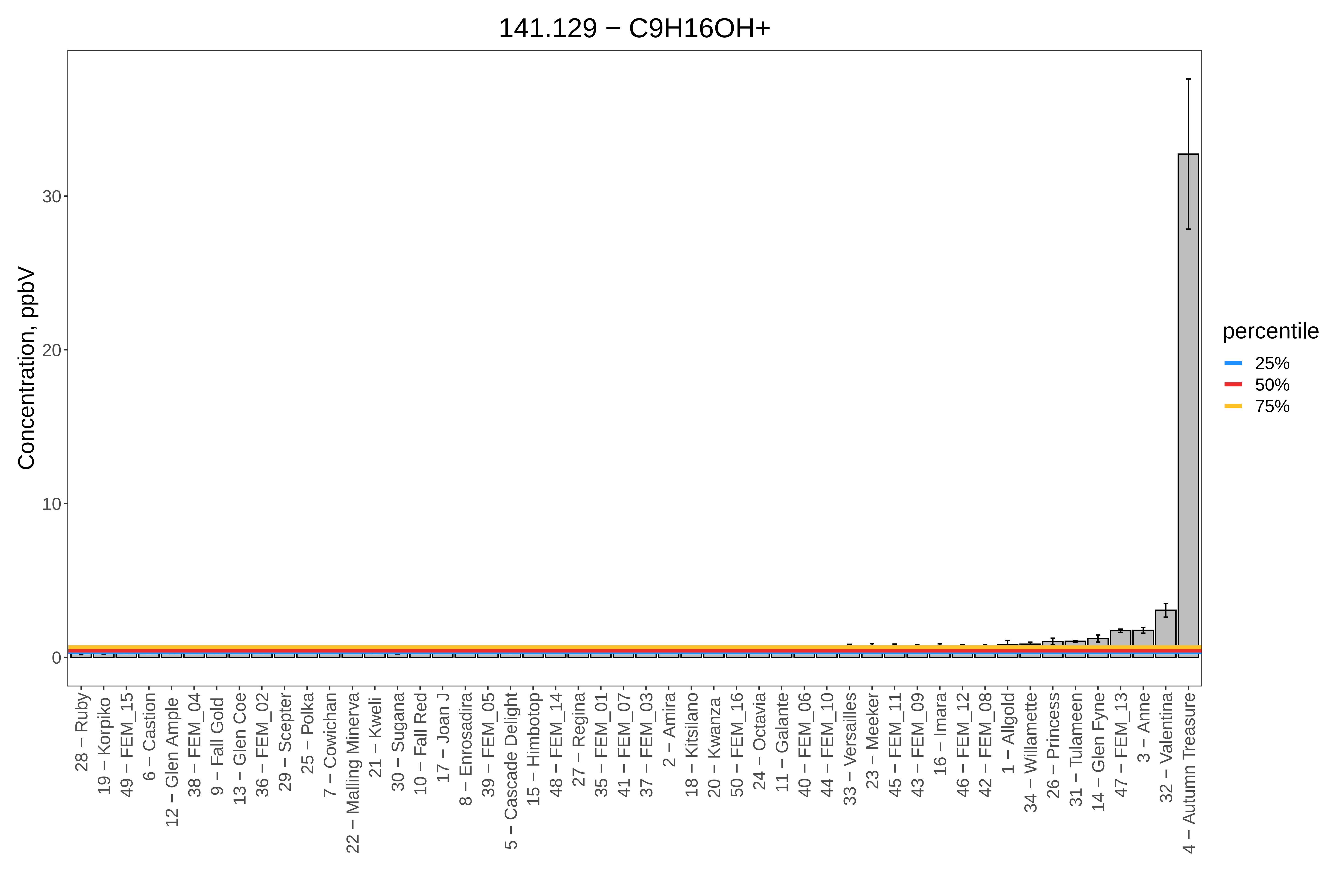
141.129 − C9H16OH+
Concentration, ppbV
0
10
20
30
percentile
25%
50%
75%
28 − Ruby
19 − Korpiko
49 − FEM_15
6 − Castion
12 − Glen Ample
38 − FEM_04
9 − Fall Gold
13 − Glen Coe
36 − FEM_02
29 − Scepter
25 − Polka
7 − Cowichan
22 − Malling Minerva
21 − Kweli
30 − Sugana
10 − Fall Red
17 − Joan J
8 − Enrosadira
39 − FEM_05
5 − Cascade Delight
15 − Himbotop
48 − FEM_14
27 − Regina
35 − FEM_01
41 − FEM_07
37 − FEM_03
2 − Amira
18 − Kitsilano
20 − Kwanza
50 − FEM_16
24 − Octavia
11 − Galante
40 − FEM_06
44 − FEM_10
33 − Versailles
23 − Meeker
45 − FEM_11
43 − FEM_09
16 − Imara
46 − FEM_12
42 − FEM_08
1 − Allgold
34 − Willamette
26 − Princess
31 − Tulameen
14 − Glen Fyne
47 − FEM_13
3 − Anne
32 − Valentina
4 − Autumn Treasure

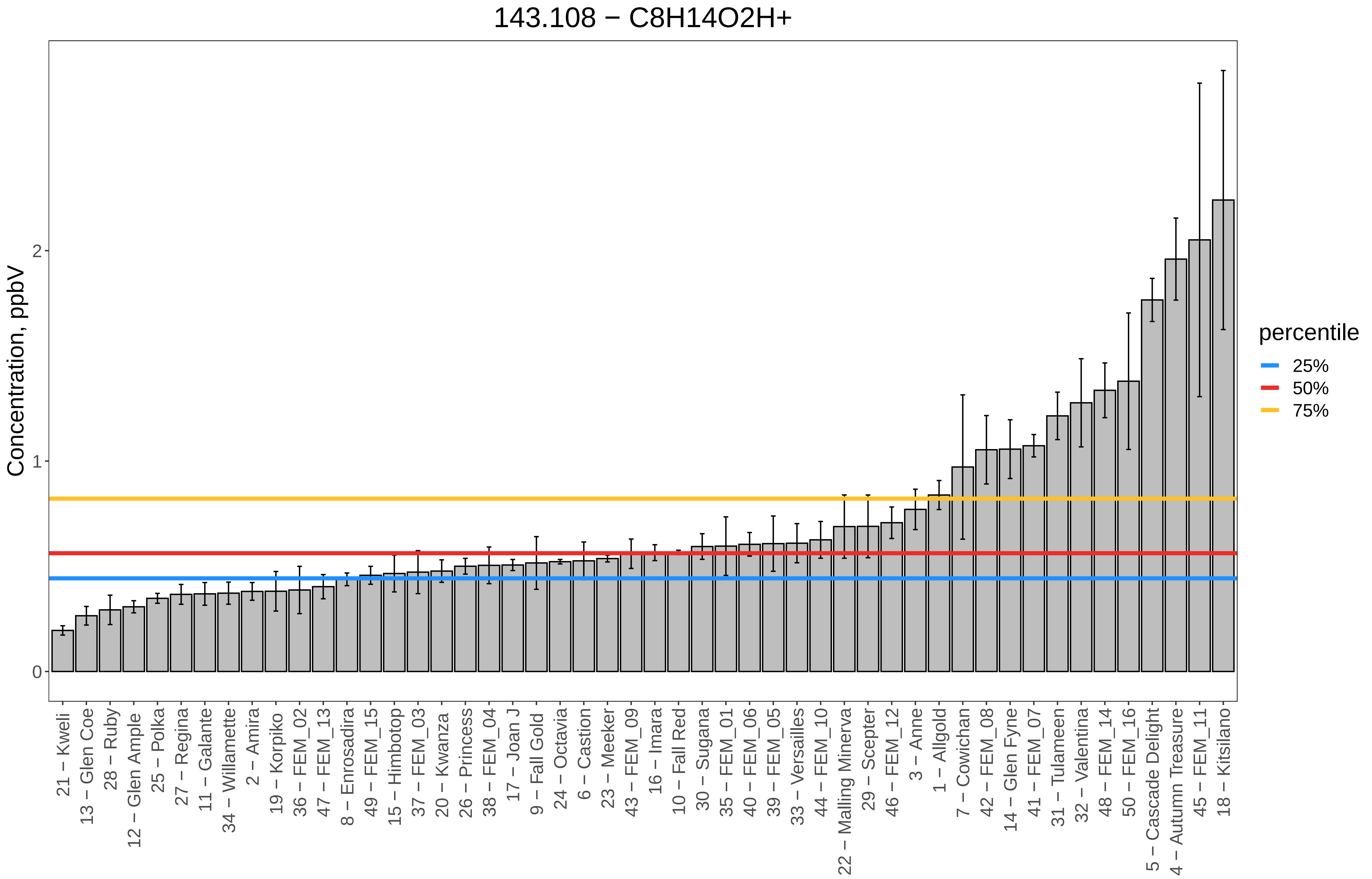
143.108 − C8H14O2H+
Concentration, ppbV
0
1
2
percentile
25%
50%
75%
21 − Kweli
13 − Glen Coe
28 − Ruby
12 − Glen Ample
25 − Polka
27 − Regina
11 − Galante
34 − Willamette
2 − Amira
19 − Korpiko
36 − FEM_02
47 − FEM_13
8 − Enrosadira
49 − FEM_15
15 − Himbotop
37 − FEM_03
20 − Kwanza
26 − Princess
38 − FEM_04
17 − Joan J
9 − Fall Gold
24 − Octavia
6 − Castion
23 − Meeker
43 − FEM_09
16 − Imara
10 − Fall Red
30 − Sugana
35 − FEM_01
40 − FEM_06
39 − FEM_05
33 − Versailles
44 − FEM_10
22 − Malling Minerva
29 − Scepter
46 − FEM_12
3 − Anne
1 − Allgold
7 − Cowichan
42 − FEM_08
14 − Glen Fyne
41 − FEM_07
31 − Tulameen
32 − Valentina
48 − FEM_14
50 − FEM_16
5 − Cascade Delight
4 − Autumn Treasure
45 − FEM_11
18 − Kitsilano

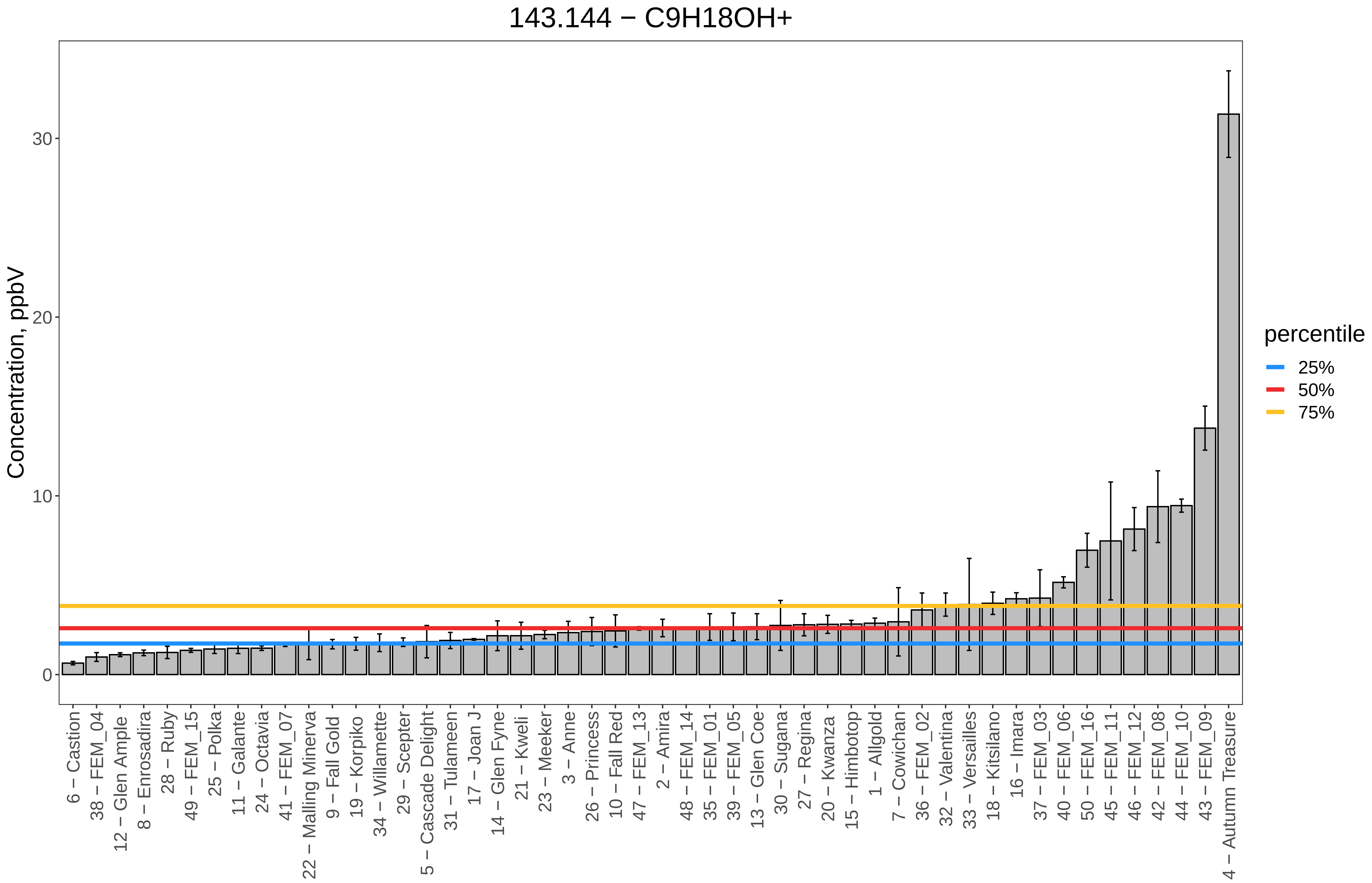

143.144 − C9H18OH+
Concentration, ppbV
0
10
20
30
percentile
25%
50%
75%
6 − Castion
38 − FEM_04
12 − Glen Ample
8 − Enrosadira
28 − Ruby
49 − FEM_15
25 − Polka
11 − Galante
24 − Octavia
41 − FEM_07
22 − Malling Minerva
9 − Fall Gold
19 − Korpiko
34 − Willamette
29 − Scepter
5 − Cascade Delight
31 − Tulameen
17 − Joan J
14 − Glen Fyne
21 − Kweli
23 − Meeker
3 − Anne
26 − Princess
10 − Fall Red
47 − FEM_13
2 − Amira
48 − FEM_14
35 − FEM_01
39 − FEM_05
13 − Glen Coe
30 − Sugana
27 − Regina
20 − Kwanza
15 − Himbotop
1 − Allgold
7 − Cowichan
36 − FEM_02
32 − Valentina
33 − Versailles
18 − Kitsilano
16 − Imara
37 − FEM_03
40 − FEM_06
50 − FEM_16
45 − FEM_11
46 − FEM_12
42 − FEM_08
44 − FEM_10
43 − FEM_09
4 − Autumn Treasure

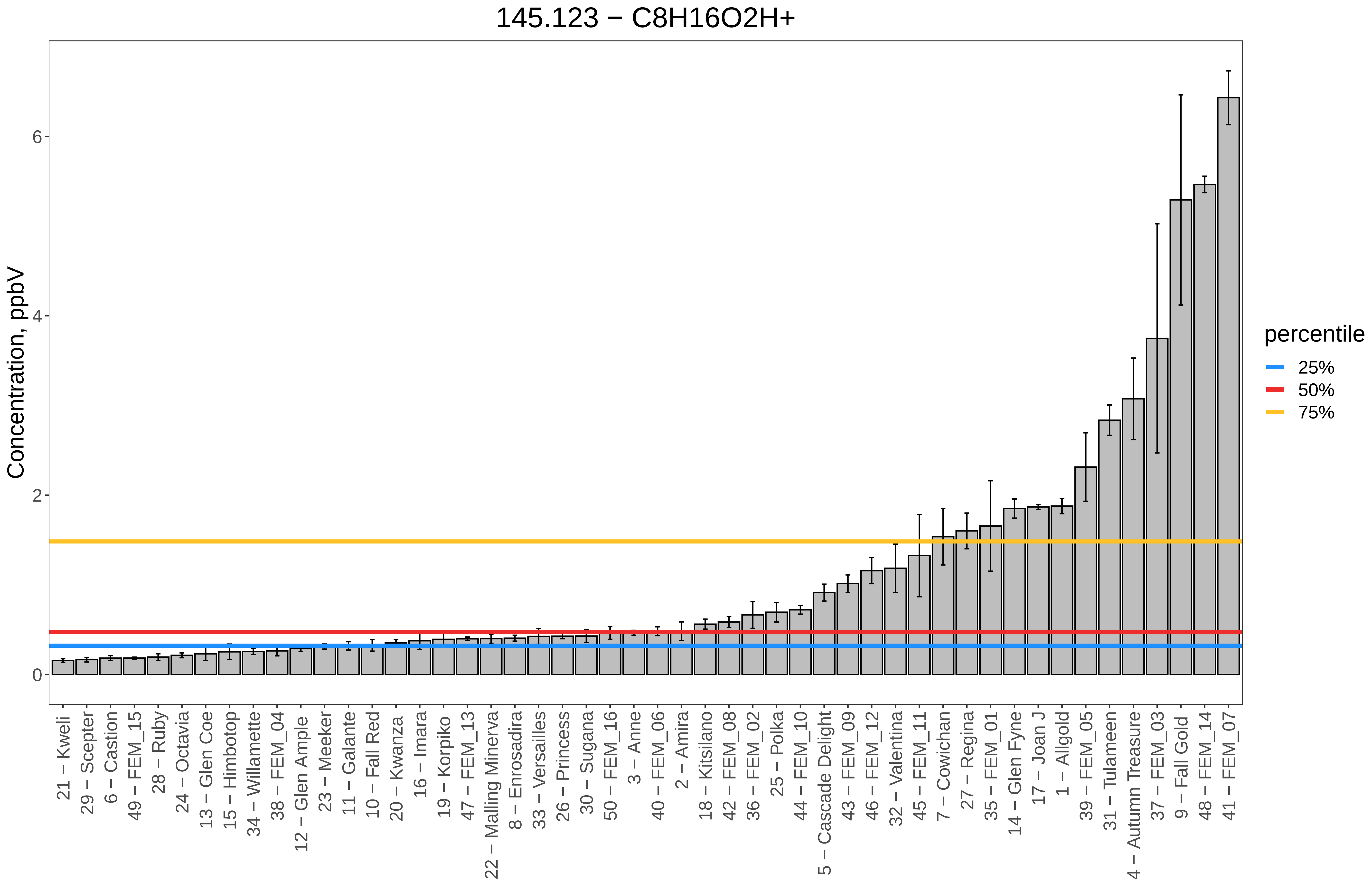
145.123 − C8H16O2H+
Concentration, ppbV
0
2
4
6
percentile
25%
50%
75%
21 − Kweli
29 − Scepter
6 − Castion
49 − FEM_15
28 − Ruby
24 − Octavia
13 − Glen Coe
15 − Himbotop
34 − Willamette
38 − FEM_04
12 − Glen Ample
23 − Meeker
11 − Galante
10 − Fall Red
20 − Kwanza
16 − Imara
19 − Korpiko
47 − FEM_13
22 − Malling Minerva
8 − Enrosadira
33 − Versailles
26 − Princess
30 − Sugana
50 − FEM_16
3 − Anne
40 − FEM_06
2 − Amira
18 − Kitsilano
42 − FEM_08
36 − FEM_02
25 − Polka
44 − FEM_10
5 − Cascade Delight
43 − FEM_09
46 − FEM_12
32 − Valentina
45 − FEM_11
7 − Cowichan
27 − Regina
35 − FEM_01
14 − Glen Fyne
17 − Joan J
1 − Allgold
39 − FEM_05
31 − Tulameen
4 − Autumn Treasure
37 − FEM_03
9 − Fall Gold
48 − FEM_14
41 − FEM_07

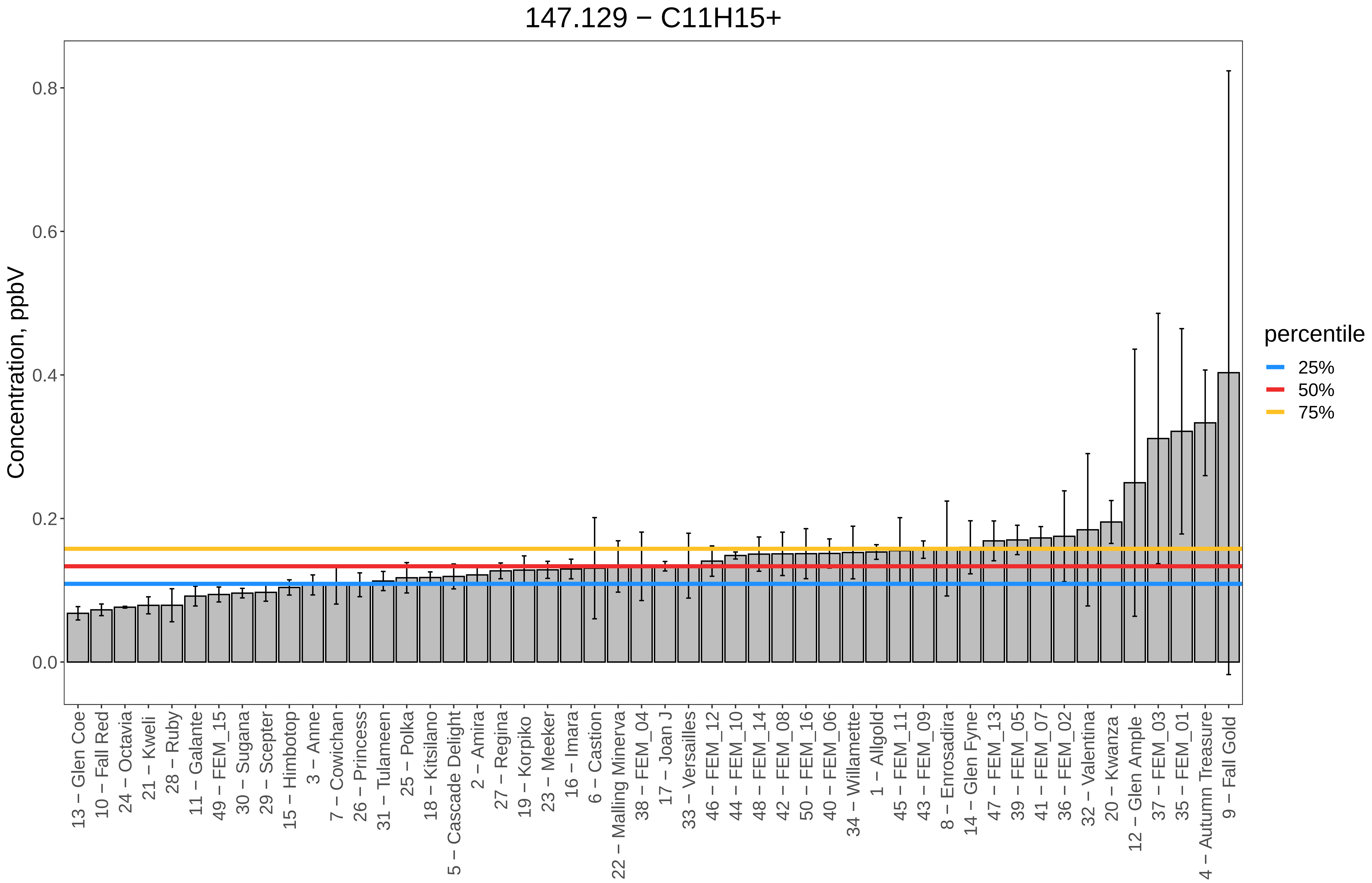
147.129 − C11H15+
Concentration, ppbV
0.0
0.2
0.4
0.6
0.8
percentile
25%
50%
75%
13 − Glen Coe
10 − Fall Red
24 − Octavia
21 − Kweli
28 − Ruby
11 − Galante
49 − FEM_15
30 − Sugana
29 − Scepter
15 − Himbotop
3 − Anne
7 − Cowichan
26 − Princess
31 − Tulameen
25 − Polka
18 − Kitsilano
5 − Cascade Delight
2 − Amira
27 − Regina
19 − Korpiko
23 − Meeker
16 − Imara
6 − Castion
22 − Malling Minerva
38 − FEM_04
17 − Joan J
33 − Versailles
46 − FEM_12
44 − FEM_10
48 − FEM_14
42 − FEM_08
50 − FEM_16
40 − FEM_06
34 − Willamette
1 − Allgold
45 − FEM_11
43 − FEM_09
8 − Enrosadira
14 − Glen Fyne
47 − FEM_13
39 − FEM_05
41 − FEM_07
36 − FEM_02
32 − Valentina
20 − Kwanza
12 − Glen Ample
37 − FEM_03
35 − FEM_01
4 − Autumn Treasure
9 − Fall Gold

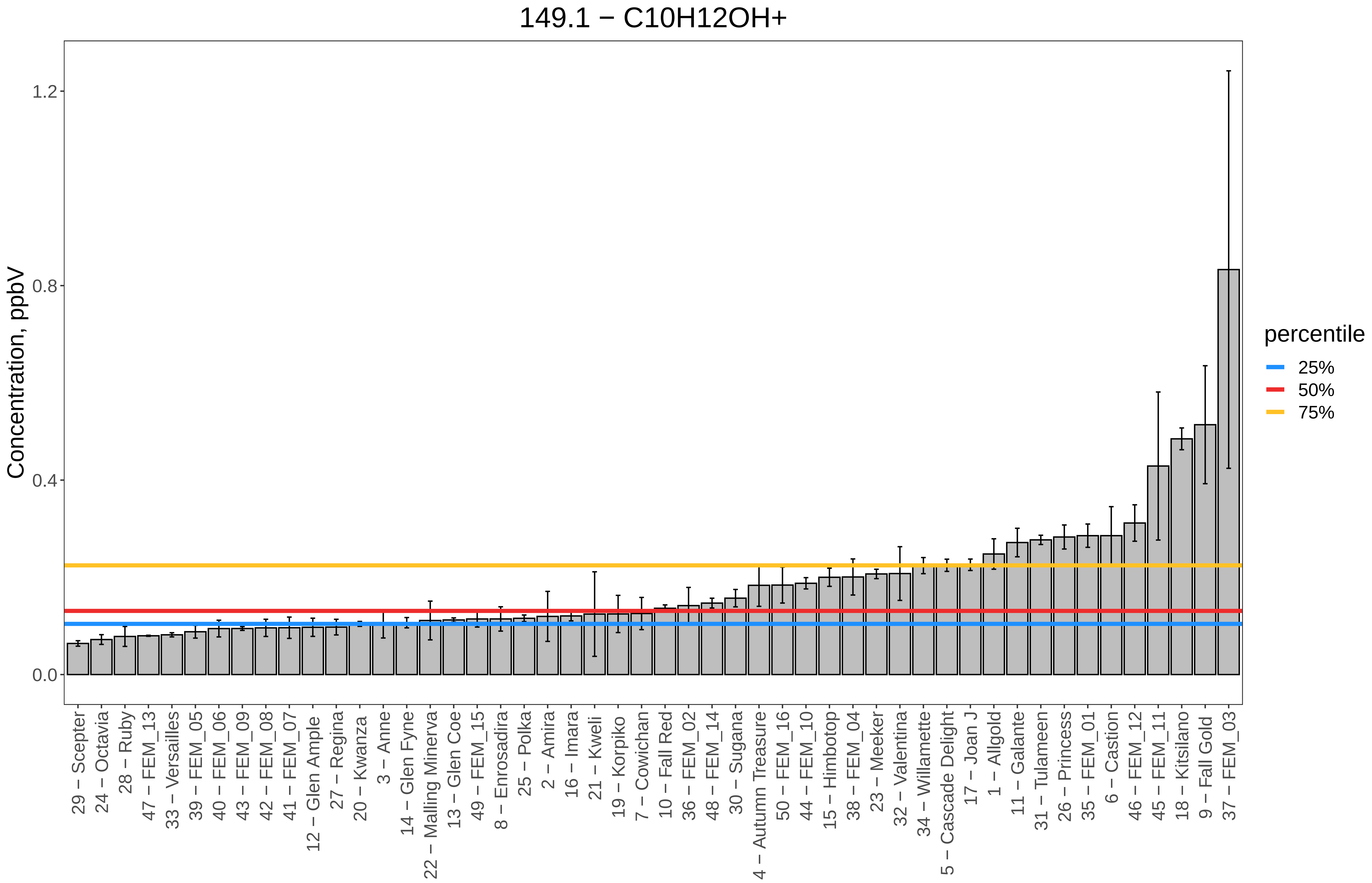

149.1 − C10H12OH+
Concentration, ppbV
0.0
0.4
0.8
1.2
percentile
25%
50%
75%
29 − Scepter
24 − Octavia
28 − Ruby
47 − FEM_13
33 − Versailles
39 − FEM_05
40 − FEM_06
43 − FEM_09
42 − FEM_08
41 − FEM_07
12 − Glen Ample
27 − Regina
20 − Kwanza
3 − Anne
14 − Glen Fyne
22 − Malling Minerva
13 − Glen Coe
49 − FEM_15
8 − Enrosadira
25 − Polka
2 − Amira
16 − Imara
21 − Kweli
19 − Korpiko
7 − Cowichan
10 − Fall Red
36 − FEM_02
48 − FEM_14
30 − Sugana
4 − Autumn Treasure
50 − FEM_16
44 − FEM_10
15 − Himbotop
38 − FEM_04
23 − Meeker
32 − Valentina
34 − Willamette
5 − Cascade Delight
17 − Joan J
1 − Allgold
11 − Galante
31 − Tulameen
26 − Princess
35 − FEM_01
6 − Castion
46 − FEM_12
45 − FEM_11
18 − Kitsilano
9 − Fall Gold
37 − FEM_03

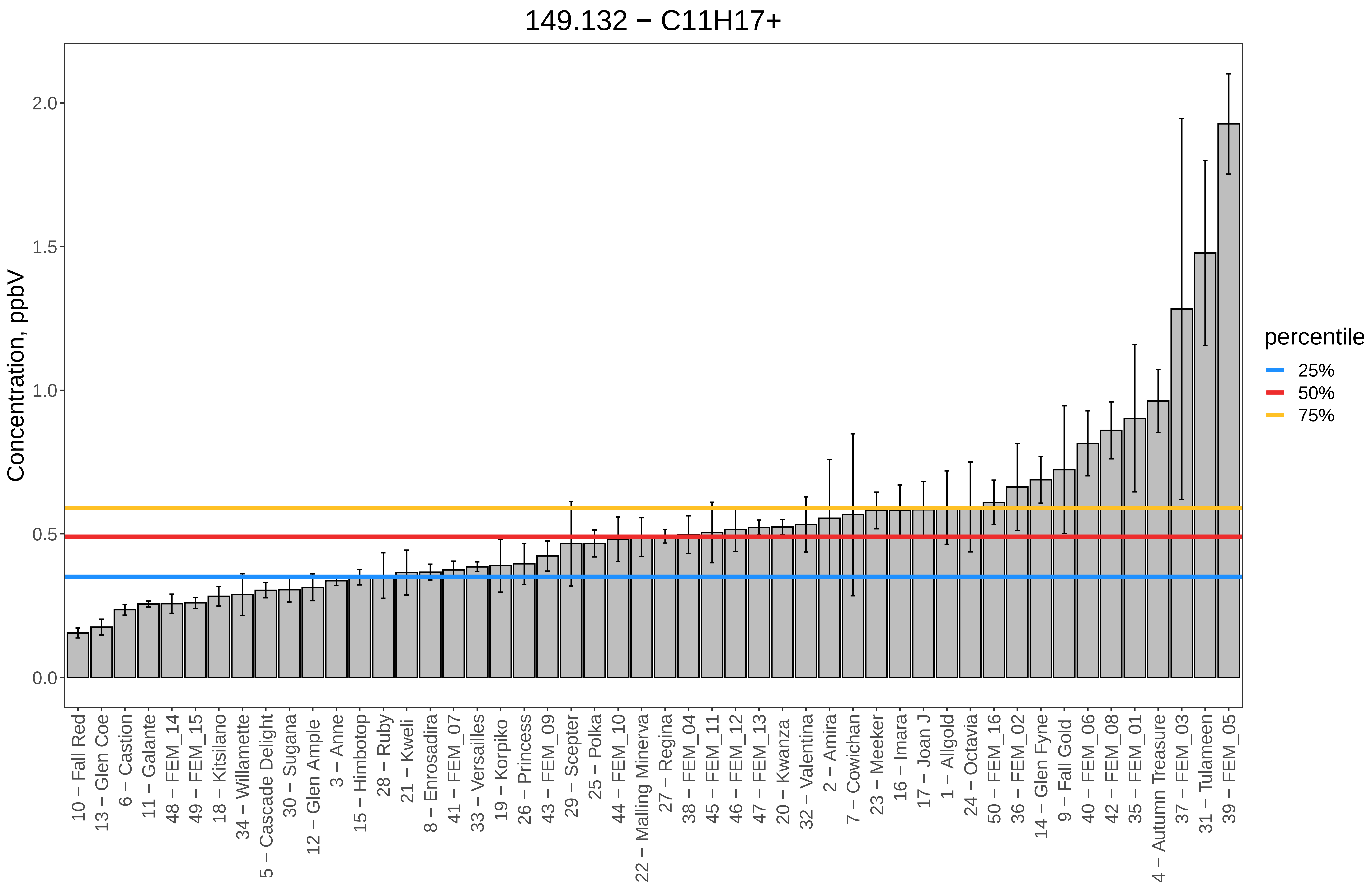

149.132 − C11H17+
Concentration, ppbV
0.0
0.5
1.0
1.5
2.0
percentile
25%
50%
75%
10 − Fall Red
13 − Glen Coe
6 − Castion
11 − Galante
48 − FEM_14
49 − FEM_15
18 − Kitsilano
34 − Willamette
5 − Cascade Delight
30 − Sugana
12 − Glen Ample
3 − Anne
15 − Himbotop
28 − Ruby
21 − Kweli
8 − Enrosadira
41 − FEM_07
33 − Versailles
19 − Korpiko
26 − Princess
43 − FEM_09
29 − Scepter
25 − Polka
44 − FEM_10
22 − Malling Minerva
27 − Regina
38 − FEM_04
45 − FEM_11
46 − FEM_12
47 − FEM_13
20 − Kwanza
32 − Valentina
2 − Amira
7 − Cowichan
23 − Meeker
16 − Imara
17 − Joan J
1 − Allgold
24 − Octavia
50 − FEM_16
36 − FEM_02
14 − Glen Fyne
9 − Fall Gold
40 − FEM_06
42 − FEM_08
35 − FEM_01
4 − Autumn Treasure
37 − FEM_03
31 − Tulameen
39 − FEM_05

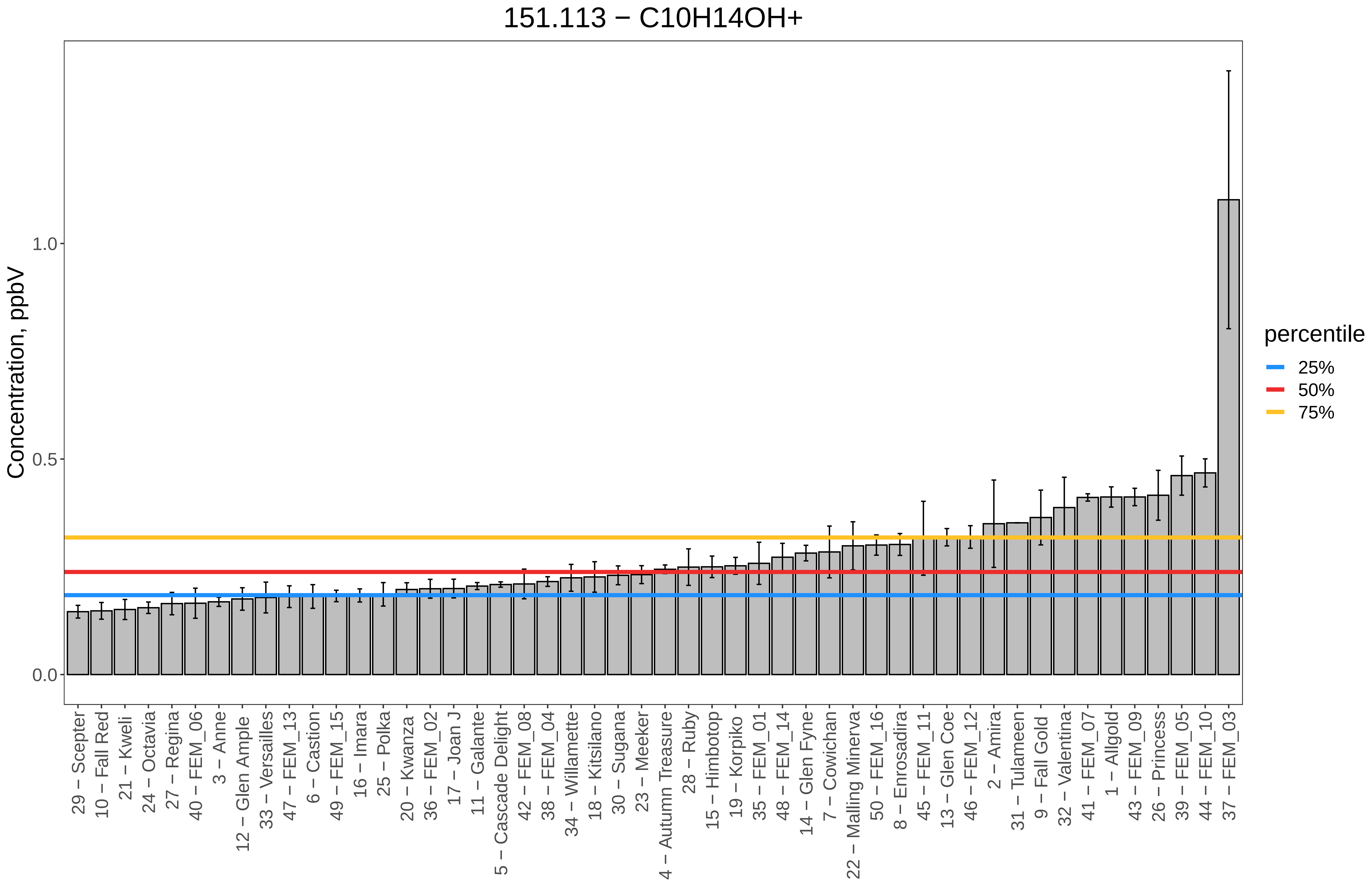
151.113 − C10H14OH+
Concentration, ppbV
0.0
0.5
1.0
percentile
25%
50%
75%
29 − Scepter
10 − Fall Red
21 − Kweli
24 − Octavia
27 − Regina
40 − FEM_06
3 − Anne
12 − Glen Ample
33 − Versailles
47 − FEM_13
6 − Castion
49 − FEM_15
16 − Imara
25 − Polka
20 − Kwanza
36 − FEM_02
17 − Joan J
11 − Galante
5 − Cascade Delight
42 − FEM_08
38 − FEM_04
34 − Willamette
18 − Kitsilano
30 − Sugana
23 − Meeker
4 − Autumn Treasure
28 − Ruby
15 − Himbotop
19 − Korpiko
35 − FEM_01
48 − FEM_14
14 − Glen Fyne
7 − Cowichan
22 − Malling Minerva
50 − FEM_16
8 − Enrosadira
45 − FEM_11
13 − Glen Coe
46 − FEM_12
2 − Amira
31 − Tulameen
9 − Fall Gold
32 − Valentina
41 − FEM_07
1 − Allgold
43 − FEM_09
26 − Princess
39 − FEM_05
44 − FEM_10
37 − FEM_03

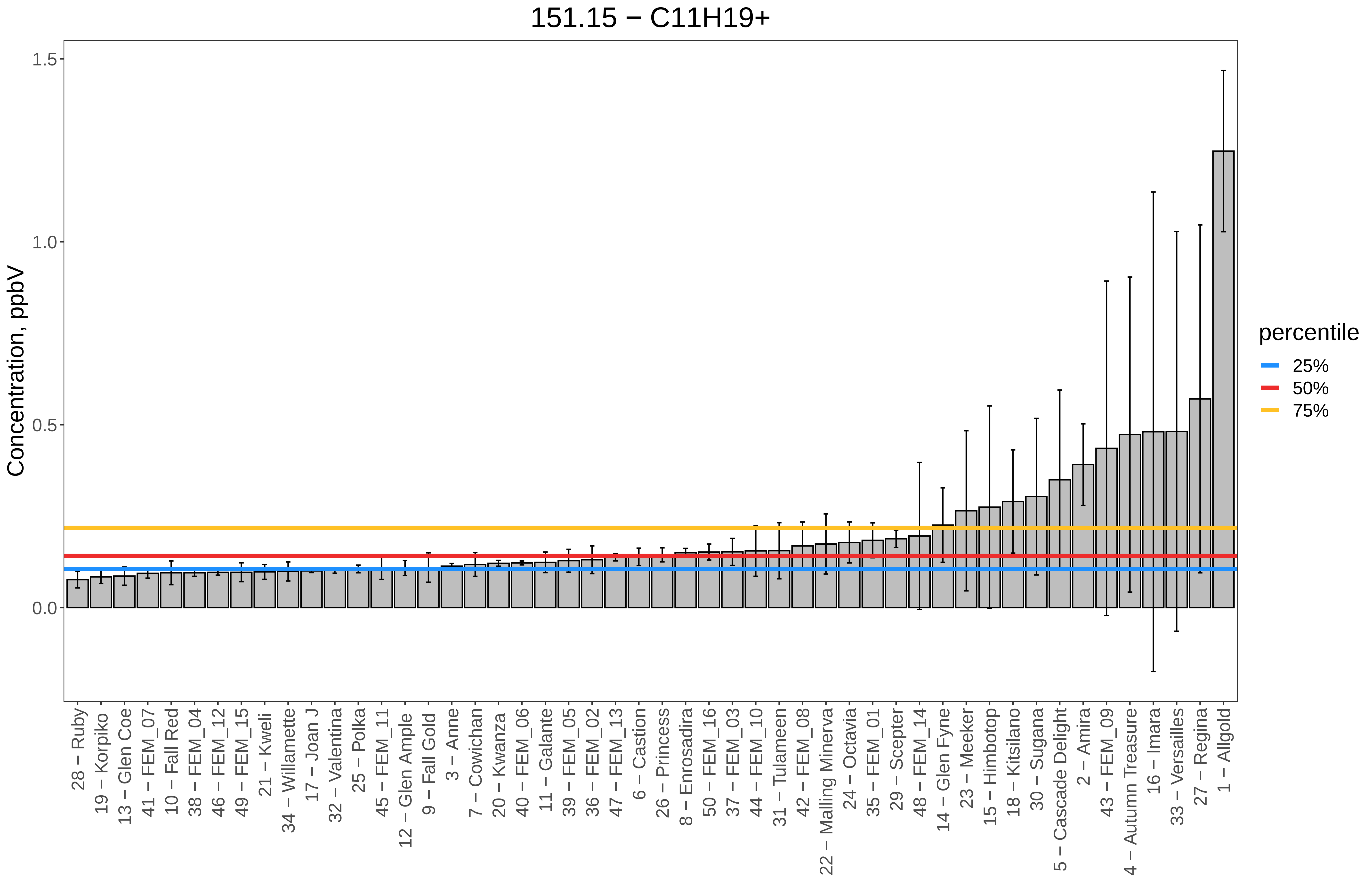

151.15 − C11H19+
Concentration, ppbV
0.0
0.5
1.0
1.5
percentile
25%
50%
75%
28 − Ruby
19 − Korpiko
13 − Glen Coe
41 − FEM_07
10 − Fall Red
38 − FEM_04
46 − FEM_12
49 − FEM_15
21 − Kweli
34 − Willamette
17 − Joan J
32 − Valentina
25 − Polka
45 − FEM_11
12 − Glen Ample
9 − Fall Gold
3 − Anne
7 − Cowichan
20 − Kwanza
40 − FEM_06
11 − Galante
39 − FEM_05
36 − FEM_02
47 − FEM_13
6 − Castion
26 − Princess
8 − Enrosadira
50 − FEM_16
37 − FEM_03
44 − FEM_10
31 − Tulameen
42 − FEM_08
22 − Malling Minerva
24 − Octavia
35 − FEM_01
29 − Scepter
48 − FEM_14
14 − Glen Fyne
23 − Meeker
15 − Himbotop
18 − Kitsilano
30 − Sugana
5 − Cascade Delight
2 − Amira
43 − FEM_09
4 − Autumn Treasure
16 − Imara
33 − Versailles
27 − Regina
1 − Allgold

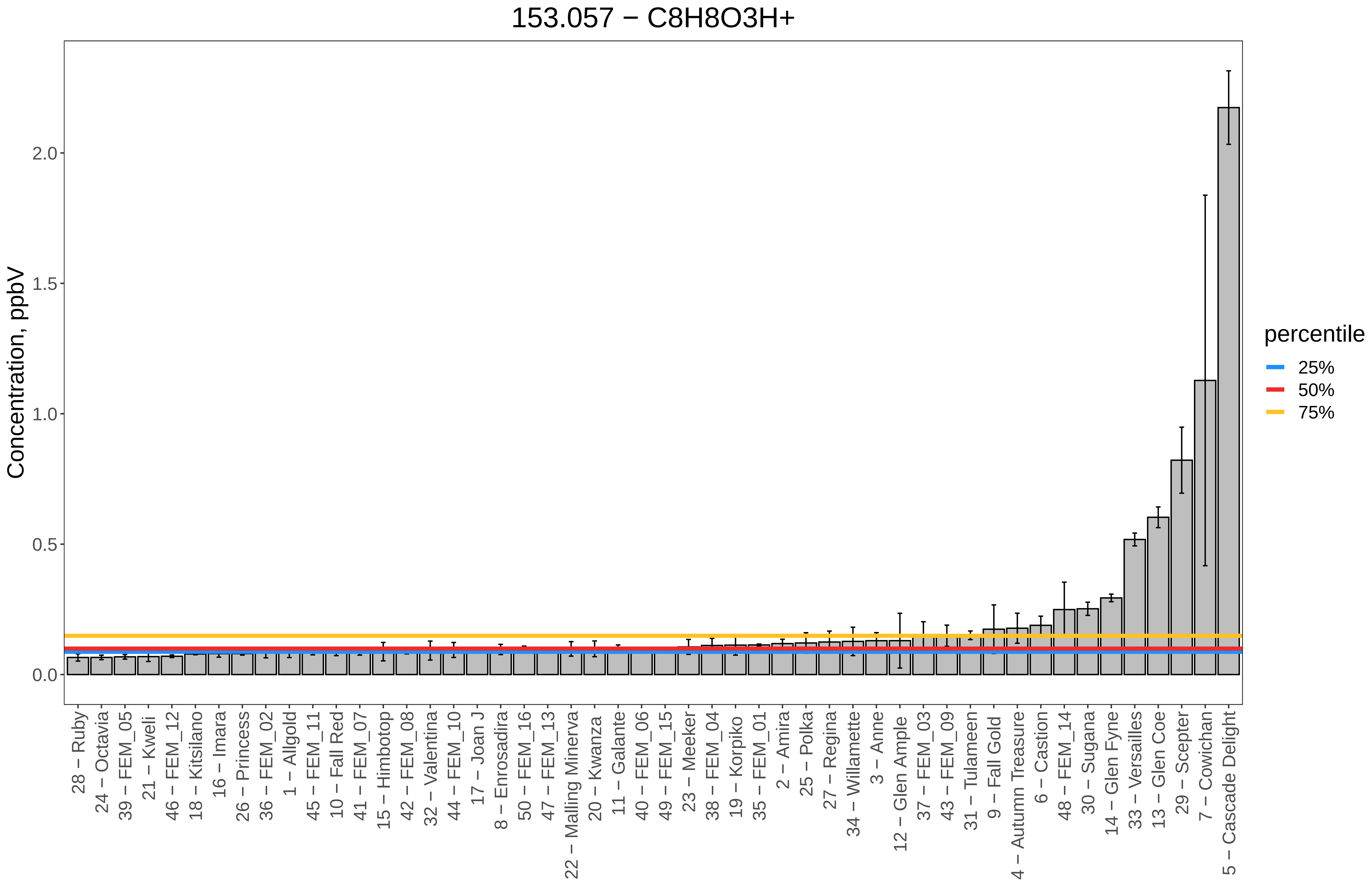
153.057 − C8H8O3H+
Concentration, ppbV
0.0
0.5
1.0
1.5
2.0
28 − Ruby
24 − Octavia
39 − FEM_05
21 − Kweli
46 − FEM_12
18 − Kitsilano
16 − Imara
26 − Princess
36 − FEM_02
1 − Allgold
45 − FEM_11
10 − Fall Red
41 − FEM_07
15 − Himbotop
42 − FEM_08
32 − Valentina
44 − FEM_10
17 − Joan J
8 − Enrosadira
50 − FEM_16
47 − FEM_13
22 − Malling Minerva
20 − Kwanza
11 − Galante
40 − FEM_06
49 − FEM_15
23 − Meeker
38 − FEM_04
19 − Korpiko
35 − FEM_01
2 − Amira
25 − Polka
27 − Regina
34 − Willamette
3 − Anne
12 − Glen Ample
37 − FEM_03
43 − FEM_09
31 − Tulameen
9 − Fall Gold
4 − Autumn Treasure
6 − Castion
48 − FEM_14
30 − Sugana
14 − Glen Fyne
33 − Versailles
13 − Glen Coe
29 − Scepter
7 − Cowichan
5 − Cascade Delight
percentile
25%
50%
75%

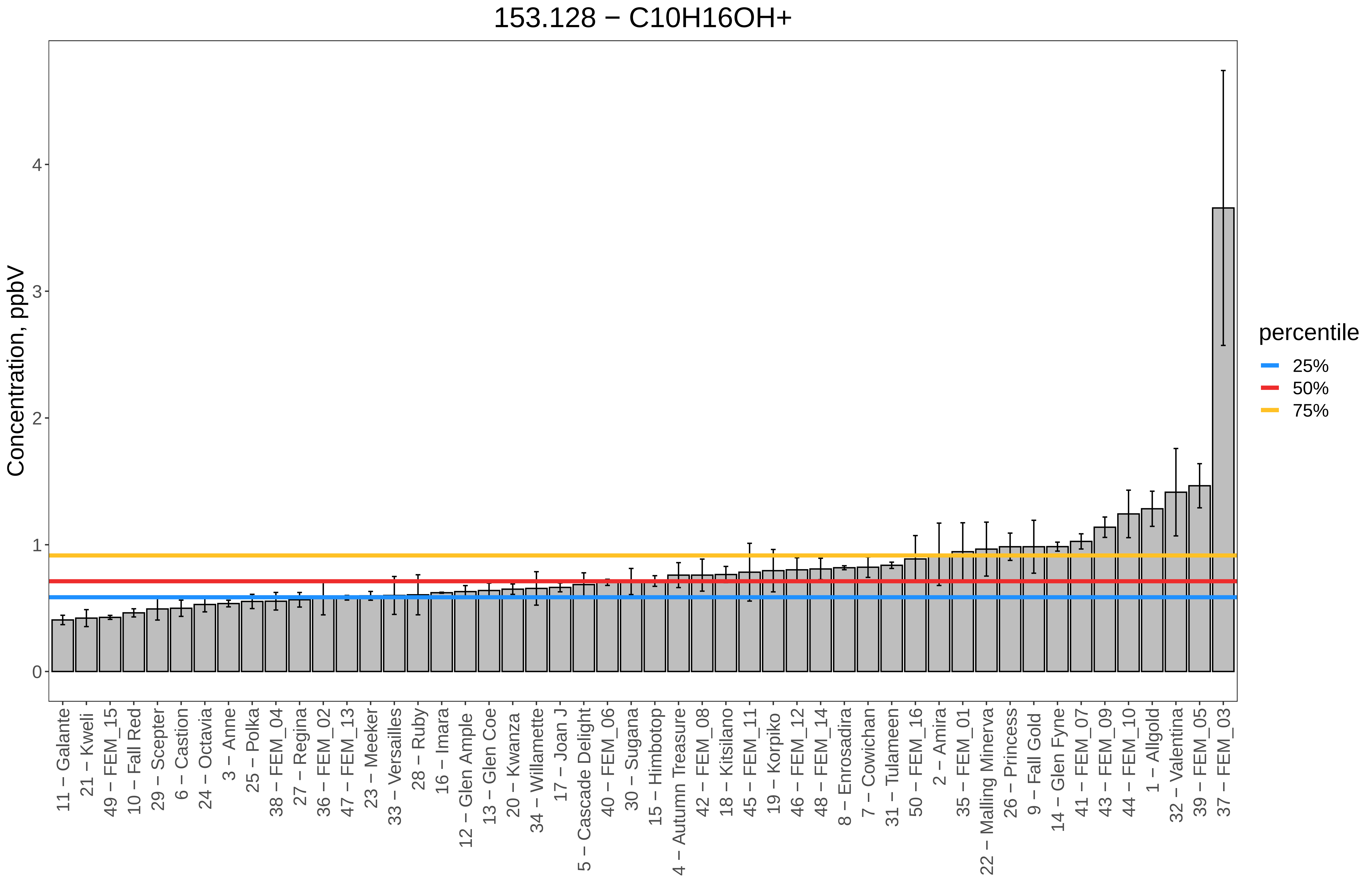

153.128 − C10H16OH+
Concentration, ppbV
0
1
2
3
4
percentile
25%
50%
75%
11 − Galante
21 − Kweli
49 − FEM_15
10 − Fall Red
29 − Scepter
6 − Castion
24 − Octavia
3 − Anne
25 − Polka
38 − FEM_04
27 − Regina
36 − FEM_02
47 − FEM_13
23 − Meeker
33 − Versailles
28 − Ruby
16 − Imara
12 − Glen Ample
13 − Glen Coe
20 − Kwanza
34 − Willamette
17 − Joan J
5 − Cascade Delight
40 − FEM_06
30 − Sugana
15 − Himbotop
4 − Autumn Treasure
42 − FEM_08
18 − Kitsilano
45 − FEM_11
19 − Korpiko
46 − FEM_12
48 − FEM_14
8 − Enrosadira
7 − Cowichan
31 − Tulameen
50 − FEM_16
2 − Amira
35 − FEM_01
22 − Malling Minerva
26 − Princess
9 − Fall Gold
14 − Glen Fyne
41 − FEM_07
43 − FEM_09
44 − FEM_10
1 − Allgold
32 − Valentina
39 − FEM_05
37 − FEM_03

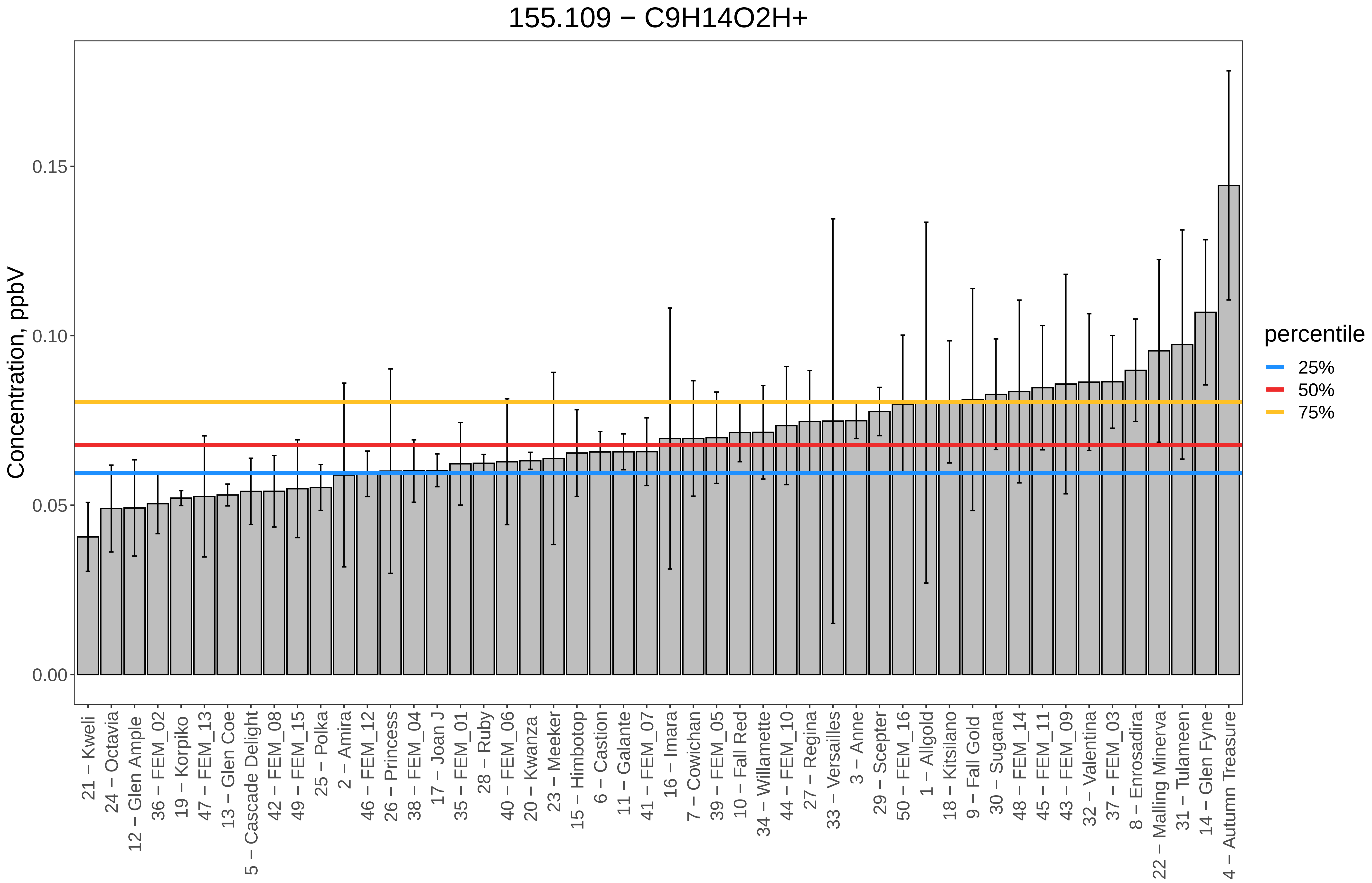
155.109 − C9H14O2H+
Concentration, ppbV
0.00
0.05
0.10
0.15
percentile
25%
50%
75%
21 − Kweli
24 − Octavia
12 − Glen Ample
36 − FEM_02
19 − Korpiko
47 − FEM_13
13 − Glen Coe
5 − Cascade Delight
42 − FEM_08
49 − FEM_15
25 − Polka
2 − Amira
46 − FEM_12
26 − Princess
38 − FEM_04
17 − Joan J
35 − FEM_01
28 − Ruby
40 − FEM_06
20 − Kwanza
23 − Meeker
15 − Himbotop
6 − Castion
11 − Galante
41 − FEM_07
16 − Imara
7 − Cowichan
39 − FEM_05
10 − Fall Red
34 − Willamette
44 − FEM_10
27 − Regina
33 − Versailles
3 − Anne
29 − Scepter
50 − FEM_16
1 − Allgold
18 − Kitsilano
9 − Fall Gold
30 − Sugana
48 − FEM_14
45 − FEM_11
43 − FEM_09
32 − Valentina
37 − FEM_03
8 − Enrosadira
22 − Malling Minerva
31 − Tulameen
14 − Glen Fyne
4 − Autumn Treasure

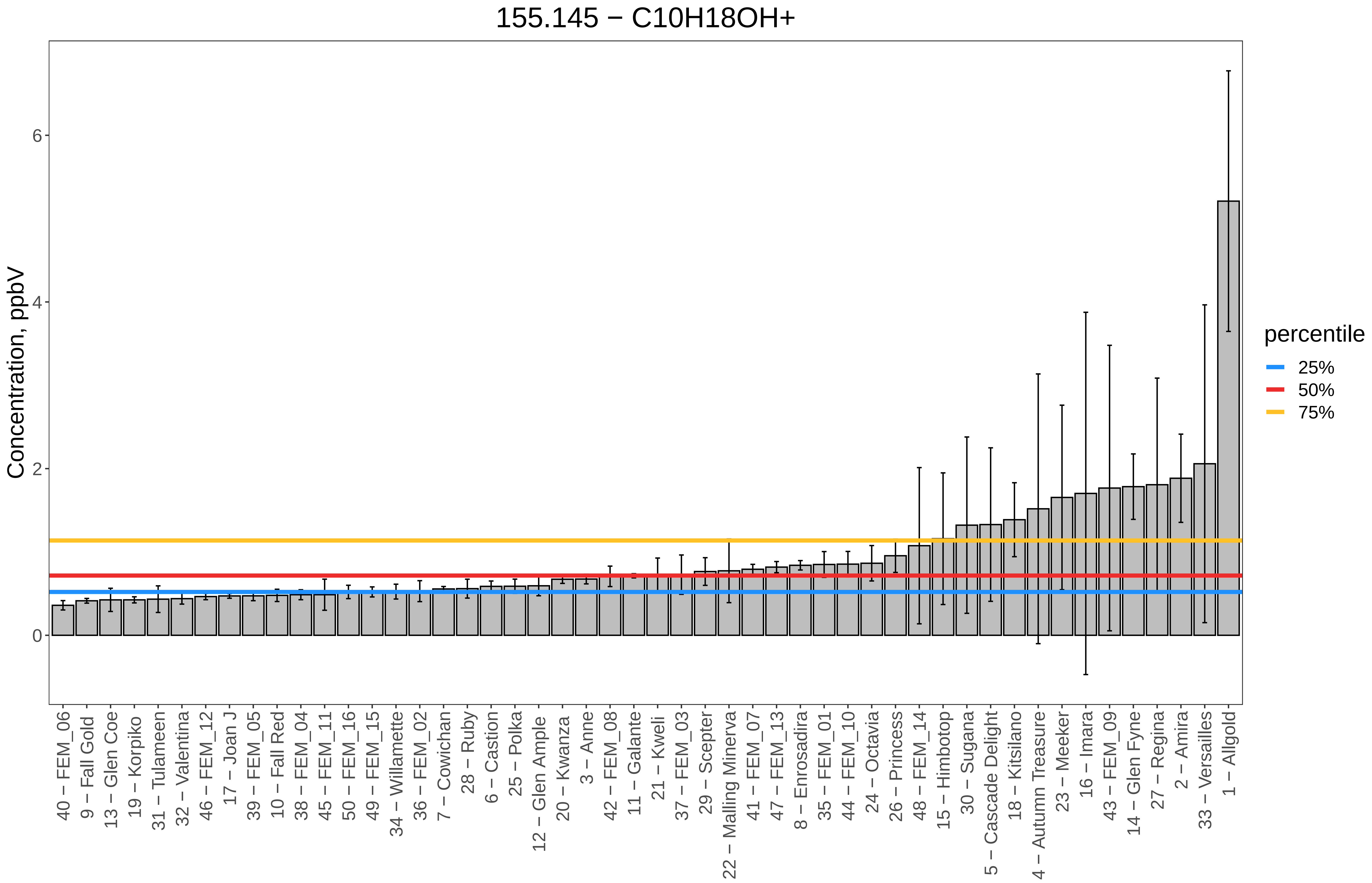

155.145 − C10H18OH+
Concentration, ppbV
0
2
4
6
percentile
25%
50%
75%
40 − FEM_06
9 − Fall Gold
13 − Glen Coe
19 − Korpiko
31 − Tulameen
32 − Valentina
46 − FEM_12
17 − Joan J
39 − FEM_05
10 − Fall Red
38 − FEM_04
45 − FEM_11
50 − FEM_16
49 − FEM_15
34 − Willamette
36 − FEM_02
7 − Cowichan
28 − Ruby
6 − Castion
25 − Polka
12 − Glen Ample
20 − Kwanza
3 − Anne
42 − FEM_08
11 − Galante
21 − Kweli
37 − FEM_03
29 − Scepter
22 − Malling Minerva
41 − FEM_07
47 − FEM_13
8 − Enrosadira
35 − FEM_01
44 − FEM_10
24 − Octavia
26 − Princess
48 − FEM_14
15 − Himbotop
30 − Sugana
5 − Cascade Delight
18 − Kitsilano
4 − Autumn Treasure
23 − Meeker
16 − Imara
43 − FEM_09
14 − Glen Fyne
27 − Regina
2 − Amira
33 − Versailles
1 − Allgold

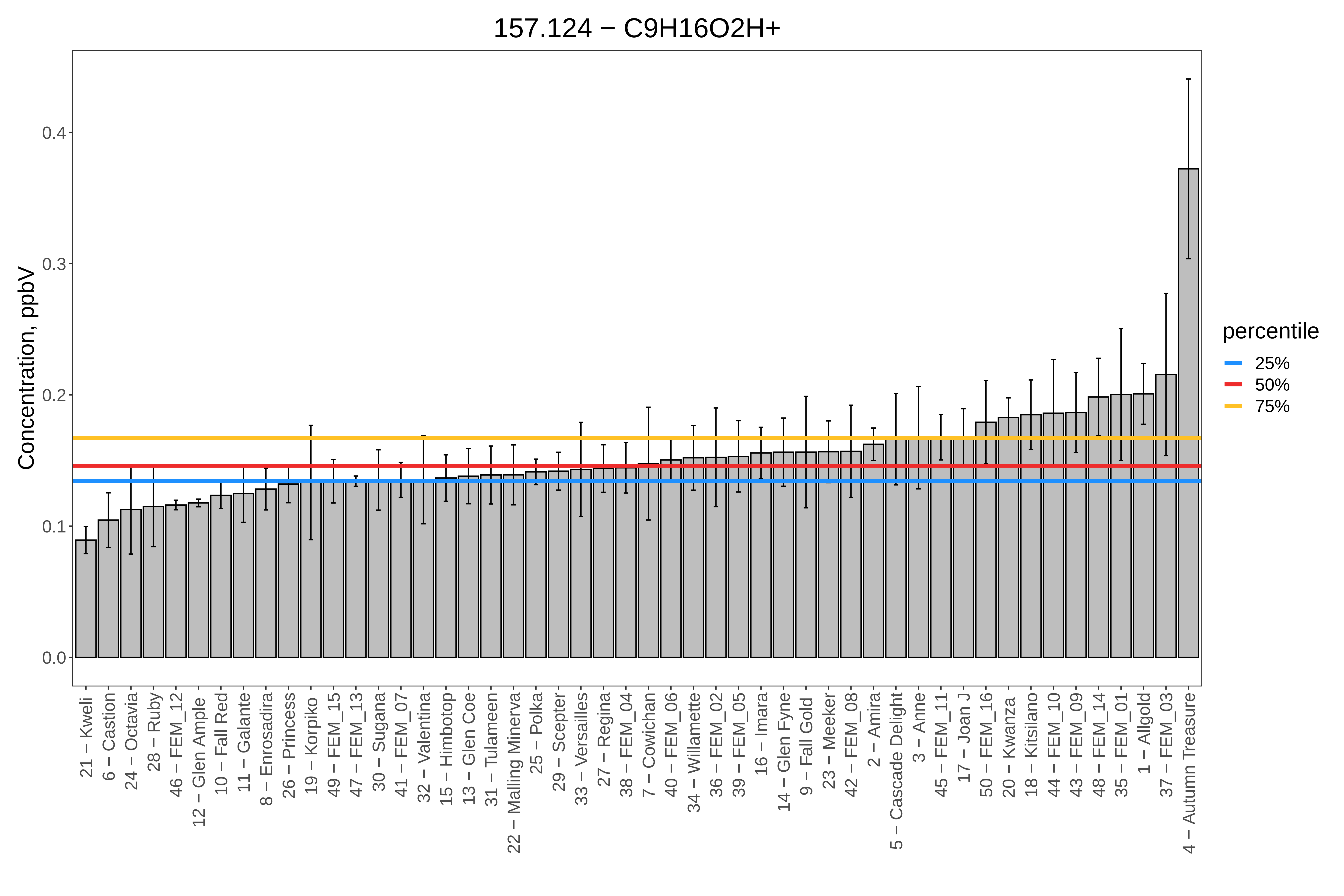

157.124 − C9H16O2H+
Concentration, ppbV
0.0
0.1
0.2
0.3
0.4
percentile
25%
50%
75%
21 − Kweli
6 − Castion
24 − Octavia
28 − Ruby
46 − FEM_12
12 − Glen Ample
10 − Fall Red
11 − Galante
8 − Enrosadira
26 − Princess
19 − Korpiko
49 − FEM_15
47 − FEM_13
30 − Sugana
41 − FEM_07
32 − Valentina
15 − Himbotop
13 − Glen Coe
31 − Tulameen
22 − Malling Minerva
25 − Polka
29 − Scepter
33 − Versailles
27 − Regina
38 − FEM_04
7 − Cowichan
40 − FEM_06
34 − Willamette
36 − FEM_02
39 − FEM_05
16 − Imara
14 − Glen Fyne
9 − Fall Gold
23 − Meeker
42 − FEM_08
2 − Amira
5 − Cascade Delight
3 − Anne
45 − FEM_11
17 − Joan J
50 − FEM_16
20 − Kwanza
18 − Kitsilano
44 − FEM_10
43 − FEM_09
48 − FEM_14
35 − FEM_01
1 − Allgold
37 − FEM_03
4 − Autumn Treasure

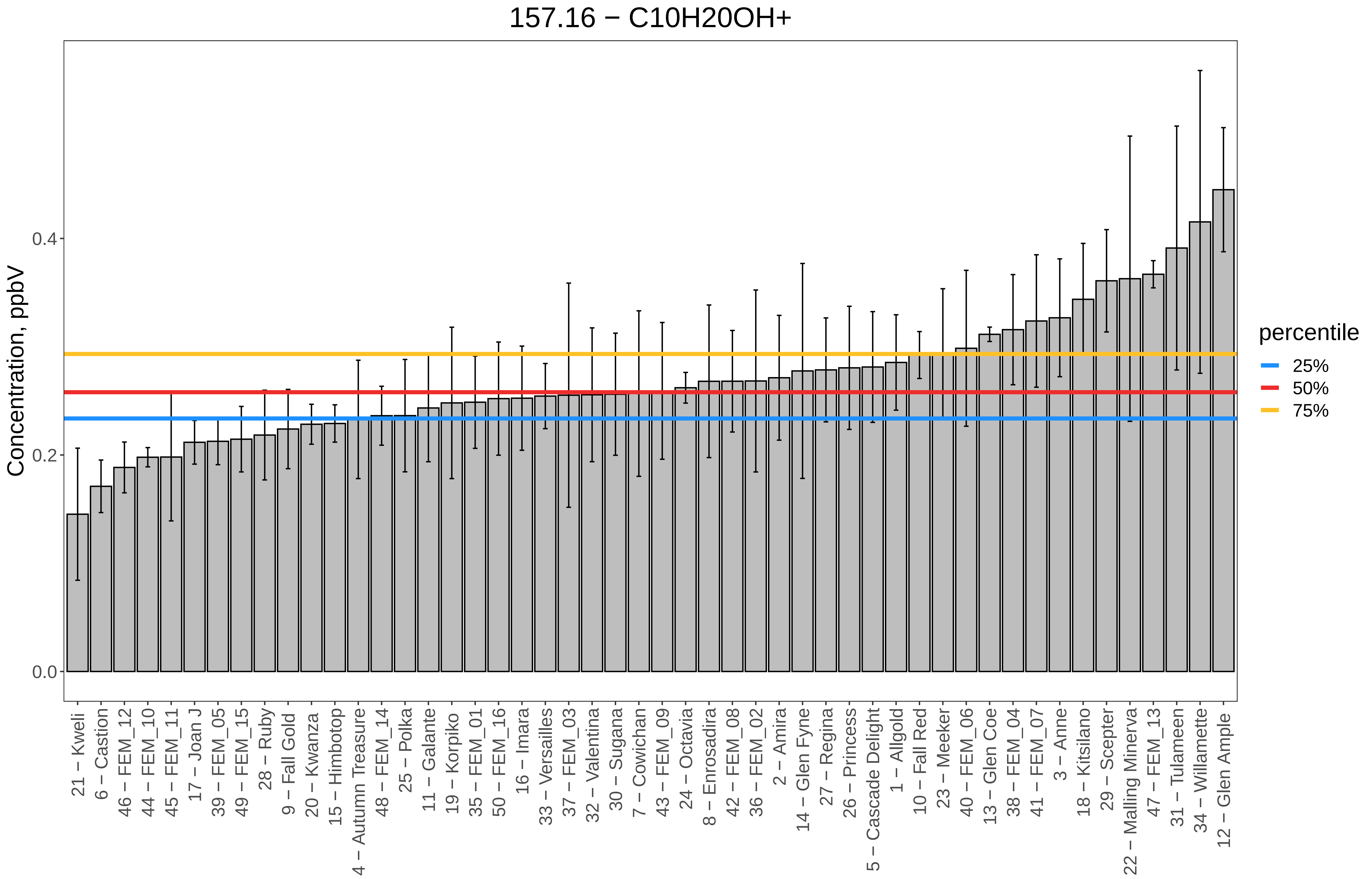

157.16 − C10H20OH+
Concentration, ppbV
0.0
0.2
0.4
percentile
25%
50%
75%
21 − Kweli
6 − Castion
46 − FEM_12
44 − FEM_10
45 − FEM_11
17 − Joan J
39 − FEM_05
49 − FEM_15
28 − Ruby
9 − Fall Gold
20 − Kwanza
15 − Himbotop
4 − Autumn Treasure
48 − FEM_14
25 − Polka
11 − Galante
19 − Korpiko
35 − FEM_01
50 − FEM_16
16 − Imara
33 − Versailles
37 − FEM_03
32 − Valentina
30 − Sugana
7 − Cowichan
43 − FEM_09
24 − Octavia
8 − Enrosadira
42 − FEM_08
36 − FEM_02
2 − Amira
14 − Glen Fyne
27 − Regina
26 − Princess
5 − Cascade Delight
1 − Allgold
10 − Fall Red
23 − Meeker
40 − FEM_06
13 − Glen Coe
38 − FEM_04
41 − FEM_07
3 − Anne
18 − Kitsilano
29 − Scepter
22 − Malling Minerva
47 − FEM_13
31 − Tulameen
34 − Willamette
12 − Glen Ample

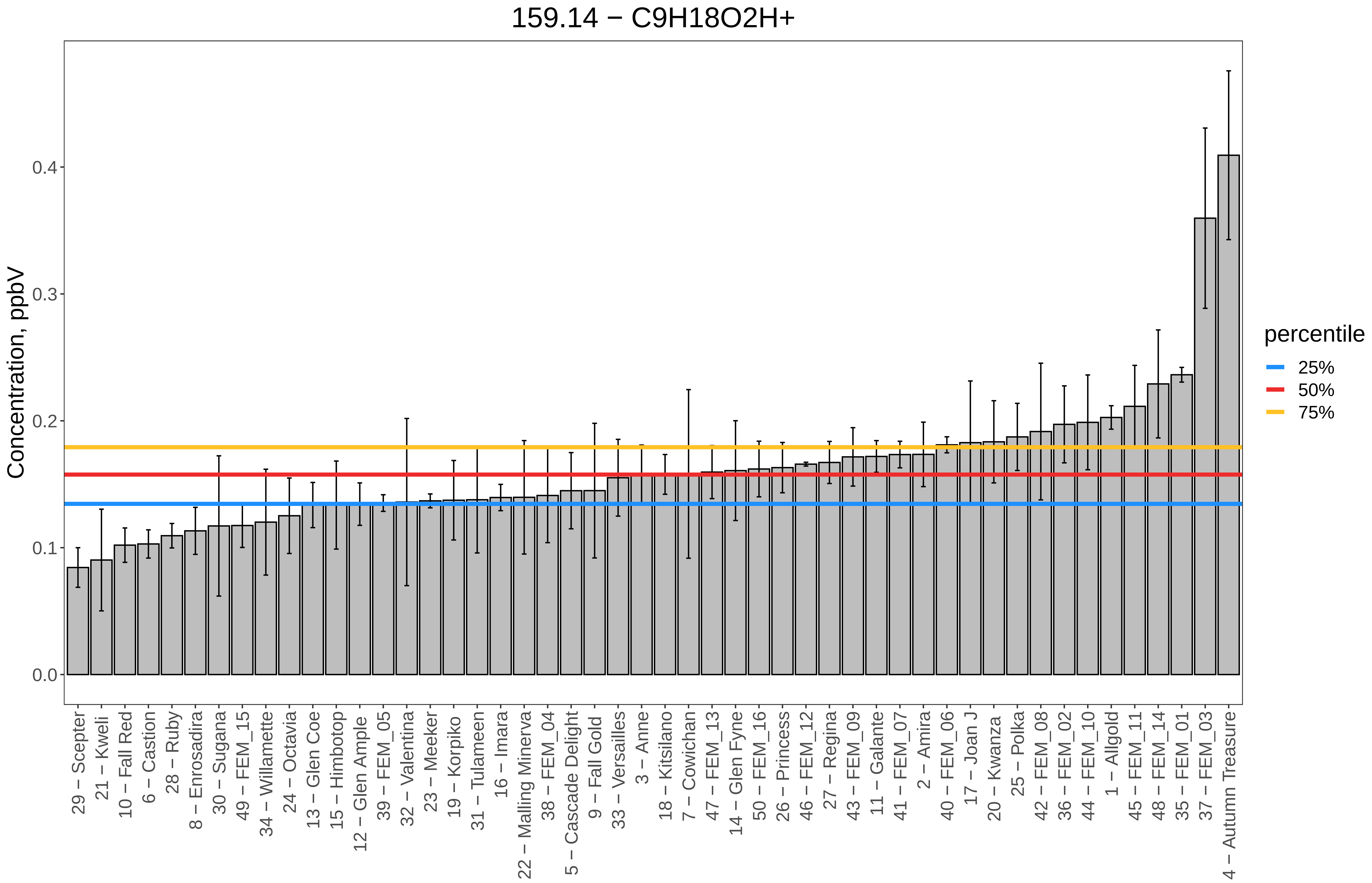

159.14 − C9H18O2H+
Concentration, ppbV
0.0
0.1
0.2
0.3
0.4
percentile
25%
50%
75%
29 − Scepter
21 − Kweli
10 − Fall Red
6 − Castion
28 − Ruby
8 − Enrosadira
30 − Sugana
49 − FEM_15
34 − Willamette
24 − Octavia
13 − Glen Coe
15 − Himbotop
12 − Glen Ample
39 − FEM_05
32 − Valentina
23 − Meeker
19 − Korpiko
31 − Tulameen
16 − Imara
22 − Malling Minerva
38 − FEM_04
5 − Cascade Delight
9 − Fall Gold
33 − Versailles
3 − Anne
18 − Kitsilano
7 − Cowichan
47 − FEM_13
14 − Glen Fyne
50 − FEM_16
26 − Princess
46 − FEM_12
27 − Regina
43 − FEM_09
11 − Galante
41 − FEM_07
2 − Amira
40 − FEM_06
17 − Joan J
20 − Kwanza
25 − Polka
42 − FEM_08
36 − FEM_02
44 − FEM_10
1 − Allgold
45 − FEM_11
48 − FEM_14
35 − FEM_01
37 − FEM_03
4 − Autumn Treasure

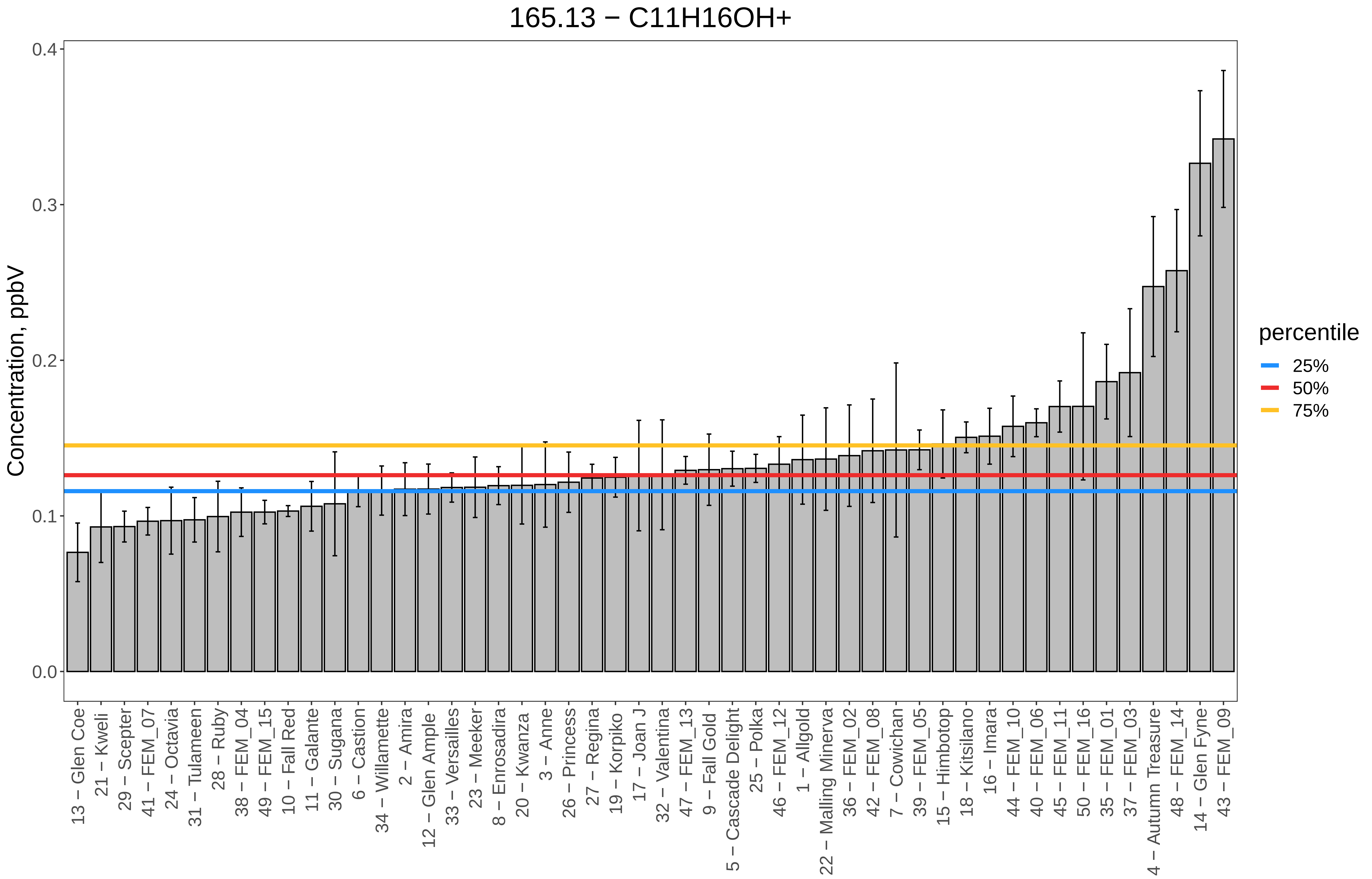

165.13 − C11H16OH+
Concentration, ppbV
0.0
0.1
0.2
0.3
0.4
percentile
25%
50%
75%
13 − Glen Coe
21 − Kweli
29 − Scepter
41 − FEM_07
24 − Octavia
31 − Tulameen
28 − Ruby
38 − FEM_04
49 − FEM_15
10 − Fall Red
11 − Galante
30 − Sugana
6 − Castion
34 − Willamette
2 − Amira
12 − Glen Ample
33 − Versailles
23 − Meeker
8 − Enrosadira
20 − Kwanza
3 − Anne
26 − Princess
27 − Regina
19 − Korpiko
17 − Joan J
32 − Valentina
47 − FEM_13
9 − Fall Gold
5 − Cascade Delight
25 − Polka
46 − FEM_12
1 − Allgold
22 − Malling Minerva
36 − FEM_02
42 − FEM_08
7 − Cowichan
39 − FEM_05
15 − Himbotop
18 − Kitsilano
16 − Imara
44 − FEM_10
40 − FEM_06
45 − FEM_11
50 − FEM_16
35 − FEM_01
37 − FEM_03
4 − Autumn Treasure
48 − FEM_14
14 − Glen Fyne
43 − FEM_09

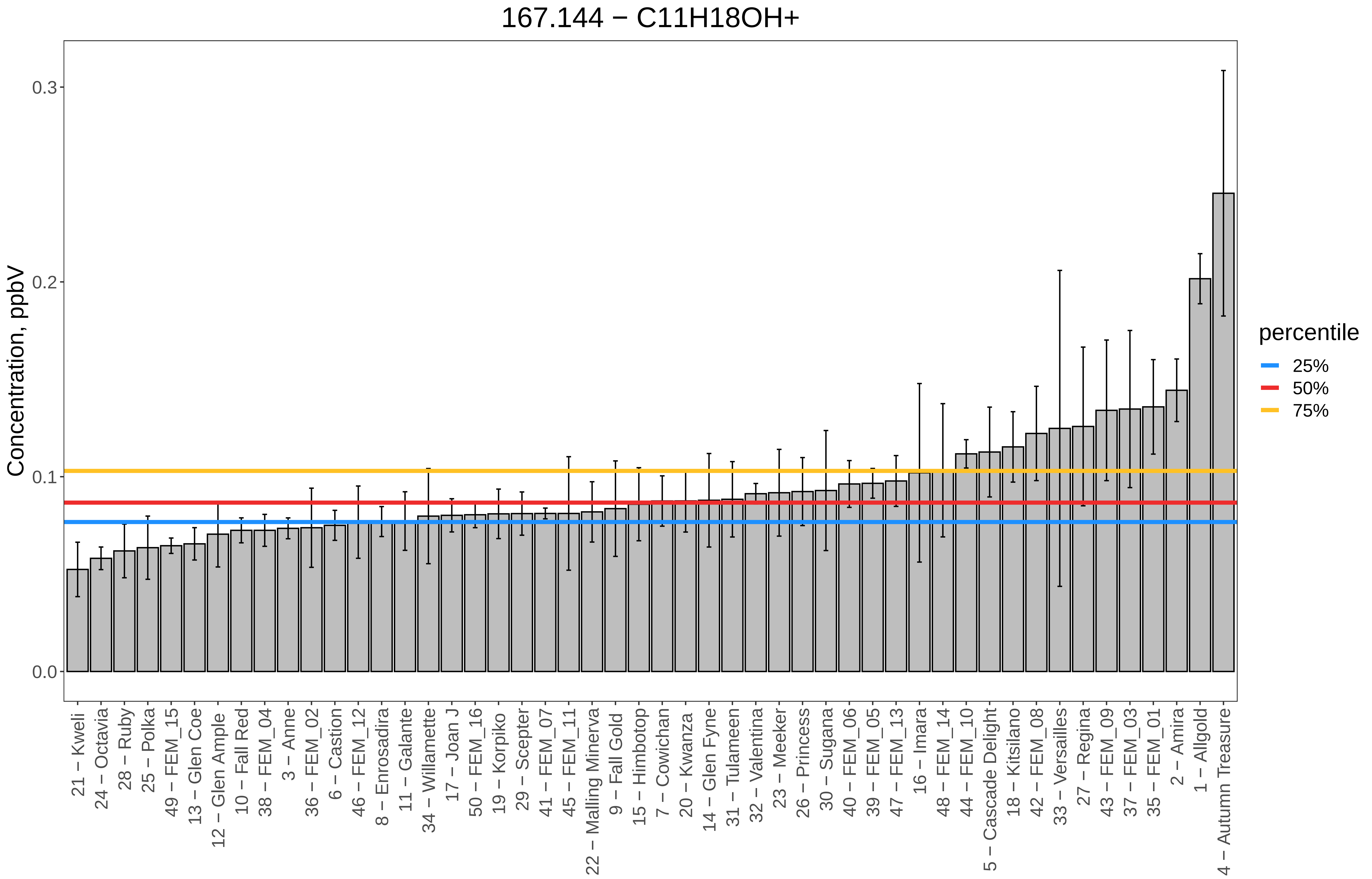

167.144 − C11H18OH+
Concentration, ppbV
0.0
0.1
0.2
0.3
percentile
25%
50%
75%
21 − Kweli
24 − Octavia
28 − Ruby
25 − Polka
49 − FEM_15
13 − Glen Coe
12 − Glen Ample
10 − Fall Red
38 − FEM_04
3 − Anne
36 − FEM_02
6 − Castion
46 − FEM_12
8 − Enrosadira
11 − Galante
34 − Willamette
17 − Joan J
50 − FEM_16
19 − Korpiko
29 − Scepter
41 − FEM_07
45 − FEM_11
22 − Malling Minerva
9 − Fall Gold
15 − Himbotop
7 − Cowichan
20 − Kwanza
14 − Glen Fyne
31 − Tulameen
32 − Valentina
23 − Meeker
26 − Princess
30 − Sugana
40 − FEM_06
39 − FEM_05
47 − FEM_13
16 − Imara
48 − FEM_14
44 − FEM_10
5 − Cascade Delight
18 − Kitsilano
42 − FEM_08
33 − Versailles
27 − Regina
43 − FEM_09
37 − FEM_03
35 − FEM_01
2 − Amira
1 − Allgold
4 − Autumn Treasure

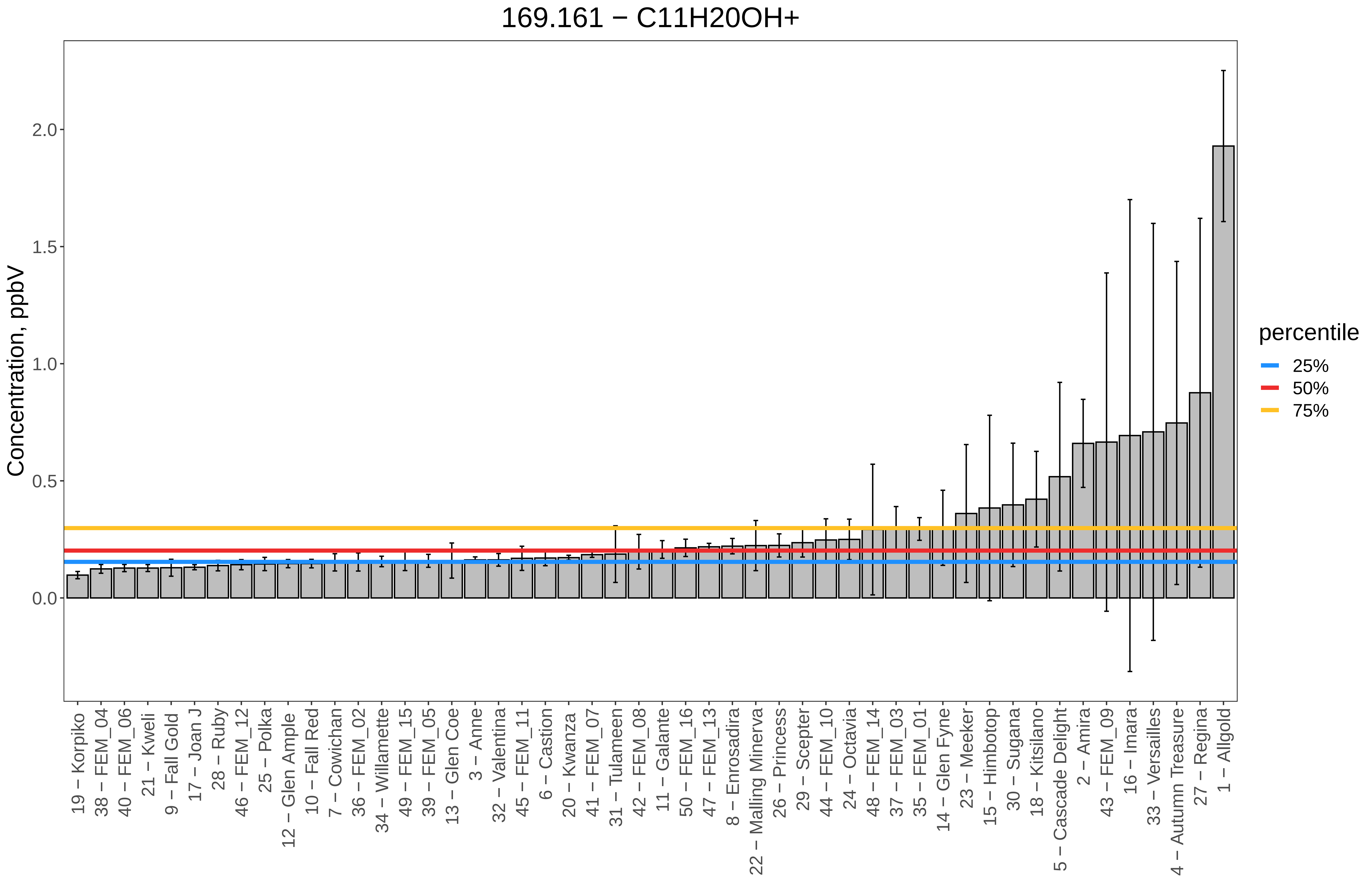
169.161 − C11H20OH+
Concentration, ppbV
0.0
0.5
1.0
1.5
2.0
percentile
25%
50%
75%
19 − Korpiko
38 − FEM_04
40 − FEM_06
21 − Kweli
9 − Fall Gold
17 − Joan J
28 − Ruby
46 − FEM_12
25 − Polka
12 − Glen Ample
10 − Fall Red
7 − Cowichan
36 − FEM_02
34 − Willamette
49 − FEM_15
39 − FEM_05
13 − Glen Coe
3 − Anne
32 − Valentina
45 − FEM_11
6 − Castion
20 − Kwanza
41 − FEM_07
31 − Tulameen
42 − FEM_08
11 − Galante
50 − FEM_16
47 − FEM_13
8 − Enrosadira
22 − Malling Minerva
26 − Princess
29 − Scepter
44 − FEM_10
24 − Octavia
48 − FEM_14
37 − FEM_03
35 − FEM_01
14 − Glen Fyne
23 − Meeker
15 − Himbotop
30 − Sugana
18 − Kitsilano
5 − Cascade Delight
2 − Amira
43 − FEM_09
16 − Imara
33 − Versailles
4 − Autumn Treasure
27 − Regina
1 − Allgold

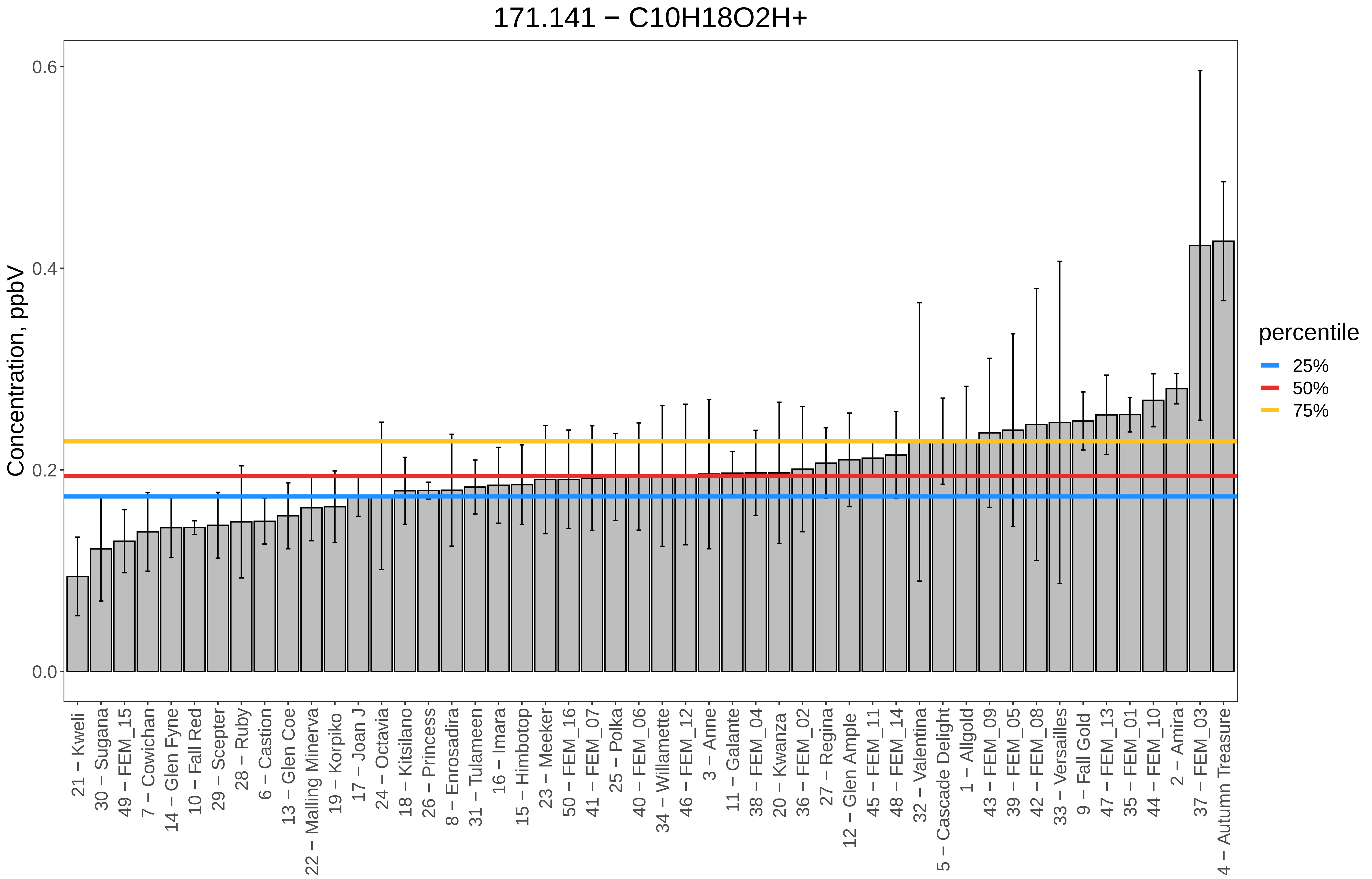
171.141 − C10H18O2H+
Concentration, ppbV
0.0
0.2
0.4
0.6
percentile
25%
50%
75%
21 − Kweli
30 − Sugana
49 − FEM_15
7 − Cowichan
14 − Glen Fyne
10 − Fall Red
29 − Scepter
28 − Ruby
6 − Castion
13 − Glen Coe
22 − Malling Minerva
19 − Korpiko
17 − Joan J
24 − Octavia
18 − Kitsilano
26 − Princess
8 − Enrosadira
31 − Tulameen
16 − Imara
15 − Himbotop
23 − Meeker
50 − FEM_16
41 − FEM_07
25 − Polka
40 − FEM_06
34 − Willamette
46 − FEM_12
3 − Anne
11 − Galante
38 − FEM_04
20 − Kwanza
36 − FEM_02
27 − Regina
12 − Glen Ample
45 − FEM_11
48 − FEM_14
32 − Valentina
5 − Cascade Delight
1 − Allgold
43 − FEM_09
39 − FEM_05
42 − FEM_08
33 − Versailles
9 − Fall Gold
47 − FEM_13
35 − FEM_01
44 − FEM_10
2 − Amira
37 − FEM_03
4 − Autumn Treasure

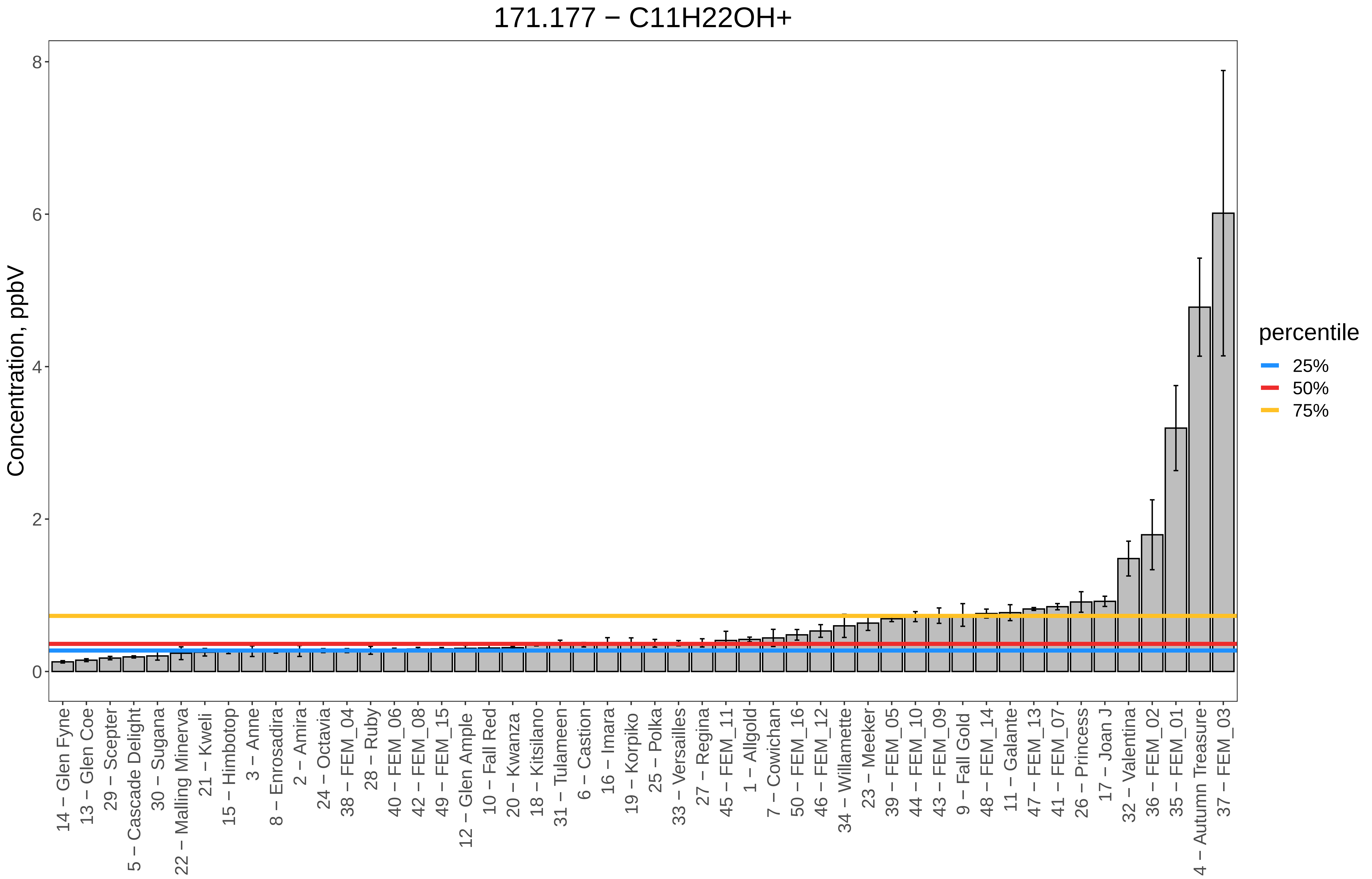

171.177 − C11H22OH+
Concentration, ppbV
0
2
4
6
8
percentile
25%
50%
75%
14 − Glen Fyne
13 − Glen Coe
29 − Scepter
5 − Cascade Delight
30 − Sugana
22 − Malling Minerva
21 − Kweli
15 − Himbotop
3 − Anne
8 − Enrosadira
2 − Amira
24 − Octavia
38 − FEM_04
28 − Ruby
40 − FEM_06
42 − FEM_08
49 − FEM_15
12 − Glen Ample
10 − Fall Red
20 − Kwanza
18 − Kitsilano
31 − Tulameen
6 − Castion
16 − Imara
19 − Korpiko
25 − Polka
33 − Versailles
27 − Regina
45 − FEM_11
1 − Allgold
7 − Cowichan
50 − FEM_16
46 − FEM_12
34 − Willamette
23 − Meeker
39 − FEM_05
44 − FEM_10
43 − FEM_09
9 − Fall Gold
48 − FEM_14
11 − Galante
47 − FEM_13
41 − FEM_07
26 − Princess
17 − Joan J
32 − Valentina
36 − FEM_02
35 − FEM_01
4 − Autumn Treasure
37 − FEM_03

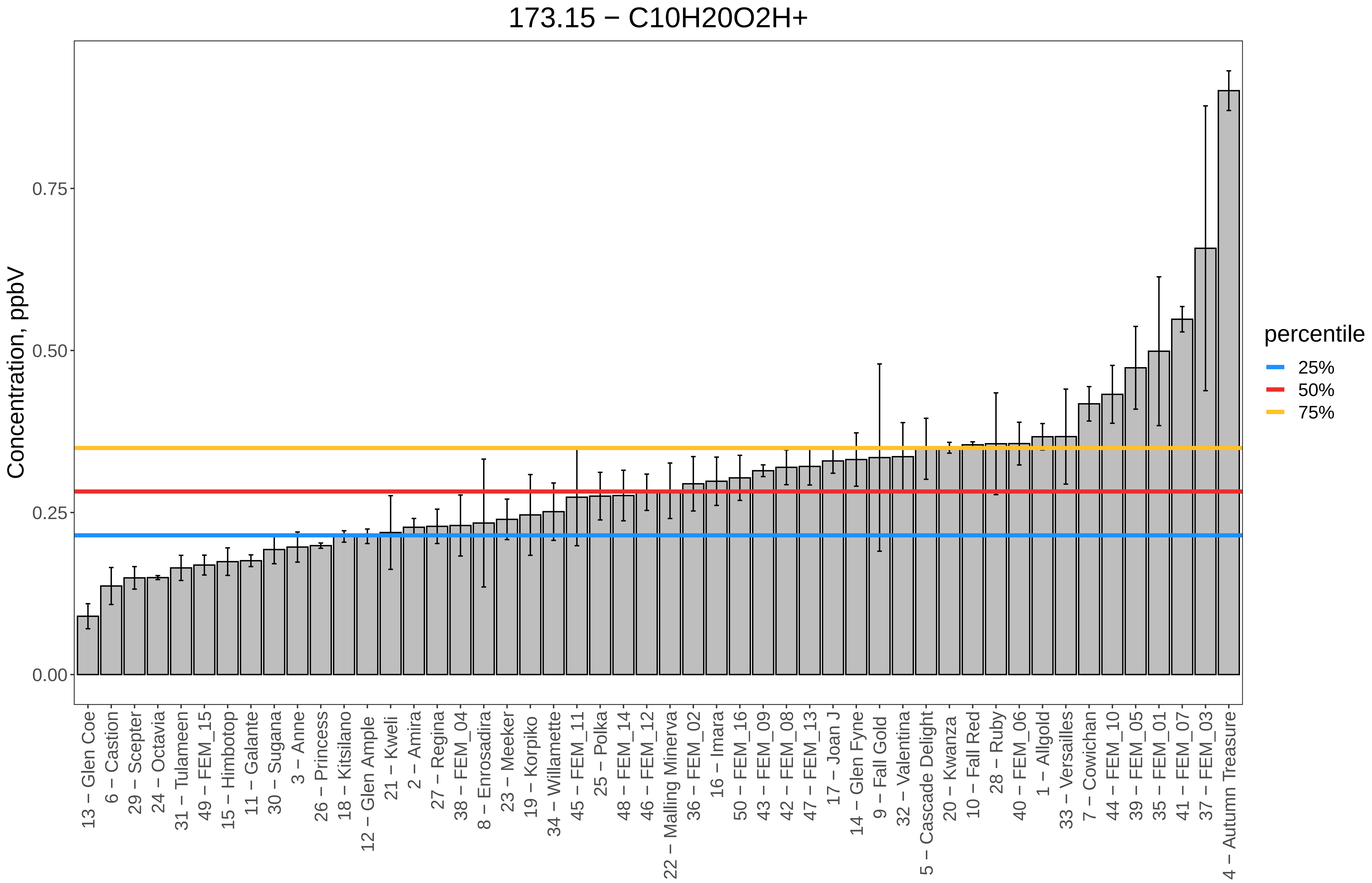
173.15 − C10H20O2H+
Concentration, ppbV
0.00
0.25
0.50
0.75
percentile
25%
50%
75%
13 − Glen Coe
6 − Castion
29 − Scepter
24 − Octavia
31 − Tulameen
49 − FEM_15
15 − Himbotop
11 − Galante
30 − Sugana
3 − Anne
26 − Princess
18 − Kitsilano
12 − Glen Ample
21 − Kweli
2 − Amira
27 − Regina
38 − FEM_04
8 − Enrosadira
23 − Meeker
19 − Korpiko
34 − Willamette
45 − FEM_11
25 − Polka
48 − FEM_14
46 − FEM_12
22 − Malling Minerva
36 − FEM_02
16 − Imara
50 − FEM_16
43 − FEM_09
42 − FEM_08
47 − FEM_13
17 − Joan J
14 − Glen Fyne
9 − Fall Gold
32 − Valentina
5 − Cascade Delight
20 − Kwanza
10 − Fall Red
28 − Ruby
40 − FEM_06
1 − Allgold
33 − Versailles
7 − Cowichan
44 − FEM_10
39 − FEM_05
35 − FEM_01
41 − FEM_07
37 − FEM_03
4 − Autumn Treasure

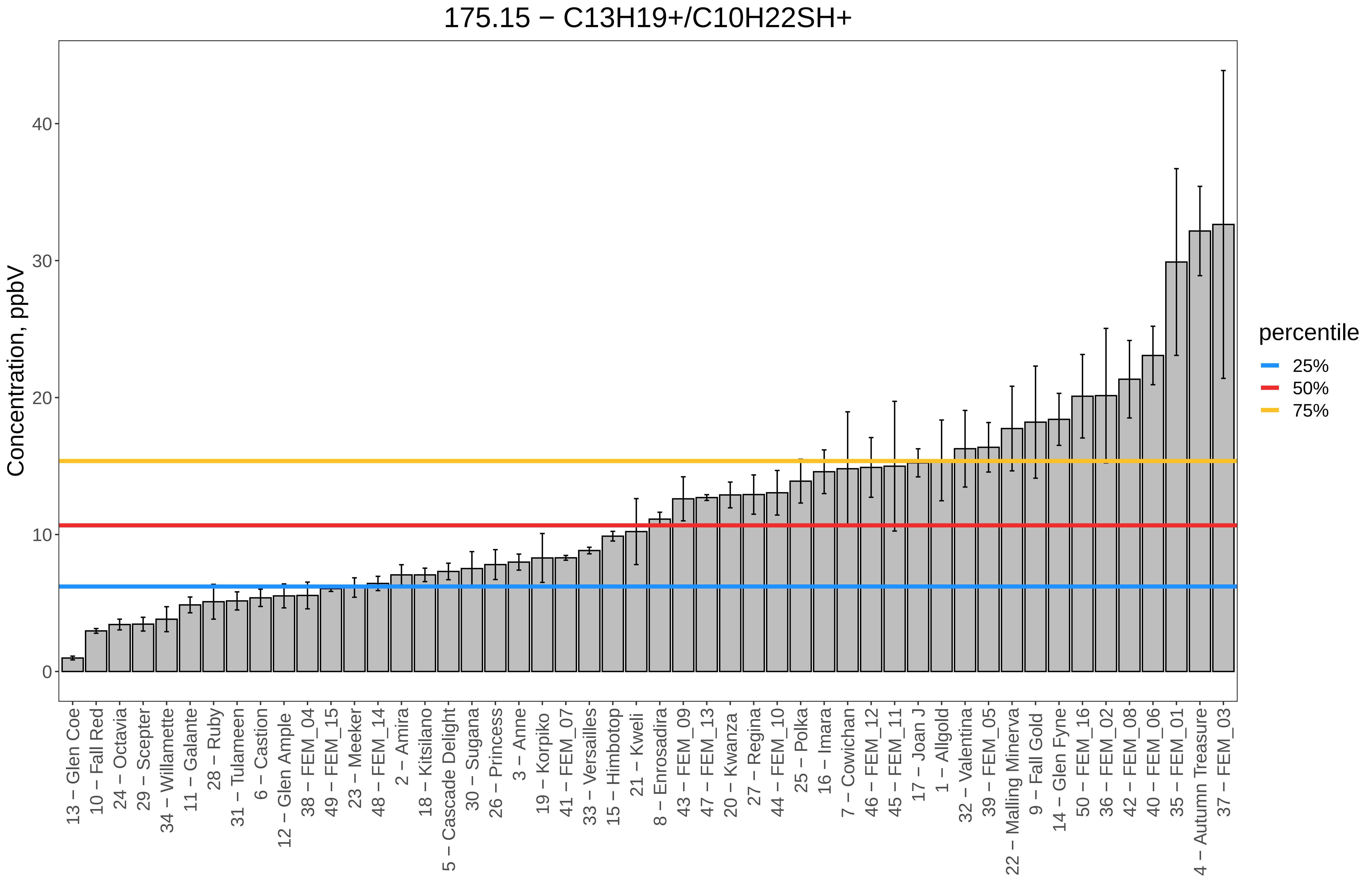

175.15 − C13H19+/C10H22SH+
Concentration, ppbV
0
10
20
30
40
percentile
25%
50%
75%
13 − Glen Coe
10 − Fall Red
24 − Octavia
29 − Scepter
34 − Willamette
11 − Galante
28 − Ruby
31 − Tulameen
6 − Castion
12 − Glen Ample
38 − FEM_04
49 − FEM_15
23 − Meeker
48 − FEM_14
2 − Amira
18 − Kitsilano
5 − Cascade Delight
30 − Sugana
26 − Princess
3 − Anne
19 − Korpiko
41 − FEM_07
33 − Versailles
15 − Himbotop
21 − Kweli
8 − Enrosadira
43 − FEM_09
47 − FEM_13
20 − Kwanza
27 − Regina
44 − FEM_10
25 − Polka
16 − Imara
7 − Cowichan
46 − FEM_12
45 − FEM_11
17 − Joan J
1 − Allgold
32 − Valentina
39 − FEM_05
22 − Malling Minerva
9 − Fall Gold
14 − Glen Fyne
50 − FEM_16
36 − FEM_02
42 − FEM_08
40 − FEM_06
35 − FEM_01
4 − Autumn Treasure
37 − FEM_03

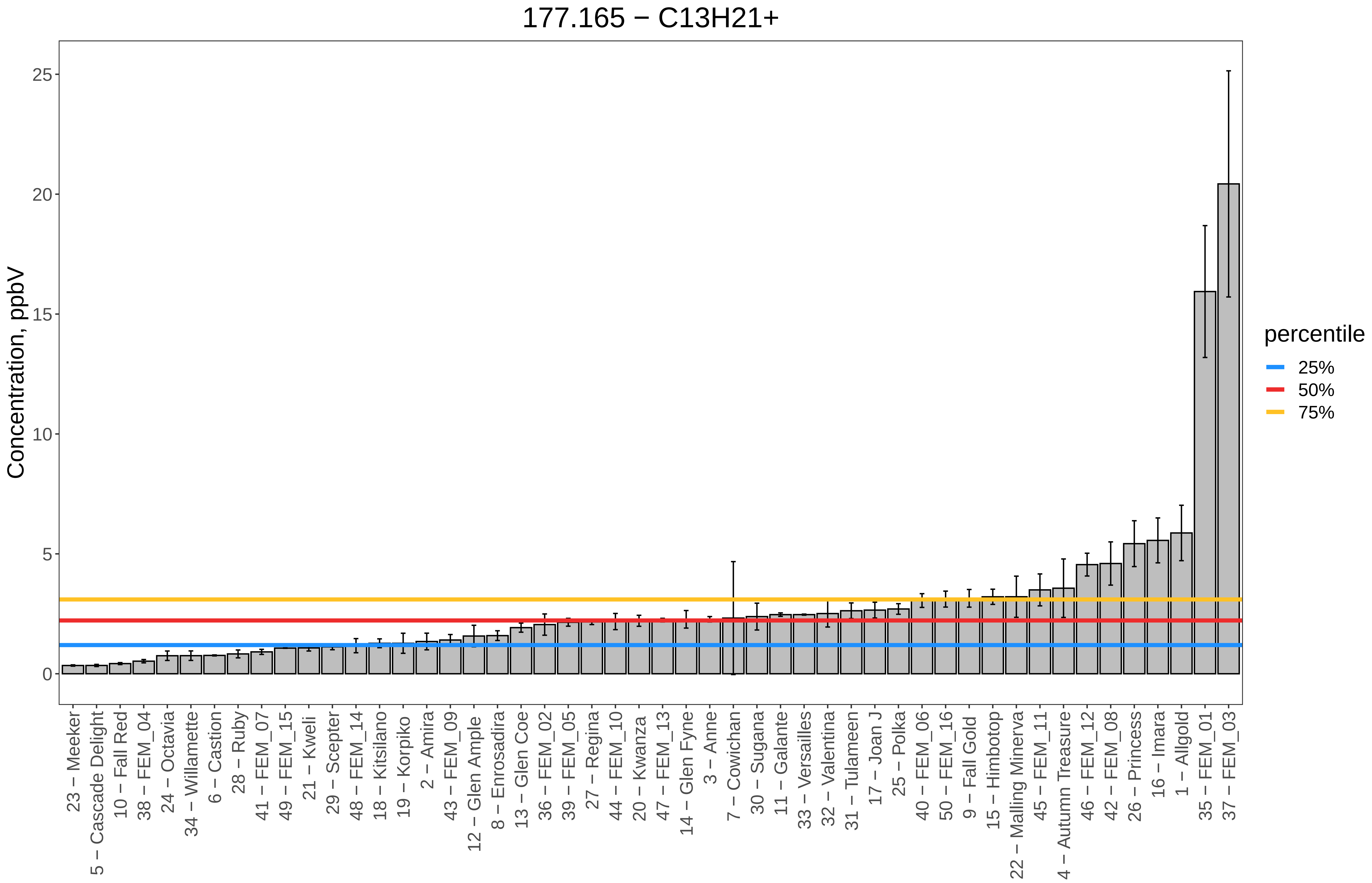

177.165 − C13H21+
Concentration, ppbV
0
5
10
15
20
25
percentile
25%
50%
75%
23 − Meeker
5 − Cascade Delight
10 − Fall Red
38 − FEM_04
24 − Octavia
34 − Willamette
6 − Castion
28 − Ruby
41 − FEM_07
49 − FEM_15
21 − Kweli
29 − Scepter
48 − FEM_14
18 − Kitsilano
19 − Korpiko
2 − Amira
43 − FEM_09
12 − Glen Ample
8 − Enrosadira
13 − Glen Coe
36 − FEM_02
39 − FEM_05
27 − Regina
44 − FEM_10
20 − Kwanza
47 − FEM_13
14 − Glen Fyne
3 − Anne
7 − Cowichan
30 − Sugana
11 − Galante
33 − Versailles
32 − Valentina
31 − Tulameen
17 − Joan J
25 − Polka
40 − FEM_06
50 − FEM_16
9 − Fall Gold
15 − Himbotop
22 − Malling Minerva
45 − FEM_11
4 − Autumn Treasure
46 − FEM_12
42 − FEM_08
26 − Princess
16 − Imara
1 − Allgold
35 − FEM_01
37 − FEM_03

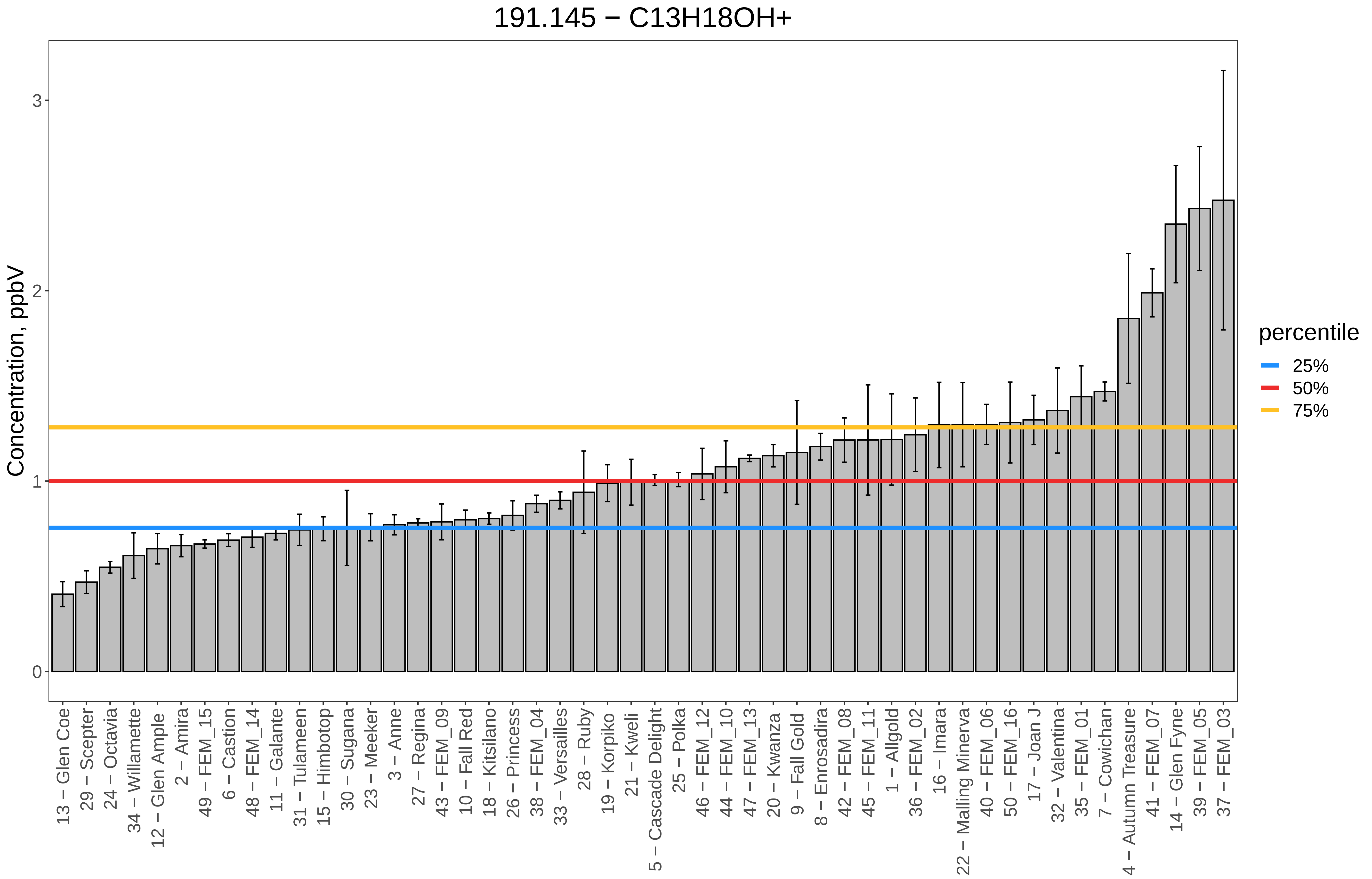

191.145 − C13H18OH+
Concentration, ppbV
0
1
2
3
percentile
25%
50%
75%
13 − Glen Coe
29 − Scepter
24 − Octavia
34 − Willamette
12 − Glen Ample
2 − Amira
49 − FEM_15
6 − Castion
48 − FEM_14
11 − Galante
31 − Tulameen
15 − Himbotop
30 − Sugana
23 − Meeker
3 − Anne
27 − Regina
43 − FEM_09
10 − Fall Red
18 − Kitsilano
26 − Princess
38 − FEM_04
33 − Versailles
28 − Ruby
19 − Korpiko
21 − Kweli
5 − Cascade Delight
25 − Polka
46 − FEM_12
44 − FEM_10
47 − FEM_13
20 − Kwanza
9 − Fall Gold
8 − Enrosadira
42 − FEM_08
45 − FEM_11
1 − Allgold
36 − FEM_02
16 − Imara
22 − Malling Minerva
40 − FEM_06
50 − FEM_16
17 − Joan J
32 − Valentina
35 − FEM_01
7 − Cowichan
4 − Autumn Treasure
41 − FEM_07
14 − Glen Fyne
39 − FEM_05
37 − FEM_03

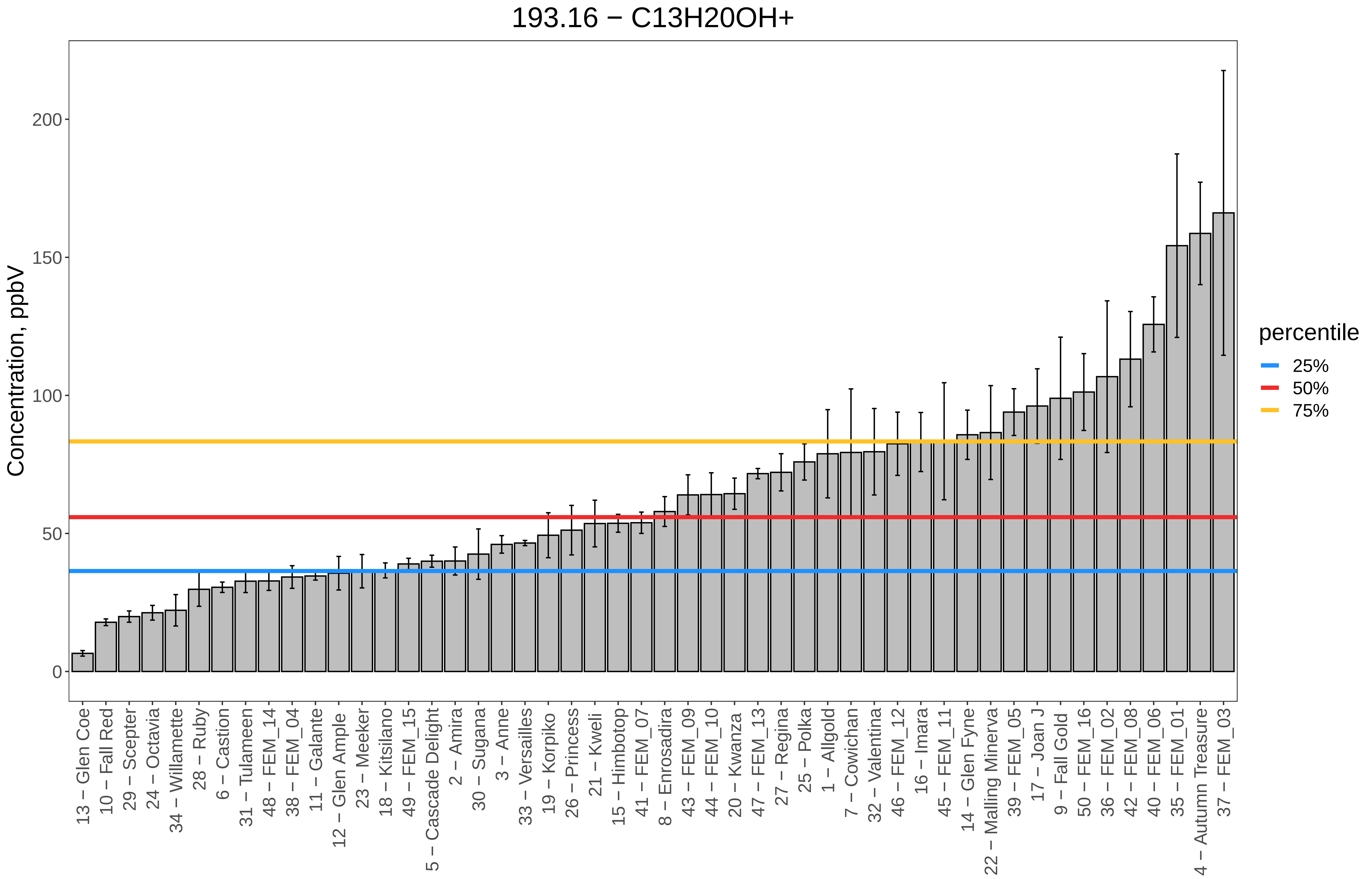
193.16 − C13H20OH+
Concentration, ppbV
0
50
100
150
200
percentile
25%
50%
75%
13 − Glen Coe
10 − Fall Red
29 − Scepter
24 − Octavia
34 − Willamette
28 − Ruby
6 − Castion
31 − Tulameen
48 − FEM_14
38 − FEM_04
11 − Galante
12 − Glen Ample
23 − Meeker
18 − Kitsilano
49 − FEM_15
5 − Cascade Delight
2 − Amira
30 − Sugana
3 − Anne
33 − Versailles
19 − Korpiko
26 − Princess
21 − Kweli
15 − Himbotop
41 − FEM_07
8 − Enrosadira
43 − FEM_09
44 − FEM_10
20 − Kwanza
47 − FEM_13
27 − Regina
25 − Polka
1 − Allgold
7 − Cowichan
32 − Valentina
46 − FEM_12
16 − Imara
45 − FEM_11
14 − Glen Fyne
22 − Malling Minerva
39 − FEM_05
17 − Joan J
9 − Fall Gold
50 − FEM_16
36 − FEM_02
42 − FEM_08
40 − FEM_06
35 − FEM_01
4 − Autumn Treasure
37 − FEM_03

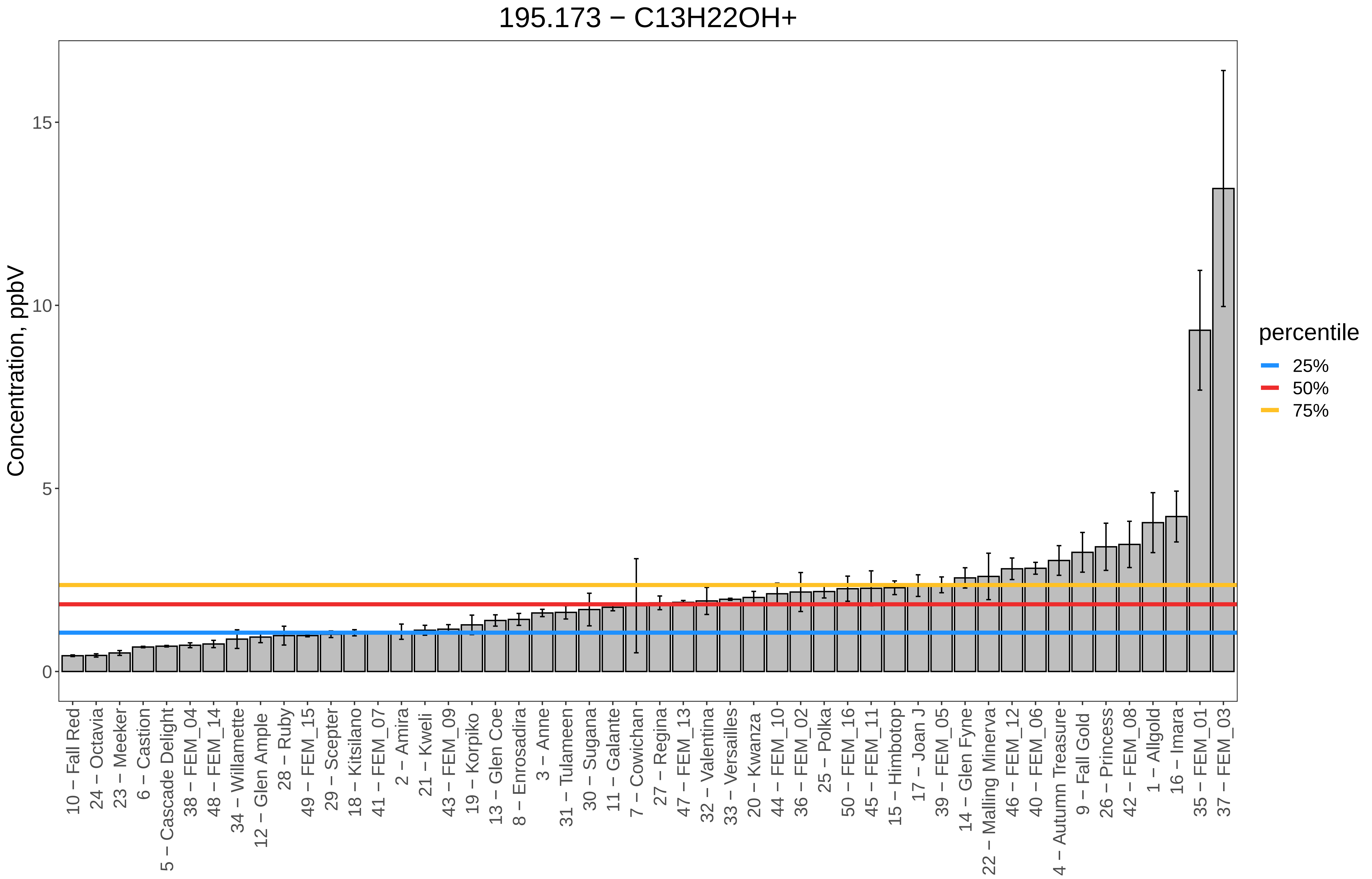

195.173 − C13H22OH+
Concentration, ppbV
0
5
10
15
percentile
25%
50%
75%
10 − Fall Red
24 − Octavia
23 − Meeker
6 − Castion
5 − Cascade Delight
38 − FEM_04
48 − FEM_14
34 − Willamette
12 − Glen Ample
28 − Ruby
49 − FEM_15
29 − Scepter
18 − Kitsilano
41 − FEM_07
2 − Amira
21 − Kweli
43 − FEM_09
19 − Korpiko
13 − Glen Coe
8 − Enrosadira
3 − Anne
31 − Tulameen
30 − Sugana
11 − Galante
7 − Cowichan
27 − Regina
47 − FEM_13
32 − Valentina
33 − Versailles
20 − Kwanza
44 − FEM_10
36 − FEM_02
25 − Polka
50 − FEM_16
45 − FEM_11
15 − Himbotop
17 − Joan J
39 − FEM_05
14 − Glen Fyne
22 − Malling Minerva
46 − FEM_12
40 − FEM_06
4 − Autumn Treasure
9 − Fall Gold
26 − Princess
42 − FEM_08
1 − Allgold
16 − Imara
35 − FEM_01
37 − FEM_03

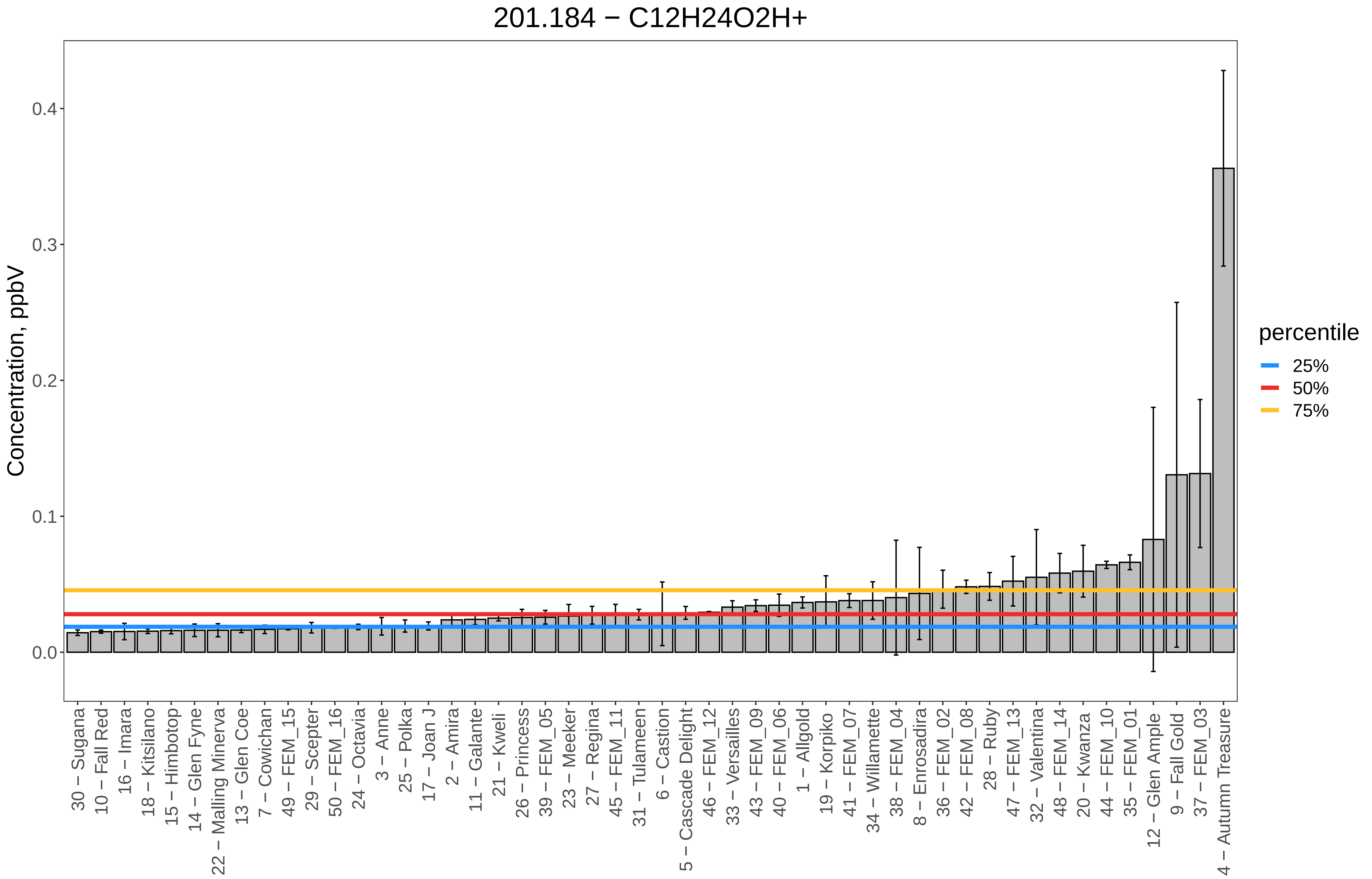

201.184 − C12H24O2H+
Concentration, ppbV
0.0
0.1
0.2
0.3
0.4
percentile
25%
50%
75%
30 − Sugana
10 − Fall Red
16 − Imara
18 − Kitsilano
15 − Himbotop
14 − Glen Fyne
22 − Malling Minerva
13 − Glen Coe
7 − Cowichan
49 − FEM_15
29 − Scepter
50 − FEM_16
24 − Octavia
3 − Anne
25 − Polka
17 − Joan J
2 − Amira
11 − Galante
21 − Kweli
26 − Princess
39 − FEM_05
23 − Meeker
27 − Regina
45 − FEM_11
31 − Tulameen
6 − Castion
5 − Cascade Delight
46 − FEM_12
33 − Versailles
43 − FEM_09
40 − FEM_06
1 − Allgold
19 − Korpiko
41 − FEM_07
34 − Willamette
38 − FEM_04
8 − Enrosadira
36 − FEM_02
42 − FEM_08
28 − Ruby
47 − FEM_13
32 − Valentina
48 − FEM_14
20 − Kwanza
44 − FEM_10
35 − FEM_01
12 − Glen Ample
9 − Fall Gold
37 − FEM_03
4 − Autumn Treasure

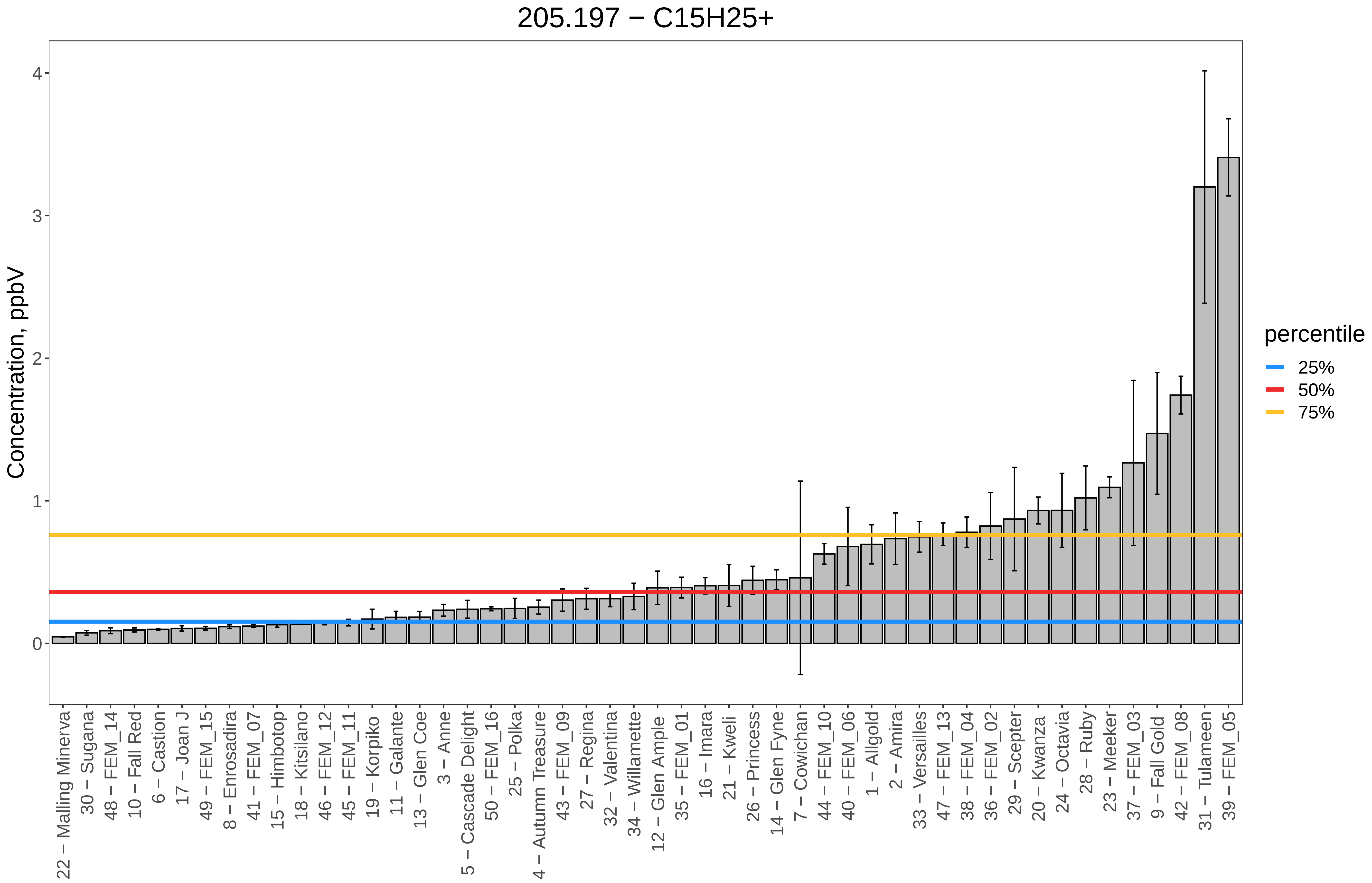

205.197 − C15H25+
Concentration, ppbV
0
1
2
3
4
percentile
25%
50%
75%
22 − Malling Minerva
30 − Sugana
48 − FEM_14
10 − Fall Red
6 − Castion
17 − Joan J
49 − FEM_15
8 − Enrosadira
41 − FEM_07
15 − Himbotop
18 − Kitsilano
46 − FEM_12
45 − FEM_11
19 − Korpiko
11 − Galante
13 − Glen Coe
3 − Anne
5 − Cascade Delight
50 − FEM_16
25 − Polka
4 − Autumn Treasure
43 − FEM_09
27 − Regina
32 − Valentina
34 − Willamette
12 − Glen Ample
35 − FEM_01
16 − Imara
21 − Kweli
26 − Princess
14 − Glen Fyne
7 − Cowichan
44 − FEM_10
40 − FEM_06
1 − Allgold
2 − Amira
33 − Versailles
47 − FEM_13
38 − FEM_04
36 − FEM_02
29 − Scepter
20 − Kwanza
24 − Octavia
28 − Ruby
23 − Meeker
37 − FEM_03
9 − Fall Gold
42 − FEM_08
31 − Tulameen
39 − FEM_05

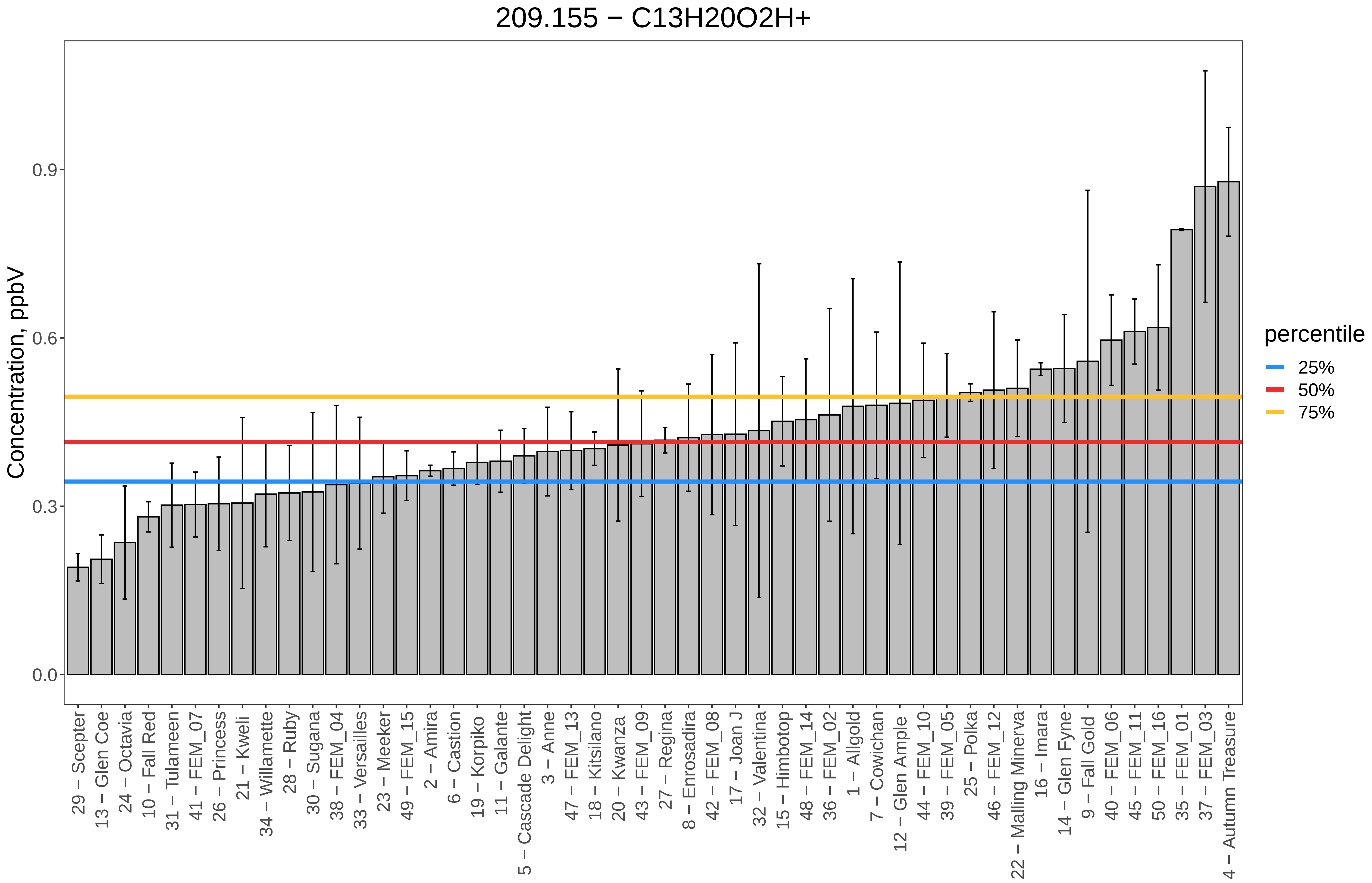
209.155 − C13H20O2H+
Concentration, ppbV
0.0
0.3
0.6
0.9
percentile
25%
50%
75%
29 − Scepter
13 − Glen Coe
24 − Octavia
10 − Fall Red
31 − Tulameen
41 − FEM_07
26 − Princess
21 − Kweli
34 − Willamette
28 − Ruby
30 − Sugana
38 − FEM_04
33 − Versailles
23 − Meeker
49 − FEM_15
2 − Amira
6 − Castion
19 − Korpiko
11 − Galante
5 − Cascade Delight
3 − Anne
47 − FEM_13
18 − Kitsilano
20 − Kwanza
43 − FEM_09
27 − Regina
8 − Enrosadira
42 − FEM_08
17 − Joan J
32 − Valentina
15 − Himbotop
48 − FEM_14
36 − FEM_02
1 − Allgold
7 − Cowichan
12 − Glen Ample
44 − FEM_10
39 − FEM_05
25 − Polka
46 − FEM_12
22 − Malling Minerva
16 − Imara
14 − Glen Fyne
9 − Fall Gold
40 − FEM_06
45 − FEM_11
50 − FEM_16
35 − FEM_01
37 − FEM_03
4 − Autumn Treasure
