# Supplementary figures and images for: Volatilomics of raspberry fruit germplasm by combining chromatographic and direct-injection mass spectrometric techniques
Source: Front Mol Biosci. 2023 Apr 13;10:1155564. doi: 10.3389/fmolb.2023.1155564 (PMC10133483; doi:10.3389/fmolb.2023.1155564)

Unknown 1 RT 19.115

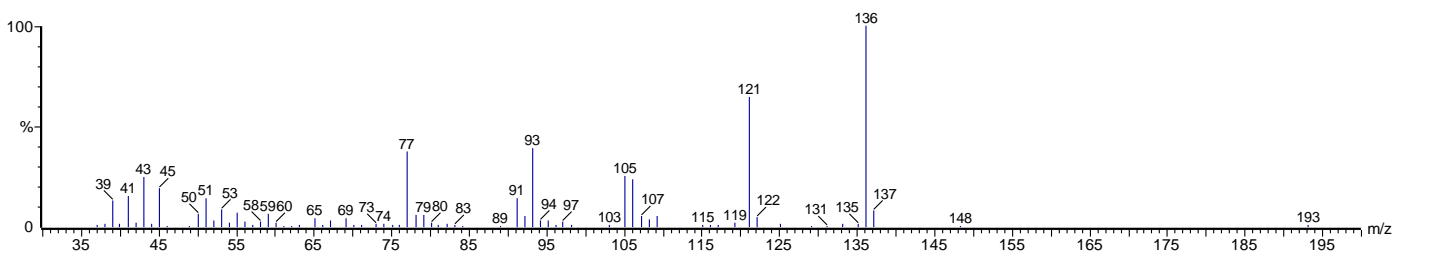

Unknown 2 RT 24.683

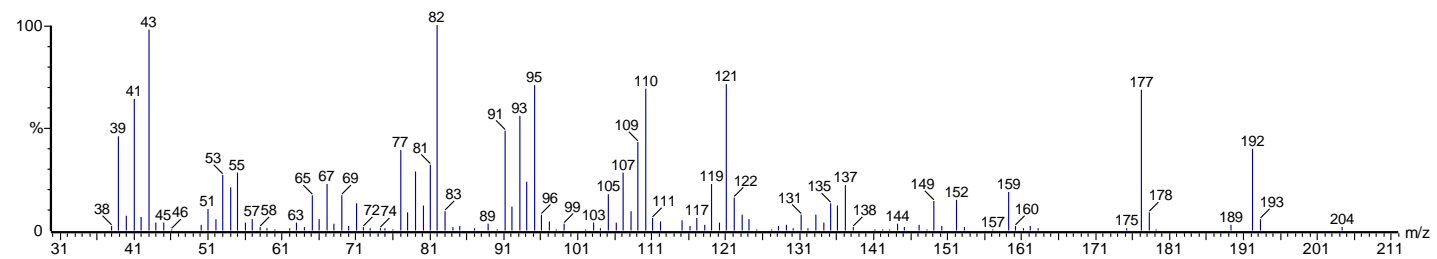

Unknown 3 RT 25.627

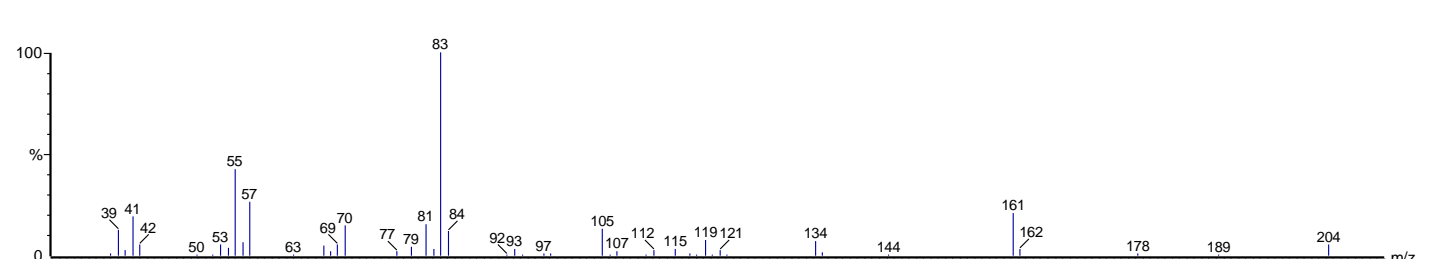

Unknown 4 RT 25.808

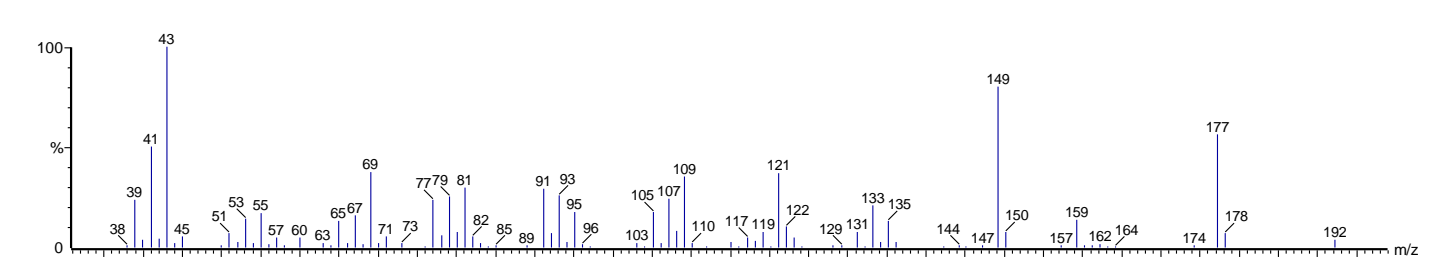

Unknown 5 RT 29.550

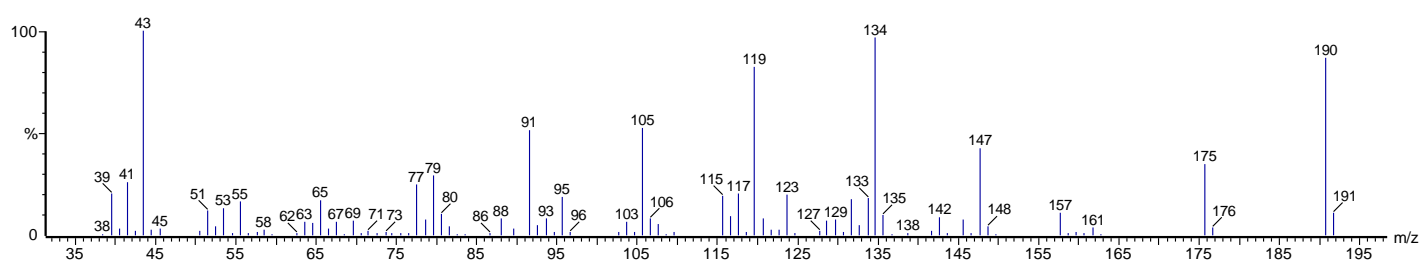

Unknown 6 RT 29.748

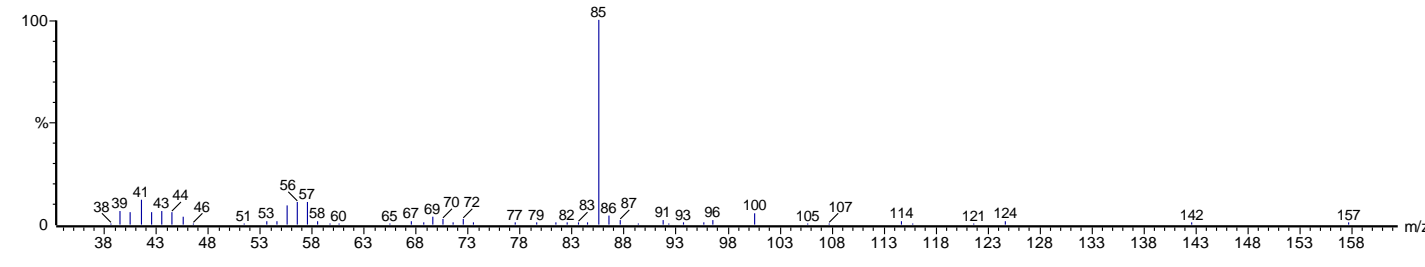

Unknown 7 RT 31.249

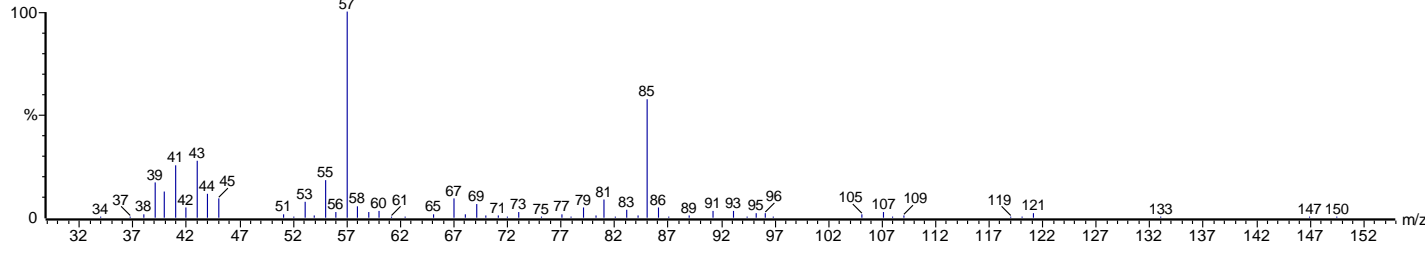

Supplement: Supplementary file 5 [file Image4.PDF]

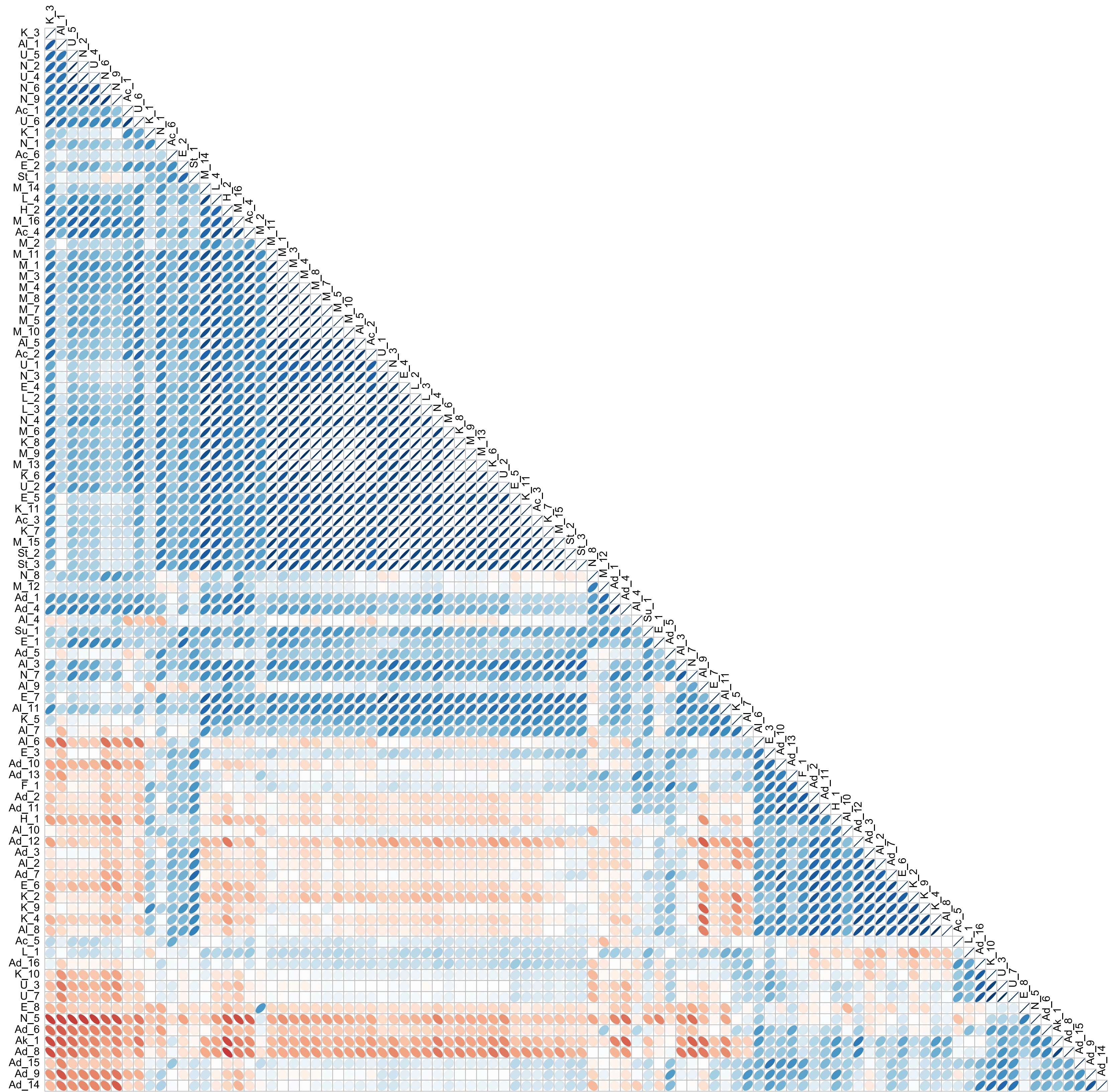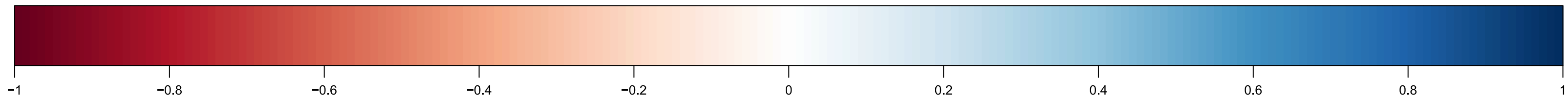

Supplement: Supplementary file 7 [file Image3.PDF]

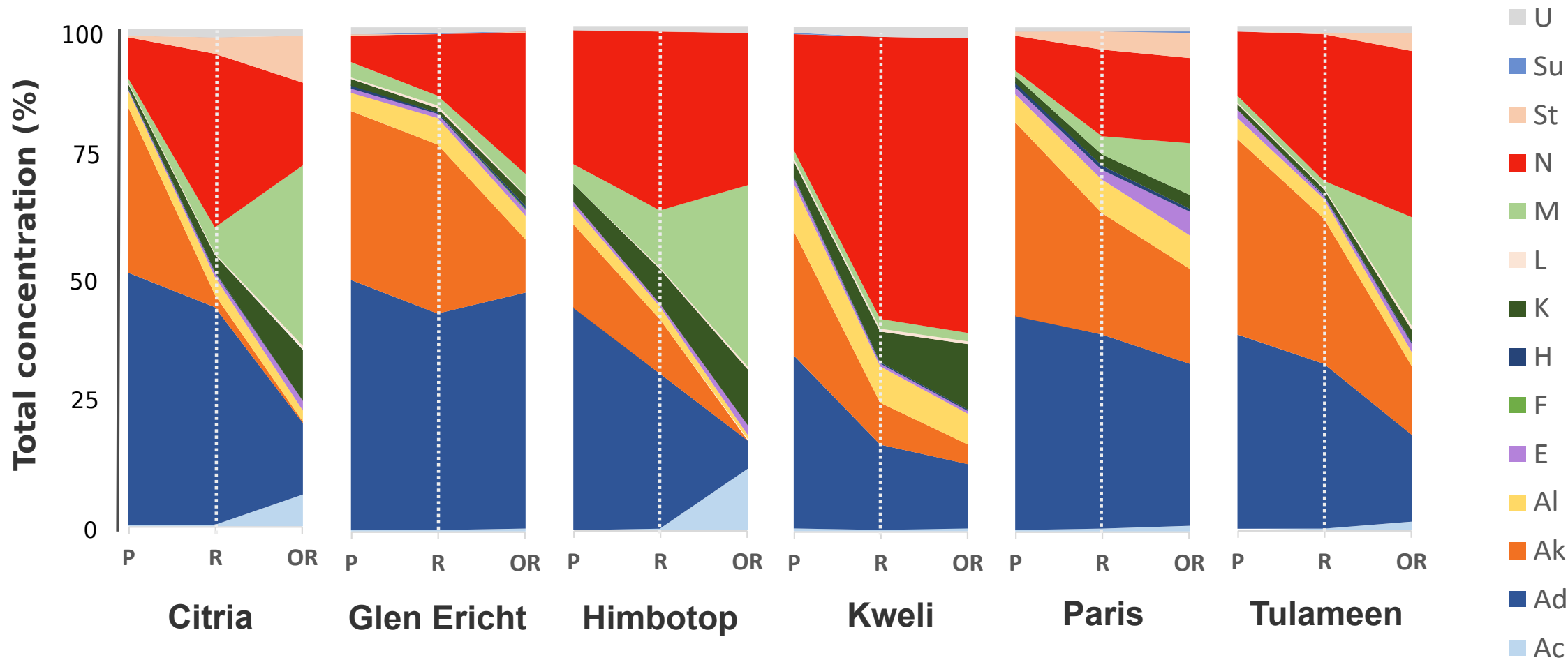

Supplement: Supplementary file 11 [file Image1.PDF]
