# Supplementary material for: Volatilomics of raspberry fruit germplasm by combining chromatographic and direct-injection mass spectrometric techniques
Source: Front Mol Biosci. 2023 Apr 13;10:1155564. doi: 10.3389/fmolb.2023.1155564 (PMC10133483; doi:10.3389/fmolb.2023.1155564)
Supplement: Supplementary file 6 [file Image2.PDF]

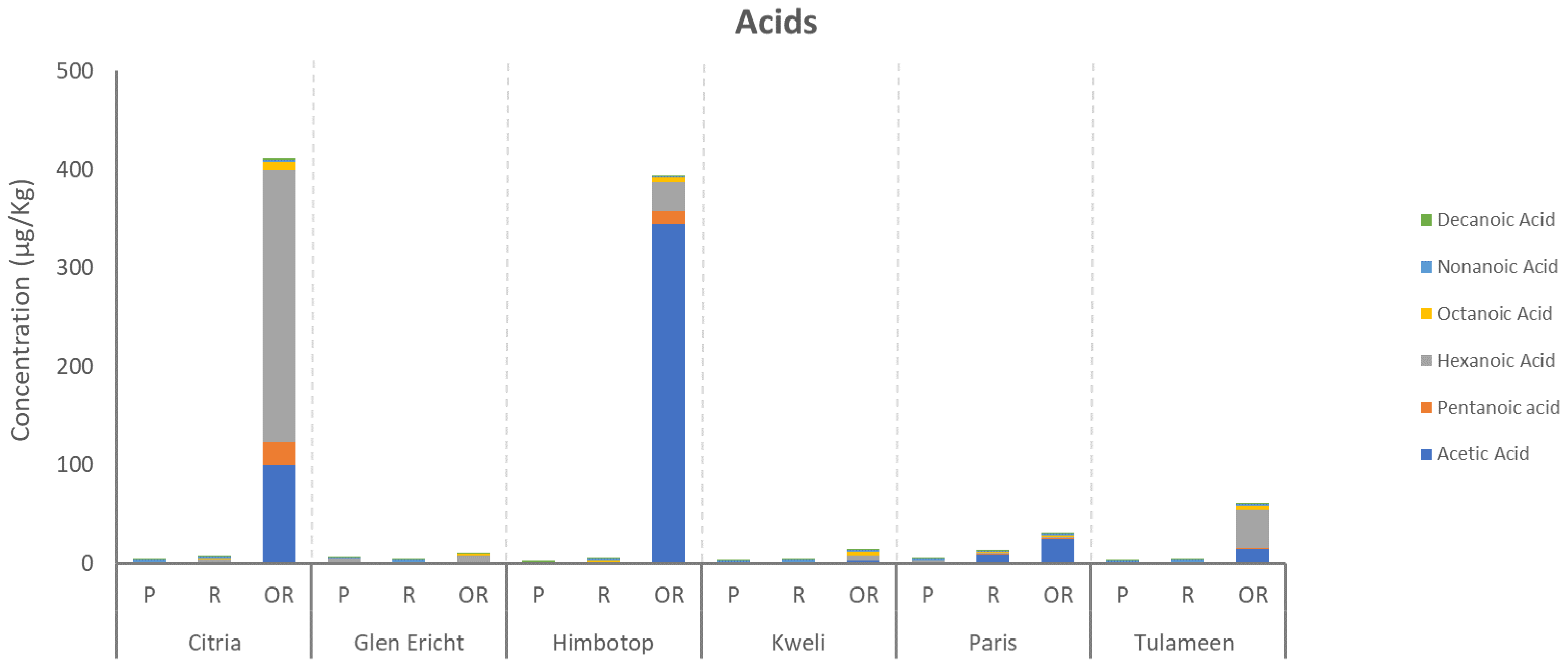
Acids
Concentration (µg/Kg)
500
400
300
200
100
0
P
R
OR
Citria
Glen Ericht
Himbotop
Kweli
Paris
Tulameen
Decanoic Acid
Nonanoic Acid
Octanoic Acid
Hexanoic Acid
Pentanoic acid
Acetic Acid

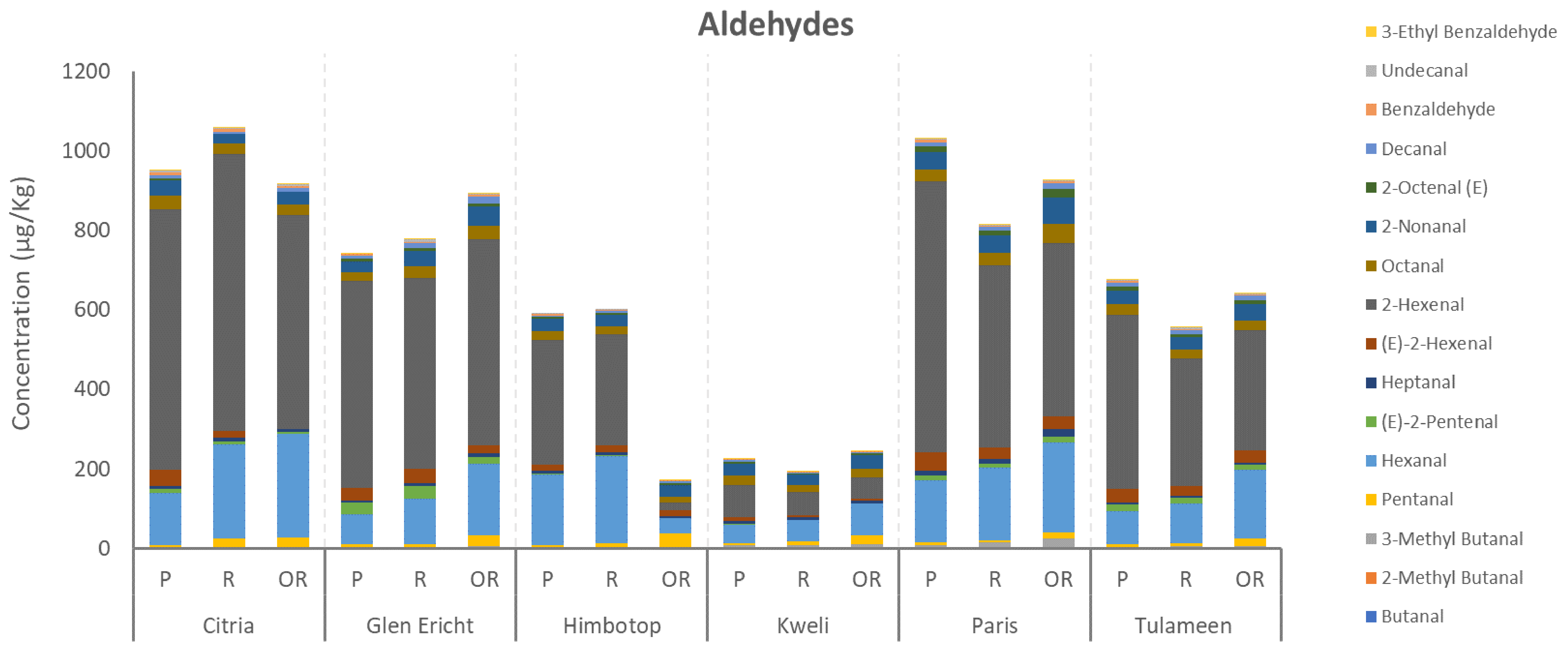
Aldehydes
Concentration (µg/Kg)
1200
1000
800
600
400
200
0
P
R
OR
Citria
Glen Ericht
Himbotop
Kweli
Paris
Tulameen
3-Ethyl Benzaldehyde
Undecanal
Benzaldehyde
Decanal
2-Octenal (E)
2-Nonanal
Octanal
2-Hexenal
(E)-2-Hexenal
Heptanal
(E)-2-Pentenal
Hexanal
Pentanal
3-Methyl Butanal
2-Methyl Butanal
Butanal

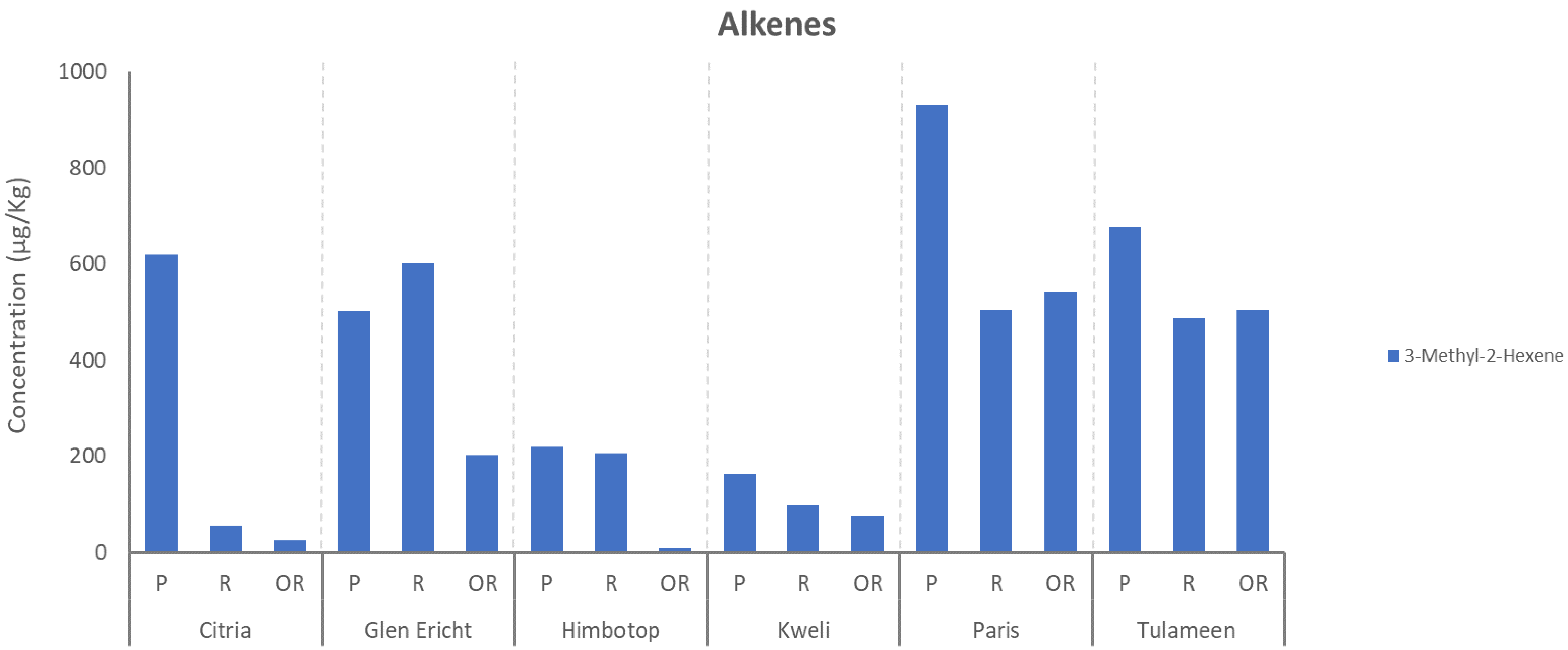
Alkenes
Concentration (μg/Kg)
1000
800
600
400
200
0
P
R
OR
Citria
Glen Ericht
Himbotop
Kweli
Paris
Tulameen
3-Methyl-2-Hexene

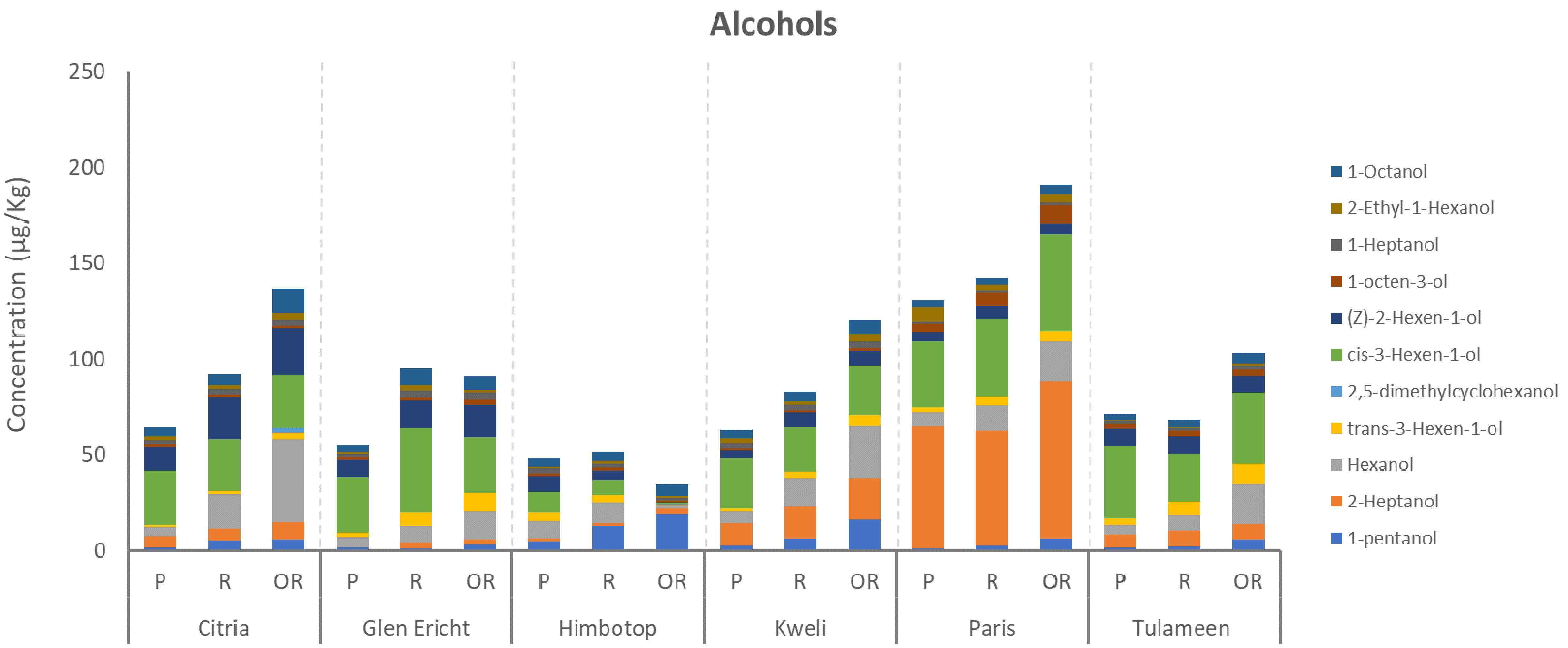
Alcohols
Concentration (µg/Kg)
0
50
100
150
200
250
P
R
OR
Citria
Glen Ericht
Himbotop
Kweli
Paris
Tulameen
1-Octanol
2-Ethyl-1-Hexanol
1-Heptanol
1-octen-3-ol
(Z)-2-Hexen-1-ol
cis-3-Hexen-1-ol
2,5-dimethylcyclohexanol
trans-3-Hexen-1-ol
Hexanol
2-Heptanol
1-pentanol

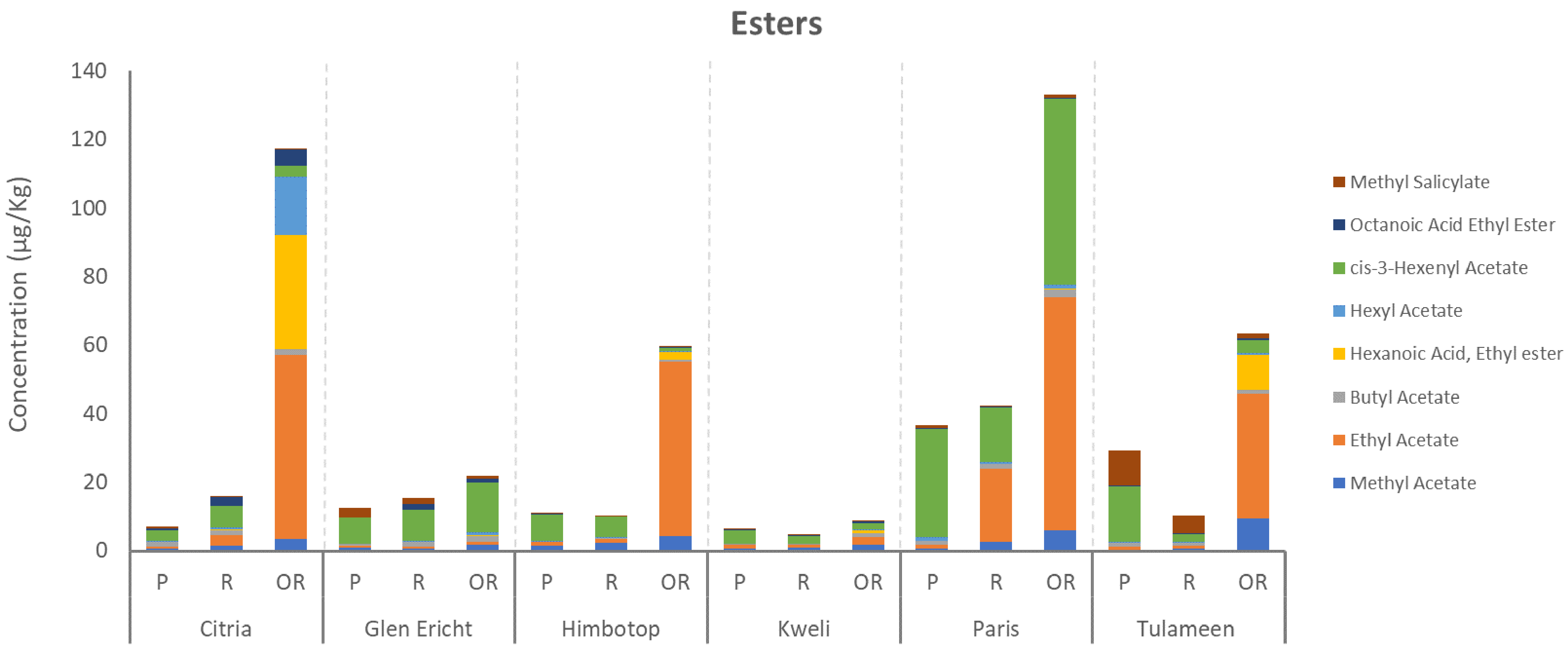
Esters
Concentration (μg/Kg)
140
120
100
80
60
40
20
0
P
R
OR
Citria
Glen Ericht
Himbotop
Kweli
Paris
Tulameen
Methyl Salicylate
Octanoic Acid Ethyl Ester
cis-3-Hexenyl Acetate
Hexyl Acetate
Hexanoic Acid, Ethyl ester
Butyl Acetate
Ethyl Acetate
Methyl Acetate

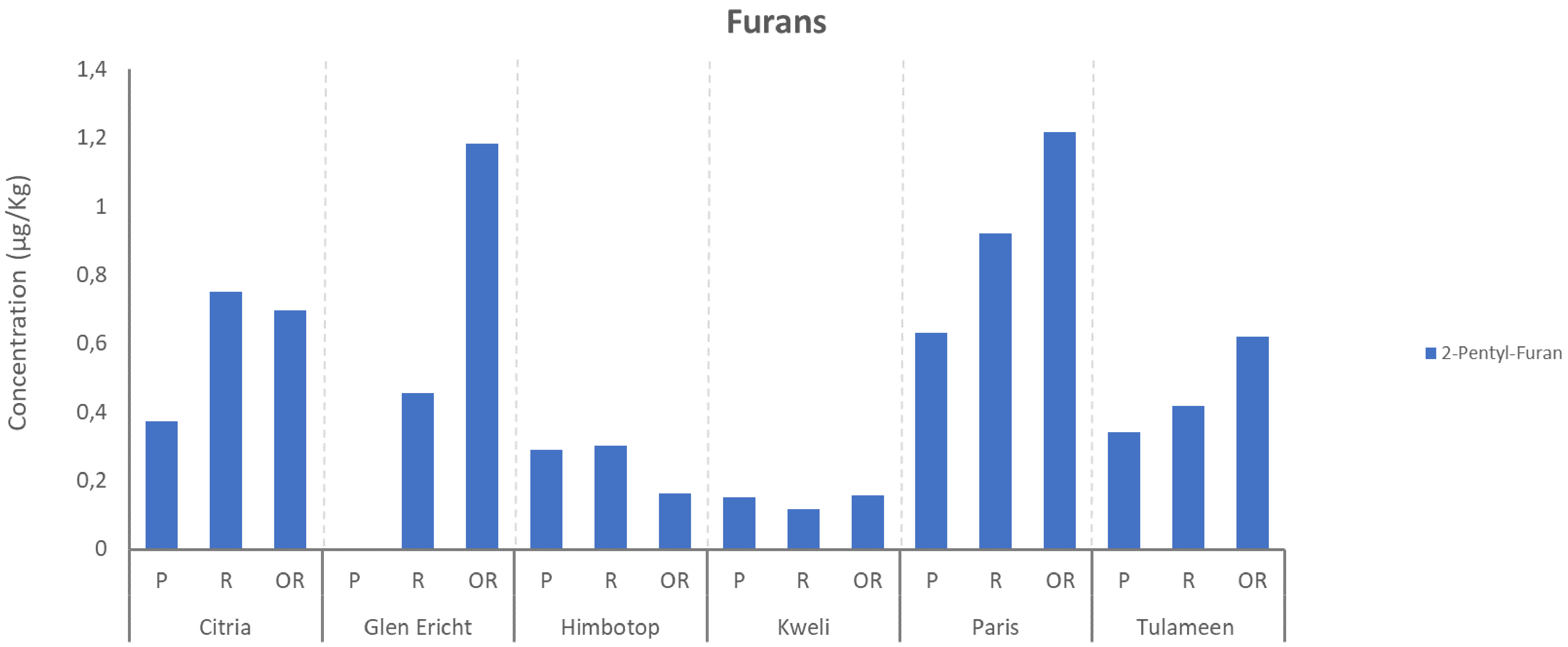
Furans
Concentration (µg/Kg)
1,4
1,2
1
0,8
0,6
0,4
0,2
0
P
R
OR
Citria
Glen Ericht
Himbotop
Kweli
Paris
Tulameen
2-Pentyl-Furan

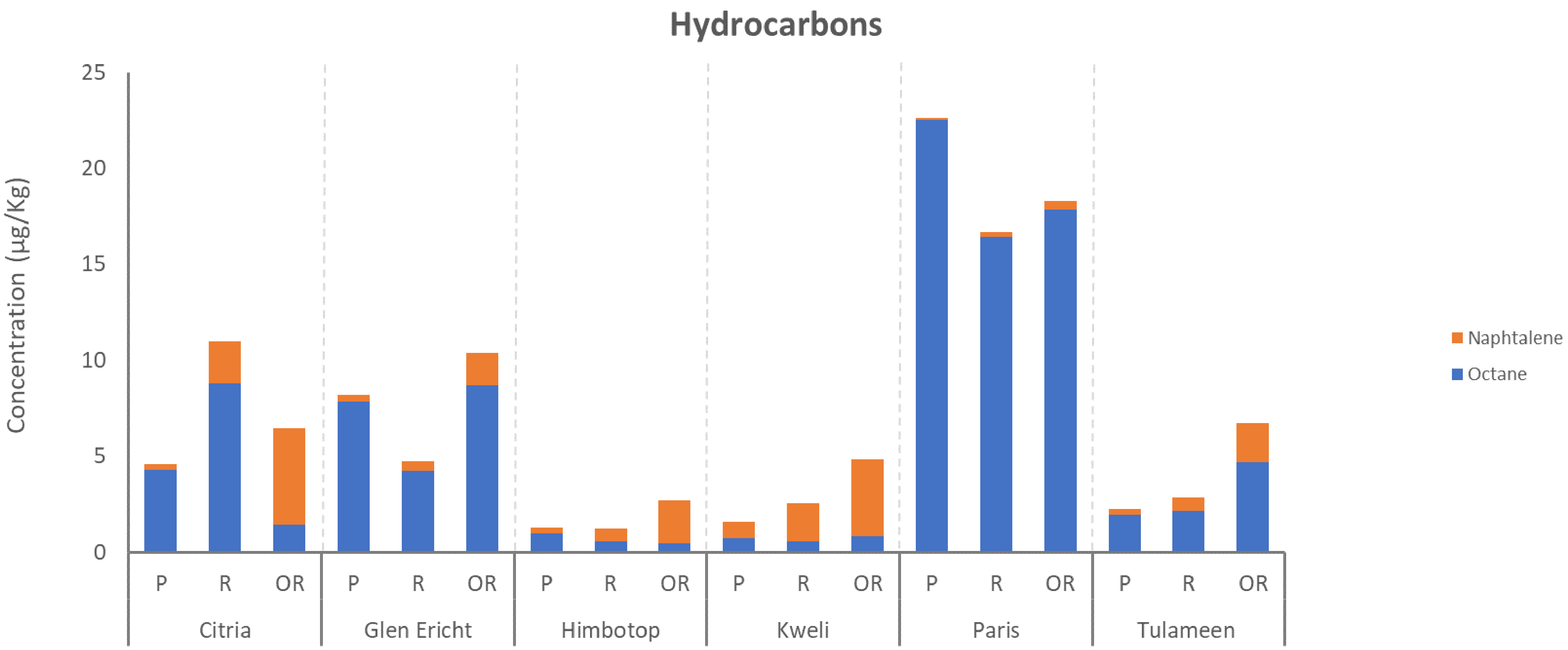
Hydrocarbons
Concentration (µg/Kg)
25
20
15
10
5
0
P
R
OR
Citria
Glen Ericht
Himbotop
Kweli
Paris
Tulameen
Naphtalene
Octane

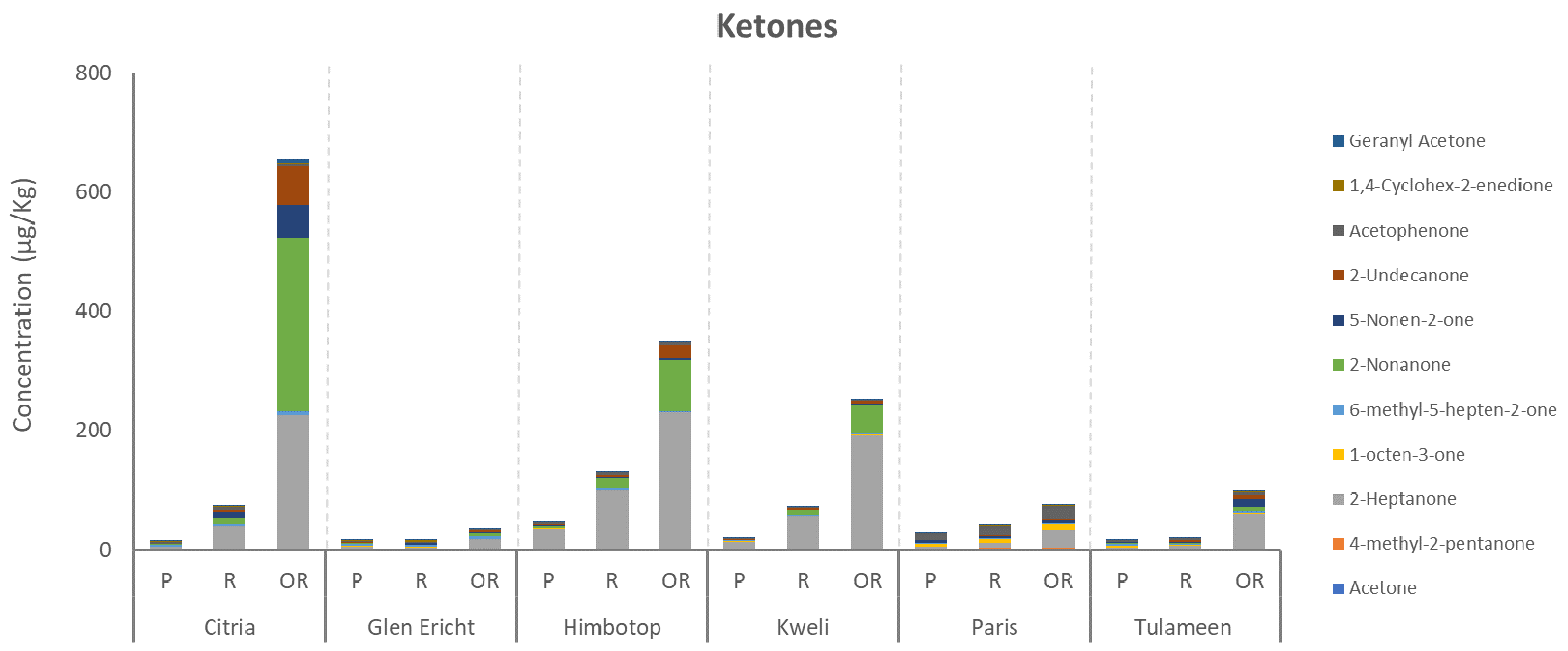
Ketones
Concentration (µg/Kg)
800
600
400
200
0
P
R
OR
Citria
Glen Ericht
Himbotop
Kweli
Paris
Tulameen
Geranyl Acetone
1,4-Cyclohex-2-enedione
Acetophenone
2-Undecanone
5-Nonen-2-one
2-Nonanone
6-methyl-5-hepten-2-one
1-octen-3-one
2-Heptanone
4-methyl-2-pentanone
Acetone

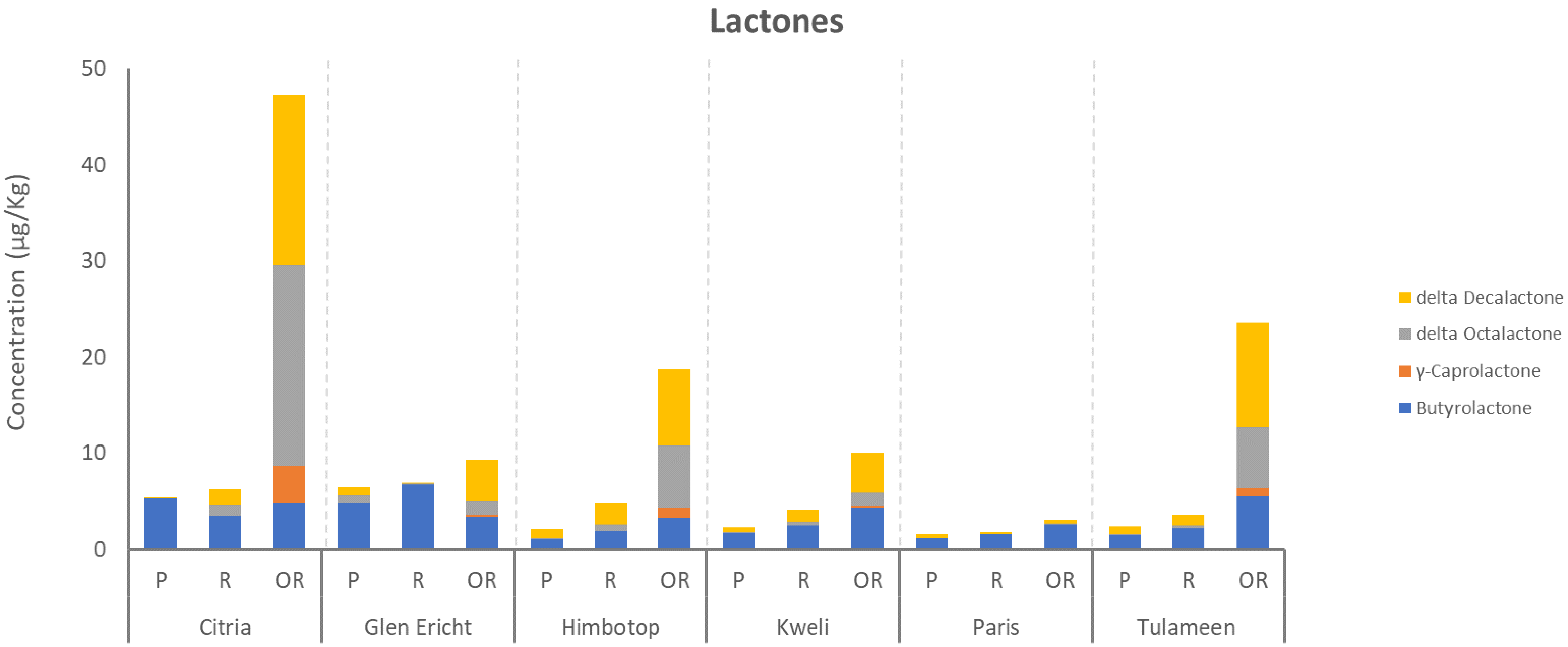
Lactones
Concentration (µg/Kg)
0
10
20
30
40
50
P
R
OR
Citria
Glen Ericht
Himbotop
Kweli
Paris
Tulameen
delta Decalactone
delta Octalactone
γ-Caprolactone
Butyrolactone

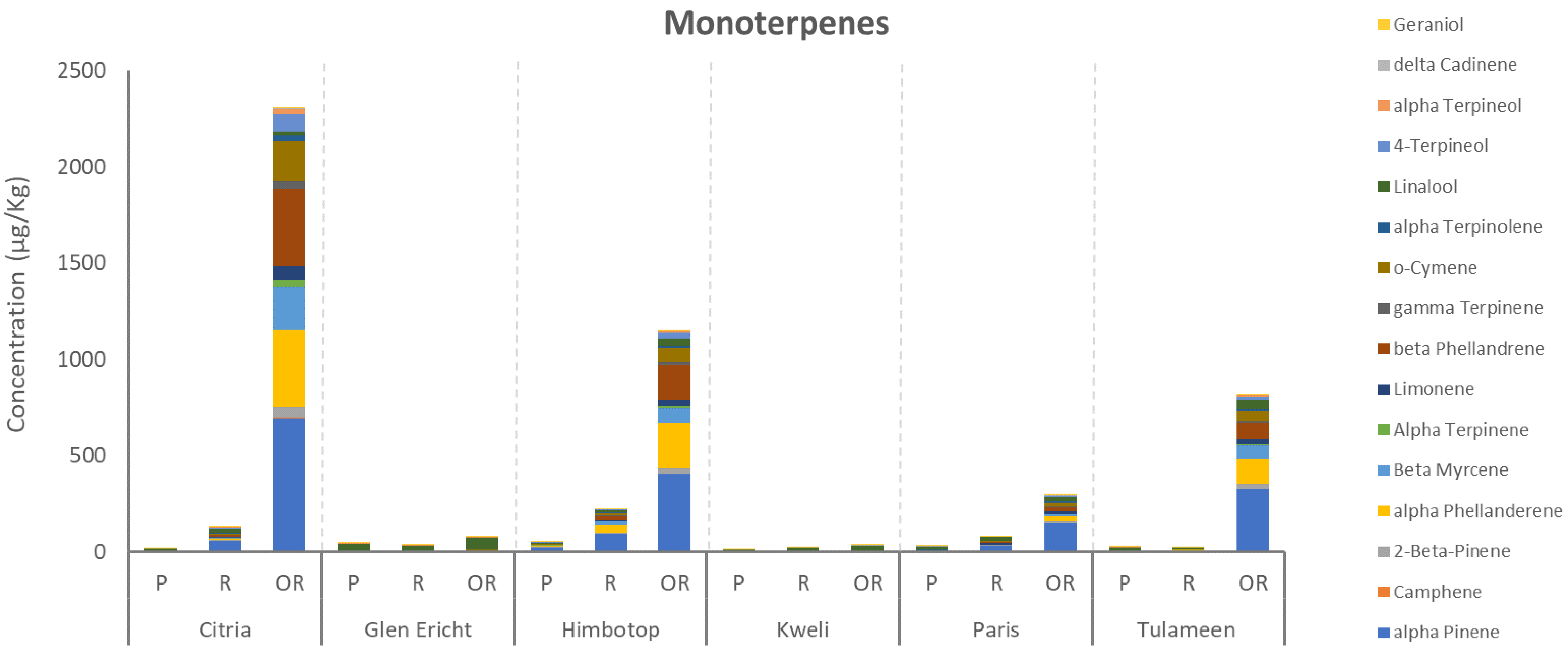

Monoterpenes
Concentration (µg/Kg)
2500
2000
1500
1000
500
0
P
R
OR
Citria
Glen Ericht
Himbotop
Kweli
Paris
Tulameen
Geraniol
delta Cadinene
alpha Terpineol
4-Terpineol
Linalool
alpha Terpinolene
o-Cymene
gamma Terpinene
beta Phellandrene
Limonene
Alpha Terpinene
Beta Myrcene
alpha Phellanderene
2-Beta-Pinene
Camphene
alpha Pinene

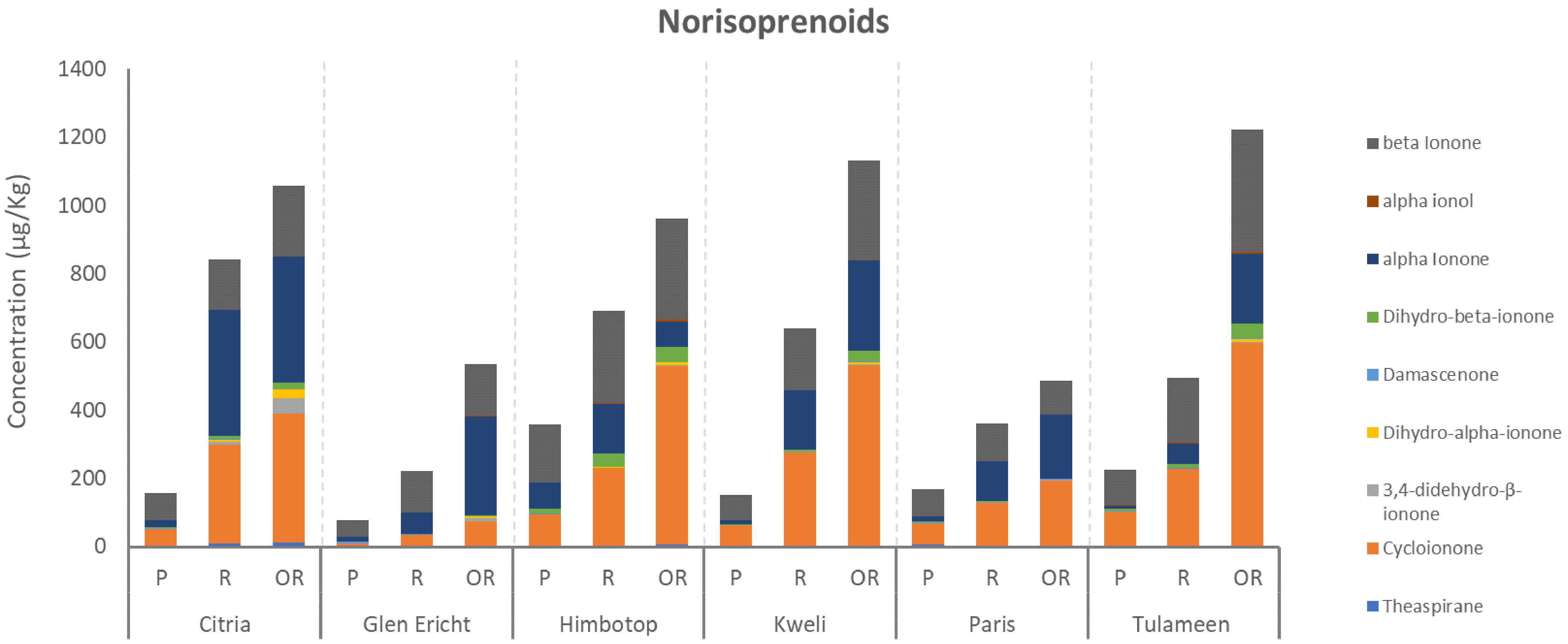
Norisoprenoids
Concentration (µg/Kg)
1400
1200
1000
800
600
400
200
0
P
R
OR
Citria
Glen Ericht
Himbotop
Kweli
Paris
Tulameen
beta Ionone
alpha ionol
alpha Ionone
Dihydro-beta-ionone
Damascenone
Dihydro-alpha-ionone
3,4-didehydro-β-ionone
Cycloionone
Theaspirane

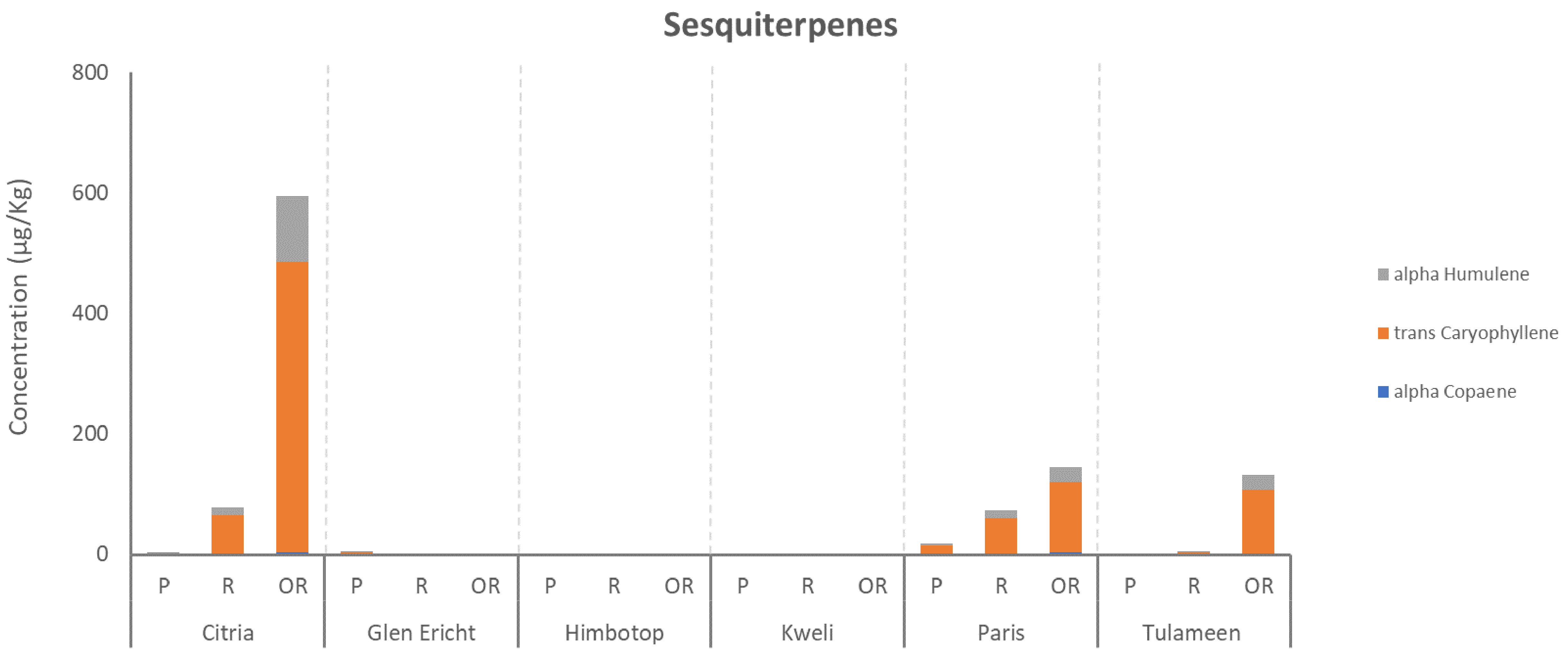
Sesquiterpenes
Concentration (µg/Kg)
800
600
400
200
0
P
R
OR
Citria
Glen Ericht
Himbotop
Kweli
Paris
Tulameen
alpha Humulene
trans Caryophyllene
alpha Copaene

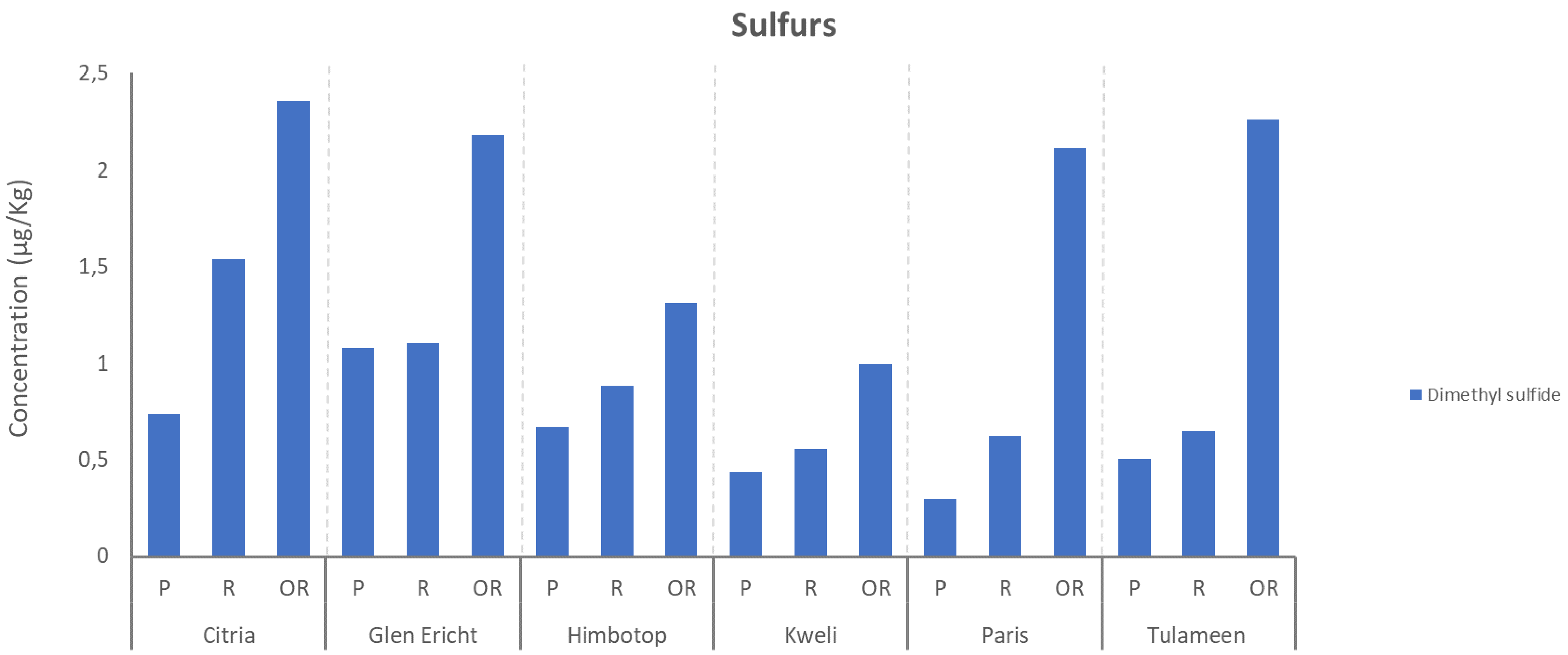
Sulfurs
Concentration (µg/Kg)
2,5
2
1,5
1
0,5
0
P
R
OR
Citria
Glen Ericht
Himbotop
Kweli
Paris
Tulameen
Dimethyl sulfide

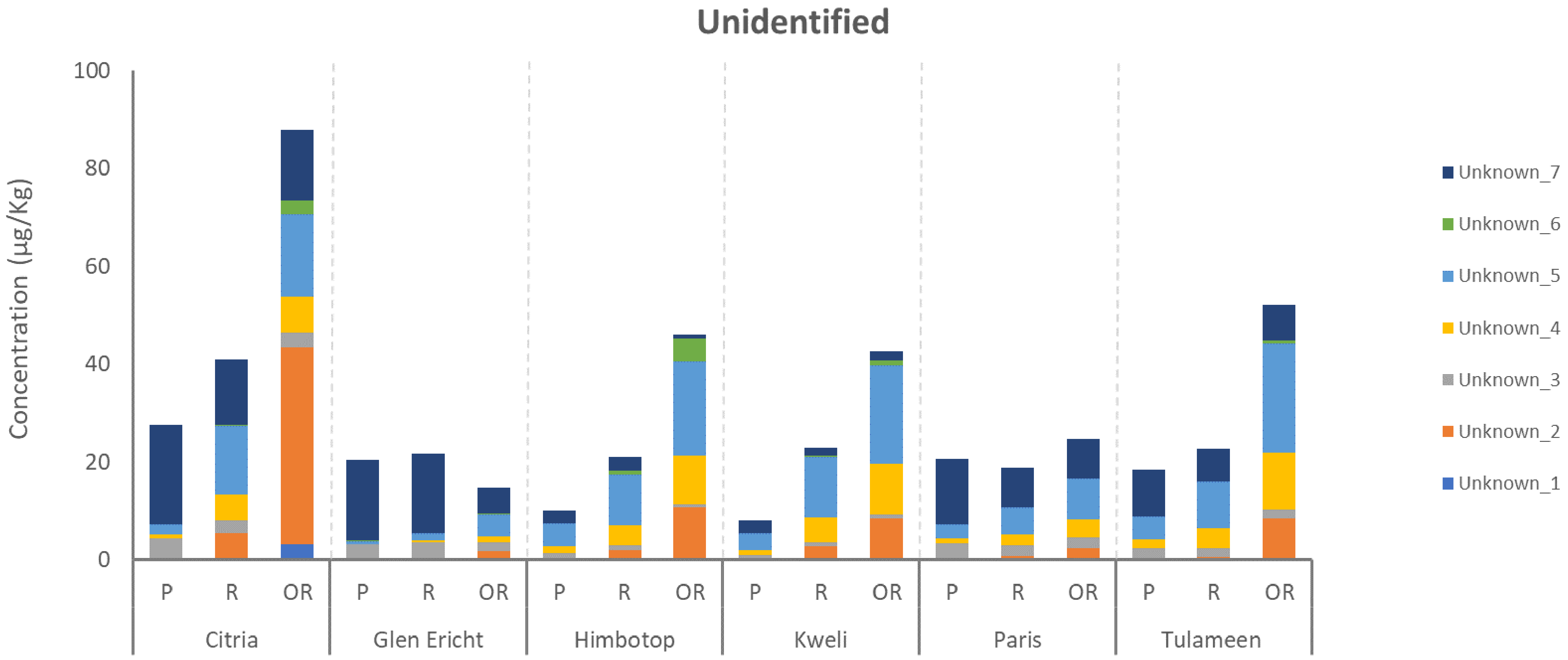
Unidentified
Concentration (µg/Kg)
100
80
60
40
20
0
P
R
OR
Citria
Glen Ericht
Himbotop
Kweli
Paris
Tulameen
Unknown_7
Unknown_6
Unknown_5
Unknown_4
Unknown_3
Unknown_2
Unknown_1
