# Supplementary material for: Volatilomics of raspberry fruit germplasm by combining chromatographic and direct-injection mass spectrometric techniques
Source: Front Mol Biosci. 2023 Apr 13;10:1155564. doi: 10.3389/fmolb.2023.1155564 (PMC10133483; doi:10.3389/fmolb.2023.1155564)

QualySort 1.2

Choose xlsx file

Browse... Analysis_lamponi_germoplasm_QualiSort.xlsx

Upload complete

Calculate

Select All

Choose parameters

- [ ] 27.0406
- [ ] 28.0184 CHNH+
- [ ] 28.0313 C2H4+
- [ ] 31.0181 CH2OH+
- [ ] 33.0329 CH4OH+
- [ ] 34.9952 H2SH+
- [ ] 39.0233 C3H3+
- [ ] 41.0385 C3H5+
- [ ] 42.0104
- [ ] 42.023
- [ ] 43.0175 C2H3O+
- [ ] 43.0538 C3H7+
- [ ] 45.0318 C2H4OH+
- [ ] 47.0482 C2H6OH+
- [ ] 49.0108 CH4SH+
- [ ] 53.0028
- [ ] 53.0394 C4H5+
- [ ] 55.0154 C3H2OH+
- [ ] 55.0543 C4H7+
- [ ] 57.0335 C3H4OH+
- [ ] 57.0699 C4H9+
- [ ] 59.0483 C3H6OH+
- [ ] 61.0274 C2H4O2H+
- [ ] 63.0267 C2H6SH+
- [ ] 65.0227
- [ ] 67.0547 C5H7+
- [ ] 69.0347 C4H4OH+
- [ ] 69.07 C5H9+
- [ ] 71.0492 C4H6OH+
- [ ] 71.0855 C5H11+
- [ ] 73.0292 C3H4O2H+
- [ ] 73.065 C4H8OH+
- [ ] 75.043 C3H6O2H+
- [ ] 77.0127 C2H4OSH+
- [ ] 79.056 C6H7+
- [ ] 81.0699 C6H9+
- [ ] 83.0529 C5H6OH+
- [x] 83.0857 C6H11+
- [x] 85.0651 C5H8OH+
- [ ] 85.0999 C6H13+
- [ ] 87.044 C4H6O2H+
- [ ] 87.0805 C5H10OH+
- [ ] 89.0586 C4H8O2H+
- [ ] 91.0595 C4H10SH+
- [ ] 93.0386 C6H4OH+
- [ ] 93.0705 C7H9+
- [ ] 94.9576 C2H3O2ClH+
- [ ] 95.0151 C2H6O2SH+
- [ ] 95.0478 C6H6OH+
- [ ] 95.0862 C7H11+
- [ ] 97.0287 C5H4O2H+
- [ ] 97.0643 C6H8OH+
- [ ] 97.1019 C7H13+
- [ ] 99.0092
- [x] 99.0806 C6H10OH+
- [ ] 101.0617 C5H8O2H+
- [x] 101.096 C6H12OH+
- [ ] 103.0749 C5H10O2H+
- [ ] 105.0378 C7H4OH+/C4H8OSH+
- [ ] 105.0619 C8H9+
- [ ] 107.0533 C7H6OH+
- [ ] 107.0822 C8H11+
- [ ] 108.9575
- [ ] 109.0188
- [ ] 109.0676 C7H8OH+
- [ ] 109.1022 C8H13+
- [ ] 111.0813 C7H10OH+
- [ ] 111.1174 C8H15+
- [ ] 113.0279
- [ ] 113.0605 C6H8O2H+
- [ ] 113.0967 C7H12OH+
- [ ] 113.1317
- [ ] 115.0793 C6H10O2H+
- [x] 115.1125 C7H14OH+
- [ ] 117.0463
- [ ] 117.0913 C6H12O2H+
- [ ] 119.039 C8H6OH+
- [ ] 119.0946 C9H11+/C6H14SH+
- [ ] 121.0412
- [ ] 121.0672 C8H8OH+
- [ ] 121.098 C9H13+
- [ ] 122.0644
- [ ] 123.0456 C4H10O2SH+
- [ ] 123.0824 C8H10OH+
- [ ] 123.118 C9H15+
- [ ] 125.0601
- [ ] 125.0971 C8H12OH+
- [ ] 125.133 C9H17+
- [ ] 127.0394 C6H6O3H+
- [ ] 127.0747 C7H10O2H+
- [ ] 127.1127 C8H14OH+
- [ ] 129.0591 C10H9+
- [ ] 129.0919 C7H12O2H+
- [ ] 129.1281 C8H16OH+
- [ ] 131.1073 C7H14O2H+
- [ ] 133.1067 C10H13+
- [ ] 135.0755 C9H10OH+
- [ ] 135.1145 C10H15+
- [ ] 137.0586 C8H8O2H+
- [ ] 137.0931 C9H12OH+
- [x] 137.1336 C10H17+
- [ ] 139.0409 C7H6O3H+
- [ ] 139.0756 C8H10O2H+
- [ ] 139.1134 C9H14OH+
- [ ] 141.0546 C7H8O3H+
- [ ] 141.0938 C8H12O2H+
- [ ] 141.1286 C9H16OH+
- [ ] 143.1084 C8H14O2H+
- [x] 143.1442 C9H18OH+
- [ ] 145.1227 C8H16O2H+
- [ ] 147.1285 C11H15+
- [ ] 149.1 C10H12OH+
- [ ] 149.1316 C11H17+
- [ ] 151.1134 C10H14OH+
- [ ] 151.1497 C11H19+
- [ ] 153.0569 C8H8O3H+
- [ ] 153.1283 C10H16OH+
- [ ] 155.1092 C9H14O2H+
- [ ] 155.1445 C10H18OH+
- [ ] 157.1236 C9H16O2H+
- [ ] 157.1597 C10H20OH+
- [ ] 159.1396 C9H18O2H+
- [ ] 165.1296 C11H16OH+
- [ ] 167.1442 C11H18OH+
- [ ] 169.1607 C11H20OH+
- [ ] 171.141 C10H18O2H+
- [ ] 171.1769 C11H22OH+
- [ ] 173.15 C10H20O2H+
- [ ] 175.1501 C13H19+/C10H22SH+
- [ ] 177.1654 C13H21+
- [ ] 191.145 C13H18OH+
- [x] 193.1602 C13H20OH+
- [ ] 195.1727 C13H22OH+
- [ ] 201.1842 C12H24O2H+
- [x] 205.1971 C15H25+
- [ ] 209.1545 C13H20O2H+

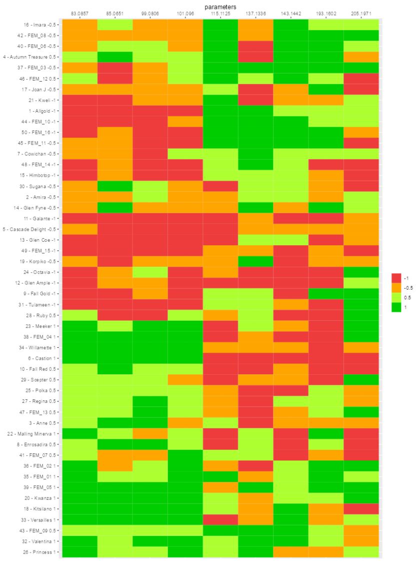

Supplement: Supplementary file 10 [file Image7.PDF]
